# The clinical practice guideline for the management of ARDS in Japan

**DOI:** 10.1186/s40560-017-0222-3

**Published:** 2017-07-25

**Authors:** Satoru Hashimoto, Masamitsu Sanui, Moritoki Egi, Shinichiro Ohshimo, Junji Shiotsuka, Ryutaro Seo, Ryoma Tanaka, Yu Tanaka, Yasuhiro Norisue, Yoshiro Hayashi, Eishu Nango, ARDS clinical practice guideline committee from the Japanese Society of Respiratory Care Medicine and the Japanese Society of Intensive Care Medicine, Yoshitaka Aoki, Kohkichi Andoh, Yusuke Iizuka, Hitoshi Imaizumi, Satoshi Okamori, Motoshi Kainuma, Jun Kataoka, Tetsuro Kamo, Atsushi Kawaguchi, Junji Kumasawa, Kiyoyasu Kurahashi, Kunihiko Kooguchi, Yutaka Kondo, Masaaki Sakuraya, Akira Shimoyama, So Suzuki, Hiroyuki Suzuki, Motohiro Sekino, Mikio Nakajima, Tetsuro Nishimura, Tatsuma Fukuda, Jun Makino, Ryoichi Miyashita, Ryutaro Moriwaki, Hideto Yasuda, Shigenori Yoshitake, Yumi Yamashita, Yoshiko Nakagawa, Takaaki Suzuki, Toshiyuki Aokage, Kimitaka Tajimi, Hidemichi Yuasa, Masamitsu Sanui, Hideaki Imanaka, Shinichiro Ohshimo, Ryutaro Seo, Masaaki Sakuraya, Hideto Yasuda, Kazuya Ichikado, Eishu Nango, Ayumu Nozaki, Ryo Kozu, Takeshi Unoki, Yoshinori Takahashi, Akimichi Serita, Eriko Takezawa, Toshio Fukuoka, Taku Yabuki, Morio Aihara, Takeo Nakayama

**Affiliations:** 1 Department of Anesthesiology and Intensive Care, Kyoto Prefectural University of Medicine, Kyoto, Japan; 2 Department of Anesthesiology and Critical Care Medicine, Jichi Medical University Saitama Medical Center, Saitama, Japan; 3 Department of anesthesiology, Kobe University Hospital, Kobe, Japan; 4 Department of Emergency and Critical Care Medicine, Hiroshima University, Hiroshima, Japan; 5 Division of Critical Care Medicine, Okinawa Chubu Hospital, Okinawa, Japan; 6 Department of Emergency Medicine, Kobe City Medical Center General Hospital, Kobe, Japan; 7 Pulmonary & Critical Care Medicine, LDS Hospital, Salt Lake City, USA; 8 Department of Anesthesiology, Nara Medical University, Nara, Japan; 9 Department of Emergency and Critical Care Medicine, Tokyo Bay Medical Center, Tokyo, Japan; 10 Department of Intensive Care Medicine, Kameda Medical Center, Chiba, Japan; 11 Department of General Medicine, Tokyo kita Social Insurance Hospital, Tokyo, Japan

**Keywords:** ARDS, Acute lung injury, systematic review, clinical practice guideline

## Abstract

**Background:**

The Japanese Society of Respiratory Care Medicine and the Japanese Society of Intensive Care Medicine provide here a clinical practice guideline for the management of adult patients with ARDS in the ICU.

**Method:**

The guideline was developed applying the GRADE system for performing robust systematic reviews with plausible recommendations. The guideline consists of 13 clinical questions mainly regarding ventilator settings and drug therapies (the last question includes 11 medications that are not approved for clinical use in Japan).

**Results:**

The recommendations for adult patients with ARDS include: we suggest against early tracheostomy (GRADE 2C), we suggest using NPPV for early respiratory management (GRADE 2C), we recommend the use of low tidal volumes at 6-8 mL/kg (GRADE 1B), we suggest setting the plateau pressure at 30cmH_2_0 or less (GRADE2B), we suggest using PEEP within the range of plateau pressures less than or equal to 30cmH_2_O, without compromising hemodynamics (Grade 2B), and using higher PEEP levels in patients with moderate to severe ARDS (Grade 2B), we suggest using protocolized methods for liberation from mechanical ventilation (Grade 2D), we suggest prone positioning especially in patients with moderate to severe respiratory dysfunction (GRADE 2C), we suggest against the use of high frequency oscillation (GRADE 2C), we suggest the use of neuromuscular blocking agents in patients requiring mechanical ventilation under certain circumstances (GRADE 2B), we suggest fluid restriction in the management of ARDS (GRADE 2A), we do not suggest the use of neutrophil elastase inhibitors (GRADE 2D), we suggest the administration of steroids, equivalent to methylprednisolone 1-2mg/kg/ day (GRADE 2A), and we do not recommend other medications for the treatment of adult patients with ARDS (GRADE1B; inhaled/intravenous β2 stimulants, prostaglandin E_1_, activated protein C, ketoconazole, and lisofylline, GRADE 1C; inhaled nitric oxide, GRADE 1D; surfactant, GRADE 2B; granulocyte macrophage colony-stimulating factor, N-acetylcysteine, GRADE 2C; Statin.)

**Conclusions:**

This article was translated from the Japanese version originally published as the ARDS clinical practice guidelines 2016 by the committee of ARDS clinical practice guideline (Tokyo, 2016, 293p, available from http://www.jsicm.org/ARDSGL/ARDSGL2016.pdf). The original article, written for Japanese healthcare providers, provides points of view that are different from those in other countries.

**Electronic supplementary material:**

The online version of this article (doi:10.1186/s40560-017-0222-3) contains supplementary material, which is available to authorized users.

## Background

Acute respiratory distress syndrome (ARDS) is one of the major manifestations of multiple organ failure syndrome, and is a leading cause of death in the Intensive Care Unit (ICU) [1]. To improve outcomes of patients with this life-threatening condition, tremendous efforts have been made by experts in this area. As a result, a standard ventilator practice using low tidal volume ventilation while limiting plateau pressures has been established [2]. However, due to a shortage of dedicated critical care physicians in Japan [3], substantial numbers of patients suffering from ARDS are cared for by non-specialist physicians and non-physician members of the health care team. In this suboptimal environment, the standardized approach to ventilator management may be underused. Although guidelines for the care of patients with ARDS in other countries are available [4, 5], foreign guidelines cannot directly apply to the clinical practice in our country, since factors affecting clinical practice including health care policy, available medications, and patient and physician preferences vary widely. Moreover, clinical evidence has been updated on a yearly basis since 2004, when the last practical version of ARDS clinical guidelines was published in Japan [6].

To fill these gaps, we aimed to update the clinical practice guidelines (CPG) for ARDS in September 2014, and a guideline development committee was convened. For the development of an evidence-based, unbiased, practical guideline, we adopted the GRADE (Grading of Recommendations Assessment, Development and Evaluation) system, the current world-standard guideline development tool. In July 2016, the Japanese version of the ARDS CPG 2016 was published. This English version of the ARDS CPG 2016 is a translated, abbreviated form of the Japanese version.

Our final goal for the development of this guideline is to improve the outcomes of patients with ARDS in the future. The recommendations in this CPG are encouraged to be used as guidance for health care providers caring for patients with ARDS. However, it should be noted that these recommendations cannot replace clinical decision-making by health care providers, nor do the recommendations eliminate the need to consider patient and provider preferences and special backgrounds.

## Methods

The clinical practice guidelines development (CPGD) committee facilitated the recommendation development process by performing systematic reviews (SRs) on 13 clinical questions (CQs). For performing SRs and determining recommendations, the CPGD committee proposed to adopt the GRADE system, which uses a two-step method of 1) conducting a SR of each CQ and 2) developing recommendations based on the results of the SR (GRADE working group, http://www.gradeworkinggroup.org/).

Thirteen CQs were selected mainly from the ventilatory strategies and medication therapies for adult patients with ARDS. For each CQ, PICO, the abbreviated form of patient, intervention, comparison and outcome was initially determined. Outcomes considered to be important were selected and approved by the CPGD committee and panel members (see below). The importance of each outcome was ranked from 9 (most important) to 1 (least important), and accordingly classified as critical (9 to 7), important (6 to 4), and not important (3 to 1). Critical and important outcomes were chosen for the outcomes of SRs.

The majority of the reviewers are critical care physicians, recruited through a public offering including notifications via mailing lists or other methods. The reviewers extracted search terms from PICO, and then expert librarians (YY, YN and TS) searched publications from MEDLINE (PubMed), EMBASE, Cochrane CENTRAL, and Ichushi Web (the largest databases of medical papers written in Japanese, http://login.jamas.or.jp).

As a general rule, we included only randomized controlled trials (RCTs) published between January 1980 and May 2015 with a few exceptions depending on the individual CQs. In several CQs, we also searched Cochrane reviews or previously published SRs and adopted relevant RCTs from those reviews. The search flow of literature for each CQ is summarized in the form of a PRISMA (Preferred Reporting Items of Systematic Reviews and Meta-Analyses) flow diagram and shown in Additional file 1.

We entered data from selected RCTs into Cochrane Review Manager (RevMan5) software ver.5.3 (http://tech.cochrane.org/revman) for each outcome. If the outcome was binary data, we used random-effect meta-analysis (Mantel-Haenszel method) and calculated the risk ratio (RR) and its 95 % confidence interval. If an outcome was continuous data, we also used random-effects meta-analysis (Inverse Variance method) and calculated the mean difference (MD) and its standard deviation (SD). For several RCTs, we directly contacted the authors and obtained the original data (with permission) and used the data for analysis.

With selected RCTs, systematic reviewers assessed the quality of evidence of each outcome. Since we included only RCTs, the quality of the evidence is initially ranked at “high” and evaluated according to five down grade factors (risk of bias, inconsistency, indirectness, imprecision and other considerations) to determine evidence profiles, summarized in Additional file 1. The summary of finding tables and the evidence profiles was created using online software, the GRADEpro Guideline Development Tool (GDT, http://gdt.guidelinedevelopment.org/). The risk of bias tables, the risk of bias graphs, and the forest plots of the meta-analysis of the 13 CQs were created using RevMan5, as shown in Additional file 1.

Twelve panel members were selected for the panel discussion conference to determine the final recommendations. The panelists represented a wide range of stakeholders in this matter including intensive care specialists, pulmonologists, general physicians (hospitalists), nurses, pharmacists, clinical engineering technicians, physical therapists and patient families. Eight panel members were recruited in a public offering via the Internet. Four more members were selected from CPGD committee members or from among the systematic reviewers who did not have pertinent COI to declare, and did not participate in the systematic review process of the relevant CQs. The details are shown in Additional file 2. Overall certainty of the evidence across outcomes for each CQ was determined only by considering outcomes rated as “critical”.

The recommendations were determined by considering the patient's values, cost and resource use, in addition to the certainty of the evidence and the balance between the benefit and harm. We developed tables of evidence-to-decision framework with the aid of the guideline development tool for each CQs based on the summary of finding tables and evidence profiles, which finally led to making the recommendation drafts (Additional file 1).

In the evidence-to-decision framework, each of the following factors was taken into consideration in the development of the recommendations: the certainty of the evidence, the balance of desirable and undesirable consequences of compared management options, the assumptions about the values and preferences associated with the decision, the implications for costs, resource use and health equity, the acceptability of intervention to stakeholders, and the feasibility of implementation. Recommendations and their strength were determined through a comprehensive approach considering all these factors by panel consensus. The certainty of evidence was derived from the overall quality of the evidence across outcomes, evaluated by the SR process. Based on the balance between desirable and undesirable effects, a recommendation can be made if desirable effects surpass undesirable effects, but the recommendation cannot be made if the undesirable effects are greater than the desirable effects. Considering the current situation of health care finance in this country, we examined whether there would be sufficient desirable effects considering costs and use of resources. We also took into consideration the current common practice for patients with ARDS in Japan and recommendations of existing clinical practice guidelines.

A recommendation was constituted and presented as a combination of three factors: “direction of recommendation”, “strength of recommendation” and “certainty of the evidence". “Direction of recommendation” was determined as either “for” or “against” the intervention. “Strength of recommendation” was determined as either “strong (1)” or “weak (2)”. “Certainty of the evidence” was determined as either “high (A)”, “moderate (B)”, “low (C)” or “very low (D)”. Strong and weak recommendations were presented using the phrases “we recommend” or “we suggest”, respectively. Four recommendation categories can exist based on possible combinations of direction and strength of recommendations, but it is prudent not to consider that the four categories are independent and separate, but rather to consider four points of the recommendations as evenly distributed in a single continuous horizontal line.

In principle, the recommendations were determined when all panelists unanimously approved the recommendation drafts. The modified Delphi method was used to form a consensus. Prior to the discussion conference, we sent recommendation drafts and materials prepared by the systematic reviewers to all panelists. The panelists voted for their desired recommendations before the panel discussion was held (RAND 1st time).

S.H., the chairperson of the CTGD committee, facilitated the panel discussion. The panel discussion conference took a total of about 14 hours at Tokyo on September 22nd and 23rd, 2015. After the panelists approval to adopt the GRADE approach was obtained, discussions were then held with the aid of the summary of finding tables, GRADE evidence profiles, evidence-to-decision Frameworks and recommendation drafts. Accordingly, the importance of the outcomes of each CQ was evaluated, and then the balance of desirable and undesirable consequences of comparable management options, the variation of values, cost and resource use were evaluated. Thereafter, the strength of the recommendation was evaluated, and finally the recommendation of each CQ was determined. If no agreement was obtained within a predetermined time, a second vote was called by the chairperson (RAND 2nd time).

Based on recommendations made at the panel discussion, the various materials mentioned above were compiled and a draft of the clinical practice guideline was prepared. The final draft was opened for the public comment from April 18, 2016 for one month. During this period, we received 20 responses. Simultaneously, external validation members (TF, TY, MA, TN) evaluated the draft using AGREE II checklist (The Appraisal of Guidelines for Research and Evaluation, http://www.agreetrust.org/agree-ii/). Following finalization, the Japanese version of the PDF form guideline was open to the public on July 9, 2016 (http://www.jsicm.org/ARDSGL/ARDSGL2016.pdf). Since the original guideline was written in Japanese, we then translated the essential part of the guideline into English with supplemental comments added for making the English manuscript easier to read.

## Result

### CQ1: Should early tracheostomy be performed in adult patients with ARDS?

**Table:** 

**Recommendation: We suggest against early tracheostomy in adult patients with ARDS. (GRADE 2C, Strength of recommendation “weak recommendation” / Quality of evidence: ”low”)**
● **Supplementary conditions:** Early tracheostomy could be beneficial in some patients including those anticipated to have prolonged upper airway obstruction, patients with hemodynamic instability due to sedative-analgesic agents required during intubation, and patients with delayed rehabilitation. However, early tracheostomy cannot be recommended for all patients.

### Background, the priority of this issue

In patients requiring mechanical ventilation, endotracheal intubation is generally used for airway management. Tracheostomy has some advantages including potential reduction in the need for sedative-analgesic agents and avoidance of vocal cord injury by an endotracheal tube [7, 8]. However, tracheostomy is invasive and associated with complications including bleeding or tracheal stenosis [9, 10]. For these reasons, tracheostomy is generally performed after at least 14 days of endotracheal intubation and continued need for mechanical ventilation is anticipated.

However, some may suggest that early tracheostomy could shorten the duration of mechanical ventilation, reduce the incidence of ventilator associated pneumonia (VAP), and improve outcomes, if these advantages are maximized. Therefore, it is important to examine the impact of early tracheostomy on outcomes in patients with ARDS, as this cohort is likely to require long-term mechanical ventilation.

Currently, there is no study that has investigated the appropriate timing for tracheostomy solely in adult patients with ARDS. Thus, for this CQ, we include studies with patients who have respiratory failure and were anticipated to require long-term mechanical ventilation in order to determine whether early/late tracheostomy is beneficial or harmful.

### Description

#### Summary of Evidence

No study was found for adult patients with ARDS. We conducted a systematic review for randomized controlled trials (RCTs) conducted in patients anticipated to require long-term mechanical ventilation. Nineteen RCTs were found. Since the time from the initiation of mechanical ventilation to tracheostomy is generally 14 days in Japan, studies were divided into early and late tracheostomy groups using thresholds of 4, 7, and 10 days from the commencement of mechanical ventilation. Early tracheostomy within 4, 7, and 10 days from the initial mechanical ventilation did not reduce the mortality or the incidence of VAP compared with later tracheostomy. However, early tracheostomy is unlikely to worsen outcomes since the relative risk was less than 1.

### Panel meeting

#### What is the overall quality of evidence across outcomes?

Overall, the selected studies had a low risk of bias. Although the risk of bias for mortality was ‘not serious’, the risk of bias for VAP was downgraded by one level and classified as ‘serious’. Regarding inconsistency of results, heterogeneity for mortality (at 10 days) was low and ‘not serious’ since I^2^=19%. However, the heterogeneity for the remaining outcomes was high since I^2^≥50%, thus, inconsistency of results was downgraded by one level and classified as ‘serious’. Indirectness was considered as ‘serious’ in any of the outcomes because of the unmatched study subjects, as subjects included in selected RCTs were those anticipated to require long-term mechanical ventilation. The level of imprecision was downgraded by one level for all outcomes since the confidence intervals overlap the clinical decision thresholds. For publication bias for the risk of VAP was downgraded by one level and classified as ‘serious’ because the result of the funnel plot test was asymmetrical. Based on the above discussion, the overall quality of evidence was evaluated as ‘low’.

#### What is the balance between benefits and harms?

Since patients with ARDS require high inspired oxygen concentrations, high airway pressures, or high PEEP levels, they may have a higher rate of undergoing tracheostomy, compared with patients included in the selected RCTs. As no accurate method to predict the need for prolonged mechanical ventilation exists, unnecessary tracheostomy could not completely be avoided if early tracheostomy had been applied in all cases. It would be considered that 40% of tracheostomy in the early tracheostomy arm could be avoidable in the late tracheostomy arm [11]. Therefore, it cannot be determined that the benefits of performing early tracheostomy outweigh the harms of performing it in patients with ARDS.

#### What are the values and preferences of the patients?

The values and preferences of patients regarding this issue are considered consistent. Patients will want to have a tracheostomy if it has a clear benefit. If not, patients will not want it to be performed. This was unanimously agreed upon by all panel members. Therefore, the majority of the patients are likely to be against the early routine tracheostomy.

#### What about the balance between the net benefit and the cost or resources?

If early tracheostomy is performed on all patients, the rate of unnecessary tracheostomy would be increased. Since the mortality rate and the incidence rate of VAP are not decreased by using early tracheostomy, the total medical costs will be higher with early tracheostomy. It has been reported that approximately 40% of patients with early tracheostomy might not need tracheostomy if late tracheostomy had been planned [12]. Except for patients who clearly benefit from tracheostomy, the cost of early tracheostomy is considered to be high.

#### Grading of recommendation

In the discussion at the panel meeting, we did not confirm that early tracheostomy decreases mortality or rate of VAP, while it is unlikely to worsen clinical outcomes. Considering the increased cost due to an increased rate of tracheostomy, we conclude that tracheostomy should not be routinely performed at an early stage in patients with ARDS. However, there are some patients who may benefit from early tracheostomy rather than later. Therefore, we state the recommendation as “it is proposed not to perform early tracheostomy”.

### Description in other relevant clinical practice guidelines

There is no guideline describing tracheostomy in patients with ARDS.

### Monitoring and assessment of treatment

Standard monitoring for respiratory and circulatory functions is sufficient.

### Possible future studies

A study to evaluate the optimal timing of tracheostomy in patients with ARDS is needed. Also, a method to accurately identify patients requiring long-term mechanical ventilation needs to be developed. With the availability of such a method, the number of unnecessary tracheostomies could be reduced and the evaluation of early tracheostomy could be changed. There are two types of tracheostomy typically used: surgical tracheostomy and percutaneous tracheostomy. A study to investigate which type of tracheostomy is safer in patients with ARDS is needed.

### Literature search method and literature selection

We searched PubMed (through February 2, 2015), Embase (through February 2, 2015), Cochrane CENTRAL (through February 3, 2015) and Igaku Chuo Zasshi (Japana Centra Revuo Medicina, Ichushi) (through January 26, 2015) with keywords “tracheostomy” and “mechanical ventilation”. There were 2249 studies identified. After screening these studies, we included 19 randomized controlled trials in the analysis of this clinical question.

### RCTs included in this CQ

Barquist 2006 [13], Blot 2008 [14], Bosel 2013 [15], Bouderk 2004 [16], Bylappa 2011 [17], Diaz-Prieto 2014 [18], Dunham 1984 [19], Dunham 2014 [20], Fayed 2012 [21], Koch 2012 [22], Mohamed 2014 [23], Rodriguez 1990 [24], Rumbak 2004 [25], Saffle 2002 [26], Sugerman 1997 [27], Terragni 2010 [28], Trouillet 2011 [29], Young 2013 [30], Zheng 2012 [31]

### CQ2: Should non-invasive positive pressure ventilation (NPPV) be used for early respiratory management of adult patients with ARDS?

**Table:** 

**Recommendation: We suggest using NPPV for early respiratory management in adults with ARDS. (GRADE 2C,Strength of recommendation “weak recommendation” / Quality of evidence: ”low”)**
●**Supplementary conditions: Monitor the patient for clinical improvement within 1-2 hours of initiating NPPV**. Then confirm whether the patient’s respiratory status meets a predefined goal set prior to initiating NPPV within 4-6 hours. Many RCTs exclude unconscious patients and hemodynamically unstable patients. Applying this recommendation requires caution in these populations [32].

### Background, the priority of this issue

Although non-invasive positive pressure ventilation (NPPV) is used for treating hypoxia worldwide, its efficacy for patients with ARDS has not been evaluated thoroughly. It is shown that the use of NPPV may lead to a lower intubation rate and reduced mortality in patients with ARDS. Therefore, its priority for clinical use is high.

### Description

#### Summary of Evidence

Since it was anticipated that the number of studies concerning the efficacy of early NPPV initiation in patients with ARDS is small, we searched for comparative RCTs dealing with the efficacy of NPPV in patients with hypoxemia. The choice to use oxygen therapy or conventional mechanical ventilation depends primarily on the severity of hypoxemia, hence, in our systematic review, we compared the efficacy of NPPV with oxygen therapy, and also, the efficacy of NPPV compared to conventional mechanical ventilation. We excluded studies involving patients with COPD or congestive heart failure, because it is expected that these studies would strongly support the use of NPPV. As a result, a total of 21 studies were included. Among these studies, 17 compared the efficacy of NPPV with oxygen therapy and 21 compared the efficacy of NPPV with conventional mechanical ventilation.

In the 17 RCTs comparing NPPV to oxygen therapy, NPPV significantly reduced the mortality (RR 0.71, 95%CI 0.54~0.92). In 16 RCTs comparing NPPV to oxygen therapy, NPPV significantly reduced the intubation rate (RR 0.58, 95%CI 0.46~0.74). In 4 RCTs comparing NPPV to conventional mechanical ventilation, NPPV significantly reduced the mortality (RR 0.65, 95%CI 0.46~0.93).

### Panel meeting

#### What is the overall quality of evidence across outcomes?

In 17 RCTs comparing NPPV to oxygen therapy, the risk of bias for mortality is ‘not serious’. The risk of bias for intubation was downgraded by one level and classified as ‘serious’ because the open-label fashion seemed to affect the decision to intubate. Inconsistency of results for mortality was downgraded as ‘serious’ since a wide variance of point estimates across studies was found and heterogeneity for mortality was moderate (I^2^=44%). However, in terms of intubation, the heterogeneity is low (I^2^=25%) and the variance of point estimates across studies is not significant. The inconsistency of results was ‘not serious’. Indirectness was considered as ‘serious’ in both outcomes, mortality and intubation rate, because of unmatched study subjects, as subjects included in selected RCTs had hypoxemia, not ARDS. The level of imprecision was ‘not serious’ for mortality and intubation since criteria of the optimal information size (OIS) were met. Based on the above discussion, the overall quality of evidence is evaluated as ‘low’.

In 4 RCTs comparing NPPV to conventional mechanical ventilation, the risk of bias for mortality is ‘not serious’. Inconsistency of results for mortality is ‘not serious’ since heterogeneity is low (I^2^=0%) and the variance of point estimates across studies is not significant. Indirectness is considered as ‘not serious’ although all subjects included in selected RCTs didn’t meet criteria of ARDS. The level of imprecision for mortality is ‘serious’ since criteria for the OIS were not met. Based on the above discussion, the overall quality of evidence is evaluated as ‘low’.

#### What is the balance between benefits and harms?

Use of an NPPV mask may complicate skin ulcers. According to the current study, comparing estimated benefits and harms related to NPPV use, the harm seems insignificant, although, in comparison to oxygen therapy, NPPV may impair a patient’s hemodynamic stability by decreasing the right atrial venous return or cause lung injury due to positive airway pressure.

#### What are the values and preferences of the patients?

Compared to conventional mechanical ventilation, the distress of patients undergoing NPPV is considered to be less. However, NPPV masks may make a patient uncomfortable and they may prefer oxygen therapy to NPPV.

#### What about the balance between the net benefit and the cost or resources?

Despite the fact that NPPV has become a therapy that can be used in many hospitals in Japan, the cost of NPPV may be higher than that of oxygen therapy or intubation due to the cost of a ventilator with an NPPV mode or specialized for NPPV, the interface for NPPV, amount of oxygen required, cost of training medical staff, cost of hiring support staff, etc. In addition, disposable masks for NPPV are quite costly. There is the possibility that the overall cost of NPPV may be lower by avoiding intubation. Therefore, it cannot be determined whether the net benefit of NPPV outweighs the cost or resources in patients with ARDS.

#### Grading of recommendation

In the discussion at the panel meeting, all panel members supported the use of NPPV as first-line therapy for patients with hypoxemia, since NPPV decreases mortality and intubation rates compared to oxygen therapy, and decreases the mortality rate compared to immediate intubation, despite the expected increase in cost. However, indirectness is ‘serious’ because of unmatched subjects. As a result, all panel members supported the recommendation “We suggest using NPPV for the early respiratory management of adults with ARDS.”

Most panel members were concerned about the harm of delayed intubation. When a patient’s general condition is critical or when there is no improvement in oxygenation after initiating NPPV, immediate intubation is essential. During the panel meeting, we discussed the validity of wording and eventually adopted usage of the word “NPPV” instead of non-invasive ventilation (NIV) and “early respiratory management of adults with ARDS” instead of “respiratory management in adults with mild ARDS.”

### Description in other relevant clinical practice guidelines

The Japanese Respiratory Society published the ‘Guidelines for Noninvasive Positive Pressure Ventilation (NPPV), 2nd Edition’ in 2015 [33]. In the guidelines, “(1) NPPV should be used with caution in patients with ARDS, (2) the use of NPPV is recommended in patients with mild ARDS with fewer other organs failing,” and there is no conflict between that statement and our recommendation [33].

### Monitoring and assessment of treatment

While undergoing NPPV, the patient’s respiratory status, circulatory status, consciousness and arterial blood gas should be evaluated repeatedly. First, monitor the patient for clinical improvement within 1-2 hours of initiating NPPV. Then, confirm whether the patient’s respiratory status meets a predefined goal set prior to initiating NPPV within 4-6 hours. When the patient is not improving clinically within 1-2 hours and not achieving the goal within 4-6 hours, the patient should be intubated. One study suggested that delayed intubation is related to higher mortality [34].

### Possible future studies

More studies to evaluate the efficacy of NPPV for patients with ARDS are needed. Also, the efficacy of other non-invasive respiratory support techniques such as high-flow nasal therapy should be compared to oxygen therapy, conventional mechanical ventilation and NPPV.

### Literature search method and literature selection

We searched PubMed (through May 6, 2015), EMBASE (through June 2, 2015), Cochrane CENTRAL (through June 1, 2015) and Igaku Chuo Zasshi (Japana Centra Revuo Medicina) (through June 2, 2015) with the keywords “NPPV” and “hypoxemia.” There were 3985 studies identified. After screening these studies, we identified 21 randomized controlled trials for the analysis of this clinical question.

### RCTs included in this CQ

Antonelli 2000 [35], Antonelli 1998 [36], Brambilla 2014 [37], Confalonieri 1999 [38], Cosentini 2010 [39], Delclaux 2000 [40], Ferrer 2003 [41], Gunduz 2005 [42], Gupta 2010 [43], Hernandez 2010 [44], Hilbert 2001 [45], Honrubia 2005 [46], Kramer 1995 [47], Matic 2007 [48], Martin 2000 [49], Nava 2013 [50], Squadrone 2010 [51], Wermke 2012 [52], Wood 1998 [53], Wysocki 1995 [54], Zhan 2012 [55].

### CQ3:Should low tidal volume be used in adult patients with ARDS?

**Table:** 

**Recommendation:We recommend the use of low tidal volume at 6-8 mL/kg (predicted body weight: PBW) in adult patients with ARDS. (GRADE 1B, STRONG RECOMMENDATION / MODERATE-QUALITY EVIDENCE)**
● **Supplementary conditions:** Tidal volume is calculated based on PBW (Male:50+0.91 x [Height(cm) – 152.4], Female: 45.5+0.9x[Height(cm) – 152.4]) rather than actual body weight. When a lung protective ventilation strategy is applied, a tidal volume equal to or less than 10mL/kg PBW is considered beneficial. However, the optimal tidal volume still remains to be determined. Of the RCTs analyzed in this review, the actual tidal volume delivered in the lung protective strategy group was 6.2-7.6 mL/kg. Therefore, we recommend a tidal volume of 6-8 mL/kg PBW. In case of an excessive spontaneous breathing effort, the actual tidal volume may sometimes exceed the targeted tidal volume. To prevent this, respiratory parameters such as driving pressure or trans-pulmonary pressure may need to be used as monitoring tools to determine an appropriate tidal volume.

### Background, the priority of this issue

The strategy for mechanical ventilation is very important in patients with acute respiratory distress syndrome (ARDS), in addition to the treatment of the primary disease. In particular, mechanical ventilation settings have the highest priority for patients with ARDS. Studies have been conducted to determine the optimal ventilation strategy to limit tidal volume and airway pressure, thereby further reducing lung injury by providing lung-protective ventilation in patients with ARDS.

### Description

#### Summary of Evidence

A total of six randomized controlled trials (RCTs) qualified for inclusion for the systematic review, where a lung protective ventilation strategy with low tidal volume was studied in adult patients with ARDS. These six RCTs were also analyzed by Petrucci et al. in 2013 and no new RCT has been published since then [56]. Although the duration of follow-up was different, all six RCTs (n=1,305) demonstrated a non-significant decrease in mortality in the low tidal volume group compared with the conventional tidal volume group (RR0.84, 95%CI 0.67-1.07). The occurrence of barotrauma (pneumothorax secondary to elevated airway pressure) was analyzed in all six RCTs, and there was no significant difference between the two groups (RR0.82, 95%CI 0.48-1.41). Ventilator Free Days (VFD) was analyzed in only three RCTs and VFD was significantly longer (median, 2.52 more days) in the low tidal volume group than in the conventional tidal volume group (95%CI 0.53 to 4.51).

### Panel meeting

#### What about the quality of evidence regarding outcome in general?

The certainty of evidence regarding mortality decreased by two levels and was rated “low” for three reasons. First, there was a difference in the length of follow-up regarding mortality (28-day, 60-day, and hospital) among the RCTs. Second, there was heterogeneity of the cohorts among the RCTs (I2=50%). Third, the confidence interval was wide. For barotrauma, the certainty of evidence was rated “very low”. For VFD, the certainty of evidence was rated “moderate”. Overall, the quality of evidence was rated “moderate” since a lung protective ventilation strategy had a non-significant, but positive impact on all outcomes.

#### What about the balance between benefits and harms?

Selection of a low tidal volume (lung protective ventilation strategy) will increase VFD. Although this strategy may induce hypercarbia or respiratory acidosis, the benefits will eventually outweigh the risks.

#### What about the values and preferences of the patients?

Use of low tidal volume is not associated with an increase in sedative or analgesic doses compared with the use of conventional tidal volume strategies [57]. Therefore, the pain and discomfort felt by patients undergoing mechanical ventilation will not increase with the use of a low tidal volume strategy. Variation in patient preferences is considered minimal because the panel members unanimously agreed on the recommendation for the use of low tidal volume in adult patients with ARDS.

#### What about the balance between the net benefit and the cost or resources?

A change in tidal volume settings with mechanical ventilation can be applied to all patients undergoing mechanical ventilation and requires no new resources or additional costs. The use of low tidal volume increases VFD significantly with a tendency to decrease mortality and barotrauma. Although this strategy may induce hypercarbia or respiratory acidosis as a potential complication, benefits will outweigh the potential risks.

#### Grading of recommendation

The recommendation “We recommend the use of low tidal volume at 6-8mL/kg (predicted body weight: PBW) in adult patients with ARDS” was decided unanimously during the panel meeting.

### Descriptions in other relevant clinical practice guidelines

Use of low tidal volume is recommended in international guidelines for the management of sepsis, the Surviving Sepsis Campaign 2012 [58] which suggests a target tidal volume of 6mL/kg, based on predicted body weight in patients with sepsis-induced ARDS (grade 1A). The Japanese guidelines for the management of sepsis [59] also recommend the use of low tidal volumes. These recommendations for the use of tidal volumes of 6ml/kg (PBW) are based on a large RCT, reported by the ARDS network 2000,9) alone. Although we recommend the use of a tidal volume of 6-8ml/kg (PBW) based on the results of six RCTs, including the ARDS network 2000 report, our recommendation is not different from these guidelines regarding the use of low tidal volume ventilation.

A recent meta-analysis by the Cochrane Institution [56] analyzed six identical RCTs, and reported that the use of low tidal volumes decreased mortality at 28 days and at the end of follow-up periods compared with the use of conventional tidal volume ventilation. While they used a fixed effect model to achieve the decrease in mortality in the forest plot, we applied a random effect model for the analysis due to non-negligible heterogeneity among the six RCTs. An association of the use of low tidal volumes and a decrease in short-term mortality was not shown in our analysis. The discrepancy in the results from the Cochrane Institution and our results should be appreciated, but the mortality at the end of follow up would not differ significantly if they used the same random effect model we used. Although barotrauma and an increase of VFD were analyzed in this CQ, they were not analyzed in the Cochrane meta-analysis.

### Monitoring and evaluation of the treatment

Monitoring of respiratory parameters (arterial oxygen or carbon dioxide levels, airway pressure etc.) is required to assess adequate arterial oxygenation and ventilation.

### Possible future studies

Since a lung protective strategy has been accepted as the global standard ventilation technique in patients with ARDS, a new RCT to compare the efficacy of a low tidal volume strategy with a conventional tidal volume strategy has not been conducted since 2006. However, the ideal tidal volume still remains to be determined (e.g. 6mL/kg vs. 8mL/kg PBW), and thus, further studies are required. Another future research interest may focus on driving pressure or transpulmonary pressure as potential ideal markers for lung protective low-tidal ventilation.

### Literature search method and literature selection

This clinical question was evaluated by Petrucci, reported by the Cochrane Institution (through Sep 2012) [56]. Therefore, we conducted a literature search from Sep 2011 through May 2015. Five hundred and eighty-one studies were identified in PubMed, EMBASE, Cochrane CENTRAL and Igaku Chuo Zasshi (Japana Centra Revuo Medicina) using the keywords “acute respiratory failure” and “low tidal volume”. We could not find a new RCT after screening these studies, and included six RCTs evaluated in the meta-analysis performed by the Cochrane Institution.

### RCTs reviewed in this CQ

Amato 1998 [60], Brochard 1998 [61], Stewart 1998 [62], Brower 1999 [63], ARDS Network 2000 [2], Villar 2006 [64]

### CQ4:What is the optimal plateau pressure for mechanical ventilation in adult patients with ARDS?

**Table:** 

**Recommendation:We suggest setting the plateau pressure at 30cmH20 or less in adult patients with ARDS undergoing mechanical ventilation. (GRADE2B, weak recommendation / evidence level moderate)**
● **Supplementary conditions:** The optimal plateau pressure remains to be determined in the future.

### Background, the priority of this issue

When providing mechanical ventilation to adult patients with ARDS, decreased lung compliance is one of the main etiologic factors for the development of ventilator associated lung injury. Ventilator associated lung injury leads not only to delayed weaning, but also to increased mortality [65]. Among several causes of ventilator associated lung injury, increased tidal volume and airway pressure are important factors, both of which can be controlled by limiting the plateau pressure [66]. While limiting the plateau pressure can be beneficial, it can also lead to adverse events such as hypercapnia [67]. Although there is no practical way to determine the optimal plateau pressure, a method should be developed to minimize ventilator associated lung injury. Therefore, the priority of this CQ is high.

### Description

#### Summary of Evidence

Four RCTs (total number of patients=1132) were selected for this systematic review. By setting the plateau pressure below 30cmH2O, the number of ventilator free days (VFD) was significantly increased (mean 2.5days, 95% CI 0.51-4.49). The mortality (RR 0.84, 95%CI 0.62-1.15)and lung injury caused by high airway pressure (RR 0.92, 95%CI 0.65-1.31) were decreased, but there is no statistically significant difference.

### Panel meeting

#### What is the overall quality of evidence across outcomes?

In these RCTs that compare values of ventilator settings (such as comparing two plateau pressures), it is difficult to blind the entire study to patients and medical staff. Thus, we determined the risk of bias for all outcomes as ‘serious’ and downgraded them as a whole. There is no inconsistency or indirectness. Although the total number of events (death, 381/1132 patients), is sufficient, there is no statistically significant difference. The 95% confidence interval is considered to be wide. Thus, we determined it is ‘serious’ in terms of mortality. Overall, we conclude that the quality of evidence in these 4 RCTs is “moderate”.

#### What is the balance between benefits and harms?

Hypoxemia, hypercapnia and increased work of breathing work caused by inappropriate ventilator settings are possible harms in this CQ. However, these harms are relatively permissive and should not cause any serious sequelae.

#### What are the values and preferences of the patients?

Ventilator associated respiratory injury caused by an increase in plateau pressure is obviously a serious adverse event. Thus, limiting the plateau pressure would be accepted by patients without any hesitation. It is assumed that most patients will select limiting plateau pressure on the ventilator.

#### What about the balance between the net benefit and the cost or resources?

There is no change in resources required by changing the ventilator settings. As there is no additional increase of required resources, the benefit prevails.

#### Grading of recommendation

Among three critical outcomes, mortality and barotrauma were not significantly decreased, but the number of VFD increased significantly. At the panel conference, after reviewing these results, it was unanimously concluded that ventilator settings which limit plateau pressure to less than 30cmH2O should be achieved, with a weak recommendation.

### Description in other relevant clinical practice guidelines

In the Scandinavian clinical practice guideline 2014 [5], which cited a Cochrane review [56], they strongly recommended that airway pressure and tidal volume should be limited. However, we should be aware that the guideline stated no upper limit for the plateau pressure. While, in the Surviving Sepsis Campaign Guideline 2012[58], it is recommended that plateau pressure should be measured with an initial plateau pressure less than 30cmH2O, at the time of passive pulmonary expansion (1B).

### Monitoring and assessment of treatment

As the intervention is a change in ventilator settings, we should monitor oxygenation and other appropriate parameters of mechanical ventilation.

### Possible future studies

As the optimal plateau pressure is undefined, studies comparing various plateau pressures are needed. As trans-pulmonary pressure is now drawing a lot of attention, it is necessary to consider a comparison of plateau pressures when patients are spontaneously breathing.

### Literature search method and literature selection

We searched PubMed (through May 2, 2015), Embase (through May 2, 2015), Cochrane CENTRAL (through May 2, 2015) and Igaku Chuo Zasshi (Japana Centra Revuo Medicina, Ichushi) (through May 2, 2015). The search strategies combined terms for Acute Respiratory Distress Syndrome, terms for lung protective ventilation, and a search filter for RCTs. A total of 3324 RCTs were identified using this search strategy. As a result of the screening, 4 RCTs were included in the final analysis.

### RCTs included in this CQ

Brochard 1998[61], Brower 1999[63], ARDS Network 2000[2], Villar 2006[64]

### CQ5:What is the optimal positive end-expiratory pressure (PEEP) in adult patients with ARDS?

**Table:** 

**Recommendation:We suggest using PEEP within the range of plateau pressures less than or equal to 30cmH2O, without compromising hemodynamics (Grade 2B, strength of recommendation “weak recommendation” / Quality of evidence ”moderate”). We also suggest using higher PEEP levels in patients with moderate to severe ARDS (Grade 2B,Strength of recommendation “weak recommendation” / Quality of evidence “moderate”).**
●**Supplementary statements:** Increasing PEEP levels may result in high plateau pressures, hypotension or a decrease in tidal volume. Close monitoring of hemodynamics and other parameters is necessary when high PEEP levels are used.

### Background and the priority of this issue

It is well known that PEEP prevents atelectasis and improves oxygenation in patients undergoing mechanical ventilation. It has been suggested that PEEP not only improves oxygenation but also prevents ventilator-induced lung injury by recruiting alveoli collapsed by inflammation and exudative fluid in patients with ARDS [68, 69].

The priority of this issue is thought to be high although the optimal PEEP level is undefined.

### Description

#### Summary of Evidence

We conducted a systematic review and included seven randomized clinical trials, which show that there are no differences in hospital mortality, incidence of barotrauma or ventilator-free days (VFD) comparing patient groups receiving higher PEEP and lower PEEP levels (hospital mortality RR 0.93; 95%CI 0.83-1.04, barotrauma RR 0.97; 95%CI 0.66-1.42, VFD 1.89 days more; 95%CI -3.58 to 7.36).

Only three trials (Brower2004、Meade2008、Mercat2008) were included in the analysis for hospital mortality because trials that had interventions with potential effects on the outcome other than PEEP in the experimental groups were excluded[70–72].

The meta-analysis included all studies and showed no significant difference in hospital mortality comparing higher PEEP and lower PEEP groups (RR 0.87;95%CI 0.74-1.02).

A subgroup analysis comparing the mortality rate of higher PEEP and lower PEEP groups in patients with moderate to severe ARDS (P/F ≤200) showed a significantly lower hospital mortality in the higher PEEP group in both analyses in all trials and the analysis excluding trials that had interventions other than PEEP in experimental groups (RR 0.82, 95%CI 0.73 to 0.92、RR 0.85, 95%CI 0.75 to 0.96 respectively).

### Panel meeting

#### What is the overall quality of evidence regarding outcomes in included studies?

Among the seven studies included in this systematic review, five (Amato 1998, Brower 2004, Mercat 2008, Talmor 2008 and Villar 2006) were terminated early [60, 64, 70, 72, 73] and three (Amato 1998, Talmor 2008 and Villar 2006) had inadequate sample sizes. Therefore, the overall quality of evidence for each outcome is considered “moderate” after downgrading by one level.

#### What about the balance between benefits and harms?

No obvious benefits or harms were identified. There are no direct effects on cost by changing ventilator settings. Hemodynamic changes due to high levels of PEEP needs to be monitored.

#### What about patients’ values and preferences?

The variability in patients’ values and preferences about the main outcomes is thought to be small.

#### What about the balance between the net benefit and the costs or resources?

As there is no difference in the number of VFD comparing groups receiving high and low PEEP, no differences in costs or resources are expected. Therefore, it is hard to refer to the balance between benefits and the costs or resources based on the level of PEEP.

#### Grading of the recommendations

In the panel discussion, the focus was on the higher level of PEEP in patients with moderate to severe ARDS. It was proposed that another panel discussion be held after adding a subgroup analysis including only patients with moderate to severe ARDS. Since the subgroup analysis did not show a significant difference in mortality comparing higher and lower PEEP levels, the recommendation not to use a higher PEEP level routinely was suggested. However, because the PEEP levels used in the lower PEEP groups in Brower 2004, Meade 2008, and Mercat 2008 were not “low” in general, some panelists raised the concern that the recommendation may lead to ventilator settings with unnecessarily low PEEP and there was no consensus to accept the recommendation during the second panel discussion. The final version was approved through an email discussion among panelists. After approval of the final version, it was decided to use a random effect model rather than a fixed effect model to analyze all clinical questions. CQ5 was re-analyzed using the random effect model and showed that the mortality in the higher PEEP group is significantly lower compared to the lower PEEP group in patients with moderate to severe ARDS (P/F≤200) in both analyses with all studies and after excluding studies with a significant difference in interventions other than PEEP level between two groups. Another email discussion was held and all panelists agreed that the final version did not need to be changed, as the recommendations are still consistent with the results.

### Descriptions in other relevant clinical practice guidelines

The Surviving Sepsis Campaign and the Scandinavian clinical practice guideline on mechanical ventilation in ARDS 2014 recommend or suggest using PEEP rather then not using PEEP (at least higher or equal to 5cmH2O in general) [5, 58].

### Monitoring and evaluation during management

Monitoring of indices related to mechanical ventilation such as oxygenation, ventilation, pressures and volumes is important. High PEEP requires careful monitoring of hemodynamic status.

### Implications for future studies

The panel meeting concluded that it is appropriate to use the FiO2/PEEP ladder used in ARDSnetwork 2000 and Brower 2004 to determine the PEEP level required at present [2], as there are no other methods shown to be more practical or better to determine the optimal PEEP level. It is necessary to identify which subgroups benefit from lower PEEP or higher PEEP. Further studies are also required to compare methods to determine the optimal PEEP level for individual patients, rather than compare lower and higher PEEP levels.

### Methods for literature search and inclusion

We used the seven studies included in the previous Cochrane systematic review [74] for the studies published until March 31, 2012.

We searched PubMed, EMBASE, Cochrane CENTRAL and Igaku Chuo Zasshi (Japana Centra Revuo Medicina) from April 1, 2012 to June 30, 2015 with the keywords “ARDS” and “PEEP”. A total of 871 studies were identified. We included 6 randomized controlled trials in this guideline after screening.

### Randomized control trials included in this clinical question

Amato 1998 [60], Brower 2004 [70], Huh 2009 [75], Mercat 2008 [72], Villar 2006 [64], Meade 2008 [71]

### CQ6:Should liberation from mechanical ventilation be protocolized in patients with ARDS?

**Table:** 

**Recommendation: We suggest using protocolized methods for liberation from mechanical ventilation in patients with ARDS (Grade 2D,Strength of recommendation “weak recommendation” / Quality of evidence “Very low”).**
●**Supplementary statements:** When developing protocols for liberation, the level of knowledge and skills of the personnel who apply the protocol in each facility must be taken into account. Education and training regarding mechanical ventilation are required, especially for non-physician staff members. A previous meta-analysis [76] showed a reduction in the duration of mechanical ventilation for patients in medical, surgical and medical/surgical ICUs but not in a neurological ICU.

### Background and the priority of this issue

Because the process of liberation (formerly referred to as “weaning”) from mechanical ventilation is not standardized in Japan, it is likely that a large number of patients remain on mechanical ventilation longer than necessary. It is suggested that the use of protocols for liberation from mechanical ventilation prevents unnecessarily prolonged mechanical ventilation with a significant reduction in the duration of mechanical ventilation [77, 78]. Many patients with ARDS require a long period of mechanical ventilation and they would greatly benefit if the use of liberation protocols is effective in shortening the period of mechanical ventilation. As there are no previous studies to evaluate the usefulness of liberation protocols specifically in patients with ARDS, we included studies that evaluated the effect of protocolized liberation in critically ill patients undergoing mechanical ventilation to investigate whether protocolized liberation should be used in patients with ARDS.

### Description

#### Summary of Evidence

Since this systematic review revealed that there are no previous studies which evaluated only patients with ARDS, we included 12 RCTs that included critical ill patients undergoing mechanical ventilation in this meta-analysis. The meta-analysis shows a significantly shorter duration of mechanical ventilation in patients liberated according to a protocol compared to patients liberated without a protocol (average difference -21.51 hours 95%Cl -38.45 - -4.56 hours). It also shows that protocolized liberation from mechanical ventilation significantly reduced the number of tracheostomies needed (RR 0.72, 95%CI 0.52-0.99). There were no significant differences in the incidence of adverse events between the two groups (re-intubation: RR 0.79, 95%CI 0.50-1.26, hospital mortality: RR 1.04, 95%CI 0.88-1.23).

### Panel meeting

#### What is the overall quality of evidence regarding outcomes in included studies?

The results of this meta-analysis must be cautiously applied to clinical practice as it includes studies that included “critically ill patients on mechanical ventilation” not “patients with ARDS” resulting in the inclusion of a large variety of patients, including those in medical, surgical and neurological ICUs. The heterogeneity of the analysis is high (p=0.01, I2=55%) leading to downgrading of the evidence. As none of the studies was blinded due to their design, the possibility of having an impact on outcomes cannot be excluded and the risk of bias is high. As a result, the confidence level on the overall quality of evidence was rated as “very low”.

#### What about the balance between benefits and harms?

The benefits are expected to overweigh the harms, as the initiation of liberation protocols is less likely to increase the patients’ burden or incidence of adverse events.

#### What about patients’ values and preferences?

Since the patients’ burden and incidence of adverse events are not expected to increase by initiating liberation protocols, patients’ values and preference have little impact on the recommendation.

#### What about the balance between the net benefit and the costs or resources?

The cost of using liberation protocols is expected to be minimal except when protocols programmed into the ventilators are used. Development of protocols and education of staff to apply a protocol may incur some cost. The benefits are expected to outweigh the costs or resources needed when liberation protocols programmed into the ventilator are not used.

#### Grading of the recommendations

Some panelists questioned the primary outcome being the duration of mechanical ventilation rather than mortality. After an explanation from the SR committee that the most important result expected from the use of liberation protocols is a reduction in the duration of mechanical ventilation, it was agreed that the duration of mechanical ventilation is the most important outcome to be evaluated. Based on the results of this meta-analysis, all panelists agreed with the recommendation “We suggest using protocolized methods of liberation from mechanical ventilation in patients with ARDS”

Liberation protocols are divided into two groups, “step-wise reduction of mechanical ventilator support protocols” and “spontaneous breathing trial (SBT) protocols”. Another way to classify liberation protocols is to divide them into “professional-led protocols” where staff such as nurses or respiratory therapists change ventilator settings based on protocols and “computer-driven protocols” where the settings are changed automatically based on computer programs built into the ventilators. Although it was decided that another panel discussion be held to reassess the recommendation after subgroup analyses are conducted, the recommendation did not require any change based on the subgroup analyses.

### Description in other relevant clinical practice guidelines

There are no existing guidelines regarding protocols for liberation from mechanical ventilation in patients with ARDS. A ventilator card is available on the website of NIH-NHLBI ARDS Network (www.ardsnet.org/files/ventilator_protocol_2008-07.pdf) where SBT is used in the protocol. In Japan, three societies, the Japanese Society of Intensive Care Medicine, the Japanese Respiratory Society and the Japan Academy of Critical Care Nursing published “The protocol developed by the three societies for liberation from mechanical ventilation” (www.jsicm.org/pdf/kokyuki_ridatsu1503b.pdf) where it is stated that a simple and practical protocol which can be used in common by various professionals is required to facilitate daily interruption of sedation and early liberation from mechanical ventilation.

### Monitoring and evaluation during the management

In addition to respiratory and hemodynamic parameters, respiratory patterns and patient’s facial expressions need to be observed.

### Implications for future researches

Studies including only patients with ARDS are needed. It is also necessary to identify subgroups which may benefit more or less from liberation protocols.

### Methods for literature search and inclusion

We used the 17 studies included in the previous Cochrane systematic review [76] for studies published until March 31, 2012.

We searched PubMed, EMBASE, Cochrane CENTRAL and Igaku Chuo Zasshi (Japana Centra Revuo Medicina) from April 1, 2012 to June 30, 2015 with the keywords “weaning”, “liberation”, “mechanical ventilation” and “protocol”. A total of 1660 studies were identified. We included 12 randomized controlled trials in this guideline after screening.

### Randomized control trials included in this clinical question

Chaiwat 2010 [79], Kollef 1997 [80], Ely 1996 [81], Krishnan 2004 [82], Marelich 2000 [83], Namen 2001 [84], Navalesi 2008 [85], Piotto 2011 [86], Roh 2012 [87], Rose 2008 [88], Simeone 2002 [89], Stahl 2009 [90]

### CQ7: Should prone positioning be used in adult patients with ARDS?

**Table:** 

**Recommendation: We suggest prone positioning in adult patients with ARDS (especially in patients with moderate to severe respiratory dysfunction). (GRADE 2C, Strength of recommendation “weak recommendation” / Quality of evidence “low”)**
●**Supplementary conditions:** Experienced staff are required to perform prone positioning. The effect of prone positioning may be insufficient if performed for a short time, for example, if sufficient personnel are available only for a limited period of time. Knowing the capabilities of the treating facility, especially staffing levels, is required.

### Background, the priority of this issue

Prone positioning is effective in patients with ARDS because of physiologic improvement in respiratory mechanics, oxygenation, and hemodynamics or prevention of VILI [91, 92]. Although many RCTs and meta-analyses regarding prone positioning in patients with ARDS have been conducted, the results are not consistent, and therefore, the benefits of prone positioning in patients with ARDS are not clearly defined [93–97]. Since prone positioning is performed without specialized equipment, examining the effectiveness of prone positioning for ARDS is a high priority.

### Description

#### Summary of Evidence

We conducted a systematic review of RCTs regarding prone positioning in adult patients with ARDS. In a meta-analysis of eight RCTs, prone positioning significantly reduced the mortality (RR 0.77, 95%CI 0.62〜0.96). In a subgroup analysis of four RCTs which addressed patients with moderate and severe ARDS (P/F ≤ 200), the mortality was significantly reduced (RR 0.71, 95%CI 0.52〜0.97). In a subgroup analysis of six RCTs which addressed prolonged prone positioning (≥8 hours), although a similar tendency was shown, there was no significant difference between prone and supine positioning (RR 0.77, 95%CI 0.58〜1.02). In addition, although prone positioning did not increase the incidence of significant adverse events such as endotracheal tube complications (RR 1.29, 95%CI 0.87〜1.91), it significantly increased the incidence of decubitus ulcers (RR 1.36, 95%CI 1.06〜1.75).

### Panel meeting

#### What about the quality of evidence concerning overall outcomes?

All studies included in the meta-analysis are RCTs. RCTs examining mortality generally had a low risk of bias but had inconsistency. There was no serious indirectness or imprecision. Publication bias could not be assessed because of the small number of studies. The certainty of the evidence of effects of prone positioning for adult patients with ARDS on mortality was evaluated as “moderate”. In a subgroup analysis focusing on patients with moderate and severe ARDS, the sample size was small. In a subgroup analysis focusing on prolonged prone positioning, the confidence interval crossed the clinical decision threshold. Thus, the certainty of the evidence for these two subgroup analyses is evaluated as “low” because of imprecision.

Since the meta-analysis addressing endotracheal tube complications included RCTs with a risk of bias that was not low and showed imprecision, the certainty of the evidence is evaluated as “low”. The meta-analysis addressing decubitus ulcers included RCTs with a low risk of bias and did not show serious inconsistency or indirectness. However, because of the small number of studies and imprecision, the certainty of the evidence is evaluated as “moderate”. Thus, the overall certainty of the evidence is evaluated as “low”.

#### What about the balance between benefits and harms?

Although prone positioning significantly increases the incidence of decubitus ulcers, mortality is reduced without a significant increase in the incidence of significant adverse events such as endotracheal tube complications. Therefore, the benefit is greater than the harm.

#### What about the values and preference of the patients?

Nearly all patients can tolerate being in the prone position. However, some patients may refuse it if they are forced to be in the prone position for a long time for therapeutic purposes.

#### What about the balance between the benefit and the cost or resources?

Changing a patient to the prone position requires more manpower than usual. Although there is a specialized bed that can reduce this burden in terms of manpower needed (e.g. RotoProne bed®), the bed is expensive and is not approved in Japan. In addition, prone positioning requires more vigilant monitoring than a supine patient. However, even when considering the added burdens in manpower and cost, prone positioning significantly reduces mortality without an increase in the incidence of severe adverse events. Therefore, the benefit of prone positioning is greater than the burden in cost or resources.

#### Grading of recommendation

At the panel meeting, prone positioning for adult patients with ARDS was considered preferable because its use reduced the mortality rate without increasing the incidence of significant adverse events. Among panelists, there was an opinion that a “strong recommendation” is more preferable with emphasis on the effect of reduction in mortality. However, the certainty of the evidence is low and prone positioning requires experience. Additionally, implementation rates differ greatly among facilities. Thus, the panel concluded that the use of prone positioning for adult patients with ARDS is a “weak recommendation”. As a supplemental explanation, this recommendation does not mean that prone positioning should be restricted to a certain facility with personnel who have extensive experience in the use of this approach. Rather, organizing a system, including a sufficient number of staff and educating that staff, for providing prone positioning at any facility is important. In subgroup analyses, although the estimate of the effect of prolonged prone positioning (≥8 hours) was similar, there was no statistically significant difference. However, the effect of prone positioning for patients with moderate and severe ARDS (P/F ≤ 200) was expected to be greater.

### Description in other relevant clinical practice guidelines

The recommendation of prone positioning for adult patients with ARDS in the Scandinavian adult ARDS clinical practice guidelines is consistent with our guidelines, with a “weak recommendation” (the certainty of the evidence: low) [5]. In the Scandinavian guidelines, patients included in the analyses were restricted to prone positioning no more than 12 hours per day and prone positioning significantly reduced the 28-day mortality (meta-analysis of 3 RCTs: RR 0.85, 95%CI 0.73〜0.99) and 90-day mortality (meta-analysis of 3 RCTs: RR 0.86, 95%CI 0.74〜0.98). Endotracheal tube complications and the incidence of decubitus ulcers were not assessed in the Scandinavian guidelines.

### Monitoring and evaluation of treatment

Increase in blood pressure and heart rate due to stimulation, decrease in blood pressure due to fluid shifts, arrhythmias, change in tidal volume or airway pressure due to a decrease in lung-thorax compliance, obstruction, malposition, or unplanned removal of the endotracheal tube, aspiration of oral secretions, flexion or accidental dislodgement of tubes and lines, compression injuries of the eyes or external genitals, decubitus ulcers, peripheral neuropathy, vascular insufficiency of skin.

### Possible future studies

Investigation of long-term mortality and functional prognosis as well as short-term mortality is required. In addition, studies to determine the optimal subject (severity) or optimal method (i.e., duration or repetition) of prone positioning is required.

### Literature search method and literature selection

We searched PubMed, EMBASE, Cochrane CENTRAL, and Ichushi with keywords “ARDS” and “prone”. Of the 592 identified studies, 8 RCTs were included in meta-analyses after screening.

### RCT articles included in this CQ

Beuret 2002 [98], Fernandez 2008 [99], Gattinoni 2001 [100], Guerin 2004 [101], Guerin 2013 [102], Mancebo, 2006 [103], Taccone, 2009 [104], Voggenreiter 2005 [105]

### CQ8: Should High Frequency Oscillation be used in adult patients with ARDS?

**Table:** 

**Recommendation: We suggest against the use of High Frequency Oscillation (HFO) in adult patients with ARDS. (GRADE 2C,Strength of recommendation “weak recommendation” / Quality of evidence: ”low”)**
ᅟ

### Background, the priority of this issue

It is important to avoid ventilation-related lung injuries, which may lead to prolonged ventilation or an increased mortality rate, in patients with ARDS, by using proper ventilation strategies [65]. The mortality rate due to ARDS is still high, despite enormous efforts and multiple studies to define lung protective strategies [2, 106].

HFO is an artificial ventilation mode, which can restrict the ventilation tidal volume as well as provide a lung recruitment effect [107]. HFO has been recognized to provide lung protection, however, it is still not commonly used in adult intensive care [108]. Further studies are necessary to determine its effectiveness and safety. However, we conclude that this should not be prioritized to be resolved at the present time.

### Description

#### Summary of Evidence

Four randomized controlled trials (RCT) were found for evaluating HFO in adult patients with ARDS. There was no statistically significant difference in mortality rate and ventilation free days (VFD) by utilizing HFO; mortality rate (Relative Risk (RR): 1.05, 95% Confidence Interval (CI): 0.82 - 1.36) and VFD (mean difference: -0.30 days, 95%CI: -1.23 - 0.64). Although there is no statistically significant difference, the results show an increasing trend in the incidence of barotrauma in patients treated with HFO (RR: 1.21, 95%CI: 0.83 - 1.36).

### Panel meeting

#### What is the overall quality of evidence across outcomes?

Studies which use ventilation settings as an intervention cannot be performed in a double blinded manner, and the selected studies were no exceptions. The high likelihood of critical selection biases was estimated for all selected studies. One study was conducted with an intent-to-treat-analysis, however, a cross-over RCT was used as the design [109]. One study did not provide a patient flow diagram which can be important to decide the quality of the study [110]. The other two studies excluded more than half of the initial participants without a clear description of selection criteria in the manuscript [111, 112]. Heterogeneity among studies can be high with I2 statistics of 69%. Indirectness in the four selected studies was not sufficiently obvious to lower the evidence grade, given other factors. Precision was considered to be low given the wide 95%CIs, although the number of included cases seemed adequate. Due to the small number of studies selected, publication bias could not be examined. In conclusion, the overall quality of evidence was concluded as low. This included very low for mortality, low for VFD, and very low for barotrauma, respectively.

#### What is the balance between benefits and harms?

Given that most available ventilators cannot provide a HFO mode, in order to introduce HFO as a new modality, a facility needs to invest a large amount of money. Benefits are estimated to be relatively small even with increased spending, considering the results of this review. Although there was an increasing tendency for the development of barotrauma in the HFO group, no statistically significant difference could be seen, hence, the potential for harm secondary to HFO is concluded as low.

#### What are the values and preferences of the patients?

HFO is not a first-line choice in the management of patients with ARDS, and only a limited number of facilities can provide this modality. Since relatively deep sedation is required to use HFO in adult patients, values and preferences can vary among patients.

#### What about the balance between the net benefit and the cost or resources?

The overall benefit for patients by introducing HFO is likely not to be great, based on the results of this review. Furthermore, the estimated cost increase likely exceeds the potential benefits except in facilities which already have access to HFO.

#### Grading of recommendation

In the discussion prior to the panel meeting, it was decided not to propose HFO (weak recommendation), however, a revote was conducted, taking into account the potential benefits and drawbacks described in the previous sections. As a result, it was proposed not to use HFO (weak recommendation); four answered for “not to recommend” (strong recommendation) and eight voted “not to propose” (weak recommendation) respectively.

This recommendation was made considering facilities which already have access to HFO. We emphasize that this recommendation is for adult patients. HFO is not commonly used in current practice in adult patients with ARDS, compared to pediatric patients or neonates. HFO can cause harm if used incorrectly. However, it can provide better outcomes with less complications when applied appropriately.

### Description in other relevant clinical practice guidelines

The Scandinavian clinical practice guideline in 2014 concluded “not to recommend” HFO (strong recommendation), given the results of a Cochrane systematic review in 2013 [5, 113].

### Monitoring and assessment of treatment

Standard monitoring for oxygenation status, ventilation, and work of breathing are sufficient. It is not feasible to examine ventilation using tidal ventilation volume, end-tidal CO2, or lung sounds in patients receiving HFO, thus alternative monitoring is necessary.

### Possible future studies

Two of the selected studies [111, 112] adopted a P/F ratio ≤ 200 as the inclusion criteria, therefore, a significant number of patients with moderate ARDS are included, which might dilute the effects of HFO. The effect of HFO in patients with severe ARDS, which is unable to be managed with conventional lung protective strategies, should be evaluated in future studies.

### Literature search method and literature selection

We searched PubMed, EMBASE, Cochrane CENTRAL, and Igaku Chuo Zasshi (Japana Centra Revuo Medicina) with the keywords “ARDS”, “RCT”, and “HFO”. There were 304 studies identified. After screening these studies, we included four randomized controlled trials in the analysis of this clinical question.

### RCTs included in this CQ

Derdak 2002 [109], Bollen 2005 [110], Young 2013 [111], Ferguson 2013 [112].

### CQ9:Should neuromuscular blocking agents be used in adult patients with ARDS requiring mechanical ventilation?

**Table:** 

**Recommendation: We suggest the use of neuromuscular blocking agents (NMBAs) in adult patients with ARDS requiring mechanical ventilation, under certain circumstances. (GRADE 2B, Strength of recommendation “weak recommendation” / Quality of evidence “moderate”)**
●**Supplementary conditions:** The routine use of NMBAs should be avoided. Their use would be justified only if the Berlin definition of ARDS is fulfilled for patients with moderate or severe ARDS (P/F</=200 on PEEP of >/=5cmH2O). We would also limit their use to less than 48 hours in the early phase of the disease. The NMBAs currently available in Japan have some risks for causing myopathy. In particular, the concurrent use of steroids increases the risk, which should be taken into account [114–116]. NMBAs are generally categorized into depolarizing agents and non-depolarizing agents based on their pharmacologic mechanism. Compared to non-depolarizing agents, depolarizing agents have more side effects such as myalgia, hyperkalemia, and elevated intracranial pressure. Therefore, non-depolarizing agents are preferable in clinical practice. Non-depolarizing NMBAs are further classified into aminosteroids (Rocuronium, Vecuronium, Pancuronium) and benzylisoquinolines (Atracurium, Cisatracurium, Mivacurium) on the basis of their chemical structure. Cisatracurium, which was used in all three RCTs analyzed in this systematic review, is not available in Japan. Rocuronium or vecuronium are alternatives. However, special consideration is required. While the metabolism of benzylisoquinolines such as cisatricurium is not influenced by hepatic or renal function, the metabolism of aminosteroids such as rocuronium or vecuronium is delayed in patients with hepatic or renal dysfunction. In addition, attention needs to be paid to the risk of muscular atrophy due to aminosteroid use. There was a suggestion given by one of the panelists that the routine use of NMBAs should not be recommended because NMBAs currently available in Japan may increase the risk of myopathy. After extensive discussion among the panelists, agreement was reached to make a weak recommendation for their use under certain circumstances, as described in the comments.

### Background, the priority of this issue

Recent studies suggest that treatment modalities preserving spontaneous breathing prevent ICU-acquired weakness and ventilation-perfusion mismatch in patients with ARDS [117]. However, several studies suggest that excessive stress in alveoli due to spontaneous breathing impairs alveolar stability, which may contribute to the poor prognosis in patients with ARDS [118]. The decision to preserve spontaneous breathing or to decrease/prohibit spontaneous breathing by using neuromuscular blockers may have opposite effects on the prognosis in patients with ARDS so the priority of this clinical question is high.

### Description

#### Summary of Evidence

All three RCTs analyzed in this systematic review were conducted by the same French group which studied the efficacy of NMBAs in adult patients with ARDS requiring mechanical ventilation [119–121]. All cohorts fulfilled the criterion of having moderate or severe ARDS (P/F</=200 on PEEP of >/=5cmH2O) based on the Berlin definition. NMBA use was limited to less than 48 hours from the onset of the disease. Meta-analysis of these 3 RCTs (total 431 patients) demonstrated that the ICU mortality, 28-day mortality, and the rate of barotrauma are significantly lower in the NMBA group compared to the control group (ICU mortality: RR 0.70, 95%CI 0.55-0.89; 28-day mortality: RR 0.68, 95%CI 0.50-0.91; the rate of barotrauma: RR 0.43. 95% CI 0.20-0.90). There is no statistically significant difference between the two groups regarding the occurrence of myopathy due to NMBA use.

### Panel meeting

#### What is the overall quality of evidence across outcomes?

All three RCTs demonstrated that the NMBA-treated groups had a consistent, significant improvement in mortality compared to control groups [119–121]. The statistical significance was also confirmed by meta-analysis (I2=0% in all outcomes). Although complete concealment of the study drug was not possible due to its pharmacologic characteristics, the possibility of other risk of biases was considered to be low. There was no major issue in selection of the study population or outcome measurement. However, the level of recommendation was downgraded, because cisatracurium, used in these three RCTs, is currently not available in Japan, and as a result, indirectness of these studies is considered serious. The ICU mortality and 28-day mortality were 163/431 (38%) and 123/395 (31%), respectively, and the number of events was considered sufficient to provide precise effect estimates. We need a special caution here for the following reasons before interpreting the results. First, all three RCTs analyzed in this meta-analysis were conducted by the same French study group. Second, the Papazian 2010 study enrolled a much larger cohort compared to the other studies [121]. As a result, this study might have a disproportionate impact on the results. The number of patients with barotrauma and myopathy was either quite low or not assessed in the other two RCTs. Therefore, when all three RCTs are compared to the Papazian study alone, the outcomes are similar.

#### What is the balance between benefits and harms?

Since a certain degree of benefit is expected with NMBAs, use without serious complications, treatment with NMBAs will be accepted by most patients. However, we recognize that cisatracurium, the drug used in the RCTs, is not available in Japan.

#### What are the values and preferences of the patients?

The recommendation “We suggest the use of neuromuscular blocking agents (NMBAs) in adult patients with ARDS requiring mechanical ventilation, under certain circumstances” was unanimously approved by all panelists including patient representatives, suggesting no significant biases in the preferences of patients. However, as mentioned in the supplementary conditions, the NMBA used in these three RCTs, cisatracurium, is not available in Japan, which may induce biases in the preferences of patients.

#### What about the balance between the net benefit and the cost or resources?

Systematic review demonstrated that the use of NMBAs in adult patients with ARDS requiring mechanical ventilation reduced the ICU mortality, 28-day mortality and the rate of barotrauma. However, there was no statistically significant difference between the two groups regarding the occurrence of myopathy due to NMBA use. The use of NMBAs is feasible with no difficulties, requiring very limited equipment. The net benefits outweigh the costs. However, specific considerations are necessary for the use of NMBAs, because cisatracurium is currently not available in Japan.

#### Grading of recommendation

The use of NMBAs in adult patients with ARDS requiring mechanical ventilation reduced the ICU mortality, 28-day mortality and the rate of barotrauma. However, there is no statistically significant difference between the two groups regarding the incidence of myopathy, due to NMBA use. Therefore, the statement “We suggest the use of neuromuscular blocking agents (NMBAs) in adult patients with ARDS requiring mechanical ventilation” was recommended by the panel. However, the NMBAs currently used in Japan are aminosteroids, which can increase the risk of muscular atrophy with a long duration of use. The concurrent use of steroids further increases the risk. After extensive discussion among the panelists, the weak recommendation for use of NMBAs under certain circumstances with additional comments regarding routine use, limited use for 48 or fewer hours in the early phase of the disease and caution regarding concomitant use of steroids was unanimously approved by all panelists.

### Description in other relevant clinical practice guidelines

There are no statements regarding the use of NMBAs in adult patients with ARDS requiring mechanical ventilation in the Scandinavian clinical practice guideline on mechanical ventilation in adults with ARDS [5] or the Japanese guidelines for the management of sepsis [59]. In SSCG 2012 [58], the use of NMBAs for 48 or fewer hours is weakly recommended in patients with early, sepsis-induced ARDS with a PaO2/FIO2 less than 150 mmHg (Grade 2C). The rationale for this statement is based on the same three RCTs in the current guideline. The recommendation in this review and the statement in the SSCG 2012 are quite similar.

### Monitoring and assessment of treatment

Respiratory and circulatory monitoring, neuromuscular monitoring with train-of-four (TOF) stimulation,and sedative monitoring (BIS®: Bispectral Index) are necessary to evaluate the adequacy of neuromuscular blockade.

### Possible future studies

For patients who fulfill the Berlin definition for mild ARDS, the safety and efficacy of cisatracurium, as well as vecuronium, pancuronium, and rocuronium need to be assessed in further clinical trials.

### Literature search method and literature selection

We searched PubMed (through May 8, 2015), EMBASE (through June 1, 2015), Cochrane CENTRAL (through May 8, 2015) and Igaku Chuo Zasshi (Japana Centra Revuo Medicina) (through May 8, 2015) with keywords “neuromuscular blockade”, ”neuromuscular blocking agents”, ”muscle Relaxants”, ”neuromuscular blocke”, ”neuromuscular blockade”, ”neuromuscular blocking agent*”, ”muscle relaxant”, ”paralytics”, ”respiratory paralysis”, ”vecuronium”, ”pancuronium”, ”rocuronium”, ”atracurium”, ”cisatracurium”, ”succinylcholine”, ”curare”, ”rapacuronium”, ”mivacurium”, ”mivacron”, ”tracrium”, ”doxacurium”, ”nuromax”, ”nimbex”, ”norcuron”, ”zemuron”, ”pavulo”, ”tubocurarine”, ”gallamine”, ”flaxedil”, ”pipecuronium”, ”alcuronium”, ”toxiferine”, ”suxamethonium”, ”raplon” and ”jexin”. There were 1692 studies identified. After screening these studies, we included 3 randomized controlled trials in the analysis of this clinical question.

### RCTs included in this CQ

Forel 2006 [119], Gainnier M 2004 [120], Papazian 2010 [121]

### CQ10:How should fluid balance be maintained on a daily basis in adult patients with ARDS?

**Table:** 

**Recommendation: We suggest fluid restriction in the management of adult patients with ARDS. (GRADE 2B,Strength of recommendation “weak recommendation” / Quality of evidence ”moderate”)**
●**Supplementary conditions:** It was considered that there is no evidence for optimal indicators or target values for fluid management, as well as methods of fluid restriction, in adult patients with ARDS. Two RCTs to evaluate indicators for fluid management comparing extravascular lung water and PAWP or CVP have been recently reported, but the mortality rate was not improved in these studies. In addition, although the duration of mechanical ventilation was shortened in one study [122], the usefulness was not particularly shown in the study [123].

### Background, the priority of this issue

In patients with ARDS, pulmonary edema is caused by vascular endothelial dysfunction or increased vascular permeability [124]. A positive fluid balance in patients with ARDS increases the mortality rate [125]. Extravascular lung water content is associated with disease severity and mortality [126].

However, there is no previous RCT that reported an improvement in mortality rate by changing the fluid management in patients with ARDS. It has not been established how fluid balance is maintained in patients with ARDS despite the fact that the importance of optimal reduction in fluid volume is well known, and this goal is sought in daily clinical practice. Therefore, the priority of this issue is considered to be high.

### Description

#### Summary of Evidence

As a result of a systematic review, three RCTs comparing adult patients with ARDS who underwent fluid restriction with patients who were not fluid restricted were found [127–129]. A study that examined the infused fluid volume in patients with shock in addition to patients with ARDS was excluded. While FACTT 2006 included a large number of patients, the other two studies included a small number. There was no significant difference in short-term mortality, but VFD was significantly increased (+2.5 days) in patients who underwent fluid restriction. There was no difference in the need for renal replacement therapy within 60 days.

### Panel meeting

#### What about the quality of evidence regarding outcome in general?

There is no large-scale study that evaluates this CQ other than FACTT 2006, which is a large-scale multi-center study. As a result, two RCTs were included in the meta-analysis for mortality and only FACTT 2006 was included in the meta-analysis for other outcomes. Although FACTT 2006 was insufficiently blinded, it has a low risk for other biases and a sufficient number of patients. Inconsistency in the mortality rate between the studies was low (I^2^=0%), but Martin 2002 included only 37 patients while FACTT 2006 included 1000 patients. Indirectness was classified as ‘not serious’ because the result of FACTT 2006 is well matched to the PICO in this CQ. However, imprecision was classified as ‘serious’ because the confidence interval overlaps with the clinical decision threshold. Based on the above discussion, the overall quality of evidence was evaluated as ‘moderate’.

#### What about the balance between benefits and harms?

In patients who were fluid restricted, the need for renal replacement therapy was not increased although the mortality rate was not decreased. It is considered that the benefits to be obtained are greater than the harms because the duration of mechanical ventilation is expected to be shortened. If furosemide is used, there is a risk of electrolyte abnormalities.

#### What about the values and preferences of the patients?

Fluid restriction with diuretics is commonly used with little difference among health care facilities. Although the mortality rate was not improved, the number of VFD was increased and the need for renal replacement therapy was not increased by fluid restriction. Therefore, this intervention is likely to be accepted by most patients. Also, the values regarding these outcomes are less likely to vary among patients.

#### What about the balance between the net benefit and the cost or resources?

While fluid management, including the use of diuretics, is practiced in many health care facilities, there are a variety of diuretics. In the selected studies for this CQ, furosemide is most commonly used. Not only is furosemide a low-cost drug, but its use can also shorten the duration of mechanical ventilation without increasing the need for renal replacement therapy. Based on these reasons, it is considered that the benefits to be obtained are greater than the harms.

#### Grading of recommendation

In the discussion at the panel meeting, fluid restriction was supported based on the results of the meta-analysis. Although the mortality rate was not improved, the VFD was increased and the need for renal replacement therapy was not increased. However, since there is no high-quality study other than FACTT 2006 and there is no improvement in short-term mortality, we unanimously decided to make the recommendation that “we suggest fluid restriction in the management”.

### Description in other relevant clinical practice guidelines

According to SSCG 2012, “a conservative fluid strategy is recommended for ARDS patients with sepsis, who do not have evidence of tissue hypo-perfusion” [58]. However, in this guideline we decided to describe it as “we suggest” rather than “we recommend” because there is currently no large-scale study except for FACTT 2006, and no improvement in mortality rate was observed in the systematic review.

### Monitoring and evaluation of treatment

In the sub-group analysis in FACTT 2006, there was no obvious difference between patients with a central venous catheter and those with a pulmonary artery catheter. Therefore, monitoring with a pulmonary artery catheter is not always required. Although there are other indicators including extravascular lung water content, cerebral natriuretic peptide level, and weight, there is no obvious answer regarding which measurement is more useful and what target value is appropriate for each measurement.

When using furosemide, electrolytes should be carefully monitored for abnormalities such as hypokalemia.

### Possible future studies

Further study is required to determine which measurement is useful and what target value is appropriate for each measurement.

In addition, another study may be needed to examine the optimal diuretic medication and infusion fluid.

The study that followed the patients in FACTT 2006 up to 12 months suggests that management with fluid restriction might be a risk factor for cognitive dysfunction [130]. Therefore, an additional study to examine long-term outcomes are also necessary.

### Literature search method and literature selection

We searched PubMed, EMBASE, Cochrane CENTRAL and Igaku Chuo Zasshi (Japana Centra Revuo Medicina) with keywords “ARDS” and “fluid management” to May 17, 2015. There were 3160 studies identified. After screening these studies, we included 2 randomized controlled trials in this guideline.

### RCT articles included in this CQ

FACTT 2006 [127], Martin 2002 [128], Mojtahedzadeh 2005 [129]

### CQ11:Should neutrophil elastase inhibitors be used in the treatment of adult patients with ARDS?

**Table:** 

**Recommendation: We do not suggest the use of neutrophil elastase inhibitors in adult patients with ARDS. (GRADE 2D, Strength of recommendation “weak recommendation” / Quality of evidence “very low”)**
●**Supplementary conditions:** Neutrophil elastase inhibitors are reimbursed by the national health insurance system in Japan to treat patients with ARDS with the proviso that the use of neutrophil elastase inhibitors is not recommended in patients with multiple organ failure (four or more organs), burn injuries, or trauma. A nationwide survey conducted by the Japanese Respiratory Society in 2010 showed that neutrophil elastase inhibitors are widely used in Japan for the treatment of patients with ARDS.

### Background, the priority of this issue

The pathogenesis of acute respiratory distress syndrome (ARDS) is pulmonary edema caused by increased permeability associated with nonspecific alveolar inflammation. Neutrophil elastase is thought to be one of the most important mediators related to the pathogenesis of ARDS [131, 132]. A neutrophil elastase inhibitor, available for clinical use in Japan, has been intensively investigated as a treatment to improve the prognosis of patients with ARDS. Several meta-analyses showed that the use of a neutrophil elastase inhibitor did not improve mortality [133], while other studies suggested potential benefits [134]. This discrepancy in results indicates the importance of this issue. Since a neutrophil elastase inhibitor is reimbursed by the national health insurance system in Japan and is widely administered in Japan, the priority of this issue is high.

### Description

#### Summary of Evidence

A total of six randomized clinical trials (RCTs, 815 patients) were selected in a systematic review. Meta-analysis demonstrated that neutrophil elastase inhibitors did not improve the short-term (<90 days) mortality (RR 0.92, 95%CI 0.64-1.32), the rate of severe adverse effects (RR 0.79, 95%CI 0.47-1.34) or number of ventilation-free days (VFD) (Mean 1.58 days more, 95%CI 2.72 days fewer to 5.89 days more).

### Panel meeting

#### What is the overall quality of evidence across outcomes?

Many studies had a high risk of bias in blinding. Moderate to severe inconsistency was observed for (short-term (<90 days) mortality, I2 = 31%; and severe adverse effects, I2 = 31%; VFD, I2 =86%). No indirectness was observed. Since the number of patients was less than optimal for the information size resulting in a large 95%CI, the imprecision of this meta-analysis was high. Publication bias could not be determined because of the small number of reported studies.

#### What is the balance between benefits and harms?

Systematic review demonstrated that neither efficacy nor significant adverse effects were found. The benefit was considered to be low compared to the increase in cost.

#### What are the values and preferences of the patients?

The recommendation “We do not suggest the use of neutrophil elastase inhibitors in adult patients with ARDS” was unanimously approved by all panelists including the patient representative, suggesting no significant biases in preferences of the patients. However, as mentioned in the supplementary conditions, the neutrophil elastase inhibitor is currently approved for reimbursement by the health insurance system in Japan, and the meta-analysis demonstrated no significant increase in the number of significant adverse events, which may induce bias in the preferences of patients.

#### What about the balance between the net benefit and the cost or resources?

Systematic review did not demonstrate that the use of neutrophil elastase inhibitors in adult patients with ARDS reduced the short-term (<90 days) mortality, the rate of severe adverse effects or number of VFD. Although the use of neutrophil elastase inhibitors is feasible with no difficulties, requiring very limited equipment, they are expensive. The net benefits outweigh the costs.

#### Grading of recommendation

The use of neutrophil elastase inhibitors in adult patients with ARDS did not reduce either the short-term (<90 days) mortality or the rate of significant adverse events. However, it is currently approved for reimbursement by the national health insurance system, and is widely used for treating ARDS in many institutions in Japan. Based on this background information the statement “We do not suggest the use of neutrophil elastase inhibitors in adult patients with ARDS” was recommended at the panel meeting. Thereafter, however, analysis of the number of VFD as one of the main outcomes was recommended. The additional analysis did not demonstrate that the use of neutrophil elastase inhibitors increased the number of VFD. Based on these results, a second vote was conducted on-line, which unanimously concluded that the statement “We do not suggest the use of neutrophil elastase inhibitors in adult patients with ARDS” is recommended.

### Description in other relevant clinical practice guidelines

There are no statements about the use of neutrophil elastase inhibitors in the Scandinavian clinical practice guideline on mechanical ventilation in adults with ARDS (2015) or the section of ARDS in SSCG 2012 [5, 58]. The Japanese guidelines for the management of sepsis (2012) recommended that the use of neutrophil elastase inhibitors can be considered for adult patients with ARDS (Grade 2C) [59], which was not based on a systematic review.

### Monitoring and assessment of treatment

Cardiorespiratory monitoring and blood tests are necessary to identify the onset of adverse effects.

### Possible future studies

Due to the limited number of high-quality RCTs, large-scale, high-quality clinical trials are necessary to demonstrate the efficacy of neutrophil elastase inhibitors in adult patients with ARDS.

### Literature search method and literature selection

We searched PubMed (through June 13, 2015), EMBASE (through June 7, 2015), Cochrane CENTRAL (through July 21, 2015) and Igaku Chuo Zasshi (Japana Centra Revuo Medicina) (through June 13, 2015) with keywords including surfactant, prostaglandin, prostacyclin, acetylcysteine, neutrophil elastase, colony stimulating factor, nitric oxide, protein c, antithrombin, ulinastatin, macrolide, beta agonist, mesenchymal stem cell, ketoconazole, statin, thrombomodulin, ibuprofen, nafamostat, gabexate and lisofylline as therapeutic medicines except for corticosteroids and neuromuscular blockers. There were 7687 studies identified. After screening these studies, we included six randomized controlled trials in the analysis of this clinical question.

### RCTs included in this CQ

Endo 2006 [135], Kadoi 2004 [136], Nakayama 2013 [137], Shirai 2006 [138], Tamakuma 1998 [139], Zeiher 2004 [140]

### CQ12:Should steroids be used in adult patients with ARDS?

**Table:** 

**Recommendation:We suggest the administration of steroids (equivalent to methylprednisolone 1-2mg/kg/ day) to adult patients with ARDS. (GRADE 2B,strength of recommendation “ weak recommendation” / Quality of evidence “moderate”)**
●**Supplementary conditions:** There is one RCT to assess the effect of hydrocortisone (200mg/day) and 3 RCTs to assess the effect of methylprednisolone(1-2mg/kg/day). Therefore, we use “equivalent to methylprednisolone 1-2mg/kg/day” for the dose of steroids.

### Background, the priority of this issue

ARDS is defined as non-cardiogenic pulmonary edema, which may be caused by increased permeability due to epithelial and endothelial cell damage and neutrophil infiltration [141, 142]. Steroids, as anti-inflammatory therapy, may improve the pathologic changes associated with ARDS and a number of studies have assessed the risks and benefits of their use [143]. However, this treatment also has the potential to be detrimental to patients, and there is concern regarding an increased risk of infection. Therefore, this issue is a high priority in the management of adult patients with ARDS.

### Description

#### Summary of Evidence

There are a number of randomized controlled trials of steroid therapy in patients with ARDS, including the effect of low to middle dose steroids as such methylprednisolone 1-2mg/kg/day or hydrocortisone 200mg/day, and high doses such as methylprednisolone 120mg/kg/day. Steroid administration did not significantly decrease the mortality compared to placebo. However, it significantly increased the number of ventilator free days (VFD). In addition, steroid therapy did not significantly increase the incidence of infection. All randomized controlled trials to assess the VFD evaluated the effect of methylprednisolone 1-2mg/kg/day. In an RCT conducted by Bernard et al, it was shown that steroid therapy (methylprednisolone 120mg/kg/day) had a trend to increase the incidence of infection (odds ratio=1.57).

### Panel meeting

#### What is the overall quality of evidence across outcomes?

We collected RCTs to propose a recommendation on this issue. For mortality as outcome, we considered there is serious bias on inconsistency and imprecision. For the risk of infection, there is serious bias on the imprecision. There are few studies, and the publication bias is unclear. Therefore, the overall quality of evidence across outcomes is considered to be ”moderate”.

#### What is the balance between benefits and harms?

Steroid therapy is feasible in almost all facilities in Japan. Unfortunately, it is not evaluated in each study, but it is possible to delay the diagnosis of infections by administering steroids. In addition, a risk of side effects (hyperglycemia, infection, etc.) is associated with this treatment. There is also concern regarding ICU-related muscle weakness due to steroid use.

#### What are the values and preferences of the patients?

It is thought that there is no unevenness in the sense of values and the preferences of patients.

#### What about the balance between the net benefit and the cost or resources?

Steroid therapy is feasible in almost all facilities in Japan. There is no significant reduction of mortality with steroid therapy. Steroid therapy did not have a tendency to increase the risk of infection, and is expected to increase the number of VFD. Unfortunately, it is not evaluated in each study, but it is possible to delay the diagnosis of infections by using steroid. In addition, a risk of side effects (hyperglycemia, infection, etc.) is associated with this treatment. There is also concern regarding ICU-related muscle weakness due to steroid use

#### Grading of recommendation

In the first panel meeting, both mortality and the incidence of infection were reported. The committee proposed “We suggest not to use steroids in adult patients with ARDS”. Since not all participants agreed with this recommendation, a vote was conducted; one person supported the suggestion to use steroids, and eleven supported the suggestion not to use steroids. One person who supported not using steroids, had a specific concern regarding a lack of effect on the number of VFD. Therefore, the results regarding VFD as the outcome was reported again. After reporting the additional results about VFD, it was proposed that “We suggest the use of steroids in adult patients with ARDS”. A second vote was conducted. Ten people supported the suggestion to support using steroids, and two supported the suggestion not to use steroids. The opinions raised included “importance of VFD should not be high as mortality” and “the side effect of steroids which is not evaluate in SR was not negligible.”

The authors of RCTs which reported the benefit of steroids in adults with ARDS, reported that the delayed use of steroids, such as 14 days after the onset of ARDS, would be associated with increased mortality, thus, it was suggested to use steroids during the early stage of ARDS [144].

In a domestic survey conducted by a Japanese respiratory society in 2014, it was reported that many Japanese doctors (about one third of respondents) formerly gave 500-1,000 mg/day of methylprednisolone to patients with ARDS. However, there is no RCT which examines the risk or benefit of this therapy, and thus it could not be assessed. Accordingly, future studies are required to assess the impact of the dose or timing of steroid therapy in this cohort.

#### Result of two votes by the panel

Since there was no unanimous consensus, votes were conducted. In the second vote after reporting information regarding VFD, 10 people (83%) supported the recommendation to “suggest the use of steroids”. The panel finally concluded to recommend it. However, the panel also showed concerns such as “importance of VFD should not be high as mortality” and “the side effect of steroid which is not evaluate in SR was not negligible.”.

### Description in other relevant clinical practice guidelines

There is no guideline describing steroid therapy in patients with ARDS.

### Monitoring and assessment of treatment

Standard monitoring is sufficient. Careful evaluation for the development of secondary infections is required.

### Possible future studies

It is possible that the effects of steroid therapy in adult patients with ARDS may be different according to the timing of initiating therapy, dose, duration of treatment and the manner of tapering the dose. Thus, future RCTs are necessary in consideration of these points as well.

### Literature search method and literature selection

We conducted a systematic Medline, EMBASE, Cochrane CENTRAL and JAPAN medical abstract society search using the following keywords: “corticosteroid”, “respiratory distress”, “ARDS”, “acute lung injury”, pneumonia” and “lung edema” until 24th April 2014. We found 1132 articles and finally selected 5 RCTs.

### RCTs included in this CQ

Annane 2006 [145], Steinberg 2006 [146], Bernard 1987 [147], Meduri 1998 [148], Meduri 2007 [149]

### CQ13:Should the following drugs be used to treat adult patients with ARDS?

(inhaled nitric oxide (NO), inhaled/intravenous β2 stimulant, granulocyte macrophage colony-stimulating factor (GM-CSF), prostaglandin E1 (PGE1), statin, surfactant, activated protein C (APC), N-acetylcysteine (NAC), and ketoconazole or lisofylline)

**Table:** 

**Recommendation:** **We do not recommend using the following drugs to treat adult patients with ARDS (strength of recommendation “strong recommendation”).** **GRADE 1B Inhaled/ intravenous β2 stimulant, prostaglandin E1 (PGE1), activated protein C (APC), ketoconazole, and lisofylline (Quality of evidence ”moderate”)** **GRADE 1C Inhaled nitric oxide (NO) (Quality of evidence ”low”)** **GRADE 1D Surfactant (Quality of evidence ”very low”)** **We do not suggest using the following drugs to treat adult patients with ARDS (strength of recommendation “weak recommendation”).** **GRADE 2B granulocyte macrophage colony-stimulating factor (GM-CSF), N-acetylcysteine (NAC) (Quality of evidence ”moderate”)** **GRADE 2C Statin (Quality of evidence ”low”)**
**●Supplementary conditions:** These drugs are not approved for clinical use by the Japanese national health insurance system.

### Background, the priority of this issue

The pathogenesis of ARDS is non-cardiogenic pulmonary edema with increased permeability caused by nonspecific inflammation in the pulmonary alveolar space [150]. A number of factors including alveolar epithelial injury, increased pulmonary vascular resistance due to hypoxic pulmonary vasoconstriction, ventilation-perfusion mismatch and endogenous surfactant dysfunction are associated with the pathogenesis of ARDS. Therefore, a number of drugs have been investigated to treat ARDS including inhaled nitric oxide (NO)[151] as a pulmonary vasodilator, aerosolized/intravenous β2 stimulants [152–155] to resolve pulmonary edema, granulocyte-macrophage colony stimulating factor (GM-CSF) [156, 157] promoting growth of alveolar macrophages and alveolar epithelial cells, prostaglandin E1 (PGE1) [158, 159] as an anti-inflammatory agent, 3-hydroxy-3-methylglutaryl (HMG-CoA) reductase inhibitors including statin [160, 161], the antifungal drug ketoconazole [162, 163], lisofylline [164, 165], activated protein C (APC) [166, 167] which has anticoagulant and anti-inflammatory properties, and N-acetylcysteine (NAC) with antioxidant effects and exogenous surfactant supplementation to improve endogenous surfactant dysfunction. These agents have variable domestic availability, cost, and safety. If these agents are clinically indicated for the treatment of patients with ARDS, off-label use is mandatory in Japan. Due to the very limited number of effective agents for the treatment of ARDS, the priority of this problem is high.

### Description

#### Summary of Evidence

1. A total of 7 RCTs (699 patients) evaluating the efficacy of inhaled NO were selected in a systematic review. Meta-analysis demonstrated that inhaled NO is not associated with improvement in short-term (<90 days) mortality (RR 1.18, 95%CI 0.91-1.52) or the rate of significant adverse events (RR 1.90, 95%CI 0.78-4.66).
2. A total of 1 RCT (282 patients) evaluating the efficacy of inhaled β_2_ stimulant was selected in a systematic review. Meta-analysis demonstrated that inhaled β_2_ stimulant is not associated with an improvement in short-term (<90 days) mortality (RR 1.32, 95%CI 0.83-2.08), or the rate of significant adverse events (RR 2.57, 95%CI 0.85-7.76).
3. A total of 2 RCTs (365 patients) evaluating the efficacy of intravenous β_2_ stimulant were included in a systematic review. Meta-analysis showed that intravenous β_2_ stimulant is not associated with improvement in short-term (<90 days) mortality (RR 1.16, 95%CI 0.68-1.96). The rate of significant adverse events with administration of intravenous β_2_ stimulant was significantly increased (RR 5.78, 95%CI 1.34-24.92).
4. A total of 2 RCTs (148 patients) evaluating the efficacy of GM-CSF were selected for analysis in a systematic review. The meta-analysis showed that GM-CSF is not associated with an improvement in short-term (<90 days) mortality (RR 0.76, 95%CI 0.40-1.46), or rate of significant adverse events (RR 0.87, 95%CI 0.42-1.80).
5. A total of 8 RCTs (786 patients) evaluating the efficacy of PGE_1_ administration were included in a systematic review. The meta-analysis demonstrated that PGE_1_ is not associated with an improvement in short-term (<90 days) mortality (RR 1.07, 95%CI 0.90-1.27). However, PGE_1_ is significantly associated with an increase in the rate of significant adverse events (RR 2.07, 95%CI 1.12-3.83).
6. A total of 2 RCTs (1,284 patients) evaluating the efficacy of statin were selected for analysis in a systematic review. The meta-analysis demonstrated that statin did not have beneficial effects in terms of short-term (<90 days) mortality (RR 0.98, 95%CI 0.71-1.36) or the rate of significant adverse events (RR 1.36, 95%CI 0.69-2.67).
7. A total of 10 RCTs (2,894 patients) evaluating the efficacy of surfactant were included in a systematic review. The meta-analysis demonstrated that surfactant is not associated with an improvement in short-term (<90 days) mortality (RR 0.98, 95%CI 0.88-1.09), or rate of significant adverse events (RR 1.44, 95%CI 0.99-2.09).
8. A total of 2 RCTs (146 patients) evaluating the efficacy of APC were selected for analysis in a systematic review. The meta-analysis demonstrated that APC had no beneficial effects on short-term (<90 days) mortality (RR 0.64, 95%CI 0.21-1.95), or the rate of significant adverse events (RR 0.78, 95%CI 0.43-1.40).
9. A total of 4 RCTs (180 patients) evaluating the efficacy of NAC were included in a systematic review. The meta-analysis demonstrated that NAC is not associated with improvement in short-term (<90 days) mortality (RR 0.89, 95%CI 0.63-1.25). No RCT evaluated the rate of significant adverse events with the use of NAC.
10. A total of 1 RCT (234 patients) evaluating the efficacy of ketoconazole was selected for analysis in a systematic review. The meta-analysis demonstrated that ketoconazole did not improve the short-term (<90 days) mortality (RR 1.02, 95%CI 0.72-1.46), or the rate of significant adverse events (RR 1.25, 95%CI 0.74-2.12).
11. A total of 1 RCTs (235 patients) evaluating the efficacy of lisofylline was selected in a systematic review. The meta-analysis demonstrated that lisofylline had beneficial effects in terms of short-term (<90 days) mortality (RR 1.31, 95%CI 0.87-1.98). No RCT evaluated the rate of significant adverse events associated with the use of lisofylline.

#### What is the balance between benefits and harms?

Systematic review demonstrated that neither efficacy nor the rate of significant adverse effects was found for any drug except intravenous β2 stimulant and PGE1. The benefit was considered to be small compared to the increase in cost. However, intravenous β2 stimulant and PGE1 are associated with an increase in the rate of significant adverse events. With these medications, the benefit was considered to be small compared to the increase in cost.

#### What are the values and preferences of the patients?

1. The recommendation “We do not recommend or suggest using the following drugs in adult patients with ARDS (inhaled nitric oxide (NO), inhaled/intravenous β2 stimulant, granulocyte macrophage colony-stimulating factor (GM-CSF), prostaglandin E1 (PGE1), statin, surfactant, activated protein C (APC), N-acetylcysteine (NAC), or ketoconazole or lisofylline)” was unanimously approved by all panel members including the patient representative, suggesting no significant bias in the preferences of patients. However, as mentioned in the supplementary conditions, use of these drugs for the treatment of patients with ARDS is off-label in Japan, and not common therapeutic options. In addition, although GM-CSF, intravenous NAC, ketoconazole and lisofylline are not available in Japan, these four drugs do not increase the rate of significant adverse events, which may induce biases in the preferences of patients.
2. Intravenous β2 stimulant and PGE1 have no benefit in improving short-term (<90 days) mortality, but may increase the rate of significant adverse events. These consistent results will probably minimize the variation in patient values and preferences.

#### What about the balance between the net benefit and the cost or resources?

1. From the results of the systematic review, using these drugs has no effect on short-term (<90 days) mortality or the rate of significant adverse events in adult patients with ARDS except for intravenous β2 stimulant and PGE1. Since inhaled/intravenous β2 stimulant and statins are readily available at a reasonable price, the net benefits appear to outweigh the costs. However, since inhaled NO, surfactant and APC are expensive, the costs may outweigh the net benefits. Although GM-CSF, intravenous NAC, ketoconazole and lisofylline are not available in Japan, the costs probably outweigh the net benefits.
2. A systematic review demonstrated that the use of Intravenous β2 stimulant and PGE1 had no benefit in improved short-term (<90 days) mortality, but there is an increase in the rate of significant adverse events. The costs apparently outweigh the net benefits.

#### Grading of recommendation

1. In the systematic review, the use of inhaled NO has no benefit on the short-term (<90 days) mortality or the rate of significant adverse events, although inhaled NO is currently used in a substantial number of institutions in Japan. The panel reached the preliminary agreement “not to suggest”, rather than “not to recommend” the use of inhaled NO in adult patients with ARDS. However, since the panel took into consideration the fact that inhaled NO is not commonly used, nor is it approved for clinical use for treating patients with ARDS by the Japanese national health insurance system, the final statement was unanimously upgraded from “not to suggest” to “not to recommend”.
2. The use of inhaled β2 stimulant, surfactant and APC have no beneficial effects on short-term (<90 days) mortality or the rate of significant adverse events. These three drugs are neither frequently used nor approved for clinical use by the national health insurance in Japan. The statement, “We do not recommend using these drugs for the treatment of adult patients with ARDS” was unanimously adopted in the panel meeting.
3. The use of ketoconazole and lisofylline have no beneficial effects on the short-term (<90 days) mortality or the rate of significant adverse events. In addition, these two drugs are not available in Japan. The statement “We do not recommend the use of these drugs in the treatment of adult patients with ARDS” was unanimously adopted in the panel meeting.
4. The use of intravenous β2 stimulant or PGE1 has no beneficial effect on the short-term (<90 days) mortality, but is associated with an increased rate of significant adverse events. The statement “We do not recommend the use of these drugs for the treatment of adult patients with ARDS” was unanimously recommended in the panel meeting.
5. The use of GM-CSF, statin and intravenous NAC have no benefit on the short-term (<90 days) mortality or the rate of significant adverse events. Since the number of patients was smaller than optimal for the information size and therefore 95%CI was large, the imprecision of this meta-analysis was high. The statement “We do not suggest the use of these drugs in the treatment of adult patients with ARDS” was unanimously recommended in the panel meeting.

### Description in other relevant clinical practice guidelines

1. There are no statements regarding the use of inhaled NO, intravenous β2 stimulant, GM-CSF, PGE1, statin, surfactant, NAC, ketoconazole and lisofylline in the Scandinavian clinical practice guideline on mechanical ventilation in adults with ARDS (2015) [5], the Japanese guidelines for the management of sepsis (2012) [59] or the section regarding ARDS in SSCG 2012 [58].
2. The statement “we suggest that the use of inhaled β2 stimulant in adult patients with ARDS is limited to patients complicated by bronchospasm” was described in the section of ARDS in SSCG 2012 [58] which is consistent with current guidelines. APC was described in SSCG 2012 [58] with no recommendation. None of these drugs is described in the Scandinavian clinical practice guideline on mechanical ventilation in adults with ARDS (2015) [5] or the Japanese guidelines for the management of sepsis (2012) [59].

### Monitoring and assessment of treatment

Cardiorespiratory monitoring and blood testing are necessary to detect adverse events.

### Possible future studies

Because of a limited number of high-quality RCTs, large-scale, high-quality clinical trials are necessary to evaluate the efficacy of these medications.

### Literature search method and literature selection

We searched PubMed (through June 13, 2015), EMBASE (through June 7, 2015), Cochrane CENTRAL (through July 21, 2015) and Igaku Chuo Zasshi (Japana Centra Revuo Medicina) (through June 13, 2015) with keywords of surfactant, prostaglandin, prostacyclin, acetylcysteine, neutrophil elastase, colony stimulating factor, nitric oxide, protein c, antithrombin, ulinastatin, macrolide, beta agonist, mesenchymal stem cell, ketoconazole, statin, thrombomodulin, ibuprofen, nafamostat, gabexate and lisofylline as therapeutic medicines except for corticosteroids and neuromuscular blockers. There were 7687 studies identified. After screening these studies, we included 7 RCTs for inhaled NO, 1 RCT for inhaled β2 stimulant, 2 RCTs for intravenous β2 stimulant, 2 RCTs for GM-CSF, 7 RCTs for PGE1, 3 RCTs for statin, 10 RCTs for surfactant, 2 RCTs for APC, 4 RCTs for NAC, 1 RCT for ketoconazole, and 1 RCT for lisofylline in the analysis of this clinical question.

### RCTs included in this CQ

Dellinger 1998 [168], Gerlach 2003 [169], Mehta 2001 [170], Michael 1998 [171], Park 2003 [172], Taylor 2004 [173], Troncy 1998 [174], Matthay 2011 [175], Gao 2012 [176], Perkins 2006 [177], Paine 2012 [178], Presneill 2002 [179], Abraham 1996 [180], Abraham 1999 [181], Bone 1989 [182], Holcroft 1986 [183], Rossignon 1990 [184], Slotman 1992 [185], Vincent 2001 [186], Craig 2011 [187], McAuley 2014 [188], Truwit 2014 [189], Anzueto 1996 [190], Gregory 1997 [191], Kesecioglu 2009 [192], Markart 2007 [193], Spragg 2003 [194], Spragg 2004 [195], Spragg 2011 [196], Tsangaris 2007 [197], Weg 1994 [198], Willson 2015 [199], Cornet 2014 [200], Liu 2008 [201], Bernard 1997 [202], Jepsen 1992 [203], Ortolani 2000 [204], Suter 1994 [205], ARDS Network 2000 [206], ARDS Network 2002 [207]

## Discussion

We provide here a clinical practice guideline for adult patients with ARDS in the ICU. This article is an English version translated from the original Japanese version published as the ARDS clinical practice guidelines 2016 in July 2016. The assessment of the Japanese version by four external validation members using the AGREE II checklist showed that the overall quality of the guideline was 6.5 (average) (with lowest possible quality of 1 to highest of 7) and all members recommended this guideline for clinical use. The original one consisted of both narrative review part (Part 1), which described basic knowledge of epidemiology, pathophysiology, diagnosis, and common clinical practice regarding ARDS, and systematic review and recommendations part (Part 2) (http://www.jsicm.org/ARDSGL/ARDSGL2016.pdf). The present article solely focuses on Part 2, which was developed applying the GRADE system in performing systematic reviews and determining recommendations. We believe that Part 2 is one of the first guidelines strictly following the GRADE methodology in Japan.

Although most of the clinical questions evaluated here are commonly considered around the world, and some of the results are similar to previously published GRADE based guidelines [4, 5, 58], this guideline has some unique qualities. First, Japan is nearly racially homogeneous with a population of over 120 million and has a rather unique healthcare system of a culturally-specific nature. In Japan, there are several unique medications approved for therapeutic use in patients with ARDS, including sivelestat (neutrophil elastase inhibitor) [139], human recombinant thrombomodulin [208], gabexate mesilate, pulse therapy of glucocorticoid (ex. 1g methylprednisolone pulse per day for 3 days), polymyxin B-immobilized fiber, and edaravone (free radical scavenger). These therapies are widely used in patients with sepsis and ARDS in Japan, but none have been shown to have clear benefits in large-scale RCTs. The only therapy we included in the CQ was sivelestat, a widely-used protease inhibitor having anti-inflammatory property, since the effectiveness of sivelestat was examined by several RCTs although the quality of the studies is not high. Regarding the other therapies, no randomized control studies have been performed for patients with ARDS. While these therapies are being administered, the guidelines could be renewed by inclusion of future valid observational studies or RCTs, it is no doubt that the current guidelines, developed with relevant and valid methodology, is worth the effort to present standard therapies for patients with ARDS in Japan.

Second, as an outcome of the systematic reviews, we included “ventilator free days, VFD” as a “critical” outcome along with the mortality. In fact, vigorous efforts were made to collect data regarding VFD, including direct contact to the authors for obtaining original data [63, 112]. As a result, the overall recommendations are not entirely different from other guidelines except for the use of steroids in patients with ARDS. The Scandinavian guidelines by Claesson et al are against the use of steroids (2C) [4], whereas we favor their use (2B). At the panel discussion, eleven of twelve panelists first voted against the use of steroids based on the results of the mortality and the infection rate. However, one panelist commented that the panel should also consider VFD as it should be included in one of the critical outcomes from a practical point of view. Thus, we re-evaluated the results including VFD, which was significantly prolonged by the use of steroids. Considering the fact that the mortality was not significantly increased while no serious increase in the infection rate was seen, 10 panelists voted for the weak recommendation to use low dose steroids in patients with ARDS in the second polls.

As for the quality of evidence, after the publication of the Japanese version of this guideline, we received the comments that the inconsistency and imprecision should be determined as serious in each outcome of the CQ12. The commenters also suggested that the quality of evidence in CQ3, 4, 5, 8, 10 might be better to be reconsidered for the down-grading. Thus, we re-evaluated the recommendations of CQ10 and CQ12 where minor changes were made. However, as we have not yet re-convened the panel conference for the other CQs, we show here the original decisions. We cannot deny the possibility that the evaluation of the quality of evidence may change in future.

In the current guidelines, we acknowledge several limitations as follows:

1. As we only included RCTs for the systematic reviews and did not include observational studies, the number of studies was small and many of the RCTs were already reviewed by the previous meta-analysis or guidelines [4, 5, 58].
2. Clinicians may have substantial numbers of clinical questions on the diagnosis and the therapy of ARDS, but we limited the number of the questions to thirteen solely on the therapeutic measures and no information of diagnosis was provided.
3. The great heterogeneity of ARDS in nature makes clinical trials of therapeutic approaches for ARDS inevitably difficult. In the current guidelines, several clinical questions including CQ1 and CQ2, the study patients were not recruited solely from patients with ARDS, but from patients expected to require mechanical ventilation for a relatively long time. Although one can assume that substantial numbers of ARDS patients were included in this cohort, a resultant serious indirectness in performing this systematic review should not be ignored.
4. Since the durational definition of mortality varied among the RCTs, we integrated different durations between 28 and 90 days from the admission of ICU into “short-term mortality”. Thus, the obtained recommendations can be possibly affected by this durational integration. Differences in clinical practice between countries can also be a source of indirectness when making recommendation: e.g. cisatracurium is not available in Japan. As a result, considerable amounts of uncertainty could not be eliminated in the process of evaluating the quality of evidence in each outcome of CQs.
5. Although two GRADE working group members helped the guideline development process, few members have a full experience of the pertinent process of the guideline development. As a result, temporary confusion existed in the final determination process of the outcomes and the baseline risks of each CQ obtained from the systematic review process. Whether the results are affected by this confusion, or not, is unknown.

## Conclusions

In conclusion, we provide here a GRADE-based CPG for the management of patients with ARDS. The recommendations in the clinical practice guidelines should not be used to restrict management or force clinicians to strictly follow the recommendations in the guidelines, leaving room for clinical judgement. As considerable knowledge gaps exist in the management of ARDS, the effort to add the evidence available for future guidelines is critically important.

## Additional files

Additional file 1: contains Modified Preferred Reporting Items of Systematic Reviews and Meta-Analyses (PRISMA) flow-chart, risk of bias table, forest plots, evidence profiles, and evidence to decision table for each CQ according to the GRADE system. (PDF 32153 kb)

Additional file 2: contains tables disclosing intellectual and financial conflicts of interest for each person who participated in the creation of this guideline. (PDF 76 kb)

## Abbreviations

CI: Confidence interval
MD: Mean difference
RR: Risk ratio
Short Term Mortality: The definition varies among the CQs, and is thus defined in the tables
VAP: Ventilator associated pneumonia. The definition of VAP varies among the studies
VFD: Ventilation-free days, VFD means the number of days free from mechanical ventilation for the initial 28 days. If the patient expired within 28 days, VFD was counted as zero
