# Supplementary material for: The clinical practice guideline for the management of ARDS in Japan
Source: J Intensive Care. 2017 Jul 25;5:50. doi: 10.1186/s40560-017-0222-3 (PMC5526253; doi:10.1186/s40560-017-0222-3)
Supplement: Supplementary file 1 — contains Modified Preferred Reporting Items of Systematic Reviews and Meta-Analyses (PRISMA) flow-chart, risk of bias table, forest plots, evidence profiles, and evidence to decision table for each CQ according to the GRADE system. (PDF 32153 kb) [file 40560_2017_222_MOESM1_ESM.pdf]

CQ01. Study flow diagram

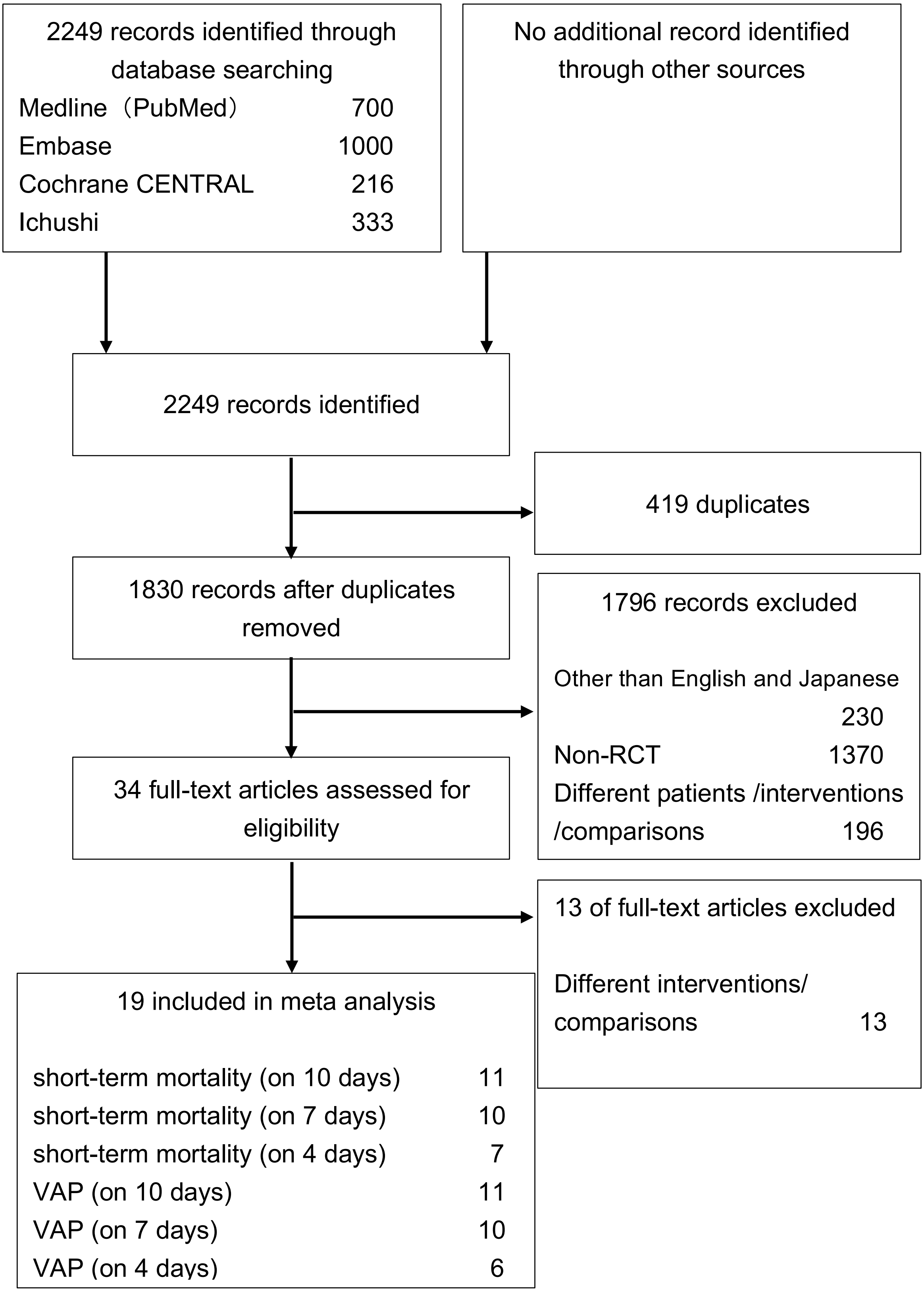

CQ01
Risk of bias Table, Mortality

Outcome		Short term mortality		risk of bias		serious (−1)			
番号	著者名 発表年（Forest plot表示）	risk of bias評価							
		ランダム割付順番の生成 random sequence generation	割り付けの隠蔽化 allocation concealment	ブラインド blinding		不完全なアウトカムデータ incomplete outcome data	選択されたアウトカムの報告 selective outcome reporting	その他のバイアス Other sources of bias	研究内でのバイアスのリスク Risk of bias within a study
				研究参加者と治療提供者 participants and personnel	アウトカム評価者 outcome assessors				
1	Braquist 2006	Low risk	Low risk	Unclear risk	Low risk	Low risk	Unclear risk	Unclear risk	Low risk
2	Blot 2008	Low risk	Low risk	High risk	Low risk	Low risk	Unclear risk	Low risk	Unclear risk
3	Bosel 2013	Low risk	Low risk	High risk	Low risk	Low risk	Low risk	High risk	High risk
4	Bouderka 2004	Low risk	Unclear risk	Unclear risk	Low risk	Low risk	Unclear risk	Unclear risk	Unclear risk
5	Diaz-Prieto 2014	Low risk	Low risk	Unclear risk	Low risk	Low risk	Low risk	Low risk	Low risk
6	Dunham 2014	Unclear risk	Unclear risk	Unclear risk	Low risk	High risk	Unclear risk	High risk	High risk
7	Fayed 2013	Unclear risk	Unclear risk	Unclear risk	Low risk	Unclear risk	Unclear risk	High risk	High risk
8	Koch 2012	Low risk	Low risk	Unclear risk	Low risk	Low risk	High risk	Low risk	Low risk
9	Mohamed 2014	Unclear risk	Unclear risk	High risk	Low risk	Low risk	Unclear risk	Unclear risk	Unclear risk
10	Rodriguez 1990	High risk	High risk	Unclear risk	Low risk	Unclear risk	Unclear risk	Unclear risk	High risk
11	Rumbak 2004	Unclear risk	Low risk	Unclear risk	Low risk	Low risk	Unclear risk	Unclear risk	Unclear risk
12	Saffle 2002	Low risk	Low risk	Unclear risk	Low risk	Low risk	Unclear risk	Unclear risk	Low risk
14	Sugerman 1997	Low risk	Low risk	Unclear risk	Low risk	High risk	Unclear risk	High risk	High risk
15	Terragni 2010	Low risk	Low risk	Unclear risk	Low risk	Low risk	High risk	Low risk	Low risk
16	Trouillet 2011	Low risk	Low risk	Unclear risk	Low risk	Low risk	Low risk	Low risk	Low risk
17	Young 2013	Low risk	Low risk	High risk	Low risk	Low risk	Low risk	Low risk	Low risk
18	Zheng 2012	Low risk	Low risk	Unclear risk	Low risk	Low risk	Unclear risk	Low risk	Low risk

CQ01-03
Risk of bias table, VAP

Outcome		VAP		risk of bias		serious (−1)			
番号	著者名 発表年（Forest plot表示）	risk of bias評価							
		ランダム割付順番の生成 random sequence generation	割り付けの隠蔽化 allocation concealment	ブラインド blinding		不完全なアウトカムデータ incomplete outcome data	選択されたアウトカムの報告 selective outcome reporting	その他のバイアス Other sources of bias	研究内でのバイアスのリスク Risk of bias within a study
				研究参加者と治療提供者 participants and personnel	アウトカム評価者 outcome assessors				
1	Braquist 2006	Low risk	Low risk	Unclear risk	Unclear risk	Low risk	Unclear risk	Unclear risk	Unclear risk
2	Blot 2008	Low risk	Low risk	High risk	Low risk	Low risk	Unclear risk	Low risk	Unclear risk
3	Bouderka 2004	Low risk	Unclear risk	Unclear risk	Unclear risk	Low risk	Unclear risk	Unclear risk	Unclear risk
4	Bylappa 2011	Unclear risk	Unclear risk	Unclear risk	Unclear risk	Low risk	Unclear risk	Unclear risk	Unclear risk
5	Diaz-Prieto 2014	Low risk	Low risk	Unclear risk	Unclear risk	Low risk	Low risk	Low risk	Low risk
6	Dunham 1984	High risk	High risk	Unclear risk	Unclear risk	Low risk	Unclear risk	Unclear risk	High risk
7	Dunham 2014	Unclear risk	Unclear risk	Unclear risk	Unclear risk	High risk	Unclear risk	High risk	High risk
8	Fayed 2013	Unclear risk	Unclear risk	Unclear risk	Unclear risk	Low risk	Unclear risk	High risk	High risk
9	Koch 2012	Low risk	Low risk	Unclear risk	Low risk	Low risk	High risk	Low risk	Unclear risk
10	Mohamed 2014	Unclear risk	Unclear risk	High risk	High risk	Low risk	Unclear risk	Unclear risk	High risk
11	Rodriguez 1990	High risk	High risk	Unclear risk	Unclear risk	Unclear risk	Unclear risk	Unclear risk	High risk
12	Rumbak 2004	Unclear risk	Low risk	Unclear risk	Unclear risk	Low risk	Unclear risk	Unclear risk	Unclear risk
13	Saffle 2002	Low risk	Low risk	Unclear risk	Unclear risk	Low risk	Unclear risk	Unclear risk	Unclear risk
14	Sugerman 1997	Low risk	Low risk	Unclear risk	Unclear risk	High risk	Unclear risk	High risk	High risk
15	Terragni 2010	Low risk	Low risk	Unclear risk	Low risk	Low risk	High risk	Low risk	Low risk
16	Trouillet 2011	Low risk	Low risk	Unclear risk	Low risk	Low risk	Low risk	Low risk	Low risk
17	Zheng 2012	Low risk	Low risk	Unclear risk	Low risk	Low risk	Unclear risk	Low risk	Low risk

CQ01. Risk of bias summary, Risk of bias graph

Short term mortality (4 days)

	Random sequence generation (selection bias)	Allocation concealment (selection bias)	Blinding of participants and personnel (performance bias)	Blinding of outcome assessment (detection bias)	Incomplete outcome data (attrition bias)	Selective reporting (reporting bias)	Other bias
Blot 2008	+	+	−	+	+	?	+
Bösel 2013	+	+	−	+	+	+	−
Koch 2012	+	+	?	+	+	−	+
Rumbak 2004	?	+	?	+	+	?	?
Saffle 2002	+	+	?	+	+	?	?
Young 2013	+	+	−	+	+	+	+
Zheng 2012	+	+	?	+	+	?	+

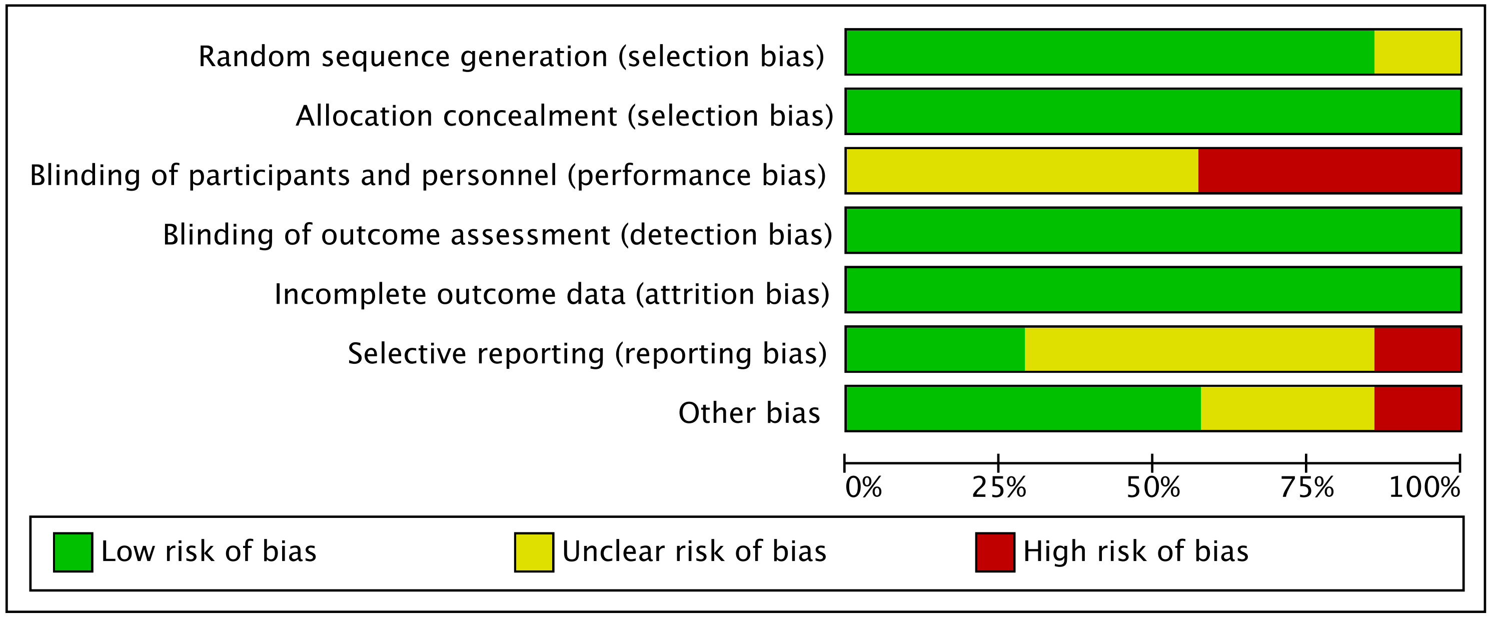

CQ01. Risk of bias summary, Risk of bias graph

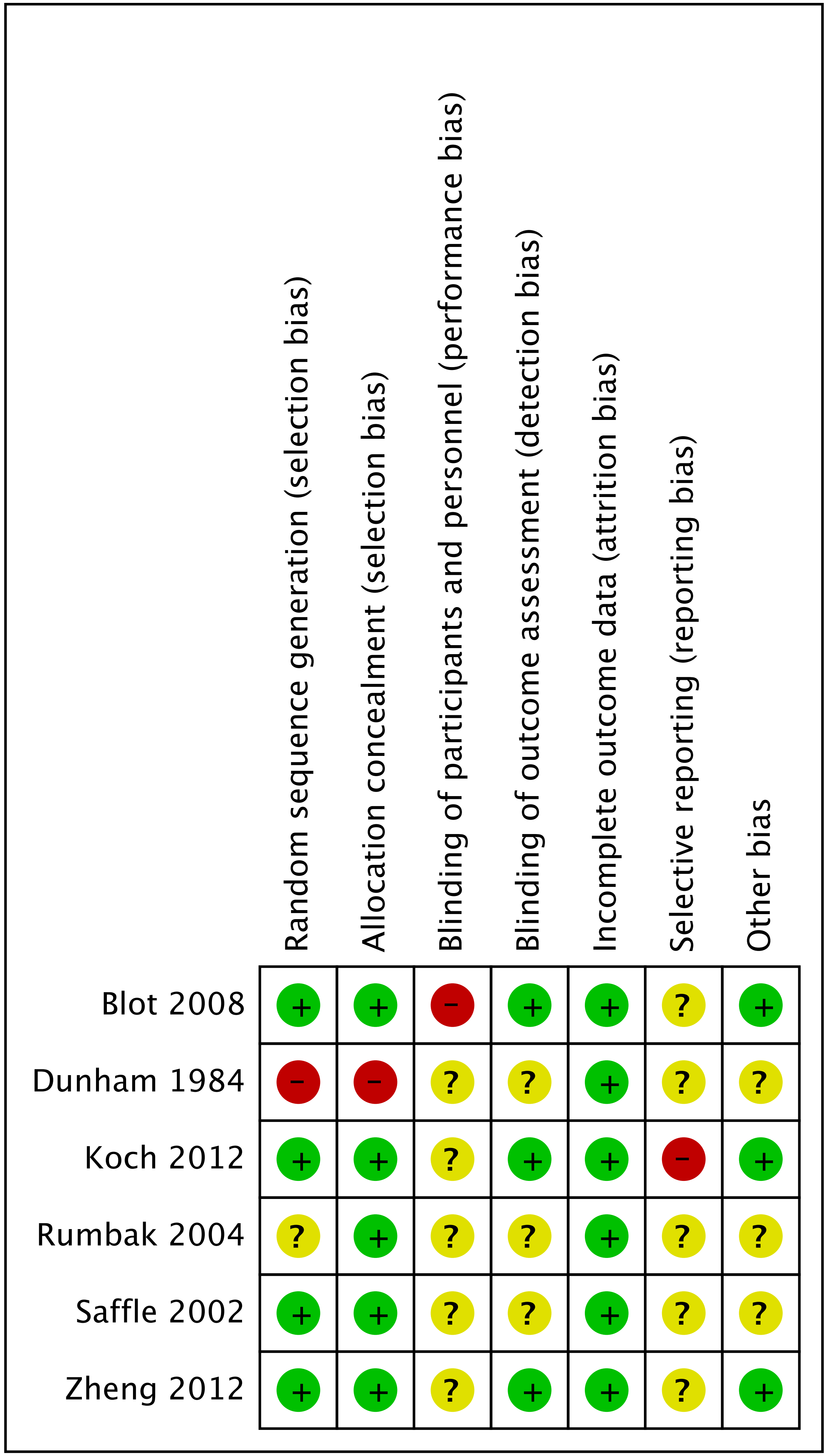

VAP (4 days)

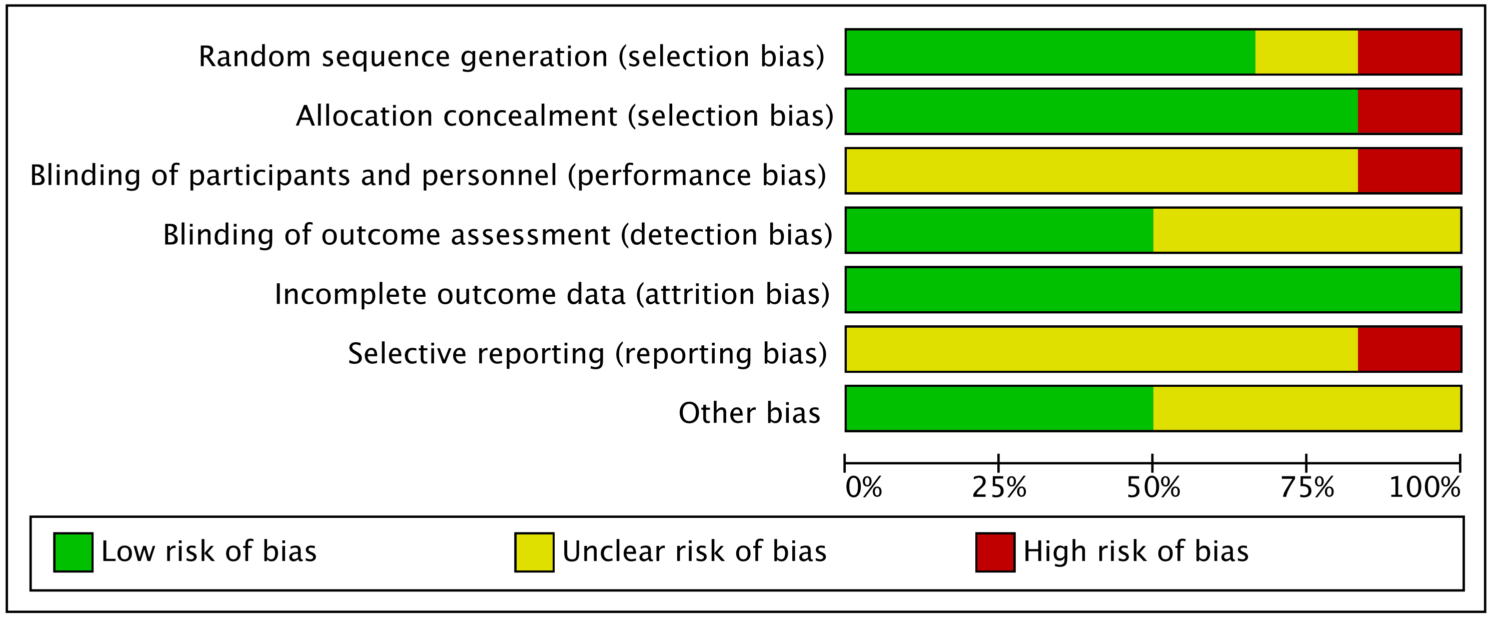

CQ01. Risk of bias summary, Risk of bias graph

Short term mortality (7 days)

	Random sequence generation (selection bias)	Allocation concealment (selection bias)	Blinding of participants and personnel (performance bias)	Blinding of outcome assessment (detection bias)	Incomplete outcome data (attrition bias)	Selective reporting (reporting bias)	Other bias
Bösel 2013	+	+	–	+	+	+	–
Bouderka 2004	+	?	?	+	+	?	?
Dunham 2014	?	?	?	+	–	?	–
Rodriguez 1990	–	–	?	+	?	?	?
Rumbak 2004	?	+	?	+	+	?	?
Saffle 2002	+	+	?	+	+	?	?
Sugerman 1997	+	+	?	+	–	?	–
Trouillet 2011	+	+	?	+	+	+	+
Young 2013	+	+	–	+	+	+	+
Zheng 2012	+	+	?	+	+	?	+

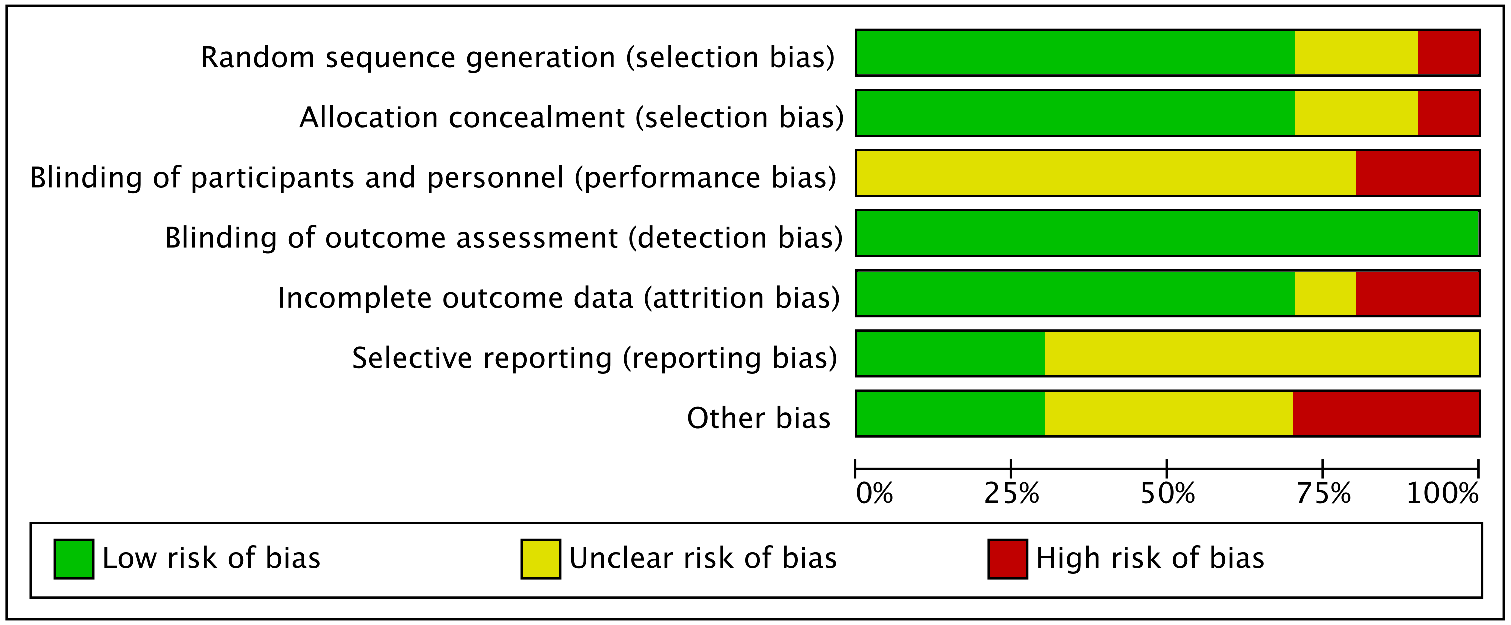

CQ01. Risk of bias summary, Risk of bias graph

VAP（7 days）

	Random sequence generation (selection bias)	Allocation concealment (selection bias)	Blinding of participants and personnel (performance bias)	Blinding of outcome assessment (detection bias)	Incomplete outcome data (attrition bias)	Selective reporting (reporting bias)	Other bias
Bouderka 2004	+	?	?	?	+	?	?
Bylappa 2011	?	?	?	?	+	?	?
Dunham 1984	−	−	?	?	+	?	?
Dunham 2014	?	?	?	?	−	?	−
Rodriguez 1990	−	−	?	?	?	?	?
Rumbak 2004	?	+	?	?	+	?	?
Saffle 2002	+	+	?	?	+	?	?
Sugerman 1997	+	+	?	?	−	?	−
Trouillet 2011	+	+	?	+	+	+	+
Zheng 2012	+	+	?	+	+	?	+

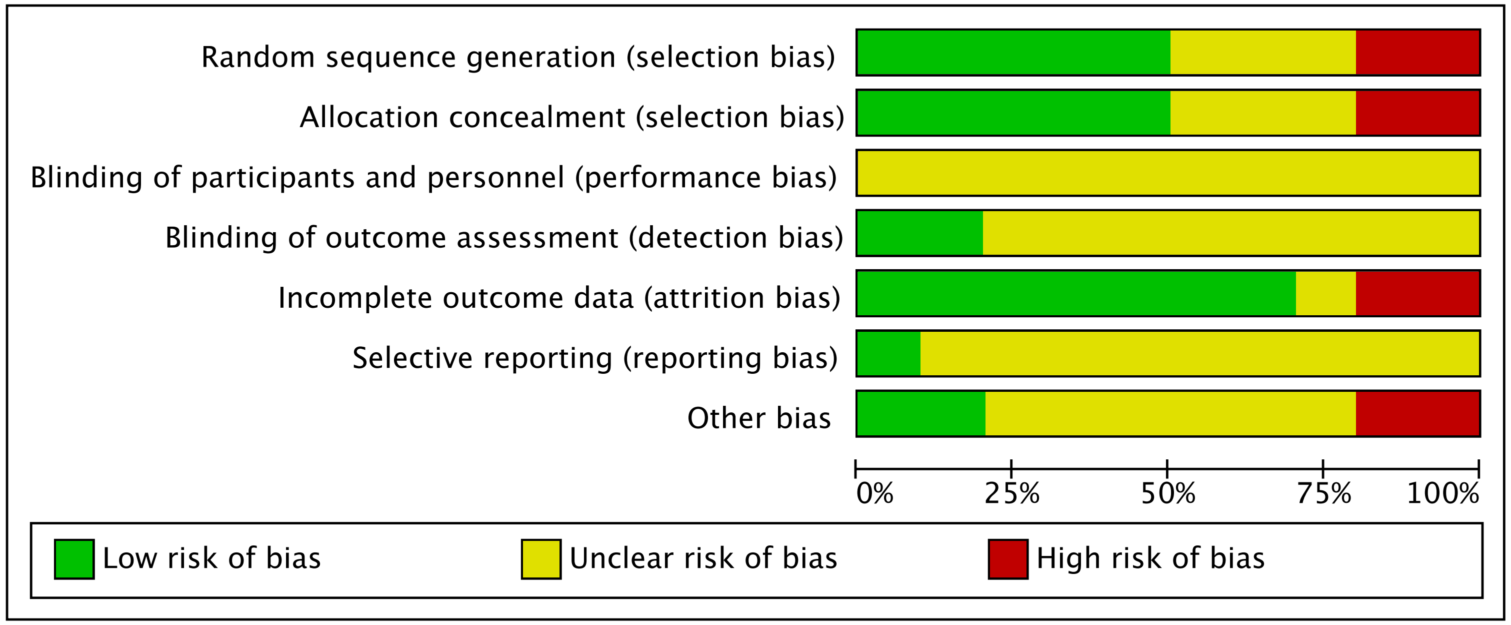

CQ01. Risk of bias summary, Risk of bias graph

Short term mortality (10 days)

	Random sequence generation (selection bias)	Allocation concealment (selection bias)	Blinding of participants and personnel (performance bias)	Blinding of outcome assessment (detection bias)	Incomplete outcome data (attrition bias)	Selective reporting (reporting bias)	Other bias
Barquist 2006	+	+	?	+	+	?	?
Diaz–Prieto 2014	+	+	?	+	+	+	+
Dunham 2014	?	?	?	+	−	?	−
Mohamed 2014	?	?	−	+	+	?	?
Rumbak 2004	?	+	?	+	+	?	?
Saffle 2002	+	+	?	+	+	?	?
Sugerman 1997	+	+	?	+	−	?	−
Terragni 2010	+	+	?	+	+	−	+
Trouillet 2011	+	+	?	+	+	+	+
Young 2013	+	+	−	+	+	+	+
Zheng 2012	+	+	?	+	+	?	+

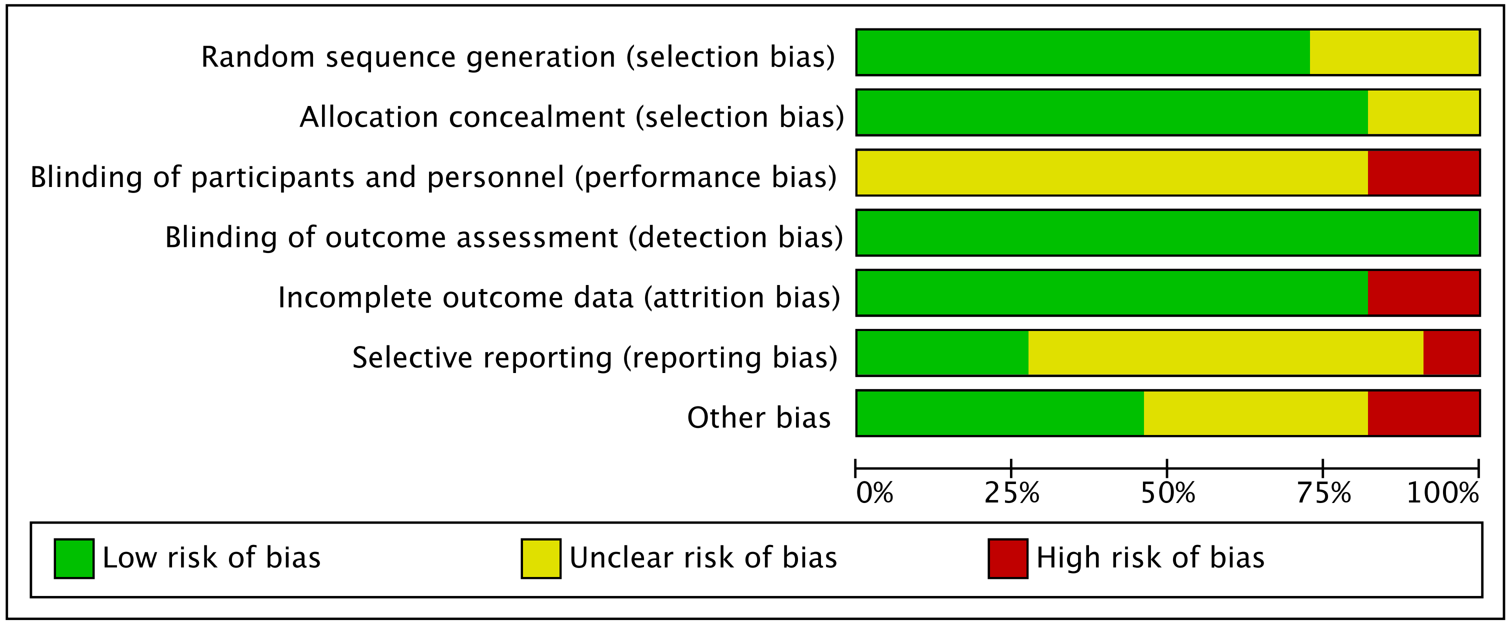

CQ01. Risk of bias summary, Risk of bias graph

VAP （10 days）

	Random sequence generation (selection bias)	Allocation concealment (selection bias)	Blinding of participants and personnel (performance bias)	Blinding of outcome assessment (detection bias)	Incomplete outcome data (attrition bias)	Selective reporting (reporting bias)	Other bias
Barquist 2006	+	+	?	?	+	?	?
Diaz–Prieto 2014	+	+	?	?	+	+	+
Dunham 1984	–	–	?	?	+	?	?
Dunham 2014	?	?	?	?	–	?	–
Mohamed 2014	?	?	–	–	+	?	?
Rumbak 2004	?	+	?	?	+	?	?
Saffle 2002	+	+	?	?	+	?	?
Sugerman 1997	+	+	?	?	–	?	–
Terragni 2010	+	+	?	+	+	–	+
Trouillet 2011	+	+	?	+	+	+	+
Zheng 2012	+	+	?	+	+	?	+

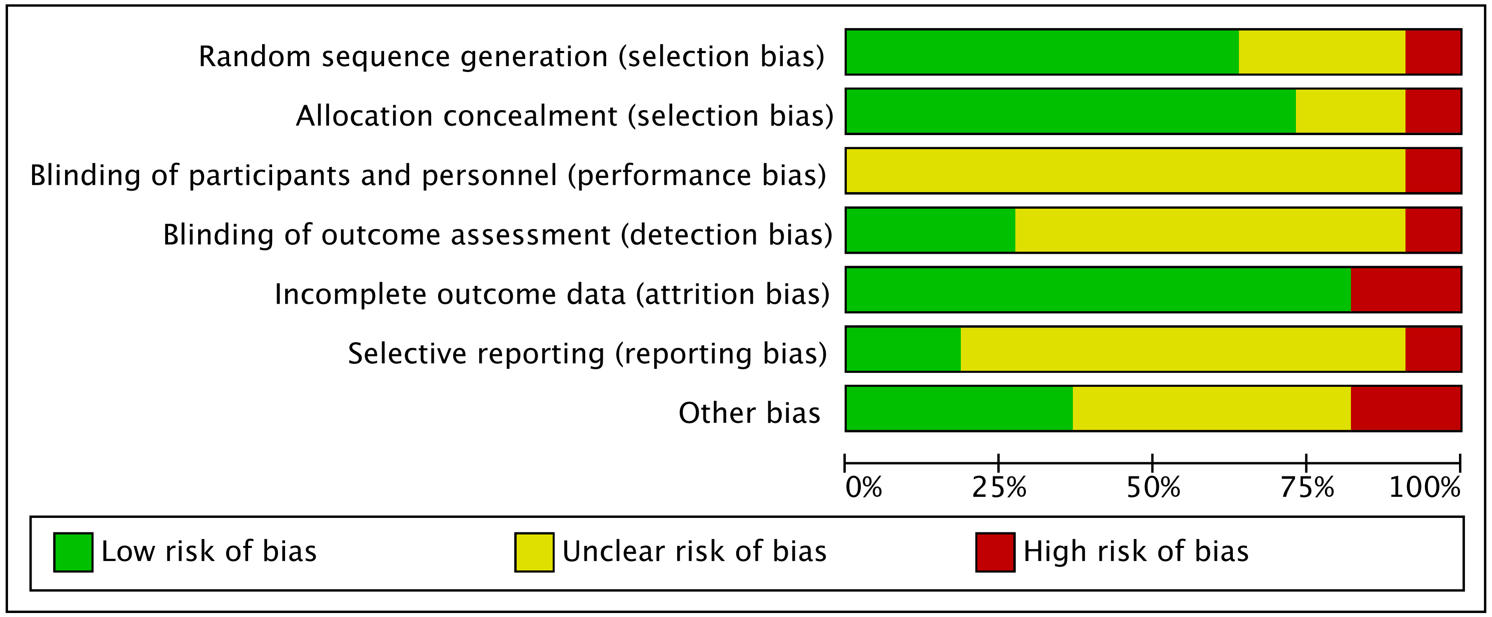

Short term mortality (4 days)

Study or Subgroup	Early Events	Early Total	Late Events	Late Total	Weight	Risk Ratio M-H, Random, 95% CI
Blot 2008	12	61	15	62	14.6%	0.81 [0.42, 1.59]
Bösel 2013	3	30	14	30	7.4%	0.21 [0.07, 0.67]
Koch 2012	10	50	11	50	12.7%	0.91 [0.42, 1.95]
Rumbak 2004	19	60	37	60	21.2%	0.51 [0.34, 0.78]
Saffle 2002	4	21	6	23	7.6%	0.73 [0.24, 2.23]
Young 2013	139	451	141	448	27.6%	0.98 [0.81, 1.19]
Zheng 2012	8	58	6	61	9.0%	1.40 [0.52, 3.80]
**Total (95% CI)**		**731**		**734**	**100.0%**	**0.74 [0.52, 1.06]**
Total events	195		230			

Heterogeneity: $Tau^2 = 0.11$; $Chi^2 = 14.23$, df = 6 (P = 0.03); $I^2 = 58\%$

Test for overall effect: Z = 1.63 (P = 0.10)

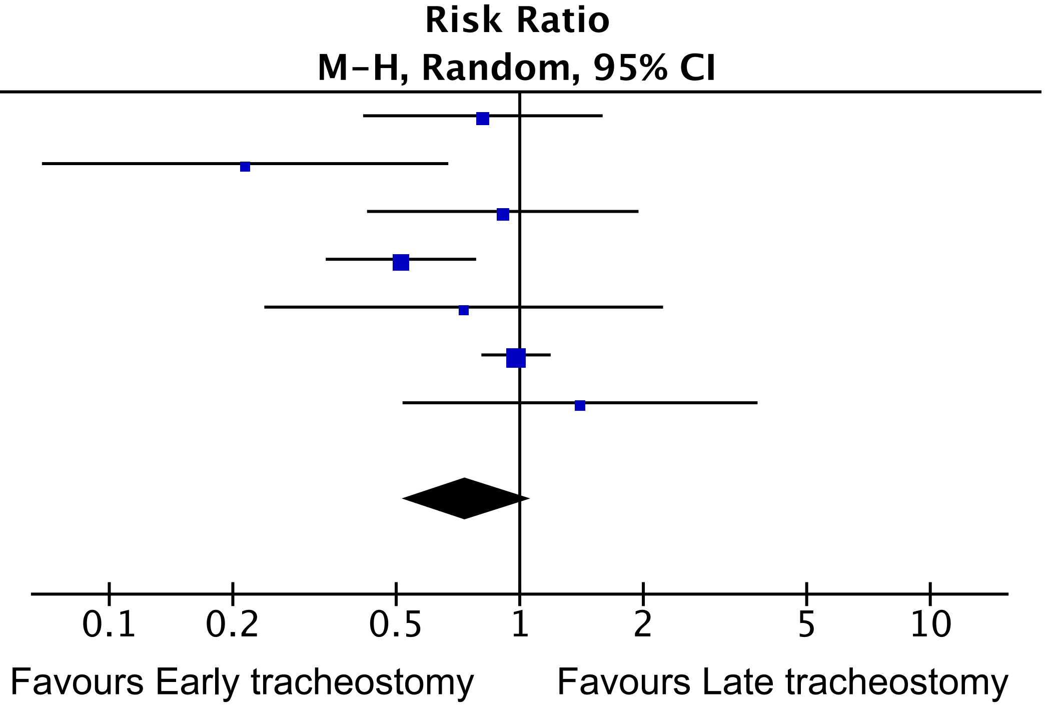

VAP (4 days)

Study or Subgroup	Early Events	Early Total	Late Events	Late Total	Weight	Risk Ratio M-H, Random, 95% CI
Blot 2008	30	61	31	62	18.7%	0.98 [0.69, 1.40]
Dunham 1984	20	34	20	40	17.7%	1.18 [0.77, 1.79]
Koch 2012	19	50	32	50	17.8%	0.59 [0.39, 0.90]
Rumbak 2004	3	60	15	60	7.6%	0.20 [0.06, 0.66]
Saffle 2002	21	21	22	23	21.4%	1.04 [0.92, 1.18]
Zheng 2012	17	58	30	61	16.8%	0.60 [0.37, 0.96]
**Total (95% CI)**		**284**		**296**	**100.0%**	**0.76 [0.51, 1.15]**
Total events	110		150			

Heterogeneity: $Tau^2 = 0.20$; $Chi^2 = 36.41$, df = 5 (P < 0.00001); $I^2 = 86\%$

Test for overall effect: Z = 1.29 (P = 0.20)

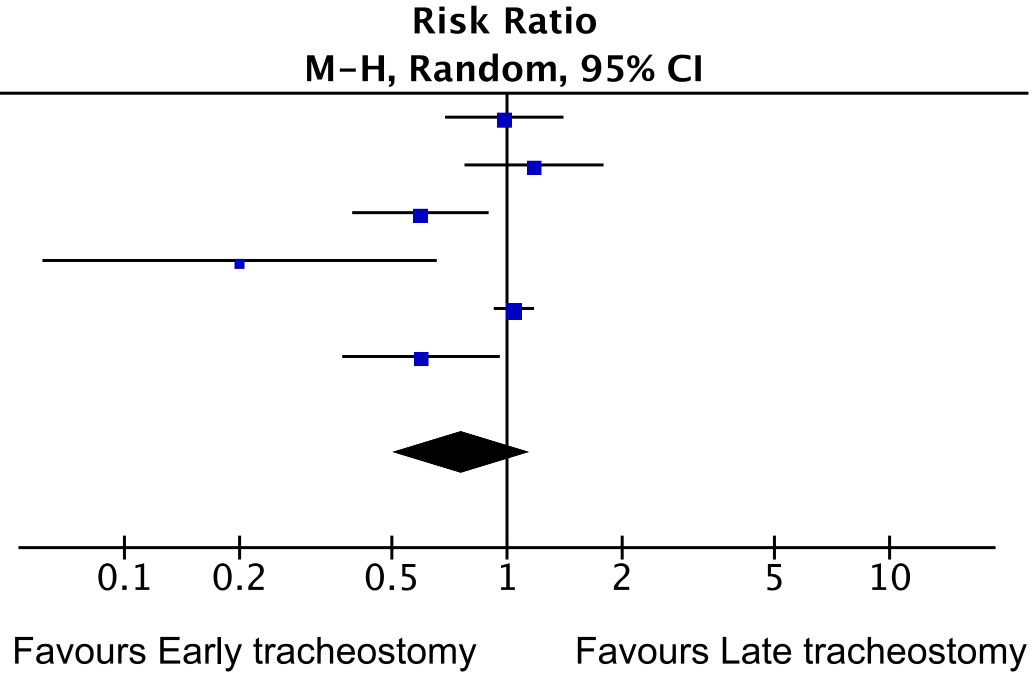

Short term mortality (7 days)

Study or Subgroup	Early Events	Early Total	Late Events	Late Total	Weight	Risk Ratio M-H, Random, 95% CI
Bouderka 2004	12	31	7	31	9.5%	1.71 [0.78, 3.77]
Bösel 2013	3	30	14	30	5.8%	0.21 [0.07, 0.67]
Dunham 2014	0	15	0	9		Not estimable
Rodriguez 1990	9	51	13	55	9.9%	0.75 [0.35, 1.60]
Rumbak 2004	19	60	37	60	16.5%	0.51 [0.34, 0.78]
Saffle 2002	4	21	6	23	5.9%	0.73 [0.24, 2.23]
Sugerman 1997	13	53	11	59	10.7%	1.32 [0.65, 2.68]
Trouillet 2011	17	109	23	107	13.3%	0.73 [0.41, 1.28]
Young 2013	139	451	141	448	21.5%	0.98 [0.81, 1.19]
Zheng 2012	8	58	6	61	7.0%	1.40 [0.52, 3.80]
**Total (95% CI)**		**879**		**883**	**100.0%**	**0.83 [0.60, 1.13]**
Total events	224		258			

Heterogeneity: $Tau^2 = 0.11$; $Chi^2 = 18.88$, df = 8 (P = 0.02); $I^2 = 58\%$
Test for overall effect: Z = 1.18 (P = 0.24)

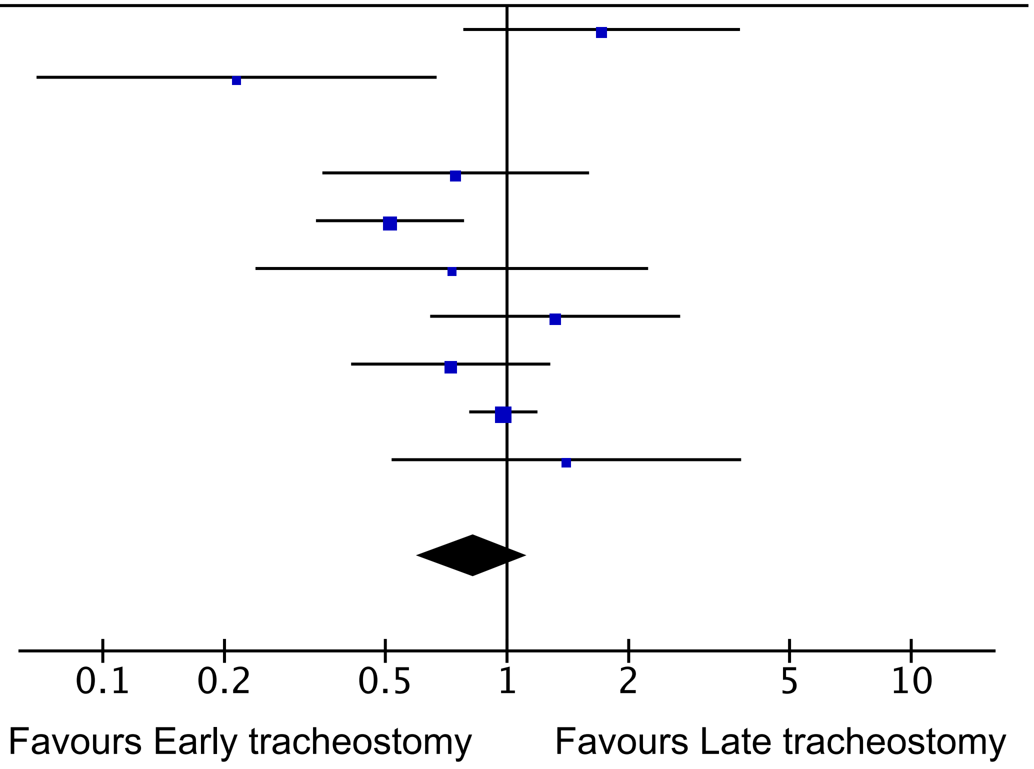

VAP（7 days)

Study or Subgroup	Early Events	Early Total	Late Events	Late Total	Weight	Risk Ratio M-H, Random, 95% CI
Bouderka 2004	18	31	19	31	10.7%	0.95 [0.63, 1.43]
Bylappa 2011	3	22	13	22	2.8%	0.23 [0.08, 0.70]
Dunham 1984	20	34	20	40	10.5%	1.18 [0.77, 1.79]
Dunham 2014	7	15	4	9	3.9%	1.05 [0.42, 2.61]
Rodriguez 1990	40	51	53	55	17.1%	0.81 [0.70, 0.95]
Rumbak 2004	3	60	15	60	2.5%	0.20 [0.06, 0.66]
Saffle 2002	21	21	22	23	17.7%	1.04 [0.92, 1.18]
Sugerman 1997	26	53	32	56	11.9%	0.86 [0.60, 1.23]
Trouillet 2011	50	109	47	107	13.5%	1.04 [0.78, 1.40]
Zheng 2012	17	58	30	61	9.3%	0.60 [0.37, 0.96]
**Total (95% CI)**		**454**		**464**	**100.0%**	**0.85 [0.70, 1.05]**
Total events	205		255			

Heterogeneity: $Tau^2 = 0.06$; $Chi^2 = 31.55$, df = 9 (P = 0.0002); $I^2 = 71\%$
Test for overall effect: Z = 1.52 (P = 0.13)

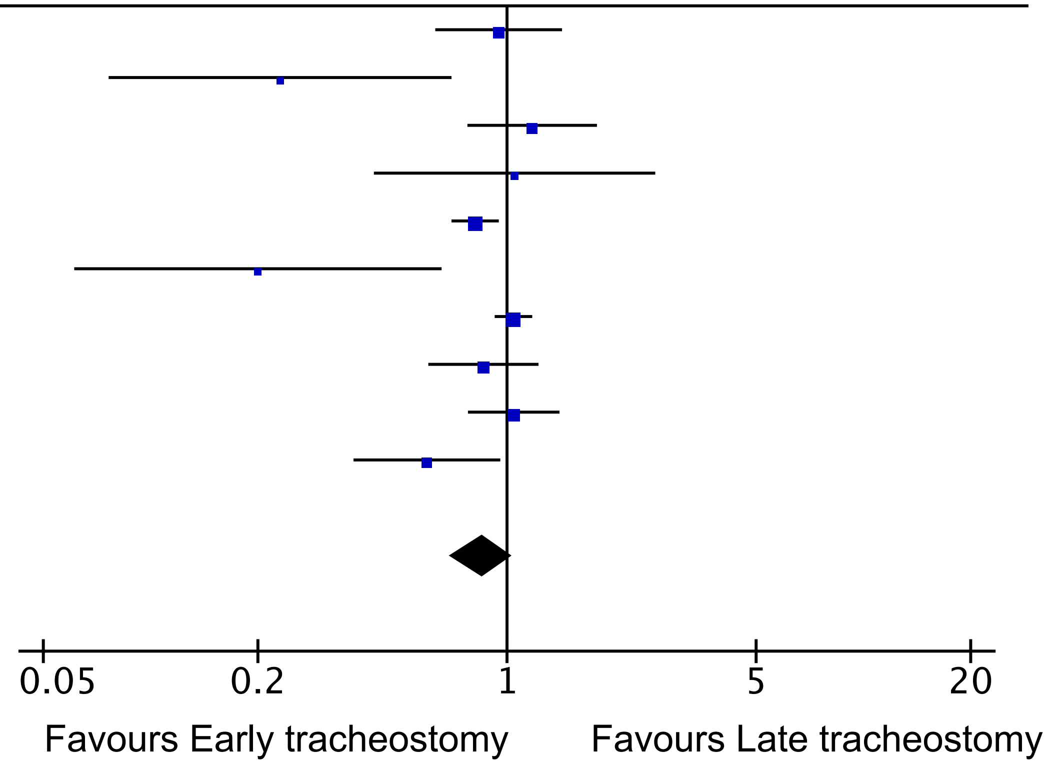

Short term mortality (10 days)

Study or Subgroup	Early Events	Early Total	Late Events	Late Total	Weight	Risk Ratio M-H, Random, 95% CI
Barquist 2006	2	29	5	31	1.1%	0.43 [0.09, 2.03]
Diaz-Prieto 2014	42	245	47	244	14.3%	0.89 [0.61, 1.30]
Dunham 2014	0	15	0	9		Not estimable
Mohamed 2014	8	20	8	20	4.4%	1.00 [0.47, 2.14]
Rumbak 2004	19	60	37	60	12.0%	0.51 [0.34, 0.78]
Saffle 2002	4	21	6	23	2.1%	0.73 [0.24, 2.23]
Sugerman 1997	13	53	11	59	4.9%	1.32 [0.65, 2.68]
Terragni 2010	55	209	66	210	19.5%	0.84 [0.62, 1.13]
Trouillet 2011	17	109	23	107	7.3%	0.73 [0.41, 1.28]
Young 2013	139	451	141	448	31.7%	0.98 [0.81, 1.19]
Zheng 2012	8	58	6	61	2.6%	1.40 [0.52, 3.80]
**Total (95% CI)**		**1270**		**1272**	**100.0%**	**0.86 [0.73, 1.01]**
Total events	307		350			

Heterogeneity: $Tau^2 = 0.01$; $Chi^2 = 11.08$, df = 9 (P = 0.27); $I^2 = 19\%$
Test for overall effect: Z = 1.85 (P = 0.06)

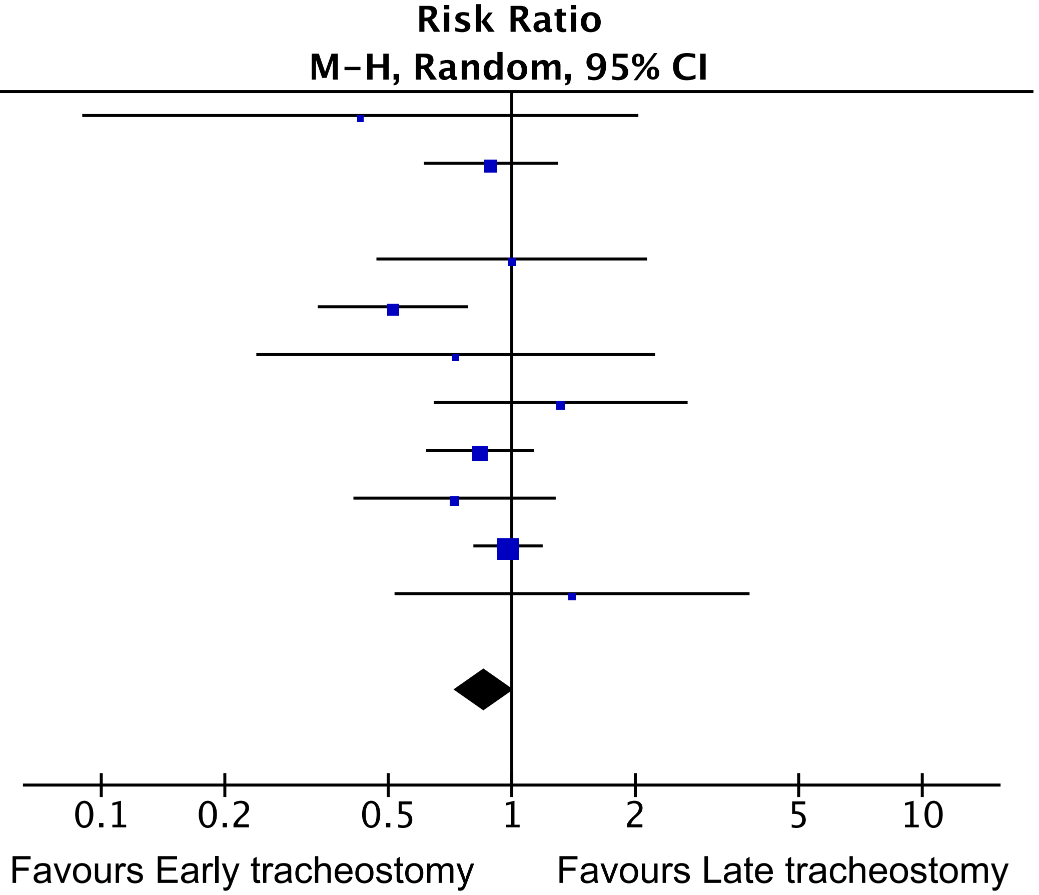

VAP（10 days)

Study or Subgroup	Early Events	Early Total	Late Events	Late Total	Weight	Risk Ratio M-H, Random, 95% CI
Barquist 2006	28	29	28	31	17.0%	1.07 [0.93, 1.22]
Diaz-Prieto 2014	33	245	23	244	7.7%	1.43 [0.86, 2.36]
Dunham 1984	20	34	20	40	9.3%	1.18 [0.77, 1.79]
Dunham 2014	7	15	4	9	3.3%	1.05 [0.42, 2.61]
Mohamed 2014	4	20	8	20	2.7%	0.50 [0.18, 1.40]
Rumbak 2004	3	60	15	60	2.1%	0.20 [0.06, 0.66]
Saffle 2002	21	21	22	23	17.3%	1.04 [0.92, 1.18]
Sugerman 1997	26	53	32	56	10.8%	0.86 [0.60, 1.23]
Terragni 2010	30	209	44	210	9.2%	0.69 [0.45, 1.05]
Trouillet 2011	50	109	47	107	12.5%	1.04 [0.78, 1.40]
Zheng 2012	17	58	30	61	8.2%	0.60 [0.37, 0.96]
**Total (95% CI)**		**853**		**861**	**100.0%**	**0.93 [0.77, 1.11]**
Total events	239		273			

Heterogeneity: $Tau^2 = 0.05$; $Chi^2 = 30.36$, df = 10 (P = 0.0007); $I^2 = 67\%$
Test for overall effect: Z = 0.84 (P = 0.40)

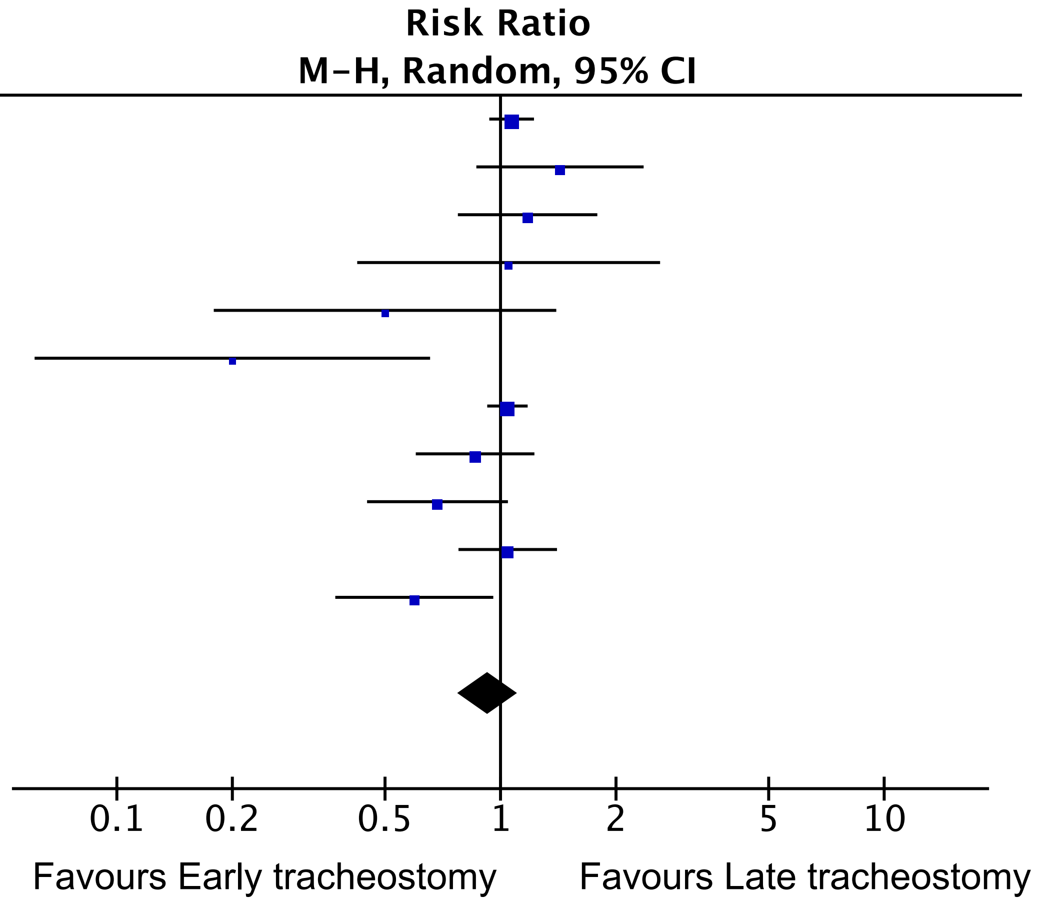

Short term mortality (7 days)

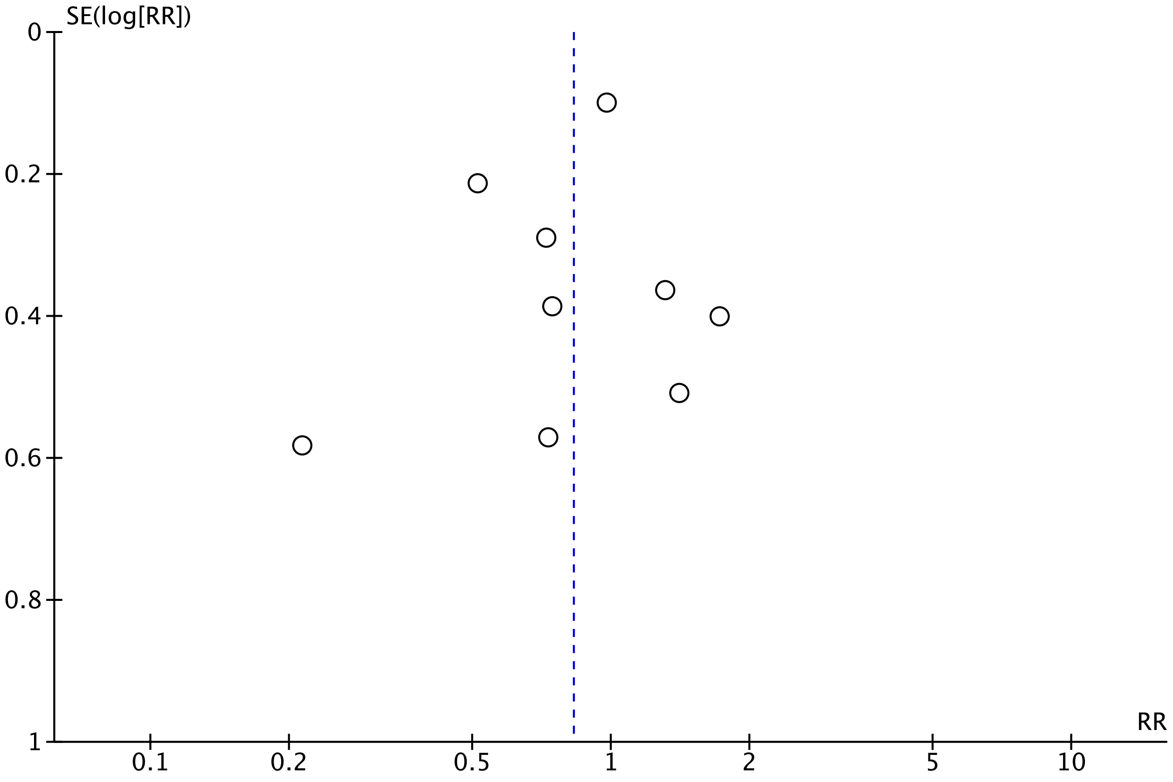

Short term mortality (10 days)

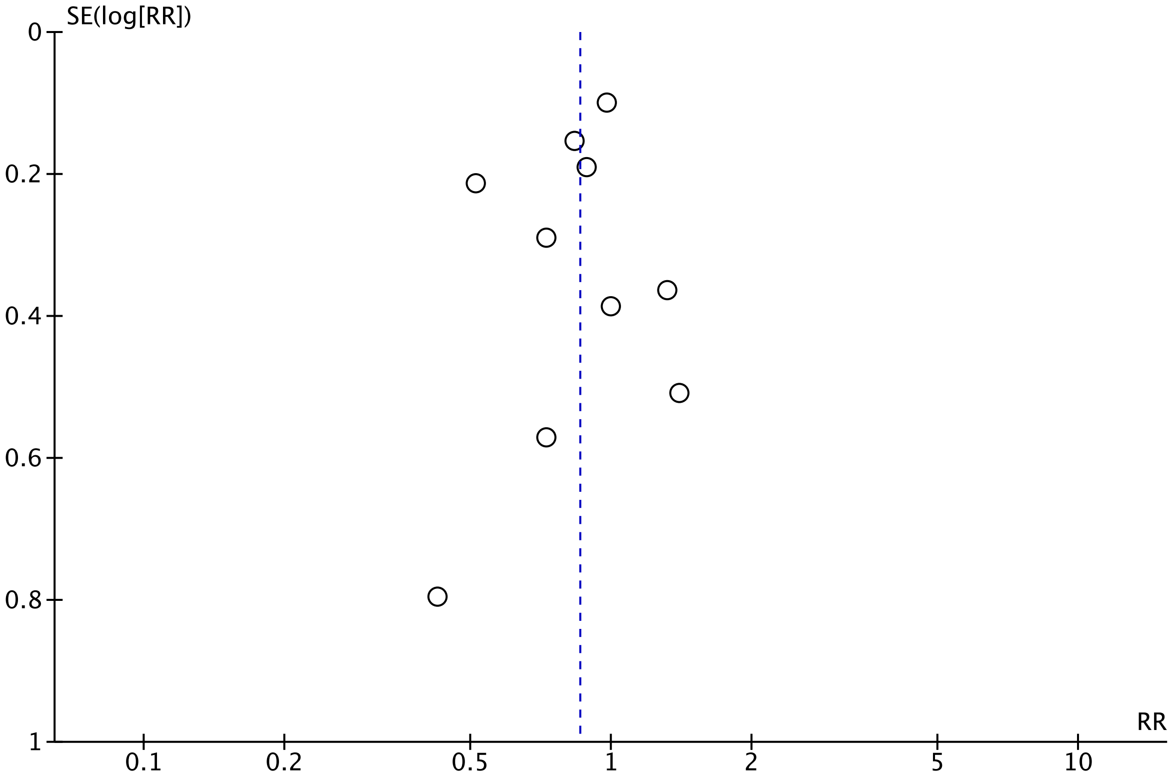

VAP（7 days)

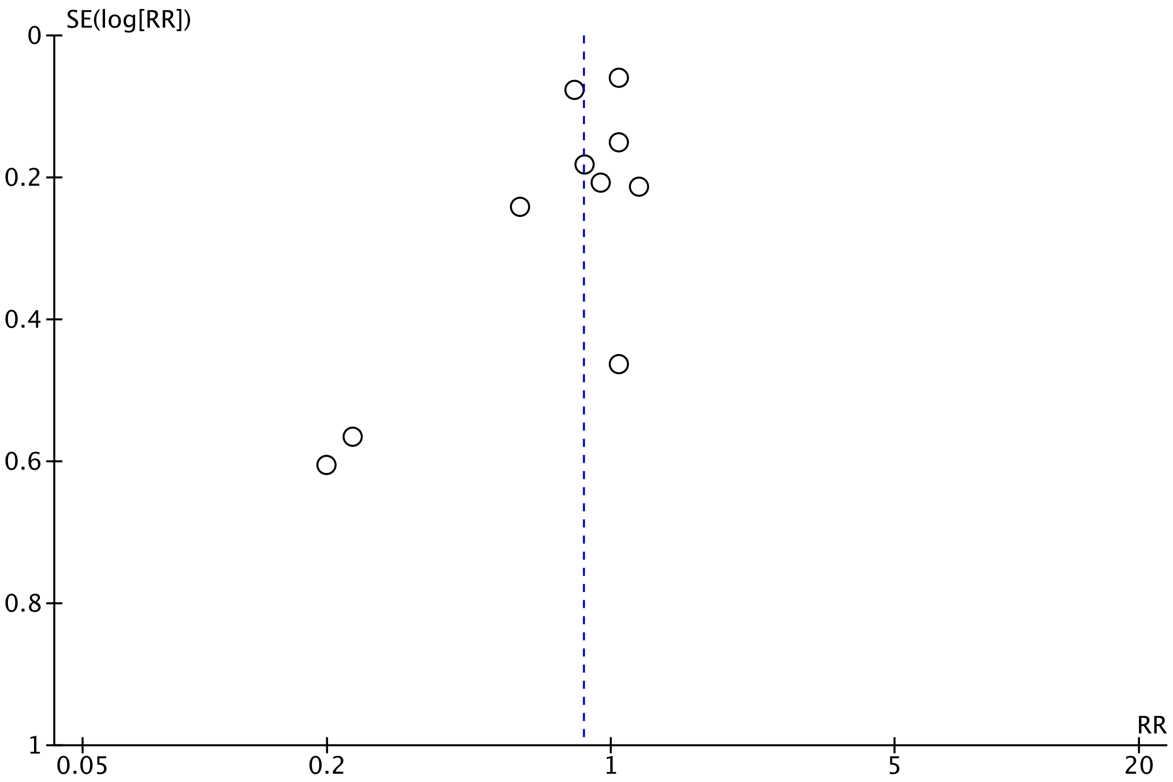

VAP（10 days)

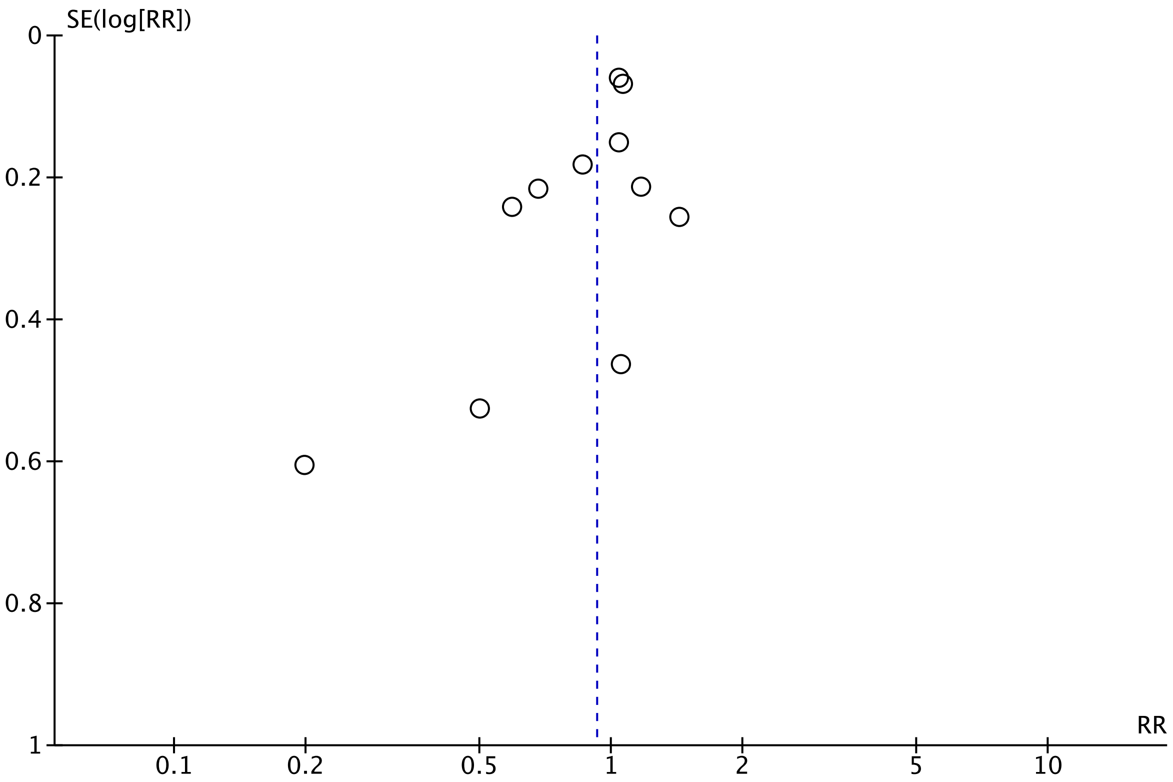

Summary of findings:

Early tracheotomy compared to late tracheotomy for ARDS

Patient or population: ARDS

Intervention: early tracheotomy

Comparison: late tracheotomy

Outcomes	Anticipated absolute effects* (95% CI)		Relative effect (95% CI)	№ of participants (studies)	Quality of the evidence (GRADE)	Comments
	Risk with late tracheotomy	Risk with early tracheotomy				
Short term mortality (on 10 days)	**Study population**		**RR 0.86** (0.73 to 1.01)	2542 (11 RCTs)	⨁⨁◯◯ LOW [1]	
	275 per 1000	**237 per 1000** (201 to 278)				
	**Low**					
	156 per 1000	**134 per 1000** (114 to 158)				
	**High**					
	308 per 1000	**265 per 1000** (225 to 311)				
Short term mortality (on 7 days)	**Study population**		**RR 0.83** (0.60 to 1.13)	1762 (10 RCTs)	⨁◯◯◯ VERY LOW [1,2]	
	292 per 1000	**243 per 1000** (175 to 330)				
	**Low**					
	156 per 1000	**129 per 1000** (94 to 176)				
	**High**					
	308 per 1000	**256 per 1000** (185 to 348)				
Short term mortality (on 4 days)	**Study population**		**RR 0.74** (0.52 to 1.06)	1465 (7 RCTs)	⨁◯◯◯ VERY LOW [1,3]	
	313 per 1000	**232 per 1000** (163 to 332)				
	**Low**					
	156 per 1000	**115 per 1000** (81 to 165)				
	**High**					
	308 per 1000	**228 per 1000** (160 to 326)				
VAP (on 10 days)	**Study population**		**RR 0.93** (0.77 to 1.11)	1714 (11 RCTs)	⨁◯◯◯ VERY LOW [1,4,5,6]	
	317 per 1000	**295 per 1000** (244 to 352)				
	**Low**					
	134 per 1000	**125 per 1000** (103 to 149)				
	**High**					
	459 per 1000	**427 per 1000** (353 to 509)				

VAP (on 7 days)	**Study population**		**RR 0.85** (0.70 to 1.05)	918 (10 RCTs)	⊕◯◯◯ VERY LOW [1,4,6,7]
	550 per 1000	**467 per 1000** (385 to 577)			
	**Low**				
	134 per 1000	**114 per 1000** (94 to 141)			
	**High**				
	459 per 1000	**390 per 1000** (321 to 482)			
VAP (on 4 days)	**Study population**		**RR 0.76** (0.51 to 1.15)	580 (6 RCTs)	⊕◯◯◯ VERY LOW [1,4,6,8]
	507 per 1000	**385 per 1000** (258 to 583)			
	**Low**				
	134 per 1000	**102 per 1000** (68 to 154)			
	**High**				
	459 per 1000	**349 per 1000** (234 to 528)			

***The risk in the intervention group** (and its 95% confidence interval) is based on the assumed risk in the comparison group and the **relative effect** of the intervention (and its 95% CI).

CI: Confidence interval; **RR:** Risk ratio;

GRADE Working Group grades of evidence
High quality: We are very confident that the true effect lies close to that of the estimate of the effect
Moderate quality: We are moderately confident in the effect estimate: The true effect is likely to be close to the estimate of the effect, but there is a possibility that it is substantially different
Low quality: Our confidence in the effect estimate is limited: The true effect may be substantially different from the estimate of the effect
Very low quality: We have very little confidence in the effect estimate: The true effect is likely to be substantially different from the estimate of effect

1. Since the upper and lower limits of the confidence interval of the effect estimate overlap the "clinical decision thresholds", the quality of evidence was downgraded by one level. In addition, the subjects are not necessarily patients with ARDS but "critically ill patients who are dependent on mechanical ventilator"
2. Since the confidence interval is partially overlapped and the heterogeneity is significant with I^2=58% and P=0.02, the quality of evidence was downgraded by one level.
3. Since the confidence interval is partially overlapped and the heterogeneity is significant with I^2=58% and P=0.03, the quality of evidence was downgraded by one level.
4. Since the serious limitations are exist, the quality of evidence was downgraded by one level.
5. Since the confidence interval is partially overlapped and the heterogeneity is significant with I^2=67% and P=0.0007, the quality of evidence was downgraded by one level.
6. Since funnel plot is asymmetry, publication bias was suspected.
7. Since the confidence interval is partially overlapped and the heterogeneity is significant with I^2=71% and P=0.0002, the quality of evidence was downgraded by one level.
8. Since the confidence interval is partially overlapped and the heterogeneity is significant with I^2=86% and P<0.000001, the quality of evidence was downgraded by one level.

CQ1:
Question: Early tracheotomy compared to late tracheotomy for ARDS

Quality assessment							Number of patients		Effect		Quality	Importance
**Number of studies**	**Study design**	**Risk of bias**	**Inconsistency**	**Indirectness**	**Imprecision**	**Other considerations**	**Early**	**Late**	**Relative (95% CI)**	**Absolute (95% CI)**		
Short-term mortality (on 10 days)												
11	Randomized trials	Not serious	Not serious	Serious	Serious [1]	None	307/1270 (24.2%)	350/1272 (27.5%)	**RR 0.86** (0.73 to 1.01)	39 fewer per 1000 (from 3 more to 74 fewer)	⊕⊕⊖⊖ LOW [1]	CRITICAL
								15.6%		22 fewer per 1000 (from 2 more to 42 fewer)		
								30.8%		43 fewer per 1000 (from 3 more to 83 fewer)		
Short-term mortality (on 7 days)												
10	Randomized trials	Not serious	Serious [2]	Serious	Serious [1]	None	224/879 (25.5%)	258/883 (29.2%)	**RR 0.83** (0.60 to 1.13)	50 fewer per 1000 (from 38 more to 117 fewer)	⊕⊖⊖⊖ VERY LOW [1,2]	CRITICAL
								15.6%		27 fewer per 1000 (from 20 more to 62 fewer)		
								30.8%		52 fewer per 1000 (from 40 more to 123 fewer)		
Short-term mortality (on 4 days)												
7	Randomized trials	Not serious	Serious [3]	Serious	Serious [1]	None	195/731 (26.7%)	230/734 (31.3%)	**RR 0.74** (0.52 to 1.06)	81 fewer per 1000 (from 19 more to 150 fewer)	⊕⊖⊖⊖ VERY LOW [1,3]	CRITICAL
								15.6%		41 fewer per 1000 (from 9 more to 75 fewer)		
								30.8%		80 fewer per 1000 (from 18 more to 148 fewer)		
VAP (on 10 days)												
11	Randomized trials	Serious [4]	Serious [5]	Serious	Serious [1]	Publication bias suspected [6]	239/853 (28.0%)	273/861 (31.7%)	**RR 0.93** (0.77 to 1.11)	22 fewer per 1000 (from 35 more to 73 fewer)	⊕⊖⊖⊖ VERY LOW [1,4,5,6]	CRITICAL
								13.4%		9 fewer per 1000 (from 15 more to 31 fewer)		

								45.9%		32 fewer per 1000<br>(from 50 more to 106 fewer)		
VAP (on 7 days)												
10	Randomized trials	Serious [4]	Serious [7]	Serious	Serious [1]	Publication bias suspected [6]	205/454<br>(45.2%)	255/464 (55.0%)	**RR 0.85**<br>(0.70 to 1.05)	82 fewer per 1000<br>(from 27 more to 165 fewer)	⊕⊖⊖⊖<br>VERY LOW [1 4 6 7]	CRITICAL
								13.4%		20 fewer per 1000<br>(from 7 more to 40 fewer)		
								45.9%		69 fewer per 1000<br>(from 23 more to 138 fewer)		
VAP (on 4 days)												
6	Randomized trials	Serious [4]	Serious [8]	Serious	Serious [1]	None	110/284<br>(38.7%)	150/296 (50.7%)	**RR 0.76**<br>(0.51 to 1.15)	122 fewer per 1000<br>(from 76 more to 248 fewer)	⊕⊖⊖⊖<br>VERY LOW [1 4 6 8]	CRITICAL
								13.4%		32 fewer per 1000<br>(from 20 more to 66 fewer)		
								45.9%		110 fewer per 1000<br>(from 69 more to 225 fewer)		

CI – confidence interval, RR – relative risk

1. Since the upper and lower limits of the confidence interval of the effect estimate overlap the “clinical decision thresholds”, the quality of evidence was downgraded by one level. In addition, the subjects are not necessarily patients with ARDS but “critically ill patients who are dependent on mechanical ventilator”
2. Since the confidence interval is partially overlapped and the heterogeneity is significant with I^2=58% and P=0.02, the quality of evidence was downgraded by one level.
3. Since the confidence interval is partially overlapped and the heterogeneity is significant with I^2=58% and P=0.03, the quality of evidence was downgraded by one level.
4. Since the serious limitations are exist, the quality of evidence was downgraded by one level.
5. Since the confidence interval is partially overlapped and the heterogeneity is significant with I^2=67% and P=0.0007, the quality of evidence was downgraded by one level.
6. Since funnel plot is asymmetry, publication bias was suspected.
7. Since the confidence interval is partially overlapped and the heterogeneity is significant with I^2=71% and P=0.0002, the quality of evidence was downgraded by one level.
8. Since the confidence interval is partially overlapped and the heterogeneity is significant with I^2=86% and P<0.000001, the quality of evidence was downgraded by one level.

Evidence-to-Dicision table

CQ1：Should early tracheostomy be performed in adult patients with ARDS?

POPULATION：ADULT PATIENTS ANTICIPATED TO REQUIRE LONG-TERM MECHANICAL VENTILATION

INTERVENTION：EARLY TRACHEOSTOMY

	CRITERIA	CRITERIA	CRITERIA	ADDITIONAL CONSIDERATIONS
PROBLEM	**Is the problem a priority?**	◯No<br>◯Probably no<br>⬤Probably yes<br>◯Yes<br>------------------<br>◯Varies<br>◯Don't know	In patients requiring mechanical ventilation, airway management by tracheal intubation is generally used. However, airway management by tracheostomy has some advantages including potential reduction of the need for sedative-analgesic agents and avoidance of vocal cord injury by the endotracheal tube[1, 2]. However, tracheostomy is invasive in itself and is also associated with complications such as bleeding or tracheal stenosis[3, 4]. For these reasons, tracheostomy is generally performed when the trachea has been intubated for at least 14 days and prolonged mechanical ventilation is anticipated. However, it has been suggested that early tracheostomy could shorten the duration of mechanical ventilation, reduce ventilator associated pneumonia (VAP), and improve outcome by maximizing these advantages. Therefore, it is important to examine the potential for early tracheostomy to improve outcomes in patients with ARDS anticipating prolonged mechanical ventilation.<br>Currently, there is no study that has investigated the appropriate timing for tracheostomy exclusively in adult patients with ARDS. In this CQ, several studies including patients anticipated to require long-term mechanical ventilation, including patients with ARDS, were examined in order to determine whether early tracheostomy is beneficial in improving patient outcomes.	
DESIRABLE AND UNDESIRABLE EFFECTS	**What is the overall certainty of the evidence of effects?**	◯Very low<br>⬤Low<br>◯Moderate<br>◯High<br>------------------<br>◯No included studies	**The relative importance or values of the main outcomes of interest:** (see table below)	
	**Is there important uncertainty about or variability in how much people value the main outcomes?**	◯Important uncertainty or variability<br>◯Possibly important uncertainty or variability<br>◯Possibly no important uncertainty or variability<br>⬤No important uncertainty or variability<br>------------------<br>◯No known undesirable outcomes		We collected and analyzed reports associated with tracheostomy within 4, 7, and 10 days after the initiation of mechanical ventilation, and summarized the results at 4, 7, and 10 days respectively.
	**How substantial are the desirable anticipated effects?**	⬤Trivial<br>◯Small<br>◯Moderate<br>◯Large<br>------------------<br>◯Varies<br>◯Don't know		Many patients with ARDS are included in the group of "patients who are anticipated to require long-term mechanical ventilation", the subjects in this CQ. However, patients with different

The relative importance or values of the main outcomes of interest:

Outcome	Relative importance	Certainty of the evidence (GRADE)
Mortality(short-term) (Note 1<br>(on 10 days)	CRITICAL	⊕⊕⊖⊖<br>LOW
Mortality(short-term) (Note 1<br>(on 7 days)	CRITICAL	⊕⊖⊖⊖<br>VERY LOW
Mortality(short-term) (Note 1<br>(on 4 days)	CRITICAL	⊕⊖⊖⊖<br>VERY LOW
VAP (Note 2<br>(on 10 days)	CRITICAL	⊕⊖⊖⊖<br>VERY LOW
VAP (Note 2<br>(on 7 days)	CRITICAL	⊕⊖⊖⊖<br>VERY LOW
VAP (Note 2<br>(on 4 days)	CRITICAL	⊕⊖⊖⊖<br>VERY LOW
VFD (Note 3	CRITICAL	No studies (Additional considerations

<table>
<tr>
<td rowspan="2"></td>
<td>How substantial are the undesirable anticipated effects?</td>
<td>○Large
○Moderate
○Small
○Trivial

○Varies
●Don't know</td>
<td rowspan="2">Summary of findings:
<table>
<tr><th>Outcome</th><th>Late</th><th>Early</th><th>Absolute effect (95% CI)</th><th>Relative effect (RR) (95% CI)</th></tr>
<tr><td rowspan="3">Mortality (short-term) (Note 1
(on 10 days)</td><td>275 / 1000</td><td>237 / 1000 (201 to 278)</td><td>39 fewer per 1000 (from 3 more to 74 fewer)</td><td rowspan="3">RR 0.86 (0.73 to 1.01)</td></tr>
<tr><td>156 / 1000</td><td>134 / 1000 (114 to 158)</td><td>22 fewer per 1000 (from 2 more to 42 fewer)</td></tr>
<tr><td>308 / 1000</td><td>265 / 1000 (225 to 311)</td><td>43 fewer per 1000 (from 3 more to 83 fewer)</td></tr>
<tr><td rowspan="3">Mortality (short-term) (Note 1
(on 7 days)</td><td>292 / 1000</td><td>243 / 1000 (175 to 330)</td><td>50 fewer per 1000 (from 38 more to 117 fewer)</td><td rowspan="3">RR 0.83 (0.60 to 1.13)</td></tr>
<tr><td>156 / 1000</td><td>129 / 1000 (94 to 176)</td><td>27 fewer per 1000 (from 20 more to 62 fewer)</td></tr>
<tr><td>308 / 1000</td><td>256 / 1000 (185 to 348)</td><td>52 fewer per 1000 (from 40 more to 123 fewer)</td></tr>
<tr><td rowspan="3">Mortality (short-term) (Note 1
(on 4 days)</td><td>313 / 1000</td><td>232 / 1000 (163 to 332)</td><td>81 fewer per 1000 (from 19 more to 150 fewer)</td><td rowspan="3">RR 0.74 (0.52 to 1.06)</td></tr>
<tr><td>156 / 1000</td><td>115 / 1000 (81 to 165)</td><td>41 fewer per 1000 (from 9 more to 75 fewer)</td></tr>
<tr><td>308 / 1000</td><td>228 /1000 (160 to 326)</td><td>80 fewer per 1000 (from 18 more to 148 fewer)</td></tr>
<tr><td rowspan="3">VAP (Note 2
(on 10 days)</td><td>317 / 1000</td><td>295 / 1000 (244 to 352)</td><td>22 fewer per 1000 (from 35 more to 73 fewer)</td><td rowspan="3">RR 0.93 (0.77 to 1.11)</td></tr>
<tr><td>134 / 1000</td><td>125 / 1000 (103 to 149)</td><td>9 fewer per 1000 (from 15 more to 31 fewer)</td></tr>
<tr><td>459 / 1000</td><td>427 / 1000 (353 to 509)</td><td>32 fewer per 1000 (from 50 more to 106 fewer)</td></tr>
<tr><td rowspan="3">VAP (Note 2
(on 7 days)</td><td>550 / 1000</td><td>467 / 1000 (385 to 577)</td><td>82 fewer per 1000 (from 27 more to 165 fewer)</td><td rowspan="3">RR 0.85 (0.70 to 1.05)</td></tr>
<tr><td>134 / 1000</td><td>114 / 1000 (94 to 141)</td><td>20 fewer per 1000 (from 7 more to 40 fewer)</td></tr>
<tr><td>459 / 1000</td><td>390 / 1000 (321 to 482)</td><td>69 fewer per 1000 (from 23 more to 138 fewer)</td></tr>
<tr><td rowspan="3">VAP (Note 2
(on 4 days)</td><td>507 / 1000</td><td>385 / 1000 (258 to 583)</td><td>122 fewer per 1000 (from 76 more to 248 fewer)</td><td rowspan="3">RR 0.76 (0.51 to 1.15)</td></tr>
<tr><td>134 / 1000</td><td>102 / 1000 (68 to 154)</td><td>32 fewer per 1000 (from 20 more to 66 fewer)</td></tr>
<tr><td>459 / 1000</td><td>349 / 1000 (234 to 528)</td><td>110 fewer per 1000 (from 69 more to 225 fewer)</td></tr>
</table>
Summary: Early tracheostomy, within 4, 7, or 10 days after the initiation of mechanical ventilation, in patients anticipated to require long-term</td>
<td rowspan="2">backgrounds as "patients with prolonged alterations in consciousness after head trauma" are also included. Therefore, the degree of indirectness was classified as 'serious', especially in terms of VFD. It is difficult to extract data for patients with ARDS exclusively from the selected reports. Therefore, VFD was not used as a clinical outcome for this clinical question.</td>
</tr>
<tr>
<td>Does the balance between desirable and undesirable effects favor the intervention or the comparison?</td>
<td>○Favors the comparison
○Probably favors the comparison
●Does not favor either the intervention or the comparison
○Probably favors the intervention
○Favors the intervention

○Varies
○Don't know</td>
</tr>
</table>

			mechanical ventilation did not significantly reduce the short-term mortality or the incidence of VAP.	
RESOURCES REQUIRED	**How large are the resource requirements (costs)?**	○Large costs<br>○Moderate costs<br>○Negligible costs and savings<br>●Moderate savings<br>○Large savings<br>------------------<br>○Varies<br>○Don't know	The cost of performing a tracheostomy in Japan is about 25,000 yen. Probably, there is no need to purchase special equipment to perform tracheostomy because it is a routine procedure.	
	**Does the cost-effectiveness of the intervention favor the intervention or the comparison?**	○Favors the comparison<br>●Probably favors the comparison<br>○Does not favor either the intervention or the comparison<br>○Probably favors the intervention<br>○Favors the intervention<br>------------------<br>○Varies<br>○No included studies	Since the mortality rate and the incidence of VAP are not decreased by early tracheostomy, if all patients undergo early tracheostomy, the costs increase along with the increased number of unnecessary tracheostomies. It has been reported that 91% of patients underwent tracheostomy with the early tracheostomy strategy while 54% underwent tracheostomy with the late tracheostomy strategy[5)]. Thus, approximately 40% of tracheostomy performed with the early tracheostomy strategy might be unnecessary if the late tracheostomy strategy is used. Except for patients who clearly or probably benefit from tracheostomy, the cost of early tracheostomy is considered to be high.	
EQUITY	**What would be the impact on health equity?**	○Reduced<br>○Probably reduced<br>○Probably no impact<br>○Probably increased<br>●Increased<br>------------------<br>○Varies<br>○Don't know	Special medical facilities or equipment are not required for this procedure.	
ACCEPTABILITY	**Is the intervention acceptable to key stakeholders?**	○No<br>○Probably no<br>○Probably yes<br>○Yes<br>------------------<br>●Varies<br>○Don't know	It cannot be said unconditionally because changes in the timing of tracheostomy have various influences on the stakeholders and the influences vary depending on their position.	
FEASIBILITY	**Is the intervention feasible implement?**	○No<br>○Probably no<br>○Probably yes<br>●Yes<br>------------------<br>○Varies<br>○Don't know	Special medical facilities or equipment are not required for this procedure.	

Recommendation

CQ1 : Should early tracheostomy be performed in adult patients with ARDS?					
**Balance of consequences**	Undesirable consequences clearly outweigh desirable consequences in most settings	Undesirable consequences probably outweigh desirable consequences in most settings	The balance between desirable and undesirable consequences is closely balanced or uncertain	Desirable consequences probably outweigh undesirable consequences in most settings	Desirable consequences clearly outweigh undesirable consequences in most settings
Judgement	○	○	●	○	○

**Type of recommendation**	Strong recommendation against the intervention	Conditional recommendation against the intervention	Conditional recommendation for either the intervention or the comparison	Conditional recommendation for the intervention
**Judgement**	○	●	○	○
**Recommendation**	**We suggest against early tracheostomy in adult patients with ARDS. (GRADE 2C, Strength of recommendation "weak recommendation" / Quality of evidence "low")**			
**Justification**	**Question:** Should early tracheostomy be performed in adult patients with ARDS?<br>**Patients:** Adult Patients who were anticipated to require prolonged mechanical ventilation<br>**Interventions:** early tracheostomy<br>**Comparison:** late tracheostomy<br>**Outcomes:** Short-term mortality (Note1, VAP (Note2<br><br>**Summary of the evidence:** There was no study conducted in adult patients with ARDS. We conducted systematical review for RCTs which conducted in patients who are anticipated to require long-term mechanical ventilation, and then found 19 RCTs. Since the number of days from initiation of mechanical ventilation to tracheostomy is generally 14 days in Japan, the studies were divided into an early and late tracheostomy groups with using thresholds of 4, 7, and 10 days from commencement of mechanical ventilation.<br>Early tracheostomy within 4, 7, and 10 days from the initial mechanical ventilation did not reduce the mortality and the incidence of VAP in compared with later tracheostomy. However, early tracheostomy is unlikely to worsen the patient outcome since the relative risk was less than 1.<br><br>**Quality of the evidence:** Overall, the selected studies had low risk of bias. Although the risk of bias for mortality was 'not serious', the risk of bias for VAP was downgraded by one level and classified as 'serious'.<br>Regarding with inconsistency of the results, heterogeneity for mortality (on 10 days) was low and 'not serious' as I^2=19%. However, heterogeneity for the others was high as $I^2 \geq 50\%$, thus, inconsistency of the results for them was downgraded by one level and classified as 'serious'.<br>Indirectness was considered as 'serious' in any of the outcomes because of the unmatched study subjects, as subjects included in selected RCTs were those anticipated to require long-term mechanical ventilation.<br>The level of imprecision was downgraded by one level for all outcomes since the confidence intervals overlap with the clinical decision thresholds.<br>For publication bias for the risk of VAP was downgraded by one level and classified as 'serious' because the result of the funnel plot test was asymmetric.<br>To consider above aspects, the overall quality of evidence was evaluated as 'low'.<br><br>**Judgement of benefit and harm, resources and cost:** Since ARDS patients requiring high oxygen concentration, high airway pressure, or high PEEP, they may have higher risk for tracheostomy, in compared with patients included in selected RCTs. As there is still no accurate methods to predict the long term mechanical ventilation, it is difficult to avoid the unnecessary tracheostomy, when early tracheostomy would be applied in all cases[6)]. It would be considered that 40 % of tracheostomy in early tracheostomy arm could be avoidable in late tracheostomy arm. Therefore, it cannot be determined that the benefits of performing early tracheostomy outweigh the harms of performing it in patients with ARDS.<br><br>**Recommendations;** We suggest against early tracheostomy in adult patients with ARDS. (GRADE 2C, Strength of recommendation "weak recommendation" / Quality of evidence "low")			

	**Additional considerations:** None
**Subgroup considerations**	None
**Implementation considerations**	For patients with severe ARDS presenting severe hypoxemia, cautious tracheostomy based on sufficient preparation will be required. When performing tracheostomy, informed consent from all persons concerned should be obtained, including medical staff as well as patient herself and her family.
**Monitoring and evaluation considerations**	The standard monitoring for respiration and circulation generally carried out in ICU is appropriate.
**Research possibilities**	A study to examine the optimal timing of tracheostomy in patients with ARDS is needed.<br>A method to accurately identify the patients requiring long-term mechanical ventilation is desired to be developed. With such a method, the number of unnecessary tracheostomy could be reduced and the evaluation regarding early tracheostomy could be changed.<br>There are mainly two types of tracheostomy: surgical tracheostomy and percutaneous tracheostomy. A study to investigate which type of tracheostomy is more safely performed in patients with ARDS is needed.

Note 1) Among the deaths within 90 days, those assessed as a primary outcome in each study.
Note 2) VAP: ventilator associated pneumonia. The definition of VAP varies among the studies.
Note 3) Out of 28 days, the number of days for which the patient is not dependent on the mechanical ventilator. If the patient dies within 28 days, the number should be zero.

1. Cheung NH, Napolitano LM: Tracheostomy: epidemiology, indications, timing, technique, and outcomes. *Respir Care* **59**(6): 895-915; discussion 916-899, 2014 PMID 24891198
2. Freeman BD, Morris PE: Tracheostomy practice in adults with acute respiratory failure. *Crit Care Med* **40**(10): 2890-2896, 2012 PMID 22824938
3. Epstein SK: Late complications of tracheostomy. *Respir Care* **50**(4): 542-549, 2005 PMID 15807919
4. Stauffer JL, Olson DE, Petty TL: Complications and consequences of endotracheal intubation and tracheotomy. A prospective study of 150 critically ill adult patients. *Am J Med* **70**(1): 65-76, 1981 PMID 7457492
5. Siempos I, Ntaidou TK, Filippidis FT, et al: Effect of early versus late or no tracheostomy on mortality and pneumonia of critically ill patients receiving mechanical ventilation: a systematic review and meta-analysis. *Lancet Respir Med* **3**(2): 150-158, 2015 PMID 25680911
6. Figueroa-Casas JB, Dwivedi AK, Connery SM, et al: Predictive models of prolonged mechanical ventilation yield moderate accuracy. *J Crit Care* **30**(3): 502-505, 2015 PMID 25682346

CQ02. Study flow diagram

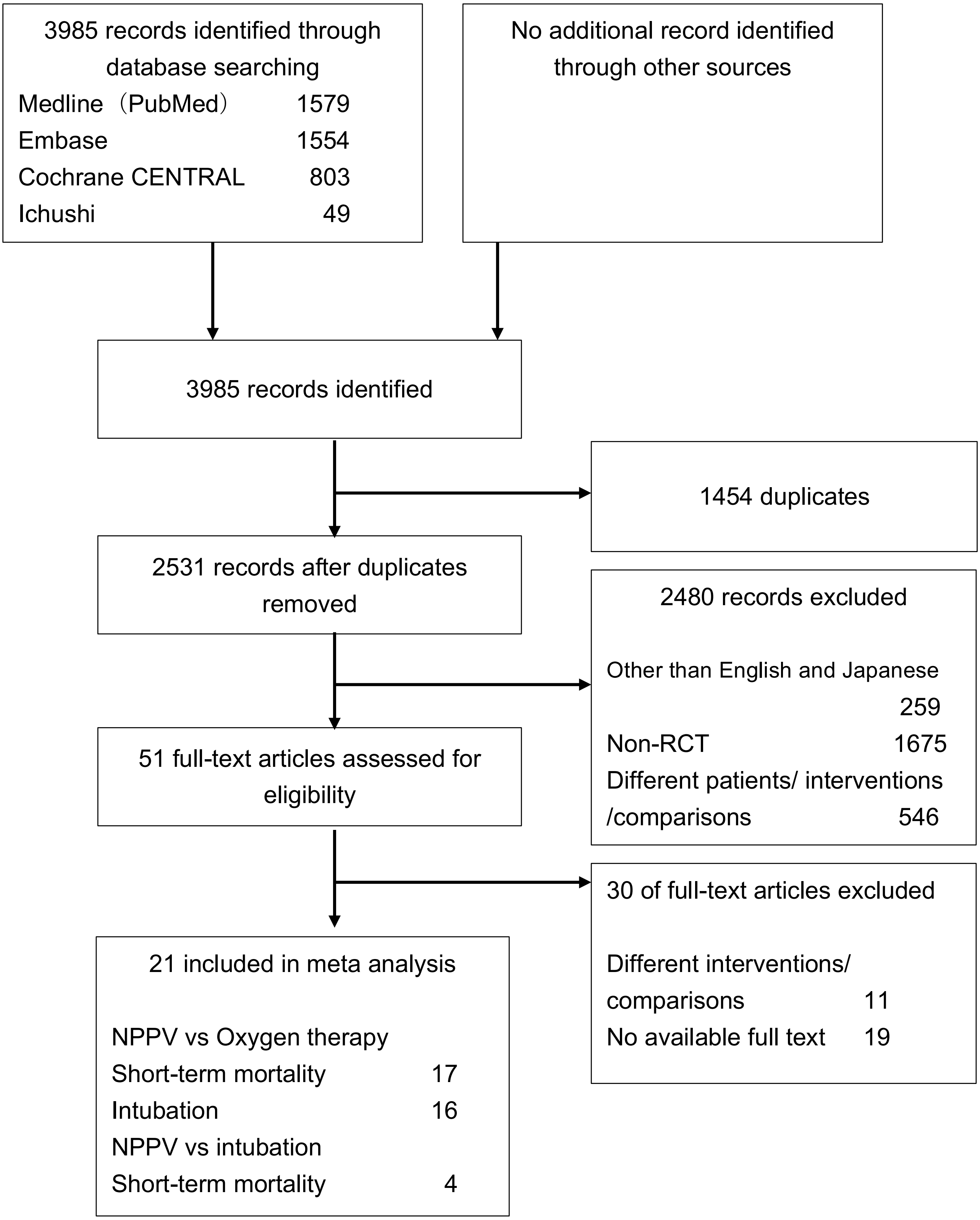

CQ02 Oxygen therapy
Risk of bias table, mortality

Outcome		Short Term mortality		risk of bias		not serious (0)			
番号	著者名 発表年（Forest plot表示）	risk of bias評価							
		ランダム割付順番の生成 random sequence generation	割り付けの隠蔽化 allocation concealment	ブラインド blinding 研究参加者と治療提供者 participants and personnel	ブラインド blinding アウトカム評価者 outcome assessors	不完全なアウトカムデータ incomplete outcome data	選択されたアウトカムの報告 selective outcome reporting	その他のバイアス Other sources of bias	研究内でのバイアスのリスク Risk of bias within a study
1	Antonelli 2000	Low risk	Low risk	High risk	Low risk	Low risk	Unclear risk	Low risk	Unclear risk
2	Brambilla 2014	low risk	Low risk	High risk	Low risk	Low risk	Low risk	Low risk	Low risk
3	Confalonieri 1999	Low risk	Low risk	High risk	Low risk	Low risk	Unclear risk	Low risk	Unclear risk
4	Cosentini 2010	Low risk	Low risk	High risk	Low risk	Low risk	Low risk	Low risk	Low risk
5	Delclaux 2000	Low risk	Low risk	High risk	Low risk	Low risk	Unclear risk	Low risk	Unclear risk
6	Ferrer 2003	Unclear risk	Unclear risk	High risk	Low risk	Low risk	Unclear risk	Low risk	Unclear risk
7	Gupta 2010	Low risk	Low risk	High risk	Low risk	Low risk	Low risk	Low risk	Low risk
8	Hernandez 2010	Low risk	Low risk	High risk	Low risk	Low risk	High risk	Low risk	High risk
9	Hilbert 2001	Unclear risk	Low risk	High risk	Low risk	Low risk	Unclear risk	Low risk	Unclear risk
10	Kramer 1995	Unclear risk	Unclear risk	High risk	Low risk	Low risk	Unclear risk	Low risk	Unclear risk
11	Martin 2000	Unclear risk	Unclear risk	High risk	Low risk	Low risk	Unclear risk	Low risk	Unclear risk
12	Nava 2013	Low risk	Low risk	High risk	Low risk	Low risk	Low risk	Low risk	Low risk
13	Squadrone 2010	Low risk	Low risk	High risk	Low risk	Low risk	Unclear risk	Low risk	Unclear risk
14	Wermke 2012	Low risk	Unclear risk	High risk	Low risk	Low risk	Unclear risk	Low risk	Unclear risk
15	Wood 1998	Unclear risk	Low risk	High risk	Low risk	Low risk	Unclear risk	Low risk	Unclear risk
16	Wysocki 1995	Low risk	Unclear risk	High risk	Low risk	Low risk	Unclear risk	Low risk	Unclear risk
17	Zhan 2012	Unclear risk	Low risk	High risk	Low risk	Low risk	Unclear risk	Low risk	Unclear risk

番号	著者名 発表年（Forest plot表示）	risk of biasコメント							
1	Antonelli 2000	コンピューターで作成	封筒法	NIVという治療の特性上、患者、担当者へのblindは不可能	評価者に対するブラインドによって、アウトカムは影響されない	データ欠損の報告なし	研究計画書を得られなかった	全項目ほぼLow risk	high riskが1つ、unclearが1つあり、バイアスの程度を評価できない
2	Brambilla 2014	random number generatorを利用	封筒法	NIVという治療の特性上、患者、担当者へのblindは不可能	評価者に対するブラインドによって、アウトカムは影響されない	データ欠損の報告なし	研究計画書どおり（NCT01383213）	全項目ほぼLow risk	ブラインド以外は全てLow risk。研究参加者と治療提供者に対するブラインドは、NIVの特性上ほぼ不可能である。
3	Confalonieri 1999	ソフトウェア(RND)を利用	封筒法	NIVという治療の特性上、患者、担当者へのblindは不可能	評価者に対するブラインドによって、アウトカムは影響されない	データ欠損の報告なし	研究計画書を得られなかった	全項目ほぼLow risk	high riskが1つ、unclearが1つあり、バイアスの程度を評価できない
4	Cosentini 2010	コンピュータで作成	封筒法	NIVという治療の特性上、患者、担当者へのblindは不可能	評価者に対するブラインドによって、アウトカムは影響されない	データ欠損の報告なし	研究計画書どおり（NCT00603564）	全項目ほぼLow risk	ブラインド以外は全てLow risk。研究参加者と治療提供者に対するブラインドは、NIVの特性上ほぼ不可能である。
5	Delclaux 2000	コンピュータで作成	封筒法	NIVという治療の特性上、患者、担当者へのblindは不可能	評価者に対するブラインドによって、アウトカムは影響されない	データ欠損の報告なし	研究計画書を得られなかった	全項目ほぼLow risk	high riskが1つ、unclearが1つあり、バイアスの程度を評価できない
6	Ferrer 2003	不明	不明	NIVという治療の特性上、患者、担当者へのblindは不可能	評価者に対するブラインドによって、アウトカムは影響されない	データ欠損の報告なし	研究計画書を得られなかった	全項目ほぼLow risk	Unclearの項目が多く、バイアスの程度を評価できない
7	Gupta 2010	コンピュータで作成	封筒法	NIVという治療の特性上、患者、担当者へのblindは不可能	評価者に対するブラインドによって、アウトカムは影響されない	データ欠損の報告なし	研究計画書どおり（NCT00510991）	全項目ほぼLow risk	ブラインド以外は全てLow risk。研究参加者と治療提供者に対するブラインドは、NIVの特性上ほぼ不可能である。
8	Hernandez 2010	random number generatorを利用	中央割り付け	NIVという治療の特性上、患者、担当者へのblindは不可能	評価者に対するブラインドによって、アウトカムは影響されない	データ欠損の報告なし	挿管の判定期間が、研究計画書（NCT 00557752）と実際とでは異なっている。	全項目ほぼLow risk	High riskが2つあり、risk of biasは高いと考えられる
9	Hilbert 2001	不明	封筒法	NIVという治療の特性上、患者、担当者へのblindは不可能	評価者に対するブラインドによって、アウトカムは影響されない	データ欠損の報告なし	研究計画書を得られなかった	全項目ほぼLow risk	Unclearの項目が多く、バイアスの程度を評価できない
10	Kramer 1995	不明	不明	NIVという治療の特性上、患者、担当者へのblindは不可能	評価者に対するブラインドによって、アウトカムは影響されない	データ欠損の報告なし	研究計画書を得られなかった	全項目ほぼLow risk	Unclearの項目が多く、バイアスの程度を評価できない
11	Martin 2000	不明	不明	NIVという治療の特性上、患者、担当者へのblindは不可能	評価者に対するブラインドによって、アウトカムは影響されない	データ欠損の報告なし	研究計画書を得られなかった	全項目ほぼLow risk	Unclearの項目が多く、バイアスの程度を評価できない
12	Nava 2013	コンピュータで作成	封筒法	NIVという治療の特性上、患者、担当者へのblindは不可能	評価者に対するブラインドによって、アウトカムは影響されない	データ欠損の報告なし	研究計画書どおり（NCT00533143）	全項目ほぼLow risk	ブラインド以外は全てLow risk。研究参加者と治療提供者に対するブラインドは、NIVの特性上ほぼ不可能である。
13	Squadrone 2010	コンピュータで作成	中央割り付け	NIVという治療の特性上、患者、担当者へのblindは不可能	評価者に対するブラインドによって、アウトカムは影響されない	データ欠損の報告なし	研究計画書を得られなかった	全項目ほぼLow risk	high riskが1つ、unclearが1つあり、バイアスの程度を評価できない
14	Wermke 2012	コンピュータで作成	不明	NIVという治療の特性上、患者、担当者へのblindは不可能	評価者に対するブラインドによって、アウトカムは影響されない	データ欠損の報告なし	研究計画書を得られなかった	全項目ほぼLow risk	Unclearの項目が多く、バイアスの程度を評価できない
15	Wood 1998	不明	封筒法	NIVという治療の特性上、患者、担当者へのblindは不可能	評価者に対するブラインドによって、アウトカムは影響されない	データ欠損の報告なし	研究計画書を得られなかった	全項目ほぼLow risk	Unclearの項目が多く、バイアスの程度を評価できない
16	Wysocki 1995	乱数表を用いて作成	不明	NIVという治療の特性上、患者、担当者へのblindは不可能	評価者に対するブラインドによって、アウトカムは影響されない	データ欠損の報告なし	研究計画書を得られなかった	全項目ほぼLow risk	Unclearの項目が多く、バイアスの程度を評価できない
17	Zhan 2012	不明	中央割り付け	NIVという治療の特性上、患者、担当者へのblindは不可能	評価者に対するブラインドによって、アウトカムは影響されない	データ欠損の報告なし	研究計画書を得られなかった	全項目ほぼLow risk	Unclearの項目が多く、バイアスの程度を評価できない

CQ02 Oxygen therapy
Risk of bias table, Intubation

Outcome		Intubation		risk of bias		serious (−1)			
番号	著者名 発表年（Forest plot表示）	risk of bias評価							
		ランダム割付順番の生成 random sequence generation	割り付けの隠蔽化 allocation concealment	ブラインド blinding 研究参加者と治療提供者 participants and personnel	ブラインド blinding アウトカム評価者 outcome assessors	不完全なアウトカムデータ incomplete outcome data	選択されたアウトカムの報告 selective outcome reporting	その他のバイアス Other sources of bias	研究内でのバイアスのリスク Risk of bias within a study
1	Antonelli 2000	Low risk	Low risk	High risk	Unclear risk	Low risk	Unclear risk	Low risk	Unclear risk
2	Brambilla 2014	low risk	Low risk	High risk	Low risk	Low risk	Low risk	Low risk	Low risk
3	Confalonieri 1999	Low risk	Low risk	High risk	High risk	Low risk	Unclear risk	Low risk	High risk
4	Cosentini 2010	Low risk	Low risk	High risk	Unclear risk	Low risk	Low risk	Low risk	Unclear risk
5	Delclaux 2000	Low risk	Low risk	High risk	Unclear risk	Low risk	Unclear risk	Low risk	Unclear risk
6	Ferrer 2003	Unclear risk	Unclear risk	High risk	Unclear risk	Low risk	Unclear risk	Low risk	Unclear risk
7	Gupta 2010	Low risk	Low risk	High risk	Unclear risk	Low risk	Low risk	Low risk	Unclear risk
8	Hernandez 2010	Low risk	Low risk	High risk	Low risk	Low risk	High risk	Low risk	High risk
9	Hilbert 2001	Unclear risk	Low risk	High risk	Low risk	Low risk	Unclear risk	Low risk	Unclear risk
10	Kramer 1995	Unclear risk	Unclear risk	High risk	High risk	Low risk	Unclear risk	Low risk	High risk
11	Martin 2000	Unclear risk	Unclear risk	High risk	High risk	Low risk	Unclear risk	Low risk	High risk
13	Squadrone 2010	Low risk	Low risk	High risk	Low risk	Low risk	Unclear risk	Low risk	Unclear risk
14	Wermke 2012	Low risk	Unclear risk	High risk	High risk	Low risk	Unclear risk	Low risk	High risk
15	Wood 1998	Unclear risk	Low risk	High risk	High risk	Low risk	Unclear risk	Low risk	High risk
16	Wysocki 1995	Low risk	Unclear risk	High risk	Low risk	Low risk	Unclear risk	Low risk	Unclear risk
17	Zhan 2012	Unclear risk	Low risk	High risk	High risk	Low risk	Unclear risk	Low risk	High risk

番号	著者名 発表年（Forest plot表示）	risk of biasコメント							
1	Antonelli 2000	コンピューターで作成	封筒法	NIVという治療の特性上、患者、担当者へのblindは不可能	挿管の判断をする担当者の記載がない	データ欠損の報告なし	研究計画書を得られなかった	全項目ほぼLow risk	Unclearの項目が多く、バイアスの程度を評価できない
2	Brambilla 2014	random number generatorを利用	封筒法	NIVという治療の特性上、患者、担当者へのblindは不可能	挿管の判断をするsenior physiciansには介入がブラインドされている	データ欠損の報告なし	研究計画書どおり（NCT01383213）	全項目ほぼLow risk	ブラインド以外は全てLow risk。研究参加者と治療提供者に対するブラインドは、NIVの特性上ほぼ不可能である。
3	Confalonieri 1999	ソフトウェア(RND)を利用	封筒法	NIVという治療の特性上、患者、担当者へのblindは不可能	担当医と研究者によって挿管が決定	データ欠損の報告なし	研究計画書を得られなかった	全項目ほぼLow risk	High riskが2つあり、risk of biasは高いと考えられる
4	Cosentini 2010	コンピュータで作成	封筒法	NIVという治療の特性上、患者、担当者へのblindは不可能	挿管の判断をする医療者は不明	データ欠損の報告なし	研究計画書どおり（NCT00603564）	全項目ほぼLow risk	high riskが1つ、unclearが1つあり、バイアスの程度を評価できない
5	Delclaux 2000	コンピュータで作成	封筒法	NIVという治療の特性上、患者、担当者へのblindは不可能	挿管基準は決まっているものの、挿管の判断をする医療者は不明	データ欠損の報告なし	研究計画書を得られなかった	全項目ほぼLow risk	Unclearの項目が多く、バイアスの程度を評価できない
6	Ferrer 2003	不明	不明	NIVという治療の特性上、患者、担当者へのblindは不可能	挿管基準は決まっている者のの、挿管の判断をする医療者は不明	データ欠損の報告なし	研究計画書を得られなかった	全項目ほぼLow risk	Unclearの項目が多く、バイアスの程度を評価できない
7	Gupta 2010	コンピュータで作成	封筒法	NIVという治療の特性上、患者、担当者へのblindは不可能	挿管基準・判断をする医療者が不明	データ欠損の報告なし	研究計画書どおり(NCT00510991)	全項目ほぼLow risk	high riskが1つ、unclearが1つあり、バイアスの程度を評価できない
8	Hernandez 2010	random number generatorを利用	中央割り付け	NIVという治療の特性上、患者、担当者へのblindは不可能	挿管基準が明確	データ欠損の報告なし	挿管の判定期間が、研究計画書(NCT 00557752)と実際とでは異なっている。	全項目ほぼLow risk	High riskが2つあり、risk of biasは高いと考えられる
9	Hilbert 2001	不明	封筒法	NIVという治療の特性上、患者、担当者へのblindは不可能	挿管基準が明確	データ欠損の報告なし	研究計画書を得られなかった	全項目ほぼLow risk	Unclearの項目が多く、バイアスの程度を評価できない
10	Kramer 1995	不明	不明	NIVという治療の特性上、患者、担当者へのblindは不可能	担当医によって挿管が決定	データ欠損の報告なし	研究計画書を得られなかった	全項目ほぼLow risk	High riskが2つあり、risk of biasは高いと考えられる
11	Martin 2000	不明	不明	NIVという治療の特性上、患者、担当者へのblindは不可能	担当医によって挿管が決定	データ欠損の報告なし	研究計画書を得られなかった	全項目ほぼLow risk	High riskが2つあり、risk of biasは高いと考えられる
13	Squadrone 2010	コンピュータで作成	中央割り付け	NIVという治療の特性上、患者、担当者へのblindは不可能	挿管基準が明確	データ欠損の報告なし	研究計画書を得られなかった	全項目ほぼLow risk	high riskが1つ、unclearが1つあり、バイアスの程度を評価できない
14	Wermke 2012	コンピュータで作成	不明	NIVという治療の特性上、患者、担当者へのblindは不可能	挿管基準は存在するが、判断する担当者の主観が関与する項目がある	データ欠損の報告なし	研究計画書を得られなかった	全項目ほぼLow risk	High riskが2つあり、risk of biasは高いと考えられる
15	Wood 1998	不明	封筒法	NIVという治療の特性上、患者、担当者へのblindは不可能	挿管基準は存在するが、判断する担当者の主観が関与する項目がある	データ欠損の報告なし	研究計画書を得られなかった	全項目ほぼLow risk	High riskが2つあり、risk of biasは高いと考えられる
16	Wysocki 1995	乱数表を用いて作成	不明	NIVという治療の特性上、患者、担当者へのblindは不可能	挿管基準が明確	データ欠損の報告なし	研究計画書を得られなかった	全項目ほぼLow risk	Unclearの項目が多く、バイアスの程度を評価できない

CQ02 conventional mechanical ventilation
Risk of bias table, mortality

Outcome		Short term mortality		risk of bias		not serious (0)			
番号	著者名 発表年（Forest plot表示）	risk of bias評価							
		ランダム割付順番の生成 random sequence generation	割り付けの隠蔽化 allocation concealment	ブラインド blinding		不完全なアウトカムデータ incomplete outcome data	選択されたアウトカムの報告 selective outcome reporting	その他のバイアス Other sources of bias	研究内でのバイアスのリスク Risk of bias within a study
				研究参加者と治療提供者 participants and personnel	アウトカム評価者 outcome assessors				
1	Antonelli 1998	unclear risk	Unclear risk	High risk	Low risk	Low risk	Unclear risk	Low risk	Unclear risk
2	Gunduz 2005	Unclear risk	Unclear risk	High risk	Low risk	Low risk	Unclear risk	Low risk	Unclear risk
3	Honrubia 2005	Low risk	Low risk	High risk	Low risk	Low risk	Unclear risk	High risk	High risk
4	Matic 2007	Unclear risk	Low risk	High risk	Low risk	Low risk	Unclear risk	Low risk	Unclear risk
番号	著者名 発表年（Forest plot表示）	risk of biasコメント							
1	Antonelli 1998	ランダム化の方法が未記載	割り付けの隠蔽化についての記載なし	NIVという治療の特性上、患者、担当者へのblindは不可能	評価者に対するブラインドによって、アウトカムは影響されない	データ欠損の報告なし	研究計画書を得られなかった	全項目ほぼLow risk	Unclearの項目が多く、バイアスの程度を評価できない
2	Gunduz 2005	不明	不明	NIVという治療の特性上、患者、担当者へのblindは不可能	評価者に対するブラインドによって、アウトカムは影響されない	データ欠損の報告なし	研究計画書を得られなかった	全項目ほぼLow risk	Unclearの項目が多く、バイアスの程度を評価できない
3	Honrubia 2005	コンピュータで作成	中央割り付け	NIVという治療の特性上、患者、担当者へのblindは不可能	評価者に対するブラインドによって、アウトカムは影響されない	データ欠損の報告なし	研究計画書を得られなかった	途中で打ち切り	High riskが2つあり、risk of biasは高いと考えられる
4	Matic 2007	不明	封筒法	NIVという治療の特性上、患者、担当者へのblindは不可能	評価者に対するブラインドによって、アウトカムは影響されない	データ欠損の報告なし	研究計画書を得られなかった	全項目ほぼLow risk	バイアスの程度を評価できない

CQ02 Risk of bias summary, Risk of bias graph

Short term mortality (NPPV vs Oxygen therapy)

	Random sequence generation (selection bias)	Allocation concealment (selection bias)	Blinding of participants and personnel (performance bias)	Blinding of outcome assessment (detection bias)	Incomplete outcome data (attrition bias)	Selective reporting (reporting bias)	Other bias
Antonelli 2000	+	+	−	+	+	?	+
Brambilla 2014	+	+	−	+	+	+	+
Confalonieri 1999	+	+	−	+	+	?	+
Cosentini 2010	+	+	−	+	+	+	+
Delclaux 2000	+	+	−	+	+	?	+
Ferrer 2003	?	?	−	+	+	?	+
Gupta 2010	+	+	−	+	+	+	+
Hernandez 2010	+	+	−	+	+	−	+
Hilbert 2001	?	+	−	+	+	?	+
Kramer 1995	?	?	−	+	+	?	+
Martin 2000	?	?	−	+	+	?	+
Nava 2013	+	+	−	+	+	+	+
Squadrone 2010	+	+	−	+	+	+	+
Wermke 2012	+	?	−	+	+	?	+
Wood 1998	?	+	−	+	+	?	+
Wysocki 1995	+	?	−	+	+	?	+
Zhan 2012	?	+	−	+	+	?	+

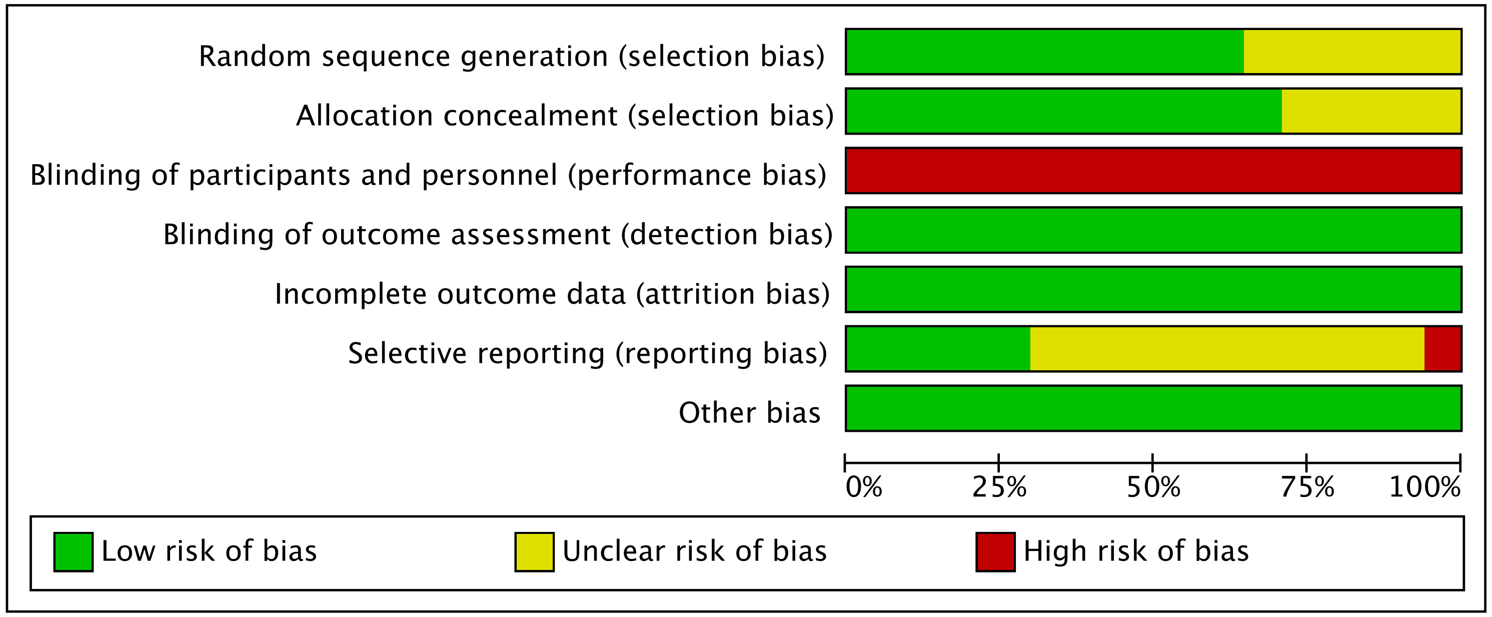

CQ02 Risk of bias summary, Risk of bias graph

Intubation （NPPV vs Oxygen therapy）

	Random sequence generation (selection bias)	Allocation concealment (selection bias)	Blinding of participants and personnel (performance bias)	Blinding of outcome assessment (detection bias)	Incomplete outcome data (attrition bias)	Selective reporting (reporting bias)	Other bias
Antonelli 2000	+	+	-	?	+	?	+
Brambilla 2014	+	+	-	+	+	+	+
Confalonieri 1999	+	+	-	-	+	?	+
Cosentini 2010	+	+	-	?	+	+	+
Delclaux 2000	+	+	-	?	+	?	+
Ferrer 2003	?	?	-	?	+	?	+
Gupta 2010	+	+	-	?	+	+	+
Hernandez 2010	+	+	-	+	+	-	+
Hilbert 2001	?	+	-	+	+	?	+
Kramer 1995	?	?	-	-	+	?	+
Martin 2000	?	?	-	-	+	?	+
Squadrone 2010	+	+	-	+	+	+	+
Wermke 2012	+	?	-	-	+	?	+
Wood 1998	?	+	-	-	+	?	+
Wysocki 1995	+	?	-	+	+	?	+
Zhan 2012	?	+	-	+	+	?	+

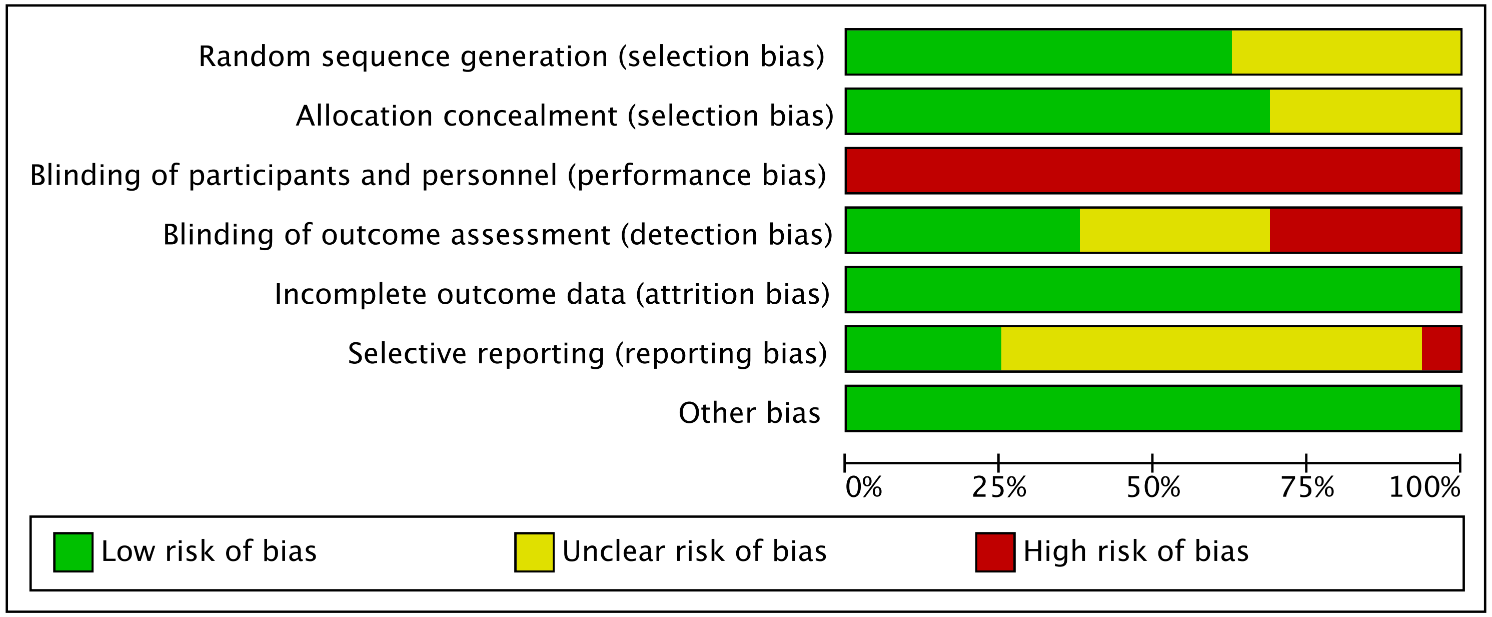

Short term mortality (NPPV vs Intubation)

	Random sequence generation (selection bias)	Allocation concealment (selection bias)	Blinding of participants and personnel (performance bias)	Blinding of outcome assessment (detection bias)	Incomplete outcome data (attrition bias)	Selective reporting (reporting bias)	Other bias
Antonelli 1998	?	?	−	+	+	?	+
Gunduz 2005	?	?	−	+	+	?	+
Honrubia 2005	+	+	−	+	+	?	−
Matic 2007	?	+	−	+	+	?	+

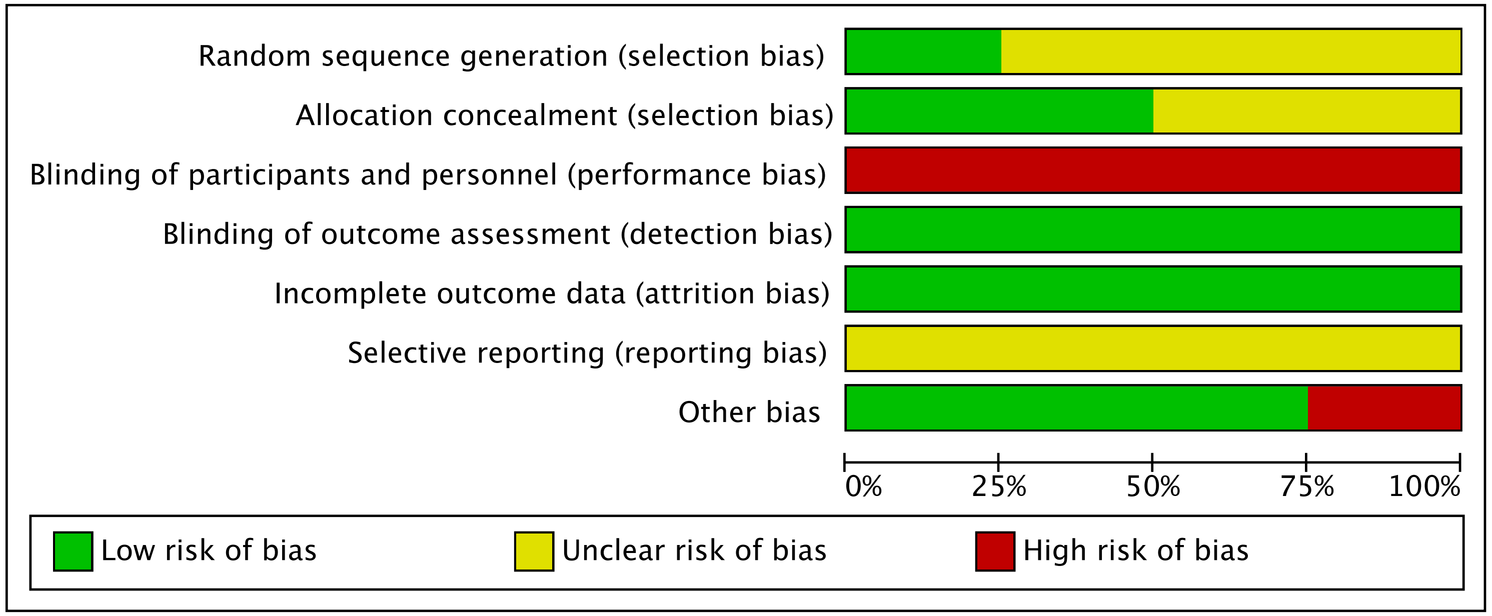

Short term mortality (NPPV vs Oxygen therapy)

	NPPV		Oxygen therapy			Risk Ratio
**Study or Subgroup**	**Events**	**Total**	**Events**	**Total**	**Weight**	**M-H, Random, 95% CI**
Antonelli 2000	7	20	11	20	8.2%	0.64 [0.31, 1.30]
Brambilla 2014	2	40	7	41	2.7%	0.29 [0.06, 1.33]
Confalonieri 1999	7	28	6	28	5.6%	1.17 [0.45, 3.04]
Cosentini 2010	0	20	0	27		Not estimable
Delclaux 2000	19	61	18	62	11.0%	1.07 [0.63, 1.84]
Ferrer 2003	9	51	21	54	8.7%	0.45 [0.23, 0.90]
Gupta 2010	0	28	0	25		Not estimable
Hernandez 2010	1	25	1	25	0.9%	1.00 [0.07, 15.12]
Hilbert 2001	13	26	21	26	13.2%	0.62 [0.40, 0.95]
Kramer 1995	1	16	2	15	1.3%	0.47 [0.05, 4.65]
Martin 2000	5	32	10	29	5.6%	0.45 [0.18, 1.17]
Nava 2013	61	99	66	101	17.8%	0.94 [0.76, 1.16]
Squadrone 2010	3	20	15	20	4.7%	0.20 [0.07, 0.59]
Wermke 2012	16	42	14	44	10.3%	1.20 [0.67, 2.14]
Wood 1998	4	16	0	11	0.8%	6.35 [0.38, 107.30]
Wysocki 1995	7	21	10	20	7.8%	0.67 [0.32, 1.41]
Zhan 2012	1	21	5	19	1.5%	0.18 [0.02, 1.41]
**Total (95% CI)**		**566**		**567**	**100.0%**	**0.71 [0.54, 0.92]**
Total events	156		207			

Heterogeneity: $Tau^2 = 0.09$; $Chi^2 = 25.12$, df = 14 (P = 0.03); $I^2 = 44\%$
Test for overall effect: Z = 2.58 (P = 0.01)

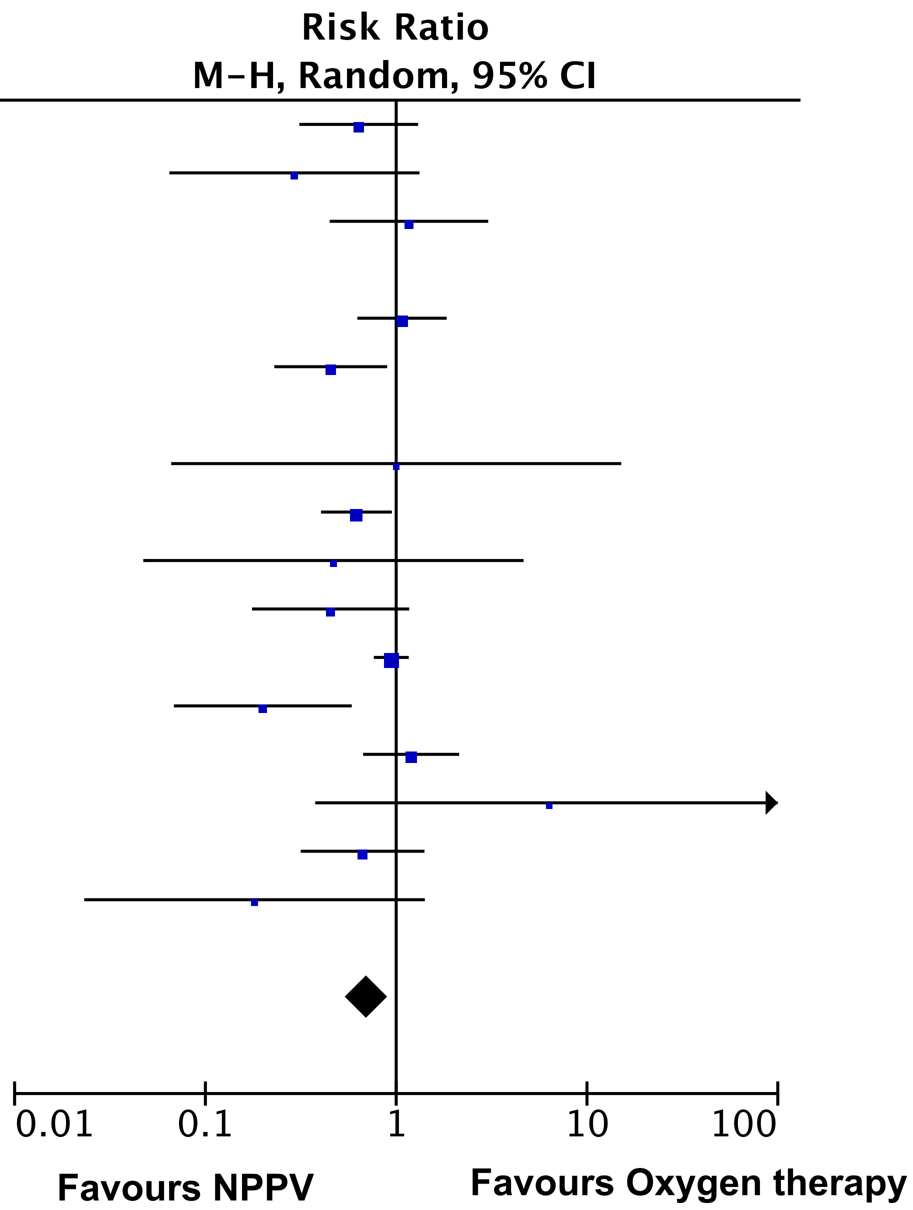

Intubation（NPPV vs Oxygen therapy）

	NPPV		Oxygen therapy			Risk Ratio
Study or Subgroup	Events	Total	Events	Total	Weight	M-H, Random, 95% CI
Antonelli 2000	4	20	14	20	5.2%	0.29 [0.11, 0.72]
Brambilla 2014	2	40	1	40	0.9%	2.00 [0.19, 21.18]
Confalonieri 1999	6	28	14	28	6.5%	0.43 [0.19, 0.95]
Cosentini 2010	0	20	0	27		Not estimable
Delclaux 2000	21	61	24	62	13.4%	0.89 [0.56, 1.42]
Ferrer 2003	13	51	28	54	11.5%	0.49 [0.29, 0.84]
Gupta 2010	2	28	0	25	0.6%	4.48 [0.23, 89.13]
Hernandez 2010	3	25	10	25	3.5%	0.30 [0.09, 0.96]
Hilbert 2001	12	26	20	26	13.5%	0.60 [0.38, 0.96]
Kramer 1995	5	16	11	15	6.7%	0.43 [0.19, 0.94]
Martin 2000	9	32	17	29	9.2%	0.48 [0.25, 0.90]
Squadrone 2010	2	20	8	20	2.4%	0.25 [0.06, 1.03]
Wermke 2012	6	42	11	44	5.4%	0.57 [0.23, 1.41]
Wood 1998	7	16	5	11	5.9%	0.96 [0.41, 2.26]
Wysocki 1995	13	21	14	20	14.3%	0.88 [0.57, 1.38]
Zhan 2012	1	21	4	19	1.2%	0.23 [0.03, 1.85]
**Total (95% CI)**		**467**		**465**	**100.0%**	**0.58 [0.46, 0.74]**
Total events	106		181			

Heterogeneity: $Tau^2 = 0.05$; $Chi^2 = 18.58$, df = 14 (P = 0.18); $I^2 = 25\%$
Test for overall effect: Z = 4.57 (P < 0.00001)

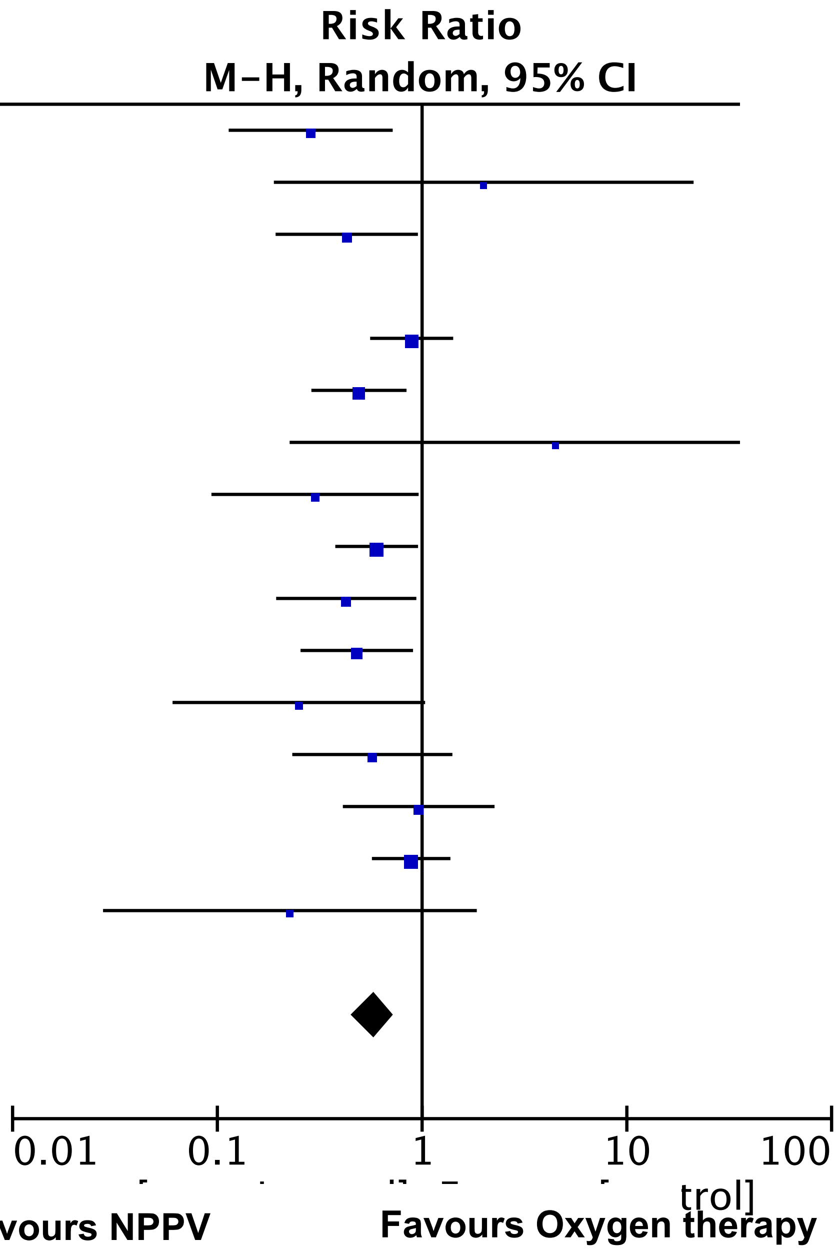

ias)

Short term mortality (NPPV vs Intubation)

	NPPV		Oxygen therapy			Risk Ratio
**Study or Subgroup**	**Events**	**Total**	**Events**	**Total**	**Weight**	**M-H, Random, 95% CI**
Antonelli 1998	10	32	16	32	32.7%	0.63 [0.34, 1.16]
Gunduz 2005	2	22	7	21	5.9%	0.27 [0.06, 1.17]
Honrubia 2005	10	31	14	33	30.0%	0.76 [0.40, 1.45]
Matic 2007	15	195	21	192	31.4%	0.70 [0.37, 1.32]
**Total (95% CI)**		**280**		**278**	**100.0%**	**0.65 [0.46, 0.93]**
Total events	37		58			

Heterogeneity: $Tau^2 = 0.00$; $Chi^2 = 1.68$, df = 3 (P = 0.64); $I^2 = 0\%$
Test for overall effect: Z = 2.34 (P = 0.02)

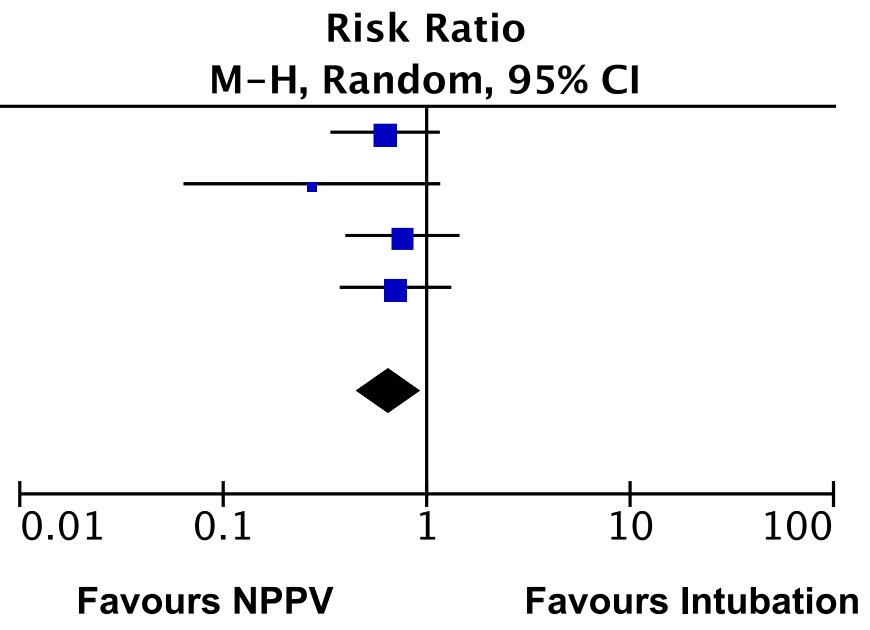

Short term mortality
(NPPV vs Oxygen therapy)

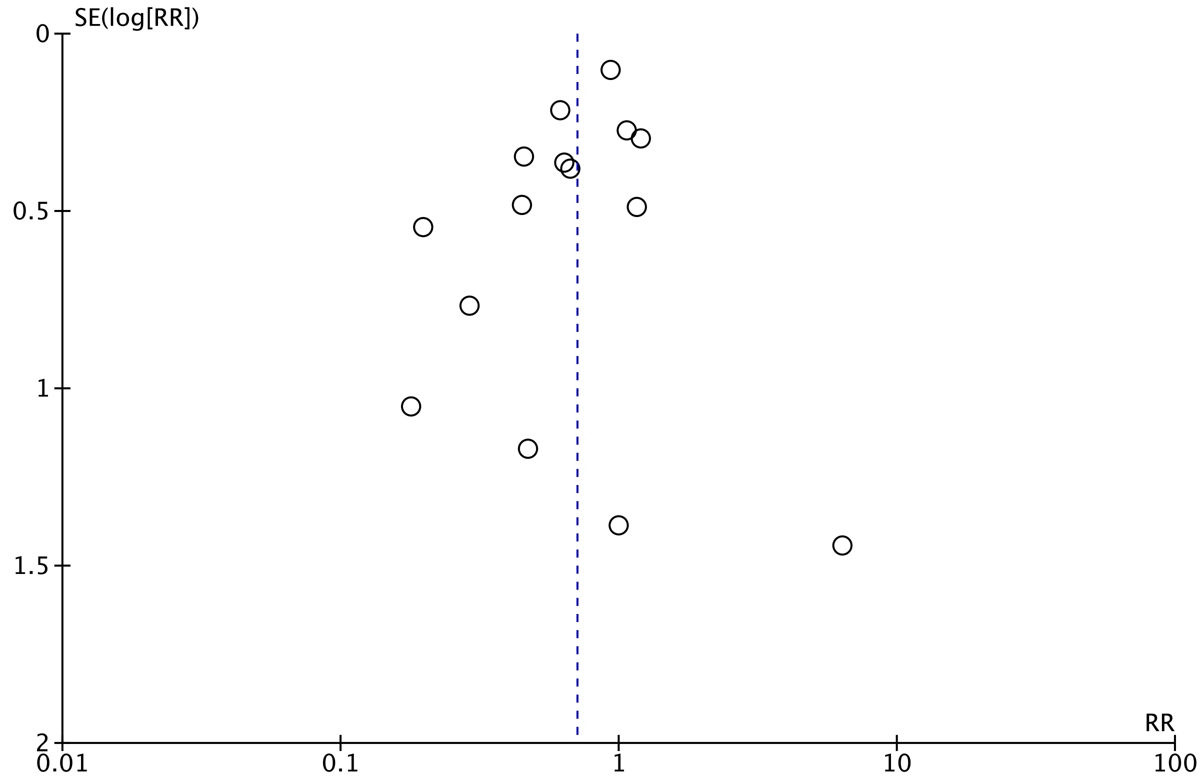

Intubation （NPPV vs Oxygen therapy）

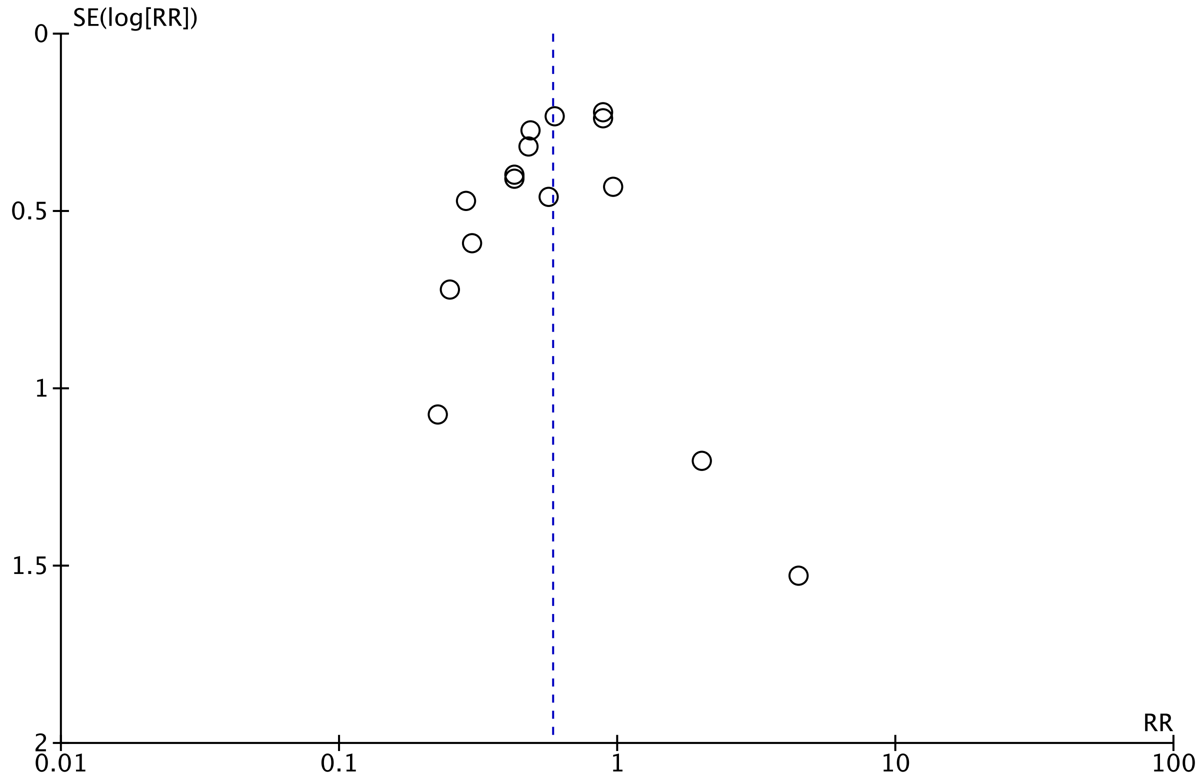

Summary of findings:

CQ2: Should non-invasive positive pressure ventilation (NPPV) be used as early respiratory management in adult patients with ARDS? Oxygen therapy vs. NPPV

Patient or population: Adult patients with hypoxemia
Intervention: NPPV
Comparison: oxygen therapy

Outcomes	Anticipated absolute effects* (95% CI)		Relative effect (95% CI)	№ of participants (studies)	Quality of the evidence (GRADE)	Comments
	Risk with oxygen therapy	Risk with NPPV				
Mortality (Short-term) (in hospital or ICU)	**Study population**		**RR 0.71** (0.54 to 0.92)	1133 (17 RCTs)	⨁⨁◯◯ LOW [1,2,3,4]	
	365 per 1,000	**259 per 1,000** (197 to 336)				
	**Low**					
	133 per 1,000	**94 per 1,000** (72 to 122)				
	**High**					
	750 per 1,000	**533 per 1,000** (405 to 690)				
Intubation	**Study population**		**RR 0.58** (0.46 to 0.74)	932 (16 RCTs)	⨁⨁◯◯ LOW [3,5,6,7]	
	389 per 1,000	**226 per 1,000** (179 to 288)				
	**Low**					
	211 per 1,000	**122 per 1,000** (97 to 156)				
	**High**					
	733 per 1,000	**425 per 1,000** (337 to 542)				

Summary of findings:

CQ2: Should non-invasive positive pressure ventilation (NPPV) be used as early respiratory management in adult patients with ARDS? Oxygen therapy vs. NPPV

Patient or population: Adult patients with hypoxemia
Intervention: NPPV
Comparison: oxygen therapy

Outcomes	Anticipated absolute effects* (95% CI)		Relative effect (95% CI)	№ of participants (studies)	Quality of the evidence (GRADE)	Comments
	Risk with oxygen therapy	Risk with NPPV				

***The risk in the intervention group** (and its 95% confidence interval) is based on the assumed risk in the comparison group and the **relative effect** of the intervention (and its 95% CI).

CI: Confidence interval; **RR:** Risk ratio

GRADE Working Group grades of evidence
High quality: We are very confident that the true effect lies close to that of the estimate of the effect
Moderate quality: We are moderately confident in the effect estimate: The true effect is likely to be close to the estimate of the effect, but there is a possibility that it is substantially different
Low quality: Our confidence in the effect estimate is limited: The true effect may be substantially different from the estimate of the effect
Very low quality: We have very little confidence in the effect estimate: The true effect is likely to be substantially different from the estimate of effect

1. Although it was impossible to blind patients or caregivers in RCTs evaluating NPPV, lack of blinding was not considered as a factor of downgrading because of a property of mortality as an outcome. In some RCTs, allocation concealment and selective outcome reporting were ‘unclear’, however we decided not to downgrade.
2. The point estimates were significantly inconsistent across the studies and heterogeneity was moderate (I^2 = 44%).
3. Subjects were patients with hypoxemia, not ARDS.
4. RR 0.71 [0.54-0.92]. Criteria of the optimal information size (OIS) were met.
5. Decision of intubation depended on clinicians at bedside and in 6 of 21 RCTs included, blinding outcome assessors was considered as ‘high risk.’
6. Variance of point estimates across studies was not significant and heterogeneity was low (I^2 = 25%).
7. RR 0.58 [0.46-0.74]. Criteria of the OIS were met.

Summary of findings:

CQ2: Should non-invasive positive pressure ventilation (NPPV) be used as early respiratory management in adult patients with ARDS? Conventional mechanical ventilation vs. NPPV

Patient or population: Adult patients with hypoxemia
Intervention: NPPV
Comparison: Conventional mechanical ventilation

Outcomes	Anticipated absolute effects* (95% CI)		Relative effect (95% CI)	№ of participants (studies)	Quality of the evidence (GRADE)	Comments
	Risk with invasive MV	Risk with NIV				
Mortality (Short-term) (in hospital or ICU)	**Study population**		**RR 0.65** (0.46 to 0.93)	558 (4 RCTs)	⊕⊕◯◯ LOW [1,2,3,4]	
	209 per 1,000	**136 per 1,000** (96 to 194)				
	**Moderate**					
	379 per 1,000	**246 per 1,000** (174 to 352)				

***The risk in the intervention group** (and its 95% confidence interval) is based on the assumed risk in the comparison group and the **relative effect** of the intervention (and its 95% CI).

CI: Confidence interval; **RR:** Risk ratio

GRADE Working Group grades of evidence
High quality: We are very confident that the true effect lies close to that of the estimate of the effect
Moderate quality: We are moderately confident in the effect estimate: The true effect is likely to be close to the estimate of the effect, but there is a possibility that it is substantially different
Low quality: Our confidence in the effect estimate is limited: The true effect may be substantially different from the estimate of the effect
Very low quality: We have very little confidence in the effect estimate: The true effect is likely to be substantially different from the estimate of effect

1. It was impossible to blind patients or caregivers in RCTs evaluating NPPV, and lack of blinding was not considered as a factor of downgrading because of a property of mortality as an outcome. In some RCTs, allocation concealment and selective outcome reporting were 'unclear', however we decided not to downgrade.
2. Variance of the point estimates across studies was not significant and heterogeneity was low (I^2 = 0%).
3. Subjects were patients with hypoxemia, not ARDS.
4. RR 0.65 [0.46-0.93]. Criteria of the optimal information size (OIS) were not met.

CQ2:
Question: Should non-invasive positive pressure ventilation (NPPV) be used as early respiratory management in adult patients with ARDS?

Oxygen therapy vs. NPPV

Quality assessment							№ of patients		Effect		Quality	Importance
**№ of studies**	**Study design**	**Risk of bias**	**Inconsistency**	**Indirectness**	**Imprecision**	**Other considerations**	**NPPV**	**oxygen therapy**	**Relative (95% CI)**	**Absolute (95% CI)**		
Short term Mortality [Note 1)]												
17	Randomised trials	Not serious [1]	Serious [2]	Serious [3]	Not serious [4]	None	156/566 (27.6%)	207/567 (36.5%)	**RR 0.71** (0.54 to 0.92)	**106 fewer per 1,000** (from 29 fewer to 168 fewer)	⨁⨁◯◯ LOW	CRITICAL
								13.3%		**39 fewer per 1,000** (from 11 fewer to 61 fewer)		
								75.0%		**218 fewer per 1,000** (from 60 fewer to 345 fewer)		
Intubation												
16	Randomised trials	Serious [5]	Not serious [6]	Serious [3]	Not serious [7]	None	106/467 (22.7%)	181/465 (38.9%)	**RR 0.58** (0.46 to 0.74)	**163 fewer per 1,000** (from 101 fewer to 210 fewer)	⨁⨁◯◯ LOW	IMPORTANT
								21.1%		**89 fewer per 1,000** (from 55 fewer to 114 fewer)		
								73.3%		**308 fewer per 1,000** (from 191 fewer to 396 fewer)		

CI: Confidence interval; **RR:** Risk ratio

1. Although it was impossible to blind patients or caregivers in RCTs evaluating NPPV, lack of blinding was not considered as a factor of downgrading because of a property of mortality as an outcome. In some RCTs, allocation concealment and selective outcome reporting were 'unclear', however we decided not to downgrade.
2. The point estimates were significantly inconsistent across the studies. According to I2, heterogeneity was considered to be moderate (I^2 = 44%).
3. Subjects were patients with hypoxemia, not ARDS.
4. RR 0.71 [0.54-0.92]. Criteria of the optimal information size (OIS) were met.
5. Decision of intubation depended on clinicians at bedside and in 6 of 21 RCTs included, blinding outcome assessors was considered as 'high risk.'
6. Variance of point estimates across studies was not significant. According to I2, heterogeneity was considered to be low (I^2 = 25%).
7. RR 0.58 [0.46-0.74]. Criteria of the OIS were met.

CQ2:

Question: Should non-invasive positive pressure ventilation (NPPV) be used as early respiratory management in adult patients with ARDS?

Conventional mechanical ventilation vs. NPPV

Quality assessment							№ of patients		Effect		Quality	Importance
**№ of studies**	**Study design**	**Risk of bias**	**Inconsistency**	**Indirectness**	**Imprecision**	**Other considerations**	**NPPV**	**Conventional mechanical ventilation**	**Relative (95% CI)**	**Absolute (95% CI)**		
Short term Mortality [Note 1)]												
4	randomised trials	not serious [1]	not serious [2]	serious [3]	serious [4]	none	37/280 (13.2%)	58/278 (20.9%)	**RR 0.65** (0.46 to 0.93)	73 fewer per 1,000 (from 15 fewer to 113 fewer)	⨁⨁◯◯ LOW	CRITICAL
								37.9%		133 fewer per 1,000 (from 27 fewer to 205 fewer)		

CI: Confidence interval; **RR:** Risk ratio

1. It was impossible to blind patients or caregivers in RCTs evaluating NPPV, and lack of blinding was not considered as a factor of downgrading because of a property of mortality as an outcome. In some RCTs, allocation concealment and selective outcome reporting were 'unclear', however we decided not to downgrade.
2. Variance of the point estimates across studies was not significant. According to I2, heterogeneity was considered to be low (I2 = 0%).
3. Subjects were patients with hypoxemia, not ARDS.
4. RR 0.65 [0.46-0.93]. Criteria of the optimal information size (OIS) were not met.

Evidence-to-Dicision table

CQ2: Should non-invasive positive pressure ventilation (NPPV) be used as early respiratory management in adult patients with ARDS?

PATIENTS: ADULT PATIENTS WITH HYPOXEMIA

INTERVENTION: NON-INVASIVE POSITIVE PRESSURE VENTIOATION (NPPV)

CRITERIA		JUDGEMENTS	RESEARCH EVIDENCE	ADDITIONAL CONSIDERATIONS
PROBLEM	**Is the problem a priority?**	◯No<br>◯Probably no<br>◯Probably yes<br>●Yes<br>------------------<br>◯Varies<br>◯Don't know	Although non-invasive positive pressure ventilation (NPPV) is used for treating hypoxia worldwide, its efficacy for ARDS patients has not been understood thoroughly. It is shown that there is a possibility that the use of NPPV leads to decreasing the number of intubation and mortality among ARDS patients, hence, its priority in clinical use is high.	
BENEFITS & HARMS OF THE OPTIONS	What is the overall certainty of the evidence of effects?	◯Very low<br>●Low<br>◯Moderate<br>◯High<br>------------------<br>◯No included studies	See "The relative importance or values of the main outcomes of interest" below	
	Is there important uncertainty about or variability in how much people value the main outcomes?	◯Important uncertainty or variability<br>◯Possibly important uncertainty or variability<br>◯Possibly no important uncertainty or variability<br>●No important uncertainty or variability<br>------------------<br>◯No known undesirable outcomes	See "Oxygen therapy vs. NPPV" below	
	**How substantial are the desirable anticipated effects?**	◯Trivial<br>◯Small<br>●Moderate<br>◯Large<br>------------------<br>◯Varies<br>◯Don't know		
	**How substantial are the undesirable anticipated effects?**	◯Large<br>◯Moderate<br>●Small<br>◯Trivial<br>------------------<br>◯Varies<br>◯Don't know		
	**Does the balance between desirable effects and undesirable effects favour the option or the comparison ?**	◯Favors the comparison<br>◯Probably favors the comparison<br>◯Does not favor either the intervention or the comparison		

The relative importance or values of the main outcomes of interest:

Outcome	Relative importance	Certainty of the evidence (GRADE)
Short term Mortality Note 1)	CRITICAL	⊕⊕◯◯<br>LOW
Intubation	IMPORTANT	⊕⊕◯◯<br>LOW

Oxygen therapy vs. NPPV

Summary of findings:

Outcome	Oxygen therapy	NPPV	Difference (95% CI)	Relative effect (RR) (95% CI)
Short term Mortality Note 1)	365 / 1000	259 / 1000 (197 to 336)	106 fewer per 1,000 (from 29 fewer to 168 fewer)	RR 0.71 (0.54 to 0.92)
	133 / 1000	94 / 1000 (72 to 122)	39 fewer per 1,000 (from 11 fewer to 61 fewer)	
	750 / 1000	533 / 1000 (405 to 690)	218 fewer per 1,000 (from 60 fewer to 345 fewer)	
Intubation	389 / 1000	226 / 1000 (179 to 288)	163 fewer per 1,000 (from 101 fewer to 210 fewer)	RR 0.58 (0.46 to 0.74)
	211 / 1000	122 / 1000 (97 to 156)	89 fewer per 1,000 (from 55 fewer to 114 fewer)	
	733 / 1000	425 / 1000 (337 to 542)	308 fewer per 1,000 (from 191 fewer to 396 fewer)	

Summary: In 17 RCTs comparing NPPV to oxygen therapy in patients with hypoxemia, NPPV significantly reduced the mortality (RR 0.71, 95%CI 0.54-0.92). In 16 RCTs comparing NPPV to oxygen therapy in patients with hypoxemia, NPPV significantly reduced the intubation (RR 0.58, 95%CI 0.46-0.74).

<table>
<tr><td></td><td></td><td>●Probably favors the intervention
〇Favors the intervention

〇Varies
〇Don't know</td><td>Conventional mechanical ventilation vs. NPPV
Summary of findings:
<table><tr><td>Outcome</td><td>Oxygen therapy</td><td>NPPV</td><td>Difference (95% CI)</td><td>Relative effect (RR) (95% CI)</td></tr><tr><td rowspan="2">Mortality (Short term)*1</td><td>209 / 1,000</td><td>136 / 1,000 (96 to 194)</td><td>73 fewer per 1,000 (from 15 fewer to 113 fewer)</td><td rowspan="2">RR 0.65 (0.46 to 0.93)</td></tr><tr><td>379 / 1,000</td><td>246 / 1,000 (174 to 352)</td><td>133 fewer per 1,000 (from 27 fewer to 205 fewer)</td></tr></table>Summary: In 4 RCTs comparing NPPV to conventional mechanical ventilation, NPPV significantly reduced the mortality (RR 0.65, 95%CI 0.46-0.93).</td><td></td></tr>
<tr><td rowspan="2">資源利用
RESOURCE USE</td><td>How large are the resource requirements (costs)?</td><td>〇Large costs
●Moderate costs
〇Negligible costs and savings
〇Moderate savings
〇Large savings

〇Varies
〇Don't know</td><td>For NPPV application, costs for mechanical ventilator with NPPV mode or one specialized to NPPV, interface of NPPV, amount of oxygen required and training for medical staffs are incured.</td><td></td></tr>
<tr><td>Does the cost effectiveness of the option favour the option or the comparison?</td><td>〇Favors the comparison
〇Probably favors the comparison
●Does not favor either the intervention or the comparison
〇Probably favors the intervention
〇Favors the intervention

〇Varies
〇No included studies</td><td>It cannot be determined that the net benefit of NPPV outweighs the cost or resources in patients with ARDS. Disposable masks for NPPV are quite costly.</td><td></td></tr>
<tr><td>EQUITY</td><td>What would be the impact on health equity?</td><td>〇Reduced
〇Probably reduced
〇Probably no impact
〇Probably increased
〇Increased

〇Varies
●Don't know</td><td>There is no data for evaluating the equity although NPPV is more available in hospitals with more experience of NPPV.</td><td></td></tr>
</table>

**ACCEPTABILITY**	Is the option acceptable to key stakeholders?	◯No<br>◯Probably no<br>●Probably yes<br>◯Yes<br>------------------<br>◯Varies<br>◯Don't know	NPPV has been used broadly in Japan and therefore can be expected to be readily accepted.	
**FEASIBILITY**	**Is the option feasible to implement?**	◯No<br>◯Probably no<br>◯Probably yes<br>●Yes<br>------------------<br>◯Varies<br>◯Don't know	NPPV has become a treatment that can be used in many hospitals in Japan already.	

Note 1) Among the deaths in hospital or in ICU

Recommendation

CQ2: **Should non-invasive positive pressure ventilation (NPPV) be used as early respiratory management in adult patients with ARDS?**					
**Balance of consequences**	Undesirable consequences *clearly outweigh* desirable consequences in most settings	Undesirable consequences *probably outweigh* desirable consequences in most settings	The balance between desirable and undesirable consequences is closely *balanced or uncertain*	Desirable consequences *probably outweigh* undesirable consequences in most settings	Desirable consequences *clearly outweigh* undesirable consequences in most settings
Judgement	○	○	○	●	○

**Type of recommendation**	Strong recommendation against the intervention	Conditional recommendation against the intervention	Conditional recommendation for either the intervention or the comparison	Conditional recommendation for the intervention
Judgement	○	○	●	○
**Recommendation**	**We suggest using NPPV as early respiratory management in adults with ARDS. (GRADE 2C, Strength of recommendation "weak recommendation" / Quality of evidence: "low")**<br><br>Supplementary conditions: Monitor the patient for clinical improvement within 1-2 hours of NPPV application. Furthermore, confirm whether the patient's respiratory status meets a predefined goal set in prior to NPPV application within 4-6 hours. When the patient is not clinically improving within 1-2 hours nor achieving the goal within 4-6 hours, the patient should be intubated.			
**Justification**	**Question:** Should non-invasive positive pressure ventilation (NPPV) be used as early respiratory management in adult patients with ARDS?<br>**Patients:** Adult Patients with hypoxemia<br>**Interventions:** NPPV<br>**Comparison:** Oxygen therapy, comventional mechanical ventilation<br>**Outcomes:** Short-term mortality Note 1), intubation<br><br>**Summary of the evidence:** Since it was anticipated that the number of studies concerning the efficacy of early NPPV usage among ARDS patients would be small, we searched for comparative RCTs dealing with the efficacy of NPPV among hypoxemic patients. The choice of whether using oxygen therapy or conventional mechanical ventilation depends primarily on the severity of hypoxemia, hence, in our systematic review, we compared the efficacy of NPPV and that of oxygen therapy, and also, the efficacy of NPPV and that of conventional mechanical ventilation. Moreover, we excluded studies involving patients with COPD or congestive heart failure. This was because it was expected that these studies would strongly support the efficacy of NPPV. As a result, a total of 21 studies were obtained. Among these studies, 17 compared the efficacy of NPPV and that of oxygen therapy and 21 compared the efficacy of NPPV and that of conventional mechanical ventilation.<br>In 17 RCTs comparing NPPV to oxygen therapy, NPPV significantly reduced the mortality (RR 0.71, 95%CI 0.54-0.92). In 16 RCTs comparing NPPV to oxygen therapy, NPPV significantly reduced the intubation (RR 0.58, 95%CI 0.46-0.74). In 4 RCTs comparing NPPV to conventional mechanical ventilation, NPPV significantly reduced the mortality (RR 0.65, 95%CI 0.46-0.93).<br><br>**Quality of the evidence:** In 17 RCTs comparing NPPV to oxygen therapy, the risk of bias for mortality was 'not serious' and the risk of bias for intubation was downgraded by one level and classified as 'serious' because open-label fashion seemed to affect on decision of intubation. Inconsistency of results for mortality was downgraded as 'serious' since wide variance of point estimates across studies was found and heterogeneity for mortality was moderate (I^2=44%). However, in terms of intubation, heterogeneity was low (I^2=25%) and variance of point estimates across studies was not significant, and so inconsistency of results was 'not serious'. Indirectness was considered as 'serious' in both outcomes, mortality and intubation, because of the unmatched study subjects, as subjects included in selected RCTs had hypoxemia, not ARDS. The level of imprecision was 'not serious' for mortality and intubation since criteria of the optimal information size (OIS) were met. Based on the above discussion, the overall quality of evidence was evaluated as 'low'.<br>In 4 RCTs comparing NPPV to conventional mechanical ventilation, the risk of bias for mortality			

	was 'not serious'. Inconsistency of results for mortality was 'not serious' since heterogeneity was low (I^2=0%) and variance of point estimates across studies was not significant. Indirectness was considered as 'not serious' although all subjects included in selected RCTs didn't meet criteria of ARDS. The level of imprecision for mortality was 'serious' since criteria of the OIS were not met. Based on the above discussion, the overall quality of evidence was evaluated as 'low'.<br><br>**Judgement of benefit and harm, resources and cost:** In spite that NPPV has become a treatment that can be used in many hospitals in Japan, cost of NPPV may be higher than that of oxygen therapy or intubation due to price of mechanical ventilator with NPPV mode or specialized to NPPV, interface of NPPV, amount of oxygen required, cost of training for medical staffs, cost of hiring related staffs and so on. On the other hand, there is a possibility that the cost of NPPV may be lower due to avoidance of intubation. Therefore, it cannot be determined that the benefits of NPPV outweigh the harms in patients with ARDS.<br><br>**Recommendations;** We suggest using NPPV as early respiratory management in adults with ARDS. (GRADE 2C, Strength of recommendation "weak recommendation" / Quality of evidence: "low")<br><br>**Additional considerations:** Applying NPPV to hypoxemic patients, outcome may vary depending on skill and experience of NPPV amang medical staffs. It is suggested that delayed intubateion relates to mortality; thus criteria of intubateion should be predefined applying NPPV. In addition, most RCTs evaluating benefit of NPPV exclude unconscious patients and hemodynamically unstable patients, hence, applying this recommendation requires cautiousness to such populations.[1]<br>At panel meeting, we discussed the validity of wording and eventually we adopted usage of the word "NPPV" instead of non-invasive ventilation (NIV) and "early respiratory management in adults with ARDS" instead of "respiratory management in adults with mild ARDS." And in discussion, we decided to add the comment about need predefining criteria of intubation to the recommendation.
**Subgroup considerations**	In RCTs comparing NPPV to oxygen therapy, even when excluding Gupta 2010, in which only patients with asthma attack were included, we obtained similar results (mortality (RR 0.71, 95%CI 0.54-0.92)、intubation (RR 0.58, 95%CI 0.46-0.73)) to evaluation of whole RCTs.
**Implementation considerations**	
**Monitoring and evaluation considerations**	During NPPV, respiratory status, circulatory status and consciousness and blood gas analysis should be evaluated repeatedly. Monitor the patient for clinical improvement within 1-2 hours of NPPV application. Furthermore, confirm whether the patient's respiratory status meets a predefined goal set in prior to NPPV application within 4-6 hours. When the patient is not clinically improving within 1-2 hours nor achieving the goal within 4-6 hours, the patient should be intubated. One study suggested that delayed intubation is related to higher mortality[2].
**Research possibilities**	More studies to evaluate the efficacy of NPPV for patients with ARDS are needed. Also, efficacy of other non-invasive respiratory managements such as high-flow nasal therapy should be compared to oxygen therapy, conventional mechanical ventilation and NPPV.

Note 1) Mortality in hospital or in ICU.

References

1. Esteban A, Frutos-Vivar F, Ferguson ND, et al. Noninvasive positive-pressure ventilation for respiratory failure after extubation. *The New England journal of medicine* **350**(24): 2452-60, 2004. PMID 15190137
2. Epstein SK, Ciubotaru RL. Independent effects of etiology of failure and time to reintubation on outcome for patients failing extubation. *American journal of respiratory and critical care medicine* **158**(2): 489-93, 1998. PMID 9700126

CQ03. Study flow diagram

*This CQ was partly evaluated by Petrucci using Cochrane database (to Sep 2012)[1)].
We also searched literature from Sep 2011 to May 2015.

1. Petrucci N, De Feo C. Lung protective ventilation strategy for the acute respiratory distress syndrome. Cochrane database of systematic reviews. 2:CD003844 2013, PMID 23450544

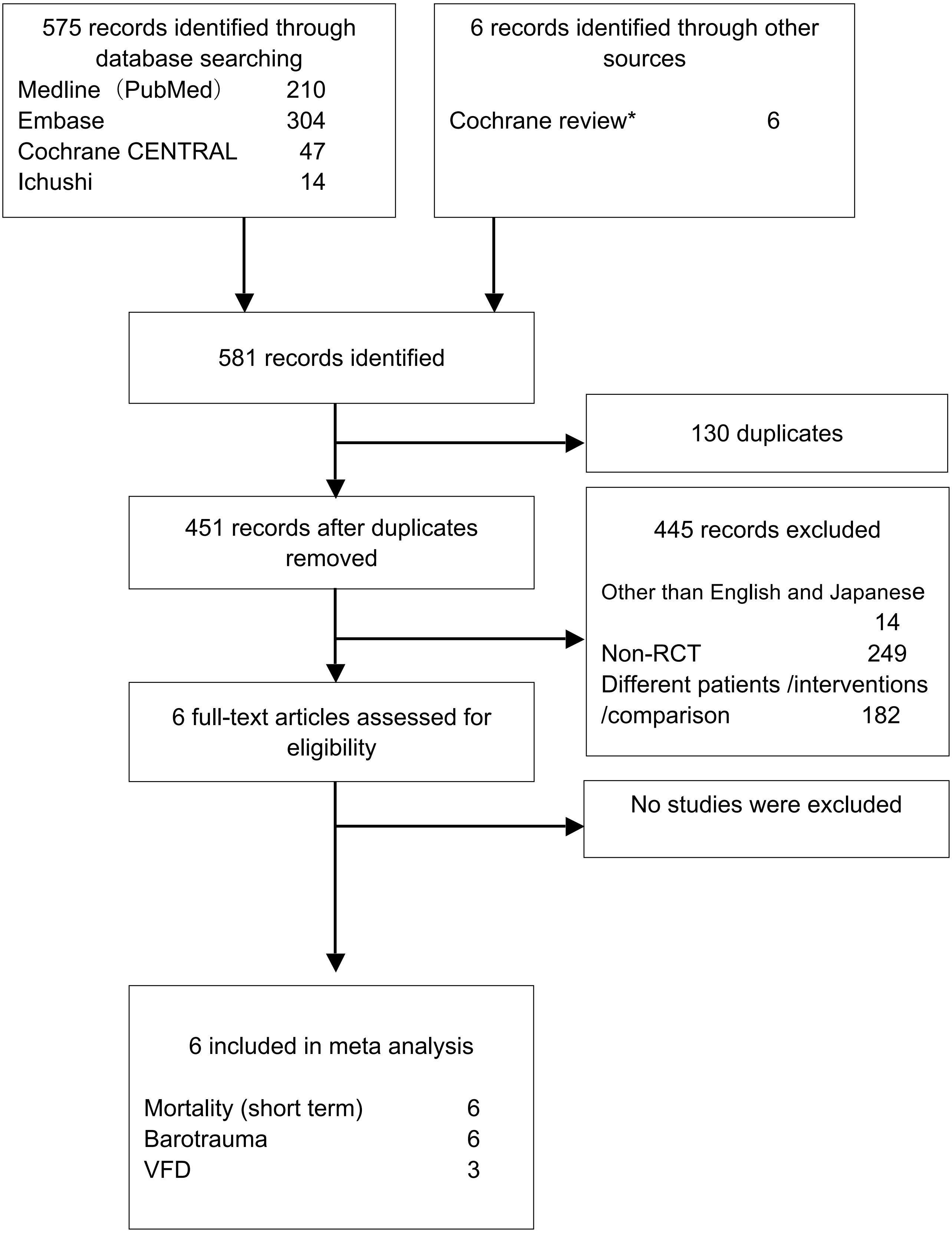

CQ03
Risk of bias table, mortality

Outcome		Short term mortality		risk of bias		not serious (0)			
番号	著者名 発表年（Forest plot表示）	risk of bias評価							
		ランダム割付順番の生成 random sequence generation	割り付けの隠蔽化 allocation concealment	ブラインド blinding 研究参加者と治療提供者 participants and personnel	ブラインド blinding アウトカム評価者 outcome assessors	不完全なアウトカムデータ incomplete outcome data	選択されたアウトカムの報告 selective outcome reporting	その他のバイアス Other sources of bias	研究内でのバイアスのリスク Risk of bias within a study
1	Amato 1998 (in-hospital mortality)	Low risk	Low risk	High risk	Unclear risk	Low risk	Unclear risk	High risk	Unclear risk
2	Brochard 1998 (60days)	Low risk	Low risk	High risk	Unclear risk	Low risk	Unclear risk	Low risk	Low risk
3	Stewart 1998 (in-hospital mortality)	Low risk	Low risk	High risk	Unclear risk	Low risk	Unclear risk	Low risk	Low risk
4	Brower 1999(in-hospital mortality)	Unclear risk	Low risk	High risk	Unclear risk	Low risk	Unclear risk	Low risk	Unclear risk
5	ARDS network 2000 (180days)	Low risk	Low risk	High risk	Unclear risk	Low risk	Low risk	High risk	Unclear risk
6	Villar 2006 (in-hospital mortality)	Low risk	Low risk	High risk	Unclear risk	Low risk	Unclear risk	High risk	Unclear risk

番号	著者名 発表年（Forest plot表示）	risk of biasコメント							
1	Amato 1998	封筒法	隠蔽化されている	換気量の違いをblindすることはできない	Blindに関しては不明だが、結果の評価についてバイアスが生じる可能性は低い	100%報告されている	protocolが不明である	中間解析に基づき、研究が早期中断されている (stopping early for benefits)	high 2項目、Unclear2項目、low2項目でunclearに引き下げ
2	Brochard 1998	封筒法	隠蔽化されている	一回換気量の違いを盲検化することは不可能	Blindに関しては不明だが、結果の評価についてバイアスが生じる可能性は低い	100%報告されている	protocolを参照できない	ただし中間解析で両群に有意差なく早期中止されている	多くはlow riskで総合的にlow riskと判断
3	Stewart 1998	中央コンピューター方式	隠蔽化されている	一回換気量の違いを盲検化することは不可能	Blindに関しては不明だが、結果の評価についてバイアスが生じる可能性は低い	100%報告されている	protocolを参照できない	ただし、sample sizeの計算についての記載がなく、統計学的パワーは不明	多くはlow riskで総合的に判断
4	Brower 1999	不明	隠蔽化されている	一回換気量の違いを盲検化することは不可能	Blindに関しては不明だが、結果の評価についてバイアスが生じる可能性は低い	100%報告されている	protocolを参照できない	ただし、中間解析に基づき、有意差なしとして研究が早期中止されている	low3項目、unclear3項目で下方修正
5	ARDS network 2000	センターでの音声システムを使用した	隠蔽化されている	tidal volumeの違いを盲検化することは不可能	Blindはされているか不明。しかし結果の評価についてバイアスが生じる可能性は低い	なし	主要なアウトカムの全てが報告されている。ただし、28日死亡は文献の生存曲線より得たものである。	中間解析に基づき、研究が早期中断されている (stopping early for benefits).	High risk 2項目でunclear riskに引き下げ
6	Villar 2006	封筒法	隠蔽化されている	一回換気量の違いを盲検化することは不可能	Blindに関しては不明だが、結果の評価についてバイアスが生じる可能性は低い	100%報告されている	protocolを参照できない	中間解析に基づき、研究が早期中断されている (stopping early for benefits)	high2項目, uncler2項目でunclear riskに引き下げ

CQ03

Risk of bias table, barotrauma

Outcome		Barotrauma		risk of bias		serious (−1)			
番号	著者名 発表年<br>（Forest plot表示）	risk of bias評価							
		ランダム割付順番の生成<br>random sequence generation	割り付けの隠蔽化<br>allocation concealment	ブラインド blinding<br>研究参加者と治療提供者<br>participants and personnel	ブラインド blinding<br>アウトカム評価者<br>outcome assessors	不完全なアウトカムデータ<br>incomplete outcome data	選択されたアウトカムの報告<br>selective outcome reporting	その他のバイアス<br>Other sources of bias	研究内でのバイアスのリスク<br>Risk of bias within a study
1	Amato 1998	Low risk	Low risk	High risk	Unclear risk	Low risk	Unclear risk	High risk	Unclear risk
2	Brochard 1998	Low risk	Low risk	High risk	Unclear risk	Low risk	Unclear risk	Low risk	Low risk
3	Stewart 1998	Low risk	Low risk	High risk	Unclear risk	Low risk	Unclear risk	Low risk	Low risk
4	Brower 1999	Unclear risk	Low risk	High risk	Unclear risk	Low risk	Unclear risk	Low risk	Unclear risk
5	ARDS network 2000	Low risk	Low risk	High risk	Unclear risk	Low risk	Low risk	High risk	Unclear risk
6	Villar 2006	Low risk	Low risk	High risk	Unclear risk	High risk	Unclear risk	High risk	High risk

番号	著者名 発表年<br>（Forest plot表示）	risk of biasコメント							
1	Amato 1998	封筒法	隠蔽化されている	換気量の違いをblindすることはできない	Blindに関しては不明だが、結果の評価についてバイアスが生じる可能性は低い	100%報告されている	protocolが不明である	中間解析に基づき、研究が早期中断されている（stopping early for benefits）	high 2項目、Unclear2項目、low2項目でunclearに引き下げ
2	Brochard 1998	封筒法	隠蔽化されている	一回換気量の違いを盲検化することは不可能	Blindに関しては不明だが、結果の評価についてバイアスが生じる可能性は低い	100%報告されている	protocolを参照できない	ただし中間解析で両群に有意差なく早期中止されている	多くはlow riskで総合的にlow riskと判断
3	Stewart 1998	中央コンピューター方式	隠蔽化されている	一回換気量の違いを盲検化することは不可能	Blindに関しては不明だが、結果の評価についてバイアスが生じる可能性は低い	100%報告されている	protocolを参照できない	ただし、sample sizeの計算についての記載がなく、統計学的パワーは不明	多くはlow riskで総合的に判断
4	Brower 1999	不明	隠蔽化されている	一回換気量の違いを盲検化することは不可能	Blindに関しては不明だが、結果の評価についてバイアスが生じる可能性は低い	100%報告されている	protocolを参照できない	ただし、中間解析に基づき、有意差なしとして研究が早期中止されている	low3項目、unclear3項目で下方修正
5	ARDS network 2000	センターでの音声システムを使用した	隠蔽化されている	一回換気量の違いを盲検化することは不可能	Blindはされているか不明。しかし結果の評価についてバイアスが生じる可能性は低い	なし	主要なアウトカムの全てが報告されている	中間解析に基づき、研究が早期中断されている（stopping early for benefits）	High risk 2項目でunclear riskに引き下げ
6	Villar 2006	封筒法	隠蔽化されている	一回換気量の違いを盲検化することは不可能	Blindに関しては不明だが、結果の評価についてバイアスが生じる可能性は低い	protocol逸脱が103人中8人いて、解析から除外されている。ITT解析でない	protocolを参照できない	中間解析に基づき、研究が早期中断されている（stopping early for benefits）	high3項目, uncler2項目でHigh riskに引き下げ

CQ03
Risk of bias table VFD

Outcome		VFD		risk of bias		serious (−1)			
番号	著者名 発表年<br>（Forest plot表示）	risk of bias評価							
		ランダム割付順番の生成<br>random sequence generation	割り付けの隠蔽化<br>allocation concealment	ブラインド blinding<br>研究参加者と治療提供者<br>participants and personnel	ブラインド blinding<br>アウトカム評価者<br>outcome assessors	不完全なアウトカムデータ<br>incomplete outcome data	選択されたアウトカムの報告<br>selective outcome reporting	その他のバイアス<br>Other sources of bias	研究内でのバイアスのリスク<br>Risk of bias within a study
1	Brower 1999	Unclear risk	Low risk	High risk	Unclear risk	Low risk	Unclear risk	Low risk	Unclear risk
2	ARDS network 2000	Low risk	Low risk	High risk	Unclear risk	Low risk	Low risk	High risk	Unclear risk
3	Villar 2006	Low risk	Low risk	High risk	Unclear risk	High risk	Unclear risk	High risk	High risk
番号	著者名 発表年<br>（Forest plot表示）	risk of biasコメント							
1	Brower 1999	不明	隠蔽化されている	一回換気量の違いを盲検化することは不可能	Blindに関しては不明だが、結果の評価についてバイアスが生じる可能性は低い	100%報告されている	protocolを参照できない VFD28daysは著者に問い合わせて得られたデータである。Unpublished data	ただし、中間解析に基づき、有意差なしとして研究が早期中止されている	low3項目、unclear3項目で下方修正
2	ARDS network 2000	センターでの音声システムを使用した	隠蔽化されている	一回換気量の違いを盲検化することは不可能	Blindはされているか不明。しかし結果の評価についてバイアスが生じる可能性は低い	なし	主要なアウトカムの全てが報告されている	中間解析に基づき、研究が早期中断されている (stopping early for benefits)	High risk 2項目でunclear riskに引き下げ
3	Villar 2006	封筒法	隠蔽化されている	一回換気量の違いを盲検化することは不可能	Blindに関しては不明だが、結果の評価についてバイアスが生じる可能性は低い	protocol逸脱が103人中8人いて、解析から除外されている。ITT解析でない	protocolを参照できない	中間解析に基づき、研究が早期中断されている (stopping early for benefits)	high3項目, uncler2項目でHigh riskに引き下げ

CQ03 Risk of bias summary, Risk of bias graph

Short term mortality

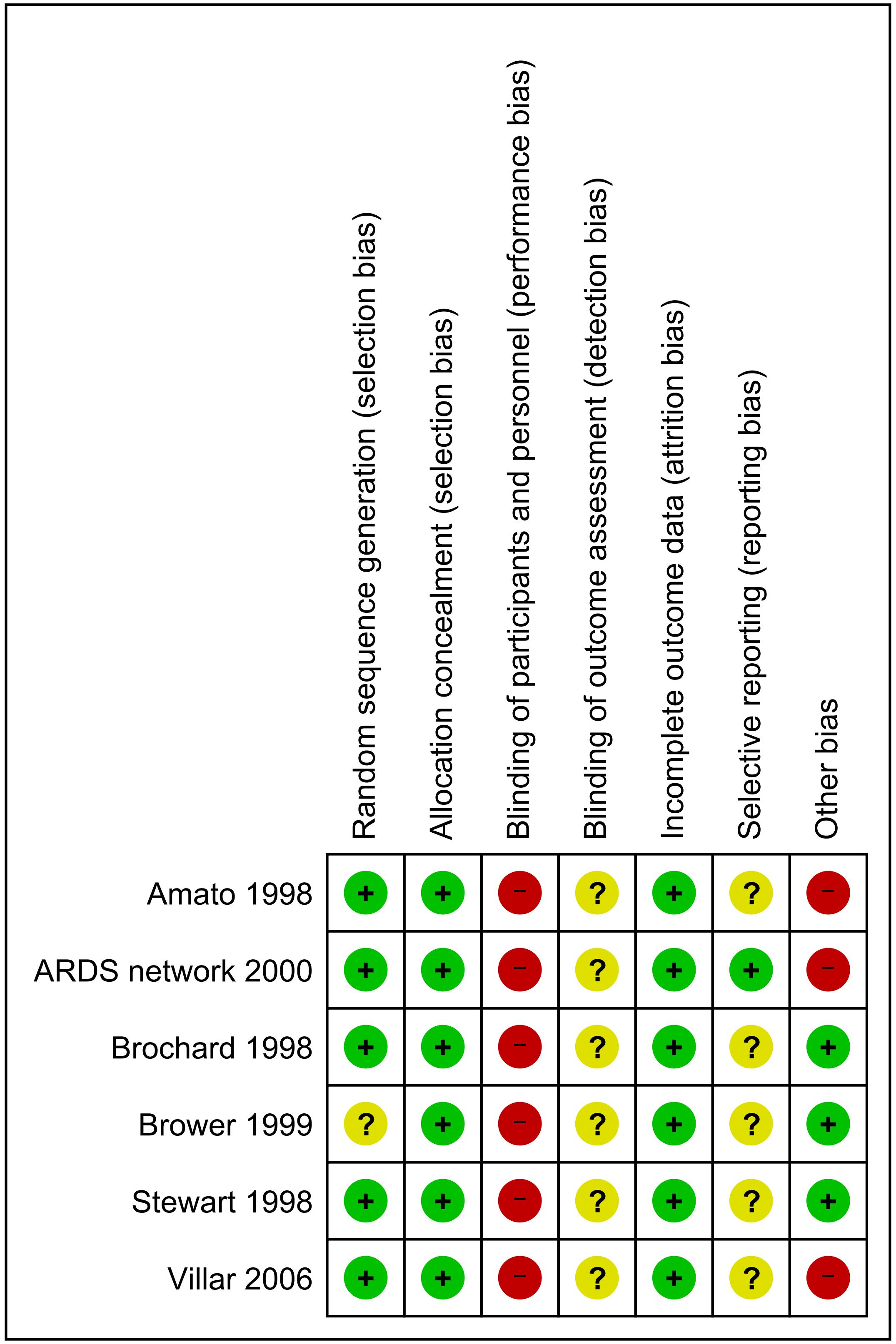

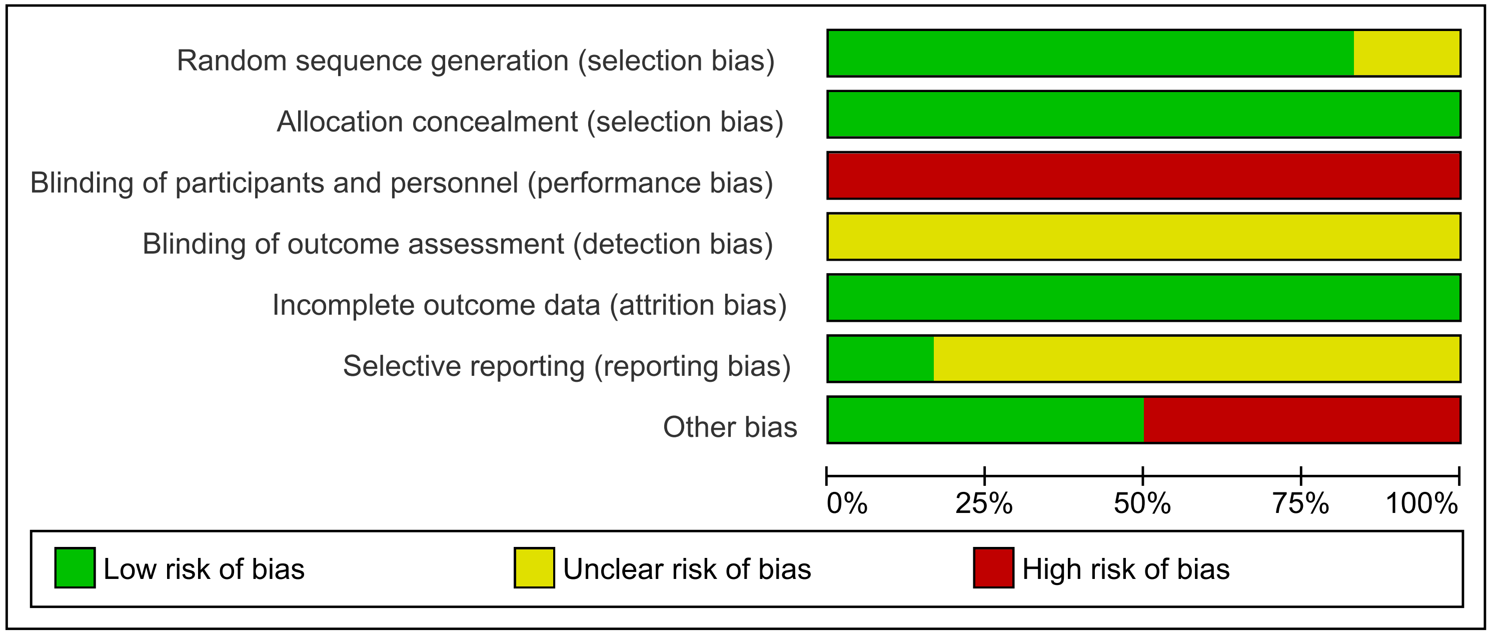

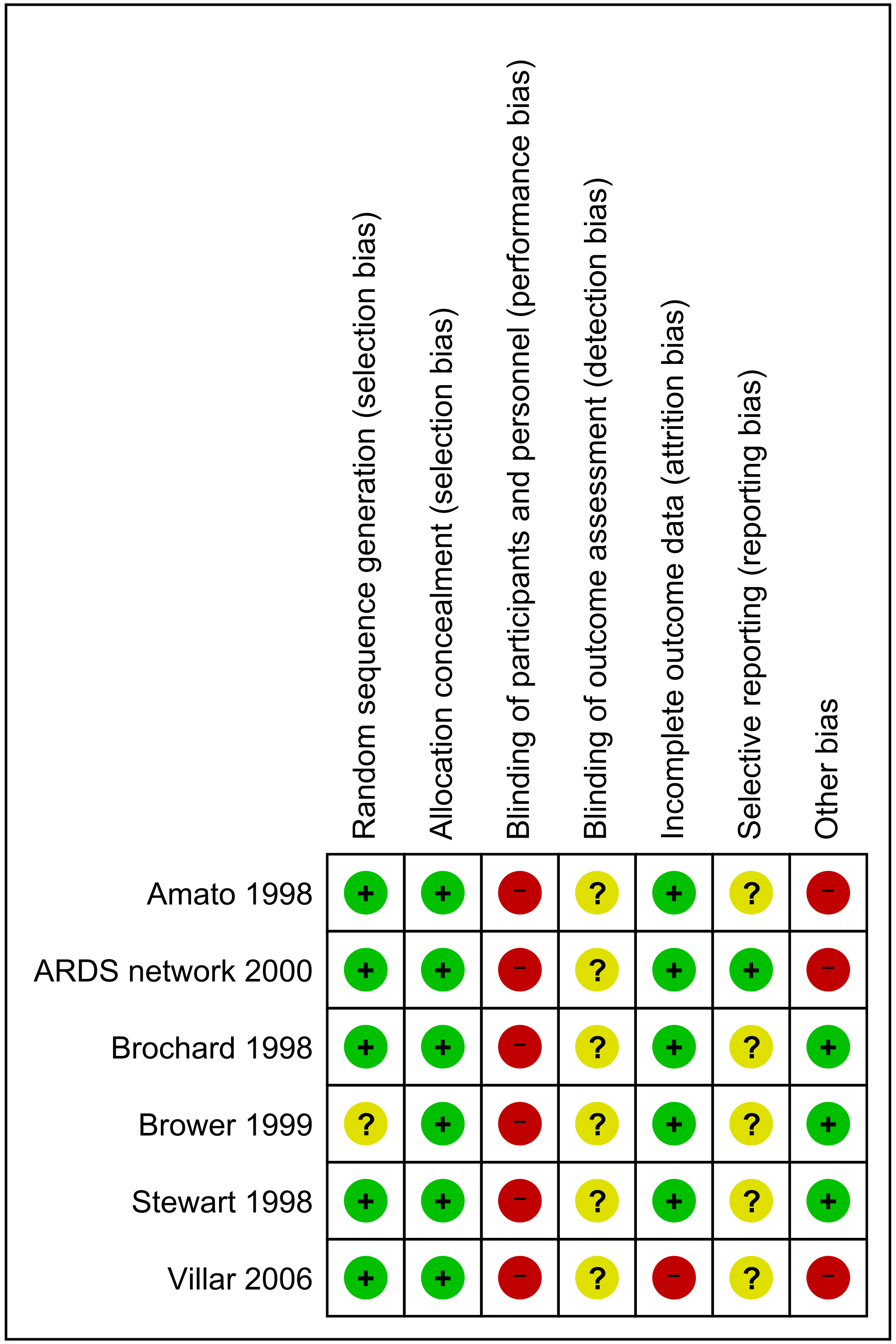
Random sequence generation (selection bias)
Allocation concealment (selection bias)
Blinding of participants and personnel (performance bias)
Blinding of outcome assessment (detection bias)
Incomplete outcome data (attrition bias)
Selective reporting (reporting bias)
Other bias
Amato 1998
ARDS network 2000
Brochard 1998
Brower 1999
Stewart 1998
Villar 2006

Barotrauma

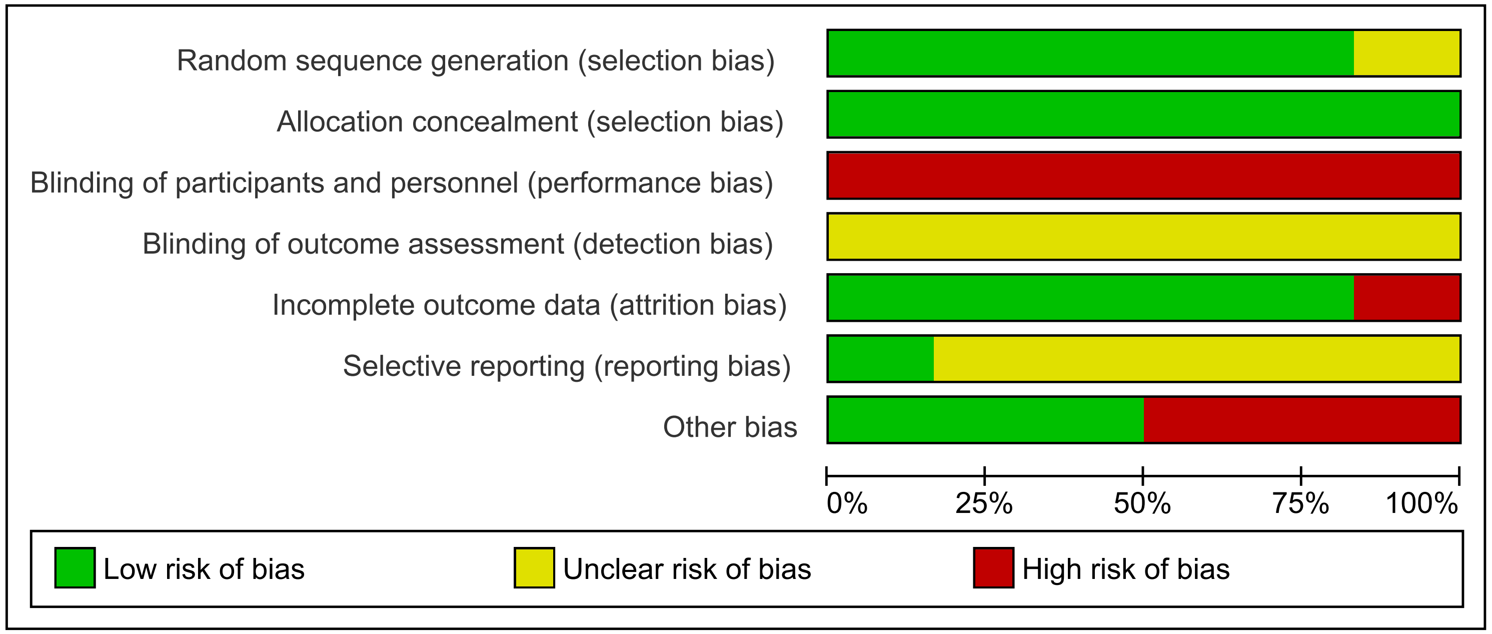
Random sequence generation (selection bias)
Allocation concealment (selection bias)
Blinding of participants and personnel (performance bias)
Blinding of outcome assessment (detection bias)
Incomplete outcome data (attrition bias)
Selective reporting (reporting bias)
Other bias
0%
25%
50%
75%
100%
Low risk of bias
Unclear risk of bias
High risk of bias

VFD

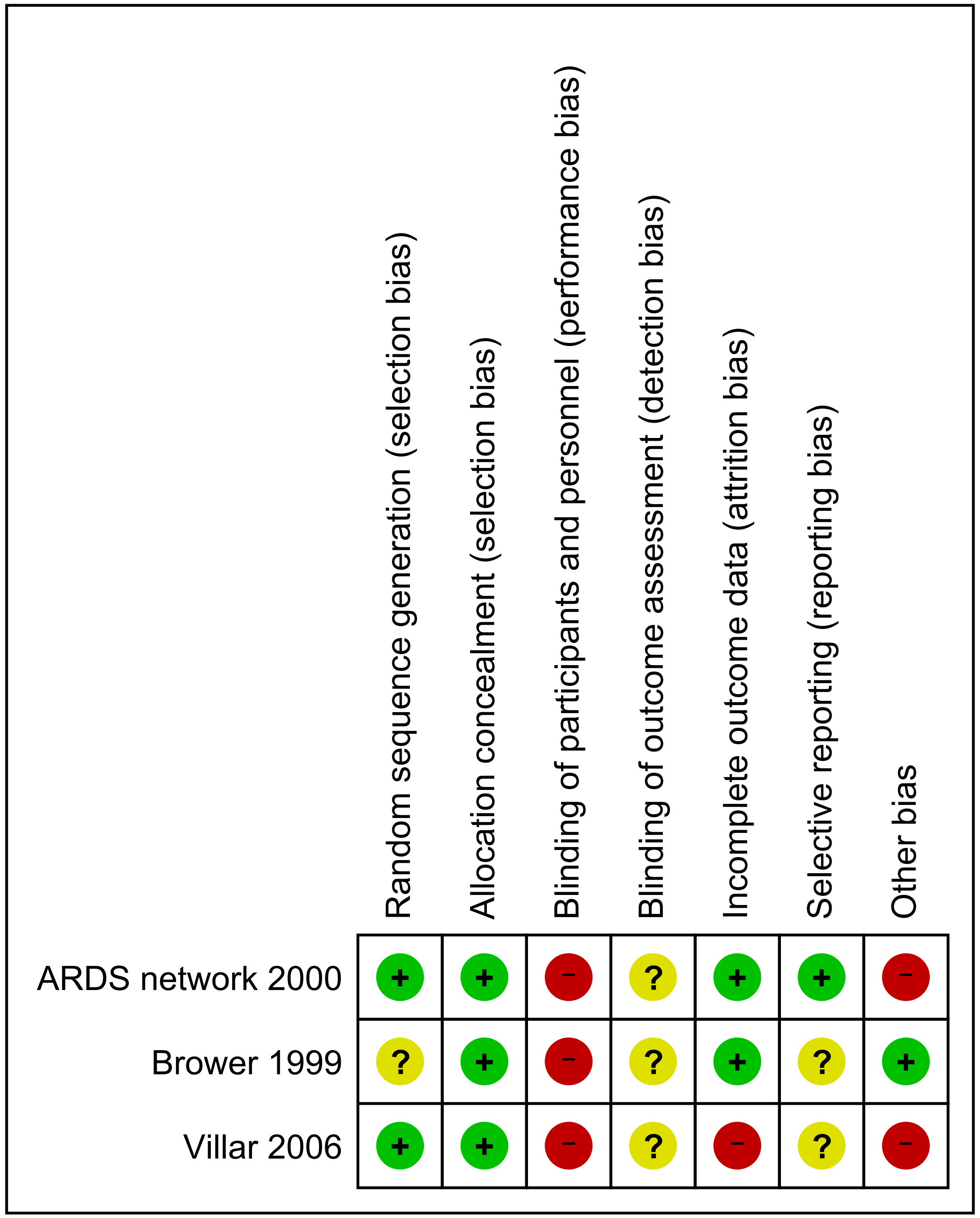
Random sequence generation (selection bias)
Allocation concealment (selection bias)
Blinding of participants and personnel (performance bias)
Blinding of outcome assessment (detection bias)
Incomplete outcome data (attrition bias)
Selective reporting (reporting bias)
Other bias
ARDS network 2000
Brower 1999
Villar 2006

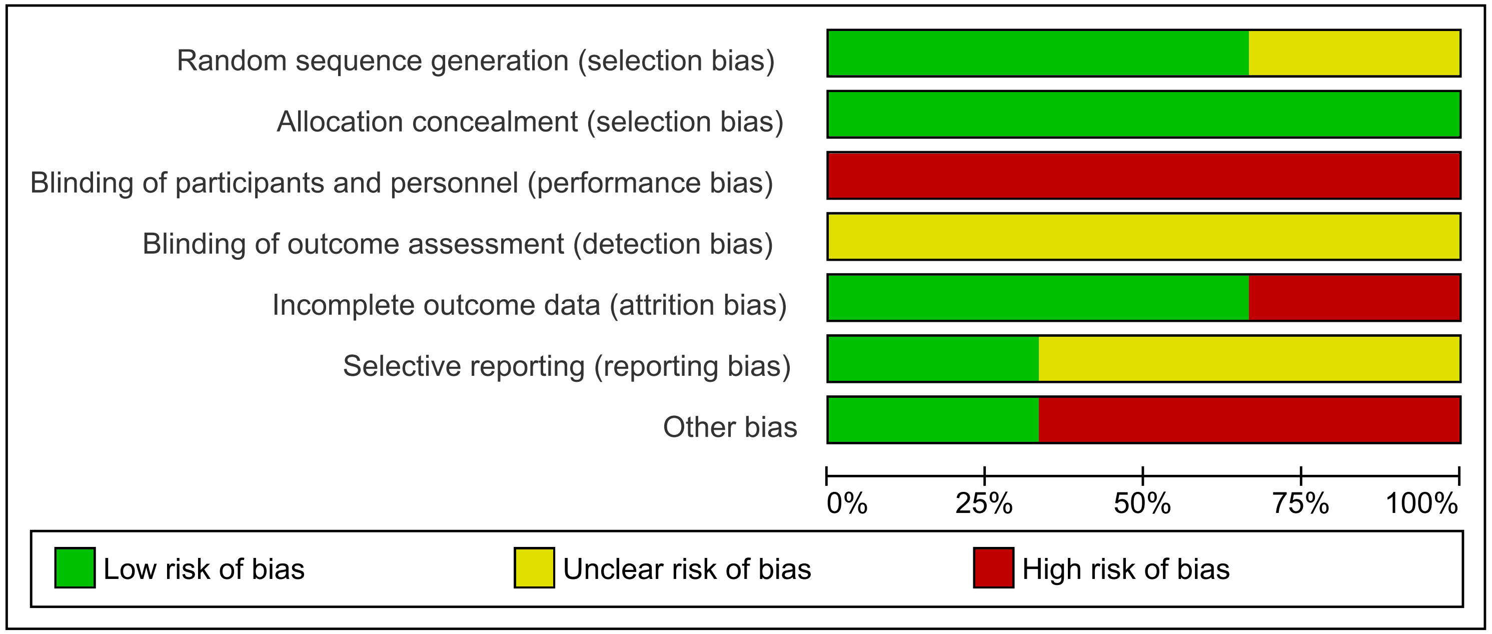
Random sequence generation (selection bias)
Allocation concealment (selection bias)
Blinding of participants and personnel (performance bias)
Blinding of outcome assessment (detection bias)
Incomplete outcome data (attrition bias)
Selective reporting (reporting bias)
Other bias
0%
25%
50%
75%
100%
Low risk of bias
Unclear risk of bias
High risk of bias

Short term mortality

Study or Subgroup	lower tidal volume Events	lower tidal volume Total	conventional tidal volume Events	conventional tidal volume Total	Weight	Risk Ratio M-H, Random, 95% CI
Amato 1998	13	29	17	24	13.9%	0.63 [0.39, 1.02]
ARDS network 2000	112	432	150	429	26.4%	0.74 [0.60, 0.91]
Brochard 1998	27	58	22	58	15.7%	1.23 [0.80, 1.89]
Brower 1999	13	26	12	26	11.3%	1.08 [0.62, 1.91]
Stewart 1998	30	60	28	60	18.1%	1.07 [0.74, 1.55]
Villar 2006	17	53	28	50	14.5%	0.57 [0.36, 0.91]
**Total (95% CI)**		**658**		**647**	**100.0%**	**0.84 [0.67, 1.07]**
Total events	212		257			

Heterogeneity: Tau² = 0.04; Chi² = 10.71, df = 5 (P = 0.06); I² = 53%
Test for overall effect: Z = 1.42 (P = 0.16)

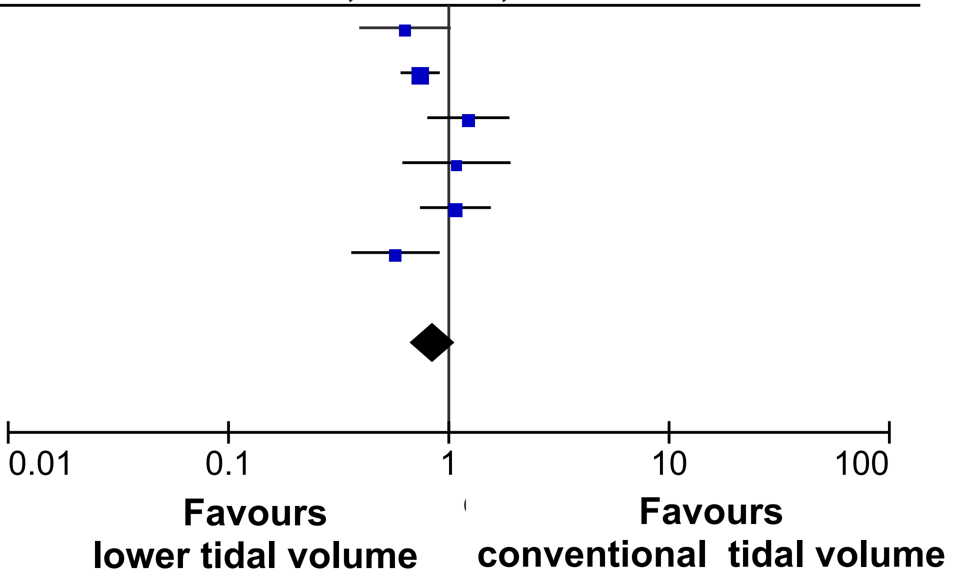

Barotrauma

Study or Subgroup	lower tidal volume Events	lower tidal volume Total	conventional tidal volume Events	conventional tidal volume Total	Weight	Risk Ratio M-H, Random, 95% CI
Amato 1998	2	29	10	24	11.3%	0.17 [0.04, 0.68]
ARDS network 2000	43	432	47	429	40.9%	0.91 [0.61, 1.34]
Brochard 1998	8	58	7	58	19.9%	1.14 [0.44, 2.95]
Brower 1999	2	26	1	26	4.8%	2.00 [0.19, 20.72]
Stewart 1998	6	60	4	60	14.3%	1.50 [0.45, 5.05]
Villar 2006	2	50	4	45	8.8%	0.45 [0.09, 2.34]
**Total (95% CI)**		**655**		**642**	**100.0%**	**0.82 [0.48, 1.41]**
Total events	63		73			

Heterogeneity: Tau² = 0.14; Chi² = 7.55, df = 5 (P = 0.18); I² = 34%
Test for overall effect: Z = 0.71 (P = 0.48)

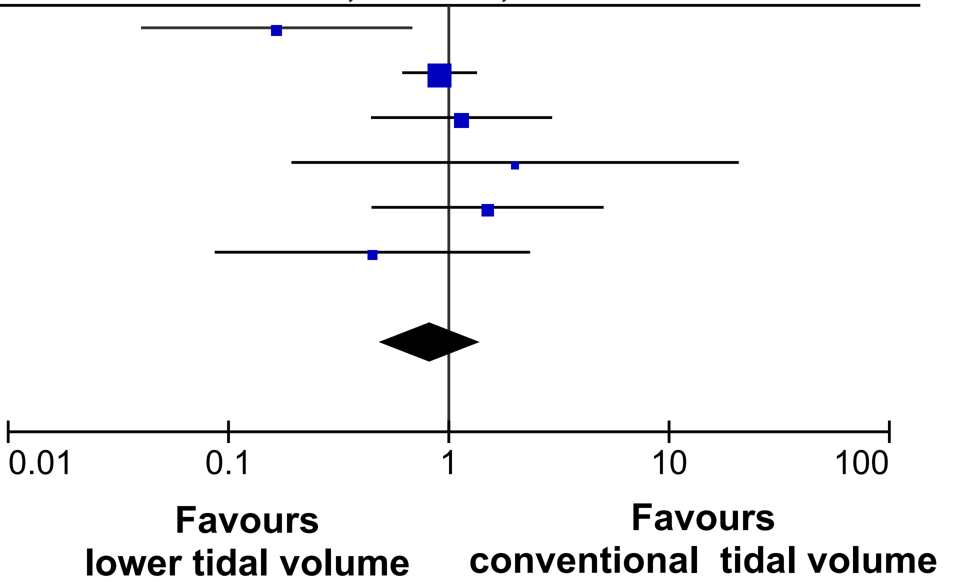

VFD

Study or Subgroup	lower tidal volume Mean	lower tidal volume SD	lower tidal volume Total	conventional tidal volume Mean	conventional tidal volume SD	conventional tidal volume Total	Weight	Mean Difference IV, Random, 95% CI
ARDS network 2000	12	11	432	10	11	429	63.8%	2.00 [0.53, 3.47]
Brower 1999	9.62	10.3	26	9.27	9.77	26	11.7%	0.35 [-5.11, 5.81]
Villar 2006	10.9	9.4	50	6	7.9	45	24.5%	4.90 [1.42, 8.38]
**Total (95% CI)**			**508**			**500**	**100.0%**	**2.52 [0.53, 4.51]**

Heterogeneity: Tau² = 1.06; Chi² = 2.79, df = 2 (P = 0.25); I² = 28%
Test for overall effect: Z = 2.48 (P = 0.01)

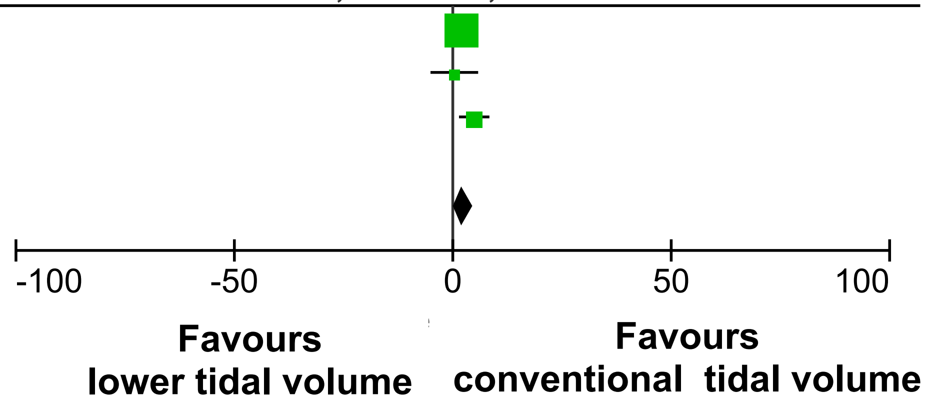

Summary of findings:

CQ03：Should low tidal volume be used in adult patients with ARDS?

Patient or population: [health problem]
Setting:
Intervention: lower tidal volume
Comparison: conventional tidal volume

Outcomes	Anticipated absolute effects* (95% CI)		Relative effect (95% CI)	№ of participants (studies)	Quality of the evidence (GRADE)	Comments
	**Risk with conventional tidal volume**	**Risk with lower tidal volume**				
mortality	**Study population**		**RR 0.84** (0.67 to 1.07)	1305 (6 RCTs)	⊕⊕◯◯ LOW [1,2,5]	
	397 per 1000	**334 per 1000** (266 to 425)				
	**Low**					
	380 per 1000	**319 per 1000** (255 to 407)				
	**High**					
	560 per 1000	**470 per 1000** (375 to 599)				
barotrauma	**Study population**		**RR 0.82** (0.48 to 1.41)	1297 (6 RCTs)	⊕◯◯◯ VERY LOW [2,3,4,5]	
	114 per 1000	**93 per 1000** (55 to 160)				
	**Low**					
	38 per 1000	**31 per 1000** (18 to 54)				
	**High**					
	120 per 1000	**98 per 1000** (58 to 169)				
VFD	Mean **8.93 days**	2.52 days more MD (0.53 more to 4.51 more)	-	1008 (3 RCTs)	⊕⊕⊕◯ MODERATE [3]	

***The risk in the intervention group** (and its 95% confidence interval) is based on the assumed risk in the comparison group and the **relative effect** of the intervention (and its 95% CI).

CI: Confidence interval; **RR:** Risk ratio; **MD:** Mean difference

GRADE Working Group grades of evidence
High quality: We are very confident that the true effect lies close to that of the estimate of the effect
Moderate quality: We are moderately confident in the effect estimate: The true effect is likely to be close to the estimate of the effect, but there is a possibility that it is substantially different
Low quality: Our confidence in the effect estimate is limited: The true effect may be substantially different from the estimate of the effect
Very low quality: We have very little confidence in the effect estimate: The true effect is likely to be substantially different from the estimate of effect

1. Significant heterogeneity with $I^2=50\%$
2. Different length of follow-up period
3. More than half of studies had unclear or high risk of bias
4. Different definition of barotrauma
5. Wide confidence limits

CQ3

Question: Low tidal volume compared with conventional tidal volume ventilation for adult patients with ARDS

Quality assessment							№ of patients		Effect		Quality	Importance
**№ of studies**	**Study design**	**Risk of bias**	**Inconsistency**	**Indirectness**	**Imprecision**	**Other considerations**	**lower tidal volume**	**conventional tidal volume**	**Relative (95% CI)**	**Absolute (95% CI)**		
Short-term mortality												
6	Randomized trials	Not serious	Serious [1,2]	Not serious	Serious [5]	None	212/658 (32.2%)	257/647 (39.7%)	**RR 0.84** (0.67 to 1.07)	**64 fewer per 1000** (from 28 more to 131 fewer)	⨁⨁⊝⊝ LOW	CRITICAL
								38.0%		**61 fewer per 1000** (from 27 more to 125 fewer)		
								56.0%		**90 fewer per 1000** (from 39 more to 185 fewer)		
barotrauma												
6	randomised trials	serious [3]	serious [2,4]	not serious	serious [5]	none	63/655 (9.6%)	73/642 (11.4%)	**RR 0.82** (0.48 to 1.41)	**20 fewer per 1000** (from 47 more to 59 fewer)	⨁⊝⊝⊝ VERY LOW	CRITICAL
								3.8%		**7 fewer per 1000** (from 16 more to 20 fewer)		
								12.0%		**22 fewer per 1000** (from 49 more to 62 fewer)		
VFD												
3	randomised trials	serious [3]	not serious	not serious	not serious	none	508	500	-	MD **2.52 more** (0.53 more to 4.51 more)	⨁⨁⨁⊝ MODERATE	CRITICAL

CI: Confidence interval; RR: Risk ratio; MD: Mean difference

1. Significant heterogeneity with I^2=50%
2. Different length of follow-up period
3. More than half of studies had unclear or high risk of bias
4. Different definition of barotrauma
5. Wide confidence limits

Evidence-to-Decision table

CQ03 : Should low tidal volume be used in adult patients with ARDS?

PATIENTS : ADULT PATIENTS WITH ARDS

INTERVENTION : LOW TIDAL VOLUME

CRITERIA		JUDGEMENTS	RESERCH EVIDENCE	ADDITIONAL CONSIDERRATIONS
PROBLEM	**Is the problem a priority?**	○No<br>○Probably no<br>○Probably yes<br>●Yes<br>------------------<br>○Varies<br>○Don't know	The strategy for mechanical ventilation is very important in patients with acute respiratory distress syndrome (ARDS), as well as the treatment of the primary disease. In particular, mechanical ventilation settings have the highest priority for patients with ARDS. To reduce further lung injury in patients with ARDS, studies have been conducted to determine the optimal ventilation strategy to limit tidal volume and airway pressure.	
BENEFITS & HARMS OF THE OPTIONS	**What is the overall certainty of the evidence of effects?**	○Very low<br>○Low<br>●Moderate<br>○High<br>------------------<br>○No included studies	**The relative importance or values of the main outcomes of interest:** (see table below)	
	**Is there important uncertainty about or variability in how much people value the main outcomes?**	○Important uncertainty or variability<br>○Possibly important uncertainty or variability<br>●Possibly no important uncertainty or variability<br>○No important uncertainty or variability<br>------------------<br>○No known undesirable outcomes	Summary of findings: (see table below)	
	**How substantial are the desirable anticipated effects?**	○Trivial<br>○Small<br>●Moderate<br>○Large<br>------------------<br>○Varies<br>○Don't know		The ventilation settings used in the low tidal volume and conventional tidal volume groups were from Amato1998: 6 [illegible] 12 mL/kg(actual body weight: ABW), Brochard1998: 6-10 v[illegible] 10-15ml/kg (ABW), Stewart1998: 8 vs. 10-15ml/kg (predicted body weight: PBW), Brower 1999: 5-8 vs. 10-12ml/kg (PBW), ARDS netwok2000: 6 vs. 12ml/kg (PBV[illegible] and Villar 2006: 5 to 8 vs. 9 to 11 mL/kg (PBW), respectivel[illegible] with a range of 5 to 10 mL/kg in the low tidal volume group[illegible] However, the actual ventilation was approximately 6.2 to 7.[illegible] mL/kg in the low tidal volume group and approximately 10 t[illegible] 11.8 mL/kg in the conventional tidal volume group
	**How substantial are the undesirable anticipated effects?**	○Large<br>○Moderate<br>●Small<br>○Trivial<br>------------------<br>○Varies<br>○Don't know		Although a low tidal volume can cause hypercapnia, it can be overcome to some extent by increasing the ventilator rate.<br>In general, patients on mechanical ventilation require sedative or analgesic agents to improve patient-ventilator synchronization and to reduce discomfort during ventilator wearing, but the dosage of sedatives or analgesics during low tidal volume ventilation are not increased.[1]

The relative importance or values of the main outcomes of interest:

Outcome	Relative importance	Certainty of the evidence (GRADE)
Mortality (Short term)(note 1	CRITICAL	⊕⊕⊖⊖<br>LOW
barotrauma	CRITICAL	⊕⊖⊖⊖<br>VERY LOW
VFD(note 2	CRITICAL	⊕⊕⊕⊖<br>MODERATE

Summary of findings:

Outcome	Conventional tidal volume	Low tidal volume	Difference (95% CI)	Relative effect (RR) (95% CI)
Short-term mortality(note 1	397 per 1000	334 per 1000 (266 to 425)	64 fewer per 1000 (from 28 more to 131 fewer)	**RR 0.84** (0.67-1.07)
	380 per 1000	319 per 1000 (255 to 407)	61 fewer per 1000 (from 27 more to 125 fewer)	
	560 per 1000	470 per 1000 (375 to 599)	90 fewer per 1000 (from 39 more to 185 fewer)	
Barotrauma	114 per 1000	93 per 1000 (55 to 160)	20 fewer per 1000 (from 47 more to 59 fewer)	**RR 0.82** (0.48-1.41)
	38 per 1000	31 per 1000 (18 to 54)	7 fewer per 1000 (from 16 more to 20 fewer)	
	120 per 1000	98 per 1000 (58 to 169)	22 fewer per 1000 (from 49 more to 62 fewer)	
VFD(note 2	The mean VFD at 28days was 10 days	The mean VFD at 28days was 11.4. days	MD 2.52 more (from 0.53 more to 4.51 more)	-

CQ03 : Should low tidal volume be used in adult patients with ARDS?				
PATIENTS: ADULT PATIENTS WITH ARDS				
INTERVENTION: LOW TIDAL VOLUME				
CRITERIA		JUDGEMENTS	RESERCH EVIDENCE	ADDITIONAL CONSIDERRATIONS
	**Does the balance between desirable effects and undesirable effects favor the option or the comparison?**	○Favors the intervention<br>●Probably Favors the intervention<br>○Do not know<br>○Probably Favors the comparison<br>○Favors the comparison<br>------------------<br>○Varies	**Summary:**<br>Although the number of deaths in patients with ARDS tends to be lower with low tidal volume than with conventional tidal volume, the difference is insignificant (RR 0.84, 95%CI 0.67-1.07). There is no significant decrease in the incidence of barotrauma in the low tidal volume group (RR0.82, 95%CI 0.48-1.41). The mean ventilator free days (VFD) were significantly greater (mean difference 2.52 days [95%CI 0.53-4.51]) in patients with lower tidal volume compared with conventional tidal volume.	
RESOURCE USE	**How large are the resource requirements (costs)?**	○Favors the comparison<br>○Probably favors the comparison<br>○Does not favor either the intervention or the comparison<br>●Probably favors the intervention<br>○Favors the intervention<br>------------------<br>○Varies<br>○Don't know	Changes in ventilator settings can be applied for all patients by adjusting the settings panel, without any additional resources.	
	**Does the cost effectiveness of the option favor the option or the comparison?**	○Large costs<br>○Moderate costs<br>○Negligible costs and savings<br>○Moderate savings<br>●Large savings<br>------------------<br>○Varies<br>○Don't know	Since no new resources are required, there is no increase in cost.	
EQUITY	**What would be the impact on health equity?**	○Favors the comparison<br>○Probably favors the comparison<br>○Does not favor either the intervention or the comparison<br>○Probably favors the intervention<br>●Favors the intervention<br>------------------<br>○Varies<br>○No included studies	This treatment can be provided in any institution where mechanical ventilators are available. Thus, all patients will be able to receive equal treatment.	

CQ03 : Should low tidal volume be used in adult patients with ARDS?				
PATIENTS : ADULT PATIENTS WITH ARDS				
INTERVENTION : LOW TIDAL VOLUME				
CRITERIA		JUDGEMENTS	RESERCH EVIDENCE	ADDITIONAL CONSIDERRATIONS
ACCEPTABILITY	**Is the option acceptable to key stakeholders?**	●Reduced<br>○Probably reduced<br>○Probably no impact<br>○Probably increased<br>○Increased<br>------------------<br>○Varies<br>○Don't know		
FEASIBILITY	**Is the option feasible to implement?**	○No<br>○Probably no<br>●Probably yes<br>○Yes<br>------------------<br>○Varies<br>○Don't know	The widespread adoption of this ventilation strategy seems to be an achievable goal.	

Recommendation

CQ03：Should low tidal volume be used in adult patients with ARDS?					
**Balance of consequences**	Undesirable consequences *clearly outweigh* desirable consequences in most settings	Undesirable consequences *probably outweigh* desirable consequences in most settings	The balance between desirable and undesirable consequences is *closely balanced or uncertain*	Desirable consequences *probably outweigh* undesirable consequences in most settings	Desirable consequences *clearly outweigh* undesirable consequences in most settings
Judgement	○	○	○	●	○

**Type of recommendation**	Strong recommendation against the intervention	Conditional recommendation against the intervention	Conditional recommendation for either the intervention or the comparison	Conditional recommendation for the intervention
Judgement	○	○	○	●

**Recommendation**	**We recommend the use of low tidal volume at 6-8 mL/kg (predicted body weight: PBW) in adult patients with ARDS. (GRADE 1B, Strength of recommendation “strong recommendation” / Quality of evidence “moderate”)**
**Justification**	**Question:** Should low tidal volume be used in adult patients with ARDS?<br>**Patients:** Adult patients with ARDS<br>**Interventions:** low tidal volume (approximately 6-8 mL/kg PBW)<br>**Comparison:** conventional tidal volume (approximately 10-12 mL/kg PBW)<br>**Outcomes:** Short term mortality *1, barotrauma, Ventilator-free days (VFD) *2<br><br>**Summary of the evidence:**<br>Based on this systematic review, a total of six randomized controlled trials (RCTs) qualified for inclusion where a lung protective ventilation strategy with low tidal volume was studied in adult patients with ARDS. These six RCTs were also analyzed by Petrucci et al. in 2013 and no new RCT has been published since then. Although the duration of follow-up was different, all six RCTs (n=1,305) demonstrated a non-significant decrease in mortality in the low tidal volume group compared with the conventional tidal volume group (RR0.84, 95%CI 0.67-1.07). The occurrence of barotrauma (pneumothorax secondary to elevated airway pressure) was analyzed in all six RCTs, and there was no significant difference between the two groups (RR0.82, 95%CI 0.48-1.41). Ventilator Free Days (VFD) was analyzed in only three RCTs and VFD was significantly longer (median, 2.52 more days) in the low tidal volume group than in the conventional tidal volume group (95%CI 0.53 to 4.51)<br><br>**Quality of the evidence:**<br>The certainty of evidence regarding mortality decreased by two levels and was rated “low” for three reasons. First, there was a difference in the length of follow-up regarding mortality (28-day, 60-day, and hospital) among the RCTs. Second, there was heterogeneity of the cohorts among the RCTs (I^2=50%). Third, the confidence interval was wide. For barotrauma, the certainty of evidence was rated “very low”. For VFD, the certainty of evidence was rated “moderate”. Overall, the quality of evidence was rated “moderate” since a lung protective ventilation strategy had a non-significant, but positive impact on all outcomes.<br><br>Judgement of benefit and harm, resources and cost:<br>A change in tidal volume settings with mechanical ventilation can be applied to all patients undergoing mechanical ventilation and requires no new resources or additional costs. The use of low tidal volume increases VFD significantly with a tendency to decrease mortality and barotrauma. Although this strategy may induce hypercarbia or respiratory acidosis as a potential complication, benefits will outweigh the potential risks.<br><br>Recommensations;<br>We recommend the use of low tidal volume at 6-8 mL/kg (predicted body weight: PBW) in adult patients with ARDS. (GRADE 1B, Strength of recommendation “strong recommendation” / Quality of evidence “moderate”)<br><br>**Supplementary conditions:**<br>Tidal volume is calculated based on PBW {Male:50+0.91 x [Height (cm) – 152.4], Female: 45.5+0.9x[Height (cm) – 152.4]} rather than actual body weight. When a lung protective ventilation strategy is applied, a tidal volume equal to or less than 10mL/kg PBW is considered beneficial. However, the optimal tidal volume still remains to be determined. Of the RCTs analyzed in this review, the actual tidal volume delivered in the lung protective strategy group was 6.2-7.6 mL/kg. Therefore, we recommend a tidal volume of 6-8 mL/kg PBW. In case of an excessive spontaneous

	breathing effort, the actual tidal volume may sometimes exceed the targeted tidal volume. To prevent this, respiratory parameters such as driving pressure or trans-pulmonary pressure may need to be used as monitoring tools to determine an appropriate tidal volume.
**Subgroup considerations**	When patients with ARDS have been on conventional tidal volume (10 mL/kg or greater PBW) for more than a week, the efficacy of introducing a low tidal volume still remains to be determined.
**Implementation considerations**	A low tidal volume (compared to a conventional tidal volume) will decrease the minute ventilation, and as a result, hypercarbia or respiratory acidosis may pursue. However, these incidences will be reversed by increasing the set respiratory rate.
**Monitoring and evaluation considerations**	Monitoring of respiratory parameters (arterial oxygen or carbon dioxide levels, airway pressure etc.) is required to assess adequate arterial oxygenation and ventilation.
**Research priorities**	Since a lung protective strategy has been accepted as the global standard ventilation technique in patients with ARDS, a new RCT to compare the efficacy of a low tidal volume strategy with a conventional tidal volume strategy has not been conducted since 2006. However, the ideal tidal volume still remains to be determined (e.g. 6mL/kg vs. 8mL/kg PBW), and thus, further studies are required. Another future research interest may focus on driving pressure or transpulmonary pressure as potential ideal markers for lung protective low-tidal ventilation.

Note 1) Mortality at the end of the study. For the study by ARDS Network in 2000, a survival curve was used to determine short term mortality at 28-days and the number was used.

Note 2) VFD means the number of days free from mechanical ventilation for the initial 28 days. If the patient expired within 28 days, VFD was counted as a zero.

References

1. Kahn JM, Andersson L, Karir V, et al. Low tidal volume ventilation does not increase sedation use in patients with acute lung injury. *Critical care medicine* **33**(4): 766-71, 2005. PMID 15818103
2. Petrucci N, De Feo C. Lung protective ventilation strategy for the acute respiratory distress syndrome. *Cochrane database of systematic reviews* **2**: CD003844, 2013. PMID 23450544

CQ04. Study flow diagram

3324 records identified through database searching

Medline（PubMed）	1514
Embase	1461
Cochrane CENTRAL	342
Ichushi	7

No additional record identified through other sources

↓

3324 records identified

→ 921 duplicates

↓

2403 records after duplicates removed

→ 2394 records excluded

No available full-text	3
Other than English and Japanese	373
Non-RCT	1132
Different patients /interventions /comparisons	886

↓

9 full-text articles assessed for eligibility

→ 5 of full-text articles excluded w

Different interventions/ comparisons	5

↓

4 included in meta analysis

Short-term mortality	4
VFD	3
Barotrauma	4

CQ04
Risk of bias table, mortality

Outcome		Short term mortality		risk of bias		serious (−1)			
番号	著者名 発表年（Forest plot表示）	risk of bias評価							
		ランダム割付順番の生成 random sequence generation	割り付けの隠蔽化 allocation concealment	ブラインド blinding		不完全なアウトカムデータ incomplete outcome data	選択されたアウトカムの報告 selective outcome reporting	その他のバイアス Other sources of bias	研究内でのバイアスのリスク Risk of bias within a study
				研究参加者と治療提供者 participants and personnel	アウトカム評価者 outcome assessors				
1	Brochard 1998	Low risk	Low risk	High risk	Unclear risk	Low risk	Unclear risk	Low risk	Unclear risk
2	Brower 1999	Unclear risk	Low risk	High risk	Unclear risk	Low risk	Unclear risk	Low risk	Unclear risk
3	ARDS network 2000	Unclear risk	Low risk	High risk	Unclear risk	Low risk	Low risk	Low risk	Unclear risk
4	Villar 2006	Low risk	Low risk	High risk	Unclear risk	Low risk	Unclear risk	Low risk	Unclear risk

番号	著者名 発表年（Forest plot表示）	risk of biasコメント							
1	Brochard 1998	封筒法であるが乱数表を使用している	封筒法であるが隠蔽化されている	一回換気量・プラト―圧の違いを盲検化することは不可能	Blindに関しては不明だが、結果の評価についてバイアスが生じる可能性は低い	100%フォロ―された	protocolを参照できない	ただし中間解析で両群に有意差なく早期中止されている	blind化に関してbiasが大きいが、その他の項目ではlow riskが多いことから、全体的にはunclearとなった。
2	Brower 1999	乱数表を使ったか否かの説明なし	隠蔽化されている	一回換気量・プラト―圧の違いを盲検化することは不可能	記載はないが、all members of the study team and clinical staffsがマスクされているとの記載あり、同様にout comeの評価者もマスクされていると判断、統計解析する人がマスクされているかの記載はなし	100%フォロ―された	protocolを参照できない	intension-to-treat解析の記載はないが、その他のbiasはなし<br>ただし、中間解析に基づき、研究が早期中止されている	blind化に関してbiasが大きいが、その他の項目ではlow riskが多いことから、全体的にはunclearとなった。
3	ARDS network 2000	乱数表を使ったか否かの説明なし	隠蔽化されている	一回換気量・プラト―圧の違いを盲検化することは不可能	Blindに関しては不明だが、結果の評価についてバイアスが生じる可能性は低い	100%フォロ―された	100%報告された	ただし、中間解析に基づき、研究が早期中止されている	blind化に関してbiasが大きいが、その他の項目ではlow riskが多いことから、全体的にはunclearとなった。
4	Villar 2006	封筒法であるが乱数表を使用している	封筒法であるが隠蔽化されている	一回換気量・プラト―圧の違いを盲検化することは不可能	Blindに関しては不明だが、結果の評価についてバイアスが生じる可能性は低い	100%フォロ―された。しかしランダム化に問題ありとして8人が除外された。mortalityに関しては全数が報告されていたので、除外前のデータを採用した。	100%報告されたと思われるが、protocolを参照できない	ただし、中間解析に基づき、研究が早期中止されている	blind化に関してbiasが大きいが、その他の項目ではlow riskが多いことから、全体的にはunclearとなった。

CQ04
Risk of bias table VFD

Outcome		VFD		risk of bias		serious (−1)			
番号	著者名 発表年（Forest plot表示）	risk of bias評価							
		ランダム割付順番の生成 random sequence generation	割り付けの隠蔽化 allocation concealment	ブラインド blinding 研究参加者と治療提供者 participants and personnel	ブラインド blinding アウトカム評価者 outcome assessors	不完全なアウトカムデータ incomplete outcome data	選択されたアウトカムの報告 selective outcome reporting	その他のバイアス Other sources of bias	研究内でのバイアスのリスク Risk of bias within a study
1	Brower 1999	Unclear risk	Low risk	High risk	Unclear risk	Low risk	Unclear risk	Low risk	Unclear risk
2	ARDS network 2000	Unclear risk	Low risk	High risk	Unclear risk	Low risk	Low risk	Low risk	Unclear risk
3	Villar 2006	Low risk	Low risk	High risk	Unclear risk	High risk	Unclear risk	Low risk	High risk
4		Unclear risk	Unclear risk	Unclear risk	Unclear risk	Unclear risk	Unclear risk	Unclear risk	Unclear risk
5		Unclear risk	Unclear risk	Unclear risk	Unclear risk	Unclear risk	Unclear risk	Unclear risk	Unclear risk
6		Unclear risk	Unclear risk	Unclear risk	Unclear risk	Unclear risk	Unclear risk	Unclear risk	Unclear risk
7		Unclear risk	Unclear risk	Unclear risk	Unclear risk	Unclear risk	Unclear risk	Unclear risk	Unclear risk
8		Unclear risk	Unclear risk	Unclear risk	Unclear risk	Unclear risk	Unclear risk	Unclear risk	Unclear risk
9		Unclear risk	Unclear risk	Unclear risk	Unclear risk	Unclear risk	Unclear risk	Unclear risk	Unclear risk
10		Unclear risk	Unclear risk	Unclear risk	Unclear risk	Unclear risk	Unclear risk	Unclear risk	Unclear risk

番号	著者名 発表年（Forest plot表示）	risk of biasコメント							
1	Brower 1999	乱数表を使ったか否かの説明なし	隠蔽化されている	一回換気量･プラトー圧の違いを盲検化することは不可能	記載はないが、all members of the study team and clinical staffsがマスクされているとの記載あり、同様にout comeの評価者もマスクされていると判断、統計解析する人がマスクされているかの記載はなし	100%フォローされた	protocolを参照できない	intension-to-treat解析の記載はないが、その他のbiasはなし ただし、中間解析に基づき、研究が早期中止されている	blind化に関してbiasが大きいが、その他の項目ではlow riskが多いことから、全体的にはunclearとなった。
2	ARDS network 2000	乱数表を使ったか否かの説明なし	隠蔽化されている	一回換気量･プラトー圧の違いを盲検化することは不可能	Blindに関しては不明だが、結果の評価についてバイアスが生じる可能性は低い	100%フォローされた	100%報告された	ただし、中間解析に基づき、研究が早期中止されている	blind化に関してbiasが大きいが、その他の項目ではlow riskが多いことから、全体的にはunclearとなった。
3	Villar 2006	封筒法であるが乱数表を使用している	封筒法であるが隠蔽化されている	一回換気量･プラトー圧の違いを盲検化することは不可能	Blindに関しては不明だが、結果の評価についてバイアスが生じる可能性は低い	8人がランダム化に問題があったとして除外されている。	100%報告されたと思われるが、protocolを参照できない	ただし、中間解析に基づき、研究が早期中止されている	blind化に関してbiasが大きく、このアウトカムに対してはランダム化にも問題を抱えているために、全体としてhigh riskであると判断した。

CQ04
Risk of bias table Barotrauma

Outcome		Barotrauma		risk of bias		serious (−1)			
番号	著者名 発表年<br>（Forest plot表示）	risk of bias評価							
		ランダム割付順番の生成<br>random sequence generation	割り付けの隠蔽化<br>allocation concealment	ブラインド<br>blinding<br>研究参加者と治療提供者<br>participants and personnel	ブラインド<br>blinding<br>アウトカム評価者<br>outcome assessors	不完全なアウトカムデータ<br>incomplete outcome data	選択されたアウトカムの報告<br>selective outcome reporting	その他のバイアス<br>Other sources of bias	研究内でのバイアスのリスク<br>Risk of bias within a study
1	Brochard 1998	Low risk	Low risk	High risk	Unclear risk	Low risk	Unclear risk	Low risk	Unclear risk
2	Brower 1999	Unclear risk	Low risk	High risk	Unclear risk	Low risk	Unclear risk	Low risk	Unclear risk
3	ARDS network 2000	Unclear risk	Low risk	High risk	Unclear risk	Low risk	Low risk	Low risk	Unclear risk
4	Villar 2006	Low risk	Low risk	High risk	Unclear risk	High risk	Unclear risk	Low risk	High risk
5		Unclear risk	Unclear risk	Unclear risk	Unclear risk	Unclear risk	Unclear risk	Unclear risk	Unclear risk
6		Unclear risk	Unclear risk	Unclear risk	Unclear risk	Unclear risk	Unclear risk	Unclear risk	Unclear risk
7		Unclear risk	Unclear risk	Unclear risk	Unclear risk	Unclear risk	Unclear risk	Unclear risk	Unclear risk
8		Unclear risk	Unclear risk	Unclear risk	Unclear risk	Unclear risk	Unclear risk	Unclear risk	Unclear risk
9		Unclear risk	Unclear risk	Unclear risk	Unclear risk	Unclear risk	Unclear risk	Unclear risk	Unclear risk
10		Unclear risk	Unclear risk	Unclear risk	Unclear risk	Unclear risk	Unclear risk	Unclear risk	Unclear risk

番号	著者名 発表年<br>（Forest plot表示）	risk of biasコメント							
1	Brochard 1998	封筒法であるが乱数表を使用している	封筒法であるが隠蔽化されている	一回換気量･プラトー圧の違いを盲検化することは不可能	Blindに関しては不明だが、結果の評価についてバイアスが生じる可能性は低い	100%フォローされた	protocolを参照できない	ただし中間解析で両群に有意差なく早期中止されている	blind化に関してbiasが大きいが、その他の項目ではlow riskが多いことから、全体的にはunclearとなった。
2	Brower 1999	乱数表を使ったか否かの説明なし	隠蔽化されている	一回換気量･プラトー圧の違いを盲検化することは不可能	記載はないが、all members of the study team and clinical staffsがマスクされているとの記載あり、同様にout comeの評価者もマスクされていると判断、統計解析する人がマスクされているかの記載はなし	100%フォローされた	protocolを参照できない	intension-to-treat解析の記載はないが、その他のbiasはなし<br>ただし、中間解析に基づき、研究が早期中止されている	blind化に関してbiasが大きいが、その他の項目ではlow riskが多いことから、全体的にはunclearとなった。
3	ARDS network 2000	乱数表を使ったか否かの説明なし	隠蔽化されている	一回換気量･プラトー圧の違いを盲検化することは不可能	Blindに関しては不明だが、結果の評価についてバイアスが生じる可能性は低い	100%フォローされた	100%報告された	ただし、中間解析に基づき、研究が早期中止されている	blind化に関してbiasが大きいが、その他の項目ではlow riskが多いことから、全体的にはunclearとなった。
4	Villar 2006	封筒法であるが乱数表を使用している	封筒法であるが隠蔽化されている	一回換気量･プラトー圧の違いを盲検化することは不可能	Blindに関しては不明だが、結果の評価についてバイアスが生じる可能性は低い	8人がランダム化に問題があったとして除外されている。	100%報告されたと思われるが、protocolを参照できない	ただし、中間解析に基づき、研究が早期中止されている	blind化に関してbiasが大きく、このアウトカムに対してはランダム化にも問題を抱えているために、全体としてhigh riskであると判断した。

CQ04 Risk of bias summary, Risk of bias graph

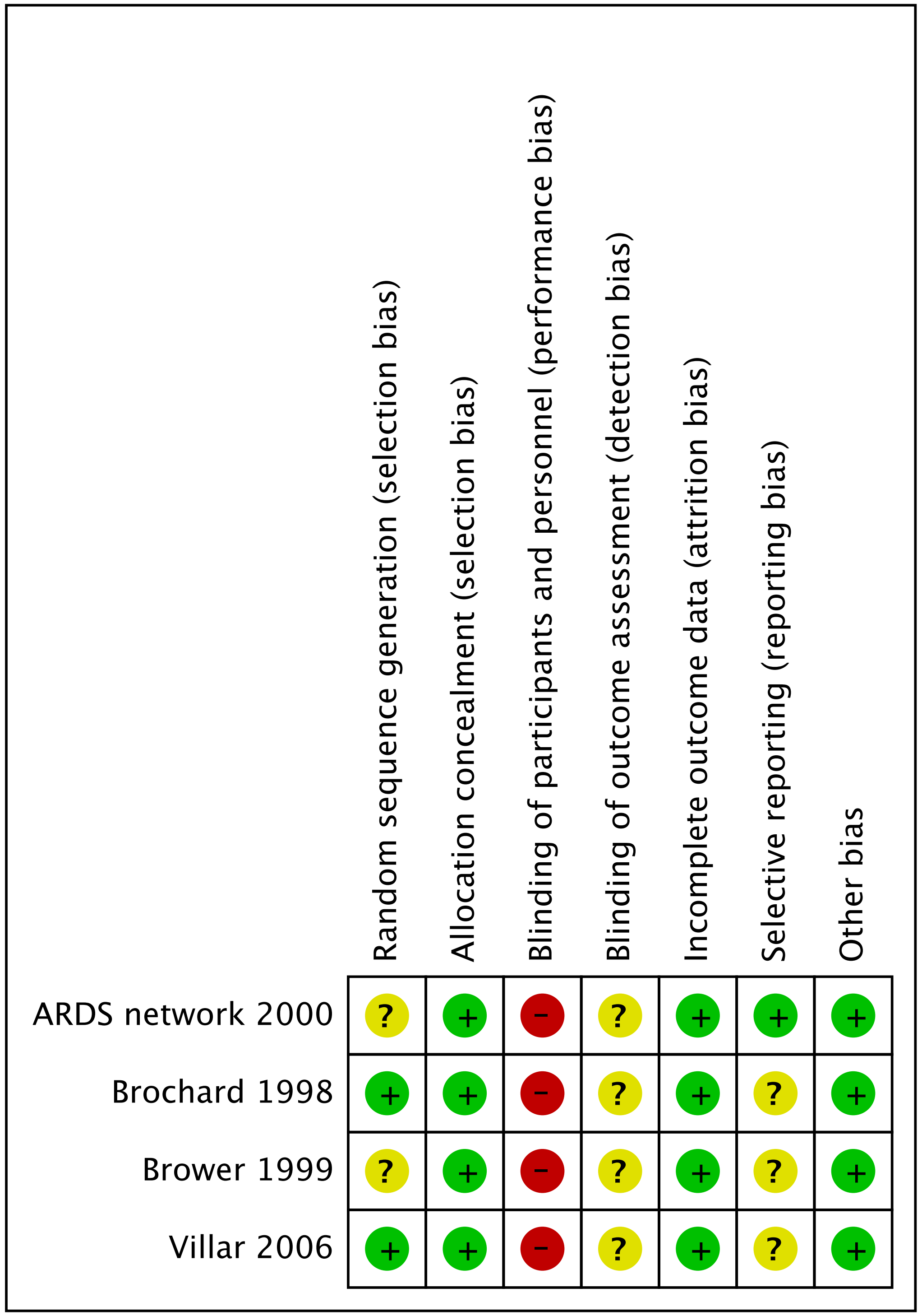

Short term mortality

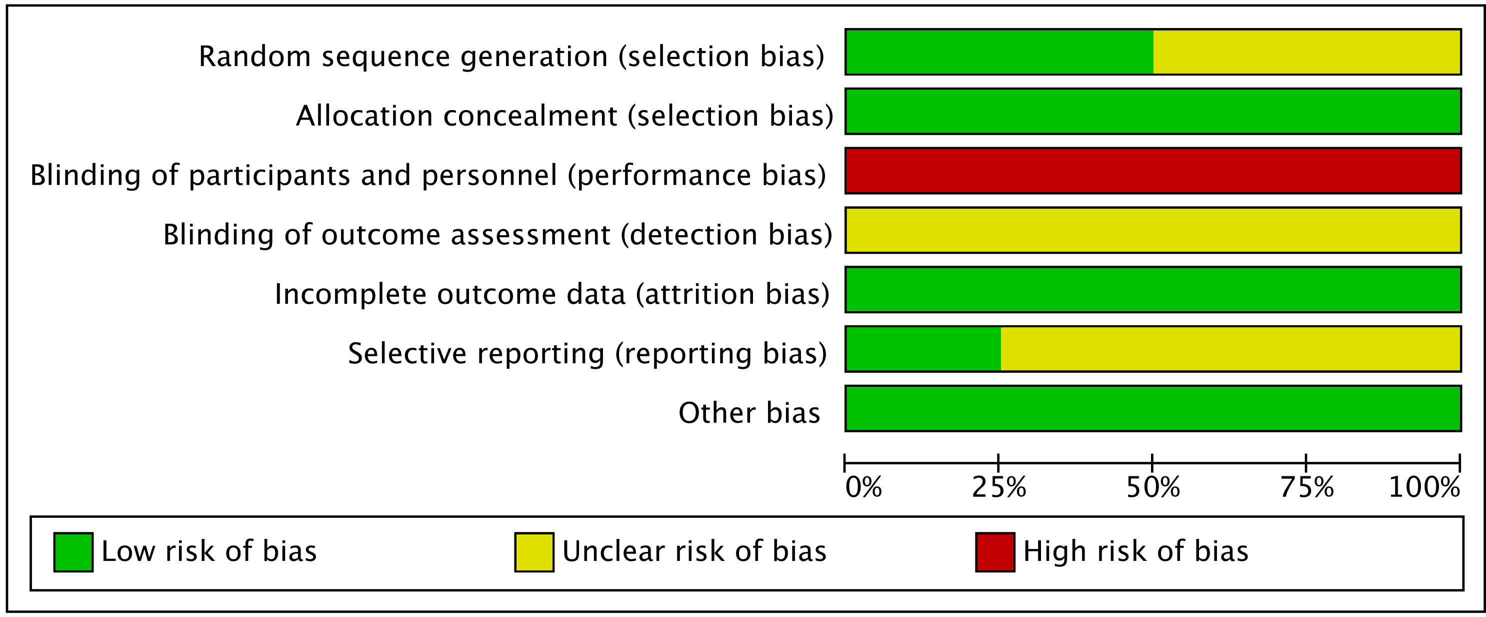

CQ04 Risk of bias summary, Risk of bias graph

VFD

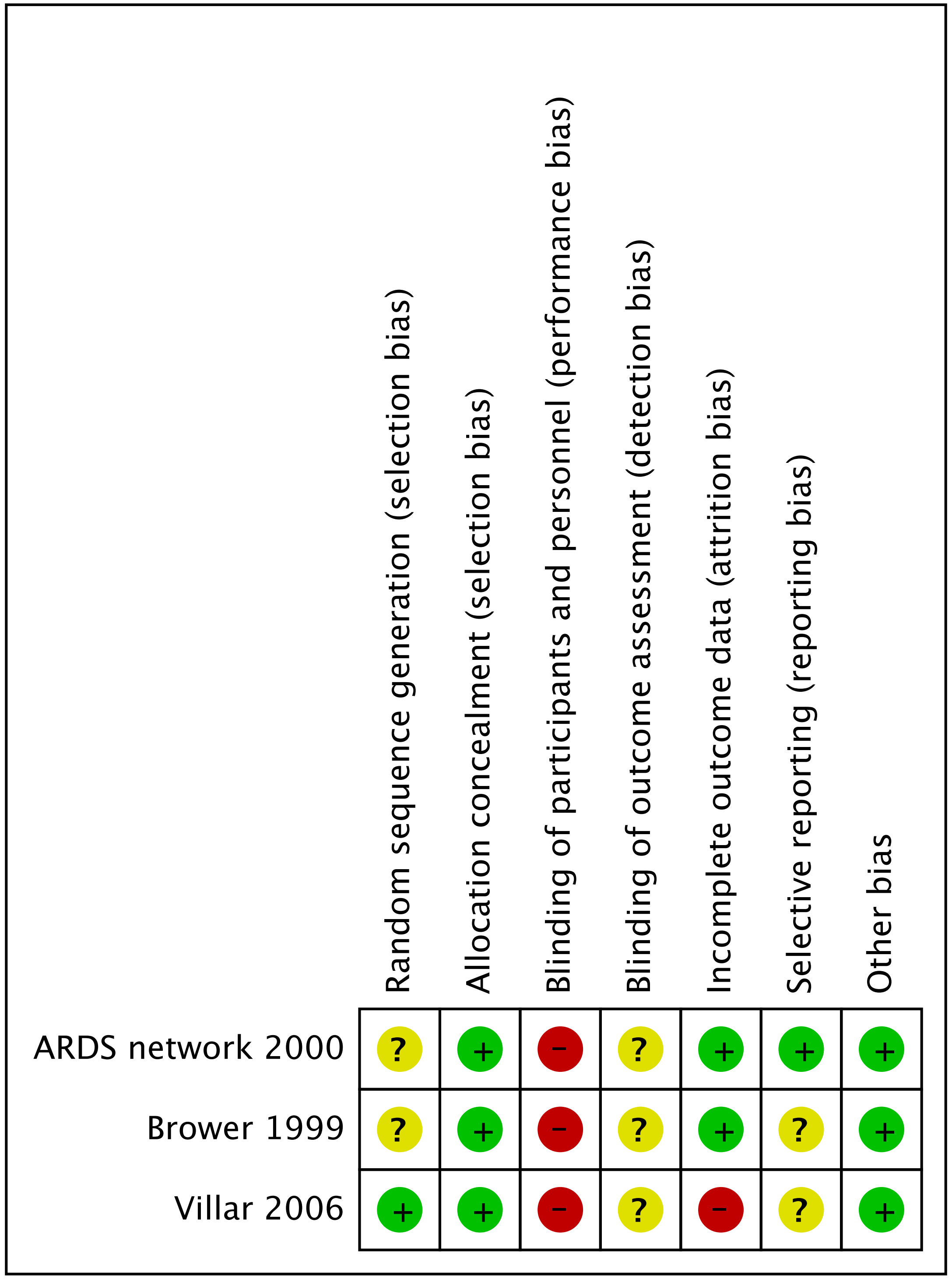

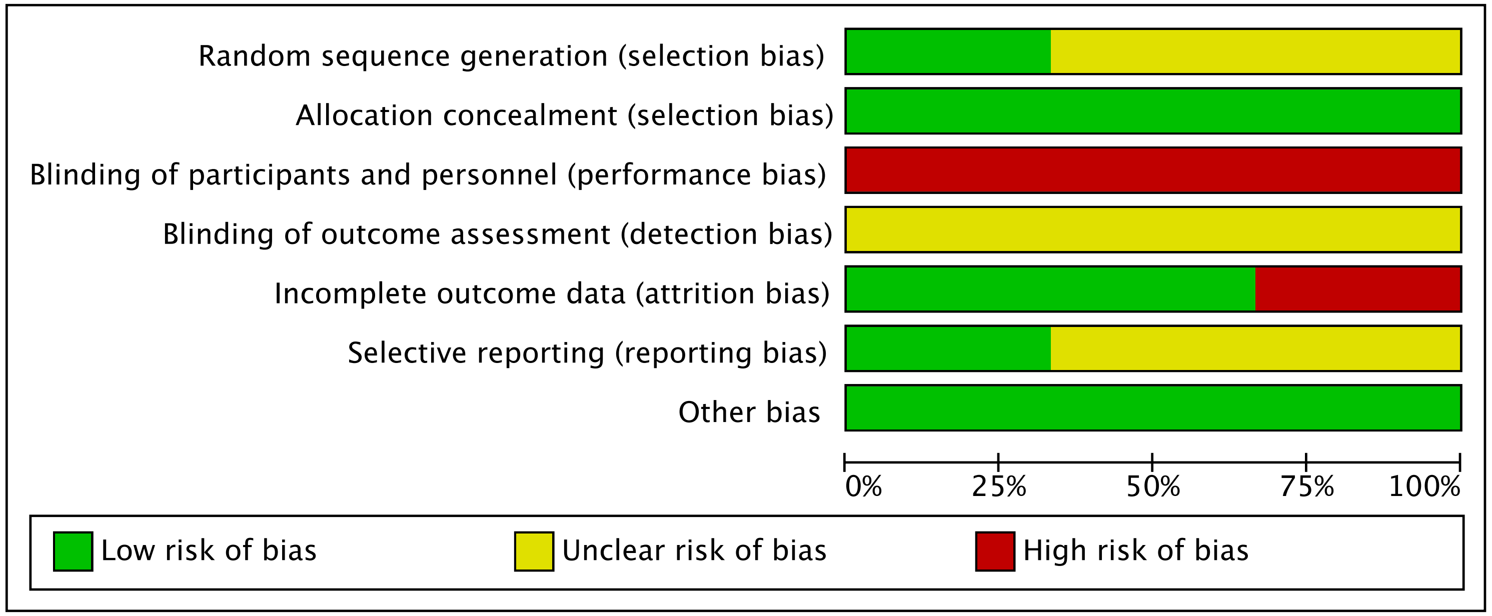

CQ04 Risk of bias summary, Risk of bias graph

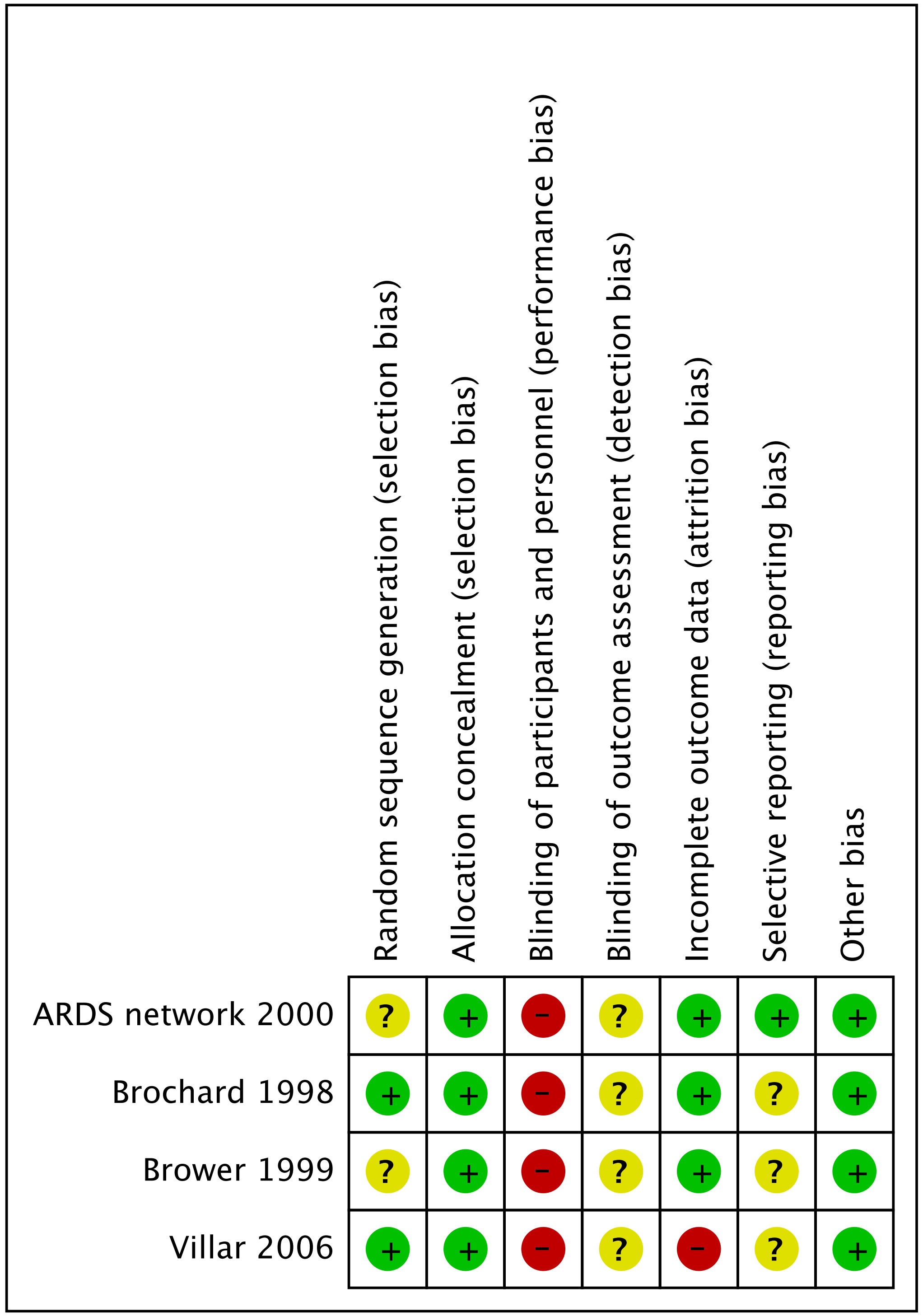

Barotrauma

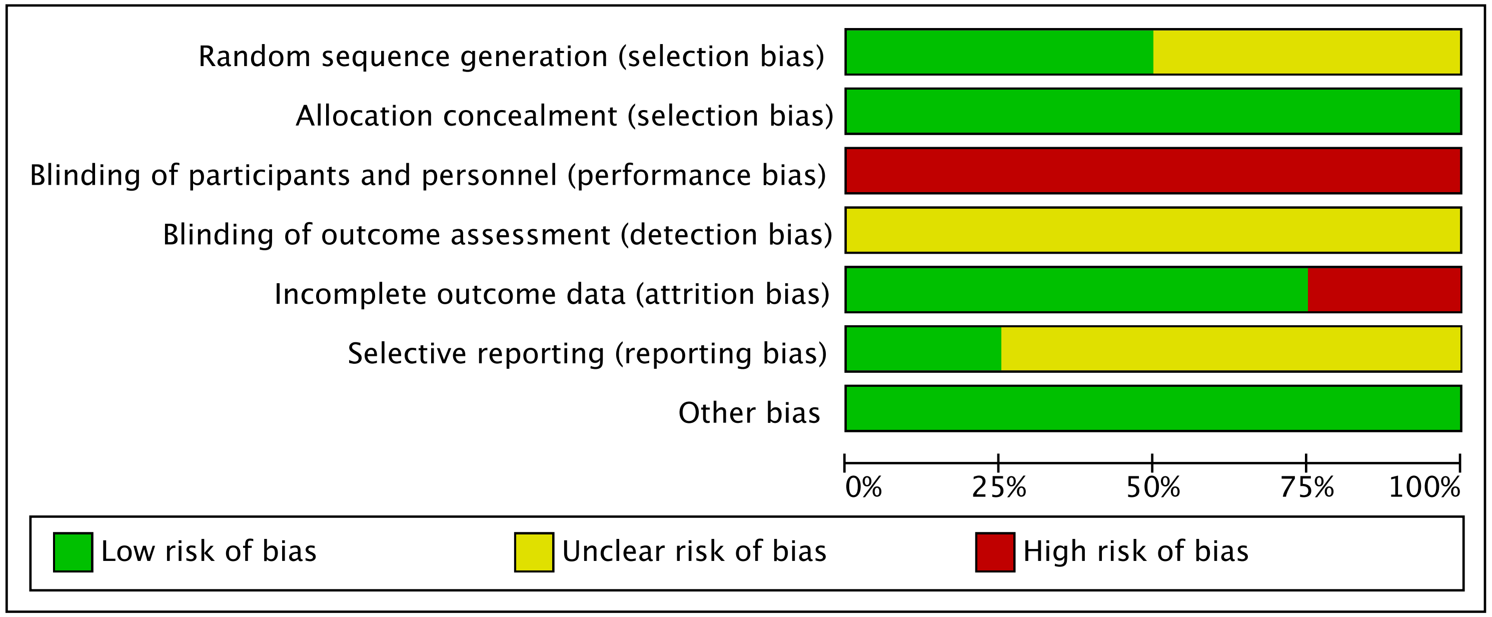

Short term mortality

Study or Subgroup	low-plateau pressure Events	low-plateau pressure Total	high-plateau pressure Events	high-plateau pressure Total	Weight	Risk Ratio M-H, Random, 95% CI
ARDS network 2000	112	432	150	429	36.4%	0.74 [0.60, 0.91]
Brochard 1998	27	58	22	58	23.7%	1.23 [0.80, 1.89]
Brower 1999	13	26	12	26	17.8%	1.08 [0.62, 1.91]
Villar 2006	17	53	28	50	22.1%	0.57 [0.36, 0.91]
**Total (95% CI)**		**569**		**563**	**100.0%**	**0.84 [0.62, 1.15]**
Total events	169		212			

Heterogeneity: $Tau^2 = 0.06$; $Chi^2 = 7.47$, df = 3 (P = 0.06); $I^2 = 60\%$
Test for overall effect: Z = 1.07 (P = 0.29)

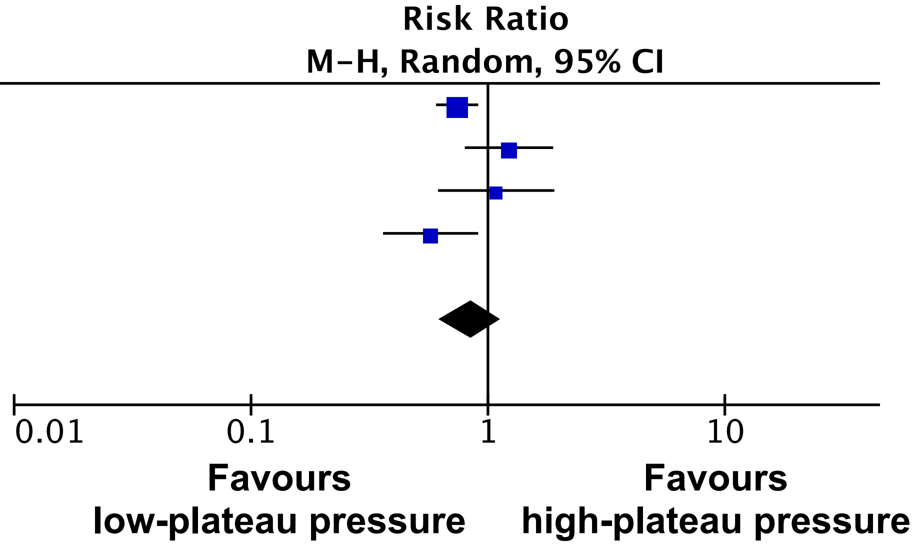

VFD

Study or Subgroup	low-plateau pressure Mean	low-plateau pressure SD	low-plateau pressure Total	high-plateau pressure Mean	high-plateau pressure SD	high-plateau pressure Total	Weight	Mean Difference IV, Random, 95% CI
ARDS network 2000	12	11	432	10	11	429	64.2%	2.00 [0.53, 3.47]
Brower 1999	9.6	10.3	26	9.3	9.8	26	11.7%	0.30 [−5.16, 5.76]
Villar 2006	10.9	9.5	50	6	8	45	24.1%	4.90 [1.38, 8.42]
**Total (95% CI)**			**508**			**500**	**100.0%**	**2.50 [0.51, 4.49]**

Heterogeneity: $Tau^2 = 1.04$; $Chi^2 = 2.77$, df = 2 (P = 0.25); $I^2 = 28\%$
Test for overall effect: Z = 2.47 (P = 0.01)

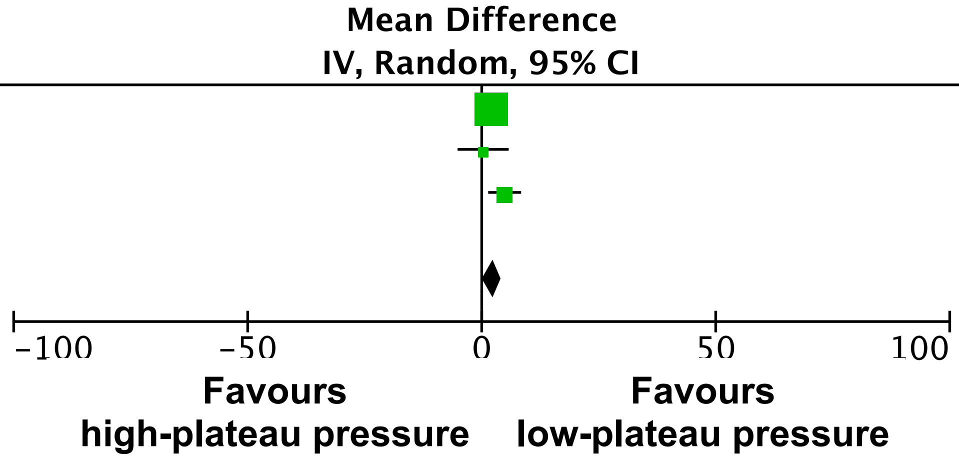

Barotrauma

Study or Subgroup	low-plateau pressure Events	low-plateau pressure Total	high-plateau pressure Events	high-plateau pressure Total	Weight	Risk Ratio M-H, Random, 95% CI
ARDS network 2000	43	432	47	429	79.7%	0.91 [0.61, 1.34]
Brochard 1998	8	58	7	58	13.6%	1.14 [0.44, 2.95]
Brower 1999	2	26	1	26	2.2%	2.00 [0.19, 20.72]
Villar 2006	2	50	4	45	4.5%	0.45 [0.09, 2.34]
**Total (95% CI)**		**566**		**558**	**100.0%**	**0.92 [0.65, 1.31]**
Total events	55		59			

Heterogeneity: $Tau^2 = 0.00$; $Chi^2 = 1.35$, df = 3 (P = 0.72); $I^2 = 0\%$
Test for overall effect: Z = 0.44 (P = 0.66)

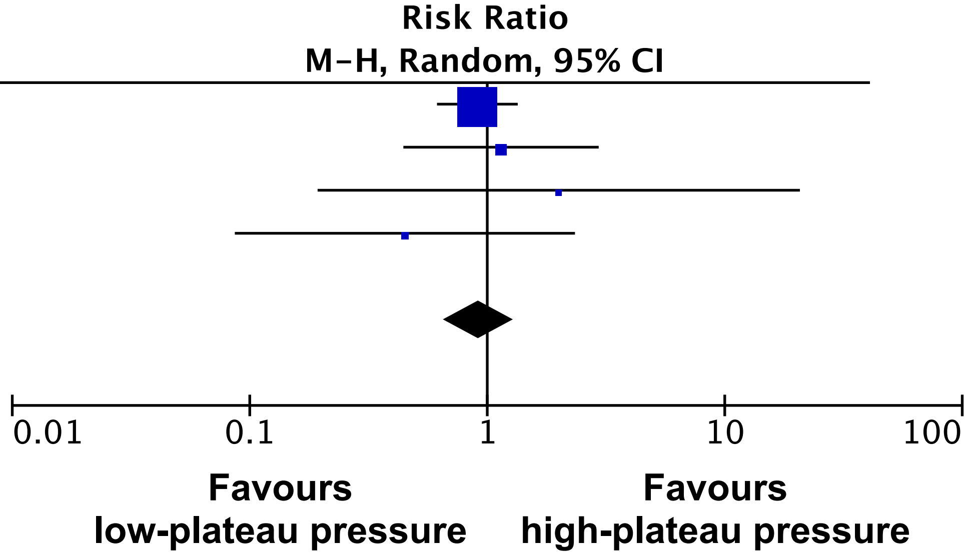

Summary of findings:

Lower plateau pressure (≦30 cmH_2O) compared to higher plateau pressure (>30cmH_2O) for ARDS

Patient or population: ARDS
Intervention: Lower plateau pressure (≦30 cmH_2O)
Comparison: higher plateau pressure (>30cmH_2O)

Outcomes	Anticipated absolute effects* (95% CI)		Relative effect (95% CI)	№ of participants (studies)	Quality of the evidence (GRADE)	Comments
	**Risk with** higher plateau pressure (>30cmH_2O)	**Risk with** lower plateau pressure (≦30 cmH_2O)				
Short term mortality	**Study population**		**RR 0.84** (0.62 to 1.15)	1132 (4 RCTs)	⊕⊕◯◯ LOW [1,2]	
	377 per 1000	**316 per 1000** (233 to 433)				
	**Low**					
	380 per 1000	**319 per 1000** (236 to 437)				
	**High**					
	560 per 1000	**470 per 1000** (347 to 644)				
VFD	Mean **9.0** days	2.5 day more MD (0.51 more to 4.49 more)		1008 (3 RCTs)	⊕⊕⊕◯ MODETATE [1]	
Barotrauma	**Study population**		**RR 0.92** (0.65 to 1.31)	1124 (4 RCTs)	⊕◯◯◯ VERY LOW [1,2,3]	
	106 per 1000	**97 per 1000** (69 to 139)				
	**Low**					
	38 per 1000	**35 per 1000** (24 to 50)				
	**High**					
	120 per 1000	**110 per 1000** (78 to 157)				

***The risk in the intervention group** (and its 95% confidence interval) is based on the assumed risk in the comparison group and the **relative effect** of the intervention (and its 95% CI).

CI: Confidence interval; **RR:** Risk ratio ; **MD:** Mean difference

GRADE Working Group grades of evidence
High quality: We are very confident that the true effect lies close to that of the estimate of the effect
Moderate quality: We are moderately confident in the effect estimate: The true effect is likely to be close to the estimate of the effect, but there is a possibility that it is substantially different
Low quality: Our confidence in the effect estimate is limited: The true effect may be substantially different from the estimate of the effect
Very low quality: We have very little confidence in the effect estimate: The true effect is likely to be substantially different from the estimate of effect

1. Since several studies included this analysis could not make physicians blinded to intervention, the quality of evidence was downgraded by one level.
2. Since the confidence interval is wide, the quality of evidence was downgraded by one level.
3. Since the sample size was very small, the quality of evidence was downgraded by one level.

CQ4

Question: How do we set the plateau pressure on artificial respiratory ventilation in adult patients with ARDS?

Quality assessment							№ of patients		Effect		Quality	Importance
№ of studies	Study design	Risk of bias	Inconsistency	Indirectness	Imprecision	Other considerations	Lower plateau pressure (≦30 cmH_2O)	higher plateau pressure (>30cmH_2O)	Relative (95% CI)	Absolute (95% CI)		
Short term mortality												
4	randomised trials	serious [1]	not serious	not serious	serious [2]	none	169/569 (29.7%)	212/563 (37.7%)	**RR 0.84** (0.62 to 1.15)	60 fewer per 1000 (from 56 more to 143 fewer)	⨁⨁◯◯ LOW [1,2]	CRITICAL
								38.0%		61 fewer per 1000 (from 57 more to 144 fewer)		
								56.0%		90 fewer per 1000 (from 84 more to 213 fewer)		
VFD												
3	randomised trials	serious [1]	not serious [2]	not serious	not serious	none	508	500		MD **2.5 day more** (0.51 more to 4.49 more)	⨁⨁⨁◯ MODERATE [1]	CRITICAL
Barotrauma												
4	randomised trials	serious [1]	not serious [3]	not serious	Very serious [2,3]	none	55/566 (9.7%)	59/558 (10.6%)	**RR 0.92** (0.65 to 1.31)	8 fewer per 1000 (from 33 more to 37 fewer)	⨁◯◯◯ VERY LOW [1,2,3]	CRITICAL
								3.8%		3 fewer per 1000 (from 12 more to 13 fewer)		
								12.0%		10 fewer per 1000 (from 37 more to 42 fewer)		

CI: Confidence interval; **RR:** Risk ratio; **MD:** Mean difference

1. Since several studies included this analysis could not make physicians blinded to intervention, the quality of evidence was downgraded by one level.
2. Since the confidence interval is wide, the quality of evidence was downgraded by one level.
3. Since the sample size was very small, the quality of evidence was downgraded by one level.

Evidence-to-Decision table

CQ4: How do we set the plateau pressure for mechanical ventilation in adult patients with ARDS?

PATIENTS: ADULT PATIENTS WITH ARDS

INTERVENTION: LOWER PLATEAU PRESSURE (≦30 cmH_2O)

	CRITERIA	JUDGEMENTS	RESEARCH EVIDENCE	ADDITIONAL CONSIDERATIONS
PROBLEM	**Is there a problem priority?**	○No<br>○Probably no<br>●Probably yes<br>○Yes<br>------------------<br>○Varies<br>○Don't know	When providing mechanical ventilation to adult patients with ARDS, decreased lung compliance is one of the main etiologic factors for the development of ventilator associated lung injury. Ventilator associated lung injury leads not only to delayed weaning, but also increased mortality [1]. Among several causes of ventilator associated lung injury, increased tidal volume and airway pressure are important. Limiting plateau pressure can control both of these factors [2]. Although limiting the plateau pressure is beneficial, it may lead to adverse events such as hypercapnia [3]. While there is as yet no practical way to determine optimal plateau pressure, a method should be developed to minimize ventilator associated lung injury. Therefore, the priority of this CQ is high. As the optimal plateau pressure is undefined, studies comparing various plateau pressures are needed.	
BENEFITS & HARMS OF THE OPTIONS	**What is the overall certainty of the evidence of effects?**	○Very low<br>○Low<br>●Moderate<br>○High<br>------------------<br>○No included studies	(see below)	
	**Is there important uncertainty about or variability in how much people value the main outcomes?**	○Important uncertainty or variability<br>○Possibly important uncertainty or variability<br>○Possibly no important uncertainty or variability<br>●No important uncertainty or variability<br>------------------<br>○No known undesirable outcomes		
	**How substantial are the desirable anticipated effects?**	○Trivial<br>○Small<br>●Moderate<br>○Large<br>------------------<br>○Varies<br>○Don't know		
	**How substantial are the undesirable anticipated effects?**	○Large<br>○Moderate<br>○Small<br>●Trivial<br>------------------<br>○Varies<br>○Don't know		

The relative importance or values of the main outcomes of interest:

Outcomes	Relative importance	Certainty of the evidence (GRADE)
Short term mortality (note 1	CRITICAL	⊕⊕○○ LOW
VFD (note 2	CRITICAL	⊕⊕⊕○ MODERATE
Barotrauma	CRITICAL	⊕○○○ VERY LOW

Summary of findings:

Outcomes	Risk with higher plateau pressure (>30cmH_2O)	Risk with lower plateau pressure (≦30 cmH_2O)	Absolute (95% CI)	Relative effect (95% CI)
Short term mortality (note 1	377/ 1000	316 / 1000 (233 to 433)	60 fewer per 1000 (from 56 more to 143 fewer)	RR 0.84 (0.62 to 1.15)
	380 / 1000	319 / 1000 (236 to 437)	61 fewer per 1000 (from 57 more to 144 fewer)	
	560 / 1000	470 / 1000 (347 to 644)	90 fewer per 1000 (from 84 more to 213 fewer)	
VFD (note 2	Avereage 9.0 day	Average 11.5 day	MD 2.5 more (0.51 more to 4.49 more)	-
Barotrauma	106 / 1000	97 / 1000 (69 to 139)	8 fewer per 1000 (from 33 more to 37 fewer)	RR 0.92 (0.65 to 1.31)
	38 / 1000	35 / 1000 (25 to 50)	3 fewer per 1000 (from 12 more to 13 fewer)	
	120 / 1000	110 / 1000 (78 to157)	10 fewer per 1000 (from 37 more to 42 fewer)	

Summary: After starting mechanical ventilation, setting plateau pressure below

	**Does the balance between desirable effects and undesirable effects favor the option or the comparison?**	○Favors the comparison<br>○Probably favors the comparison<br>○Does not favor either the intervention or the comparison<br>○Probably favors the intervention<br>●Favors the intervention<br>------------------<br>○Varies<br>○Don't know	30cmH2O show both the extention of VFD (absolute difference 2.5, 95% CI 0.51-4.49) and trends of decrease of mortality RR 0.84, 95% CI 0.62-1.15), but neither are not significant in stastical analysis.	
RESOURCE USE	**How large are the resource requirements (costs)?**	○Large costs<br>○Moderate costs<br>○Negligible costs and savings<br>○Moderate savings<br>●Large savings<br>------------------<br>○Varies<br>○Don't know	No additional resources are necessary as there is only a change in ventilator settings.	
	**Does the cost effectiveness of the option favor the option or the comparison?**	○Favors the comparison<br>○Probably favors the comparison<br>○Does not favor either the intervention or the comparison<br>○Probably favors the intervention<br>●Favors the intervention<br>------------------<br>○Varies<br>○No included studies	As there are no additional resources required, the cost effectiveness of the option favors the intervention.	
EQUITY	**What would be the impact on health equity?**	●Reduced<br>○Probably reduced<br>○Probably no impact<br>○Probably increased<br>○Increased<br>------------------<br>○Varies<br>○Don't know	The suggested intervention is available in any medical facility that provides mechanical ventilation, no additional procedures are required. Thus, unfairness cannot occur.	
ACCEPTABILITY	**Is the option acceptable to key stakeholders?**	○No<br>○Probably no<br>●Probably yes<br>○Yes<br>------------------<br>○Varies<br>○Don't know		

FEASIBILITY	**Is the option feasible to implement?**	○No<br>○Probably no<br>○Probably yes<br>●Yes<br>------------------<br>○Varies<br>○Don't know	It is feasible, because it is only a change in ventilator settings.	

Recommendation

CQ4: How do we set the plateau pressure for mechanical ventilation in adult patients with ARDS?

**Balance of consequences**	Undesirable consequences *clearly outweigh* desirable consequences in most settings	Undesirable consequences *probably outweigh* desirable consequences in most settings	The balance between desirable and undesirable consequences is closely *balanced or uncertain*	Desirable consequences *probably outweigh* undesirable consequences in most settings	Desirable consequences *clearly outweigh* undesirable consequences in most settings
Judgement	○	○	○	●	○

**Type of recommendation**	We recommend against offering this option	We suggest not offering this option	We suggest offering this option	We recommend offering this option
Judgement	○	○	●	○

**Recommendation**	**We suggest setting the plateau pressure at 30cmH$_2$0 or less in adult patients with ARDS undergoing mechanical ventilation. (GRADE2B, weak recommendation / evidence level moderate)**
**Justification**	**Question:** What is the optimal plateau pressure for mechanical ventilation in adult patients with ARDS?<br>**Patients:** ARDS patients requiring mechanical ventilation.<br>**Interventions:** Lower plateau pressure (≦30 cmH$_2$O)<br>**Comparison:** higher plateau pressure (>30cmH$_2$O)<br>**Outcomes:** Mortality*1, Ventilator Free Days*2, barotrauma*3<br><br>**Summary of the evidence:** Four RCTs (total number of patients=1132) were selected for this systematic review. By setting the plateau pressure below 30cmH$_2$O, the number of ventilator free days (VFD) was significantly increased (mean 2.5days, 95% CI 0.51-4.49). The mortality (RR 0.84, 95%CI 0.62-1.15) and lung injury caused by high airway pressure (RR 0.92, 95%CI 0.65-1.31) were decreased, but there is no statistically significant difference.<br><br>**Quality of the evidence:** In these RCTs that compare values of ventilator settings (such as comparing two plateau pressures), it is difficult to blind the entire study to patients and medical staff. Thus, we determined the risk of bias for all outcomes as 'serious' and downgraded them as a whole. There is no inconsistency or indirectness. Although the total number of events (death, 381/1132 patients), is sufficient, there is no statistically significant difference. The 95% confidence interval is considered to be wide. Thus we determined it is 'serious' in terms of mortality. Overall, we conclude that the quality of evidence in these 4 RCTs is "moderate".<br><br>**Judgement of benefit and harm, resources and cost:** Ventilator associated respiratory injury caused by an increase in plateau pressure is obviously a serious adverse event. Thus, limiting the plateau pressure would be accepted by patients without any hesitation. It is assumed that most patients will select limiting plateau pressure on the ventilator. There is no change in resources required by changing the ventilator settings. As there is no additional increase of required resources, the benefit prevails. Hypoxemia, hypercapnia and increased work of breathing work caused by inappropriate ventilator settings are possible harms in this CQ. However, these harms are relatively permissive and should not cause any serious sequelae.<br><br>**Recommendations;** We suggest setting the plateau pressure at 30cmH$_2$0 or less in adult patients with ARDS undergoing mechanical ventilation. (GRADE2B, weak recommendation / evidence level moderate)<br><br>**Additional considerations:** As trans-pulmonary pressure is now drawing a lot of attention, it is necessary to consider a comparison of plateau pressures when patients are spontaneously breathing.
**Subgroup considerations**	None
**Implementation considerations**	In some cases, it may be difficult to ensure sufficient minute ventilation by managing low plateau pressure, that may cause decreased oxygenation, increased carbon dioxide concentration and/or

	increased work of breathing.
**Monitoring and evaluation considerations**	As the intervention is a change in ventilator settings, we should monitor oxygenation and other appropriate parameters of mechanical ventilation.
**Research possibilities**	As the optimal plateau pressure is undefined, studies comparing various plateau pressures are needed. As trans-pulmonary pressure is now drawing a lot of attention, it is necessary to consider a comparison of plateau pressures when patients are spontaneously breathing.

Note 1) Mortality rate in ARDS 2000 is 28days mortality (read from Kaplan-Meier Curve), and the others are death at the end of research.

Note 2) VFD means the number of days free from mechanical ventilation in the initial 28 days. If the patient expired within 28 days, VFD was counted as zero.

CQ05. Study flow diagram

*This CQ was partly evaluated by Santa Cruz using Cochrane database (to May 2013)[1)]. We also searched literature from 2013 to May 2015.

1. Santa Cruz R, Rojas JI, Nervi R, et al. High versus low positive end-expiratory pressure (PEEP) levels for mechanically ventilated adult patients with acute lung injury and acute respiratory distress syndrome. *Cochrane Database Syst Rev* **6**: CD009098, 2013. PMID 23740697

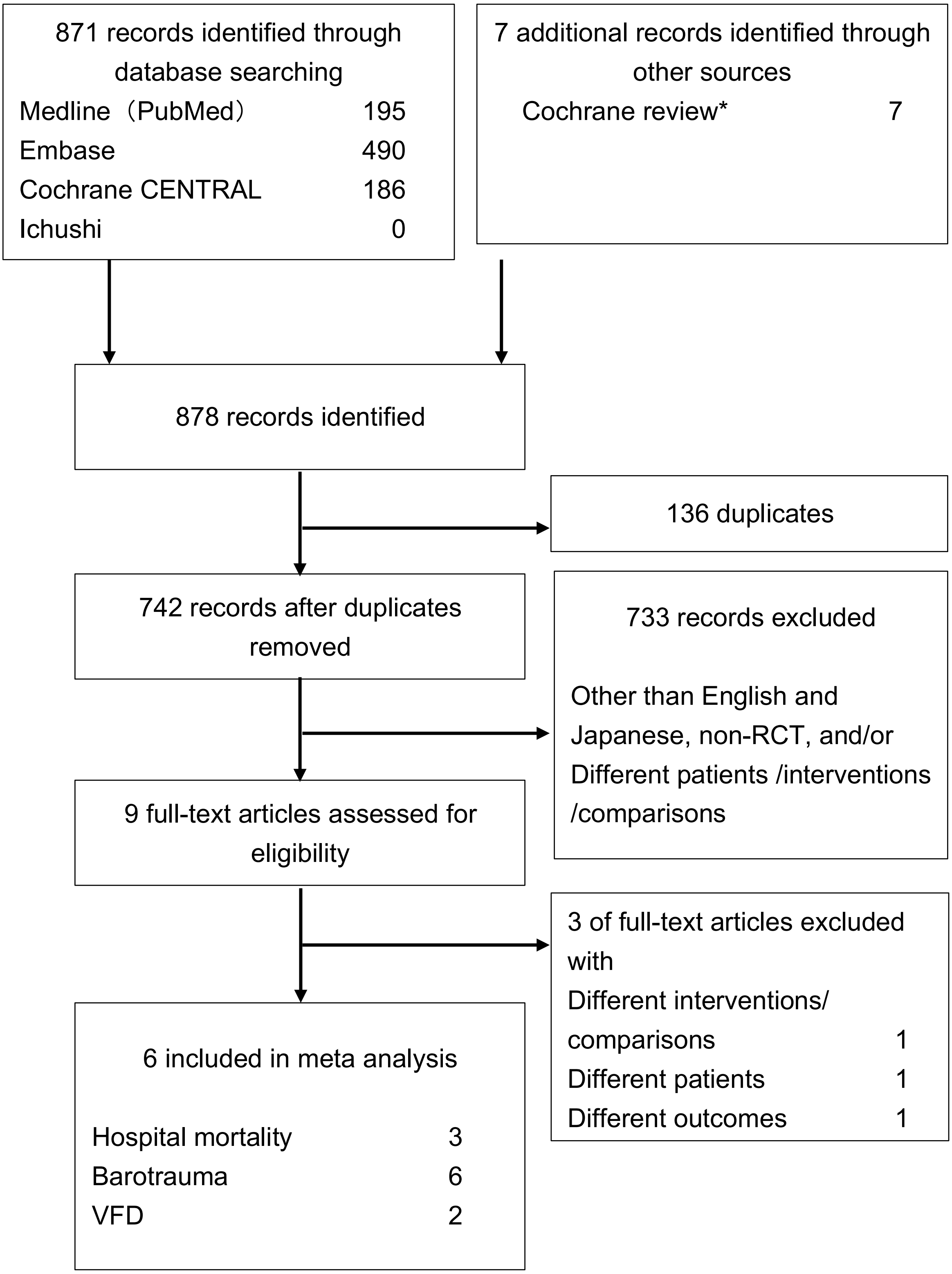

CQ05

Risk of bias table　mortality

mortality		Short term mortality		risk of bias		not serious (0)			
番号	著者名 発表年（Forest plot表示）	risk of bias評価							
		ランダム割付順番の生成 random sequence generation	割り付けの隠蔽化 allocation concealment	ブラインド blinding 研究参加者と治療提供者 participants and personnel	ブラインド blinding アウトカム評価者 outcome assessors	不完全なアウトカムデータ incomplete outcome data	選択されたアウトカムの報告 selective outcome reporting	その他のバイアス Other sources of bias	研究内でのバイアスのリスク Risk of bias within a study
1	Brower 2004	Low risk	Low risk	Low risk	Low risk	Low risk	High risk	High risk	Unclear risk
2	Meade 2008	Low risk	Low risk	Low risk	Low risk	Low risk	Low risk	Low risk	Low risk
3	Mercat 2008	Low risk	Low risk	Low risk	Low risk	Low risk	Low risk	Low risk	Low risk
番号	著者名 発表年（Forest plot表示）	risk of biasコメント							
1	Brower 2004	乱数表を使ったか否かの説明なし	隠蔽化されている	死亡というoutcomeに影響しない	死亡というoutcomeに影響しない	100%フォローされた	supplementary appendix 1で示されているoutcomeと論文中のoutcomeの記載に不一致がある	死亡数が144で中断	2つのhigh riskがあり，看過できない
2	Meade 2008	中央システムでランダム化	allocation concealmentされている	死亡というoutcomeに影響しない	死亡というoutcomeに影響しない	100%フォローされた	100%報告されている	年齢・敗血症割合ともに差は大きくない	全てがlow riskのため
3	Mercat 2008	方法は不明であるが中央でランダム割付と記されている	中央割付である	死亡というoutcomeに影響しない	死亡というoutcomeに影響しない	欠損は両群あわせて1であり、問題ない	trial registrationがされ予め決められており、全てのoutcomeが報告されている	studyが中断されているが死亡数が200以上である	全てがlow riskのため

CQ05

Risk of bias table　VFD

Outcome		VFD		risk of bias		not serious (0)			
番号	著者名 発表年<br>(Forest plot表示)	risk of bias評価							
		ランダム割付順番の生成<br>random sequence generation	割り付けの隠蔽化<br>allocation concealment	ブラインド<br>blinding		不完全なアウトカムデータ<br>incomplete outcome data	選択されたアウトカムの報告<br>selective outcome reporting	その他のバイアス<br>Other sources of bias	研究内でのバイアスのリスク<br>Risk of bias within a study
				研究参加者と治療提供者<br>participants and personnel	アウトカム評価者<br>outcome assessors				
1	Brower 2004	Low risk	Low risk	High risk	Low risk	Low risk	High risk	Low risk	Unclear risk
2	Villar 2006	Low risk	Low risk	High risk	Low risk	Low risk	Unclear risk	High risk	Unclear risk
番号	著者名 発表年<br>(Forest plot表示)	risk of biasコメント							
1	Brower 2004	ランダム化されているが方法が未記載	隠蔽化されている	Blindされていないため、抜管という判断にbiasが入る余地がある	VFDというoutcomeの評価に影響するとは考えられない.	100%フォローされた	supplementary appendix 1で示されているoutcomeと論文中のoutcomeの記載に不一致がある	studyが中断されているが、標本数が500以上.	high riskが2項目あるため
2	Villar 2006	封筒法	隠蔽化されている	Blindされていないため、抜管の判断にBiasが入る余地がある	VFDというoutcomeに影響しない	randomizationに失敗した施設の患者を除外したため、問題ない	pre-registrationについての記述がない	イベント数が98でstudyが中断	high riskが2項目あるため

CQ05
Risk of bias table Barotrauma

Outcome		barotrauma		risk of bias		not serious (0)			
番号	著者名 発表年（Forest plot表示）	risk of bias評価							
		ランダム割付順番の生成 random sequence generation	割り付けの隠蔽化 allocation concealment	ブラインド blinding		不完全なアウトカムデータ incomplete outcome data	選択されたアウトカムの報告 selective outcome reporting	その他のバイアス Other sources of bias	研究内でのバイアスのリスク Risk of bias within a study
				研究参加者と治療提供者 participants and personnel	アウトカム評価者 outcome assessors				
1	Amato 1998	Low risk	Low risk	Low risk	Unclear risk	Low risk	Low risk	High risk	Low risk
2	Brower 2004	Low risk	Low risk	Low risk	High risk	Low risk	High risk	High risk	Unclear risk
3	Huh 2009	Low risk	Unclear risk	Low risk	Unclear risk	High risk	Low risk	Low risk	Low risk
4	Meade 2008	Low risk	Low risk	Low risk	Unclear risk	Low risk	Low risk	Low risk	Low risk
5	Mercat 2008	Low risk	Low risk	Low risk	Unclear risk	Low risk	Low risk	High risk	Low risk
6	Villar 2006	Low risk	Low risk	Low risk	Unclear risk	Low risk	Unclear risk	High risk	Low risk

番号	著者名 発表年（Forest plot表示）	risk of biasコメント							
1	Amato 1998	ランダム化の方法の情報が不十分	sealed envelopeで1:1にrandomizeされている	換気量の違いをblindすることはできないがoutcomeに開胸は無いと思われる	outcomeの評価に主観が入りうる	100%フォローされた	100%報告された	死亡数が53でstudyが中断されている	全項目ほぼLow risk
2	Brower 2004	ランダム化の方法が未記載	隠蔽化されている	圧損傷というoutcomeに影響しない	画像での評価のため、主観が入りうる	100%フォローされた	supplementary appendix 1で示されているoutcomeと論文中のoutcomeの記載に不一致がある	イベント数が55で中断	全項目ほぼLow risk
3	Huh 2009	方法は不明であるがランダム割り付けしたと記されている	ランダム化の詳細の記載がなく、判断できない	肺合併症というoutcomeに影響しない	評価表の記載が無く、主観が入りうる	PEEP-only groupで10%が脱落	100%報告された	研究の中断なし	全項目ほぼLow risk
4	Meade 2008	中央システムでランダム化	Concealed randomizationされている	肺合併症というoutcomeに影響しない	評価表の記載が無く、主観が入りうる	100%フォローされた	100%報告された	7人の患者がwithdrawしているが、outcomeに影響はないと考えられる	全項目ともLow risk
5	Mercat 2008	方法は不明であるが中央でランダム割付と記されている	中央割付である	研究の主要な職員には盲検化がなされていないと考えられるが、それがアウトカムに影響するとは考えられない	評価表の記載が無く、主観が入りうる	欠損は両群あわせて1であり、問題ない	事前登録がされ予め決められていた	studyが中断されており、総event数が48でしかないためriskは高い	1項目high riskがあるものの、多くの項目がlow riskであるから
6	Villar 2006	封筒法	隠蔽化されている	肺合併症というoutcomeに影響しない	評価表の記載が無く、主観が入りうる	ランダム化に失敗した施設の患者を除外したため、問題ない	事前登録についての記述がない	studyが中断されている	1項目high riskがあるものの、多くの項目がlow riskであるから

CQ05 Risk of bias summary, Risk of bias graph

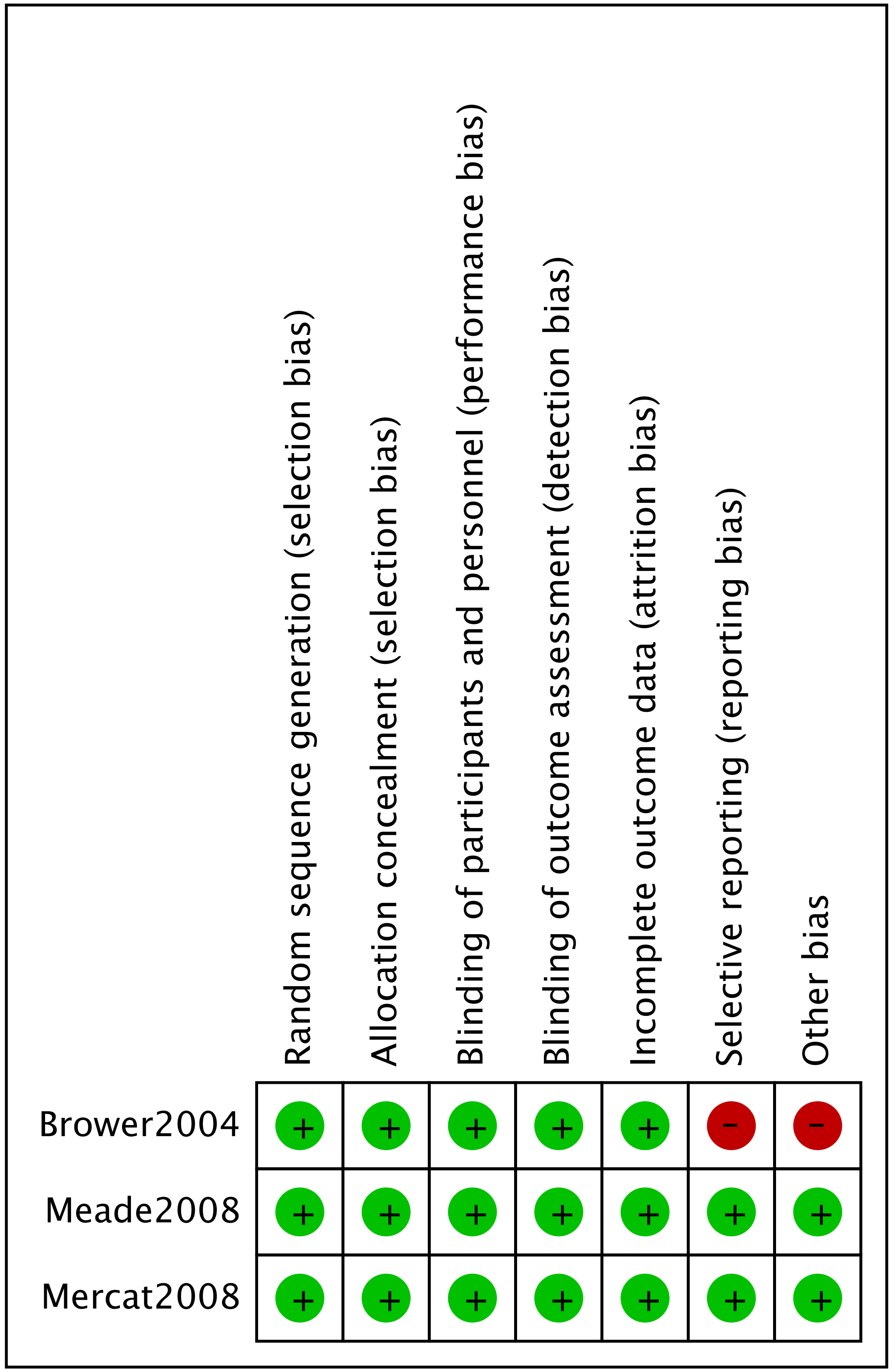

Short term mortality

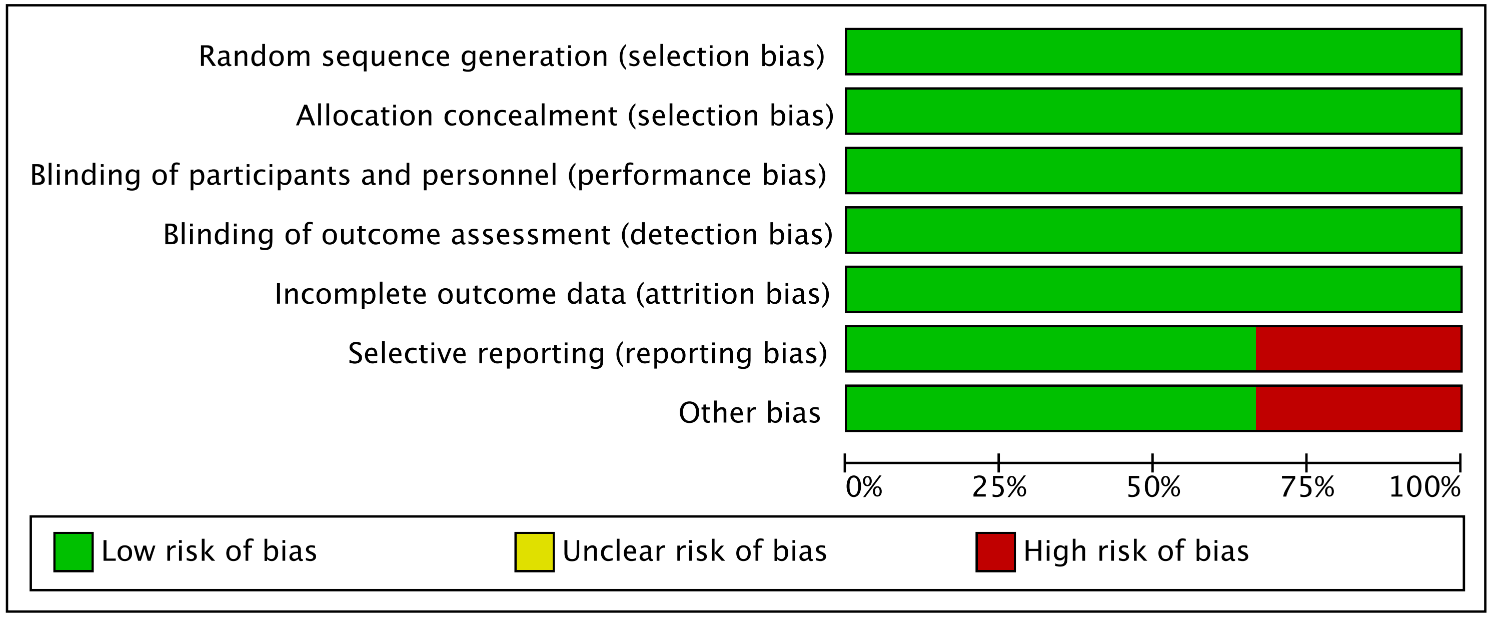

CQ05 Risk of bias summary, Risk of bias graph

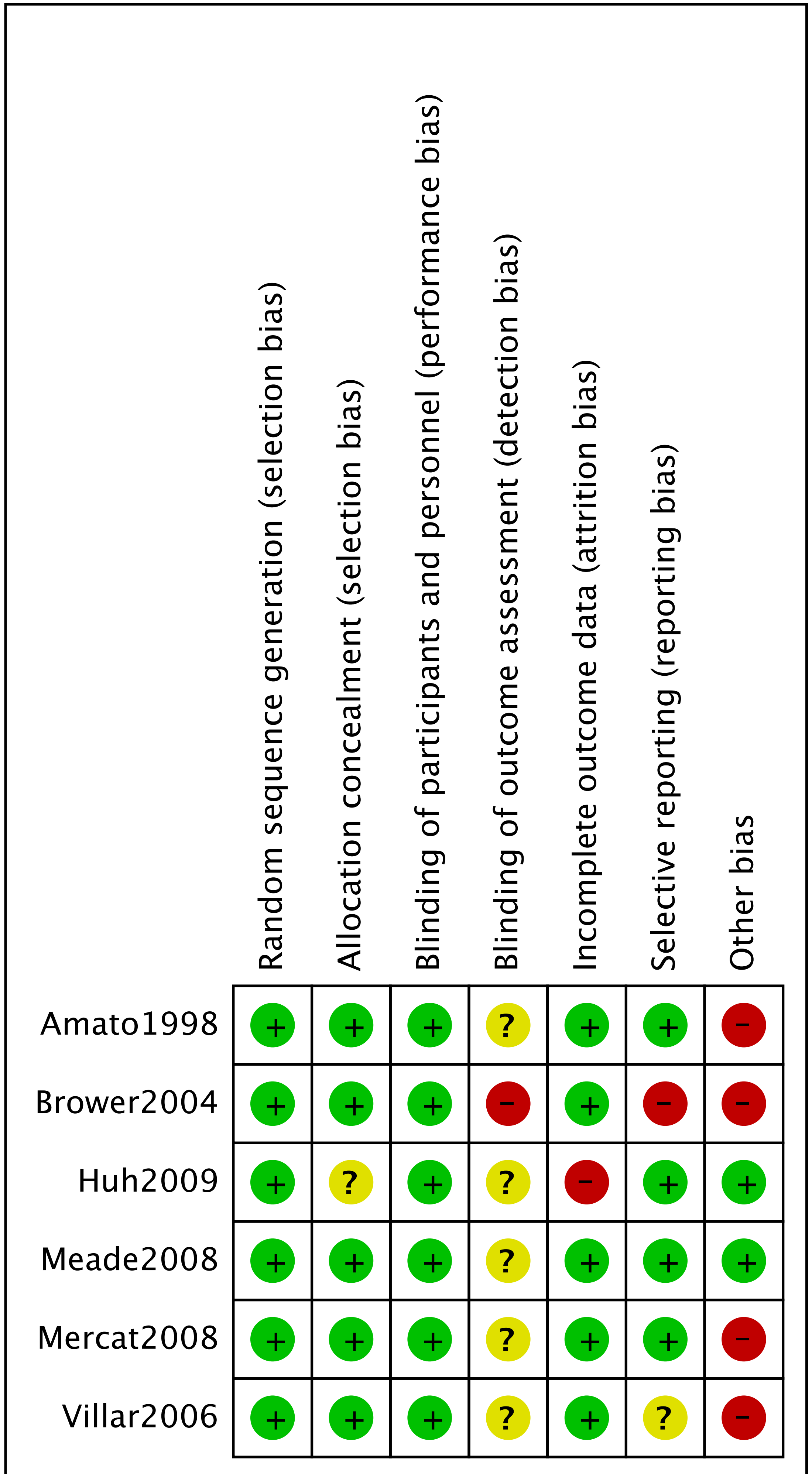

Barotrauma

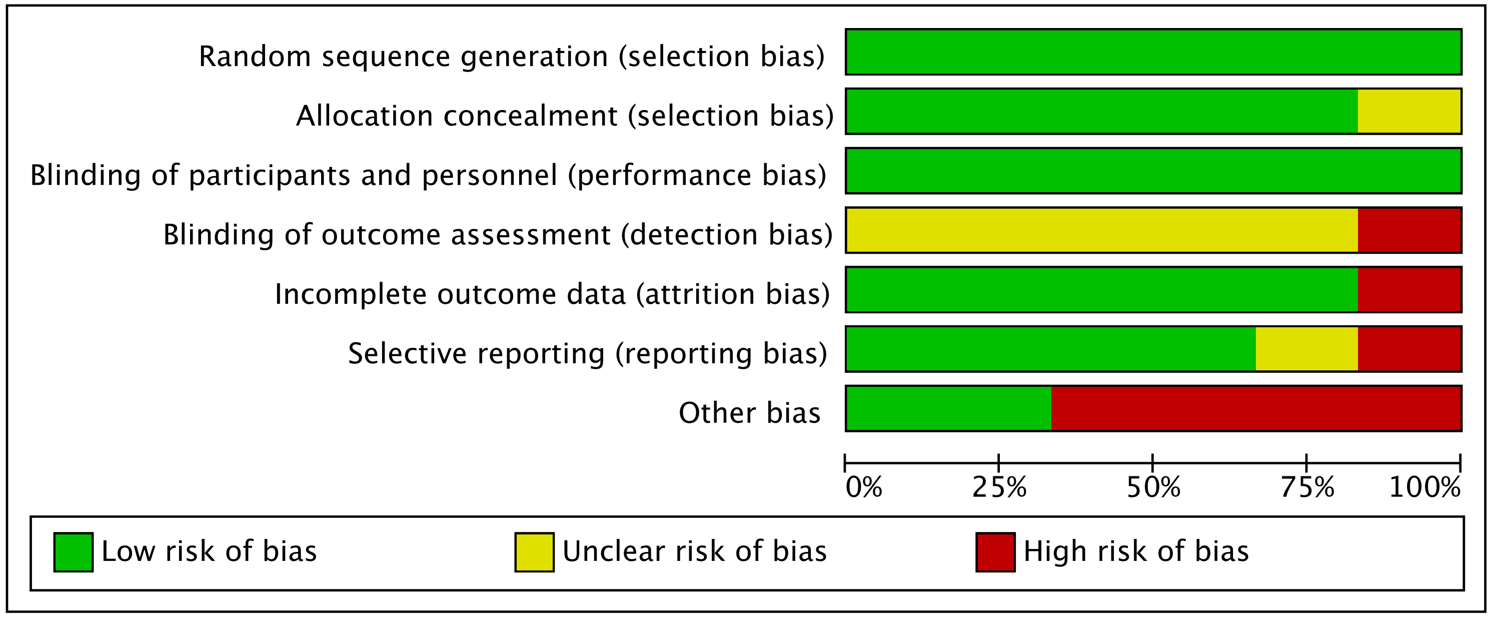

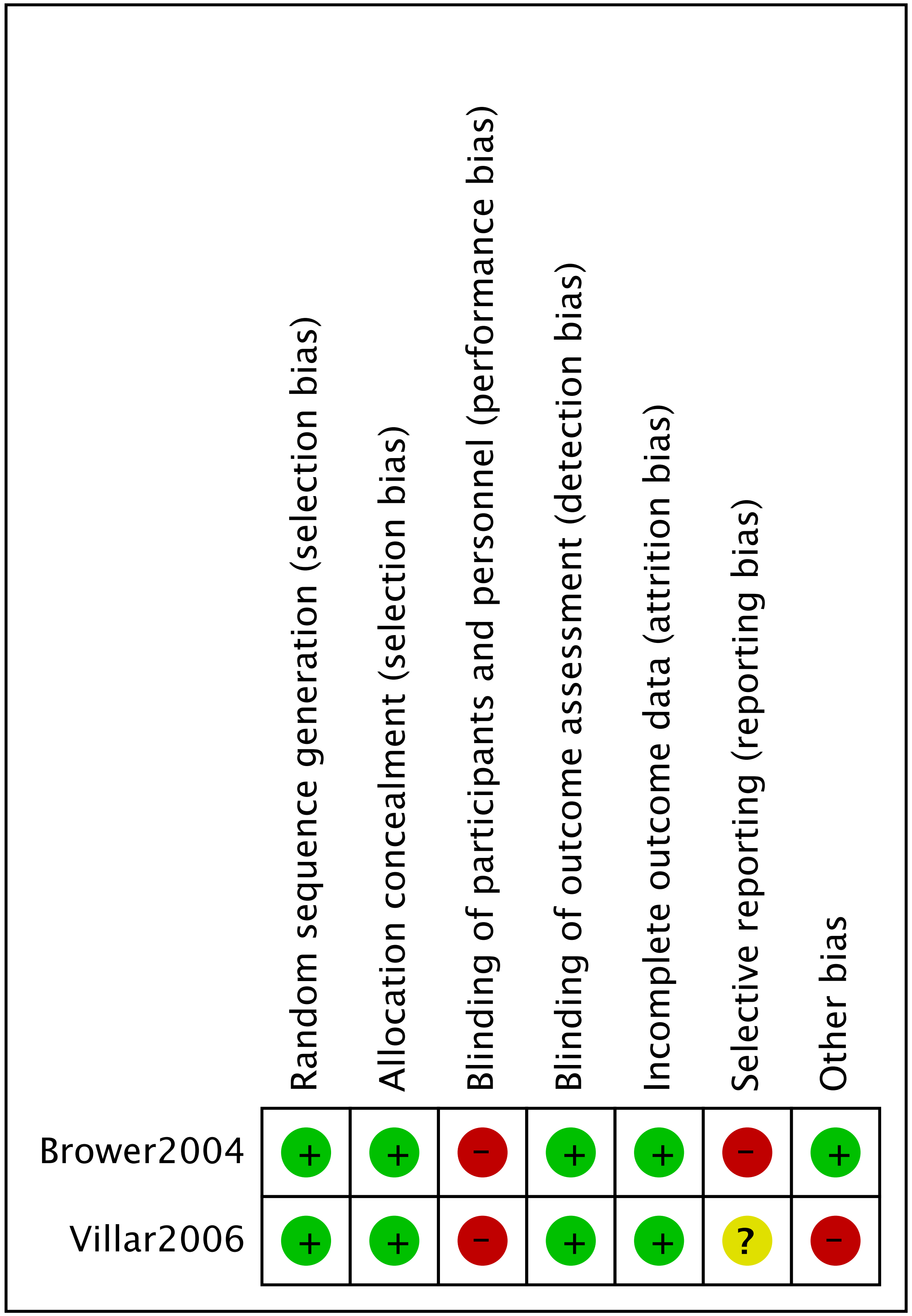
Random sequence generation (selection bias)
Allocation concealment (selection bias)
Blinding of participants and personnel (performance bias)
Blinding of outcome assessment (detection bias)
Incomplete outcome data (attrition bias)
Selective reporting (reporting bias)
Other bias
Brower2004
Villar2006
+
−
?

VFD

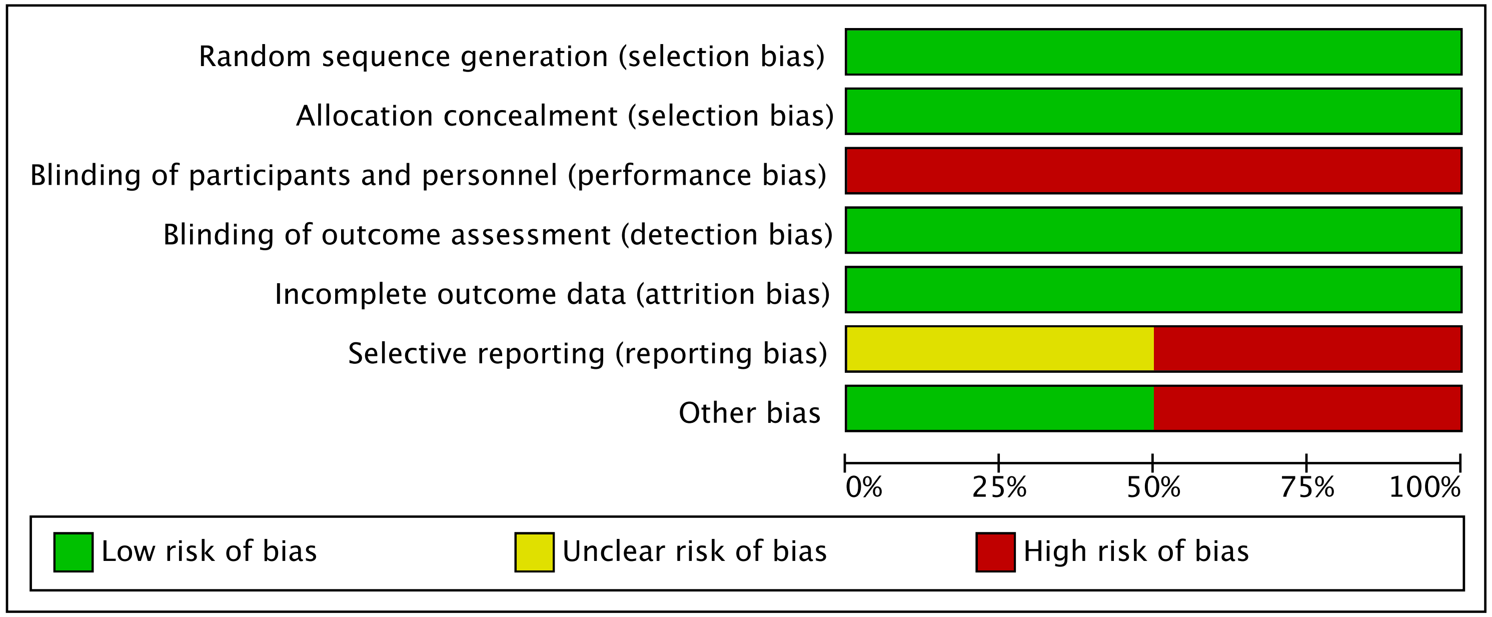
Random sequence generation (selection bias)
Allocation concealment (selection bias)
Blinding of participants and personnel (performance bias)
Blinding of outcome assessment (detection bias)
Incomplete outcome data (attrition bias)
Selective reporting (reporting bias)
Other bias
0%
25%
50%
75%
100%
Low risk of bias
Unclear risk of bias
High risk of bias

Short term mortality

Study or Subgroup	High PEEP Events	High PEEP Total	Low PEEP Events	Low PEEP Total	Weight	Risk Ratio M-H, Random, 95% CI
Brower2004	76	276	68	273	15.5%	1.11 [0.83, 1.46]
Meade2008	173	475	205	508	48.4%	0.90 [0.77, 1.06]
Mercat2008	136	385	149	382	36.0%	0.91 [0.75, 1.09]
**Total (95% CI)**		**1136**		**1163**	**100.0%**	**0.93 [0.83, 1.04]**
Total events	385		422			

Heterogeneity: $Tau^2 = 0.00$; $Chi^2 = 1.67$, df = 2 (P = 0.43); $I^2 = 0\%$
Test for overall effect: Z = 1.24 (P = 0.22)

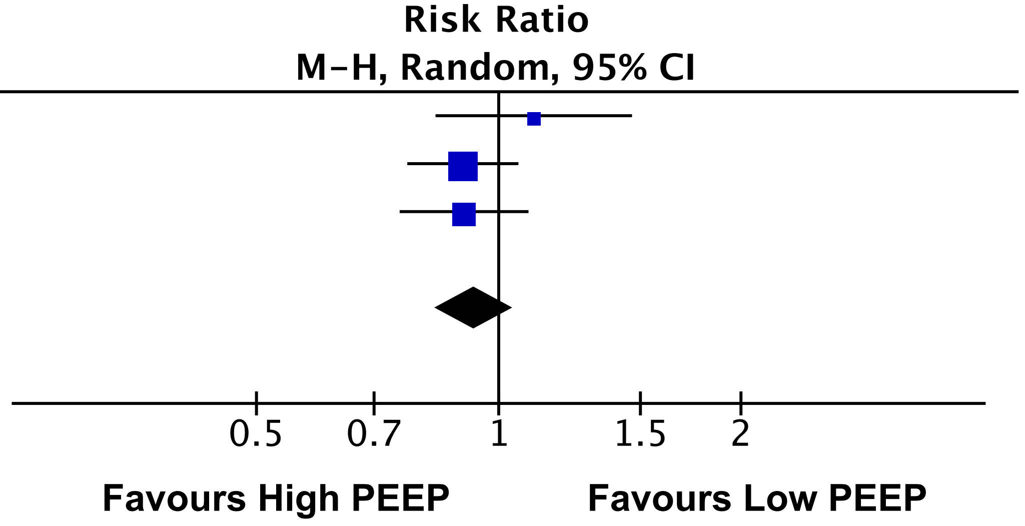

Barotrauma

Study or Subgroup	High PEEP Events	High PEEP Total	Low PEEP Events	Low PEEP Total	Weight	Risk Ratio M-H, Random, 95% CI
Amato1998	2	29	10	24	6.3%	0.17 [0.04, 0.68]
Brower2004	30	276	27	273	26.6%	1.10 [0.67, 1.80]
Huh2009	3	30	3	27	5.6%	0.90 [0.20, 4.09]
Meade2008	53	475	47	508	32.8%	1.21 [0.83, 1.75]
Mercat2008	26	385	22	382	23.9%	1.17 [0.68, 2.03]
Villar2006	2	50	4	45	4.8%	0.45 [0.09, 2.34]
**Total (95% CI)**		**1245**		**1259**	**100.0%**	**0.97 [0.66, 1.42]**
Total events	116		113			

Heterogeneity: $Tau^2 = 0.08$; $Chi^2 = 8.33$, df = 5 (P = 0.14); $I^2 = 40\%$
Test for overall effect: Z = 0.17 (P = 0.87)

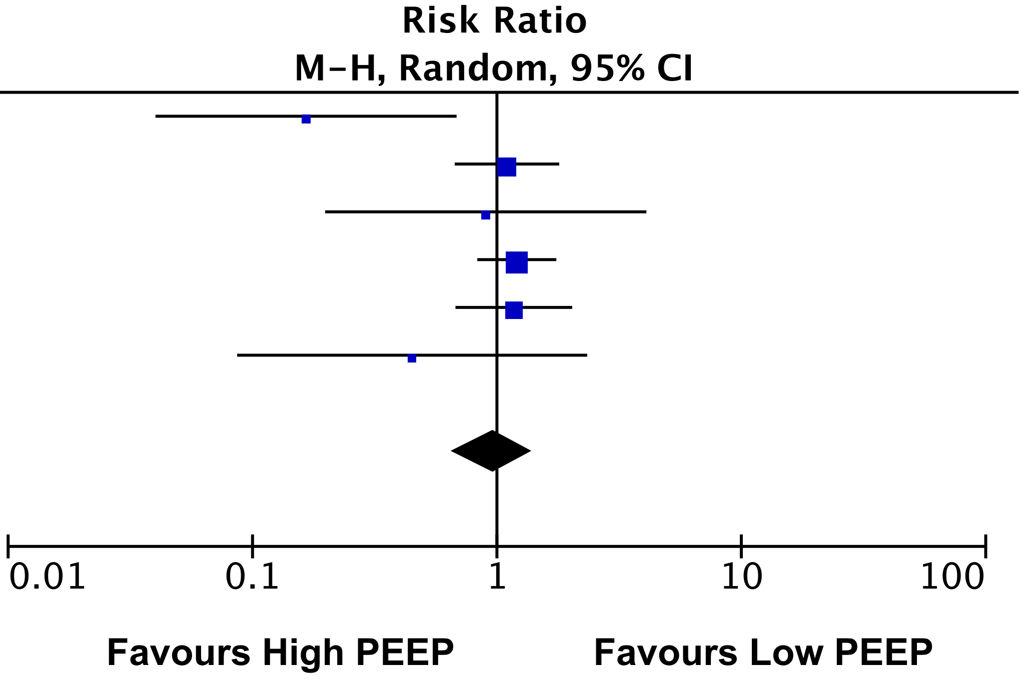

VFD

Study or Subgroup	High PEEP Mean	High PEEP SD	High PEEP Total	Low PEEP Mean	Low PEEP SD	Low PEEP Total	Weight	Mean Difference IV, Random, 95% CI
Brower2004	13.8	10.6	276	14.5	10.4	273	53.7%	-0.70 [-2.46, 1.06]
Villar2006	10.9	9.4	50	6	7.9	45	46.3%	4.90 [1.42, 8.38]
**Total (95% CI)**			**326**			**318**	**100.0%**	**1.89 [-3.58, 7.36]**

Heterogeneity: $Tau^2 = 13.70$; $Chi^2 = 7.92$, df = 1 (P = 0.005); $I^2 = 87\%$
Test for overall effect: Z = 0.68 (P = 0.50)

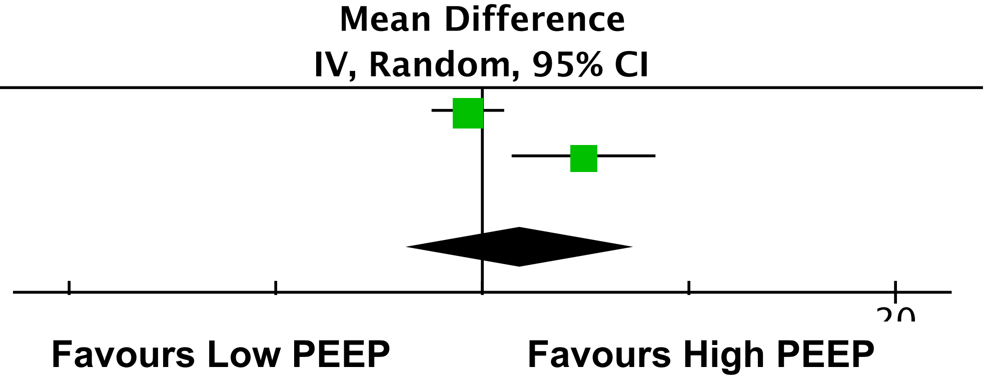

subgroup analysis
Short term mortality (All RCTs)

Study or Subgroup	High PEEP Events	High PEEP Total	Low PEEP Events	Low PEEP Total	Weight	Risk Ratio M-H, Random, 95% CI
Talmor2008	5	30	12	31	2.8%	0.43 [0.17, 1.07]
Villar2006	16	50	24	45	8.6%	0.60 [0.37, 0.98]
Amato1998	13	29	17	24	8.8%	0.63 [0.39, 1.02]
Meade2008	173	475	205	508	29.7%	0.90 [0.77, 1.06]
Mercat2008	136	385	149	382	27.0%	0.91 [0.75, 1.09]
Brower2004	76	276	68	273	18.3%	1.11 [0.83, 1.46]
Huh2009	12	30	9	27	4.8%	1.20 [0.60, 2.39]
**Total (95% CI)**		**1275**		**1290**	**100.0%**	**0.87 [0.74, 1.02]**
Total events	431		484			

Heterogeneity: $Tau^2 = 0.02$; $Chi^2 = 9.96$, df = 6 (P = 0.13); $I^2 = 40\%$
Test for overall effect: Z = 1.70 (P = 0.09)

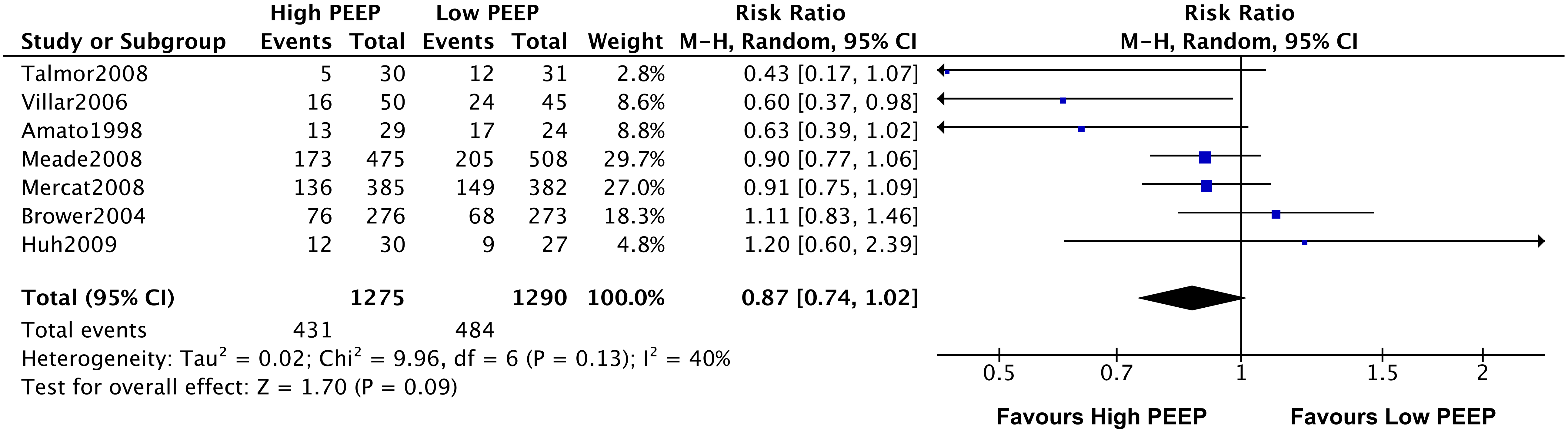

subgroup analysis
Short term mortality (All RCTs)
patients with P/F ratio ≤ 200

Study or Subgroup	High PEEP Events	High PEEP Total	Low PEEP Events	Low PEEP Total	Weight	Risk Ratio M-H, Random, 95% CI
Amato1998	13	29	17	24	5.8%	0.63 [0.39, 1.02]
Brower2004	0	0	0	0		Not estimable
Huh2009	14	30	15	27	5.1%	0.84 [0.50, 1.40]
Meade2008	155	408	187	419	49.5%	0.85 [0.72, 1.00]
Mercat2008	114	323	132	318	34.1%	0.85 [0.70, 1.04]
Talmor2008	0	0	0	0		Not estimable
Villar2006	16	50	24	45	5.6%	0.60 [0.37, 0.98]
**Total (95% CI)**		**840**		**833**	**100.0%**	**0.82 [0.73, 0.92]**
Total events	312		375			

Heterogeneity: $Tau^2 = 0.00$; $Chi^2 = 3.04$, df = 4 (P = 0.55); $I^2 = 0\%$
Test for overall effect: Z = 3.39 (P = 0.0007)

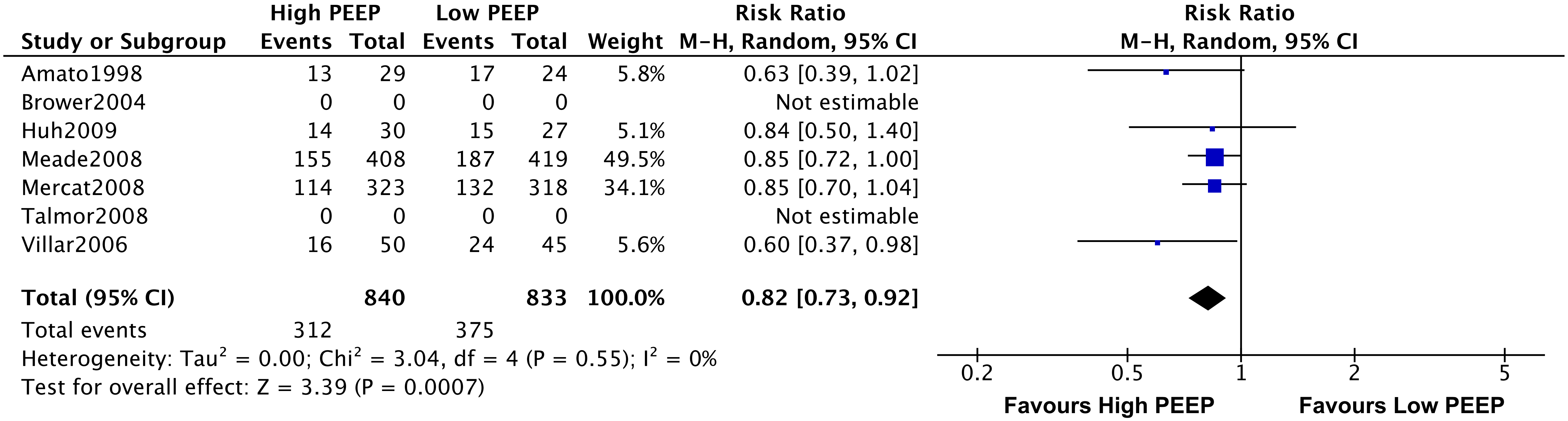

subgroup analysis
Short term mortality (RCTs were excluded, if other than PEEP might influence the study outcome)
patients with P/F ratio ≤ 200

	High PEEP		Low PEEP			Risk Ratio
Study or Subgroup	Events	Total	Events	Total	Weight	M-H, Random, 95% CI
Amato1998	13	29	17	24		Not estimable
Brower2004	0	0	0	0		Not estimable
Huh2009	12	30	9	27		Not estimable
Meade2008	155	408	187	419	59.2%	0.85 [0.72, 1.00]
Mercat2008	114	323	132	318	40.8%	0.85 [0.70, 1.04]
Talmor2008	0	0	0	0		Not estimable
Villar2006	16	50	24	45		Not estimable
**Total (95% CI)**		**731**		**737**	**100.0%**	**0.85 [0.75, 0.96]**
Total events	269		319			

Heterogeneity: $Tau^2 = 0.00$; $Chi^2 = 0.00$, df = 1 (P = 0.99); $I^2 = 0\%$
Test for overall effect: Z = 2.52 (P = 0.01)

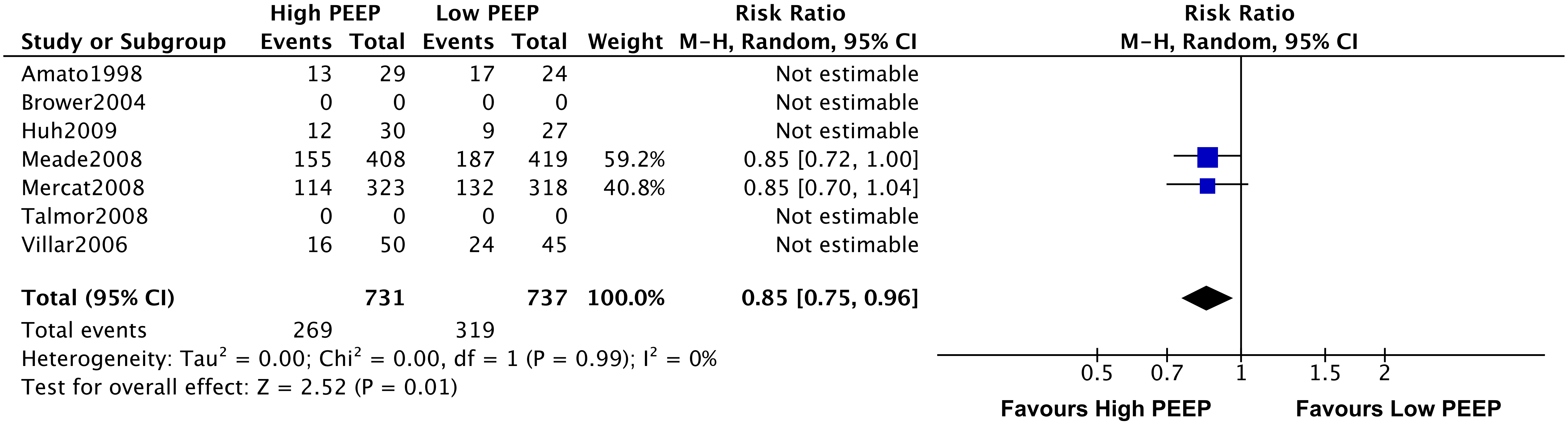

Summary of findings

High compared to low PEEP for ARDS patients

Setting :
Intervention : high PEEP
Comparison : low PEEP

Outcomes	Anticipated absolute effects (95% CI)		Relative effect (95% CI)	No of participants (studies)	Quality of the evidence (GRADE)	Comments
	Risk with low PEEP	Risk with high PEEP				
Hospital mortality	**Study population**		**RR 0.93** (0.83 - 1.04)	2299 (3 RCTs)	⊕⊕⊕◯ MODERATE [1]	
	363 / 1000	**337/ 1000** (301 - 377)				
	**Low**					
	380 / 1000	**353 / 1000** (315 - 395)				
	**High**					
	560 / 1000	**521 / 1000** (465 - 582)				
Barotrauma	**Study population**		**RR 0.97** (0.66 - 1.42)	2504 (6 RCTs)	⊕⊕⊕◯ MODERATE [1]	
	90 / 1000	**87 / 1000** (59 - 127)				
	**Low**					
	38 / 1000	**37 / 1000** (25 - 54)				
	**High**					
	120 / 1000	**116 / 1000** (79 - 170)				
VFD	Mean 10.56 days	1.89 days more MD (3.58 fewer - 7.36 more)	-	644 (2 RCTs)	⊕⊕⊕◯ MODERATE [2]	

***The risk in the intervention group** (and its 95% confidence interval) is based on the assumed risk in the comparison group and the **relative effect** of the intervention (and its 95% CI).

CI: Confidence interval; **RR:** Risk ratio ; **MD:** Mean difference

GRADE Working Group grades of evidence
High quality: We are very confident that the true effect lies close to that of the estimate of the effect
Moderate quality: We are moderately confident in the effect estimate: The true effect is likely to be close to the estimate of the effect, but there is a possibility that it is substantially different
Low quality: Our confidence in the effect estimate is limited: The true effect may be substantially different from the estimate of the effect
Very low quality: We have very little confidence in the effect estimate: The true effect is likely to be substantially different from the estimate of effect

1. Three trials were stopped early based on pre-specified efficacy stopping criteria.
2. The heterogeneity is significant with I^2=87%, p=0.005.

CQ5:

Question: How should positive end-expiratory pressure (PEEP) be set in adult patients with ARDS?

Quality assessment							No of patients		Effect		Quality	Importance
No of studies	Study design	Risk of bias	Inconsistency	Indirectness	Imprecision	Other considerations	High PEEP	Low PEEP	relative (95% CI)	absolute (95% CI)		
short term mortality												
3	randomized trials	serious[1]	not serious	not serious	not serious	none	385/1136 (33.9%)	422/1163 (36.3%)	RR 0.93 (0.83 to 1.04)	25 fewer per 1000 (from 15 more to 62 fewer)	⨁⨁⨁◯ MODERATE[1]	CRITICAL
								38.0%		27 fewer per 1000 (from 15 more to 65 fewer)		
								56.0%		39 fewer per 1000 (from 22 more to 95 fewer)		
Barotrauma												
6	randomized trials	serious[1]	not serious	not serious	not serious	none	116/1245 (9.3%)	113/1259 (9.0%)	RR 0.97 (0.66 to 1.42)	3 fewer per 1000 (from 38 more to 31 fewer)	⨁⨁⨁◯ MODERATE 1	IMPORTANT
								3.8%		1 fewer per 1000 (from 16 more to 13 fewer)		
								12.0%		4 fewer per 1000 (from 50 more to 41 fewer)		
VFD												
2	randomized trials	not serious	serious[2]	not serious	not serious	none	10.56	12.46	-	MD **1.89 more** (3.58 fewer to 7.36 more)	⨁⨁⨁◯ MODERATE[2]	IMPORTANT

MD- mean difference, RR-Relative risk

1. Three trials were stopped early based on pre-specified efficacy stopping rule.
2. The heterogeneity is significant with I2=87% p=0.005

10. Evidence-to-Decision table

<table>
<tr><td colspan="5">CQ5 : What is the optimal positive end-expiratory pressure (PEEP) in adult patients with ARDS?</td></tr>
<tr><td colspan="5">POPULATION : ADULT PATIENTS WITH ARDS</td></tr>
<tr><td colspan="5">INTERVENTION : HIGH PEEP</td></tr>
<tr><td colspan="2">CRITERIA</td><td>JUDGEMENT</td><td>RESEARCH EVIDENCE</td><td>ADDITIONAL CONSIDERATIONS</td></tr>
<tr><td>PROBLEM</td><td>Is the problem a priority?</td><td>○No
○Probably no
●Probably yes
○Yes

○Varies
○Don’t know</td><td>It is well known that PEEP prevents atelectasis and improves oxygenation in patients undergoing mechanical ventilation. It has been suggested that PEEP not only improves oxygenation but also prevents ventilator-induced lung injury by recruiting alveoli collapsed by inflammation and exudative fluid in patients with ARDS.
The priority of this issue is thought to be high although the optimal PEEP level is undefined.</td><td>See note 3 for the definitions of “higher PEEP” and “lower PEEP”</td></tr>
<tr><td rowspan="3">DESIRABLE AND UNDESIRABLE EFFECTS</td><td>What is the overall certainty of the evidence of effects?</td><td>○Very low
○Low
●Moderate
○High

○No included studies</td><td rowspan="3">The relative importance or values of the main outcomes of interest:
<table><tr><td>Outcome</td><td>Relative importance</td><td>Certainty of the evidence (GRADE)</td></tr><tr><td>Mortality(note 1</td><td>Critical</td><td>⊕⊕⊕◯
Moderate</td></tr><tr><td>Barotrauma</td><td>Critical</td><td>⊕⊕⊕◯
Moderate</td></tr><tr><td>VFD(note 2</td><td>Critical</td><td>⊕⊕⊕◯
Moderate</td></tr></table></td><td></td></tr>
<tr><td>Is there important uncertainty about or variability in how much people value the main outcomes?</td><td>○Important uncertainty or variability
○Possibly important uncertainty or variability
○Possibly no important uncertainty or variability
●No important uncertainty or variability

○No known undesirable outcomes</td><td></td></tr>
<tr><td>How substantial are the desirable anticipated effects?</td><td>○Trivial
○Small
○Moderate
○Large
------------------</td><td></td></tr>
</table>

<table>
<tr>
<td rowspan="3"></td>
<td></td>
<td>●Varies
○Don't know</td>
<td rowspan="3">
Summary of findings:
<table>
<tr><th>Outcome</th><th>High PEEP</th><th>Low PEEP</th><th>Absolute effect (95% CI)</th><th>Relative effect (RR) (95% CI)</th></tr>
<tr><td rowspan="3">Mortality</td><td>363 / 1000</td><td>337 / 1000 (301 ～ 377)</td><td>25 fewer per 1000 (from 15 more to 62 fewer)</td><td rowspan="3">RR 0.93
(0.83 ～ 1.04)</td></tr>
<tr><td>380 / 1000</td><td>353 / 1000 (315 ～ 395)</td><td>27 fewer per 1000 (from 15 more to 65 fewer)</td></tr>
<tr><td>560 / 1000</td><td>521 / 1000 (465 ～ 582)</td><td>39 fewer per 1000 (from 22 more to 95 fewer)</td></tr>
<tr><td rowspan="3">Barotrauma</td><td>90 / 1000</td><td>87 / 1000 (59 ～ 127)</td><td>3 fewer per 1000 (from 38 more to 31 fewer)</td><td rowspan="3">RR 0.97
(0.66 ～ 1.42)</td></tr>
<tr><td>38 / 1000</td><td>37 / 1000 (25 ～ 54)</td><td>1 fewer per 1000 (from 16 more to 13 fewer)</td></tr>
<tr><td>120 / 1000</td><td>116 / 1000 (79 ～ 170)</td><td>4 fewer per 1000 (from 50 more to 41 fewer)</td></tr>
<tr><td>VFD</td><td>Average 10.56 days</td><td>Average12.46 days</td><td>MD 1.89 more
(3.58 fewer to 7.36 more)</td><td></td></tr>
</table>
Summary: There are no differences in hospital mortality, incidence of barotrauma or ventilator-free days (VFD) comparing patient groups receiving higher PEEP and lower PEEP levels (hospital mortality RR 0.93; 95%CI 0.83-1.04, barotrauma RR 0.97; 95%CI 0.66-1.42, VFD 1.89 days more; 95%CI -3.58～7.36).
</td>
<td></td>
</tr>
<tr>
<td>How substantial are the undesirable anticipated effects?</td>
<td>○Large
○Moderate
○Small
○Trivial

○Varies
●Don't know</td>
<td></td>
</tr>
<tr>
<td>Does the balance between desirable and undesirable effects favor the intervention or the comparison?</td>
<td>○Favors the comparison
○Probably favors the comparison
○Does not favor either the intervention or the comparison
○Probably favors the intervention
○Favors the intervention

●Varies
○Don't know</td>
<td></td>
</tr>
<tr>
<td rowspan="2">RESOURCES REQUIRED</td>
<td>How large are the resource requirements (costs)?</td>
<td>○Large costs
○Moderate costs
●Negligible costs and savings
○Moderate savings
○Large savings

○Varies
○Don't know</td>
<td>The costs of increasing PEEP is negligible.</td>
<td></td>
</tr>
<tr>
<td>Does the cost-effectiveness of the intervention favor the intervention or the comparison?</td>
<td>○Favors the comparison
○Probably favors the comparison
○Does not favor either the intervention or the comparison
○Probably favors the</td>
<td>As there is no difference in the number of VFD comparing groups receiving high and low PEEP, no differences in costs or resources are expected.</td>
<td></td>
</tr>
</table>

		intervention<br>○Favors the intervention<br>------------------<br>●Varies<br>○No included studies		
**EQUITY**	**What would be the impact on health equity?**	○Reduced<br>○Probably reduced<br>●Probably no impact<br>○Probably increased<br>○Increased<br>------------------<br>○Varies<br>○Don't know	Special medical facilities or equipment are not required to increase PEEP.	
**ACCEPTABILITY**	**Is the intervention acceptable to key stakeholders?**	○No<br>○Probably no<br>●Probably yes<br>○Yes<br>------------------<br>○Varies<br>○Don't know		
**FEASIBILITY**	**Is the intervention feasible implement?**	○No<br>○Probably no<br>○Probably yes<br>●Yes<br>------------------<br>○Varies<br>○Don't know	Special medical facilities or equipment are not required to increase PEEP.	

Recommendations

CQ5：**What is the optimal positive end-expiratory pressure (PEEP) in adult patients with ARDS?**					
**Balance of consequences**	Undesirable consequences *clearly outweigh* desirable consequences in most settings	Undesirable consequences *probably outweigh* desirable consequences in most settings	The balance between desirable and undesirable consequences is closely *balanced or uncertain*	Desirable consequences *probably outweigh* undesirable consequences in most settings	Desirable consequences *clearly outweigh* undesirable consequences in most settings
**Judgement**	○	○	●	○	○

**Type of recommendation**	Strong recommendation against the intervention	Conditional recommendation against the intervention	Conditional recommendation for either the intervention or the comparison	Conditional recommendation for the intervention
**Judgement**	○	○	●	○
**Recommendation**	**We suggest using PEEP within the range of plateau pressures less than or equal to 30cmH_2O, without compromising hemodynamics (Grade 2B, strength of recommendation "weak recommendation" / Quality of evidence "moderate"). We also suggest using higher PEEP levels in patients with moderate to severe ARDS (Grade 2B, Strength of recommendation "weak recommendation" / Quality of evidence "moderate").**<br><br>●Supplementary statements: Increasing PEEP levels may result in high plateau pressures, hypotension or a decrease in tidal volume. Close monitoring of hemodynamics and other parameters is necessary when high PEEP levels are used.			
**Justification**	**Question:** What is the optimal positive end-expiratory pressure (PEEP) in adult patients with ARDS?<br>**Patients:** ADULT PATIENTS WITH ARDS<br>**Interventions:** High PEEP<br>**Comparison:** Low PEEP<br>**Outcomes:** Short Term Mortality[(note 1]、Barotrauma、VFD<br><br>**Summary of the evidence:** We conducted a systematic review and included seven randomized clinical trials, which show that there are no differences in hospital mortality, incidence of barotrauma or ventilator-free days (VFD) comparing patient groups receiving higher PEEP and lower PEEP levels (hospital mortality RR 0.93; 95%CI 0.83-1.04, barotrauma RR 0.97; 95%CI 0.66-1.42, VFD 1.89 days more; 95%CI -3.58～7.36).<br>Only three trials (Brower2004、Meade2008、Mercat2008) were included in the analysis for hospital mortality because trials that had interventions with potential effects on the outcome other than PEEP in the experimental groups were excluded[1-3].<br><br>**Quality of the evidence:** Among the seven studies included in this systematic review, five (Amato 1998, Brower 2004, Mercat 2008, Talmor 2008 and Villar 2006) were terminated early[1, 3-6] and three (Amato 1998, Talmor 2008 and Villar 2006) had inadequate sample sizes. Therefore, the overall quality of evidence for each outcome is considered "moderate" after downgrading by one level.<br><br>**Judgement of benefit and harm, resources and cost:** No obvious benefits or harms were identified. There are no direct effects on cost by changing ventilator settings. Hemodynamic changes due to high levels of PEEP needs to be monitored.			

	**Recommendations;** **We suggest using PEEP within the range of plateau pressures less than or equal to 30cmH_2O, without compromising hemodynamics (Grade 2B, strength of recommendation "weak recommendation" / Quality of evidence "moderate"). We also suggest using higher PEEP levels in patients with moderate to severe ARDS (Grade 2B, Strength of recommendation "weak recommendation" / Quality of evidence "moderate").**<br><br>**Additional considerations:** In the panel discussion, the focus was on the higher level of PEEP in patients with moderate to severe ARDS. It was proposed that another panel discussion be held after adding a subgroup analysis including only patients with moderate to severe ARDS. Since the subgroup analysis did not show a significant difference in mortality comparing higher and lower PEEP levels, the recommendation not to use a higher PEEP level routinely was suggested. However, because the PEEP levels used in the lower PEEP groups in Brower 2004, Meade 2008, and Mercat 2008 were not "low" in general, some panelists raised the concern that the recommendation may lead to ventilator settings with unnecessarily low PEEP and there was no consensus to accept the recommendation during the second panel discussion. The final version was approved through an email discussion among panelists.
**Subgroup considerations**	A subgroup analysis comparing the mortality rate of higher PEEP and lower PEEP groups in patients with moderate to severe ARDS (P/F ≤200) showed a significantly lower hospital mortality in the higher PEEP group in both analyses in all trials and the analysis excluding trials that had interventions other than PEEP in experimental groups (RR 0.82, 95%CI 0.73～0.92、RR 0.85, 95%CI 0.75～0.96 respectively).
**Implementation considerations**	The panel meeting concluded that it is appropriate to use the FiO_2/PEEP ladder used in ARDSnetwork 2000 and Brower 2004 to determine the PEEP level required at present [7], as there are no other methods shown to be more practical or better to determine the optimal PEEP level.
**Monitoring and evaluation considerations**	Monitoring of indices related to mechanical ventilation such as oxygenation, ventilation, pressures and volumes is important. High PEEP requires careful monitoring of hemodynamic status.
**Research possibilities**	It is necessary to identify which subgroups benefit from lower PEEP or higher PEEP. Further studies are also required to compare methods to determine the optimal PEEP level for individual patients, rather than compare lower and higher PEEP levels.

Note 1) We used 28-day mortality or ICU mortality as "short term mortality".

Note 2) VFD means the number of days free from mechanical ventilation during the initial 28 days. If the patient expired within 28 days, VFD was counted as zero.

Note 3)：The definitions of "higher PEEP" and "lower PEEP"
Each RCT has its own definitions of "higher PEEP" and "lower PEEP". The definitions of "higher PEEP" and "lower PEEP" in the RCTs included in this systematic review are as follows.

Brower2004 : The PEEP level is predetermined for each required FiO_2 level. PEEP levels in the higher PEEP group are set higher than those in the lower PEEP group for each required FiO_2 (See Part 1 section 9 in published version).

Meade2008：The PEEP level is predetermined for each required FiO_2 level. PEEP levels in the higher PEEP group are set higher than those in the lower PEEP group for each required FiO_2.

PEEP ladder used in lower PEEP group

F_IO_2	0.3	0.4	0.4	0.5	0.5	0.6	0.7	0.7	0.7	0.8	0.9	0.9	0.9	1.0	1.0	1.0	1.0
PEEP	5	5	8	8	10	10	10	12	14	14	14	16	18	18	20	22	24

PEEP ladder used in higher PEEP group

F_IO_2	0.3	0.3	0.4	0.4	0.5	0.5	0.5-0.8	0.8	0.9	1.0
PEEP	12	14	14	16	16	18	20	22	22	22-24

The two FiO_2/PEEP ladders above are based on the ladder used in the ARMA study [7].

Mercat2008：PEEP was set at 5-9 cmH_2O in the lower PEEP group and it set to reach a plateau pressure of 28 to 30 cm H_2O

1. Brower RG, Lanken PN, MacIntyre N, et al. Higher versus lower positive end-expiratory pressures in patients with the acute respiratory distress syndrome. *N Engl J Med* **351**(4): 327-36, 2004. PMID 15269312

2. Meade MO, Cook DJ, Guyatt GH, et al. Ventilation strategy using low tidal volumes, recruitment maneuvers, and high positive end-expiratory pressure for acute lung injury and acute respiratory distress syndrome: a randomized controlled trial. *JAMA* **299**(6): 637-45, 2008. PMID 18270352

3. Mercat A, Richard JC, Vielle B, et al. Positive end-expiratory pressure setting in adults with acute lung injury and acute respiratory distress syndrome: a randomized controlled trial. *JAMA* **299**(6): 646-55, 2008. PMID 18270353

4. Amato MB, Barbas CS, Medeiros DM, et al. Effect of a protective-ventilation strategy on mortality in the acute respiratory distress syndrome. *N Engl J Med* **338**(6): 347-54, 1998. PMID 9449727

5. Talmor D, Sarge T, Malhotra A, et al. Mechanical ventilation guided by esophageal pressure in acute lung injury. *N Engl J Med* **359**(20): 2095-104, 2008. PMID 19001507

6. Villar J, Kacmarek RM, Perez-Mendez L, et al. A high positive end-expiratory pressure, low tidal volume ventilatory strategy improves outcome in persistent acute respiratory distress syndrome: a randomized, controlled trial. *Crit Care Med* **34**(5): 1311-8, 2006. PMID 16557151

7. ARDS Network. Ventilation with lower tidal volumes as compared with traditional tidal volumes for acute lung injury and the acute respiratory distress syndrome. The Acute Respiratory Distress Syndrome Network. *N Engl J Med* **342**(18): 1301-8, 2000. PMID 10793162

CQ06. Study flow diagram

*This CQ was partly evaluated by Blackwood using Cochrane database (to Feb 2014)[1]. We also searched literature from 2014 to May 2015.

Blackwood B, Burns KE, Cardwell CR, et al. Protocolized versus non-protocolized weaning for reducing the duration of mechanical ventilation in critically ill adult patients. *Cochrane Database Syst Rev* **11**: CD006904, 2014. PMID 25375085

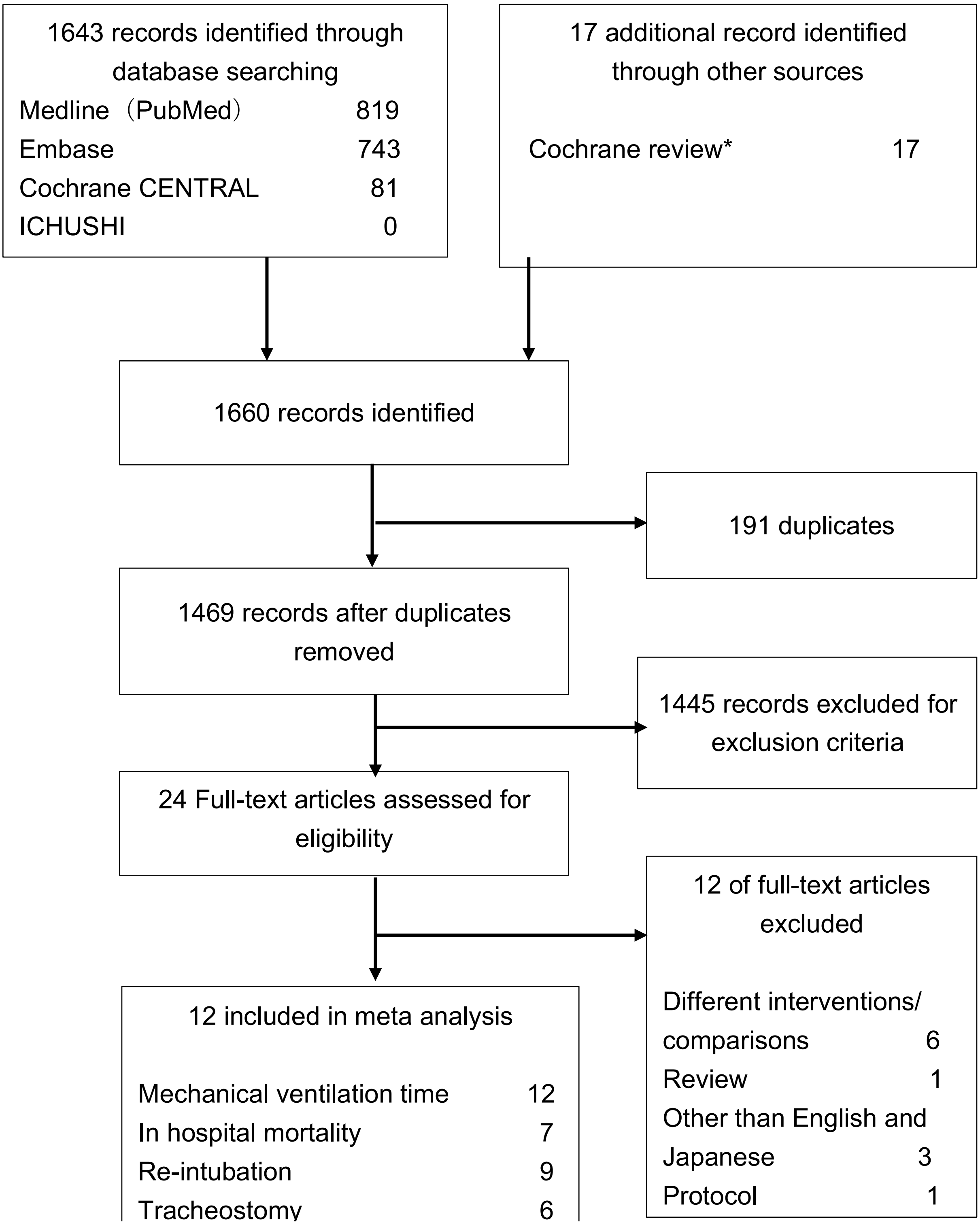

CQ06

Risk of bias table, Total duration of MV

Outcome		Total duration of MV		risk of bias		not serious (0)			
番号	著者名 発表年（Forest plot表示）	risk of bias評価							
		ランダム割付順番の生成 random sequence generation	割り付けの隠蔽化 allocation concealment	ブラインド blinding 研究参加者と治療提供者 participants and personnel	ブラインド blinding アウトカム評価者 outcome assessors	不完全なアウトカムデータ incomplete outcome data	選択されたアウトカムの報告 selective outcome reporting	その他のバイアス Other sources of bias	研究内でのバイアスのリスク Risk of bias within a study
1	Chaiwat 2010	Low risk	Unclear risk	High risk	Low risk	Low risk	Unclear risk	High risk	Unclear risk
2	Ely 1996	Low risk	Low risk	High risk	Low risk	Low risk	Low risk	Low risk	Low risk
3	Kollef 1997	Low risk	Unclear risk	High risk	Low risk	Low risk	Low risk	Low risk	Low risk
4	Krishnan 2004	High risk	High risk	High risk	Low risk	Low risk	Low risk	Low risk	Unclear risk
5	Marelich 2000	Low risk	Low risk	High risk	Low risk	Low risk	Low risk	Low risk	Low risk
6	Namen 2001	Unclear risk	Unclear risk	High risk	Low risk	Low risk	Low risk	Unclear risk	Low risk
7	Navalesi 2008	Unclear risk	Unclear risk	High risk	Low risk	Low risk	Low risk	Low risk	Low risk
8	Roh 2012	Low risk	Low risk	High risk	Low risk	Unclear risk	Unclear risk	Low risk	Low risk
9	Rose 2008	Low risk	Unclear risk	High risk	Low risk	Low risk	Low risk	Low risk	Low risk
10	Simeone 2002	Unclear risk	Unclear risk	High risk	Low risk	Unclear risk	Unclear risk	Unclear risk	Unclear risk
11	Stahl 2009	Low risk	Unclear risk	High risk	Low risk	Low risk	Low risk	Unclear risk	Low risk

番号	著者名 発表年（Forest plot表示）	risk of biasコメント							
1	Chaiwat 2010	ブロック無作為	Opaque envelopeだがブロック無作為のため	盲験化は不可能	盲験化しなくても評価に影響なし	データの欠損なし	研究プロトコールが利用できないため、情報が不十分（死亡率もない）	有意な結果で早期中止	low3項目、high2項目
2	Ely 1996	コンピュータを使用	opaque envelope	盲験化は不可能	盲験化しなくても評価に影響なし	データ欠損なし	期待アウトカム含む　プロトコル記載あり	特になし	low6項目、high1項目
3	Kollef 1997	ブロック無作為のため	ブロックのため	盲験化は不可能	盲験化しなくても評価に影響なし	データ欠損なし	期待アウトカム含む、プロトコル記載あり	特に無し	low5項目、high1項目
4	Krishnan 2004	病院での番号が偶数か奇数かで割り付け	偶数か奇数かなので予想可能	盲験化は不可能	盲験化しなくても評価に影響なし	データ欠損なし	研究プロトコル入手可能、期待アウトカム含む	特に無し	low4項目、high3項目
5	Marelich 2000	見えない状態でシャッフル	opaque envelopeを使用	盲験化は不可能	盲験化しなくても評価に影響なし	データ欠損なし	詳細プロトコール入手可、期待アウトカムあり	特に無し	low6項目、high1項目
6	Namen 2001	ランダムの記載のみで詳述なし	詳述なし	盲験化は不可能	盲験化しなくても評価に影響なし	データの欠損なし	研究プロトコールが利用でき、予め決められたアウトカムの報告あり	差が出ず早期に終了	low3項目、high1項目
7	Navalesi 2008	詳細な記載なし	詳細な記載なし	盲験化は不可能	盲験化しなくても評価に影響なし	データの欠損なし	研究プロトコールが利用でき、予め決められたアウトカムの報告あり	他のbiasなし	low4項目、high1項目
8	Roh 2012	computorによるランダム化	割り付けの隠蔽化の手順に問題なし	盲験化は不可能	盲験化しなくても評価に影響なし	データの欠損あり(122人中93人の人工呼吸期間を調査、欠損に群間差はない）	研究プロトコールが入手不可のため判断困難（再挿管率がない）	他のbiasなし	low4項目、high1項目
9	Rose 2008	computorによるランダム化	ブロック無作為化のため割り付けを予測できる可能性がある	盲験化は不可能	盲験化しなくても評価に影響なし	データの欠損なし	研究プロトコールが利用でき、アウトカムの報告あり	他のbiasなし	low5項目、high1項目
10	Simeone 2002	詳細な記載なし	詳細な記載なし	盲験化は不可能	盲験化しなくても評価に影響なし	判断するには情報が不十分	研究プロトコールが利用できず、判断するには情報が不十分	評価するための十分な情報がない	low1項目、high1項目
11	Stahl 2009	ブロックランダム割付	ブロック無作為化のため割り付けを予想できる可能性がある	盲験化は不可能	盲験化しなくても評価に影響なし	データの欠損なし	予め決められたアウトカムは報告されている	有効性認めず早期終了	low4項目、high1項目

CQ06

Risk of bias table Hospital mortality

Outcome	Hospital mortality	risk of bias	not serious (0)

番号	著者名 発表年（Forest plot表示）	risk of bias評価							
		ランダム割付順番の生成 random sequence generation	割り付けの隠蔽化 allocation concealment	ブラインド blinding		不完全なアウトカムデータ incomplete outcome data	選択されたアウトカムの報告 selective outcome reporting	その他のバイアス Other sources of bias	研究内でのバイアスのリスク Risk of bias within a study
				研究参加者と治療提供者 participants and personnel	アウトカム評価者 outcome assessors				
1	Ely 1996	Low risk	Low risk	High risk	Low risk	Low risk	Low risk	Low risk	Low risk
2	Kollef 1997	Low risk	Unclear risk	High risk	Low risk	Low risk	Low risk	Low risk	Low risk
3	Krishnan 2004	High risk	High risk	High risk	Low risk	Low risk	Low risk	Low risk	Unclear risk
4	Marelich 2000	Low risk	Low risk	High risk	Low risk	Low risk	Low risk	Low risk	Low risk
5	Namen 2001	Unclear risk	Unclear risk	High risk	Low risk	Low risk	Low risk	Unclear risk	Low risk
6	Roh 2012	Low risk	Low risk	High risk	Low risk	Unclear risk	Unclear risk	Low risk	Low risk
7	Stahl 2009	Low risk	Unclear risk	High risk	Low risk	Low risk	Low risk	Unclear risk	Low risk

番号	著者名 発表年（Forest plot表示）	risk of biasコメント							
1	Ely 1996	コンピュータを使用	opaque envelope	盲験化は不可能	盲験化しなくても評価に影響なし	データ欠損なし	期待アウトカム含む プロトコル記載あり	特になし	low6項目、high1項目
2	Kollef 1997	ブロック無作為のため	ブロックのため	盲験化は不可能	盲験化しなくても評価に影響なし	データ欠損なし	期待アウトカム含む、プロトコル記載あり	特に無し	low5項目、high1項目
3	Krishnan 2004	病院での番号が偶数か奇数かで割り付け	偶数か奇数かなので予想可能	盲験化は不可能	盲験化しなくても評価に影響なし	データ欠損なし	研究プロトコル入手可能、期待アウトカム含む	特に無し	low4項目、high3項目
4	Marelich 2000	見えない状態でシャッフル	opaque envelopeを使用	盲験化は不可能	盲験化しなくても評価に影響なし	データ欠損なし	詳細プロトコール入手可、期待アウトカムあり	特に無し	low6項目、high1項目
5	Namen 2001	ランダムの記載のみで詳述なし	詳述なし	盲験化は不可能	盲験化しなくても評価に影響なし	データの欠損なし	研究プロトコールが利用でき、予め決められたアウトカムの報告あり	差が出ず早期に終了	low3項目、high1項目
6	Roh 2012	computorによるランダム化	割り付けの隠蔽化の手順に問題なし	盲験化は不可能	盲験化しなくても評価に影響なし	データの欠損あり(122人中93人の人工呼吸期間を調査、欠損に群間差はない)	研究プロトコールが入手不可のため判断困難(再挿管率がない)	他のbiasなし	low4項目、high1項目
7	Stahl 2009	ブロックランダム割付	ブロック無作為化のため割り付けを予想できる可能性がある	盲験化は不可能	盲験化しなくても評価に影響なし	データの欠損なし	予め決められたアウトカムは報告されている	有効性認めず早期終了	low4項目、high1項目

CQ06
Risk of bias table, Reintubation

Outcome	Reintubation	risk of bias	not serious (0)

番号	著者名 発表年（Forest plot表示）	risk of bias評価							
		ランダム割付順番の生成 random sequence generation	割り付けの隠蔽化 allocation concealment	ブラインド blinding		不完全なアウトカムデータ incomplete outcome data	選択されたアウトカムの報告 selective outcome reporting	その他のバイアス Other sources of bias	研究内でのバイアスのリスク Risk of bias within a study
				研究参加者と治療提供者 participants and personnel	アウトカム評価者 outcome assessors				
1	Chaiwat 2010	Low risk	Unclear risk	High risk	Low risk	Low risk	Unclear risk	High risk	Unclear risk
2	Ely 1996	Low risk	Low risk	High risk	Low risk	Low risk	Low risk	Low risk	Low risk
3	Kollef 1997	Low risk	Unclear risk	High risk	Low risk	Low risk	Low risk	Low risk	Low risk
4	Namen 2001	Unclear risk	Unclear risk	High risk	Low risk	Low risk	Low risk	Unclear risk	Low risk
5	Navalesi 2008	Unclear risk	Unclear risk	High risk	Low risk	Low risk	Low risk	Low risk	Low risk
6	Piotto 2011	High risk	High risk	High risk	Low risk	Unclear risk	Low risk	Low risk	Unclear risk
7	Rose 2008	Low risk	Unclear risk	High risk	Low risk	Low risk	Low risk	Low risk	Low risk
8	Simeone 2002	Unclear risk	Unclear risk	High risk	Low risk	Unclear risk	Unclear risk	Unclear risk	Unclear risk
9	Stahl 2009	Low risk	Unclear risk	High risk	Low risk	Low risk	Low risk	Unclear risk	Low risk

番号	著者名 発表年（Forest plot表示）	risk of biasコメント							
1	Chaiwat 2010	ブロック無作為	Opaque envelopeだがブロック無作為のため	盲験化は不可能	盲験化しなくても評価に影響なし	データの欠損なし	研究プロトコールが利用できないため、情報が不十分（死亡率もない）	有意な結果で早期中止	low3項目、high2項目
2	Ely 1996	コンピュータを使用	opaque envelope	盲験化は不可能	盲験化しなくても評価に影響なし	データ欠損なし	期待アウトカム含む プロトコル記載あり	特になし	low6項目、high1項目
3	Kollef 1997	ブロック無作為のため	ブロックのため	盲験化は不可能	盲験化しなくても評価に影響なし	データ欠損なし	期待アウトカム含む、プロトコル記載あり	特に無し	low5項目、high1項目
4	Namen 2001	ランダムの記載のみで詳述なし	詳述なし	盲験化は不可能	盲験化しなくても評価に影響なし	データの欠損なし	研究プロトコールが利用でき、予め決められたアウトカムの報告あり	差が出ず早期に終了	low3項目、high1項目
5	Navalesi 2008	詳細な記載なし	詳細な記載なし	盲験化は不可能	盲験化しなくても評価に影響なし	データの欠損なし	研究プロトコールが利用でき、予め決められたアウトカムの報告あり	他のbiasなし	low4項目、high1項目
6	Piotto 2011	交互に割付された	交互に割付された	盲験化は不可能	盲験化しなくても評価に影響なし	判断するための十分な情報がない	研究プロトコールが利用でき、予め決められたアウトカムの報告あり	他のbiasなし	low3項目、high3項目
7	Rose 2008	computorによるランダム化	ブロック無作為化のため割り付けを予測できる可能性がある	盲験化は不可能	盲験化しなくても評価に影響なし	データの欠損なし	研究プロトコールが利用でき、アウトカムの報告あり	他のbiasなし	low5項目、high1項目
8	Simeone 2002	詳細な記載なし	詳細な記載なし	盲験化は不可能	盲験化しなくても評価に影響なし	判断するには情報が不十分	研究プロトコールが利用できず、判断するには情報が不十分	評価するための十分な情報がない	low1項目、high1項目
8	Stahl 2009	ブロックランダム割付	ブロック無作為化のため割り付けを予想できる可能性がある	盲験化は不可能	盲験化しなくても評価に影響なし	データの欠損なし	予め決められたアウトカムは報告されている	有効性認めず早期終了	low4項目、high1項目

CQ06

Risk of bias table, Tracheostomy

Tracheostomy	Tracheostomy	risk of bias	not serious (0)

番号	著者名 発表年（Forest plot表示）	risk of bias評価							
		ランダム割付順番の生成 random sequence generation	割り付けの隠蔽化 allocation concealment	ブラインド 研究参加者と治療提供者 participants and personnel	ブラインド アウトカム評価者 outcome assessors	不完全なアウトカムデータ incomplete outcome data	選択されたアウトカムの報告 selective outcome	その他のバイアス Other sources of bias	研究内でのバイアスのリスク Risk of bias within a study
1	Ely 1996	Low risk	Low risk	High risk	Low risk	Low risk	Low risk	Low risk	Low risk
2	Marelich 2000	Low risk	Low risk	High risk	Low risk	Low risk	Low risk	Low risk	Low risk
3	Namen 2001	Unclear risk	Unclear risk	High risk	Low risk	Low risk	Low risk	Unclear risk	Low risk
4	Navalesi 2008	Unclear risk	Unclear risk	High risk	Low risk	Low risk	Low risk	Low risk	Low risk
5	Roh 2012	Low risk	Low risk	High risk	Low risk	Unclear risk	Unclear risk	Low risk	Low risk
6	Rose 2008	Low risk	Unclear risk	High risk	Low risk	Low risk	Low risk	Low risk	Low risk

番号	著者名 発表年（Forest plot表示）	risk of biasコメント							
1	Ely 1996	コンピュータを使用	opaque envelope	盲験化は不可能	盲験化しなくても評価に影響なし	データ欠損なし	期待アウトカム含む プロトコル記載あり	特になし	low6項目、high1項目
2	Marelich 2000	見えない状態でシャッフル	opaque envelopeを使用	盲験化は不可能	盲験化しなくても評価に影響なし	データ欠損なし	詳細プロトコール入手可、期待アウトカムあり	特に無し	low6項目、high1項目
3	Namen 2001	ランダムの記載のみで詳述なし	詳述なし	盲験化は不可能	盲験化しなくても評価に影響なし	データの欠損なし	研究プロトコールが利用でき、予め決められたアウトカムの報告あり	差が出ず早期に終了	low3項目、high1項目
4	Navalesi 2008	詳細な記載なし	詳細な記載なし	盲験化は不可能	盲験化しなくても評価に影響なし	データの欠損なし	研究プロトコールが利用でき、予め決められたアウトカムの報告あり	他のbiasなし	low4項目、high1項目
5	Roh 2012	computorによるランダム化	割り付けの隠蔽化の手順に問題なし	盲験化は不可能	盲験化しなくても評価に影響なし	データの欠損あり（122人中93人の人工呼吸期間を調査、欠損に群間差はない）	研究プロトコールが入手不可のため判断困難（再挿管率がない）	他のbiasなし	low4項目、high1項目
6	Rose 2008	computorによるランダム化	ブロック無作為化のため割り付けを予測できる可能性がある	盲験化は不可能	盲験化しなくても評価に影響なし	データの欠損なし	研究プロトコールが利用でき、アウトカムの報告あり	他のbiasなし	low5項目、high1項目

CQ06-04. Risk of bias summary, CQ06-05. Risk of bias graph

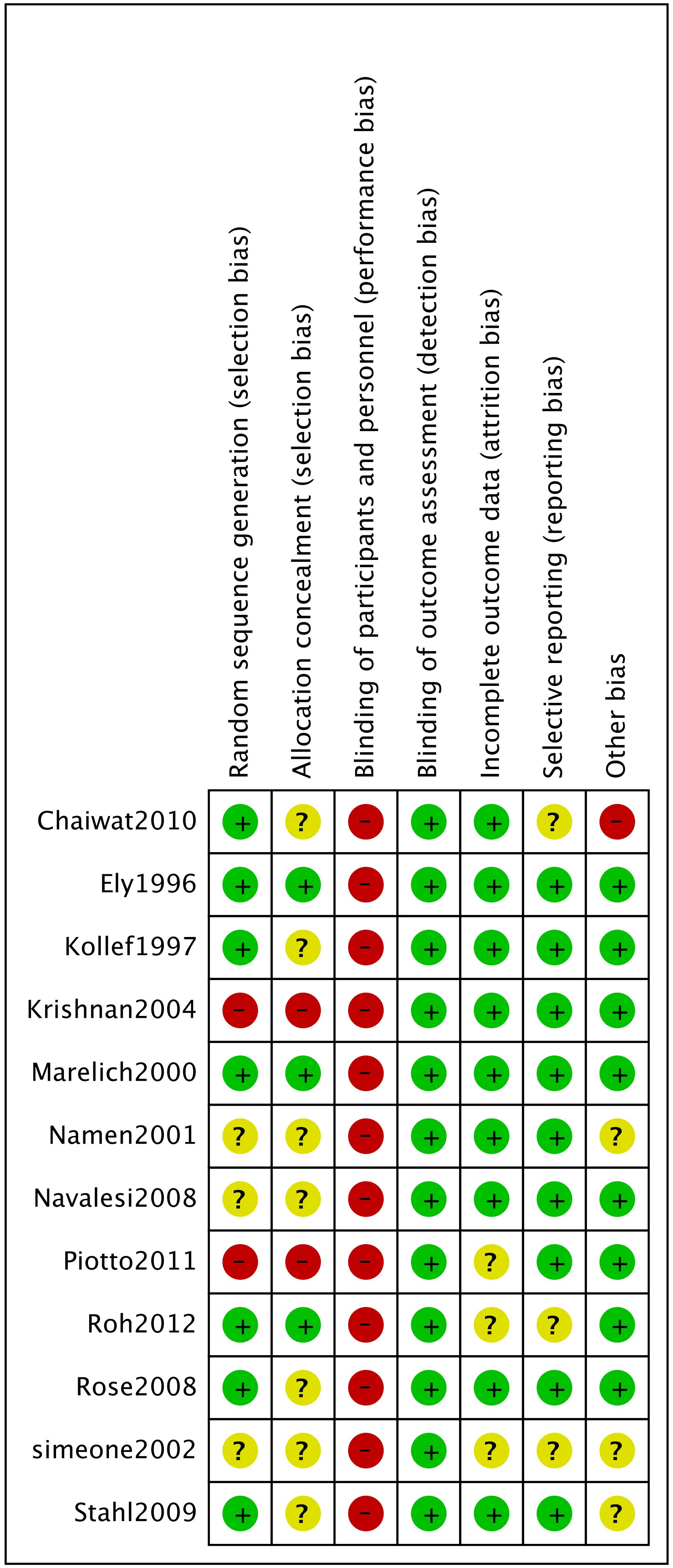

Mechanical Ventilation Time

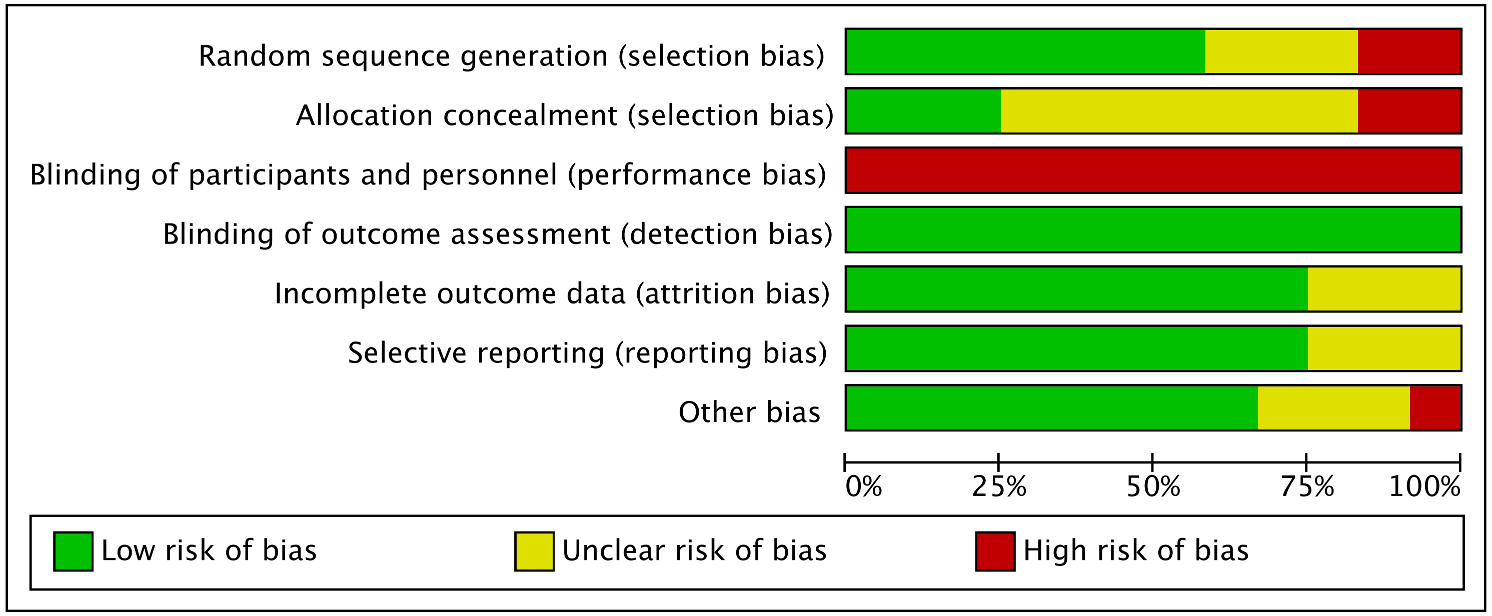

CQ06-04. Risk of bias summary, CQ06-05. Risk of bias graph

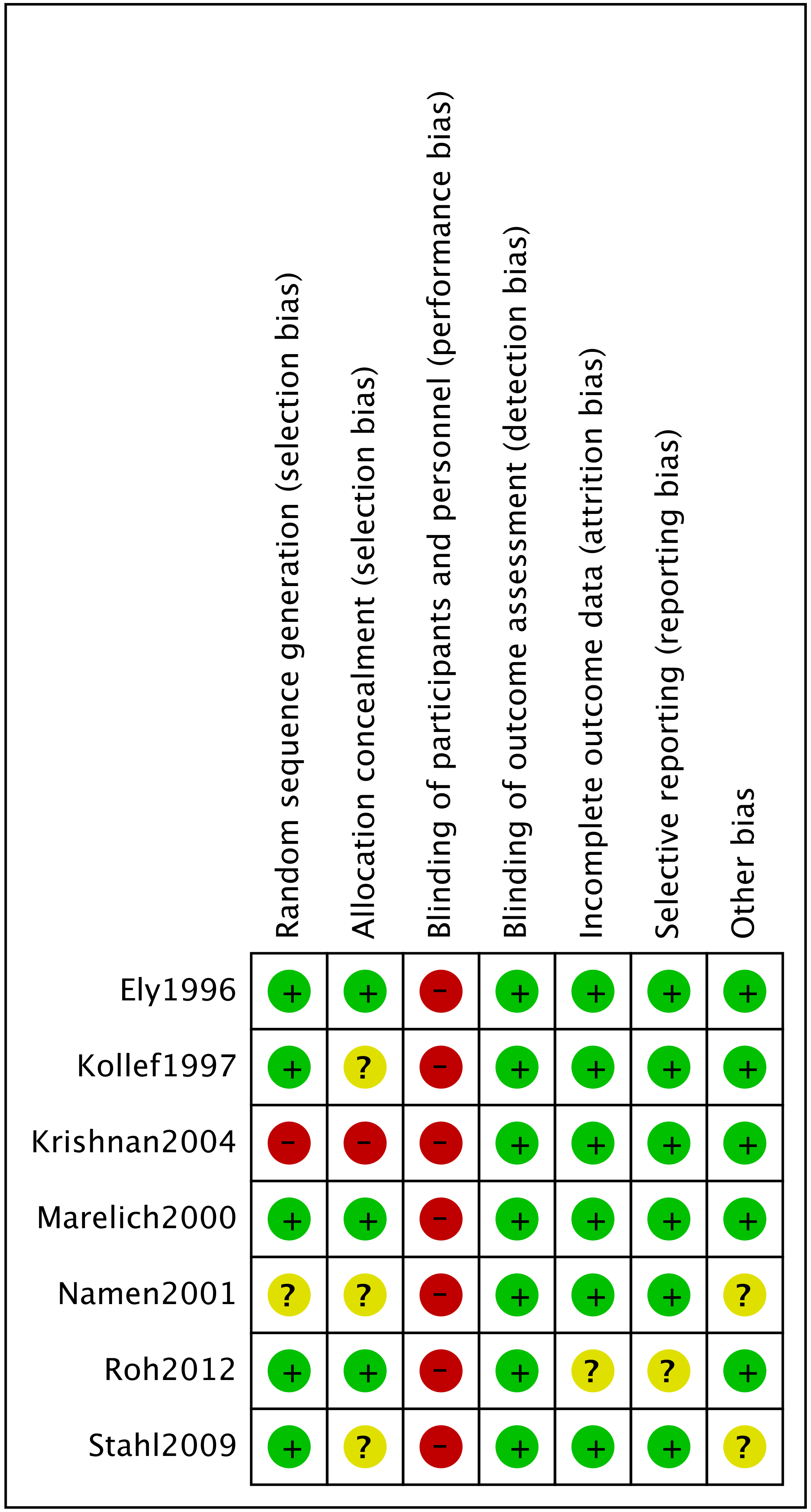

In hospital mortality

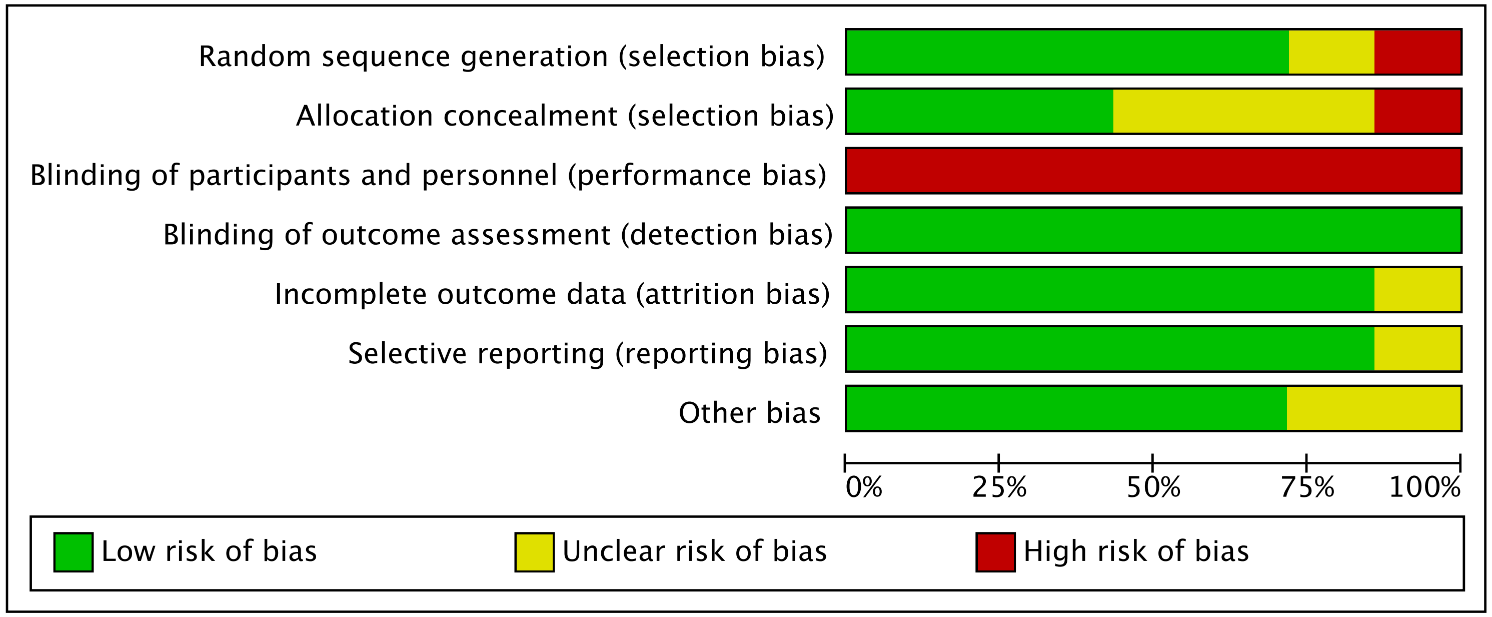

CQ06-04. Risk of bias summary, CQ06-05. Risk of bias graph

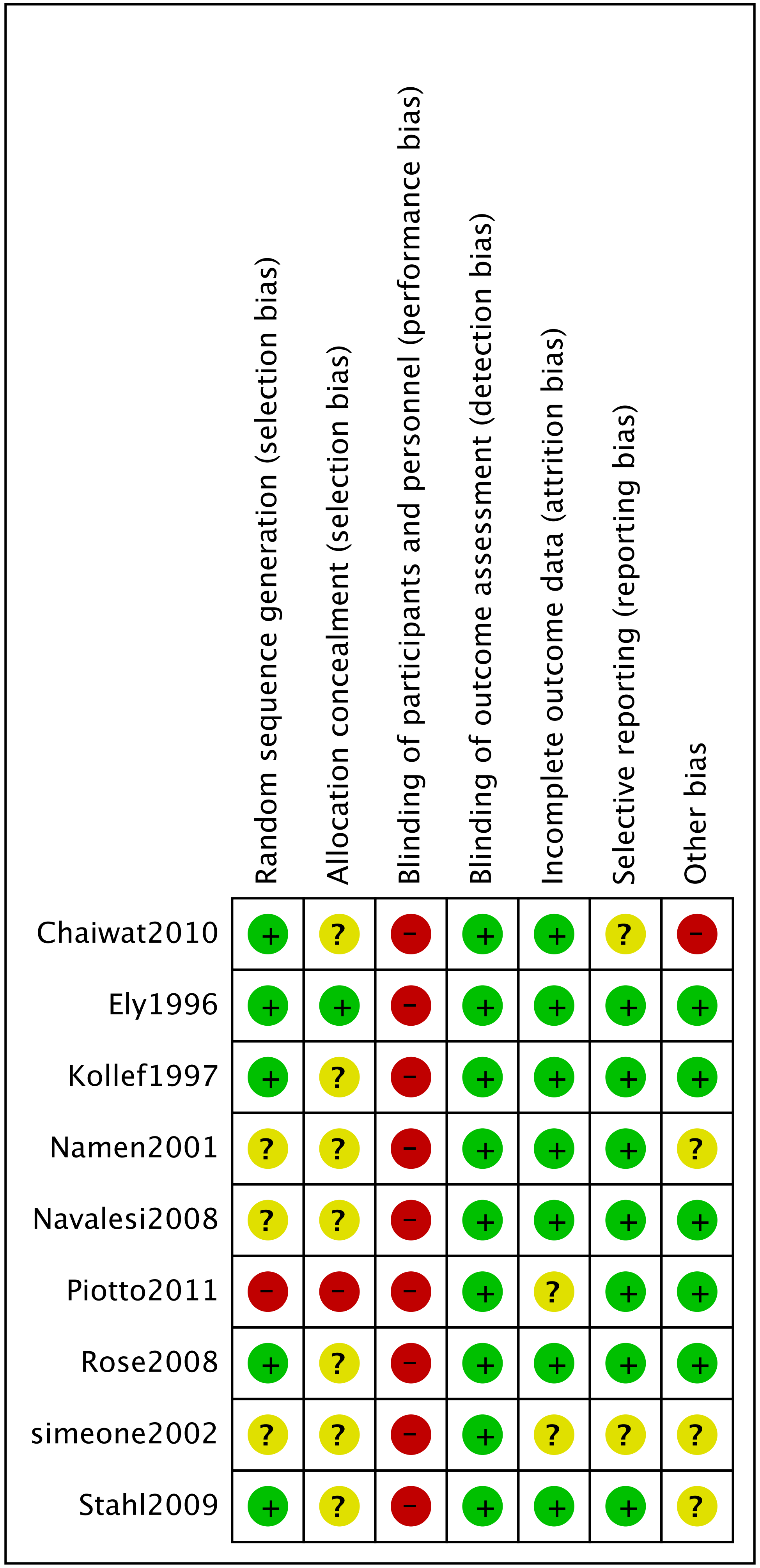

Re-intubation

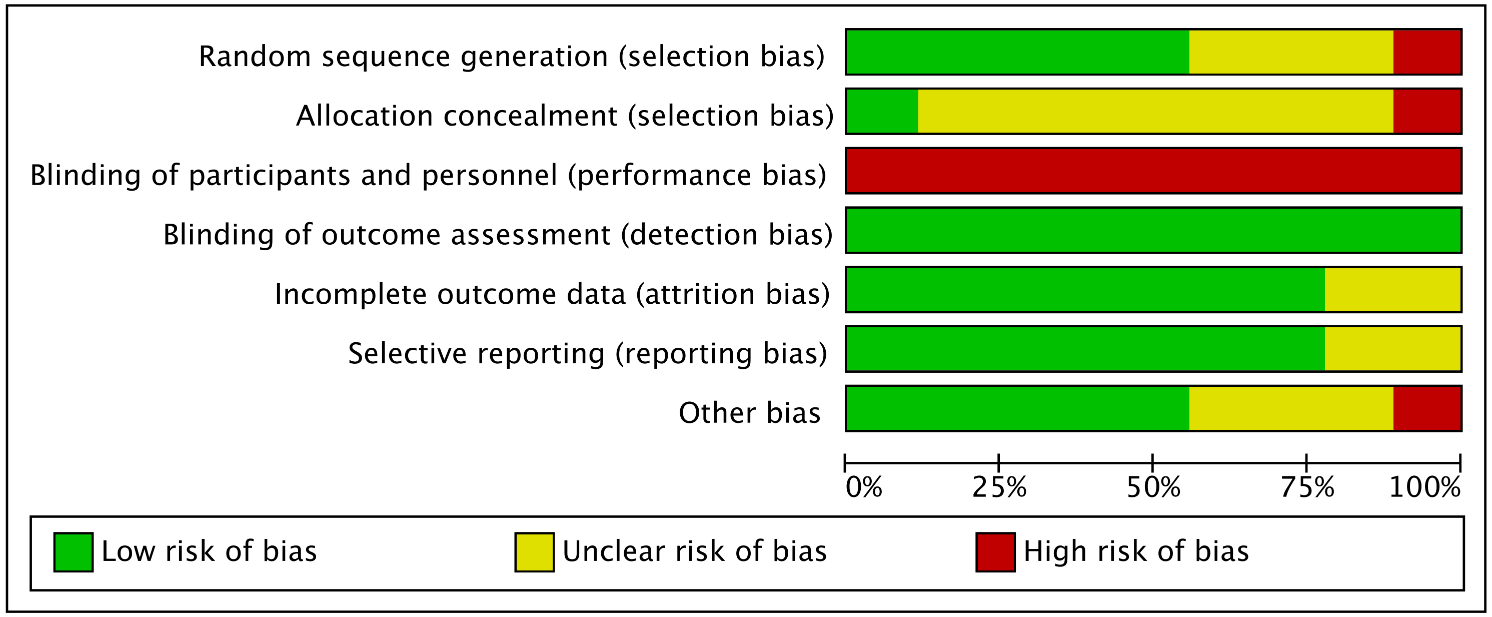

CQ06-04. Risk of bias summary, CQ06-05. Risk of bias graph

tracheostomy

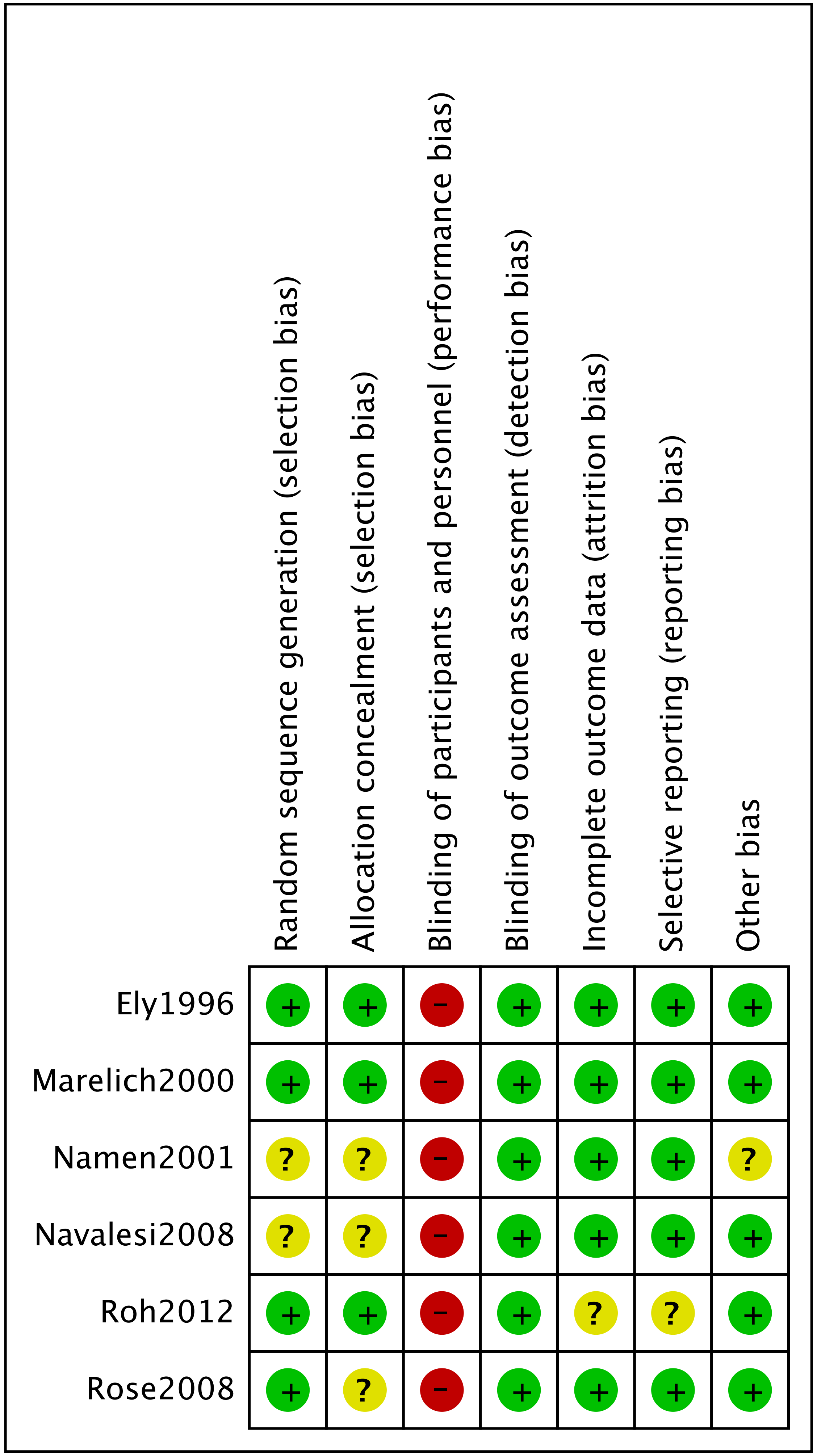

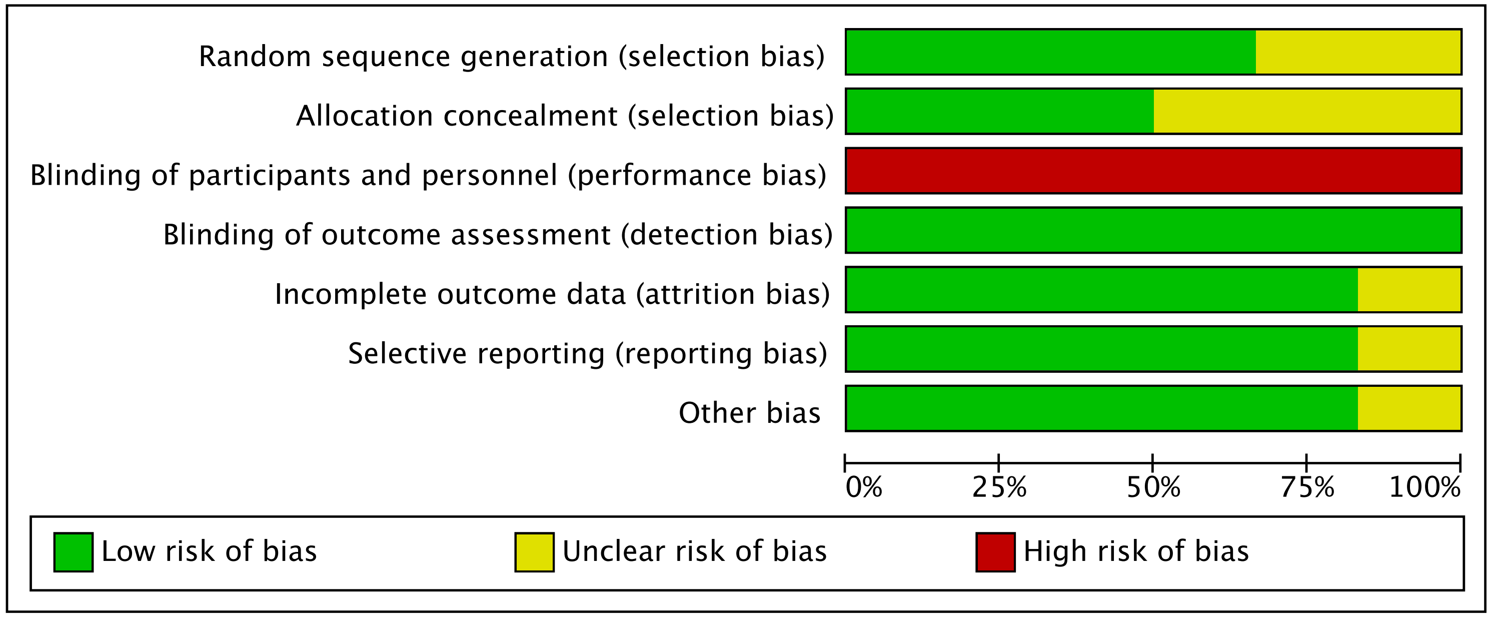

Mechanical Ventilation Time

Study or Subgroup	Protocolized Mean	Protocolized SD	Protocolized Total	non-protocolized Mean	non-protocolized SD	non-protocolized Total	Weight	Mean Difference IV, Random, 95% CI
Chaiwat2010	98.5	176.76	51	255.71	868.28	49	0.5%	−157.21 [−405.12, 90.70]
Ely1996	151.2	175.2	149	211.2	261.6	151	7.6%	−60.00 [−110.32, −9.68]
Kollef1997	69.4	123.7	179	102	169.1	178	13.2%	−32.60 [−63.35, −1.85]
Krishnan2004	60.4	103	115	68	105.3	109	14.6%	−7.60 [−34.90, 19.70]
Marelich2000	68	97	166	124	207	169	11.9%	−56.00 [−90.52, −21.48]
Namen2001	144	124.45	49	144	195.56	51	5.4%	0.00 [−63.99, 63.99]
Navalesi2008	120	134.4	165	120	120	153	14.3%	0.00 [−27.97, 27.97]
Piotto2011	189.25	463.55	18	127.48	337.37	18	0.4%	61.77 [−203.09, 326.63]
Roh2012	277.23	320.66	61	424.3	686.42	61	0.8%	−147.07 [−337.19, 43.05]
Rose2008	119	174.89	51	129	197.07	51	4.4%	−10.00 [−82.31, 62.31]
Simeone2002	6.54	3.78	24	8.58	3.45	25	23.3%	−2.04 [−4.07, −0.01]
Stahl2009	135.6	122.1	26	199.44	172.3	26	3.7%	−63.84 [−145.01, 17.33]
**Total (95% CI)**			**1054**			**1041**	**100.0%**	**−21.51 [−38.45, −4.56]**

Heterogeneity: $Tau^2 = 319.64$; $Chi^2 = 24.47$, df = 11 (P = 0.01); $I^2 = 55\%$
Test for overall effect: Z = 2.49 (P = 0.01)

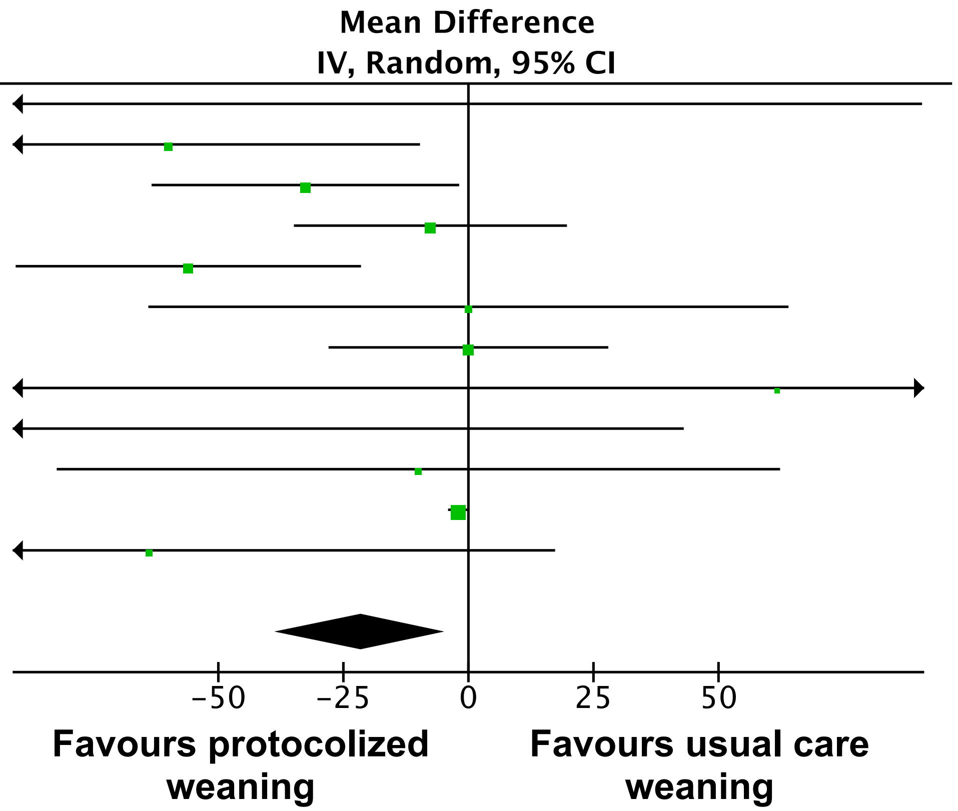

In hospital mortality

Study or Subgroup	Protocolized Events	Protocolized Total	non-protocolized Events	non-protocolized Total	Weight	Risk Ratio M−H, Random, 95% CI
Ely1996	56	149	60	151	33.0%	0.95 [0.71, 1.26]
Kollef1997	40	179	42	178	18.6%	0.95 [0.65, 1.38]
Krishnan2004	56	154	48	145	27.7%	1.10 [0.80, 1.50]
Marelich2000	17	166	10	169	4.8%	1.73 [0.82, 3.67]
Namen2001	20	49	16	51	9.7%	1.30 [0.77, 2.21]
Roh2012	9	61	11	61	4.1%	0.82 [0.37, 1.83]
Stahl2009	5	30	5	30	2.1%	1.00 [0.32, 3.10]
**Total (95% CI)**		**788**		**785**	**100.0%**	**1.04 [0.88, 1.23]**
Total events	203		192			

Heterogeneity: $Tau^2 = 0.00$; $Chi^2 = 3.58$, df = 6 (P = 0.73); $I^2 = 0\%$
Test for overall effect: Z = 0.49 (P = 0.63)

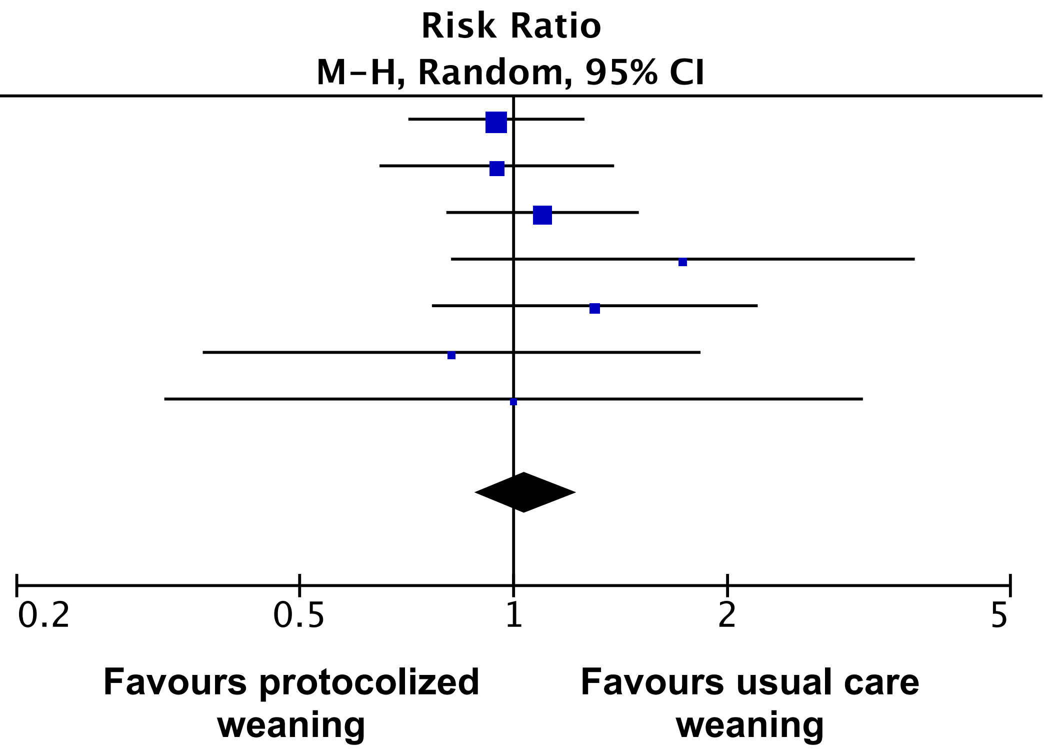

Re-intubation

Study or Subgroup	Protocolized Events	Protocolized Total	non-protocolized Events	non-protocolized Total	Weight	Risk Ratio M-H, Random, 95% CI
Chaiwat2010	2	51	3	49	5.6%	0.64 [0.11, 3.67]
Ely1996	5	149	12	151	11.6%	0.42 [0.15, 1.17]
Kollef1997	23	179	18	178	18.6%	1.27 [0.71, 2.27]
Namen2001	10	49	6	51	12.7%	1.73 [0.68, 4.41]
Navalesi2008	9	165	18	153	15.2%	0.46 [0.21, 1.00]
Piotto2011	3	18	12	18	10.8%	0.25 [0.08, 0.74]
Rose2008	5	51	6	51	10.4%	0.83 [0.27, 2.56]
Simeone2002	1	24	0	25	2.0%	3.12 [0.13, 73.04]
Stahl2009	8	26	6	26	13.1%	1.33 [0.54, 3.31]
**Total (95% CI)**		**712**		**702**	**100.0%**	**0.79 [0.50, 1.26]**
Total events	66		81			

Heterogeneity: $Tau^2 = 0.21$; $Chi^2 = 14.92$, df = 8 (P = 0.06); $I^2 = 46\%$
Test for overall effect: Z = 0.99 (P = 0.32)

Risk Ratio
M-H, Random, 95% CI

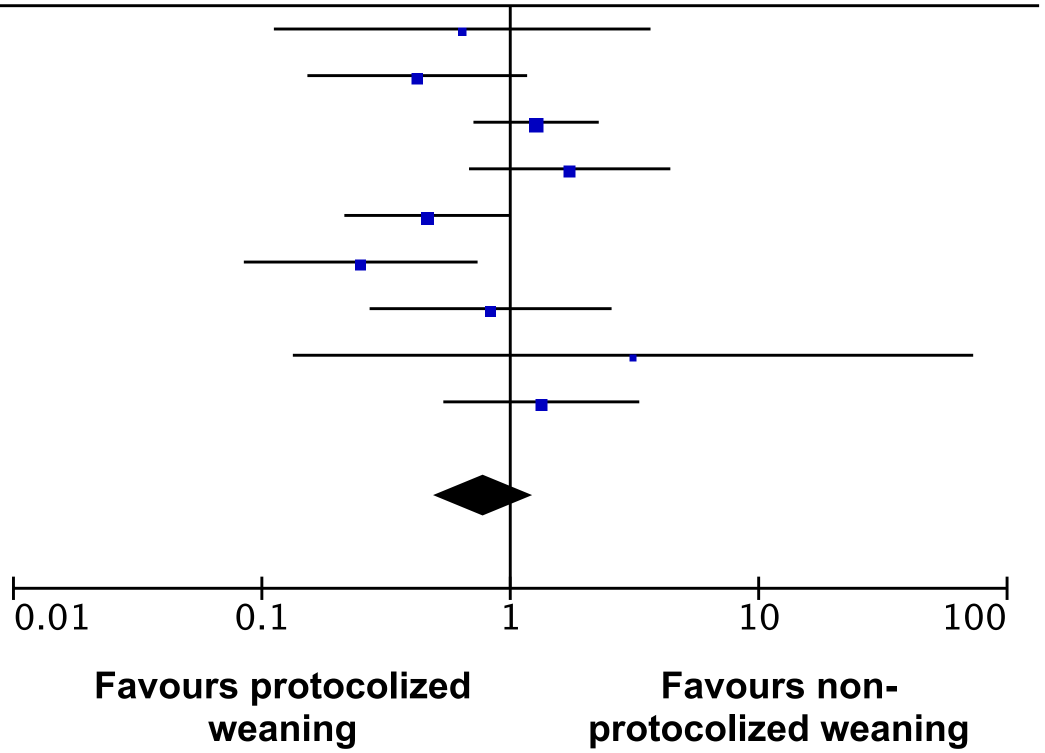

Tracheostomy

Study or Subgroup	Protocolized Events	Protocolized Total	non-protocolized Events	non-protocolized Total	Weight	Risk Ratio M-H, Random, 95% CI
Ely1996	13	149	22	151	24.3%	0.60 [0.31, 1.14]
Marelich2000	13	166	21	169	23.5%	0.63 [0.33, 1.22]
Namen2001	14	49	15	51	27.0%	0.97 [0.53, 1.79]
Navalesi2008	5	165	11	153	9.5%	0.42 [0.15, 1.19]
Roh2012	5	61	3	61	5.3%	1.67 [0.42, 6.67]
Rose2008	6	51	8	51	10.5%	0.75 [0.28, 2.01]
**Total (95% CI)**		**641**		**636**	**100.0%**	**0.72 [0.52, 0.99]**
Total events	56		80			

Heterogeneity: $Tau^2 = 0.00$; $Chi^2 = 3.85$, df = 5 (P = 0.57); $I^2 = 0\%$
Test for overall effect: Z = 2.00 (P = 0.05)

Risk Ratio
M-H, Random, 95% CI

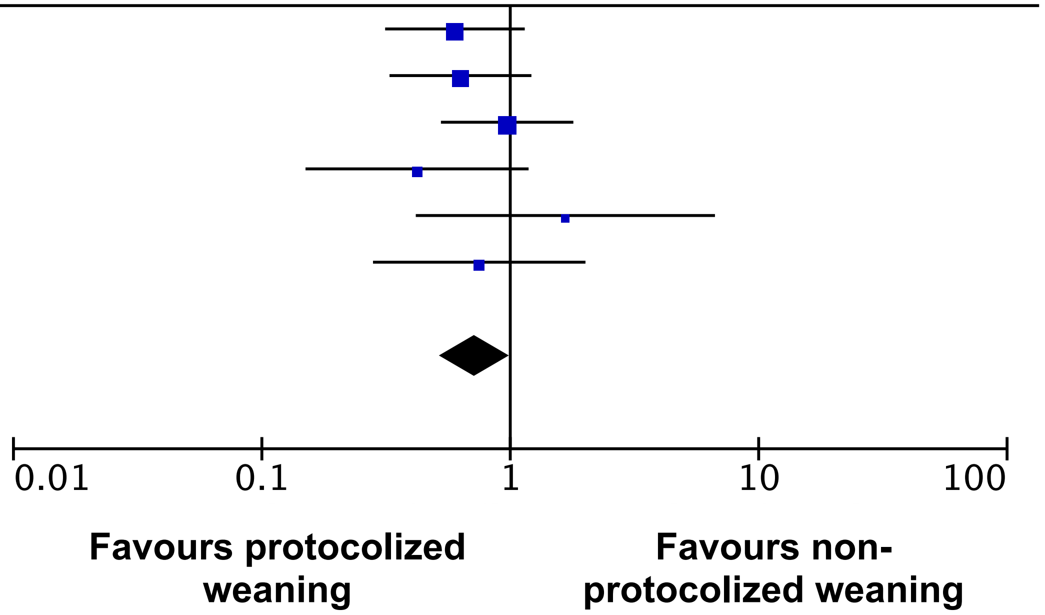

subgroup analysis
Mechanical ventilation time
(RCTs with SBT protocol)

Study or Subgroup	protocolized Mean	protocolized SD	protocolized Total	non-protocolized Mean	non-protocolized SD	non-protocolized Total	Weight	Mean Difference IV, Random, 95% CI
Chaiwat2010	98.5	176.76	51	255.71	868.28	49	0.7%	−157.21 [−405.12, 90.70]
Ely1996	151.2	175.2	149	211.2	261.6	151	15.1%	−60.00 [−110.32, −9.68]
Kollef1997	69.4	123.7	179	102	169.1	178	0.0%	−32.60 [−63.35, −1.85]
Krishnan2004	60.4	103	115	68	105.3	109	37.3%	−7.60 [−34.90, 19.70]
Marelich2000	68	97	166	124	207	169	0.0%	−56.00 [−90.52, −21.48]
Namen2001	144	124.45	49	144	195.56	51	10.0%	0.00 [−63.99, 63.99]
Navalesi2008	120	134.4	165	120	120	153	36.2%	0.00 [−27.97, 27.97]
Piotto2011	189	463.5	18	127.5	337.4	18	0.6%	61.50 [−203.35, 326.35]
Roh2012	277.23	320.66	61	424.3	686.42	61	0.0%	−147.07 [−337.19, 43.05]
Rose2008	119	174.89	51	129	197.07	51	0.0%	−10.00 [−82.31, 62.31]
Simeone2002	6.54	3.78	24	8.58	3.45	25	0.0%	−2.04 [−4.07, −0.01]
Stahl2009	135.6	122.1	26	199.44	172.3	26	0.0%	−63.84 [−145.01, 17.33]
**Total (95% CI)**			**547**			**531**	**100.0%**	**−12.67 [−34.00, 8.65]**

Heterogeneity: $Tau^2 = 123.36$; $Chi^2 = 6.03$, df = 5 (P = 0.30); $I^2 = 17\%$
Test for overall effect: Z = 1.16 (P = 0.24)

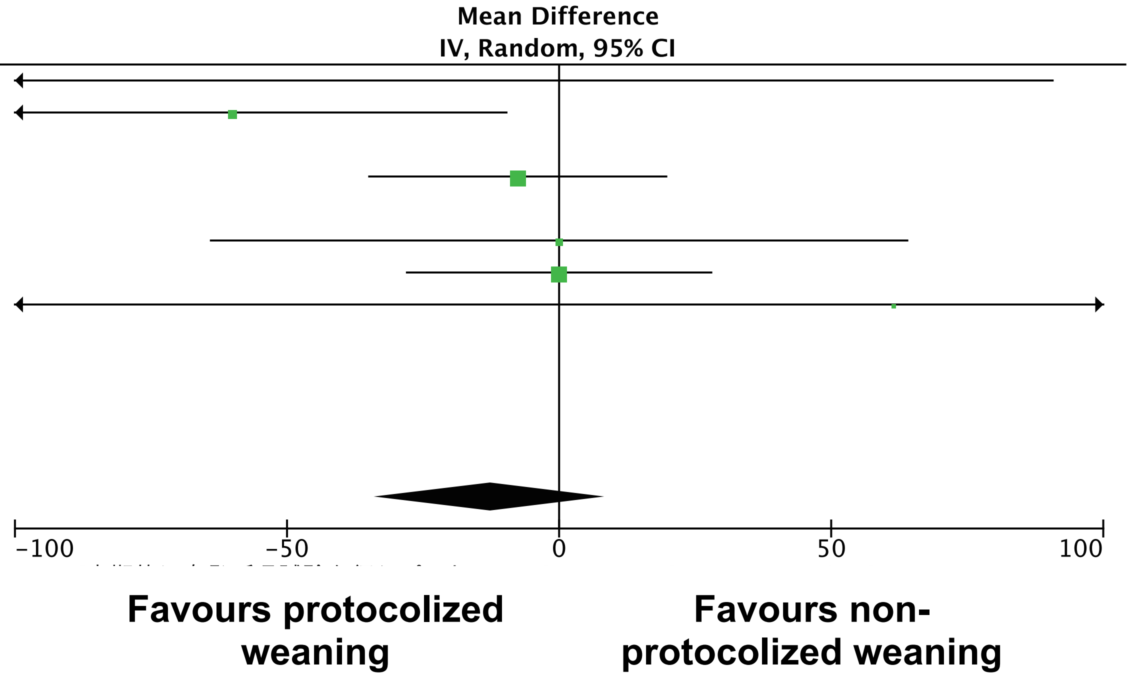

subgroup analysis
Mechanical ventilation time
(RCTs with stepwise reduction protocol)

Study or Subgroup	protocolized Mean	protocolized SD	protocolized Total	non-protocolized Mean	non-protocolized SD	non-protocolized Total	Weight	Mean Difference IV, Random, 95% CI
Chaiwat2010	98.5	176.76	51	255.71	868.28	49		Not estimable
Ely1996	151.2	175.2	149	211.2	261.6	151		Not estimable
Kollef1997	69.4	123.7	179	102	169.1	178	23.6%	−32.60 [−63.35, −1.85]
Krishnan2004	60.4	103	115	68	105.3	109		Not estimable
Marelich2000	68	97	166	124	207	169	22.2%	−56.00 [−90.52, −21.48]
Namen2001	144	124.45	49	144	195.56	51		Not estimable
Navalesi2008	120	134.4	165	120	120	153		Not estimable
Piotto2011	189.25	463.55	18	127.48	337.37	18		Not estimable
Roh2012	277.23	320.66	61	424.3	686.42	61	2.3%	−147.07 [−337.19, 43.05]
Rose2008	119	174.89	51	129	197.07	51	11.0%	−10.00 [−82.31, 62.31]
Simeone2002	6.54	3.78	24	8.58	3.45	25	31.6%	−2.04 [−4.07, −0.01]
Stahl2009	135.6	122.1	26	199.44	172.3	26	9.4%	−63.84 [−145.01, 17.33]
**Total (95% CI)**			**507**			**510**	**100.0%**	**−31.17 [−60.86, −1.49]**

Heterogeneity: $Tau^2 = 725.26$; $Chi^2 = 17.55$, df = 5 (P = 0.004); $I^2 = 72\%$
Test for overall effect: Z = 2.06 (P = 0.04)

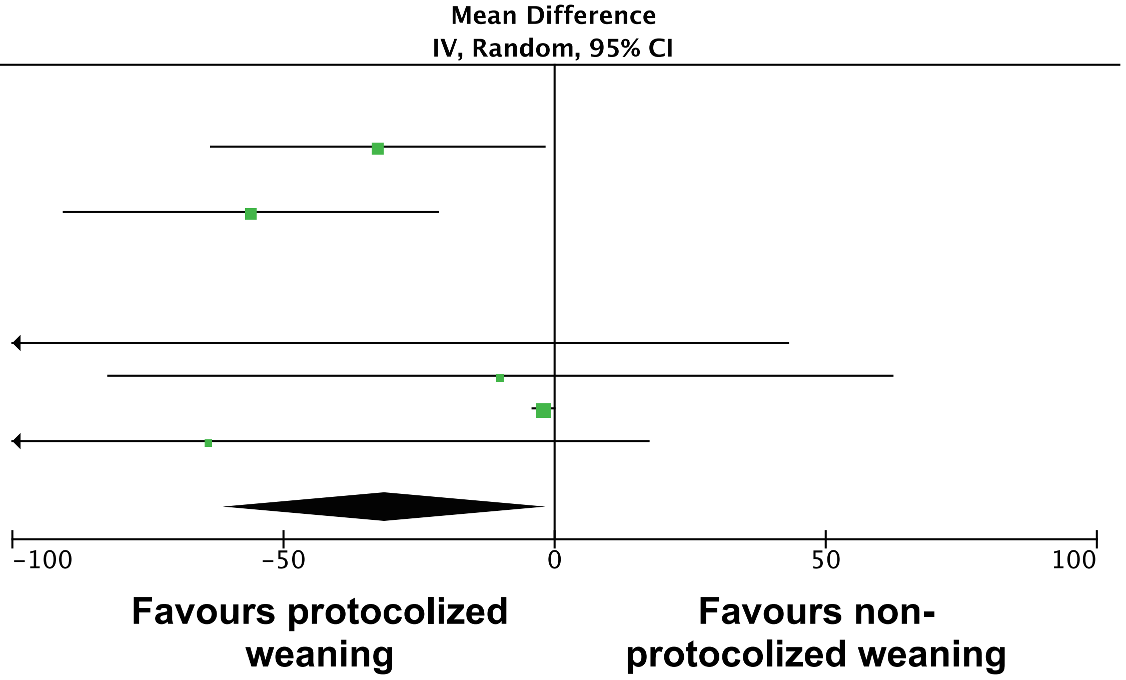

subgroup analysis
Mechanical ventilation time
(RCTs with professional-led weaning protocol)

Study or Subgroup	protocolized Mean	SD	Total	non-protocolized Mean	SD	Total	Weight	Mean Difference IV, Random, 95% CI
Chaiwat2010	98.5	176.76	51	255.71	868.28	49	0.5%	−157.21 [−405.12, 90.70]
Ely1996	151.2	175.2	149	211.2	261.6	151	8.4%	−60.00 [−110.32, −9.68]
Kollef1997	69.4	123.7	179	102	169.1	178	14.4%	−32.60 [−63.35, −1.85]
Krishnan2004	60.4	103	115	68	105.3	109	15.8%	−7.60 [−34.90, 19.70]
Marelich2000	68	97	166	124	207	169	13.0%	−56.00 [−90.52, −21.48]
Namen2001	144	124.45	49	144	195.56	51	6.0%	0.00 [−63.99, 63.99]
Navalesi2008	120	134.4	165	120	120	153	15.6%	0.00 [−27.97, 27.97]
Piotto2011	189.25	463.55	18	127.48	337.37	18	0.5%	61.77 [−203.09, 326.63]
Roh2012	277.23	320.66	61	424.3	686.42	61	0.9%	−147.07 [−337.19, 43.05]
Rose2008	119	174.89	51	129	197.07	51	0.0%	−10.00 [−82.31, 62.31]
Simeone2002	6.54	3.78	24	8.58	3.45	25	25.0%	−2.04 [−4.07, −0.01]
Stahl2009	135.6	122.1	26	199.44	172.3	26	0.0%	−63.84 [−145.01, 17.33]
**Total (95% CI)**			**977**			**964**	**100.0%**	**−20.54 [−38.47, −2.60]**

Heterogeneity: $Tau^2 = 334.55$; $Chi^2 = 22.23$, df = 9 (P = 0.008); $I^2 = 60\%$
Test for overall effect: Z = 2.24 (P = 0.02)

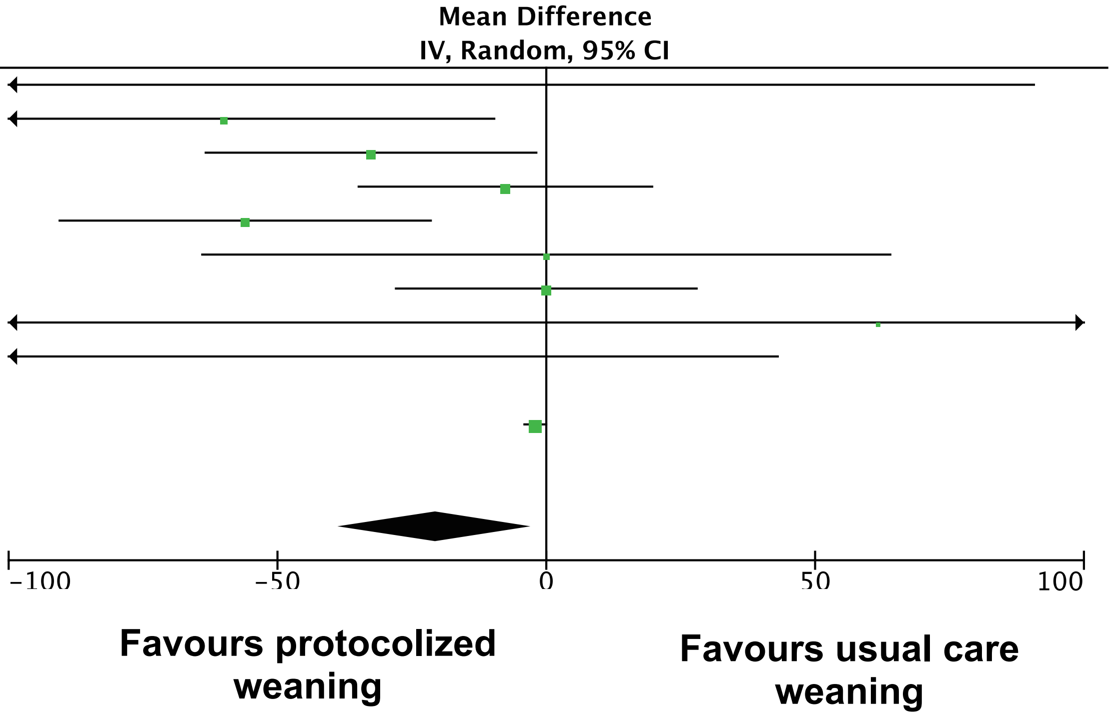

subgroup analysis
Mechanical ventilation time
(RCTs with computer-driven weaning protocol)

Study or Subgroup	protocolized Mean	SD	Total	non-protocolized Mean	SD	Total	Weight	Mean Difference IV, Random, 95% CI
Chaiwat2010	98.5	176.76	51	255.71	868.28	49	0.0%	−157.21 [−405.12, 90.70]
Ely1996	151.2	175.2	149	211.2	261.6	151	0.0%	−60.00 [−110.32, −9.68]
Kollef1997	69.4	123.7	179	102	169.1	178	0.0%	−32.60 [−63.35, −1.85]
Krishnan2004	60.4	103	115	68	105.3	109	0.0%	−7.60 [−34.90, 19.70]
Marelich2000	68	97	166	124	207	169	0.0%	−56.00 [−90.52, −21.48]
Namen2001	144	124.45	49	144	195.56	51	0.0%	0.00 [−63.99, 63.99]
Navalesi2008	120	134.4	165	120	120	153	0.0%	0.00 [−27.97, 27.97]
Piotto2011	189.25	463.55	18	127.48	337.37	18	0.0%	61.77 [−203.09, 326.63]
Roh2012	277.23	320.66	61	424.3	686.42	61	0.0%	−147.07 [−337.19, 43.05]
Rose2008	119	174.89	51	129	197.07	51	55.8%	−10.00 [−82.31, 62.31]
Simeone2002	6.54	3.78	24	8.58	3.45	25	0.0%	−2.04 [−4.07, −0.01]
Stahl2009	135.6	122.1	26	199.44	172.3	26	44.2%	−63.84 [−145.01, 17.33]
**Total (95% CI)**			**77**			**77**	**100.0%**	**−33.82 [−87.82, 20.17]**

Heterogeneity: $Tau^2 = 0.00$; $Chi^2 = 0.94$, df = 1 (P = 0.33); $I^2 = 0\%$
Test for overall effect: Z = 1.23 (P = 0.22)

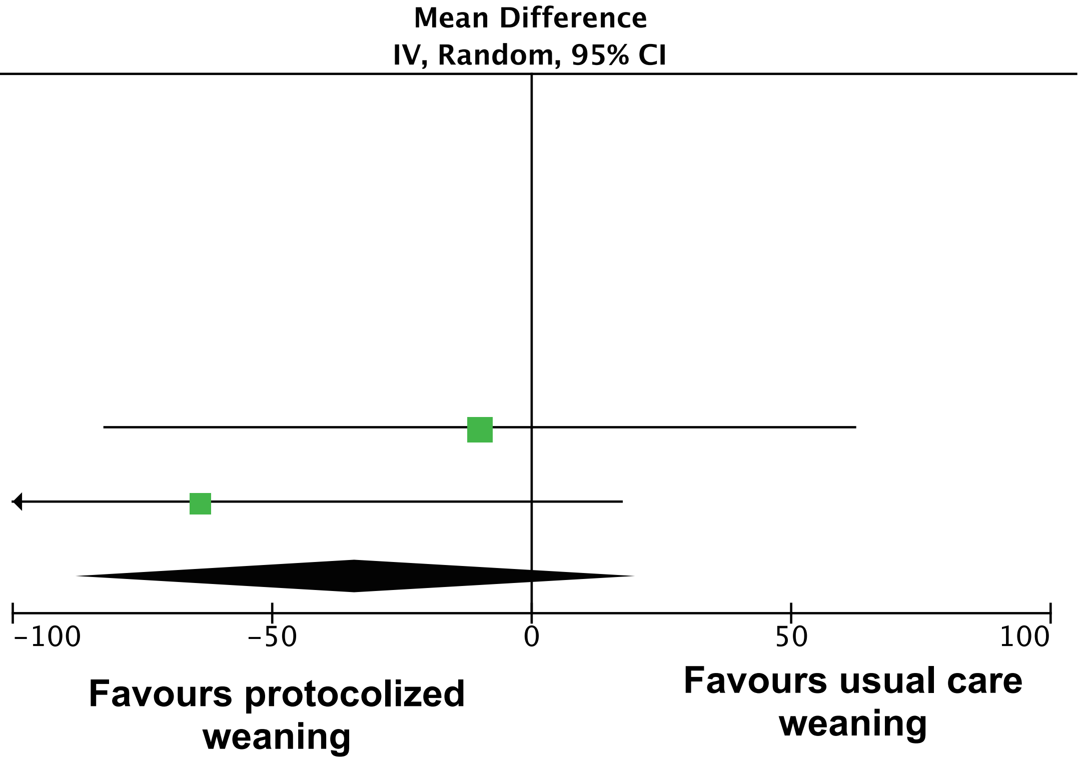

Mechanical Ventilation Time

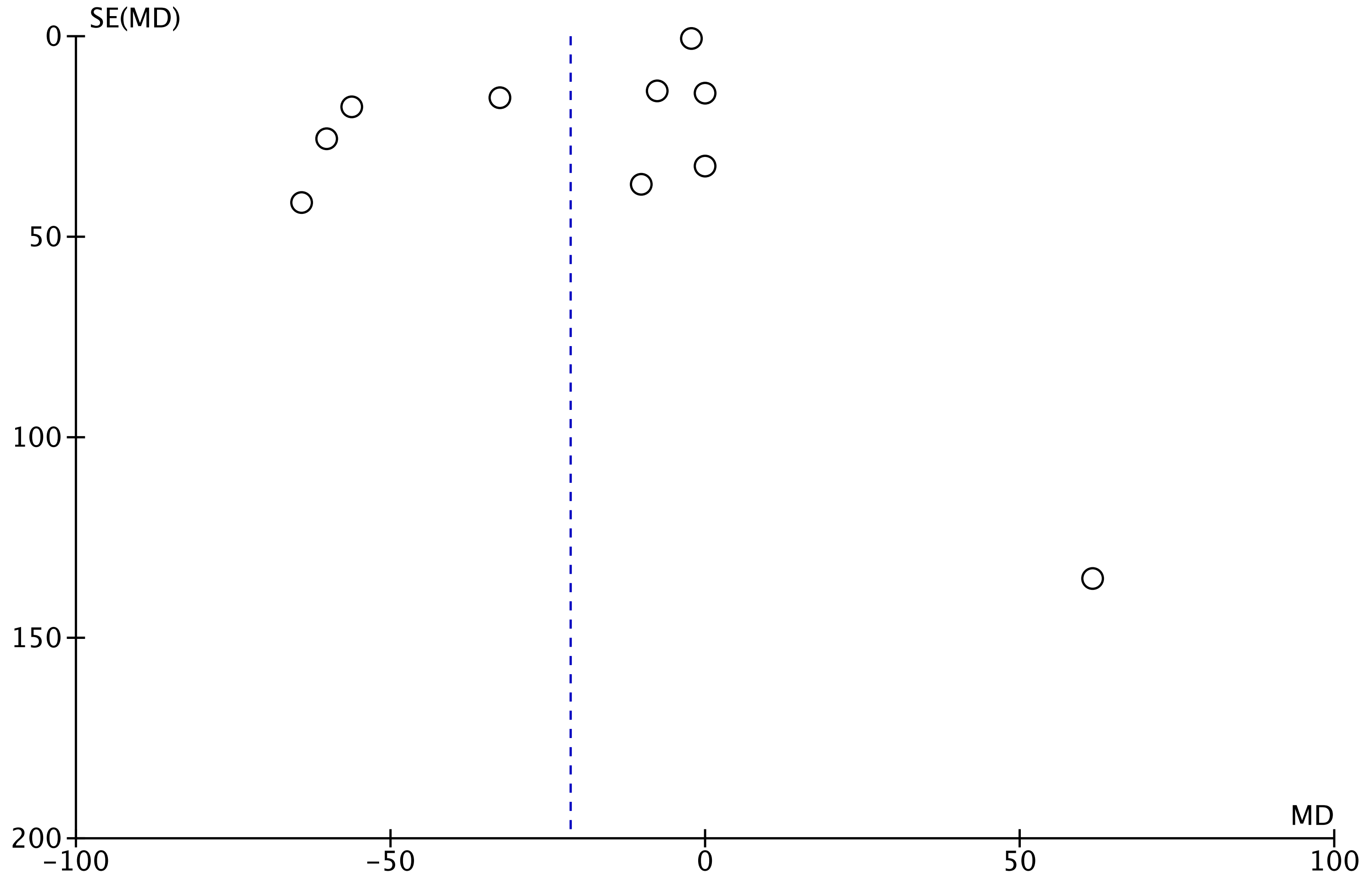

Summary of findings:

Protocolized methods for liberation from mechanical ventilation compared to non-protocolized for adult severe patients on ventilator

Patient: population: adult severe patients on ventilator
Intervention: Protocolized methods for liberation from mechanical ventilation
Comparison: non-protocolized methods for liberation from mechanical ventilation

Outcomes	Anticipated absolute effects*(95% CI)		Relative effect (95% CI)	No of participants (studies)	Quality of the evidence (GRADE)	Comments
	**Risk with non-protocolized**	**Risk with Protocolized**				
Mechanical Ventilation Time	The mean was 99.34	The mean in the intervention group was 21.51hours fewer (38.45hours fewer - 4.56hours fewer)	-	2095 (12 RCTs)	⊕○○○ VERY LOW [1,2,3]	
In hospital mortality	**Study population**		**RR 1.04** (0.88 ~ 1.23)	1573 (7 RCTs)	⊕⊕⊕○ MODERATE [3]	
	245 / 1000	**254 / 1000** (215 ~ 301)				
	**Low risk population**					
	166 / 1000	**173 / 1000** (146 ~ 204)				
	**High risk population**					
	331 / 1000	**344 / 1000** (291 ~ 407)				
Re-intubation	**Study population**		**RR 0.79** (0.50 ~ 1.26)	1414 (9 RCTs)	⊕⊕○○ LOW [1,3]	
	115 / 1000	**91 / 1000** (58 ~ 145)				
	**Low risk population**					
	61 / 1000	**48 / 1000** (31 ~ 77)				
	**High risk population**					
	230 / 1000	**182 / 1000** (115 ~ 290)				
Tracheostomy	**Study population**		**RR 0.72** (0.52 ~ 0.99)	1277 (6 RCTs)	⊕⊕○○ LOW [1,3]	
	126 / 1000	**91 / 1000** (65 ~ 125)				
	**Low risk population**					
	72 / 1000	**52 / 1000** (37 ~ 71)				
	**High risk population**					
	157 / 1000	**113 / 1000** (82 ~ 155)				

***The risk in the intervention group** (and its 95% confidence interval) is based on the assumed risk in the comparison group and the **relative effect** of the intervention (and its 95% CI).

CI: Confidence interval; **RR:** Risk ratio

GRADE Working Group grades of evidence
High quality: We are very confident that the true effect lies close to that of the estimate of the effect
Moderate quality: We are moderately confident in the effect estimate: The true effect is likely to be close to the estimate of the effect, but there is a possibility that it is substantially different
Low quality: Our confidence in the effect estimate is limited: The true effect may be substantially different from the estimate of the effect
Very low quality: We have very little confidence in the effect estimate: The true effect is likely to be substantially different from the estimate of effect

1. As none of the studied was blinded due to the nature of the design, the possibility of having impact on outcomes cannot be excluded and the risk of bias is high.
2. I^2=55% P=0.001
3. The included patients are “critically ill patients on mechanical ventilation” not only patients with ARDS.

CQ6:

Question: Should protocolized methods be used for liberation from mechanical ventilation in patients with ARDS?

Quality assessment							№ of patients		Effect		Quality	Importance
№ of studies	Study design	Risk of bias	Inconsistency	Indirectness	Imprecision	Other considerations	Protocolized	non-protocolized	Relative (95% CI)	Absolute (95% CI)		
Duration of mechanical ventilation												
12	randomised trials	serious [1]	serious [2]	not serious	not serious	none	1054	1041	-	MD **21.51 fewer** (4.56 fewer to 38.45 fewer)	⨁◯◯◯ VERY LOW [1,2,3]	CRITICAL
Hospital mortality												
7	randomised trials	not serious	not serious	not serious	not serious	none	203/788 (25.8%)	192/785 (24.5%)	**RR 1.04** (0.88 to 1.23)	10 more per 1000 (from 29 fewer to 56 more)	⨁⨁⨁◯ MODERATE [3]	CRITICAL
								17.0%		7 more per 1000 (from 20 fewer to 38 more)		
								33.0%		13 more per 1000 (from 40 fewer to 76 more)		
Re-intubation												
9	randomised trials	serious [1]	not serious	not serious	not serious	none	66/712 (9.3%)	81/702 (11.5%)	**RR 0.79** (0.50 to 1.26)	24 fewer per 1000 (from 30 more to 58 fewer)	⨁⨁◯◯ LOW [1,3]	CRITICAL
								6.1%		13 fewer per 1000 (from 16 more to 31 fewer)		
								23.0%		48 fewer per 1000 (from 60 more to 115 fewer)		
Tracheostomy												
6	randomised trials	serious [1]	not serious	not serious	not serious	none	56/641 (8.7%)	80/636 (12.6%)	**RR 0.72** (0.52 to 0.99)	35 fewer per 1000 (from 1 fewer to 60 fewer)	⨁⨁◯◯ LOW [1,3]	IMPORTANT
								7.2%		20 fewer per 1000 (from 1 fewer to 35 fewer)		
								15.7%		44 fewer per 1000 (from 2 fewer to 75 fewer)		

MD – mean difference, RR – relative risk

RR – Relative risk

1. As none of the studied was blinded due to the nature of the design, the possibility of having impact on outcomes cannot be excluded and the risk of bias is high.
2. I_2=55% P=0.001
3. The included patients are “critically ill patients on mechanical ventilation” not only patients with ARDS.

10. Evidence-to-Decision

<table>
<tr><td colspan="5">CQ6 : **Should liberation from mechanical ventilation be protocolized in patients with ARDS?**</td></tr>
<tr><td colspan="5">PUPULATION : CRITICAL ILL PATIENTS UNDERGOING MECHANICAL VENTILATION</td></tr>
<tr><td colspan="5">INTERVENTION : EARLY TRACHEOSTOMY</td></tr>
<tr><td colspan="2">CRITERIA</td><td>JUDGEMENT</td><td>RESEARCH EVIDENCE</td><td>ADDITIONAL CONSIDERATIONS</td></tr>
<tr><td>PROBLEM</td><td>**Is the problem a priority?**</td><td>◯No
◯Probably no
●Probably yes
◯Yes
―――――
◯Varies
◯Don't know</td><td>Since the process of liberation (formerly referred to as "weaning") from mechanical ventilation is not standardized in Japan, it is likely that a large number of patients remain on mechanical ventilation longer than necessary. It is suggested that the use of protocols for liberation from mechanical ventilation will prevent unnecessarily prolonged mechanical ventilation with a significant reduction in the duration of mechanical ventilation. Many patients with ARDS require a long period of mechanical ventilation and they would greatly benefit if the use of liberation protocols is effective in shortening the period of mechanical ventilation.</td><td></td></tr>
<tr><td rowspan="2">DESIRABLE AND UNDESIRABLE EFFECTS</td><td>**What is the overall certainty of the evidence of effects?**</td><td>●Very low
◯Low
◯Moderate
◯High
―――――
◯No included studies</td><td rowspan="2">**The relative importance or values of the main outcomes of interest:**
<table><tr><th>Outcome</th><th>Relative importance</th><th>Certainty of the evidence (GRADE)</th></tr><tr><td>Duration of mechanical ventilation</td><td>Critical</td><td>⨁◯◯◯
Very low</td></tr><tr><td>Hospital mortality</td><td>Critical</td><td>⨁⨁⨁◯
Moderate</td></tr><tr><td>Re-intubation</td><td>Critical</td><td>⨁⨁◯◯
Low</td></tr><tr><td>Tracheostomy</td><td>Important</td><td>⨁⨁◯◯
Low</td></tr></table></td><td></td></tr>
<tr><td>**Is there important uncertainty about or variability in how much people value the main outcomes?**</td><td>◯Important uncertainty or variability
◯Possibly important uncertainty or variability
◯Possibly no important uncertainty or variability
●No important uncertainty or variability
―――――
◯No known undesirable outcomes</td><td></td></tr>
</table>

How substantial are the desirable anticipated effects?

○Trivial
○Small
●Moderate
○Large
―――――――
○Varies
○Don't know

How substantial are the undesirable anticipated effects?

○Large
○Moderate
●Small
○Trivial
―――――――
○Varies
○Don't know

Does the balance between desirable and undesirable effects favor the intervention or the comparison?

○Favors the comparison
●Probably favors the comparison
○Does not favor either the intervention or the comparison
○Probably favors the intervention
○Favors the intervention
―――――――
○Varies
○Don't know

Summary of findings:

Outcome	Late	Early	Absolute effect (95% CI)	Relative effect (RR) (95% CI)
Duration of mechanical ventilation	Average 99.34 hours	Average 77.62 hours	Average 21.51hours fewer (38.45hours fewer - 4.56 hours fewer)	
Hospital mortality	245 / 1000	254 / 1000 (215 ～ 301)	10 more／ 1000 (29 fewer- 56 more)	RR 1.04 (0.88～1.23)
	166 / 1000	173 / 1000 (146～204)	7 more／1000 (20 fewer- 38 more)	
	331 / 1000	344 / 1000 (291～407)	13 more／1000 (40 fewer-76 more)	
IRe-intubation	115 / 1000	91 / 1000 (58 ～ 145)	24 fewer／1000 (58 fewer-30 more)	RR 0.79 (0.50～1.26)
	61 / 1000	48 / 1000 (31～77)	13 fewer／1000 (31 fewer-16more)	
	230 / 1000	182 / 1000 (115～290)	48 fewer／1000 (115 fewer～60 more)	
Tracheostomy	126 / 1000	91 / 1000 (65 ～ 125)	35 fewer／1000 (60 fewer- 1 fewer)	RR 0.72 (0.52～0.99)
	72 / 1000	52 / 1000 (37～71)	20 fewer／1000 (35 fewer- 1 fewer)	
	157 / 1000	113 / 1000 (82～155)	44 fewer／1000 (75 fewer-2 fewer)	

Summary: The meta-analysis shows a significantly shorter duration of mechanical ventilation in patients liberated according to a protocol compared to patients liberated without a protocol. It also shows that protocolized liberation from mechanical ventilation significantly reduced the number of tracheostomies needed

RESOURCES REQUIRED

How large are the resource requirements (costs)?

○Large costs
○Moderate costs
○Negligible costs and savings
○Moderate savings
○Large savings
―――――――
●Varies

The cost of using liberation protocols is expected to be minimal except when protocols programmed into the ventilators are used.

		◯Don't know		
	**Does the cost-effectiveness of the intervention favor the intervention or the comparison?**	◯Favors the comparison<br>◯Probably favors the comparison<br>◯Does not favor either the intervention or the comparison<br>◯Probably favors the intervention<br>◯Favors the intervention<br>―――――<br>●Varies<br>◯No included studies	The benefits are expected to outweigh the costs or resources needed when liberation protocols programmed into the ventilator are not used. Development of protocols and education of staff to apply a protocol may incur some cost.	
**EQUITY**	**What would be the impact on health equity?**	◯Reduced<br>●Probably reduced<br>◯Probably no impact<br>◯Probably increased<br>◯Increased<br>―――――<br>◯Varies<br>◯Don't know	There may be some difficulty in developing and initiating protocols among facilities.	
**ACCEPTABILITY**	**Is the intervention acceptable to key stakeholders?**	◯No<br>◯Probably no<br>●Probably yes<br>◯Yes<br>―――――<br>◯Varies<br>◯Don't know	Since the patients' burden and incidence of adverse events are not likely to increase and the time needed for mechanical ventilation is expected to decrease by applying the protocols, it should be acceptable.	
**FEASIBILITY**	**Is the intervention feasible implement?**	◯No<br>◯Probably no<br>●◯Probably yes<br>◯Yes<br>―――――<br>◯Varies<br>◯Don't know	It is feasible to initiate and establish liberation protocols.	

Recommendations

CQ6：Should liberation from mechanical ventilation be protocolized in patients with ARDS?					
Balance of consequences	Undesirable consequences *clearly outweigh* desirable consequences in most settings	Undesirable consequences *probably outweigh* desirable consequences in most settings	The balance between desirable and undesirable consequences is closely *balanced or uncertain*	Desirable consequences *probably outweigh* undesirable consequences in most settings	Desirable consequences *clearly outweigh* undesirable consequences in most settings
**Judgement**	○	○	○	●	○

Type of recommendation	We recommend against offering this option	We suggest not offering this option	We suggest offering this option	We recommend offering this option
**Judgement**	○	○	●	○
Recommendation	**Recommendation: We suggest using protocolized methods for liberation from mechanical ventilation in patients with ARDS (Grade 2D, Strength of recommendation "weak recommendation" / Quality of evidence "Very low").**<br>●Supplementary statements:<br>When developing protocols for liberation, the level of knowledge and skills of the personnel who apply the protocol in each facility must be taken into account. Education and training regarding mechanical ventilation are required, especially for non-physician staff members. A previous meta-analysis[1] showed a reduction in the duration of mechanical ventilation for patients in medical, surgical and medical/surgical ICUs but not in a neurological ICU.			
Justification	**Question:** Should liberation from mechanical ventilation be protocolized in patients with ARDS?<br>**Patients:** CRITICAL ILL PATIENTS UNDERGOING MECHANICAL VENTILATION<br>**Interventions:** Protocolized liberation<br>**Comparison:** Non-protocolized liberation<br>**Outcomes:** Duration of mechanical ventilation、hospital mortality、re-intubation、tracheostomy<br><br>**Summary of the evidence:** Since this systematic review revealed that there are no previous studies which evaluated only patients with ARDS, we included 12 RCTs that included critical ill patients undergoing mechanical ventilation in this meta-analysis. The meta-analysis shows a significantly shorter duration of mechanical ventilation in patients liberated according to a protocol compared to patients liberated without a protocol (average difference -21.51 hours 95%CI -38.45 - -4.56 hours). It also shows that protocolized liberation from mechanical ventilation significantly reduced the number of tracheostomies needed（RR 0.72, 95%CI 0.52-0.99）. There were no significant differences in the incidence of adverse events between the two groups（re-intubation：RR 0.79, 95%CI 0.50-1.26, hospital mortality：RR 1.04, 95%CI 0.88-1.23）.<br><br>**Quality of the evidence:** The results of this meta-analysis must be cautiously applied to clinical practice as it includes studies that included "critically ill patients on mechanical ventilation" not "patients with ARDS" resulting in the inclusion of a large variety of patients, including those in medical, surgical and neurological ICUs. The heterogeneity of the analysis is high（p=0.01, I^2=55%）leading to downgrading of the evidence. As none of the studies was blinded due to their design, the possibility of having an impact on outcomes cannot be excluded and the risk of bias is high. As a result, the confidence level on the overall quality of evidence was rated as "very low".<br><br>**Judgement of benefit and harm, resources and cost:** The benefits are expected to overweigh the harms, as the initiation of liberation protocols is less likely to increase the patients' burden or incidence of adverse events. The cost of using liberation protocols is expected to be minimal except when protocols programmed into the ventilators are used. Development of			

	protocols and education of staff to apply a protocol may incur some cost.<br><br>**<u>Recommendations;</u> We suggest using protocolized methods for liberation from mechanical ventilation in patients with ARDS (Grade 2D, Strength of recommendation "weak recommendation" / Quality of evidence "Very low").**<br><br>**<u>Additional considerations:</u>** A previous meta-analysis[1] showed a reduction in the duration of mechanical ventilation for patients in medical, surgical and medical/surgical ICUs but not in a neurological ICU.
**Subgroup considerations**	Liberation protocols are divided into two groups, "step-wise reduction of mechanical ventilator support protocols" and "spontaneous breathing trial (SBT) protocols". Another way to classify liberation protocols is to divide them into "professional-led protocols" where staff such as nurses or respiratory therapists change ventilator settings based on protocols and "computer-driven protocols" where the settings are changed automatically based on computer programs built into the ventilators. Although it was decided that another panel discussion be held to reassess the recommendation after subgroup analyses are conducted, the recommendation did not require any change based on the subgroup analyses.
**Implementation considerations**	When developing protocols for liberation, the level of knowledge and skills of the personnel who apply the protocol in each facility must be taken into account. Education and training regarding mechanical ventilation are required, especially for non-physician staff members.
**Monitoring and evaluation considerations**	In addition to respiratory and hemodynamic parameters, respiratory patterns and patient's facial expressions need to be observed.
**Research possibilities**	Studies including only patients with ARDS are needed. It is also necessary to identify subgroups which may benefit more or less from liberation protocols.

References

1. Blackwood B, Burns KE, Cardwell CR, et al. Protocolized versus non-protocolized weaning for reducing the duration of mechanical ventilation in critically ill adult patients. *Cochrane Database Syst Rev* **11**: CD006904, 2014. PMID 25375085

CQ07. Study flow diagram

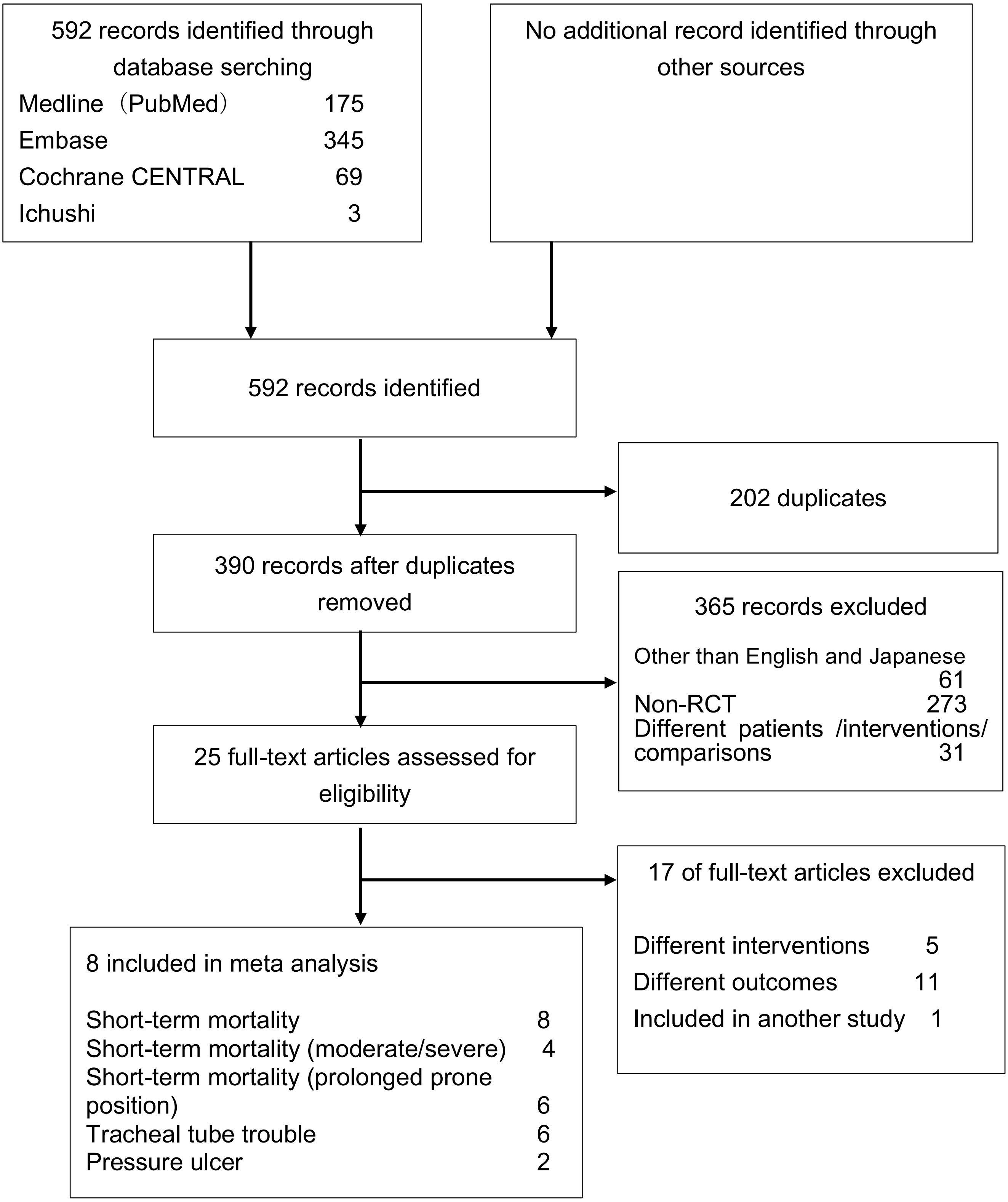

CQ07
Risk of bias table, Short term mortality

Outcome		Short term mortality		risk of bias		not serious (0)			
番号	著者名 発表年（Forest plot表示）	risk of bias評価							
		ランダム割付順番の生成 random sequence generation	割り付けの隠蔽化 allocation concealment	ブラインド blinding<br>研究参加者と治療提供者 participants and personnel	ブラインド blinding<br>アウトカム評価者 outcome assessors	不完全なアウトカムデータ incomplete outcome data	選択されたアウトカムの報告 selective outcome reporting	その他のバイアス Other sources of bias	研究内でのバイアスのリスク Risk of bias within a study
1	Beuret 2002	Unclear risk	Unclear risk	Unclear risk	Low risk	Low risk	Unclear risk	High risk	Unclear risk
2	Fernandez 2008	Low risk	Low risk	Unclear risk	Low risk	Low risk	Unclear risk	High risk	Unclear risk
3	Gattinoni 2001	Low risk	Low risk	Unclear risk	Low risk	Low risk	Unclear risk	High risk	Low risk
4	Guerin 2004	Low risk	Low risk	High risk	Low risk	Low risk	Unclear risk	High risk	Unclear risk
5	Guerin 2013	Low risk	Low risk	High risk	Low risk	Low risk	Low risk	Low risk	Low risk
6	Mancebo 2006	Low risk	Low risk	Unclear risk	Low risk	Low risk	Unclear risk	High risk	Unclear risk
7	Taccone 2009	Low risk	Low risk	Unclear risk	Low risk	Low risk	Low risk	High risk	Low risk
8	Voggenreiter 2005	Low risk	Low risk	Unclear risk	Low risk	Low risk	Unclear risk	Low risk	Low risk

番号	著者名 発表年（Forest plot表示）	risk of biasコメント							
1	Beuret 2002	記載なし	記載なし	記載なし	記載ないが影響なし	Intention to treat analysisで、欠損数<10%	Pre-registration dataなし	crossover許容	unclearが多く、crossoverの影響も不明
2	Fernandez 2008	computer-generated random sequence	a centralized call center	記載なし	記載ないが影響なし	Intention to treat analysisで、欠損数<10%	Pre-registration dataなし	必要サンプルサイズは250であったが、初年度に42人しか参加がなく、早期中止<br>crossover許容	早期中止やcrossoverの影響が不明
3	Gattinoni 2001	permuted-block algorithm	centrally by telephone	記載なし	記載ないが影響なし	Intention to treat analysisで、欠損数<10%	Pre-registration dataなし	先行1年で次第に参加者が減り、必要サンプルサイズは死亡者95人であったが、70人の時点で早期中止	早期中止されたものの、8割弱リクルートできており、結果の解釈への影響は大きくはない
4	Guerin 2004	computer-generated	sequentially numbered, opaque, and sealed envelopes	data collectors were not blinded	outcomes assessors were not blinded、ただし影響なし	Intention to treat analysisで、欠損数<10%	Pre-registration dataなし	crossover許容	crossoverの影響不明
5	Guerin 2013	computer-generated	centralized Web-based management system	data collectors were aware of study group assignments	outcomes assessors were not aware of study group assignments	Intention to treat analysisで、欠損数<10%	Pre-registration dataの通り	特記事項なし（COIあり）<br>crossoverなし	治療者のblindingはないが影響は小さい
6	Mancebo 2006	computer-generated	concealment was performed using sealed opaque envelopes	記載なし	記載ないが影響なし	Intention to treat analysisで、欠損数<10%	Pre-registration dataなし	必要サンプルサイズは200であったが、登録数の減少により、早期中止<br>crossover許容<br>著者の一人はRotaprone Bed製造業者(KCI)のコンサルタントで報酬を得ているが、この研究にRotaprone Bedは使用されておらず、製造業者の関与なし	早期中止や利益相反の研究への影響は不明
7	Taccone 2009	computer-generated with a permuted-block algorithm	a centralized telephone randomization system	記載なし	outcome data were available during the study only to the members of the data and safety monitoring board for interim analysis、ただし影響なし	Intention to treat analysisで、欠損数<10%	Pre-registration dataの通り	crossover許容<br>Rotoprone rotational bedの製造業者(KCI)により同ベッドが無料で提供され、本研究の20施設で使用された。KCIは研究のコーディネートセンターや研究者の定例会議の秘書業務を担ったが、研究自体への関与なし<br>著者の一人はKCI本部諮問会議委員として報酬を得ている	crossoverや利益相反の研究への影響は不明
8	Voggenreiter 2005	permuted-block algorithm	centrally by telephone	記載なし	記載ないが影響なし	Intention to treat analysisで、欠損数<10%	Pre-registration dataなし	特記事項なし	unclearな要素もあるが、全体的にriskは低い

CQ07
Risk of bias table, Short-term mortality (moderate/ severe)

Outcome		Short-term mortality (moderate/ severe)		risk of bias		not serious (0)			
番号	著者名 発表年（Forest plot表示）	risk of bias評価							
		ランダム割付順番の生成 random sequence generation	割り付けの隠蔽化 allocation concealment	ブラインド blinding 研究参加者と治療提供者 participants and personnel	ブラインド blinding アウトカム評価者 outcome assessors	不完全なアウトカムデータ incomplete outcome data	選択されたアウトカムの報告 selective outcome reporting	その他のバイアス Other sources of bias	研究内でのバイアスのリスク Risk of bias within a study
1	Fernandez 2008	Low risk	Low risk	Unclear risk	Low risk	Low risk	Unclear risk	High risk	Unclear risk
2	Guerin 2013	Low risk	Low risk	High risk	Low risk	Low risk	Low risk	Low risk	Low risk
3	Mancebo 2006	Low risk	Low risk	Unclear risk	Low risk	Low risk	Unclear risk	High risk	Unclear risk
4	Taccone 2009	Low risk	Low risk	Unclear risk	Low risk	Low risk	Low risk	High risk	Low risk

番号	著者名 発表年（Forest plot表示）	risk of biasコメント							
1	Fernandez 2008	computer-generated random sequence	a centralized call center	記載なし	記載ないが影響なし	Intention to treat analysisで、欠損数<10%	Pre-registration dataなし	必要サンプルサイズは250であったが、初年度に42人しか参加がなく、早期中止<br>crossover許容	早期中止やcrossoverの影響が不明
2	Guerin 2013	computer-generated	centralized Web-based management system	data collectors were aware of study group assignments	outcomes assessors were not aware of study group assignments	Intention to treat analysisで、欠損数<10%	Pre-registration dataの通り	特記事項なし（COIあり）<br>crossoverなし	治療者のblindingはないが影響は小さい
3	Mancebo 2006	computer-generated	concealment was performed using sealed opaque envelopes	記載なし	記載ないが影響なし	Intention to treat analysisで、欠損数<10%	Pre-registration dataなし	必要サンプルサイズは200であったが、登録数の減少により、早期中止<br>crossover許容<br>著者の一人はRotaprone Bed製造業者(KCI)のコンサルタントで報酬を得ているが、この研究にRotaprone Bedは使用されておらず、製造業者の関与なし	早期中止や利益相反の研究への影響は不明
4	Taccone 2009	computer-generated with a permuted-block algorithm	a centralized telephone randomization system	記載なし	outcome data were available during the study only to the members of the data and safety monitoring board for interim analysis、ただし影響なし	Intention to treat analysisで、欠損数<10%	Pre-registration dataの通り	crossover許容<br>Rotoprone rotational bedの製造業者(KCI)により同ベッドが無料で提供され、本研究の20施設で使用された。KCIは研究のコーディネートセンターや研究者の定例会議の秘書業務を担ったが、研究自体への関与なし<br>著者の一人はKCI本部諮問会議委員として報酬を得ている	crossoverや利益相反の研究への影響は不明

CQ07
Risk of bias table, Short-term mortality (prolonged prone)

Outcome		Short-term mortality (prolonged prone)		risk of bias		not serious (0)			
番号	著者名 発表年<br>(Forest plot表示)	risk of bias評価							
		ランダム割付順番の生成<br>random sequence generation	割り付けの隠蔽化<br>allocation concealment	ブラインド<br>blinding		不完全なアウトカムデータ<br>incomplete outcome data	選択されたアウトカムの報告<br>selective outcome reporting	その他のバイアス<br>Other sources of bias	研究内でのバイアスのリスク<br>Risk of bias within a study
				研究参加者と治療提供者<br>participants and personnel	アウトカム評価者<br>outcome assessors				
1	Fernandez 2008	Low risk	Low risk	Unclear risk	Low risk	Low risk	Unclear risk	High risk	Unclear risk
2	Guerin 2004	Low risk	Low risk	High risk	Low risk	Low risk	Unclear risk	High risk	Unclear risk
3	Guerin 2013	Low risk	Low risk	High risk	Low risk	Low risk	Low risk	Low risk	Low risk
4	Mancebo 2006	Low risk	Low risk	Unclear risk	Low risk	Low risk	Unclear risk	High risk	Unclear risk
5	Taccone 2009	Low risk	Low risk	Unclear risk	Low risk	Low risk	Low risk	High risk	Low risk
6	Voggenreiter 2005	Low risk	Low risk	Unclear risk	Low risk	Low risk	Unclear risk	Low risk	Low risk

番号	著者名 発表年<br>(Forest plot表示)	risk of biasコメント							
1	Fernandez 2008	computer-generated random sequence	a centralized call center	記載なし	記載ないが影響なし	Intention to treat analysisで、欠損数<10%	Pre-registration dataなし	必要サンプルサイズは250であったが、初年度に42人しか参加がなく、早期中止<br>crossover許容	早期中止やcrossoverの影響が不明
2	Guerin 2004	computer-generated	sequentially numbered, opaque, and sealed envelopes	data collectors were not blinded	outcomes assessors were not blinded、ただし影響なし	Intention to treat analysisで、欠損数<10%	Pre-registration dataなし	crossover許容	crossoverの影響不明
3	Guerin 2013	computer-generated	centralized Web-based management system	data collectors were aware of study group assignments	outcomes assessors were not aware of study group assignments	Intention to treat analysisで、欠損数<10%	Pre-registration dataの通り	特記事項なし(COIあり)<br>crossoverなし	治療者のblindingはないが影響は小さい
4	Mancebo 2006	computer-generated	concealment was performed using sealed opaque envelopes	記載なし	記載ないが影響なし	Intention to treat analysisで、欠損数<10%	Pre-registration dataなし	必要サンプルサイズは200であったが、登録数の減少により、早期中止<br>crossover許容<br>著者の一人はRotaprone Bed製造業者(KCI)のコンサルタントで報酬を得ているが、この研究にRotaprone Bedは使用されておらず、製造業者の関与なし	早期中止や利益相反の研究への影響は不明
5	Taccone 2009	computer-generated with a permuted-block algorithm	a centralized telephone randomization system	記載なし	outcome data were available during the study only to the members of the data and safety monitoring board for interim analysis、ただし影響なし	Intention to treat analysisで、欠損数<10%	Pre-registration dataの通り	crossover許容<br>Rotoprone rotational bedの製造業者(KCI)により同ベッドが無料で提供され、本研究の20施設で使用された。KCIは研究のコーディネートセンターや研究者の定例会議の秘書業務を担ったが、研究自体への関与なし<br>著者の一人はKCI本部諮問会議委員として報酬を得ている	crossoverや利益相反の研究への影響は不明
6	Voggenreiter 2005	permuted-block algorithm	centrally by telephone	記載なし	記載ないが影響なし	Intention to treat analysisで、欠損数<10%	Pre-registration dataなし	特記事項なし	unclearな要素もあるが、全体的にriskは低い

CQ07
Risk of bias table, Tracheal tube trouble

Outcome		Tracheal tube trouble		risk of bias		serious (−1)			
番号	著者名 発表年<br>（Forest plot表示）	risk of bias評価							
		ランダム割付順番の生成<br>random sequence generation	割り付けの隠蔽化<br>allocation concealment	ブラインド<br>blinding<br>研究参加者と治療提供者<br>participants and personnel	ブラインド<br>blinding<br>アウトカム評価者<br>outcome assessors	不完全なアウトカムデータ<br>incomplete outcome data	選択されたアウトカムの報告<br>selective outcome reporting	その他のバイアス<br>Other sources of bias	研究内でのバイアスのリスク<br>Risk of bias within a study
1	Fernandez 2008	Low risk	Low risk	Unclear risk	Low risk	Low risk	Unclear risk	High risk	Unclear risk
2	Gattinoni 2001	Low risk	Low risk	Unclear risk	Low risk	Low risk	Unclear risk	High risk	Low risk
3	Guerin 2013	Low risk	Low risk	High risk	Low risk	Low risk	High risk	Low risk	Unclear risk
4	Mancebo 2006	Low risk	Low risk	Unclear risk	Low risk	Low risk	Unclear risk	High risk	Unclear risk
5	Taccone 2009	Low risk	Low risk	Unclear risk	Low risk	Low risk	High risk	High risk	High risk
6	Voggenreiter 2005	Low risk	Low risk	Unclear risk	Low risk	Low risk	Unclear risk	Low risk	Low risk

番号	著者名 発表年<br>（Forest plot表示）	risk of biasコメント							
1	Fernandez 2008	computer-generated random sequence	a centralized call center	記載なし	記載ないが影響なし	Intention to treat analysisで、欠損数<10%	Pre-registration dataなし	必要サンプルサイズは250であったが、初年度に42人しか参加がなく、早期中止<br>crossover許容	早期中止やcrossoverの影響が不明
2	Gattinoni 2001	permuted-block algorithm	centrally by telephone	記載なし	記載ないが影響なし	Intention to treat analysisで、欠損数<10%	Pre-registration dataなし	先行1年で次第に参加者が減り、必要サンプルサイズは死亡者95人であったが、70人の時点で早期中止	早期中止されたものの、8割弱リクルートできており、結果の解釈への影響は大きくはない
3	Guerin 2013	computer-generated	centralized Web-based management system	data collectors were aware of study group assignments	outcomes assessors were not aware of study group assignments	Intention to treat analysisで、欠損数<10%	Pre-registration dataに合併症の記載なし	特記事項なし（COIあり）<br>crossoverなし	事前計画になかったアウトカムであり、結果の解釈への影響不明
4	Mancebo 2006	computer-generated	concealment was performed using sealed opaque envelopes	記載なし	記載ないが影響なし	Intention to treat analysisで、欠損数<10%	Pre-registration dataなし	必要サンプルサイズは200であったが、登録数の減少により、早期中止<br>crossover許容<br>著者の一人はRotaprone Bed製造業者(KCI)のコンサルタントで報酬を得ているが、この研究にRotaprone Bedは使用されておらず、製造業者の関与なし	早期中止や利益相反の研究への影響は不明
5	Taccone 2009	computer-generated with a permuted-block algorithm	a centralized telephone randomization system	記載なし	outcome data were available during the study only to the members of the data and safety monitoring board for interim analysis、ただし影響なし	Intention to treat analysisで、欠損数<10%	Pre-registration dataに合併症の記載なし	crossover許容<br>Rotoprone rotational bedの製造業者(KCI)により同ベッドが無料で提供され、本研究の20施設で使用された。KCIは研究のコーディネートセンターや研究者の定例会議の秘書業務を担ったが、研究自体への関与なし<br>著者の一人はKCI本部諮問会議委員として報酬を得ている	事前計画になかったアウトカムであり、crossoverや利益相反もあり、biasのriskは高い
6	Voggenreiter 2005	permuted-block algorithm	centrally by telephone	記載なし	記載ないが影響なし	Intention to treat analysisで、欠損数<10%	Pre-registration dataなし	特記事項なし	unclearな要素もあるが、全体的にriskは低い

CQ07
Risk of bias table, Pressure ulcer

Outcome		Pressure ulcer		risk of bias		not serious (0)			
番号	著者名 発表年 (Forest plot表示)	risk of bias評価							
		ランダム割付順番の生成 random sequence generation	割り付けの隠蔽化 allocation concealment	ブラインド blinding 研究参加者と治療提供者 participants and personnel	ブラインド blinding アウトカム評価者 outcome assessors	不完全なアウトカムデータ incomplete outcome data	選択されたアウトカムの報告 selective outcome reporting	その他のバイアス Other sources of bias	研究内でのバイアスのリスク Risk of bias within a study
1	Gattinoni 2001	Low risk	Low risk	Unclear risk	Low risk	Low risk	Unclear risk	High risk	Low risk
2	Voggenreiter 2005	Low risk	Low risk	Unclear risk	Low risk	Low risk	Unclear risk	Low risk	Low risk

番号	著者名 発表年 (Forest plot表示)	risk of biasコメント							
1	Gattinoni 2001	permuted-block algorithm	centrally by telephone	記載なし	記載ないが定義あり影響なし	Intention to treat analysisで、欠損数<10%	Pre-registration dataなし	先行1年で次第に参加者が減り、必要サンプルサイズは死亡者95人であったが、70人の時点で早期中止	早期中止されたものの、8割弱リクルートできており、結果の解釈への影響は大きくはない
2	Voggenreiter 2005	permuted-block algorithm	centrally by telephone	記載なし	記載ないが影響なし	Intention to treat analysisで、欠損数<10%	Pre-registration dataなし	特記事項なし	unclearな要素もあるが、全体的にriskは低い

CQ07 Risk of bias summary, Risk of bias graph

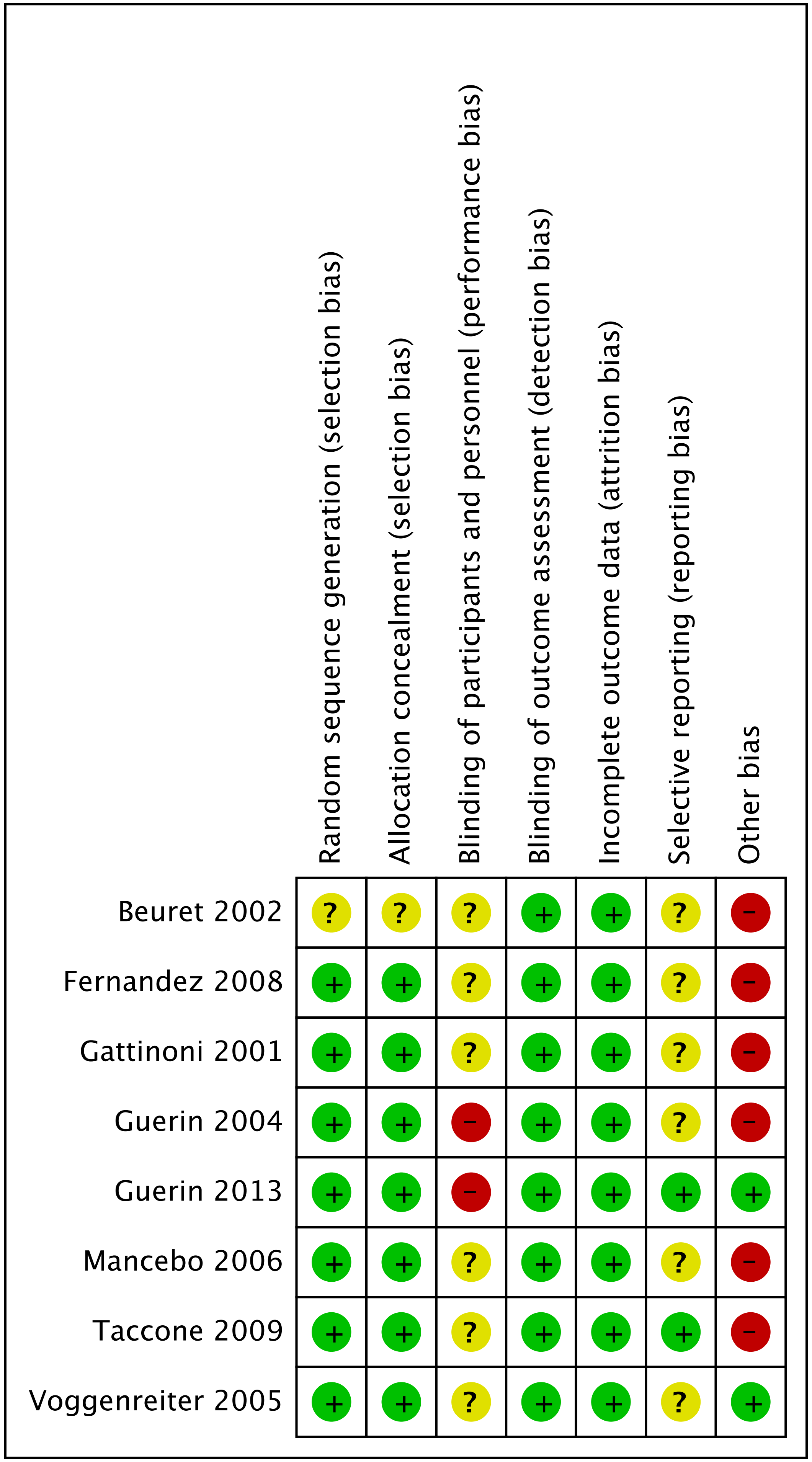

Short term mortality

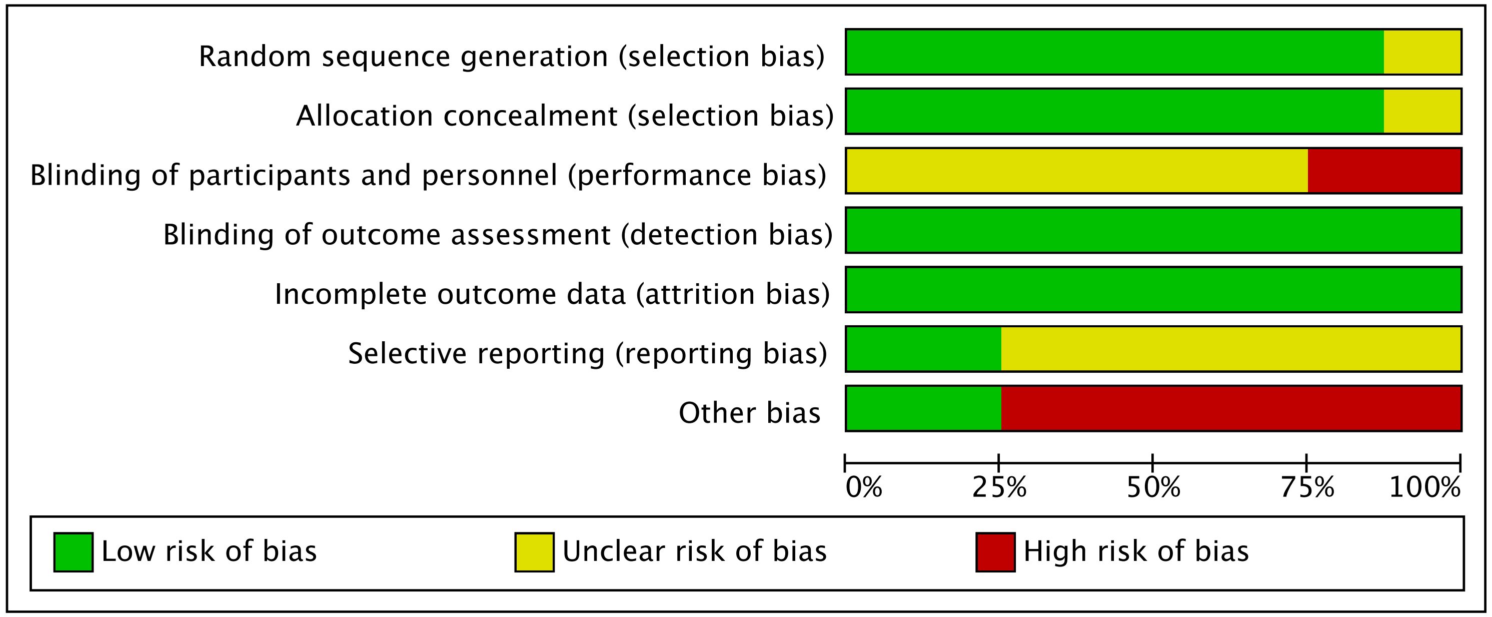

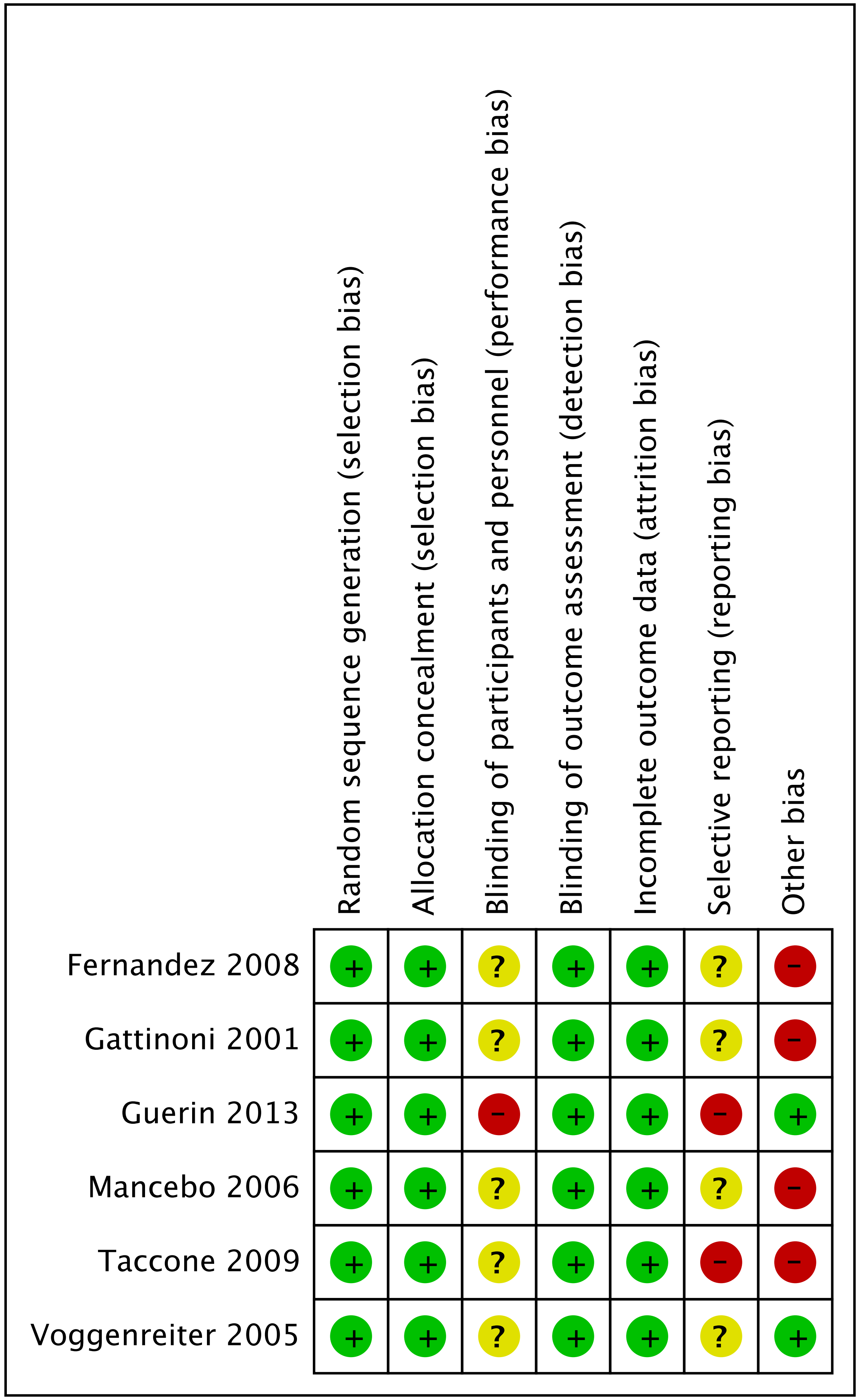

Complications on tracheal tube (accidental extubation, dislocation of tube)

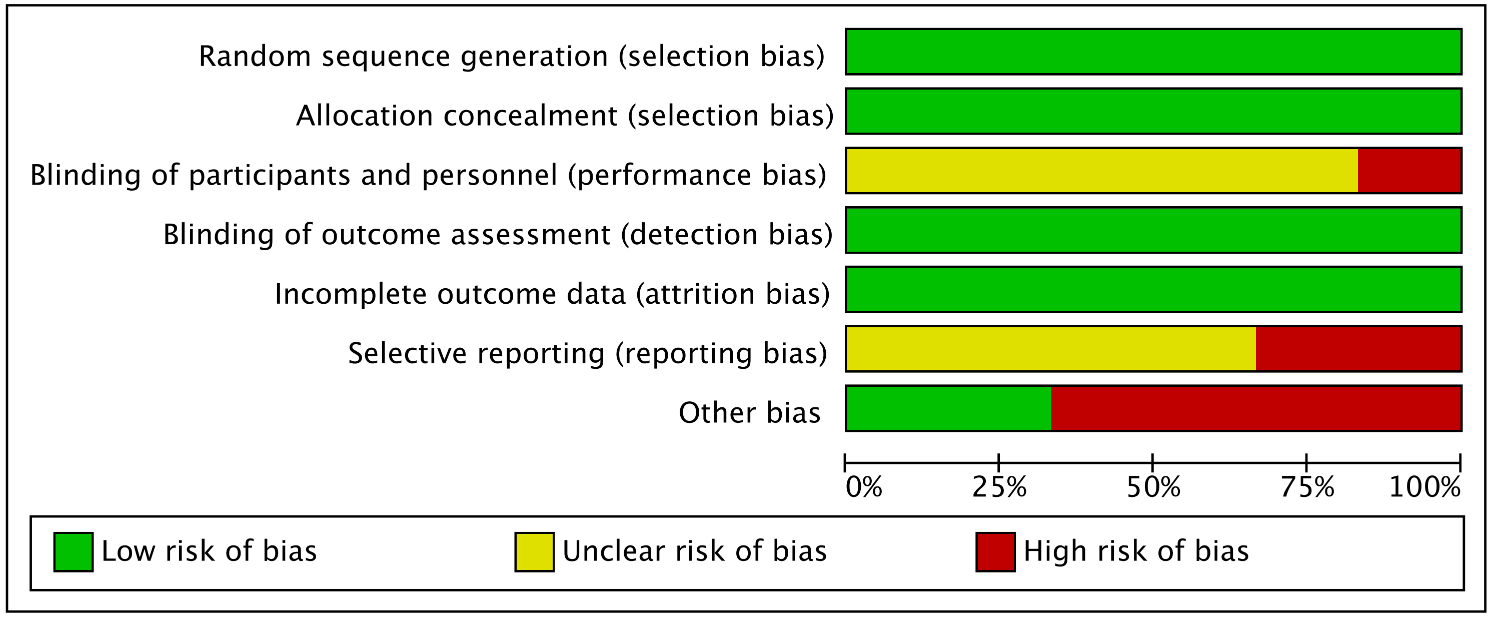

CQ07 Risk of bias summary, Risk of bias graph

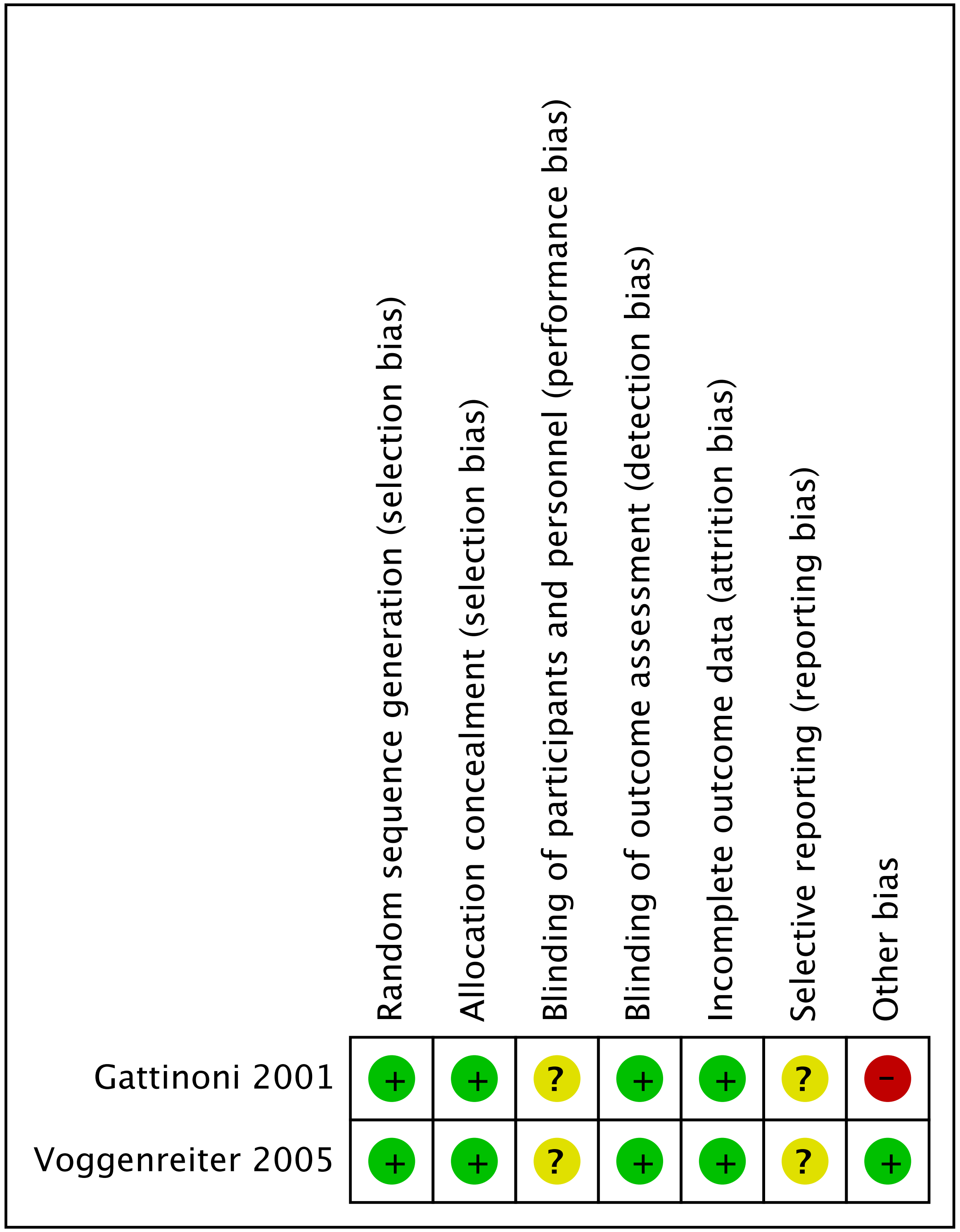

Decubitus

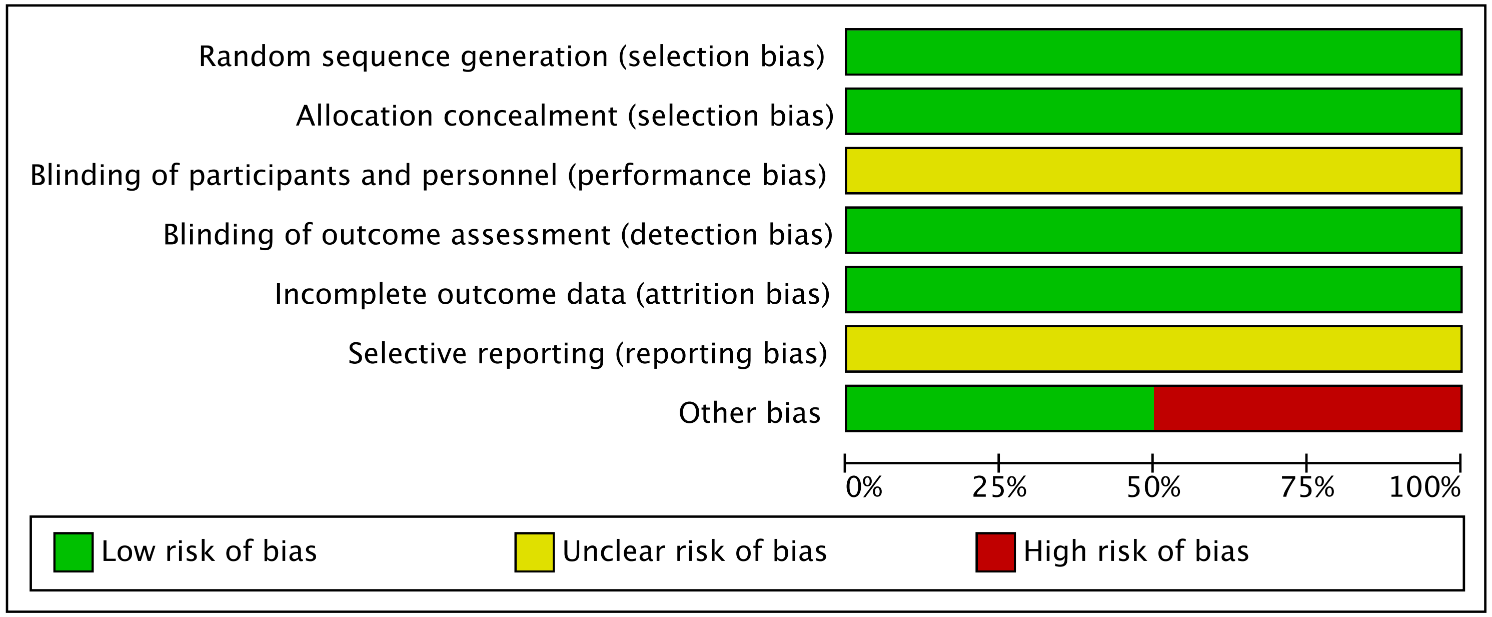

Short term mortality

Study or Subgroup	Prone Events	Prone Total	Supine Events	Supine Total	Weight	Risk Ratio M-H, Random, 95% CI
Beuret 2002	7	25	12	26	6.6%	0.61 [0.29, 1.29]
Fernandez 2008	8	21	10	19	7.4%	0.72 [0.36, 1.45]
Gattinoni 2001	32	152	38	152	13.8%	0.84 [0.56, 1.27]
Guerin 2004	134	413	119	378	21.4%	1.03 [0.84, 1.26]
Guerin 2013	38	237	75	229	16.0%	0.49 [0.35, 0.69]
Mancebo 2006	33	76	35	60	16.5%	0.74 [0.53, 1.04]
Taccone 2009	52	168	57	174	17.3%	0.94 [0.69, 1.29]
Voggenreiter 2005	1	21	3	19	1.0%	0.30 [0.03, 2.66]
**Total (95% CI)**		**1113**		**1057**	**100.0%**	**0.77 [0.62, 0.96]**
Total events	305		349			

Heterogeneity: $Tau^2 = 0.05$; $Chi^2 = 16.08$, df = 7 (P = 0.02); $I^2 = 56\%$
Z = 2.28 (P = 0.02)

Risk Ratio M-H, Random, 95% CI

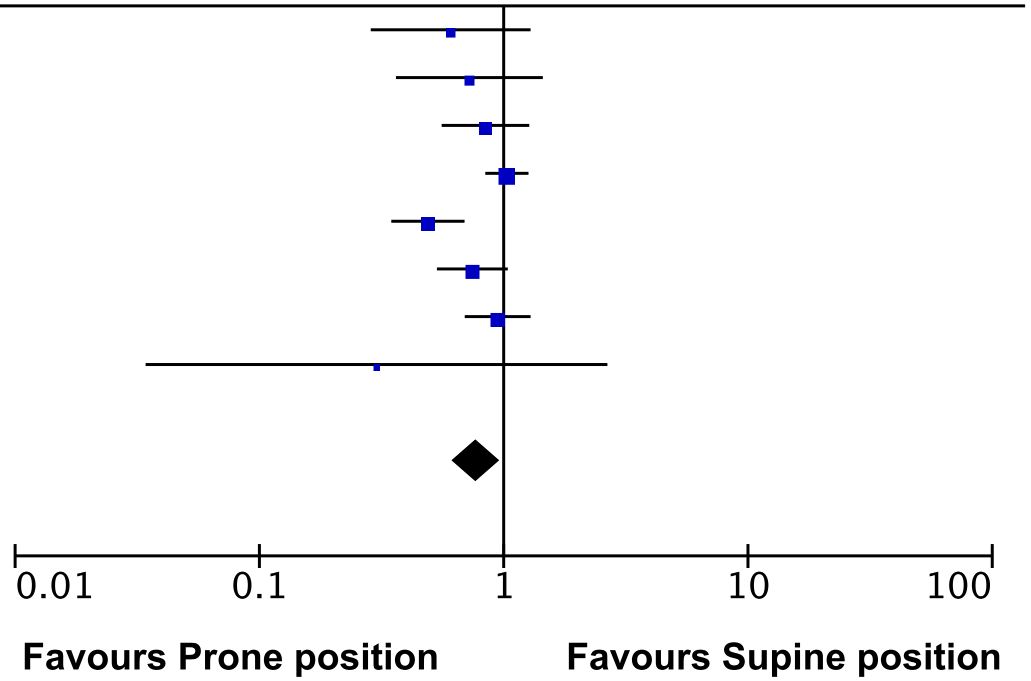

Complications on tracheal tube (accidental extubation, dislocation of tube)

Study or Subgroup	Prone Events	Prone Total	Supine Events	Supine Total	Weight	Risk Ratio M-H, Random, 95% CI
Fernandez 2008	1	21	1	19	2.1%	0.90 [0.06, 13.48]
Gattinoni 2001	12	152	15	152	25.2%	0.80 [0.39, 1.65]
Guerin 2013	31	237	25	229	46.2%	1.20 [0.73, 1.96]
Mancebo 2006	6	76	1	60	3.5%	4.74 [0.59, 38.29]
Taccone 2009	18	168	8	174	21.0%	2.33 [1.04, 5.21]
Voggenreiter 2005	1	21	1	19	2.1%	0.90 [0.06, 13.48]
**Total (95% CI)**		**675**		**653**	**100.0%**	**1.29 [0.87, 1.91]**
Total events	69		51			

Heterogeneity: $Tau^2 = 0.02$; $Chi^2 = 5.48$, df = 5 (P = 0.36); $I^2 = 9\%$
Test for overall effect: Z = 1.27 (P = 0.21)

Risk Ratio M-H, Random, 95% CI

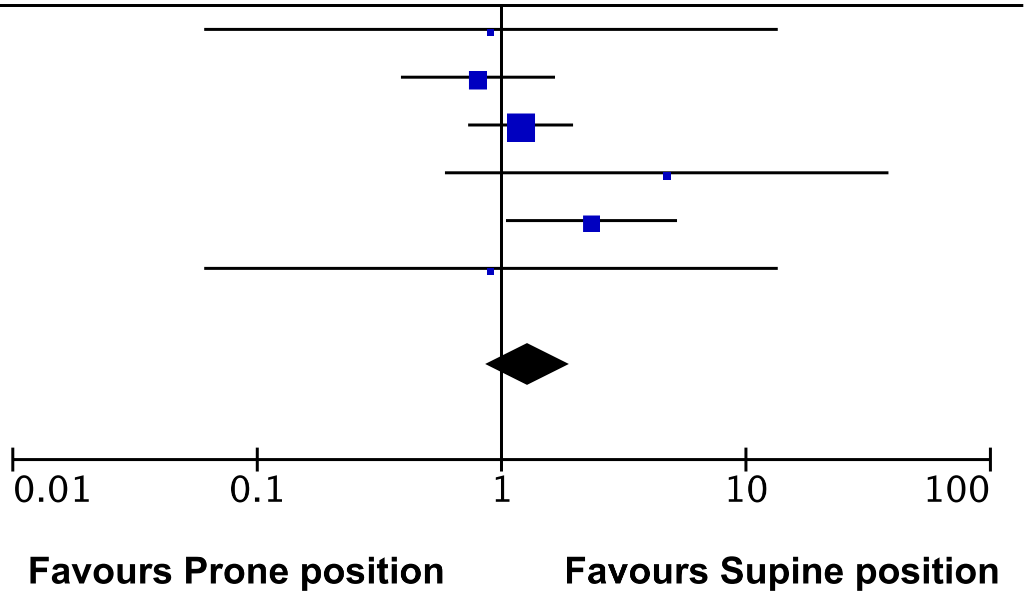

Decubitus

Study or Subgroup	Prone Events	Prone Total	Supine Events	Supine Total	Weight	Risk Ratio M-H, Random, 95% CI
Gattinoni 2001	55	152	42	152	55.3%	1.31 [0.94, 1.83]
Voggenreiter 2005	19	21	12	19	44.7%	1.43 [0.99, 2.07]
**Total (95% CI)**		**173**		**171**	**100.0%**	**1.36 [1.06, 1.75]**
Total events	74		54			

Heterogeneity: $Tau^2 = 0.00$; $Chi^2 = 0.15$, df = 1 (P = 0.70); $I^2 = 0\%$

Test for overall effect: Z = 2.45 (P = 0.01)

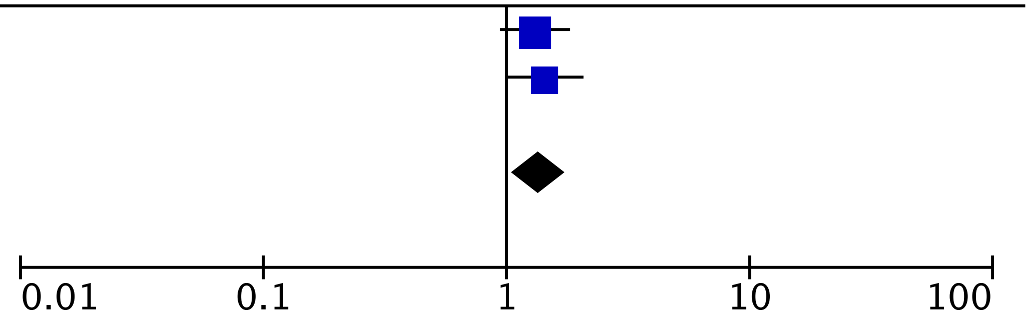

subgroup analysis, Short term mortality (moderate to severe ARDS)

Study or Subgroup	Prone Events	Prone Total	Supine Events	Supine Total	Weight	Risk Ratio M-H, Random, 95% CI
Fernandez 2008	8	21	10	19	13.8%	0.72 [0.36, 1.45]
Guerin 2013	38	237	75	229	27.9%	0.49 [0.35, 0.69]
Mancebo 2006	33	76	35	60	28.5%	0.74 [0.53, 1.04]
Taccone 2009	52	168	57	174	29.8%	0.94 [0.69, 1.29]
**Total (95% CI)**		**502**		**482**	**100.0%**	**0.71 [0.52, 0.97]**
Total events	131		177			

Heterogeneity: $Tau^2 = 0.06$; $Chi^2 = 7.86$, df = 3 (P = 0.05); $I^2 = 62\%$

Test for overall effect: Z = 2.16 (P = 0.03)

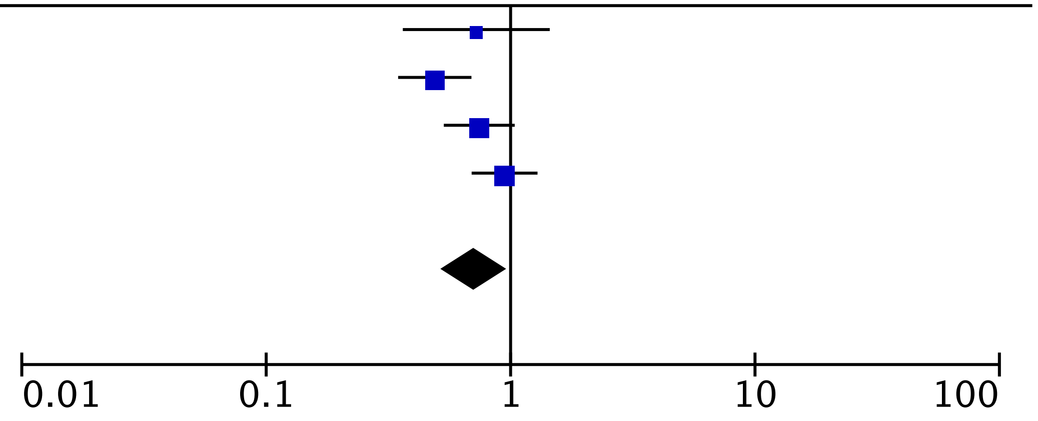

subgroup analysis, Short term mortality (prone position with long duration)

Study or Subgroup	Prone Events	Prone Total	Supine Events	Supine Total	Weight	Risk Ratio M-H, Random, 95% CI
Fernandez 2008	8	21	10	19	10.5%	0.72 [0.36, 1.45]
Guerin 2004	134	413	119	378	25.4%	1.03 [0.84, 1.26]
Guerin 2013	38	237	75	229	20.3%	0.49 [0.35, 0.69]
Mancebo 2006	33	76	35	60	20.7%	0.74 [0.53, 1.04]
Taccone 2009	52	168	57	174	21.6%	0.94 [0.69, 1.29]
Voggenreiter 2005	1	21	3	19	1.6%	0.30 [0.03, 2.66]
**Total (95% CI)**		**936**		**879**	**100.0%**	**0.77 [0.58, 1.02]**
Total events	266		299			

Heterogeneity: $Tau^2 = 0.07$; $Chi^2 = 15.39$, df = 5 (P = 0.009); $I^2 = 68\%$

Test for overall effect: Z = 1.84 (P = 0.07)

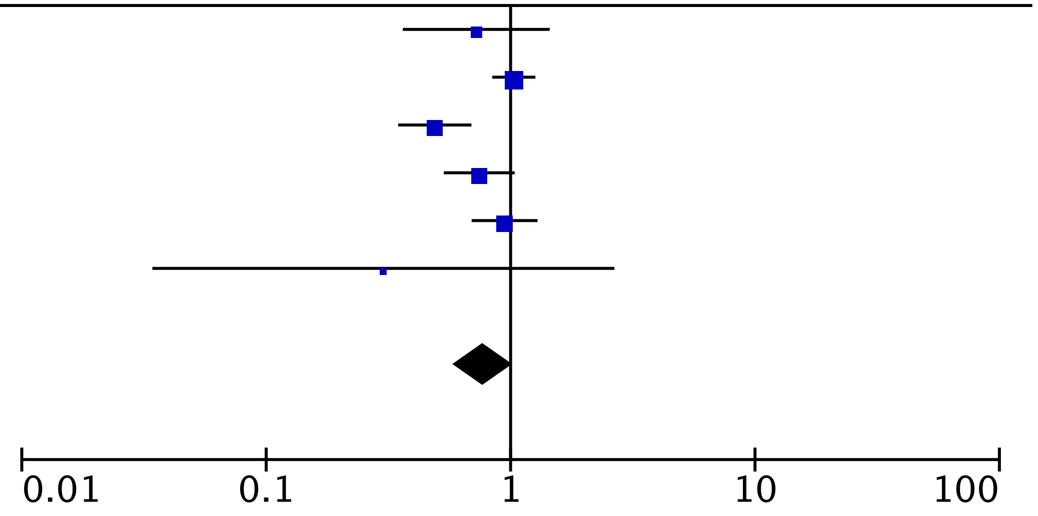

Summary of findings:

Prone positioning compared to supine positioning for adult ARDS

Patient or population: adult ARDS
Intervention: prone positioning
Comparison: supine positioning

Outcomes	Anticipated absolute effects* (95% CI)		Relative effect (95% CI)	№ of participants (studies)	Quality of the evidence (GRADE)	Comments
	Risk with supine positioning	Risk with prone positioning				
Short-term mortality	**Study population**		**RR 0.77** (0.62 to 0.96)	2170 (8 RCTs)	⊕⊕⊕◯ MODERATE [1]	
	330 / 1000	**254 / 1000** (205 ~ 317)				
	**Low**					
	250 / 1000	**193 / 1000** (155 ~ 240)				
	**High**					
	526 / 1000	**405 / 1000** (326 ~ 505)				
Short-term mortality (moderate/ severe)	**Study population**		**RR 0.71** (0.52 to 0.97)	984 (4 RCTs)	⊕⊕◯◯ LOW [2,3]	
	367 / 1000	**261 / 1000** (191 ~ 356)				
	**Low**					
	328 / 1000	**233 / 1000** (171 ~ 318)				
	**High**					
	526 / 1000	**373 / 1000** (274 ~ 510)				
Short-term mortality (prolonged prone)	**Study population**		**RR 0.77** (0.58 to 1.02)	1815 (6 RCTs)	⊕⊕◯◯ LOW [4,5]	
	340 / 1000	**262 / 1000** (197 ~ 347)				
	**Low**					
	315 / 1000	**243 / 1000** (183 ~ 321)				
	**High**					
	526 / 1000	**405 / 1000** (305 ~ 537)				
Tracheal tube trouble (unplanned extubation/ dislocation)	**Study population**		**RR 1.29** (0.87 to 1.91)	1328 (6 RCTs)	⊕⊕◯◯ LOW [6,7]	
	78 / 1000	**101 / 1000** (68 ~ 149)				
	**Low**					
	46 / 1000	**59 / 1000** (40 ~ 88)				
	**High**					
	99 / 1000	**128 / 1000** (86 ~ 189)				

Pressure ulcer	Study population		**RR 1.36** (1.06 to 1.75)	344 (2 RCTs)	⊕⊕⊕◯ MODERATE [3]
	316 / 1000	**429 / 1000** (335 ～ 553)			
	Low				
	276 / 1000	**375 / 1000** (293 ～ 483)			
	High				
	632 / 1000	**860 / 1000** (670 ～ 1000)			

***The risk in the intervention group** (and its 95% confidence interval) is based on the assumed risk in the comparison group and the **relative effect** of the intervention (and its 95% CI).

CI: Confidence interval; **RR:** Risk ratio;

GRADE Working Group grades of evidence
High quality: We are very confident that the true effect lies close to that of the estimate of the effect
Moderate quality: We are moderately confident in the effect estimate: The true effect is likely to be close to the estimate of the effect, but there is a possibility that it is substantially different
Low quality: Our confidence in the effect estimate is limited: The true effect may be substantially different from the estimate of the effect
Very low quality: We have very little confidence in the effect estimate: The true effect is likely to be substantially different from the estimate of effect

1. There is moderate heterogeneity with I2=56%.
2. There is substantial heterogeneity with I2=62%.
3. Sample size is small.
4. There is substantial heterogeneity with I2=68%.
5. Sample size is small and 95%CI crosses clinical decision threshold.
6. This outcome was not prearranged measurement item in two RCTs that have large weights.
7. Sample size is small and 95%CI is wide and crosses clinical decision threshold.

Table 1G. Evidence profile
CQ7 : Prone positioning compared with supine positioning for adult patients with ARDS

Quality assessment							№ of patients		Effect		Quality	Importance
№ of studies	Study design	Risk of bias	Inconsistency	Indirectness	Imprecision	Other considerations	Prone	Supine	Relative (95% CI)	Absolute (95% CI)		
Short-term mortality												
8	Randomized trials	Not serious	Serious [1]	Not serious	Not serious	None	305/1113 (27.4%)	349/1057 (33.0%)	**RR 0.77** (0.62 to 0.96)	76 fewer per 1000 (from 13 fewer to 125 fewer)	⊕⊕⊕◯ MODERATE [1]	CRITICAL
								25.0%		57 fewer per 1000 (from 10 fewer to 95 fewer)		
								52.6%		121 fewer per 1000 (from 21 fewer to 200 fewer)		
Short-term mortality (moderate/severe)												
4	Randomized trials	Not serious	Serious [2]	Not serious	Serious [3]	None	131/502 (26.1%)	177/482 (36.7%)	**RR 0.71** (0.52 to 0.97)	106 fewer per 1000 (from 11 fewer to 176 fewer)	⊕⊕◯◯ LOW [2 3]	CRITICAL
								32.8%		95 fewer per 1000 (from 10 fewer to 157 fewer)		
								52.6%		153 fewer per 1000 (from 16 fewer to 252 fewer)		
Short-term mortality (prolonged prone)												
6	Randomized trials	Not serious	Serious [4]	Not serious	Serious [5]	None	266/936 (28.4%)	299/879 (34.0%)	**RR 0.77** (0.58 to 1.02)	78 fewer per 1000 (from 7 more to 143 fewer)	⊕⊕◯◯ LOW [4 5]	CRITICAL
								31.5%		72 fewer per 1000 (from 6 more to 132 fewer)		
								52.6%		121 fewer per 1000 (from 11 more to 221 fewer)		
Tracheal tube trouble (unplanned extubation/dislocateion)												
6	Randomized trials	Serious [6]	Not serious	Not serious	Serious [7]	None	69/675 (10.2%)	51/653 (7.8%)	**RR 1.29** (0.87 to 1.91)	23 more per 1000 (from 10 fewer to 71 more)	⊕⊕◯◯ LOW [6 7]	CRITICAL
								4.6%		13 more per 1000 (from 6 fewer to 42 more)		
								9.9%		29 more per 1000 (from 13 fewer to 90 more)		

Pressure ulcer												
2	Randomized trials	Not serious	Not serious	Not serious	Serious[3]	None	74/173 (42.8%)	54/171 (31.6%)	**RR 1.36** (1.06 to 1.75)	114 more per 1000 (from 19 more to 237 more)	⨁⨁⨁◯ MODERATE[3]	IMPORTANT
								27.6%		99 more per 1000 (from 17 more to 207 more)		
								63.2%		228 more per 1000 (from 38 more to 474 more)		

1 There is moderate heterogeneity with I2=56%.
2 There is substantial heterogeneity with I2=62%.
3 Sample size is small.
4 There is substantial heterogeneity with I2=68%.
5 Sample size is small and 95%CI crosses the clinical decision threshold.
6 This outcome was not a predetermined measurement item in two RCTs with large weights.
7 Sample size is small and 95%CI is wide and crosses the clinical decision threshol

Evidence-to-Decision table

CQ7：Should prone positioning be performed in adult patients with ARDS?

PATIENTS：ADULT ARDS

NTERVENTION: PRONE POSITION MANAGEMENT

	CRITERIA	JUDGEMENTS	RESEARCH EVIDENCE	ADDITIONAL CONSIDERATIONS

PROBLEM

Is there a problem priority?

○No
○Probably no
●Probably yes
○Yes

○Varies
○Don't know

Prone positioning is expected to be effective for ARDS because of pathophysiological improvement in respiratory mechanics, oxygenation, and hemodynamics or prevention of VILI.[1, 2)] Although many RCTs and their meta-analyses on prone positioning for ARDS have been conducted, the results are not consistent, and therefore, the effects of prone positioning for ARDS are controversial. [3-7)] As prone positioning can be performed without specialized equipment, to examine the effectiveness of prone positioning for ARDS is considered a high priority.

BENEFITS & HARMS OF THE OPTIONS

What is the overall certainty of the evidence of effects?

○Very low
●Low
○Moderate
○High

○No included studies

Is there important uncertainty about or variability in how much people value the main outcomes?

○Important uncertainty or variability
●Possibly important uncertainty or variability
○Possibly no important uncertainty or variability
○No important uncertainty or variability

○No known undesirable outcomes

How substantial are the desirable anticipated effects?

○Trivial
○Small
●Moderate
○Large

○Varies
○Don't know

How substantial are the undesirable anticipated effects?

○Large
○Moderate
○Small
○Trivial

●Varies
○Don't know

Does the balance between desirable effects and undesirable effects favour the option or the comparison?

○Favors the comparison
○Probably favors the comparison
○Does not favor either the intervention or the comparison
●Probably favors the

The relative importance or values of the main outcomes of interest:

Outcome	Relative importance	Certainty of the evidence (GRADE)
Short-term mortality[(1]	CRITICAL	⨁⨁⨁◯ MODERATE
Short-term mortality (moderate/ severe)[(2]	CRITICAL	⨁⨁◯◯ LOW
Short-term mortality (prolonged prone)[(3]	CRITICAL	⨁⨁◯◯ LOW
Tracheal tube trouble (unplanned extubation/ dislocation)	CRITICAL	⨁⨁◯◯ LOW
Pressure ulcer	IMPORTANT	⨁⨁⨁◯ MODERATE

Summary of findings:

Outcome	Supine position	Prone position	Absolute effect (95% CI)	Relative effect (RR) (95% CI)
Short-term mortality[(1]	330 / 1000	254 / 1000 (205 ～ 317)	76 fewer per 1000 (from 13 fewer to 125 fewer)	RR 0.77 (0.62 ～ 0.96)
	250 / 1000	193 / 1000 (155 ～ 240)	57 fewer per 1000 (from 10 fewer to 95 fewer)	
	526 / 1000	405 / 1000 (326 ～ 505)	121 fewer per 1000 (from 21 fewer to 200 fewer)	
Short-term mortality (moderate/ severe)[(2]	367 / 1000	261 / 1000 (191 ～ 356)	106 fewer per 1000 (from 11 fewer to 176 fewer)	RR 0.71 (0.52 ～ 0.97)
	328 / 1000	233 / 1000 (171 ～ 318)	95 fewer per 1000 (from 10 fewer to 157 fewer)	
	526 / 1000	373 / 1000 (274 ～ 510)	153 fewer per 1000 (from 16 fewer to 252 fewer)	
Short-term mortality (prolonged prone)[(3]	340 / 1000	262 / 1000 (197 ～ 347)	78 fewer per 1000 (from 7 more to 143 fewer)	RR 0.77 (0.58 ～ 1.02)
	315 / 1000	243 / 1000 (183 ～ 321)	72 fewer per 1000 (from 6 more to 132 fewer)	
	526 / 1000	405 / 1000 (305 ～ 537)	121 fewer per 1000 (from 11 more to 221 fewer)	
Tracheal tube trouble (unplanned extubation/ dislocation)	78 / 1000	101 / 1000 (68 ～ 149)	23 more per 1000 (from 10 fewer to 71 more)	RR 1.29 (0.87 ～ 1.91)
	46 / 1000	59 / 1000 (40 ～ 88)	13 more per 1000 (from 6 fewer to 42 more)	
	99 / 1000	128 / 1000 (86 ～ 189)	29 more per 1000 (from 13 fewer to 90	

		intervention<br>○Favors the intervention<br>------------------<br>○Varies<br>○Don't know	(see table and summary below)	

			more)	
Pressure ulcer	316 / 1000	429 / 1000 (335 ～ 553)	114 more per 1000 (from 19 more to 237 more)	RR 1.36 (1.06 ～ 1.75)
	276 / 1000	375 / 1000 (293 ～ 483)	99 more per 1000 (from 17 more to 207 more)	
	632 / 1000	860 / 1000 (670 ～ 1000)	228 more per 1000 (from 38 more to 474 more)	

Summary: Prone positioning significantly reduced the mortality (RR 0.77, 95%CI 0.62～0.96). In a subanalysis of 4 RCTs which addressed moderate and severe ARDS (P/F ≤ 200), the mortality was significantly reduced (RR 0.71, 95%CI 0.52～0.97). In a subanalysis of 6 RCTs which addressed prolonged prone positioning (≥ 8 hours), although similar tendency was shown, there was no significant difference between prone and supine (RR 0.77, 95%CI 0.58～1.02). In addition, although prone positioning did not increase serious adverse events such as tracheal tube trouble (RR 1.29, 95%CI 0.87～1.91), it significantly increased pressure ulcer (RR 1.36, 95%CI 1.06～1.75).

資源利用	**How large are the resource requirements (costs)?**	○Large costs<br>●Moderate costs<br>○Negligible costs and savings<br>○Moderate savings<br>○Large savings<br>------------------<br>○Varies<br>○Don't know	Changing position to prone requires more manpower than usual. Although there is a specialized bed that can reduce burden in manpower (e.g. RotoProne bed[R]), it takes a high cost and is not approved in Japan. In addition, prone positioning requires more careful monitoring than usual. However, even if considering the burden in manpower and cost, prone position has significant effects to reduce mortality without increase in severe adverse effects. Therefore, the benefit of prone positioning is greater than the burden in cost or resources.	
	**Does the cost effectiveness of the option favour the option or the comparison?**	○Favors the comparison<br>○Probably favors the comparison<br>○Does not favor either the intervention or the comparison<br>●Probably favors the intervention<br>○Favors the intervention<br>------------------<br>○Varies<br>○No included studies	Compared to the benefit of reducing mortality, increases in cost and manpower are within an allowance.	
EQUITY	**What would be the impact on health equity?**	○Reduced<br>○Probably reduced<br>○Probably no impact<br>●Probably increased<br>○Increased<br>------------------<br>○Varies<br>○Don't know	Prone position can be performed at standard hospitals, especially at facilities that can provide intensive care for ARDS. However, the safety may differ among hospitals depending on staff resources. In addition, the effectiveness may vary according to patient's physical constitution or underlying disease.	
ACCEPTABILITY	**Is the option acceptable to key stakeholders?**	○No<br>○Probably no<br>●Probably yes<br>○Yes<br>------------------<br>○Varies<br>○Don't know	Prone position itself is one of the common positions that everyone can take. However, someone may refuse it when he/she is forced to take prone position for a long time for therapeutic purposes.	

**FEASIBILITY**	**Is the option feasible to implement?**	○No<br>○Probably no<br>●Probably yes<br>○Yes<br>------------------<br>○Varies<br>○Don't know	Prone position can be performed if plural staff can be secured while changing position and after positioning for monitoring.	

Recommendation

CQ7 : Should prone positioning be performed in adult patients with ARDS?					
**Balance of consequences**	**Undesirable consequences *clearly outweigh* desirable consequences in most settings**	**Undesirable consequences *probably outweigh* desirable consequences in most settings**	**The balance between desirable and undesirable consequences is closely *balanced or uncertain***	**Desirable consequences *probably outweigh* undesirable consequences in most settings**	**Desirable consequences *clearly outweigh* undesirable consequences in most settings**
Judgement	○	○	○	●	○

**Type of recommendation**	**We recommend against offering this option**	**We suggest not offering this option**	**We suggest offering this option**	**We recommend offering this option**
Judgement	○	○	●	○
**Recommendation**	**We suggest prone positioning in adult patients with ARDS (especially in moderate and severe cases). (GRADE 2C, Strength of recommendation "weak recommendation" / Quality of evidence "low")**			
**Justification**	**Question:** Should prone positioning be performed in adult patients with ARDS?<br>**Patients:** Adult ARDS<br>**Interventions:** Prone positioning<br>**Comparison:** Supine positioning<br>**Outcomes:** Mortality, Adverse effects (Tracheal tube trouble, Pressure ulcer)<br>**Summary of the evidence:** We conducted a systematic review of RCTs on prone positioning for adult ARDS. In a meta-analysis of 8 RCTs, prone positioning significantly reduced the mortality (RR 0.77, 95%CI 0.62～0.96). In a subanalysis of 4 RCTs which addressed moderate and severe ARDS (P/F ≤ 200), the mortality was significantly reduced (RR 0.71, 95%CI 0.52～0.97). In a subanalysis of 6 RCTs which addressed prolonged prone positioning (≥ 8 hours), although similar tendency was shown, there was no significant difference between prone and supine (RR 0.77, 95%CI 0.58～1.02). In addition, although prone positioning did not increase serious adverse events such as tracheal tube trouble (RR 1.29, 95%CI 0.87～1.91), it significantly increased pressure ulcer (RR 1.36, 95%CI 1.06～1.75).<br>**Quality of the evidence:** All studies included in the meta-analysis were RCTs. RCTs examining mortality generally had low risk of bias but had inconsistency. There were no serious indirectness or imprecision. Publication bias could not be assessed because of small number of studies. The certainty of the evidence of effects of prone positioning for adult ARDS on mortality was evaluated as "moderate". In the subanalysis focusing on moderate and severe ARDS cases, sample size was small. In the subanalysis focusing on prolonged prone positioning, confidence interval crossed clinical decision threshold. Thus, the certainty of the evidence for both of these two subanalyses was evaluated as "low" because of imprecision.<br>As the meta-analysis addressing tracheal tube trouble included RCTs whose risk of bias was not low and showed imprecision, the certainty of the evidence was evaluated as "low". The meta-analysis addressing pressure ulcer included RCTs whose risk of bias was low and did not show serious inconsistency or indirectness. However, because of the small number of studies and imprecision, the certainty of the evidence was evaluated as "moderate".<br>Thus, the overall certainty of the evidence was evaluated as "low".<br>**Judgement of benefit and harm, resources and cost:** As prone positioning reduces mortality without significant increases of serious adverse events, benefit is greater than harm, resources and cost. However, it should be noted that the facilities which participated in the RCT were well experienced. During implementation, you should be careful of troubles with tubes and lines and occurrence of pressure ulcer.<br><br>**Recommendations: We suggest prone positioning in adult patients with ARDS (especially in moderate and severe cases). (GRADE 2C, Strength of recommendation "weak recommendation" / Quality of evidence "low")**			

	**Additional considerations:** Among panelists, there was an opinion that "strong recommendation" is more preferable with emphasis on the effects of reduction of mortality. However, the certainty of the evidence is low and prone positioning requires experience. Additionally, implementation rate differs greatly between facilities. Thus, panel meeting decided prone positioning for adult ARDS as "weak recommendation". As a supplemental explanation, this recommendation does not mean that prone positioning should be restricted to a certain facility that is well experienced in prone position management. Rather, organizing a system, including securing manpower and educating staff, for providing prone position management anywhere is important.
**Subgroup considerations**	In subanalyses, although the estimate of effect of prolonged prone positioning (≥ 8 hours) was similar, there was no significant difference. On the other hand, the effect of prone positioning for moderate and severe ARDS (P/F ≤ 200) was expected to be greater.
**Implementation considerations**	Plural practiced personnel are required when performing prone positioning. The effect of prone positioning may be insufficient if it is performed in a short time only when enough personnel can be secured. Knowing actual status of one's own facility is required.
**Monitoring and evaluation considerations**	Increase of blood pressure and heart rate due to stimulus, decrease of blood pressure due to fluid shift, arrhythmia, change in tidal volume or airway pressure due to decrease of lung-thorax compliance, obstruction, malposition, or unplanned extubation of tracheal tube, aspiration of oral secretions, flexion or unplanned removal of important tube and line, compression injury of eyeball or external genitals, pressure ulcer, peripheral neuropathy, vascular insufficiency of skin.
**Research possibilities**	Investigation for long-term mortality and functional prognosis as well as short-term mortality is required. In addition, study on optimal subject (severity) or optimal methods (i.e., duration or repetition) of prone positioning is required.

1) Short-term mortality was defined as 10-day, 28-day, 60-day, 90-day, or ICU mortality

2) Moderate and severe ARDS was defined as $P/F \leq 200$

3) Prolonged prone positioning was defined as prone positioning ≥ 8 hours per day

Reference

1. Guerin C, Baboi L, Richard JC. Mechanisms of the effects of prone positioning in acute respiratory distress syndrome. *Intensive Care Med* **40**(11): 1634-42, 2014. PMID 25266133
2. Gattinoni L, Taccone P, Carlesso E, et al. Prone position in acute respiratory distress syndrome. Rationale, indications, and limits. *Am J Respir Crit Care Med* **188**(11): 1286-93, 2013. PMID 24134414
3. Bloomfield R, Noble DW, Sudlow A. Prone position for acute respiratory failure in adults. *Cochrane Database Syst Rev* **11**: CD008095, 2015. PMID 26561745
4. Sud S, Friedrich JO, Adhikari NK, et al. Effect of prone positioning during mechanical ventilation on mortality among patients with acute respiratory distress syndrome: a systematic review and meta-analysis. *CMAJ* **186**(10): E381-90, 2014. PMID 24863923

5. Beitler JR, Shaefi S, Montesi SB, et al. Prone positioning reduces mortality from acute respiratory distress syndrome in the low tidal volume era: a meta-analysis. *Intensive Care Med* **40**(3): 332-41, 2014. PMID 24435203
6. Lee JM, Bae W, Lee YJ, et al. The efficacy and safety of prone positional ventilation in acute respiratory distress syndrome: updated study-level meta-analysis of 11 randomized controlled trials. *Crit Care Med* **42**(5): 1252-62, 2014. PMID 24368348
7. Hu SL, He HL, Pan C, et al. The effect of prone positioning on mortality in patients with acute respiratory distress syndrome: a meta-analysis of randomized controlled trials. *Crit Care* **18**(3): R109, 2014. PMID 24887034

CQ08. Study flow diagram

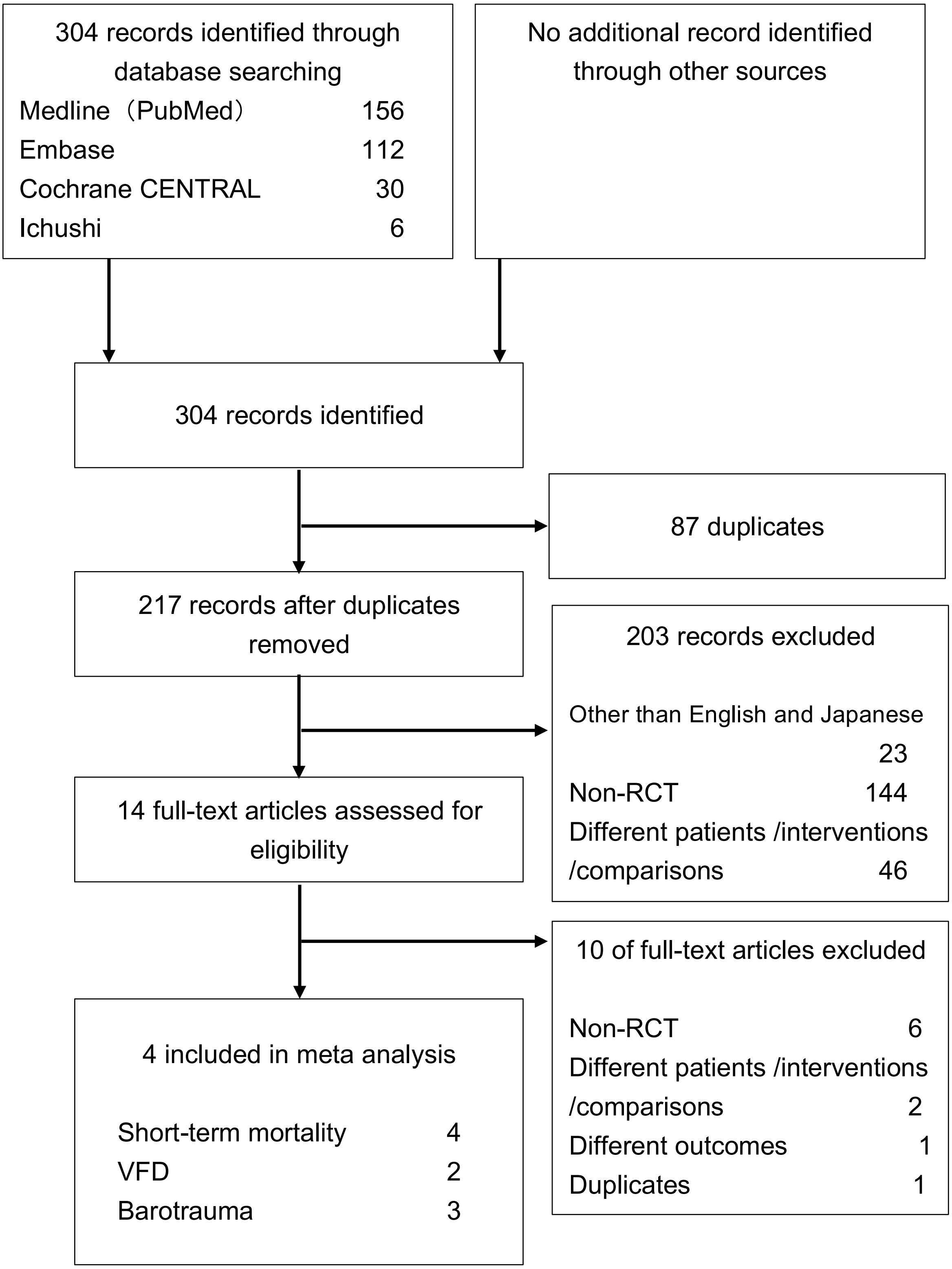

CQ08
Risk of bias table, mortality

Outcome		Short term mortality		risk of bias		serious (−1)			
番号	著者名 発表年（Forest plot表示）	risk of bias評価							
		ランダム割付順番の生成 random sequence generation	割り付けの隠蔽化 allocation concealment	ブラインド blinding 研究参加者と治療提供者 participants and personnel	ブラインド blinding アウトカム評価者 outcome assessors	不完全なアウトカムデータ incomplete outcome data	選択されたアウトカムの報告 selective outcome reporting	その他のバイアス Other sources of bias	研究内でのバイアスのリスク Risk of bias within a study
1	Derdak 2002	Low risk	Unclear risk	High risk	Unclear risk	Low risk	Low risk	High risk	High risk
2	Bollen 2005	Low risk	Unclear risk	High risk	High risk	Low risk	Low risk	High risk	High risk
3	Young 2013	Low risk	Unclear risk	High risk	Unclear risk	Low risk	Low risk	High risk	High risk
4	Fergunson 2013	Low risk	Low risk	High risk	High risk	Low risk	Low risk	High risk	High risk
番号	著者名 発表年（Forest plot表示）	risk of biasコメント							
1	Derdak 2002	コンピュータで作成された乱数を使用	記載が見当たらない	人工呼吸器のモードの比較でありブラインド化はできない	記載が見当たらない	100%フォローされた	100%報告された	クロスオーバーしている	high riskが多い
2	Bollen 2005	コンピュータで作成された乱数を使用	記載が見当たらない	人工呼吸器のモードの比較でありブラインド化はできない	ブラインド化されていない	100%フォローされた	100%報告された	患者選択のダイアグラムが提示されていないのでセレクションバイアスについては判断出来ない。その他バイアスについての情報が少なく判断出来ない。	high riskが多い
3	Young 2013	コンピュータで作成された乱数を使用	記載が見当たらない	人工呼吸器のモードの比較でありブラインド化はできない	記載が見当たらない	100%フォローされた	100%報告された	Eligibleな患者のうち半分以下しかランダマイゼーションに参加していない。試験に参加しなかった場合は従来のモードで管理されることが補償されており、セレクションバイアスが危惧される。	high riskが多い
4	Fergunson 2013	コンピュータで作成された乱数を使用	研究者たちは全員，割り付けやブロックサイズを知らされていなかった.	人工呼吸器のモードの比較でありブラインド化はできない	ブラインド化されていない	100%フォローされた	100%報告された	Eligibleな患者のうち半分以下しかランダマイゼーションに参加していない。試験に参加しなかった場合は従来のモードで管理されることが補償されており、セレクションバイアスが危惧される。	high riskが多い

CQ08
Risk of bias table VFD

Outcome		VFD		risk of bias		serious (−1)			
番号	著者名 発表年（Forest plot表示）	risk of bias評価							
		ランダム割付順番の生成 random sequence generation	割り付けの隠蔽化 allocation concealment	ブラインド blinding		不完全なアウトカムデータ incomplete outcome data	選択されたアウトカムの報告 selective outcome reporting	その他のバイアス Other sources of bias	研究内でのバイアスのリスク Risk of bias within a study
				研究参加者と治療提供者 participants and personnel	アウトカム評価者 outcome assessors				
1	Young 2013	Low risk	Unclear risk	High risk	Unclear risk	Low risk	Low risk	High risk	High risk
2	Fergunson 2013	Low risk	Low risk	High risk	High risk	Low risk	Low risk	High risk	High risk
番号	著者名 発表年（Forest plot表示）	risk of biasコメント							
1	Young 2013	コンピュータで作成された乱数を使用	記載が見当たらない	人工呼吸器のモードの比較でありブラインド化はできない	記載が見当たらない	100%フォローされた	100%報告された	Eligibleな患者のうち半分以下しかランダマイゼーションに参加していない。試験に参加しなかった場合は従来のモードで管理されることが補償されており、セレクションバイアスが危惧される。	high riskが多い
2	Fergunson 2013	コンピュータで作成された乱数を使用	研究者たちは全員，割り付けやブロックサイズを知らされていなかった.	人工呼吸器のモードの比較でありブラインド化はできない	ブラインド化されていない	100%フォローされた	100%報告された	Eligibleな患者のうち半分以下しかランダマイゼーションに参加していない。試験に参加しなかった場合は従来のモードで管理されることが補償されており、セレクションバイアスが危惧される。	high riskが多い

CQ08
Risk of bias table, barotrauma

Outcome		Barotrauma		risk of bias		serious (−1)			
番号	著者名 発表年<br>（Forest plot表示）	risk of bias評価							
		ランダム割付順番の生成<br>random sequence generation	割り付けの隠蔽化<br>allocation concealment	ブラインド<br>blinding<br>研究参加者と治療提供者<br>participants and personnel	ブラインド<br>blinding<br>アウトカム評価者<br>outcome assessors	不完全なアウトカムデータ<br>incomplete outcome data	選択されたアウトカムの報告<br>selective outcome reporting	その他のバイアス<br>Other sources of bias	研究内でのバイアスのリスク<br>Risk of bias within a study
1	Derdak 2002	Low risk	Unclear risk	High risk	Unclear risk	Low risk	Low risk	High risk	High risk
2	Bollen 2005	Low risk	Unclear risk	High risk	High risk	Low risk	Low risk	High risk	High risk
3	Fergunson 2013	Low risk	Low risk	High risk	High risk	Low risk	Low risk	High risk	High risk
番号	著者名 発表年<br>（Forest plot表示）	risk of biasコメント							
1	Derdak 2002	コンピュータで作成された乱数を使用	記載が見当たらない	人工呼吸器のモードの比較でありブラインド化はできない	記載が見当たらない	100%フォローされた	100%報告された	クロスオーバーしている	high riskが多い
2	Bollen 2005	コンピュータで作成された乱数を使用	記載が見当たらない	人工呼吸器のモードの比較でありブラインド化はできない	ブラインド化されていない	100%フォローされた	100%報告された	患者選択のダイアグラムが提示されていないのでセレクションバイアスについては判断出来ない。その他バイアスについての情報が少なく判断出来ない。	high riskが多い
3	Fergunson 2013	コンピュータで作成された乱数を使用	研究者たちは全員，割り付けやブロックサイズを知らされていなかった.	人工呼吸器のモードの比較でありブラインド化はできない	ブラインド化されていない	100%フォローされた	100%報告された	Eligibleな患者のうち半分以下しかランダマイゼーションに参加していない。試験に参加しなかった場合は従来のモードで管理されることが補償されており、セレクションバイアスが危惧される。	high riskが多い

CQ08 Risk of bias summary, Risk of bias graph

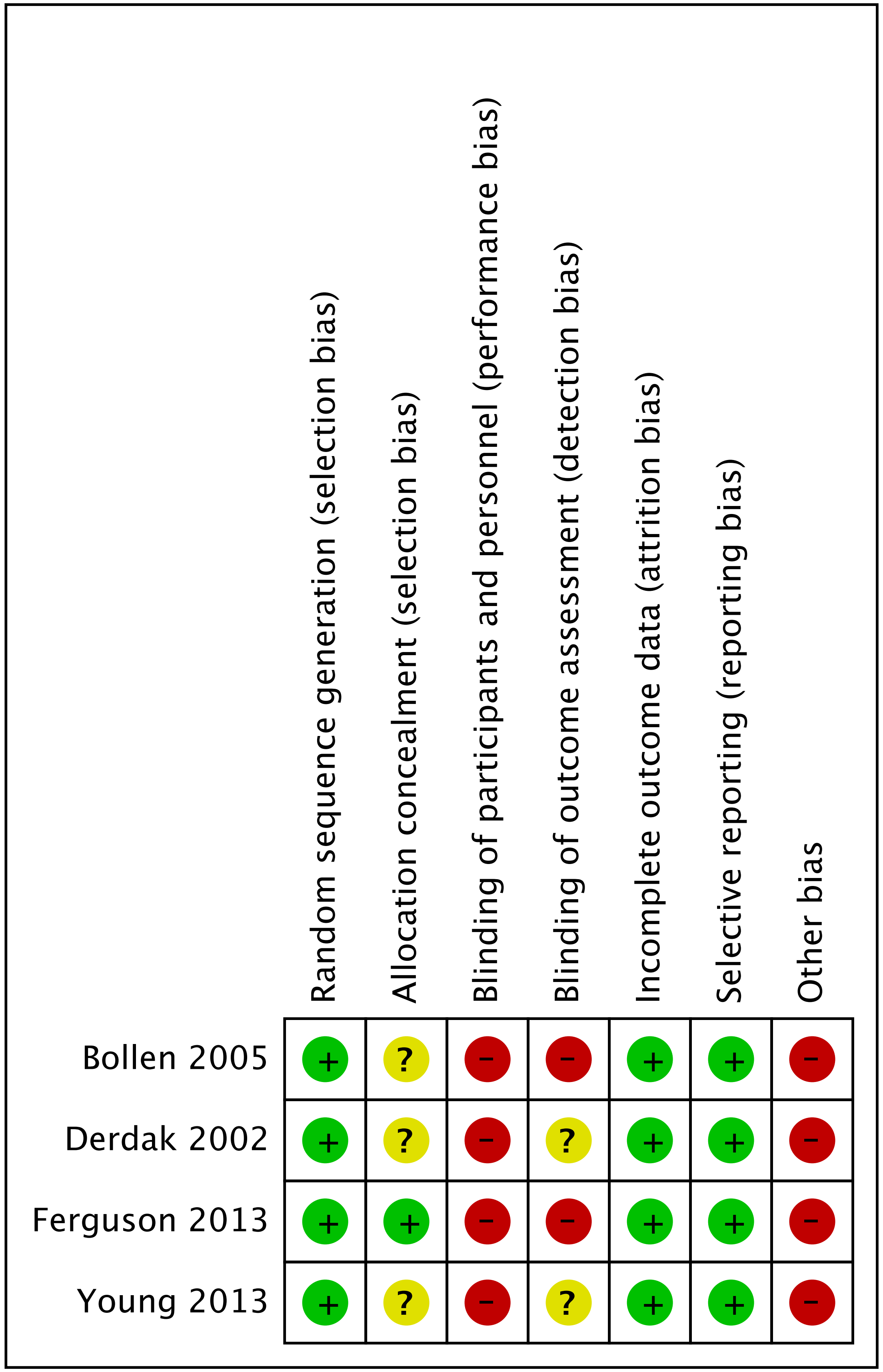

Short term mortality

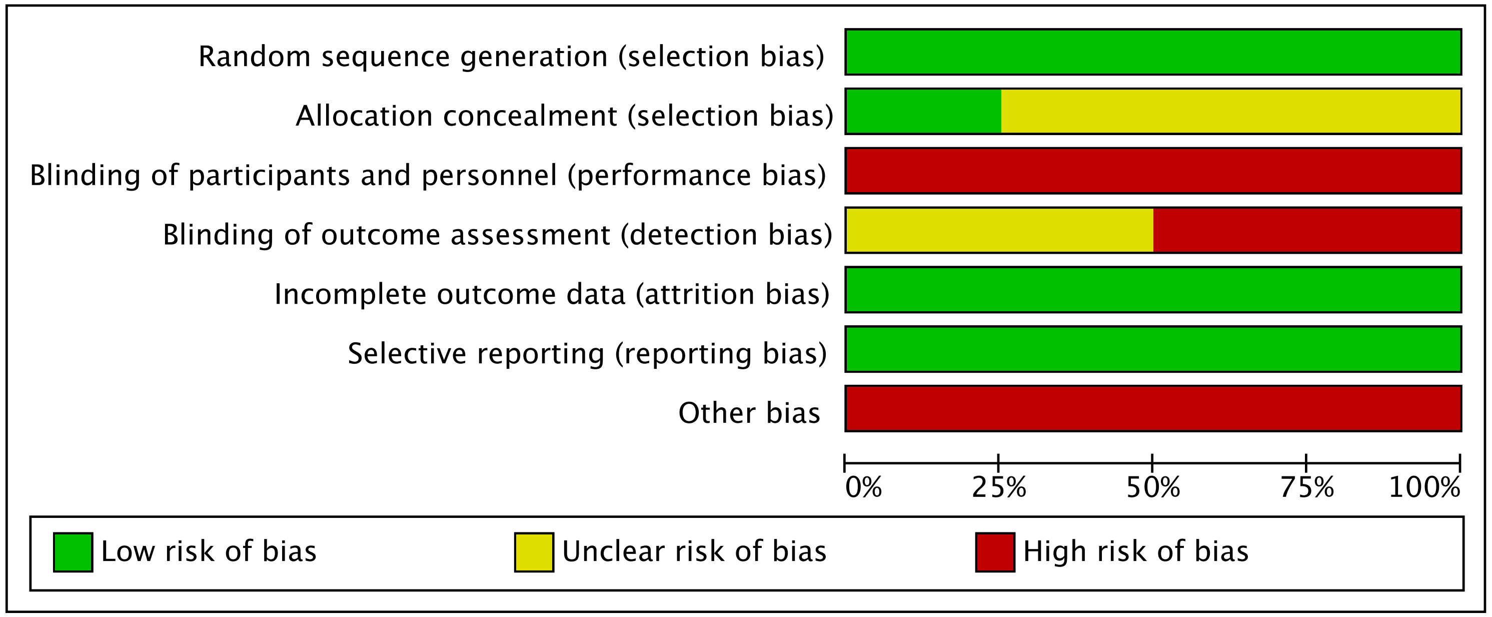

CQ08 Risk of bias summary, Risk of bias graph

VFD

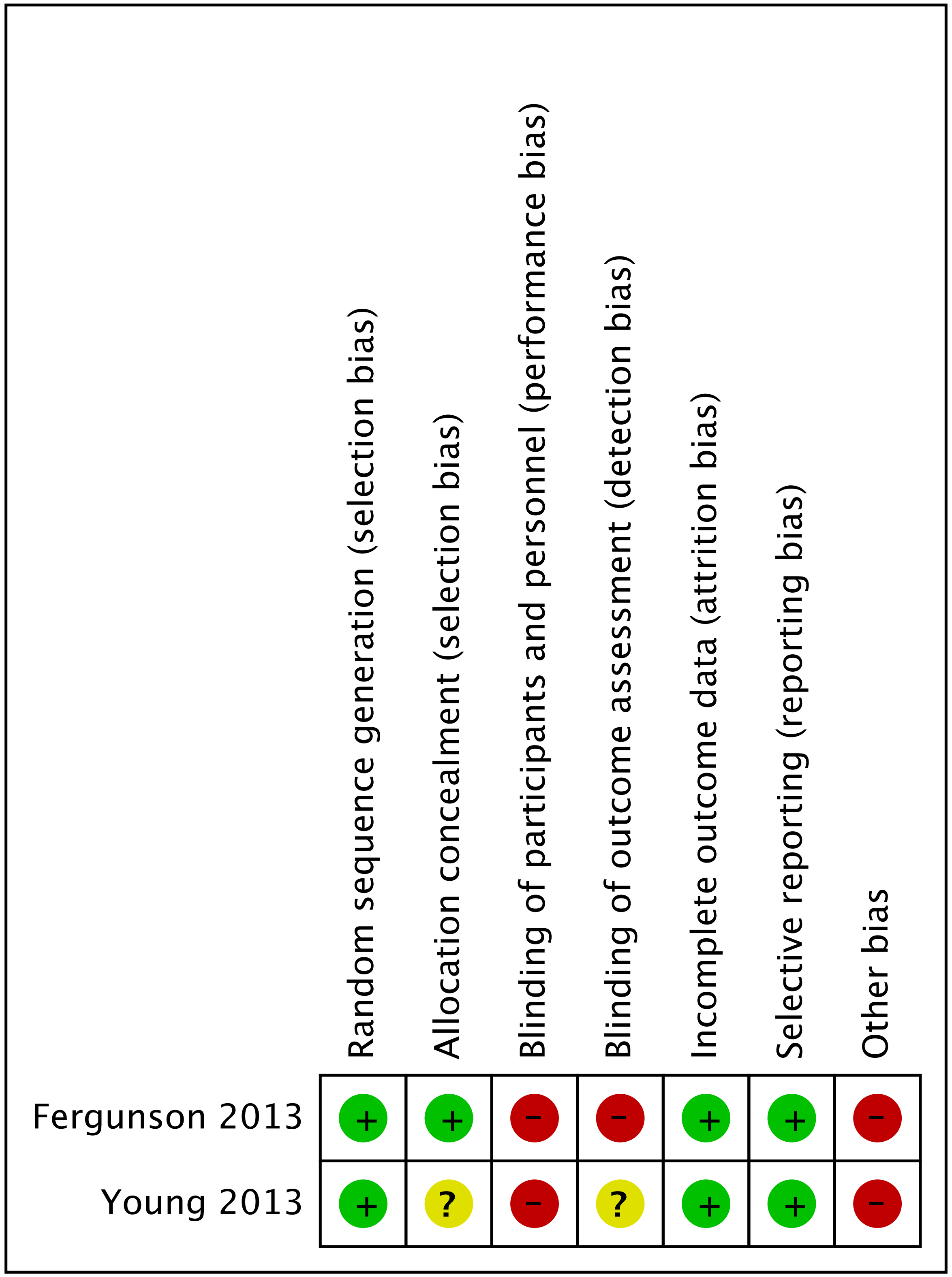

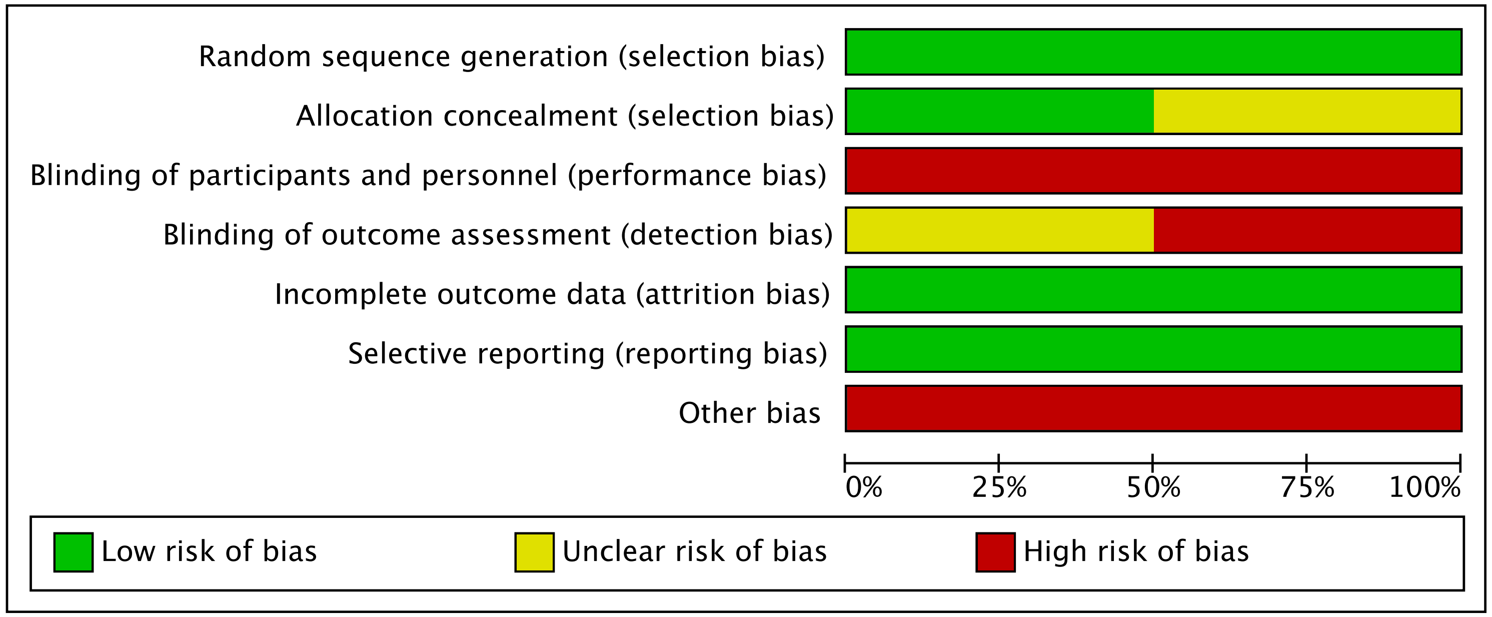

CQ08 Risk of bias summary, Risk of bias graph

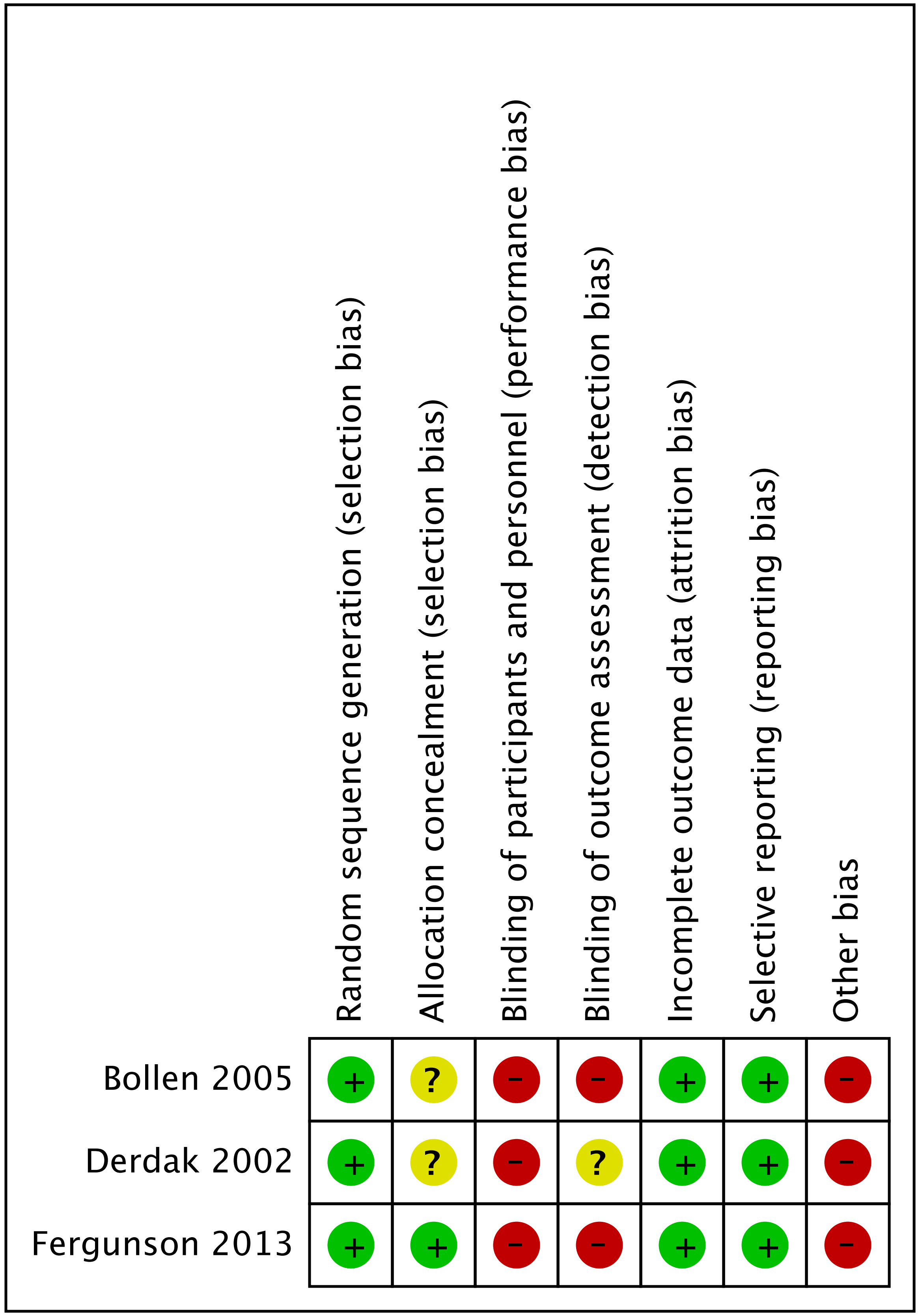

Barotrauma

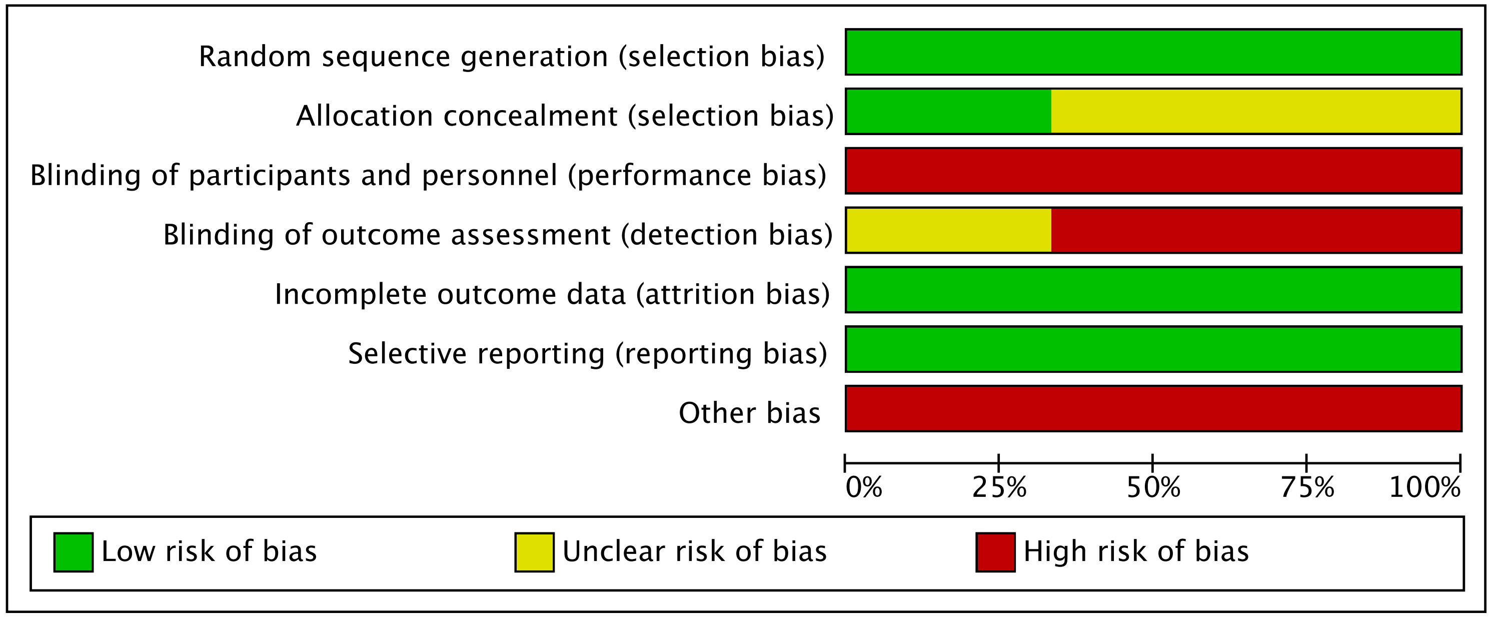

Short term mortality

Study or Subgroup	HFOV Events	HFOV Total	CMV Events	CMV Total	Weight	Risk Ratio M-H, Random, 95% CI
Bollen 2005	16	37	8	24	10.6%	1.30 [0.66, 2.55]
Derdak 2002	28	75	38	73	22.2%	0.72 [0.50, 1.03]
Ferguson 2013	129	275	96	273	32.4%	1.33 [1.09, 1.64]
Young 2013	166	398	163	397	34.9%	1.02 [0.86, 1.20]
**Total (95% CI)**		**785**		**767**	**100.0%**	**1.05 [0.82, 1.36]**
Total events	339		305			

Heterogeneity: $Tau^2 = 0.04$; $Chi^2 = 9.70$, df = 3 (P = 0.02); $I^2 = 69\%$
Test for overall effect: Z = 0.40 (P = 0.69)

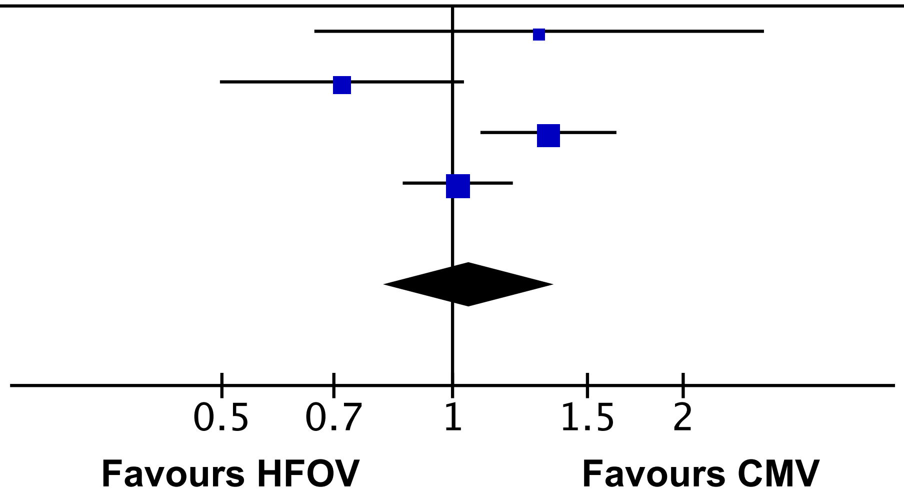

VFD

Study or Subgroup	HFOV Mean	HFOV SD	HFOV Total	CMV Mean	CMV SD	CMV Total	Weight	Mean Difference IV, Random, 95% CI
Ferguson 2013	17.6	8.7	275	17.6	8.9	273	40.3%	0.00 [−1.47, 1.47]
Young 2013	17.1	8.6	398	17.6	8.8	397	59.7%	−0.50 [−1.71, 0.71]
**Total (95% CI)**			**673**			**670**	**100.0%**	**−0.30 [−1.23, 0.64]**

Heterogeneity: $Tau^2 = 0.00$; $Chi^2 = 0.26$, df = 1 (P = 0.61); $I^2 = 0\%$
Test for overall effect: Z = 0.63 (P = 0.53)

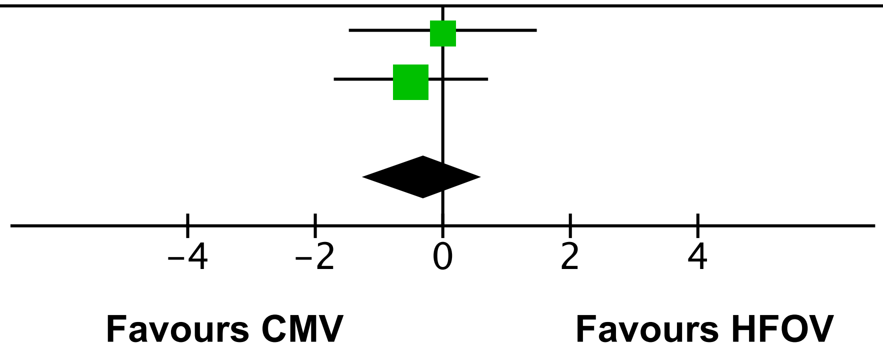

Barotrauma

Study or Subgroup	HFOV Events	HFOV Total	CMV Events	CMV Total	Weight	Risk Ratio M-H, Random, 95% CI
Bollen 2005	1	37	1	24	1.9%	0.65 [0.04, 9.88]
Derdak 2002	7	75	9	73	15.9%	0.76 [0.30, 1.93]
Ferguson 2013	46	275	34	273	82.2%	1.34 [0.89, 2.02]
**Total (95% CI)**		**387**		**370**	**100.0%**	**1.21 [0.83, 1.76]**
Total events	54		44			

Heterogeneity: $Tau^2 = 0.00$; $Chi^2 = 1.42$, df = 2 (P = 0.49); $I^2 = 0\%$
Test for overall effect: Z = 1.00 (P = 0.32)

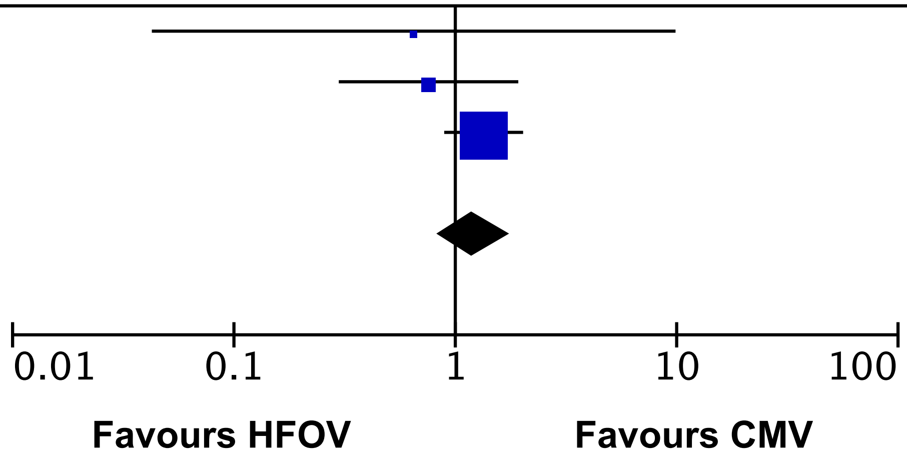

Summary of findings:

High Frequency Oscillation (HFO) compared to Conventional Mechanical Ventilation (CMV) for ARDS

Patient or population: ARDS
Intervention: High Frequency Oscillation (HFO)
Comparison: Conventional Mechanical Ventilation (CMV)

Outcomes	Anticipated absolute effects* (95% CI)		Relative effect (95% CI)	№ of participants (studies)	Quality of the evidence (GRADE)	Comments
	Risk with Conventional Mechanical Ventilation (CMV)	Risk with High Frequency Oscillation (HFO)				
Short term mortality	**Study population**		**RR 1.05** (0.82 to 1.36)	152 (4 RCTs)	⊕◯◯◯ VERY LOW 1 2 3	
	398 per 1000	**418 per 1000** (326 to 541)				
	**Low**					
	352 per 1000	**370 per 1000** (338 to 560)				
	**High**					
	412 per 1000	**433 per 1000** (358 to 644)				
VFD	Mean **17.6** days	0.3 days fewer MD (1.23 fewer to 0.64 more)		1343 (2 RCTs)	⊕⊕◯◯ LOW 1 3	
Barotrauma	**Study population**		**RR 1.21** (0.83 to 1.76)	757 (3 RCTs)	⊕◯◯◯ VERY LOW 1 3 4	
	119 per 1000	**144 per 1000** (99 to 209)				
	**Low**					
	42 per 1000	**51 per 1000** (35 to 74)				
	**High**					
	125 per 1000	**151 per 1000** (104 to 220)				

***The risk in the intervention group** (and its 95% confidence interval) is based on the assumed risk in the comparison group and the **relative effect** of the intervention (and its 95% CI).

CI: Confidence interval; **RR:** Risk ratio;

GRADE Working Group grades of evidence
High quality: We are very confident that the true effect lies close to that of the estimate of the effect
Moderate quality: We are moderately confident in the effect estimate: The true effect is likely to be close to the estimate of the effect, but there is a possibility that it is substantially different
Low quality: Our confidence in the effect estimate is limited: The true effect may be substantially different from the estimate of the effect
Very low quality: We have very little confidence in the effect estimate: The true effect is likely to be substantially different from the estimate of effect

1. Since several studies included this analysis could not make physicians blinded to intervention, the quality of evidence was downgraded by one level.
2. Since the confidence interval is partially overlapped and the heterogeneity is significant with I^2=69%, the quality of evidence was downgraded by one level.
3. Since the confidence interval is wide and is partially overlapped, the quality of evidence was downgraded by one level.
4. Since the sample size was very small, the quality of evidence was downgraded by one level.

CQ8:
Question: Should High Frequency Oscillation be used in adult patients with ARDS?

Quality assessment							№ of patients		Effect		Quality	Importance
**№ of studies**	**Study design**	**Risk of bias**	**Inconsistency**	**Indirectness**	**Imprecision**	**Other considerations**	**High Frequency Oscillation (HFO)**	**Conventional Mechanical Ventilation (CMV)**	**Relative (95% CI)**	**Absolute (95% CI)**		
Short term mortality												
4	randomised trials	serious [1]	serious [2]	not serious	serious [3]	none	339/785 (43.2%)	305/767 (39.8%)	**RR 1.05** (0.82 to 1.36)	20 more per 1000 (from 72 fewer to 143 more)	⨁◯◯◯ VERY LOW [1 2 3]	CRITICAL
								35.2%		18 more per 1000 (from 63 fewer to 127 more)		
								41.2%		21 fewer per 1000 (from 74 fewer to 148 more)		
VFD												
2	randomised trials	serious [1]	not serious	not serious	serious [1]	none	673	670		MD **0.3 day fewer** (1.23 fewer to 0.64 more)	⨁⨁◯◯ LOW [1 3]	CRITICAL
Barotrauma												
3	randomised trials	serious [1]	not serious	not serious	very serious [3 4]	none	54/387 (14.0%)	44/370 (11.9%)	**RR 1.21** (0.83 to 1.76)	25 more per 1000 (from 20 fewer to 90 more)	⨁◯◯◯ VERY LOW [1 3 4]	CRITICAL
								4.2%		9 more per 1000 (from 7 fewer to 32 more)		
								12.5%		26 more per 1000 (from 21 fewer to 95 more)		

CI: Confidence interval; **RR:** Risk ratio; **MD:** Mean difference

1. Since several studies included in this analysis could not blind physicians to the intervention, the quality of evidence was downgraded by one level.
2. Since the confidence interval is partially overlapped and the heterogeneity is significant with I^2=69%, the quality of evidence was downgraded by one level.
3. Since the confidence interval is wide and is partially overlapped, the quality of evidence was downgraded by one level.
4. Since the sample size was very small, the quality of evidence was downgraded by one level.

Evidence-to-Decision table

CQ8：Should High Frequency Oscillation be used in adult patients with ARDS?

POPULATION：ADULT PATIENTS ANTICIPATED TO REQUIRE LONG-TERM MECHANICAL VENTILATION

INTERVENTION：HIGH FREQUENCY OSCILLATION (HFO)

	CRITERIA	JUDGEMENT	RESEARCH EVIDENCE	ADDITIONAL CONSIDERATIONS
PROBLEM	**Is the problem a priority?**	○No<br>●Probably no<br>○Probably yes<br>○Yes<br>---------------<br>○Varies<br>○Don't know	It is important to avoid ventilation-related lung injuries, which may lead to prolonged ventilation or an increased mortality rate, in patients with ARDS, by using proper ventilation strategies[1]. The mortality rate due to ARDS is still high, despite enormous efforts and multiple studies to define lung protective strategies[2,3].<br>HFO is an artificial ventilation mode, which can restrict the ventilation tidal volume as well as provide a lung recruitment effect[4]. HFO has been recognized to provide lung protection, however, it is still not commonly used in adult intensive care[5]. Further studies are necessary to determine its effectiveness and safety. However, we conclude that this should not be prioritized to be resolved at the present time.	
DESIRABLE AND UNDESIRABLE EFFECTS	**What is the overall certainty of the evidence of effects?**	●Very low<br>○Low<br>○Moderate<br>○High<br>---------------<br>○No included studies	(see below)	
	**Is there important uncertainty about or variability in how much people value the main outcomes?**	○Important uncertainty or variability<br>●Possibly important uncertainty or variability<br>○Possibly no important<br>○uncertainty or variability<br>No important uncertainty or variability<br>---------------<br>○No known undesirable outcomes		
	**How substantial are the desirable anticipated effects?**	○Trivial<br>●Small<br>○Moderate<br>○Large<br>---------------<br>○Varies<br>○Don't know		
	**How substantial are the undesirable anticipated effects?**	○Large<br>●Moderate<br>○Small<br>○Trivial<br>---------------<br>○Varies<br>○Don't know		
	**Does the balance between desirable and undesirable effects favor the intervention or the comparison?**	○Favors the comparison<br>●Probably favors the comparison<br>○Does not		

The relative importance or values of the main outcomes of interest:

Outcomes	Relative importance	Certainty of the evidence (GRADE)
Short term mortality (note 1	CRITICAL	⊕○○○<br>VERY LOW
VFD (note 2	CRITICAL	⊕⊕○○<br>LOW
Barotrauma	CRITICAL	⊕○○○<br>VERY LOW

Summary of findings:

Outcomes	Risk with Conventional Mechanical Ventilation (CMV)	Risk with High Frequency Oscillation (HFO)	Absolute (95% CI)	Relative effect (95% CI)
Short term mortality (note 1	398 / 1000	418 / 1000 (326 to 541)	20 more per 1000 (from 72 fewer to 143 more)	RR 1.05 (0.82 to 1.36)
	352 / 1000	370 / 1000 (338 to 560)	18 more per 1000 (from 63 fewer to 127 more)	
	412 / 1000	433 /1000 (358 to 644)	21 fewer per 1000 (from 74 fewer to 148 more)	
VFD (note 2	Average 17.6 days	Average 17.3 day	MD 0.3 day fewer (1.23 fewer to 0.64 more)	-
Barotrauma	119 / 1000	144 / 1000 (99 to 209)	25 more per 1000 (from 20 fewer to 90 more)	RR 1.21 (0.83 to 1.76)
	42 / 1000	51 / 1000 (35 to 74)	9 more per 1000 (from 7 fewer to 32 more)	
	125 / 1000	151 / 1000 (104 to 220)	26 more per 1000 (from 21 fewer to 95 more)	

Summary: Four randomized controlled trials (RCT) were found for evaluating HFO in adult patients with ARDS. There was no statistically significant difference in mortality rate and ventilation free days (VFD) by utilizing HFO; mortality rate (Relative Risk (RR): 1.05, 95% Confidence Interval (CI): 0.82 - 1.36) and VFD (mean difference: -0.30 days, 95%CI: -1.23 - 0.64). Although there is no statistically significant difference, the results show an increasing trend in the incidence of barotrauma in patients treated with HFO (RR: 1.21, 95%CI: 0.83 -

		favor either the intervention or the comparison<br>○Probably favors the intervention<br>○Favors the intervention<br>――――――<br>○Varies<br>○Don't know	1.36).	
RESOURCES REQUIRED	**How large are the resource requirements (costs)?**	●Large costs<br>○Moderate costs<br>○Negligible costs and savings<br>○Moderate savings<br>○Large savings<br>――――――<br>○Varies<br>○Don't know	In order to use HFO, a dedicated ventilator is needed (approximately 10 million yen). There is no additional cost if the facility already has access to HFO. Since HFO is not commonly used in adults in Japan, few facilities have the equipment, and the cost for introduction is high.	
	**Does the cost-effectiveness of the intervention favor the intervention or the comparison?**	○Favors the comparison<br>●Probably favors the comparison<br>○Does not favor either the intervention or the comparison<br>○Probably favors the intervention<br>○Favors the intervention<br>――――――<br>○Varies<br>○No included studies	The overall benefit for patients by introducing HFO is likely not to be great, based on the results of this review. Furthermore, the estimated cost increase likely exceeds the potential benefits except in facilities which already have access to HFO.	
EQUITY	**What would be the impact on health equity?**	○Reduced<br>●Probably reduced<br>○Probably no impact<br>○Probably increased<br>○Increased<br>――――――<br>○Varies<br>○Don't know	Inequity on whether facilities can have an HFO dedicated ventilator may occur.	
ACCEPTABILITY	**Is the intervention acceptable to key stakeholders?**	○No<br>●Probably no<br>○Probably yes<br>○Yes<br>――――――<br>○Varies<br>○Don't know	In facilities that must acquire an expensive HFO dedicated ventilator, since there are few obvious benefits, acceptance is expected to be difficult. Educating staff in the appropriate use in each department is also labor intensive, and adds to the difficulty of acceptance.	
FEASIBILITY	**Is the intervention feasible implement?**	○No<br>○Probably no<br>○Probably yes<br>○Yes<br>――――――<br>●Varies<br>○Don't know	The intervention of this CQ is an artificial ventilation mode, but adaptation to the patient is feasible. It is necessary to acquire an expensive dedicated ventilator in order to use HFO.	

Recommendation

CQ8 : **Should High Frequency Oscillation be used in adult patients with ARDS?**					
**Balance of consequences**	Undesirable consequences clearly outweigh desirable consequences in most settings	Undesirable consequences probably outweigh desirable consequences in most settings	The balance between desirable and undesirable consequences is closely balanced or uncertain	Desirable consequences probably outweigh undesirable consequences in most settings	Desirable consequences clearly outweigh undesirable consequences in most settings
Judgement	○	○	●	○	○

**Type of recommendation**	Strong recommendation against the intervention	Conditional recommendation against the intervention	Conditional recommendation for either the intervention or the comparison	Conditional recommendation for the intervention
Judgement	○	●	○	○

**Recommendation**	**We suggest against the use of High Frequency Oscillation (HFO) in adult patients with ARDS. (GRADE 2C , Strength of recommendation "weak recommendation" / Quality of evidence: "low")**
**Justification**	**Question:** Should high frequency oscillation be used in adult patients with ARDS?<br>**Patients:** Adult patients anticipated to require long-term mechanical ventilation<br>**Interventions:** High Frequency Oscillation (HFO)<br>**Comparison:** Conventional Mechanical Ventilation (CMV)<br>**Outcomes:** Short-term mortality (note 1, VFD (note 2, Barotrauma<br><br>**Summary of the evidence:** Four randomized controlled trials (RCT) were found for evaluating HFO in adult patients with ARDS. There was no statistically significant difference in mortality rate and ventilation free days (VFD) by utilizing HFO; mortality rate (Relative Risk (RR): 1.05, 95% Confidence Interval (CI): 0.82 - 1.36) and VFD (mean difference: -0.30 days, 95%CI: -1.23 - 0.64). Although there is no statistically significant difference, the results show an increasing trend in the incidence of barotrauma in patients treated with HFO (RR: 1.21, 95%CI: 0.83 - 1.36).<br><br>**Quality of the evidence:** Studies which use ventilation settings as an intervention cannot be performed in a double blinded manner, and the selected studies were no exceptions. The high likelihood of critical selection biases were estimated for all selected studies. One study was conducted with an intent-to-treat-analysis, however, a cross-over RCT was used as the design (6). One study did not provide a patient flow diagram which can be important to decide the quality of the study (7). The other two studies excluded more than half of the initial participants without a clear description of selection criteria in the manuscript (8, 9). Heterogeneity among studies can be high with I2 statistics of 69%. Indirectness in the four selected studies was not sufficiently obvious to lower the evidence grade, given other factors. Precision was considered to be low given the wide 95%CIs, although the number of included cases seemed adequate. Due to the small number of studies selected, publication bias could not be examined. In conclusion, the overall quality of evidence was concluded as low. This included very low for mortality, low for VFD, and very low for barotrauma, respectively.<br><br>**Judgement of benefit and harm, resources and cost:** Given that most available ventilators cannot provide a HFO mode, in order to introduce HFO as a new modality, a facility needs to invest a large amount of money. Benefits are estimated to be relatively small even with increased spending, considering the results of this review. Although there was an increasing tendency for the development of barotrauma in the HFO group, no statistically significant difference could be seen, hence, the potential for harm secondary to HFO is concluded as low.<br><br>**Recommendations;** We suggest against the use of High Frequency Oscillation (HFO) in adult patients with ARDS. (GRADE 2C, Strength of recommendation "weak recommendation" / Quality of evidence: "low")<br><br>**Additional considerations:** We emphasize that this recommendation is for adult patients. HFO for adult patients with ARDS is not commonly used in current practice as compared to pediatric patients or neonates. HFO can be harmful if applied improperly. However, when used correctly, it can provide better outcomes with fewer complications.<br><br>In the panel meeting, a vote was conducted because of inability to achieve consensus. As a result, it was

	proposed not to use HFO (weak recommendation); four answered for "not to recommend" (strong recommendation) and eight voted for "not to propose" (weak recommendation) respectively.
**Subgroup considerations**	None
**Implementation considerations**	Since management requiring specialized knowledge and experience with previous ventilator therapy is needed, staff education about how to use and troubleshoot this modality is very important. The high cost, clinical complications (pneumothorax, obstruction) and several complicated issues in HFO settings (target of mean airway pressure, carbon dioxide management, cuff leak management, selection of the proper frequency, etc.) must also be considered.
**Monitoring and evaluation considerations**	Standard monitoring for oxygenation status, ventilation, and work of breathing are sufficient. It is not feasible to examine ventilation using tidal ventilation volume, end-tidal CO_2, or lung sounds in patients receiving HFO, thus alternative monitoring is necessary.
**Research possibilities**	Two of the selected studies(10, 11) adopted a P/F ratio ≤ 200 as the inclusion criteria, therefore, a significant number of patients with moderate ARDS are included, which might lead to dilute the effects of HFO. The effect of HFO in the patients with severe ARDS, which is unable to be managed with a conventional lung protective strategies, should be evaluated in the future studies.

Note 1) Short time mortality is defined as death at the end of the study.

Note 2) Count days off ventilator (until day 28), for subjects who die, ventilator free days equals 0.

References

1. Esteban A, Ferguson ND, Meade MO, et al. Evolution of mechanical ventilation in response to clinical research. Am J Respir Crit Care Med 177 (2): 170-7, 2008. PMID 17962636
2. ARDS Network. Ventilation with lower tidal volumes as compared with traditional tidal volumes for acute lung injury and the acute respiratory distress syndrome. The Acute Respiratory Distress Syndrome Network. N Engl J Med 342 (18): 1301-8, 2000. PMID 10793162
3. Phua J, Badia JR, Adhikari NK, et al. Has mortality from acute respiratory distress syndrome decreased over time?: A systematic review. Am J Respir Crit Care Med 179 (3): 220-7, 2009. PMID 19011152
4. Rimensberger PC. ICU cornerstone: high frequency ventilation is here to stay. Crit Care 7 (5): 342-4, 2003. PMID 12974963
5. Ferguson ND, Slutsky AS. Point: High-frequency ventilation is the optimal physiological approach to ventilate ARDS patients. J Appl Physiol (1985) 104 (4): 1230-1, 2008. PMID 18048584
6. Derdak S, Mehta S, Stewart TE, et al. High-frequency oscillatory ventilation for acute respiratory distress syndrome in adults: a randomized, controlled trial. Am J Respir Crit Care Med 166 (6): 801-8, 2002. PMID 12231488
7. Bollen CW, van Well GT, Sherry T, et al. High frequency oscillatory ventilation compared with conventional mechanical ventilation in adult respiratory distress syndrome: a randomized controlled trial [ISRCTN24242669]. Crit Care 9 (4): R430-9, 2005. PMID 16137357
8. Claesson J, Freundlich M, Gunnarsson I, et al. Scandinavian clinical practice guideline on mechanical ventilation in adults with the acute respiratory distress syndrome. Acta Anaesthesiol Scand 59 (3): 286-97, 2015. PMID 25524779
9. Sud S, Sud M, Friedrich JO, et al. High-frequency ventilation versus conventional ventilation for treatment of acute lung injury and acute respiratory distress syndrome. Cochrane Database Syst Rev 2 : CD004085, 2013. PMID 23450549
10. Young D, Lamb SE, Shah S, et al. High-frequency oscillation for acute respiratory distress syndrome. N Engl J Med 368 (9): 806-13, 2013. PMID 23339638
11. Ferguson ND, Cook DJ, Guyatt GH, et al. High-frequency oscillation in early acute respiratory distress syndrome. N Engl J Med 368 (9): 795-805, 2013. PMID 23339639

CQ09. Study flow diagram

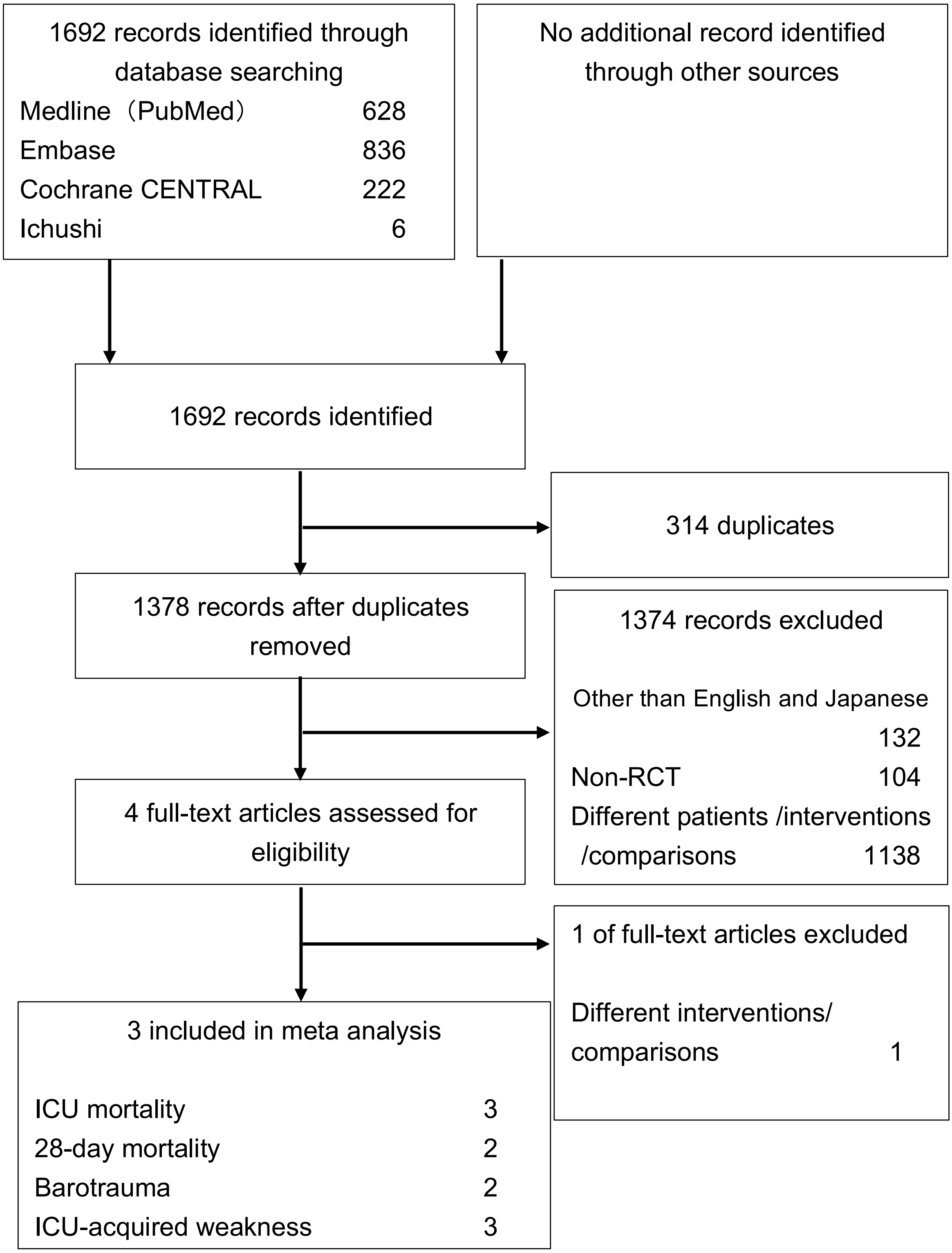

CQ09

Risk of bias table, ICU mortality

Outcome		ICU mortality		risk of bias		not serious (0)			
番号	著者名 発表年（Forest plot表示）	risk of bias評価							
		ランダム割付順番の生成 random sequence generation	割り付けの隠蔽化 allocation concealment	ブラインド blinding 研究参加者と治療提供者 participants and personnel	ブラインド blinding アウトカム評価者 outcome assessors	不完全なアウトカムデータ incomplete outcome data	選択されたアウトカムの報告 selective outcome reporting	その他のバイアス Other sources of bias	研究内でのバイアスのリスク Risk of bias within a study
1	Forel 2006	Unclear risk	Low risk	High risk	Low risk	Low risk	Low risk	Low risk	Low risk
2	Gainnier 2004	Unclear risk	Low risk	High risk	Low risk	Low risk	Low risk	Low risk	Low risk
3	Papazian 2010	Low risk	Low risk	Low risk	Low risk	Low risk	Low risk	Low risk	Low risk

番号	著者名 発表年（Forest plot表示）	risk of biasコメント							
1	Forel 2006	ランダム化の方法が未記載	ランダム化は変動サイズブロックで等数になるように割り付けられた. 研究者には割り付けを知らされていなかった.	担当看護師は筋弛緩薬の割り付けかどうかを知っていた.	研究者には割り付けを知らされていなかった.	100%フォローされた	100%報告された	研究の中断なし	全項目ほぼLow risk
2	Gainnier 2004	ランダム化の方法が未記載	研究者たちは全員, 割り付けやブロックサイズを知らされていなかった.	担当看護師は盲検化されておらず, 適切な筋弛緩が得られるまでプロトコルに基づいた流量調整を行った.	研究者には割り付けを知らされていなかった.	100%フォローされた	100%報告された	研究の中断なし	全項目ほぼLow risk
3	Papazian 2010	本研究は独立した機関によってデータと安全性を監視された. ランダム化と盲検化はCONSORTガイドラインに準じて行われた.	本研究は独立した機関によってデータと安全性を監視された. ランダム化と盲検化はCONSORTガイドラインに準じて行われた.	多施設共同ランダム化プラセボ対照二重盲検試験	多施設共同ランダム化プラセボ対照二重盲検試験	99.7% (339/340)フォローされた. 筋弛緩群の1名は治療開始前に同意撤回したため, 解析から除外した.	100%報告された	研究の中断なし	全項目ともLow risk

CQ09
Risk of bias table, 28-day mortality

28-day mortality		risk of bias		not serious (0)			
risk of bias評価							
ランダム割付順番の生成 random sequence generation	割り付けの隠蔽化 allocation concealment	ブラインド blinding		不完全なアウトカムデータ incomplete outcome data	選択されたアウトカムの報告 selective outcome reporting	その他のバイアス Other sources of bias	研究内でのバイアスのリスク Risk of bias within a study
		研究参加者と治療提供者 participants and personnel	アウトカム評価者 outcome assessors				
Unclear risk	Low risk	High risk	Low risk	Low risk	Low risk	Low risk	Low risk
Low risk	Low risk	Low risk	Low risk	Low risk	Low risk	Low risk	Low risk
risk of biasコメント							
ランダム化の方法が未記載	研究者たちは全員，割り付けやブロックサイズを知らされていなかった.	担当看護師は盲検化されておらず，適切な筋弛緩が得られるまでプロトコルに基づいた流量調整を行った.	研究者には割り付けを知らされていなかった.	100%フォローーされた	100%報告された	研究の中断なし	全項目ほぼLow risk
本研究は独立した機関によってデータと安全性を監視された. ランダム化と盲検化はCONSORTガイドラインに準じて行われた.	本研究は独立した機関によってデータと安全性を監視された. ランダム化と盲検化はCONSORTガイドラインに準じて行われた.	多施設共同ランダム化プラセボ対照二重盲検試験	多施設共同ランダム化プラセボ対照二重盲検試験	99.7% (339/340)フォローーされた. 筋弛緩群の1名は治療開始前に同意撤回したため，解析から除外した.	100%報告された	研究の中断なし	全項目ともLow risk

CQ09
Risk of bias table, barotrauma

Outcome		Barotrauma		risk of bias		not serious (0)			
番号	著者名 発表年（Forest plot表示）	risk of bias評価							
		ランダム割付順番の生成 random sequence generation	割り付けの隠蔽化 allocation concealment	ブラインド blinding 研究参加者と治療提供者 participants and personnel	ブラインド blinding アウトカム評価者 outcome assessors	不完全なアウトカムデータ incomplete outcome data	選択されたアウトカムの報告 selective outcome reporting	その他のバイアス Other sources of bias	研究内でのバイアスのリスク Risk of bias within a study
2	Gainnier 2004	Unclear risk	Low risk	High risk	Low risk	Low risk	Low risk	Low risk	Low risk
3	Papazian 2010	Low risk	Low risk	Low risk	Low risk	Low risk	Low risk	Low risk	Low risk

番号	著者名 発表年（Forest plot表示）	risk of biasコメント							
1	Gainnier 2004	ランダム化の方法が未記載	研究者たちは全員，割り付けやブロックサイズを知らされていなかった.	担当看護師は盲検化されておらず，適切な筋弛緩が得られるまでプロトコルに基づいた流量調整を行った.	研究者には割り付けを知らされていなかった.	100%フォローされた	100%報告された	研究の中断なし	全項目ほぼLow risk
2	Papazian 2010	本研究は独立した機関によってデータと安全性を監視された. ランダム化と盲検化はCONSORTガイドラインに準じて行われた.	本研究は独立した機関によってデータと安全性を監視された. ランダム化と盲検化はCONSORTガイドラインに準じて行われた.	多施設共同ランダム化プラセボ対照二重盲検試験	多施設共同ランダム化プラセボ対照二重盲検試験	99.7% (339/340)フォローされた. 筋弛緩群の1名は治療開始前に同意撤回したため，解析から除外した.	100%報告された	研究の中断なし	全項目ともLow risk

CQ09

Risk of bias table, ICU-acquired weakness

Outcome		ICU-acquired weakness		risk of bias		not serious (0)			
番号	著者名 発表年（Forest plot表示）	risk of bias評価							
		ランダム割付順番の生成 random sequence generation	割り付けの隠蔽化 allocation concealment	ブラインド blinding		不完全なアウトカムデータ incomplete outcome data	選択されたアウトカムの報告 selective outcome reporting	その他のバイアス Other sources of bias	研究内でのバイアスのリスク Risk of bias within a study
				研究参加者と治療提供者 participants and personnel	アウトカム評価者 outcome assessors				
1	Forel 2006	Unclear risk	Low risk	High risk	Low risk	Low risk	Low risk	Low risk	Low risk
2	Gainnier 2004	Unclear risk	Low risk	High risk	Low risk	Low risk	Low risk	Low risk	Low risk
3	Papazian 2010	Low risk	Low risk	Low risk	Low risk	Low risk	Low risk	Low risk	Low risk
番号	著者名 発表年（Forest plot表示）	risk of biasコメント							
1	Forel 2006	ランダム化の方法が未記載	ランダム化は変動サイズブロックで等数になるように割り付けられた．研究者には割り付けを知らされていなかった．	担当看護師は筋弛緩薬の割り付けかどうかを知っていた．	研究者には割り付けを知らされていなかった．	100%フォローされた	100%報告された	研究の中断なし	Low risk 3項目，Unclear 3項目<br>厳しい評価の方を採用
2	Gainnier 2004	ランダム化の方法が未記載	研究者たちは全員，割り付けやブロックサイズを知らされていなかった．	担当看護師は盲検化されておらず，適切な筋弛緩が得られるまでプロトコルに基づいた流量調整を行った．	研究者には割り付けを知らされていなかった．	100%フォローされた	100%報告された	研究の中断なし	全項目ほぼLow risk
3	Papazian 2010	本研究は独立した機関によってデータと安全性を監視された．ランダム化と盲検化はCONSORTガイドラインに準じて行われた．	本研究は独立した機関によってデータと安全性を監視された．ランダム化と盲検化はCONSORTガイドラインに準じて行われた．	多施設共同ランダム化プラセボ対照二重盲検試験	多施設共同ランダム化プラセボ対照二重盲検試験	99.7% (339/340)フォローされた．筋弛緩群の1名は治療開始前に同意撤回したため，解析から除外した．	100%報告された	研究の中断なし	全項目ともLow risk

Mortality in ICU

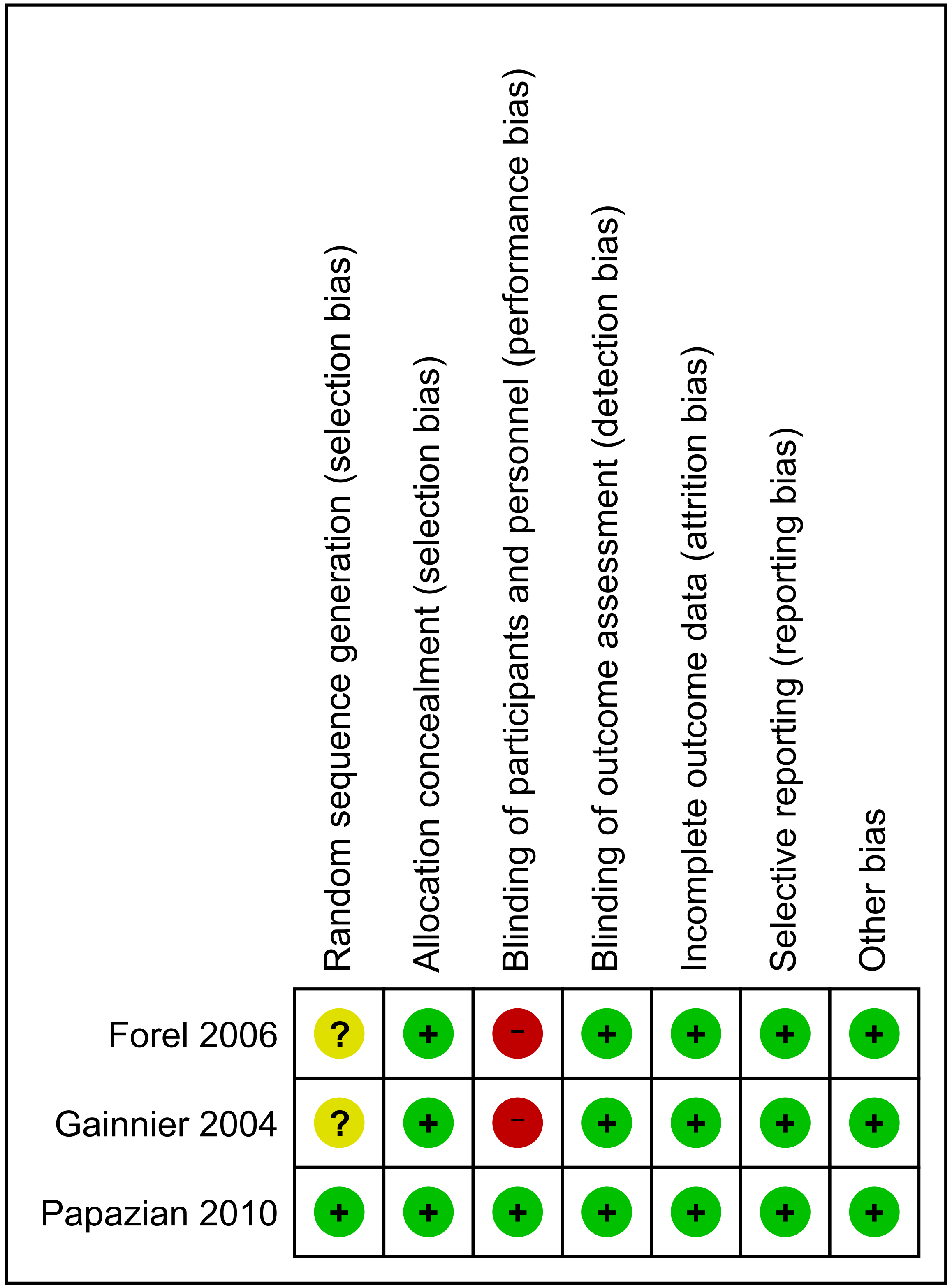

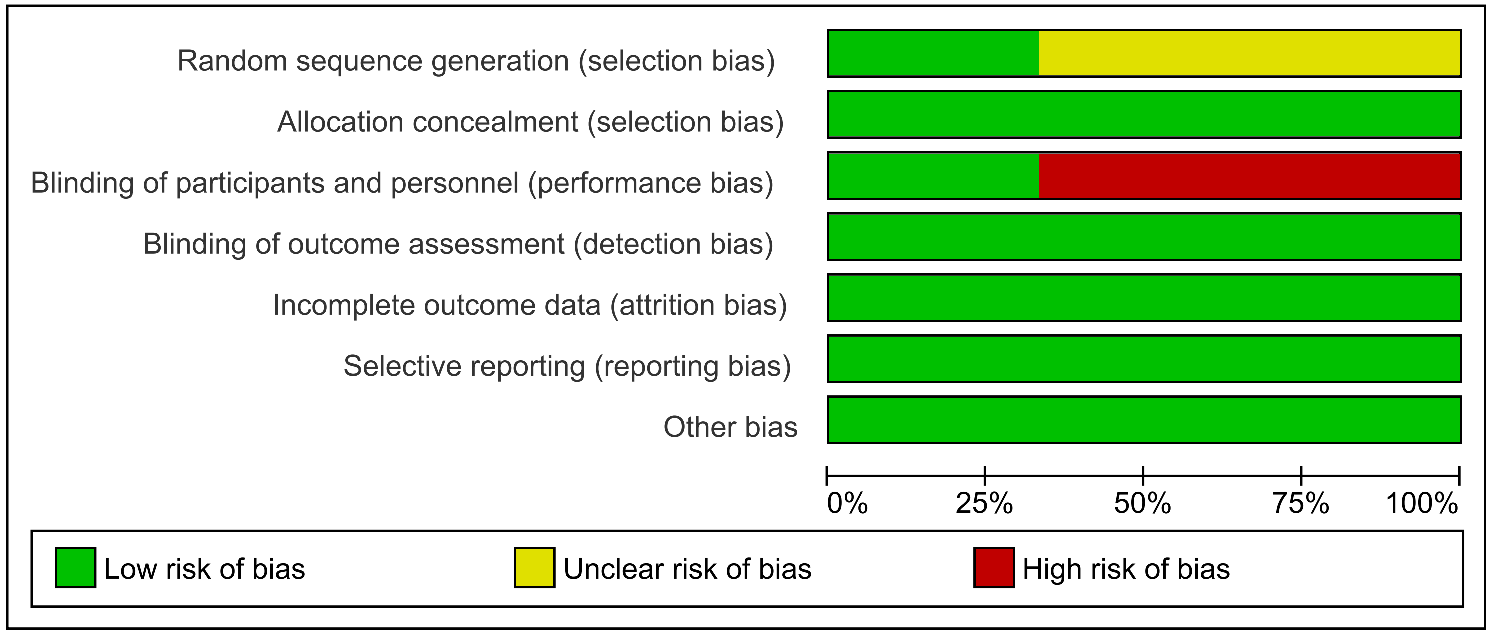

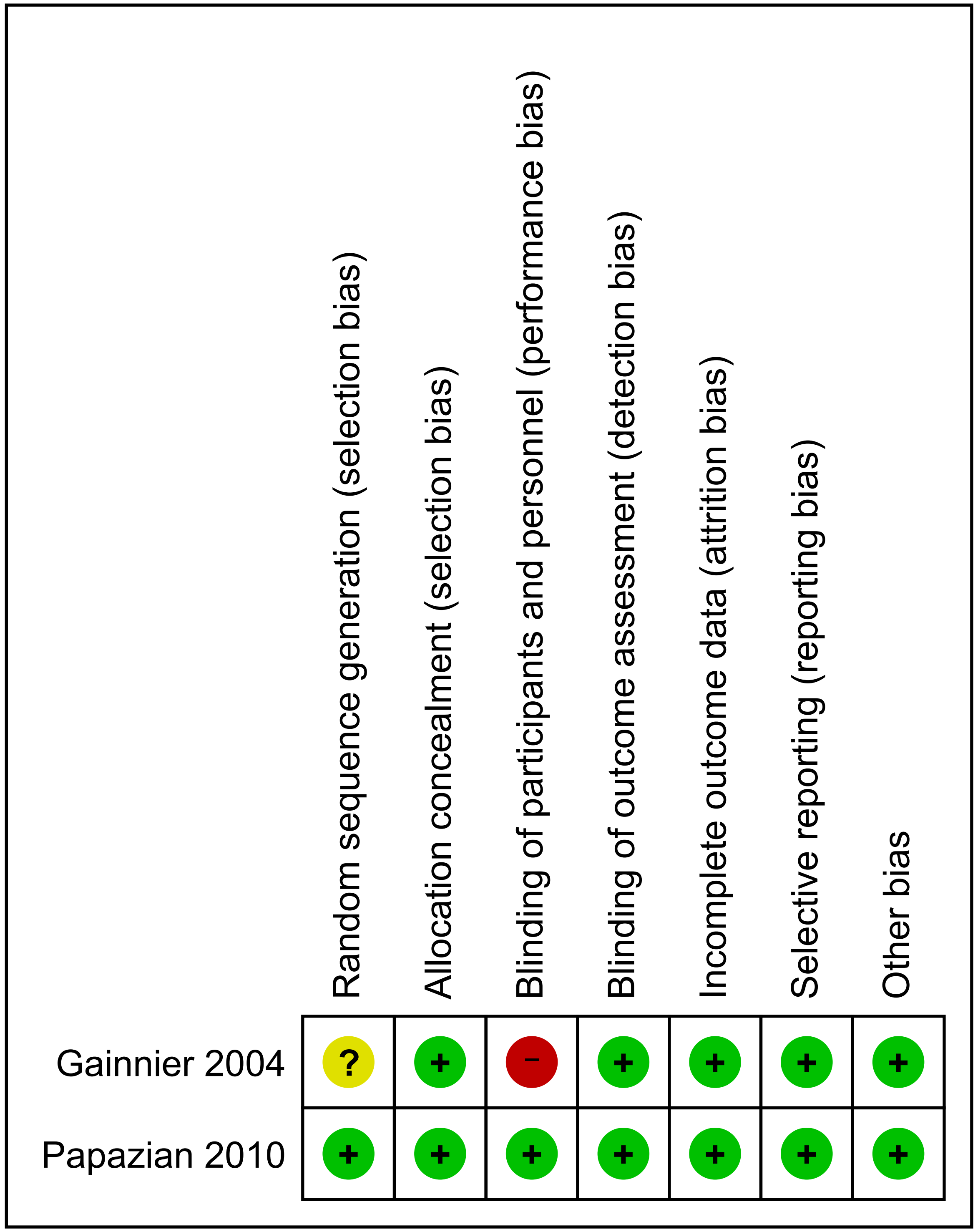

Mortality (28 days)

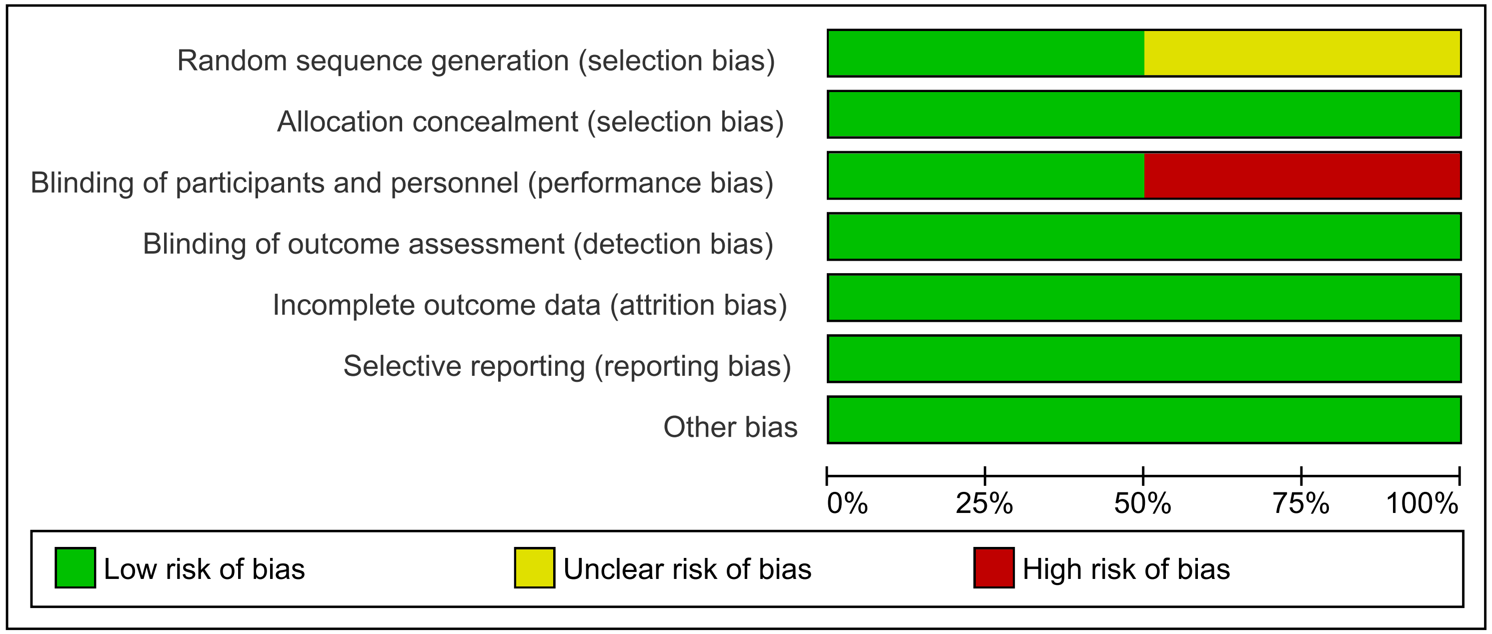

CQ09 Risk of bias summary, Risk of bias graph

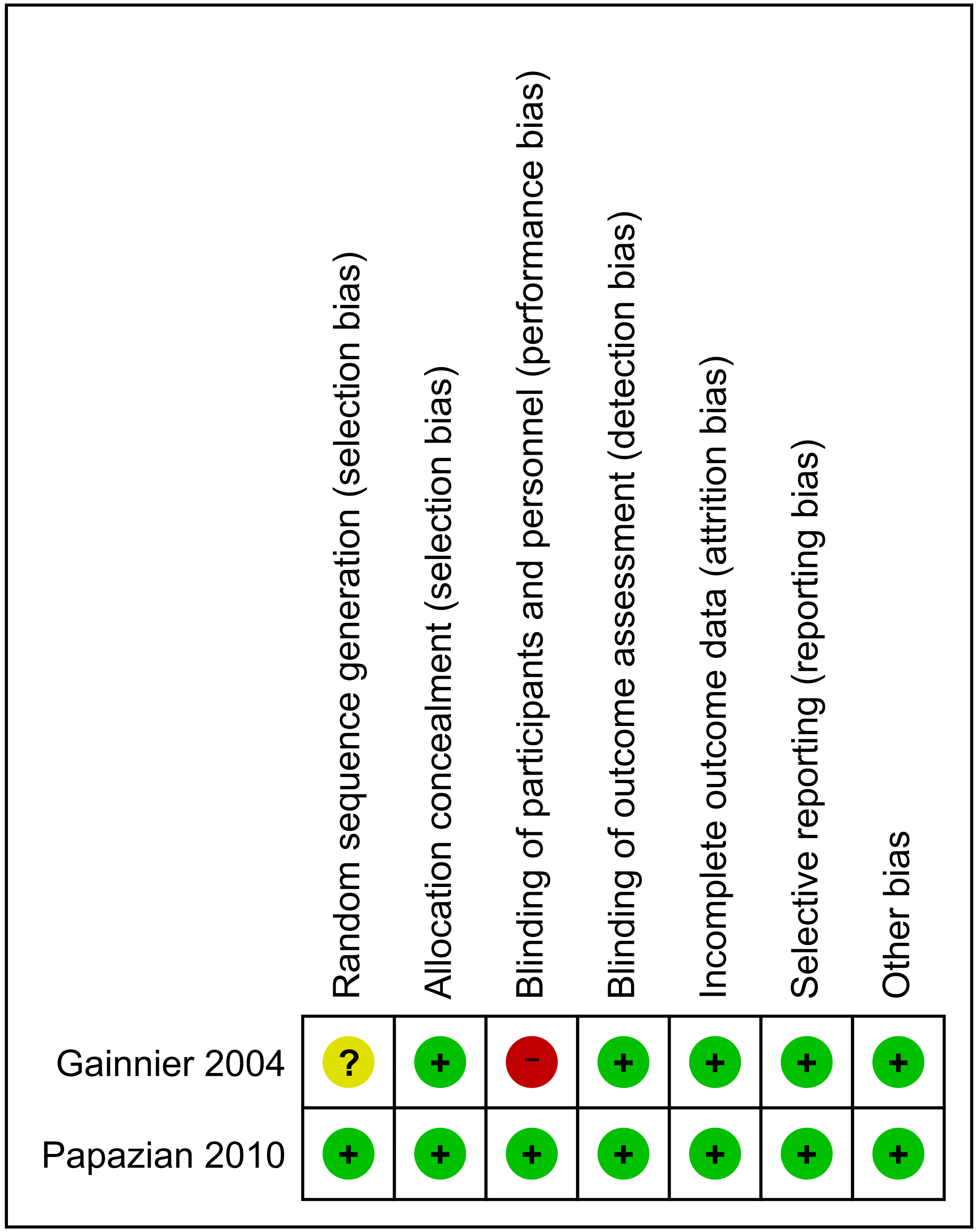

Barotrauma

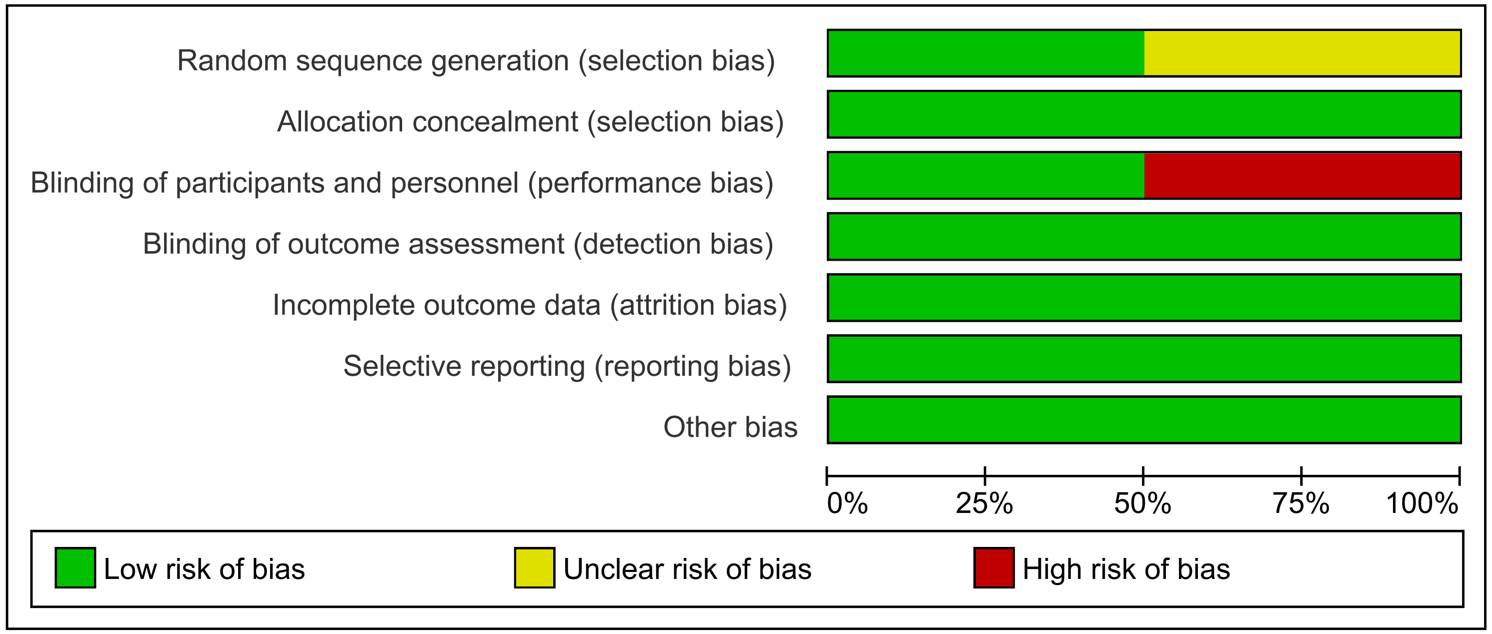

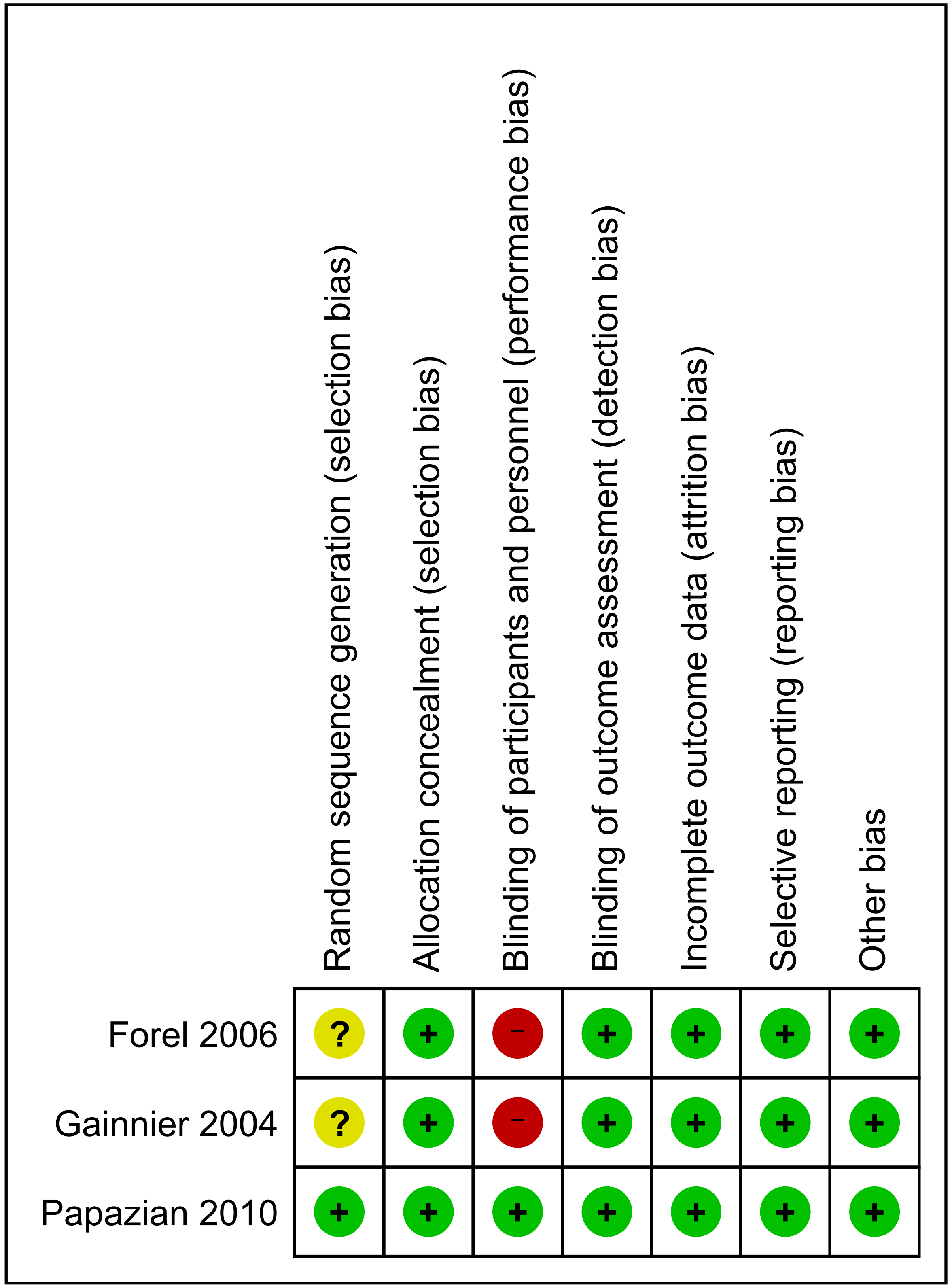

ICU acquired weakness

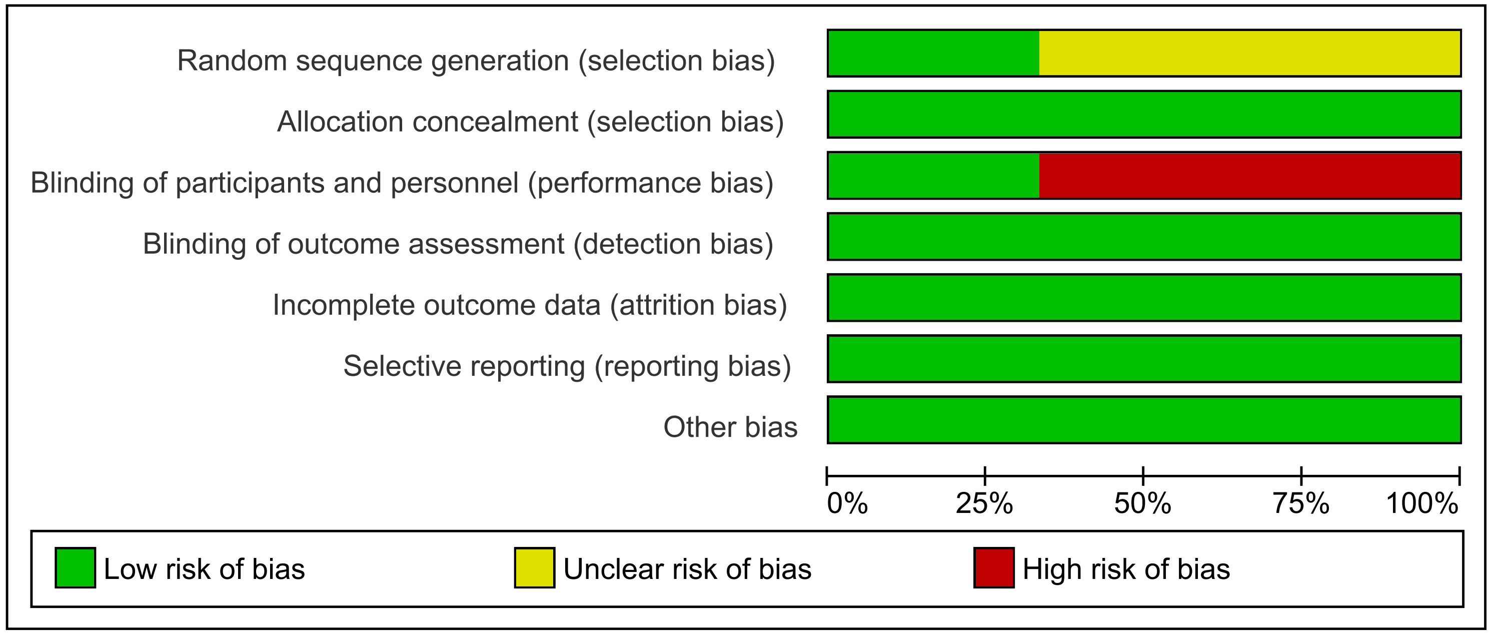

Mortality in ICU

Study or Subgroup	Cisatracurium Events	Cisatracurium Total	Placebo Events	Placebo Total	Weight	Risk Ratio M-H, Random, 95% CI
Forel 2006	5	18	10	18	8.0%	0.50 [0.21, 1.17]
Gainnier 2004	13	28	20	28	27.2%	0.65 [0.41, 1.03]
Papazian 2010	52	177	63	162	64.8%	0.76 [0.56, 1.02]
**Total (95% CI)**		**223**		**208**	**100.0%**	**0.70 [0.55, 0.89]**
Total events	70		93			

Heterogeneity: Tau² = 0.00; Chi² = 0.95, df = 2 (P = 0.62); I² = 0%
Test for overall effect: Z = 2.88 (P = 0.004)

Risk Ratio
M-H, Random, 95% CI

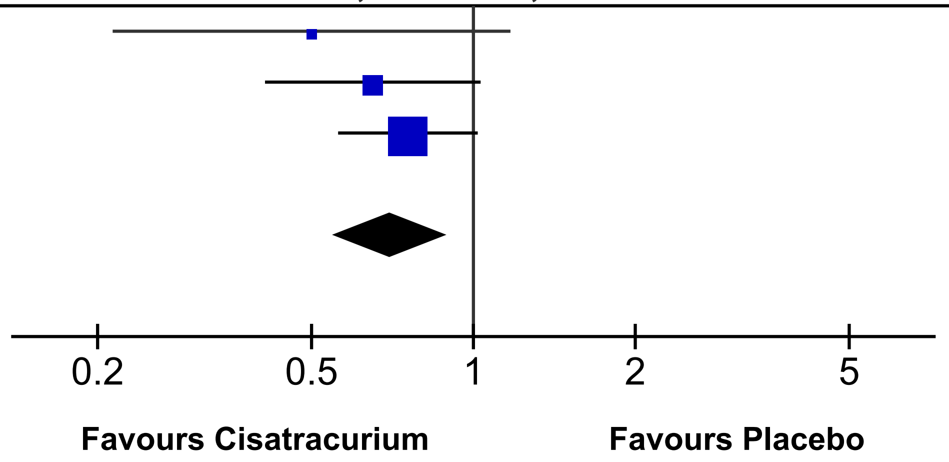

Mortality (28 days)

Study or Subgroup	Cisatracurium Events	Cisatracurium Total	Placebo Events	Placebo Total	Weight	Risk Ratio M-H, Random, 95% CI
Gainnier 2004	10	28	17	28	25.9%	0.59 [0.33, 1.05]
Papazian 2010	42	177	54	162	74.1%	0.71 [0.51, 1.00]
**Total (95% CI)**		**205**		**190**	**100.0%**	**0.68 [0.50, 0.91]**
Total events	52		71			

Heterogeneity: Tau² = 0.00; Chi² = 0.31, df = 1 (P = 0.58); I² = 0%
Test for overall effect: Z = 2.59 (P = 0.010)

Risk Ratio
M-H, Random, 95% CI

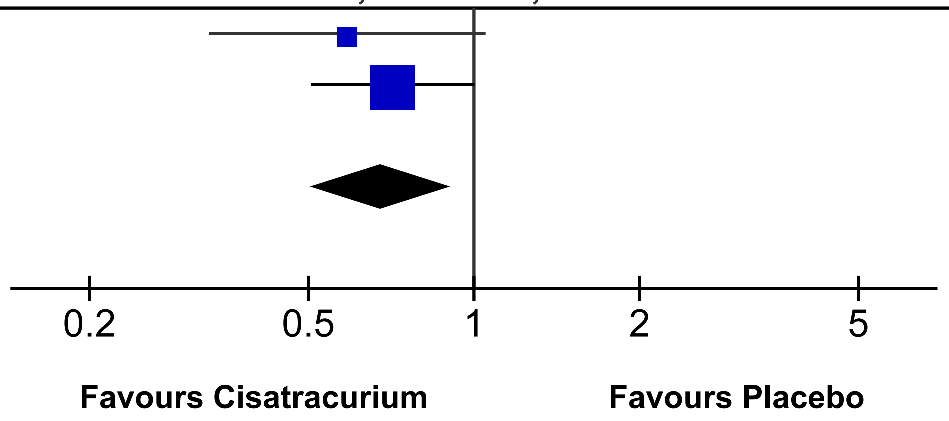

Barotrauma

	Cisatracurium		Placebo			Risk Ratio
**Study or Subgroup**	**Events**	**Total**	**Events**	**Total**	**Weight**	**M-H, Random, 95% CI**
Gainnier 2004	0	28	1	28	5.5%	0.33 [0.01, 7.85]
Papazian 2010	9	177	19	162	94.5%	0.43 [0.20, 0.93]
**Total (95% CI)**		**205**		**190**	**100.0%**	**0.43 [0.20, 0.90]**
Total events	9		20			

Heterogeneity: $Tau^2 = 0.00$; $Chi^2 = 0.03$, df = 1 (P = 0.87); $I^2 = 0\%$
Test for overall effect: Z = 2.24 (P = 0.02)

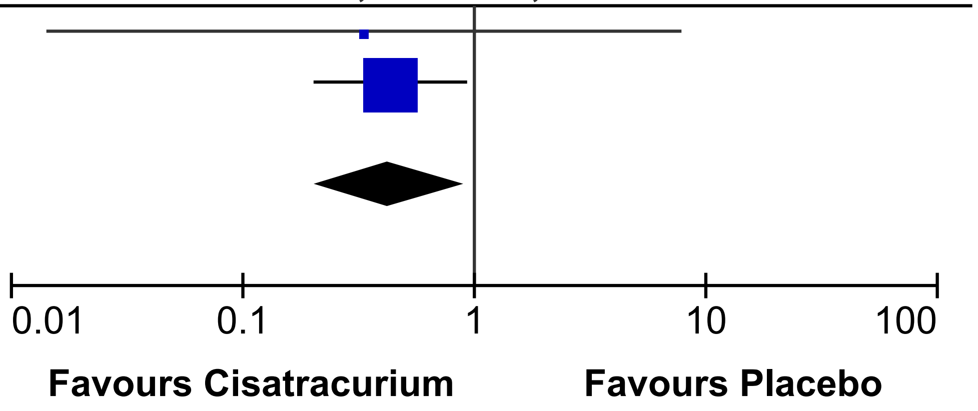

ICU acquired weakness

	Cisatracurium		Placebo			Risk Ratio
**Study or Subgroup**	**Events**	**Total**	**Events**	**Total**	**Weight**	**M-H, Random, 95% CI**
Forel 2006	1	18	1	18	2.1%	1.00 [0.07, 14.79]
Gainnier 2004	0	28	0	28		Not estimable
Papazian 2010	40	112	28	89	97.9%	1.14 [0.77, 1.68]
**Total (95% CI)**		**158**		**135**	**100.0%**	**1.13 [0.77, 1.67]**
Total events	41		29			

Heterogeneity: $Tau^2 = 0.00$; $Chi^2 = 0.01$, df = 1 (P = 0.93); $I^2 = 0\%$
Test for overall effect: Z = 0.62 (P = 0.53)

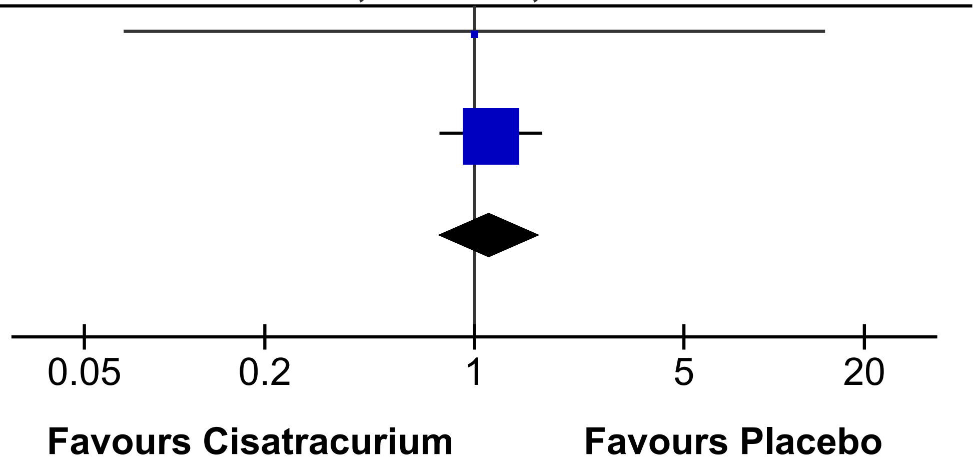

Summary of findings:

Neuromuscular blocker for adult ARDS compared to placebo for adult ARDS

Patient or population : Moderate to severe adult patients with ARDS within 48 hours from the onset
Intervention : Neuromuscular blocker (Cisatracrium) for 48-hour infusion
Comparison : Placebo

Outcomes	Anticipated absolute effects* (95% CI)		Relative effect (95% CI)	№ of participants (studies)	Quality of the evidence (GRADE)	Comments
	**Risk with placebo**	**Risk with NMB**				
ICU mortality	**Study population**		**RR 0.70** (0.55 to 0.89)	431 (3 RCTs)	⊕⊕⊕◯ MODERATE [1,2]	
	447 per 1000	**313 per 1000** (246 to 398)				
	**Low risk population**					
	313 per 1000	**219 per 1000** (172 to 279)				
	**High risk population**					
	389 per 1000	**272 per 1000** (214 to 346)				
28-day mortality	**Study population**		**RR 0.68** (0.50 to 0.91)	395 (2 RCTs)	⊕⊕⊕◯ MODERATE [1,2]	
	374 per 1000	**254 per 1000** (187 to 340)				
	**Low risk population**					
	254 per 1000	**173 per 1000** (127 to 231)				
	**High risk population**					
	320 per 1000	**218 per 1000** (160 to 291)				
Barotrauma	**Study population**		**RR 0.43** (0.20 to 0.90)	395 (2 RCTs)	⊕⊕⊕◯ MODERATE [1,2]	
	105 per 1000	**45 per 1000** (21 to 95)				
	**Moderate risk population**					
	33 per 1000	**14 per 1000** (7 to 30)				
ICU-acquired weakness	**Study population**		**RR 1.13** (0.77 to 1.67)	293 (3 RCTs)	⊕⊕⊕◯ MODERATE [1,2]	
	215 per 1000	**243 per 1000** (165 to 359)				
	**Moderate risk population**					
	63 per 1000	**71 per 1000** (49 to 105)				

***The risk in the intervention group** (and its 95% confidence interval) is based on the assumed risk in the comparison group and the **relative effect** of the intervention (and its 95% CI).

CI: Confidence interval; **RR**: Risk ratio; **OR**: Odds ratio

GRADE Working Group grades of evidence
High quality: We are very confident that the true effect lies close to that of the estimate of the effect
Moderate quality: We are moderately confident in the effect estimate: The true effect is likely to be close to the estimate of the effect, but there is a possibility that it is substantially different
Low quality: Our confidence in the effect estimate is limited: The true effect may be substantially different from the estimate of the effect
Very low quality: We have very little confidence in the effect estimate: The true effect is likely to be substantially different from the estimate of effect

1. Although an insufficient blindness of nurses could be a significant bias, a risk of bias was evaluated as “not serious”.
2. Because cisatracurium is not available in Japan, indirectness was evaluated as “serious”.

Table 1 I. Evidence profile

CQ9: Neuromuscular blocking agents (NMBA) for adult patients with ARDS compared to placebo for adult patients with ARDS

Quality assessment							№ of patients		Effect		Quality	Importance
№ of studies	Study design	Risk of bias	Inconsistency	Indirectness	Imprecision	Other considerations	NMB for ARDS	placebo	Relative (95% CI)	Absolute (95% CI)		
ICU mortality												
3	Randomized trials	Not serious [1]	Not serious	Serious [2]	Not serious	None	70/223 (31.4%)	93/208 (44.7%)	RR 0.70 (0.55 ～ 0.89)	134 fewer / 1000 (49 fewer ～ 201 fewer)	⊕⊕⊕⊖ MODERATE [1,2]	CRITICAL
								31.3%		94 fewer / 1000 (34 fewer ～ 141 fewer)		
								38.9%		117 fewer / 1000 (43 fewer ～ 175 fewer)		
28-day mortality												
2	Randomized trials	Not serious [1]	Not serious	Serious [2]	Not serious	None	52/205 (25.4%)	71/190 (37.4%)	RR 0.68 (0.50 ～ 0.91)	120 fewer / 1000 (34 fewer ～ 187 fewer)	⊕⊕⊕⊖ MODERATE [1,2]	CRITICAL
								25.4%		81 fewer / 1000 (23 fewer ～ 127 fewer)		
								32.0%		102 fewer / 1000 (29 fewer ～ 160 fewer)		
Barotrauma												
2	Randomized trials	Not serious [1]	Not serious	Serious [2]	Not serious	None	9/205 (4.4%)	20/190 (10.5%)	RR 0.43 (0.20 ～ 0.90)	60 fewer / 1000 (11 fewer ～ 84 fewer)	⊕⊕⊕⊖ MODERATE [1,2]	IMPORTANT
								3.3%		19 fewer / 1000 (3 fewer ～ 26 fewer)		
ICU-acquired weakness												
3	Randomized trials	Not serious [1]	Not serious	Serious [2]	Not serious	None	41/158 (25.9%)	29/135 (21.5%)	RR 1.13 (0.77 ～ 1.67)	28 more / 1000 (49 fewer ～ 144 more)	⊕⊕⊕⊖ MODERATE [1,2]	IMPORTANT
								6.3%		8 more / 1000 (14 fewer ～ 42 more)		

RR – relative risk

1. Although the fact that the nurses could not be blinded to the therapy could be a significant bias, the risk of bias was evaluated as "not serious".
2. Since cisatracurium is not available in Japan, indirectness was evaluated as "serious".

Evidence-to-Decision Table

CQ9:Should neuromuscular blocking agents be used in adult patients with ARDS requiring mechanical ventilation?

POPULATION : ADULT PATIENTS WITH MODERATE TO SEVERE ARDS WITHIN 48 HOURS OF ONSET

INTERVENTION : NEUROMUSCULAR BLOCKING AGENT (CISATRACRIUM) FOR 48-HOUR INFUSION

	CRITERIA	JUDGEMENTS	RESEARCH EVIDENCE	ADDITIONAL CONSIDERATION
PROBLEM	**Is the problem a priority?**	○No<br>○Probably no<br>●Probably yes<br>○Yes<br>------------------<br>○Varies<br>○Don't know	Recent studies suggest that treatment modalities preserving spontaneous breathing prevent ICU-acquired weakness and ventilation-perfusion mismatch in patients with ARDS {Girard, 2007 #130}. However, several studies suggest that excessive stress in alveoli due to spontaneous breathing impairs alveolar stability, which may contribute to the poor prognosis in patients with ARDS {Rittayamai, 2015 #131}. The decision to preserve spontaneous breathing or to decrease/prohibit spontaneous breathing by using neuromuscular blockers may have opposite effects on the prognosis in patients with ARDS so the priority of this clinical question is high.	
BENEFITS & HARMS OF THE OPTIONS	**What is the overall certainty of the evidence of effects?**	○Very low<br>○Low<br>●Moderate<br>○High<br>------------------<br>○No included studies	(see research evidence below)	
	**Is there important uncertainty about or variability in how much people value the main outcomes?**	○Important uncertainty or variability<br>○Possibly important uncertainty or variability<br>●Possibly no important uncertainty or variability<br>○No important uncertainty or variability<br>------------------<br>○No known undesirable outcomes		
	**How substantial are the desirable anticipated effects?**	○Trivial<br>○Small<br>●Moderate<br>○Large<br>------------------<br>○Varies<br>○Don't know		
	**How substantial are the undesirable anticipated effects?**	○Large<br>○Moderate<br>●Small<br>○Trivial<br>------------------<br>○Varies<br>○Don't know		
	**Does the balance between desirable effects and undesirable effects favor**	○Favors the comparison<br>○Probably favors the comparison<br>○Does not favor either		Complications associated with the use of neuromuscular blockers include the following three

The relative importance or values of the main outcomes of interest

Outcomes	Relative importance	Quality of the evidence (GRADE)
ICU mortality	CRITICAL	⨁⨁⨁⊖ MODERATE
28-day mortality	CRITICAL	⨁⨁⨁⊖ MODERATE
Barotrauma	IMPORTANT	⨁⨁⨁⊖ MODERATE
ICU-acquired weakness	IMPORTANT	⨁⨁⨁⊖ MODERATE

Summary of findings:

Outcomes	Risk with placebo	Risk with intervention	Absolute effect (95% CI)	Relative effect (95% CI)
ICU mortality	447 / 1000	**313** / **1000** (246 to 398)	134 fewer per 1000 (49 fewer to 201 fewer)	**RR 0.70** (0.55 to 0.89)
	313 / 1000	**219** / **1000** (172 to 279)	94 fewer per 1000 (34 fewer to 141 fewer)	
	389 / 1000	**272** / **1000** (214 to 346)	117 fewer per 1000 (43 fewer to 175 fewer)	
28-day mortality	374 / 1000	**254** / **1000** (187 to 340)	120 fewer per 1000 (34 fewer to 187 fewer)	**RR 0.68** (0.50 to 0.91)
	254 / 1000	**173** / **1000** (127 to 231)	81 fewer per 1000 (23 fewer to 127 fewer)	
	320 / 1000	**218** / **1000** (160 to 291)	102 fewer per 1000 (29 fewer to 160 fewer)	
Barotrauma	105 / 1000	**45** / **1000** (21 to 95)	60 fewer per 1000 (11 fewer to 84 fewer)	**RR 0.43** (0.20 to 0.90)
	33 / 1000	**14** / **1000** (7 to 30)	19 fewer per 1000 (3 fewer to 26 fewer)	
ICU-acquired weakness	215 / 1000	**243** / **1000** (165 to 359)	28 more per 1000 (49 fewer to 144 more)	**RR 1.13** (0.77 to 1.67)
	63 / 1000	**71** / **1000** (49 to 105)	8 more per 1000 (14 fewer to 42 more)	

CQ9: **Should neuromuscular blocking agents be used in adult patients with ARDS requiring mechanical ventilation?**				
POPULATION: ADULT PATIENTS WITH MODERATE TO SEVERE ARDS WITHIN 48 HOURS OF ONSET				
INTERVENTION: NEUROMUSCULAR BLOCKING AGENT (CISATRACRIUM) FOR 48-HOUR INFUSION				
	CRITERIA	JUDGEMENTS	RESEARCH EVIDENCE	ADDITIONAL CONSIDERATION
	**the option or the comparison?**	the intervention or the comparison<br>● Probably favors the intervention<br>○ Favors the intervention<br>------------------<br>○ Varies<br>○ Don't know	**Summary:** The use of neuromuscular blockers reduces the risk of ICU mortality, 28-day mortality and barotrauma. However, no correlation was observed between the use of neuromuscular blockers and the development of ICU-acquired weakness.	classifications: polyneuropathy, polymyopathy and neuromyopathy. In addition, DVT, corneal injury and anaphylaxy may also occur.
RESOURCE USE	**How large are the resource requirements (costs)?**	○ Large costs<br>● Moderate costs<br>○ Negligible costs and savings<br>○ Moderate savings<br>○ Large savings<br>------------------<br>○ Varies<br>○ Don't know	The medication is continuously delivered via peripheral vein. The required amount of materials is limited and the daily cost of medication is projected to be in the range of a few thousand yen.	
RESOURCE USE	**Does the cost effectiveness of the option favor the option or the comparison?**	○ Favors the comparison<br>○ Probably favors the comparison<br>○ Does not favor either the intervention or the comparison<br>● Probably favors the intervention<br>○ Favors the intervention<br>------------------<br>○ Varies<br>○ No included studies	The costs incurred for purchase of required materials and medications are roughly equivalent to that of similar medications (i.e. sedatives).	
EQUITY	**What would be the impact on health equity?**	○ Reduced<br>● Probably reduced<br>○ Probably no impact<br>○ Probably increased<br>○ Increased<br>------------------<br>○ Varies<br>○ Don't know	The use of the medication in question does not require special medical facilities/equipment and therefore its overall influence upon patient equality is expected to be universally negligible. However, cisatracrium is not available in Japan and therefore this must be taken into consideration.	

CQ9: **Should neuromuscular blocking agents be used in adult patients with ARDS requiring mechanical ventilation?**				
POPULATION: ADULT PATIENTS WITH MODERATE TO SEVERE ARDS WITHIN 48 HOURS OF ONSET				
INTERVENTION: NEUROMUSCULAR BLOCKING AGENT (CISATRACRIUM) FOR 48-HOUR INFUSION				
	CRITERIA	JUDGEMENTS	RESEARCH EVIDENCE	ADDITIONAL CONSIDERATIONS
ACCEPTABILITY	**Is the option acceptable to key stakeholders?**	○No<br>○Probably no<br>●Probably yes<br>○Yes<br>------------------<br>○Varies<br>○Don't know	There are no apparent disadvantages to the primary stakeholder and therefore the present option can be expected to be readily accepted.	
FEASIBILITY	**Is the option feasible to implement?**	○No<br>○Probably no<br>●Probably yes<br>○Yes<br>------------------<br>○Varies<br>○Don't know	The use of the medication in question does not require special medical facilities/equipment and is therefore appropriate for practical use.	

Recommendation

CQ9：Should neuromuscular blocking agents be used in adult patients with ARDS requiring mechanical ventilation?

**Balance of consequences**	Undesirable consequences *clearly outweigh* desirable consequences in most settings	Undesirable consequences *probably outweigh* desirable consequences in most settings	The balance between desirable and undesirable consequences is closely *balanced or uncertain*	Desirable consequences *probably outweigh* undesirable consequences in most settings	Desirable consequences *clearly outweigh* undesirable consequences in most settings
Judgement	○	○	○	●	○

**Type of recommendation**	Strong recommendation against the intervention	Conditional recommendation against the intervention	Conditional recommendation for either the intervention or the comparison	Conditional recommendation for the intervention
Judgement	○	○	●	○

Recommendation

We suggest the use of neuromuscular blocking agents (NMBAs) in adult patients with ARDS requiring mechanical ventilation, under certain circumstances. (GRADE 2B, Strength of recommendation "weak recommendation" / Quality of evidence "moderate")

Supplementary conditions: The routine use of NMBAs should be avoided. Their use would be justified only if the Berlin definition of ARDS is fulfilled for patients with moderate or severe ARDS (P/F</=200 on PEEP of >/=5cmH_2O). We would also limit their use to less than 48 hours in the early phase of the disease. The NMBAs currently available in Japan have some risks for causing myopathy. In particular, the concurrent use of steroids increases the risk, which should be taken into account {Adnet, 2001 #132;Behbehani, 1999 #133;Leatherman, 1996 #134}**.** NMBAs are generally categorized into depolarizing agents and non-depolarizing agents based on their pharmacologic mechanism. Compared to non-depolarizing agents, depolarizing agents have more side effects such as myalgia, hyperkalemia, and elevated intracranial pressure. Therefore, non-depolarizing agents are preferable in clinical practice. Non-depolarizing NMBAs are further classified into aminosteroids (Rocuronium, Vecuronium, Pancuronium) and benzylisoquinolines (Atracurium, Cisatracurium, Mivacurium) on the basis of their chemical structure. Cisatracurium, which was used in all three RCTs analyzed in this systematic review, is not available in Japan. Rocuronium or vecuronium are alternatives. However, special consideration is required. While the metabolism of benzylisoquinolines such as cisatricurium is not influenced by hepatic or renal function, the metabolism of aminosteroids such as rocuronium or vecuronium is delayed in patients with hepatic or renal dysfunction. In addition, attention needs to be paid to the risk of muscular atrophy due to aminosteroid use. There was a suggestion given by one of the panelists that the routine use of NMBAs should not be recommended because NMBAs currently available in Japan may increase the risk of myopathy. After extensive discussion among the panelists, agreement was reached to make a weak recommendation for their use under certain circumstances, as described in the comments.

Justification

Clinical question: Should neuromuscular blocking agents be used in adult patients with ARDS requiring mechanical ventilation?
Patient or population: Adult patients with moderate to severe ARDS within 48 hours of onset
Intervention: Neuromuscular blocking agent (Cisatracrium) for 48-hour infusion
Comparison: Placebo
Outcomes: ICU mortality, 28-day mortality, barotrauma, ICU-acquired weakness

Summary of the evidence: All three RCTs analyzed in this systematic review were conducted by the same French group which studied the efficacy of NMBAs in adult patients with ARDS requiring mechanical ventilation {Forel, 2006 #127;Gainnier, 2004 #128;Papazian, 2010 #129}. All cohorts fulfilled the criterion of having moderate or severe ARDS (P/F</=200 on PEEP of >/=5cmH_2O) based on the Berlin definition. NMBA use was limited to less than 48 hours from the onset of the disease. Meta-analysis of these 3 RCTs (total 431 patients) demonstrated that the ICU mortality, 28-day mortality, and the rate of barotrauma are significantly lower in the NMBA group compared to the control group (ICU mortality: RR 0.70, 95%CI 0.55-0.89; 28-day mortality: RR 0.68, 95%CI 0.50-0.91; the rate of barotrauma: RR 0.43. 95% CI 0.20-0.90). There is no statistically significant difference between the two groups regarding the occurrence of myopathy due to NMBA use.

	**Quality of the evidence:** All three RCTs demonstrated that the NMBA-treated groups had a consistent, significant improvement in mortality compared to control groups {Forel, 2006 #127;Gainnier, 2004 #128;Papazian, 2010 #129}. The statistical significance was also confirmed by meta-analysis (I^2=0% in all outcomes). Although complete concealment of the study drug was not possible due to its pharmacologic characteristics, the possibility of other risk of biases was considered to be low. There was no major issue in selection of the study population or outcome measurement. However, the level of recommendation was downgraded, because cisatracurium, used in these three RCTs, is currently not available in Japan, and as a result, indirectness of these studies is considered serious. The ICU mortality and 28-day mortality were 163/431 (38%) and 123/395 (31%), respectively, and the number of events was considered sufficient to provide precise effect estimates. We need a special caution here for the following reasons before interpreting the results. First, all three RCTs analyzed in this meta-analysis were conducted by the same French study group. Second, the Papazian 2010 study enrolled a much larger cohort compared to the other studies {Papazian, 2010 #129}. As a result, this study might have a disproportionate impact on the results. The number of patients with barotrauma and myopathy was either quite low or not assessed in the other two RCTs. Therefore, when all three RCTs are compared to the Papazian study alone, the outcomes are similar.<br><br>**Judgement of benefit and harm, resources and cost:** Since a certain degree of benefit is expected with NMBAs, use without serious complications, treatment with NMBAs will be accepted by most patients. However, we recognize that cisatracurium, the drug used in the RCTs, is not available in Japan.<br><br>**Recommendations:** We suggest the use of neuromuscular blocking agents (NMBAs) in adult patients with ARDS requiring mechanical ventilation, under certain circumstances. (GRADE 2B, Strength of recommendation “weak recommendation” / Quality of evidence “moderate”)<br><br>**Additional considerations:** NMBAs are generally categorized into depolarizing agents and non-depolarizing agents based on their pharmacologic mechanism. Compared to non-depolarizing agents, depolarizing agents have more side effects such as myalgia, hyperkalemia, and elevated intracranial pressure. Therefore, non-depolarizing agents are preferable in clinical practice. Non-depolarizing NMBAs are further classified into aminosteroids (Rocuronium, Vecuronium, Pancuronium) and benzylisoquinolines (Atracurium, Cisatracurium, Mivacurium) on the basis of their chemical structure. Cisatracurium, which was used in all three RCTs analyzed in this systematic review, is not available in Japan. Rocuronium or vecuronium are alternatives. However, special consideration is required. While the metabolism of benzylisoquinolines such as cisatricurium is not influenced by hepatic or renal function, the metabolism of aminosteroids such as rocuronium or vecuronium is delayed in patients with hepatic or renal dysfunction. In addition, attention needs to be paid to the risk of muscular atrophy due to aminosteroid use. There was a suggestion given by one of the panelists that the routine use of NMBAs should not be recommended because NMBAs currently available in Japan may increase the risk of myopathy. After extensive discussion among the panelists, agreement was reached to make a weak recommendation for their use under certain circumstances, as described in the comments.
**Subgroup considerations**	Accoridng to the severity of ARDS in the Berlin definition, recommendation for the efficacy of NMBAs may be changed.
**Implementation considerations**	Cisatracurium, which was used in all three RCTs analyzed in this systematic review, is not available in Japan. Adoption of cisatricurium in Japan is expected in the near future.
**Monitoring and evaluation considerations**	Respiratory and circulatory monitoring, neuromuscular monitoring with train-of-four (TOF) stimulation, and sedative monitoring (BIS®: Bispectral Index) are necessary to evaluate the adequacy of neuromuscular blockade.
**Research possibilities**	For patients who fulfill the Berlin definition for mild ARDS, the safety and efficacy of cisatracurium, as well as vecuronium, pancuronium, and rocuronium need to be assessed in further clinical trials.

References

CQ10. Study flow diagram

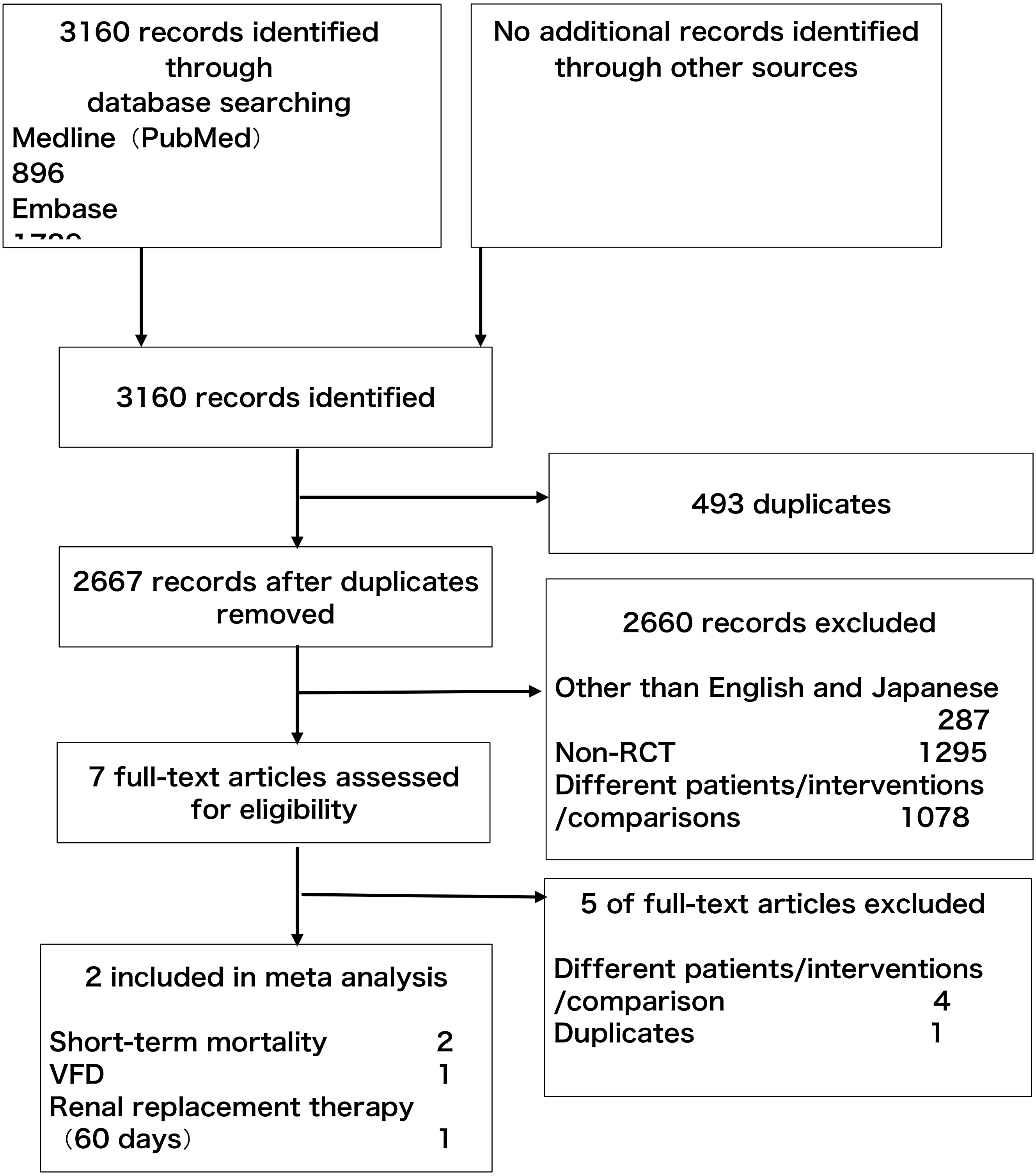

CQ10
Risk of bias table, mortality

Outcome		Short term mortality		risk of bias		not serious (0)			
番号	著者名 発表年（Forest plot表示）	risk of bias評価							
		ランダム割付順番の生成 random sequence generation	割り付けの隠蔽化 allocation concealment	ブラインド blinding		不完全なアウトカムデータ incomplete outcome data	選択されたアウトカムの報告 selective outcome reporting	その他のバイアス Other sources of bias	研究内でのバイアスのリスク Risk of bias within a study
				研究参加者と治療提供者 participants and personnel	アウトカム評価者 outcome assessors				
1	FACTT 2006	Low risk	Low risk	High risk	Low risk	Low risk	Low risk	Low risk	Low risk
2	Martin 2002	Low risk	Low risk	Low risk	Low risk	Low risk	Unclear risk	Low risk	Low risk
番号	著者名 発表年（Forest plot表示）	risk of biasコメント							
1	FACTT 2006	自動システムを用いたランダム化を行った	自動システムを用いて8個のブロックに分けているため、隠蔽化できていると判断した	ブラインド化できていない	ブラインド化できていないが,死亡の判定には影響なし	比較群で1例のみ解析から除外された	100%報告された	研究の中断はなく、他のバイアスも指摘できなかった	Low riskの項目が多いため
2	Martin 2002	コンピューターを用いてランダム化している	各施設に割り付けとブラインド化を担当する薬剤師がおり、隠蔽化されていると判断した	上記薬剤師により治験薬とプラセボが管理されており、ブラインド化されていると判断した	上記によりブラインド化できていると考えられる上に、ブラインド化の有無で結果に影響なし	100%報告された	事前に計画されたプロトコールが閲覧できなかった	研究の中断なし	Low riskの項目が多いため

CQ10

Risk of bias table VFD

Outcome		VFD		risk of bias		not serious (0)			
番号	著者名 発表年（Forest plot表示）	risk of bias評価							
		ランダム割付順番の生成 random sequence generation	割り付けの隠蔽化 allocation concealment	ブラインド blinding 研究参加者と治療提供者 participants and personnel	ブラインド blinding アウトカム評価者 outcome assessors	不完全なアウトカムデータ incomplete outcome data	選択されたアウトカムの報告 selective outcome reporting	その他のバイアス Other sources of bias	研究内でのバイアスのリスク Risk of bias within a study
1	FACTT 2006	Low risk	Low risk	High risk	Unclear risk	Low risk	Low risk	Low risk	Low risk

番号	著者名 発表年（Forest plot表示）	risk of biasコメント							
1	FACTT 2006	自動システムを用いたランダム化を行った	自動システムを用いて8個のブロックに分けているため、隠蔽化できていると判断した	ブラインド化できていない	ブラインド化できていないが、CVP測定は呼気終末に仰臥位で行っていたため、アウトカム評価者がどちらの群か容易に分からないと判断しUnclearとした	比較群で1例のみ解析から除外された	100%報告された	研究の中断はなく、他のバイアスも指摘できなかった	Low riskの項目が多いため

CQ10
Risk of bias table, Renal replacement therapy

Outcome		Renal replacement therapy		risk of bias		not serious (0)			
番号	著者名 発表年（Forest plot表示）	risk of bias評価							
		ランダム割付順番の生成 random sequence generation	割り付けの隠蔽化 allocation concealment	ブラインド blinding		不完全なアウトカムデータ incomplete outcome data	選択されたアウトカムの報告 selective outcome reporting	その他のバイアス Other sources of bias	研究内でのバイアスのリスク Risk of bias within a study
				研究参加者と治療提供者 participants and personnel	アウトカム評価者 outcome assessors				
1	FACTT 2006	Low risk	Low risk	High risk	Unclear risk	Low risk	Low risk	Low risk	Low risk
番号	著者名 発表年（Forest plot表示）	risk of biasコメント							
1	FACTT 2006	自動システムを用いたランダム化を行った	自動システムを用いて8個のブロックに分けているため、隠蔽化できていると判断した	ブラインド化できていない	ブラインド化できていないが、CVP測定は呼気終末に仰臥位で行っていたため、アウトカム評価者がどちらの群か容易に分からないと判断しUnclearとした	比較群で1例のみ解析から除外された	100%報告された	研究の中断はなく、他のバイアスも指摘できなかった	Low riskの項目が多いため

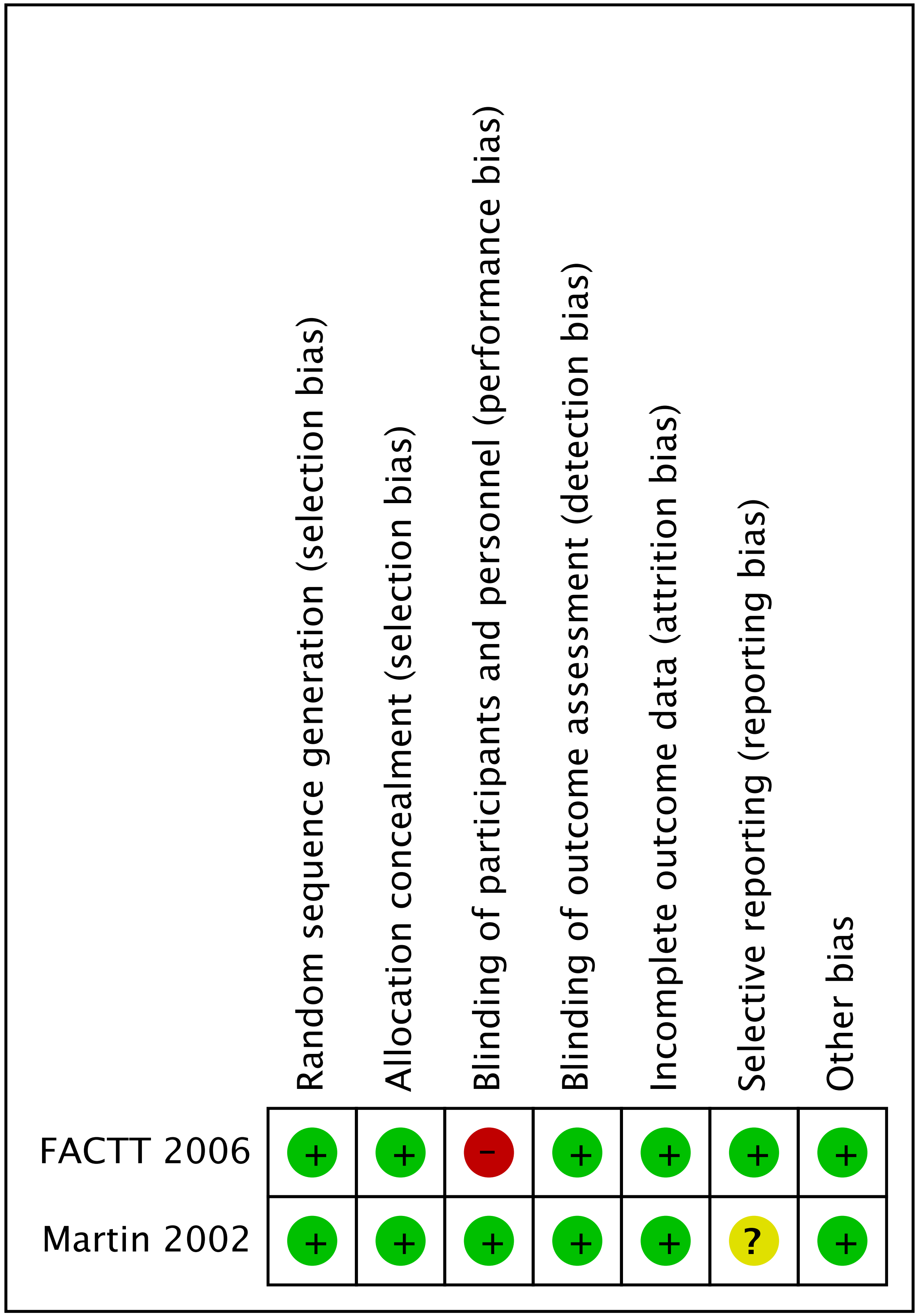

Short term mortality

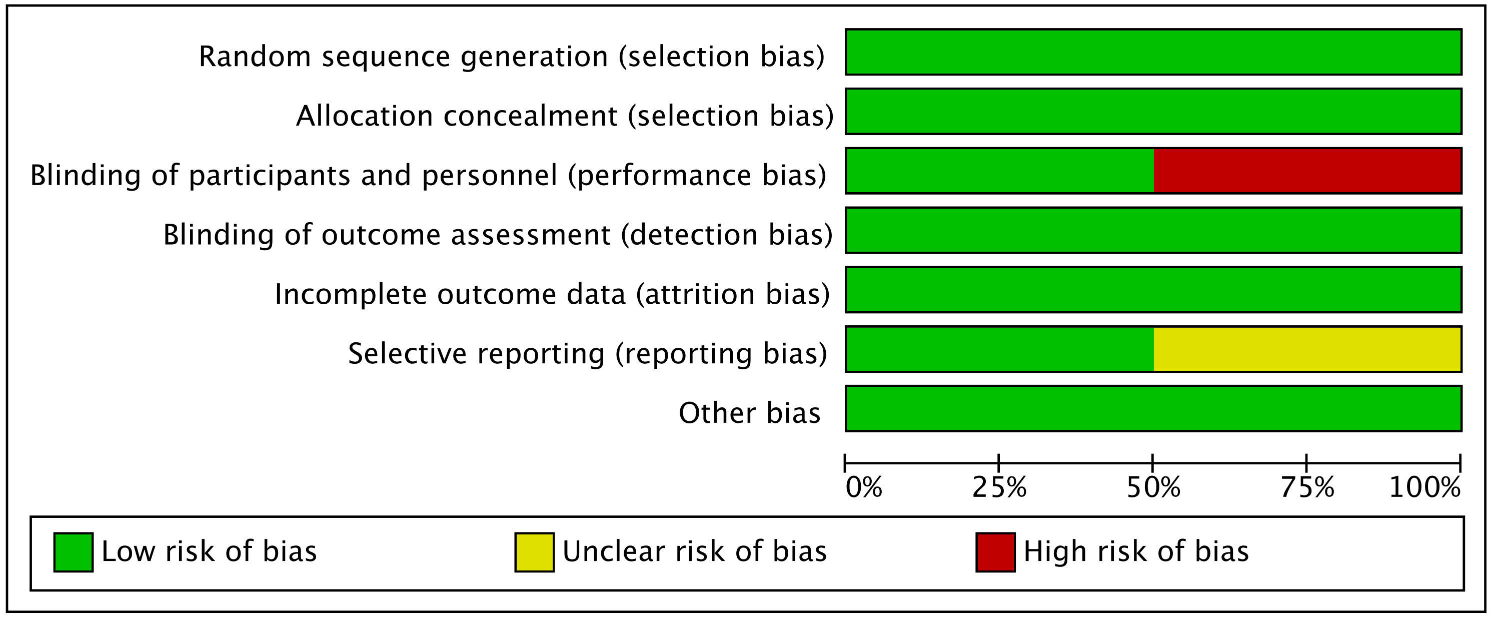

CQ10 Risk of bias summary, Risk of bias graph

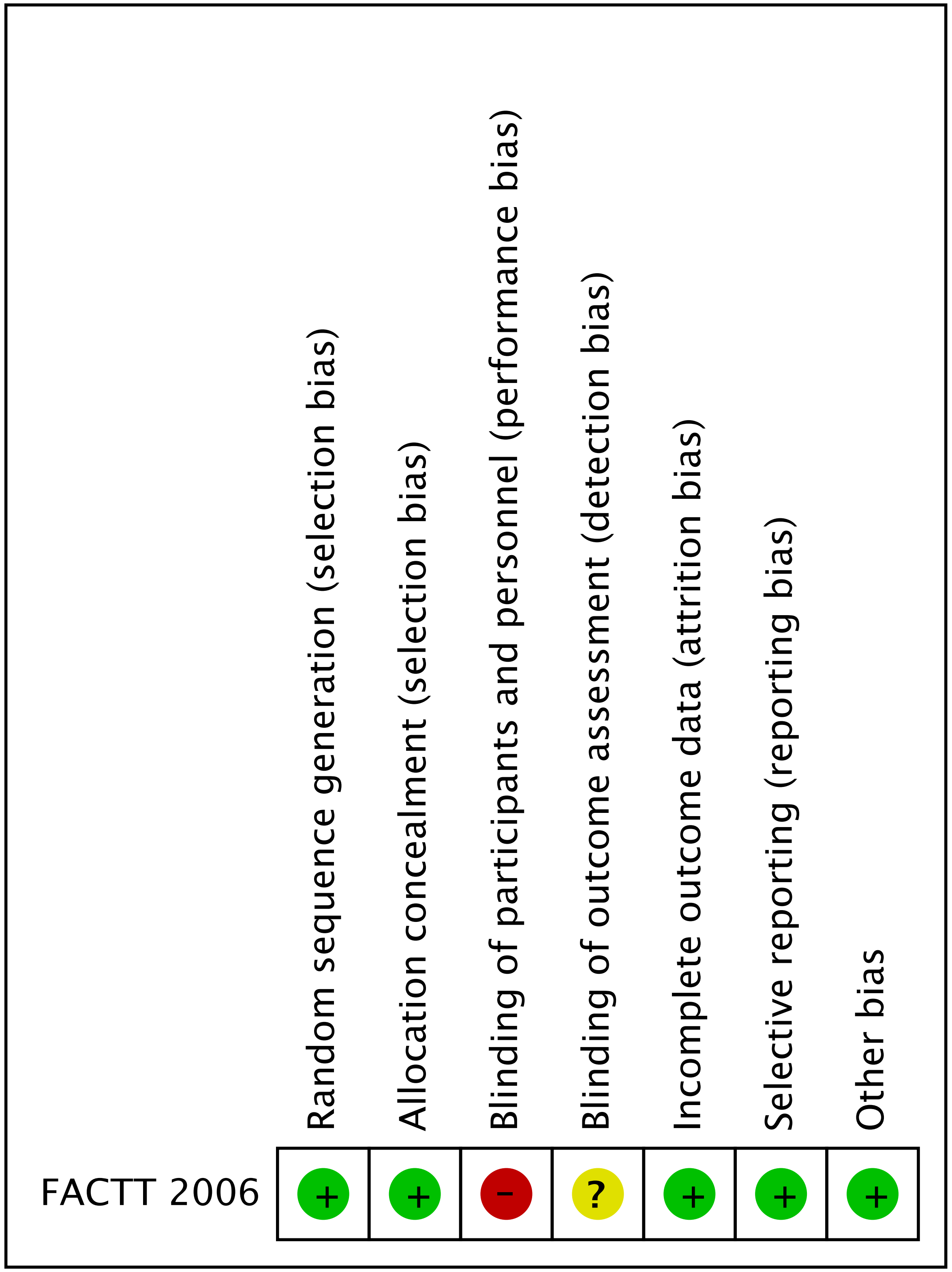

VFD

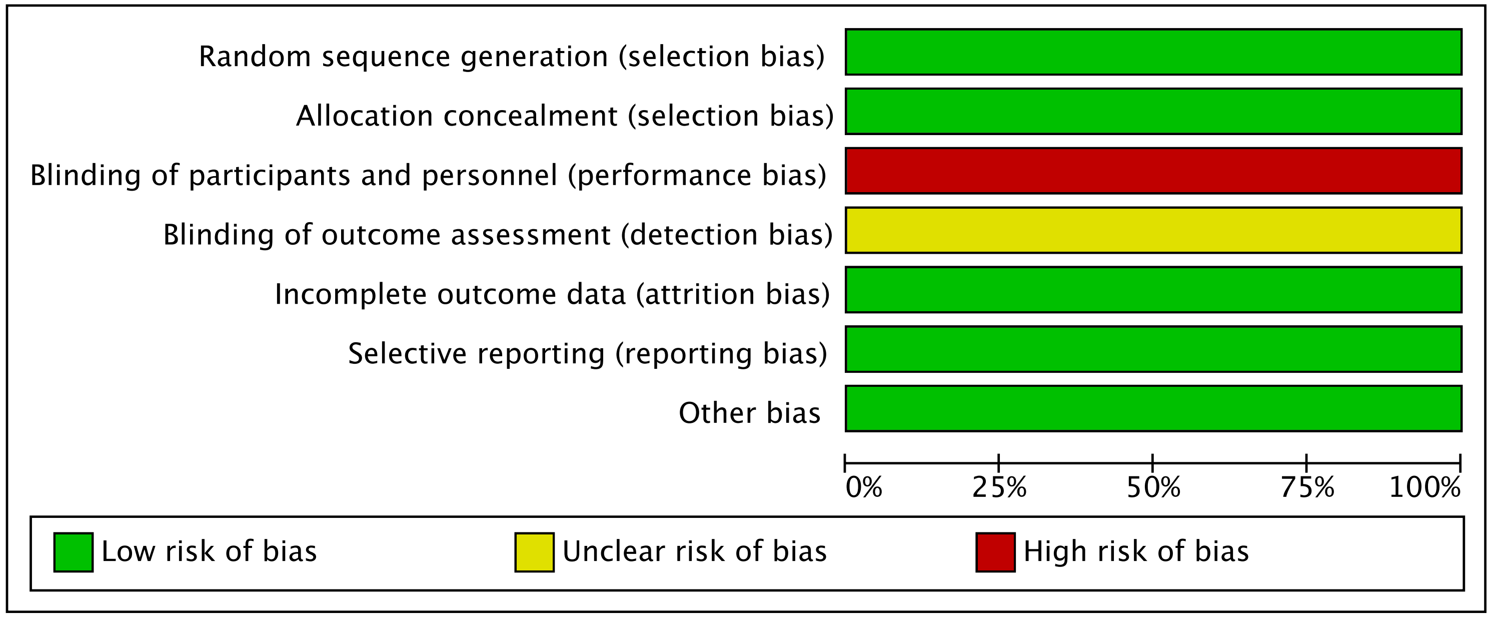

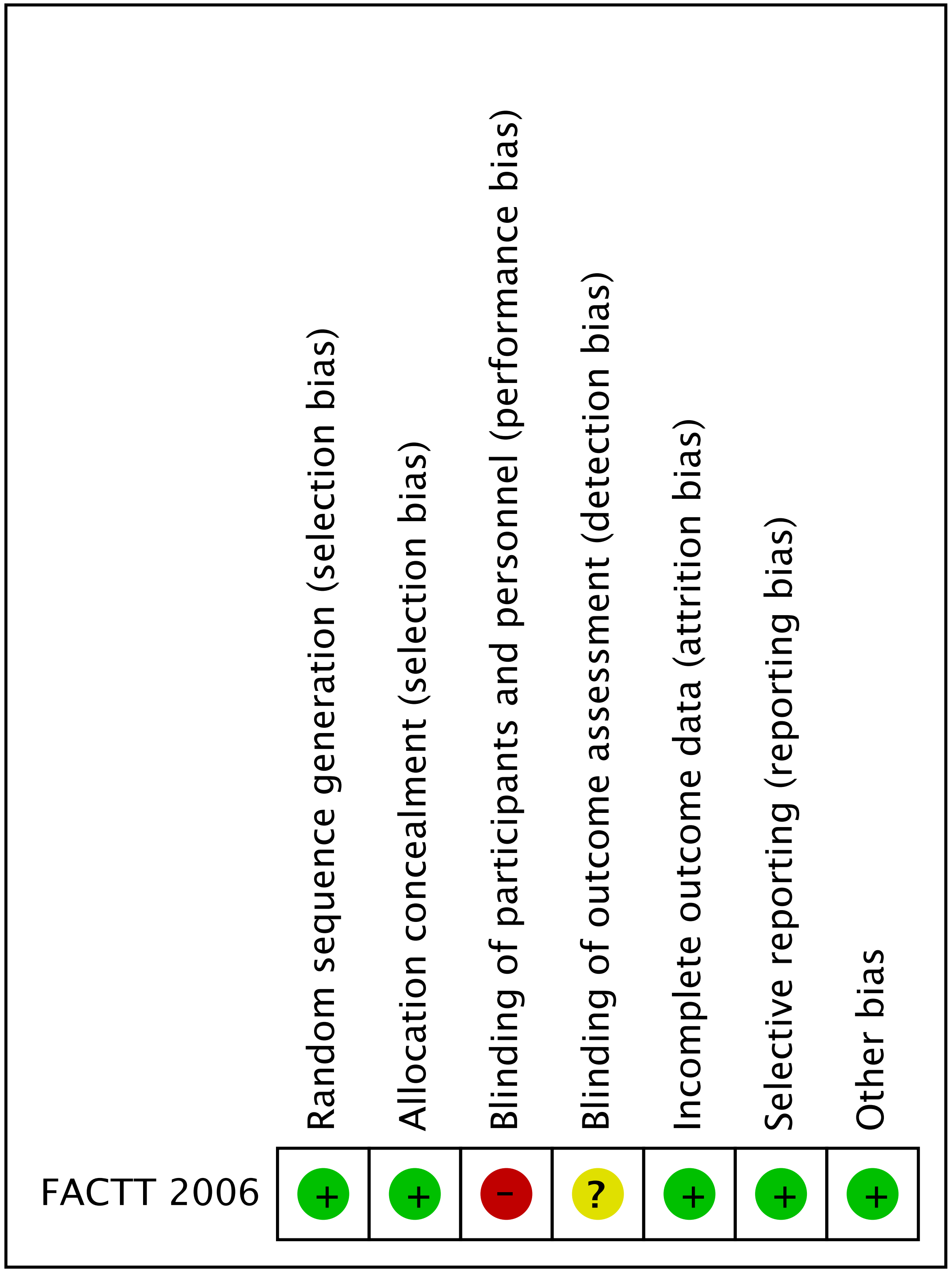

Renal replacemnt Therapy

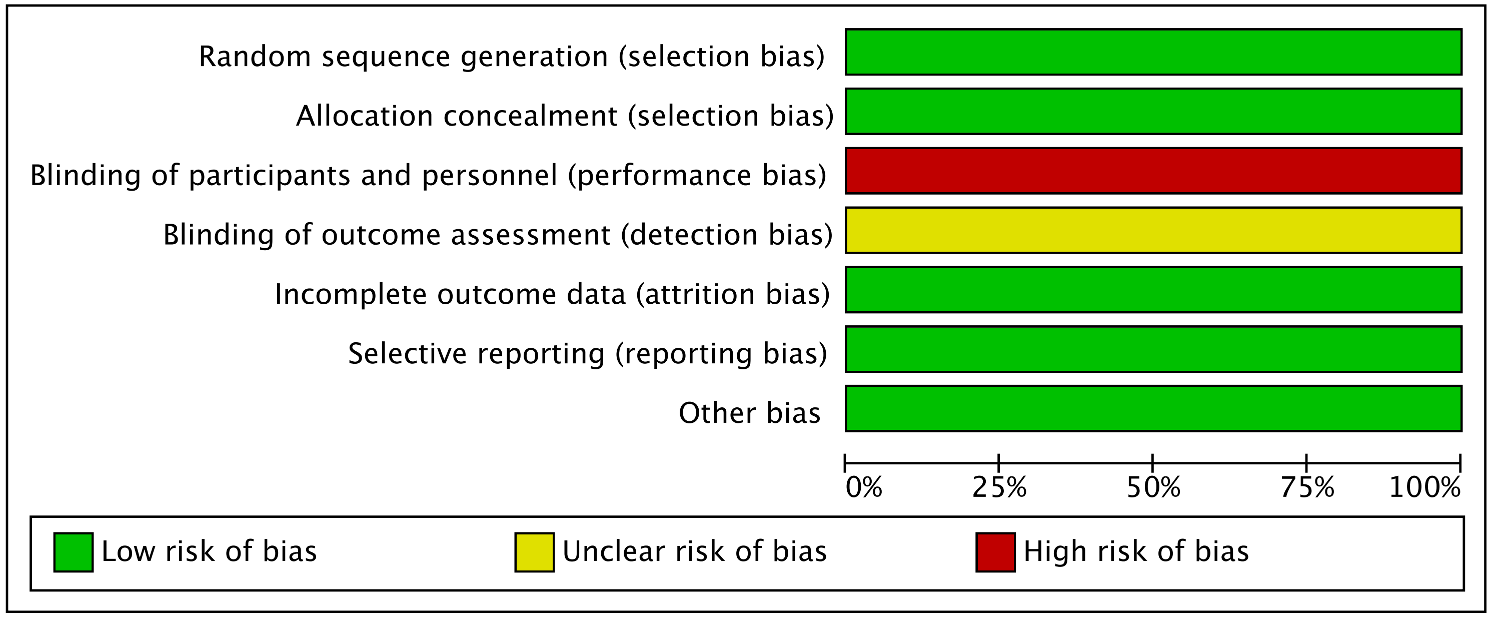

Short term mortality

Study or Subgroup	Fluid conservative Events	Fluid conservative Total	Fluid liberal Events	Fluid liberal Total	Weight	Risk Ratio M-H, Random, 95% CI
FACTT 2006	128	503	141	497	98.1%	0.90 [0.73, 1.10]
Martin 2002	3	19	3	18	1.9%	0.95 [0.22, 4.10]
**Total (95% CI)**		**522**		**515**	**100.0%**	**0.90 [0.73, 1.10]**
Total events	131		144			

Heterogeneity: $Tau^2 = 0.00$; $Chi^2 = 0.01$, df = 1 (P = 0.94); $I^2 = 0\%$
Test for overall effect: Z = 1.04 (P = 0.30)

Risk Ratio M-H, Random, 95% CI

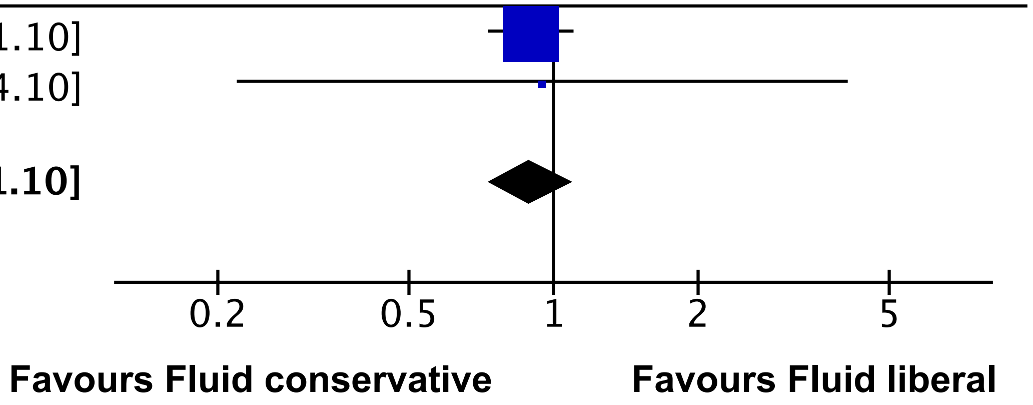

VFD

Study or Subgroup	Fluid conservative Mean	Fluid conservative SD	Fluid conservative Total	Fluid liberal Mean	Fluid liberal SD	Fluid liberal Total	Weight	Mean Difference IV, Random, 95% CI
FACTT 2006	14.6	11.2	503	12.1	11.1	497	100.0%	2.50 [1.12, 3.88]
**Total (95% CI)**			**503**			**497**	**100.0%**	**2.50 [1.12, 3.88]**

Heterogeneity: Not applicable
Test for overall effect: Z = 3.55 (P = 0.0004)

Mean Difference IV, Random, 95% CI

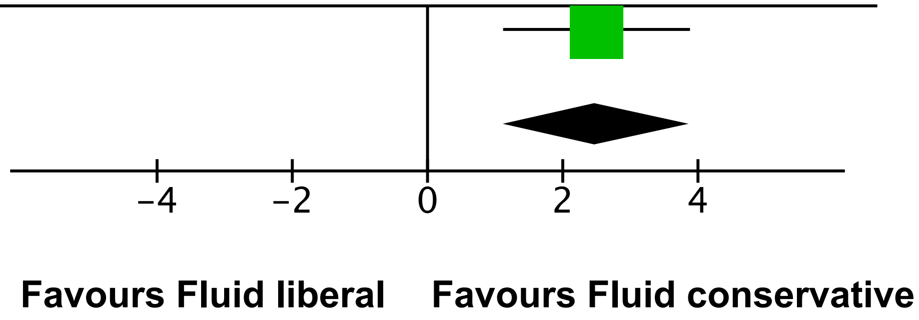

Renal replacemnt Therapy

Study or Subgroup	Fluid conservative Events	Fluid conservative Total	Fluid liberal Events	Fluid liberal Total	Weight	Risk Ratio M-H, Random, 95% CI
FACTT 2006	51	503	70	497	100.0%	0.72 [0.51, 1.01]
**Total (95% CI)**		**503**		**497**	**100.0%**	**0.72 [0.51, 1.01]**
Total events	51		70			

Heterogeneity: Not applicable
Test for overall effect: Z = 1.90 (P = 0.06)

Risk Ratio M-H, Random, 95% CI

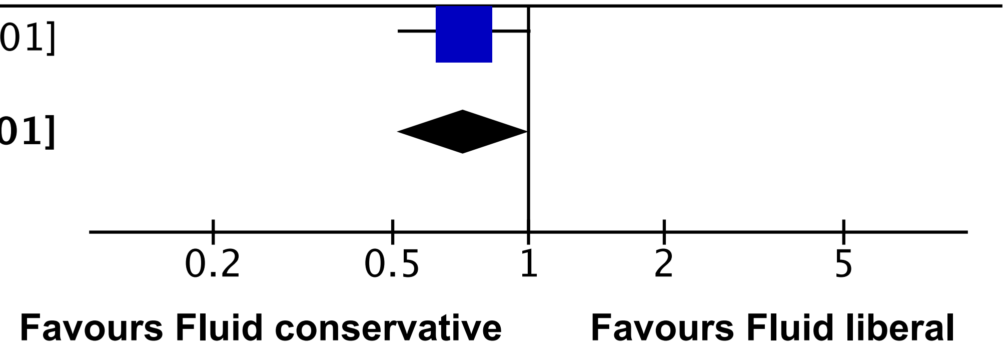

CQ10 Summary of findings:

Conservative strategy compared to liberal strategy for adult ARDS

Patients or population: adult ARDS
Intervention: conservative strategy
Comparison: liberal strategy

Outcomes	Anticipated absolute effects (95% CI)		Relative effect (95% CI)	No of participants (studies)	Quality of the evidence (GRADE)	Comments
	Risk with liberal strategy	Risk with conservative strategy				
Short-term (<90d) mortality	Study population		RR 0.90 (0.73 to 1.10)	1037 (2 RCTs)	⊕⊕⊕◯ MODERATE [1]	
	280 / 1000	**252 / 1000** (204 to 308)				
	Low risk patients					
	250 / 1000	**225 / 1000** (183 to 275)				
	High risk patients					
	450 / 1000	**405 / 1000** (329 to 495)				
VFD	Mean **12.1** days	2.5 days more MD (1.12 more to 3.88 more)	-	1000 (1 RCT)	⊕⊕⊕⊕ HIGH	
Renal replacement therapy (60 days)	Study population		RR 0.72 (0.51 to 1.01)	1000 (1 RCT)	⊕⊕⊕◯ MODERATE [1]	
	141 / 1000	**101 / 1000** (72 to 142)				
	Moderate risk patients					
	150 / 1000	**108 / 1000** (77 to 152)				

***The risk in the intervention group** (and its 95% confidence interval) is based on the assumed risk in the comparison group and the **relative effect** of the intervention (and its 95% CI).

CI: Confidence interval; **RR:** Risk ratio; **MD:** Mean difference

GRADE Working Group grades of evidence
High quality: We are very confident that the true effect lies close to that of the estimate of the effect
Moderate quality: We are moderately confident in the effect estimate: The true effect is likely to be close to the estimate of the effect, but there is a possibility that it is substantially different
Low quality: Our confidence in the effect estimate is limited: The true effect may be substantially different from the estimate of the effect
Very low quality: We have very little confidence in the effect estimate: The true effect is likely to be substantially different from the estimate of effect

1. Wide 95%CI due to small case number

CQ10
Question: How should fluid balance be maintained on a daily basis in adult patients with ARDS: Liberal vs. Conservative strategy?

Quality assessment							№ of patients		Effect		Quality	Importance
**№ of studies**	**Study design**	**Risk of bias**	**Inconsistency**	**Indirectness**	**Imprecision**	**Other considerations**	**Conservative strategy**	**Liberal strategy**	Relative **(95% CI)**	**Absolute (95% CI)**		
Short-term Mortality [Note 1)]												
2	Randomised trials	Not serious	Not serious	Not serious	Serious[1]	None	131/522 (25.1%)	144/515 (28.0%)	**RR 0.90** (0.73 to 1.10)	28 fewer / 1000 (from 28 more to 75 fewer)	⊕⊕⊕⊖ MODERATE[1]	CRITICAL
								25.0%		25 fewer / 1000 (from 25 more to 68 fewer)		
								45.0%		45 fewer / 1000 (from45 more to 122 fewer)		
VFD												
1	Randomised trials	Not serious	Not serious	Not serious	Not serious	None	503	497	-	MD 2.5 days more From (1.12 more to 3.88 more)	⊕⊕⊕⊕ HIGH	CRITICAL
Renal Replacement Therapy [Note 2)]												
1	Randomised trials	Not serious	Not serious	Not serious	Serious[1]	None	51/503 (10.1%)	70/497 (14.1%)	**RR 0.72** (0.51 to 1.01)	39 fewer / 1000 (from 1 more to 69 fewer)	⊕⊕⊕⊖ MODERATE[1]	IMPORTANT
								15.0%		42 fewer / 1000 (from 2 more to 74 fewer)		

CI: Confidence interval; **RR:** Risk ratio; **MD:** Mean difference

1. Wide 95%CI due to small number of patients

Evidence-to-Decision table

CQ10：How should fluid balance be maintained on a daily basis in adult patients with ARDS?				
POPULATION：ADULT PATIENTS WITH ARDS				
INTERVENTION：FLUID CONSERVATIVE STRATEGY				
	CRITERIA	JUDGEMENTS	RESERCH EVIDENCE	ADDITIONAL CONSIDERRATION
PROBLEM	**Is the problem a priority?**	○No<br>○Probably no<br>●Probably yes<br>○Yes<br>------------------<br>○Varies<br>○Don't know	In patients with ARDS, pulmonary edema is caused by vascular endothelial dysfunction or increased vascular permeability(3). A positive fluid balance in patients with ARDS increases the mortality rate (4). Extravascular lung water content is associated with disease severity and mortality rate (5).<br>However, there is no previous RCT that reported improvement in mortality rate by changing the fluid management in patients with ARDS. It has not been established how fluid balance is maintained in patients with ARDS despite the fact that optimally reducing fluid volume is well known and remains a goal in daily clinical practice. Therefore, the priority of this issue is considered to be high.	
BENEFITS & HARMS OF THE OPTIONS	**What is the overall certainty of the evidence of effects?**	○Very low<br>○Low<br>●Moderate<br>○High<br>------------------<br>○No included studies	See research evidence below	
	**Is there important uncertainty about or variability in how much people value the main outcomes?**	○Important uncertainty or variability<br>●Possibly important uncertainty or variability<br>○Possibly no important uncertainty or variability<br>○No important uncertainty or variability<br>------------------<br>○No known undesirable outcomes		
	**How substantial are the desirable anticipated effects?**	○Trivial<br>○Small<br>○Moderate<br>○Large<br>------------------<br>●Varies<br>○Don't know		
	**How substantial are the undesirable anticipated effects?**	○Large<br>●Moderate<br>○Small<br>○Trivial<br>------------------<br>○Varies<br>○Don't know		
	**Does the balance between desirable effects and undesirable effects favour the option or the comparison?**	○Favors the comparison<br>○Probably favors the comparison<br>○Does not favor either the intervention or the comparison<br>●Probably favors the intervention		

The relative importance or values of the main outcomes of interest:

Outcome	Relative importance	Certainty of the evidence (GRADE)
Short term mortality Note 1)	CRITICAL	⊕⊕⊕⊖<br>MODERATE
VFD Note 2)	CRITICAL	⊕⊕⊕⊕<br>HIGH
Renal Replacement Therapy Note 3)	IMPORTANT	⊕⊕⊕⊖<br>MODERATE

Summary of findings:

Outcome	Liberal	Conservative	Absolute effect (95% CI)	Relative effect (RR) (95% CI)
Short term mortality Note 1)	280 / 1000	252 / 1000 (204 to 308)	28 fewer per 1000 (from 28 more to 75fewer)	RR 0.90 (0.73 to 1.10)
	250 / 1000	225 / 1000 (183 to 275)	25 fewer per 1000 (from 25 more to 68 fewer)	
	450 / 1000	405 / 1000 (329 to 495)	45 fewer per 1000 (from 45 more to 122 fewer)	
VFD Note 2)	Average 12.1 days	Average 14.6 days	MD 2.5more (1.12 fewer to 3.38 more)	-
Renal Replacement Therapy Note 3)	141 / 1000	101 / 1000 (72 to 142)	39 fewer per 1000 (from 1 more to 69 fewer)	RR 0.72 (0.51 to 1.01)
	150 / 1000	**108 / 1000** (77 to 152)	42 fewer per 1000 (from 27 more to 74 fewer)	

Summary：There was no significant difference in short-term mortality or the need for renal replacement therapy. VFD in patients treated with a conservative strategy was greater than those treated with a liberal strategy (14.6 days vs. 12.1 days).

Renal failure free days also were not different between the two groups in FACTT 2006. In a post hoc analysis of this trial, more patients developed AKI by first the 2 days with a conservative strategy (11), however, after adjustment for fluid balance, the incidence of AKI was greater in patients treated with a liberal strategy (12). Both hypovolemia and congestion are important to maintain organ perfusion. Fluid restriction is not always associated with organ failure.

		○Favors the intervention<br>------------------<br>○Varies<br>○Don't know		
RESOURCEUSE	**How large are the resource requirements (costs)?**	○Large costs<br>○Moderate costs<br>○Negligible costs and savings<br>●Moderate savings<br>○Large savings<br>------------------<br>○Varies<br>○Don't know	PAOP or CVP was used to evaluate fluid status in FACTT 2006, but various methods were used in other trials. If we evaluate fluid status in some way, we don't need special resources.	
	**Does the cost effectiveness of the option favour the option or the comparison?**	○Favors the comparison<br>○Probably favors the comparison<br>○Does not favor either the intervention or the comparison<br>●Probably favors the intervention<br>○Favors the intervention<br>------------------<br>○Varies<br>○No included studies	The dose of furosemide increased to 600mg in patients treated with a conservative strategy during the 7day intervention period.<br>The cost of furosemide 20mg is 60 JPY. If 600mg furosemide is used additionally, it costs 1800 JPY more. But, it is considered to be effective, because VFD increases 2.5 days.	
EQUITY	**What would be the impact on health equity?**	○Reduced<br>●Probably reduced<br>○Probably no impact<br>○Probably increased<br>○Increased<br>------------------<br>○Varies<br>○Don't know	We can perform fluid restriction in routine practice.	
ACCEPTABILITY	**Is the option acceptable to key stakeholders?**	○No<br>○Probably no<br>●Probably yes<br>○Yes<br>------------------<br>○Varies<br>○Don't know	It might be acceptable, because fluid restriction is a common strategy.	
FEASIBILITY	**Is the option feasible to implement?**	○No<br>○Probably no<br>○Probably yes<br>●Yes<br>------------------<br>○Varies<br>○Don't know	We can perform fluid restriction in routine practice.	

Recommendation

CQ10：How should fluid balance be maintained on a daily basis in adult patients with ARDS?					
**Balance of consequences**	Undesirable consequences *clearly outweigh* desirable consequences in most settings	Undesirable consequences *probably outweigh* desirable consequences in most settings	The balance between desirable and undesirable consequences is *closely balanced or uncertain*	Desirable consequences *probably outweigh* undesirable consequences in most settings	Desirable consequences *clearly outweigh* undesirable consequences in most settings
Judgement	○	○	●	○	○

**Type of recommendation**	Strong recommendation against the intervention	Conditional recommendation against the intervention	Conditional recommendation for either the intervention or the comparison	Conditional recommendation for the intervention
Judgement	○	○	●	○

**Recommendation**	**We suggest fluid restriction in the management of adult ARDS patient. (GRADE 2B, "week recommendation" / Quality of evidence "Moderate")**
**Justification**	**Question:** How should fluid balance be maintained on a daily basis in adult patients with ARDS?<br>**Patients or population:** Adult patients with ARDS<br>**Intervention:** Conservative strategy<br>**Comparison:** Liberal strategy<br>**Outcomes:** Short-term mortality [Note 1]、VFD [Note 2]、Renal Replacement Therapy [Note 3]<br>**Summary of Evidence:** As a result of a systematic review, three RCTs comparing adult patients with ARDS who underwent fluid restriction with patients who were not fluid restricted were found. A study that examined the infused fluid volume in patients with shock in addition to patients with ARDS was excluded. While FACTT 2006 included a large number of patients, the other two studies included a small number. There was no significant difference in short-term mortality, but VFD out of 28 days was significantly increased (+2.5 days) in patients who underwent fluid restriction. There was no difference in the need for renal replacement therapy within 60 days.<br><br>**Quality of evidence:** There is no large-scale study that evaluates this CQ other than FACTT 2006, which is a large-scale multi-center study. As a result, two RCTs were included in the meta-analysis for mortality and only FACTT 2006 was included in the meta-analysis for other outcomes. Although FACTT 2006 was insufficiently blinded, it has a low risk for other biases and a sufficient number of patients. Inconsistency in the mortality rate between the studies was low (I^2=0%), but Martin 2002 included only 37 patients while FACTT 2006 included 1000 patients. Indirectness was classified as 'not serious' because the result of FACTT 2006 is well matched to the PICO in this CQ. However, imprecision was classified as 'serious' because the confidence interval overlaps with the clinical decision threshold. Based on the above discussion, the overall quality of evidence was evaluated as 'moderate'.<br><br>**Judgement of benefit, harms and costs:**<br>Fluid restriction didn't decrease mortality, but could shorten the duration of mechanical ventilation without increasing the need for renal replacement therapy. Furosemide, which is used in FACTT 2006, is one of common diuretic drugs and a low-cost drug. Based on these reasons, it is considered that the benefits to be obtained are greater than the harms. If furosemide is used, there is a risk of electrolyte abnormalities.<br><br>**Recommendation:** **We suggest fluid restriction in the management of adult ARDS patient. (GRADE 2B, "week recommendation" / Quality of evidence "Moderate")**<br><br>**Additional considerations:** We have no evidence about how to manage fluid balance, including monitoring or evaluation of fluid status. In recently 2 RCTs, fluid management using extravascular lung water (EVLW) was compared with pulmonary artery wedge pressure (PAWP) or central venous pressure (CVP). EVLW decreased the duration of mechanical ventilation compared with PAWP[10], but there were no survival benefits in both studies[10, 11].
**Subgroup considerations**	none
**Implementation considerations**	We included the study for ARDS patients with hemodynamic stability. If ARDS patient is demonstrating hemodynamic instability, we should consider fluid resuscitation. Furosemide was used in 3 RCTs included our analysis, but we could not find the study about other diuretics.<br>In FACTT 2006, day 1 fluid balance was In 4200ml / Out 3000ml (using furosemide 150mg) in conservative group, In 5000ml / Out 2500ml (using furosemide 75mg) in liberal group.<br>After day 2, the daily fluid balance in conservative group was less than liberal group (-400 to -150ml /day vs. about +500ml). The patients in conservative group were administrated more furosemide (130 to 160mg/day vs. 50 to 80mg/day)

**Monitoring and evaluation considerations**	In the sub-group analysis in FACTT 2006, there was no obvious difference between patients with a central venous catheter and those with a pulmonary artery catheter. Therefore, monitoring with a pulmonary artery catheter is not always required. Although there are other indicators including extravascular lung water content, cerebral natriuretic peptide level, and weight, there is no obvious answer regarding which measurement is more useful and what target value is appropriate for each measurement.<br>When using furosemide, electrolytes should be carefully monitored for abnormalities such as hypokalemia.
**Research priorities**	Further study is required to determine which measurement is useful and what target value is appropriate for each measurement.<br>In addition, another study may be needed to examine the optimal diuretic medication and infusion fluid.<br>The study that followed the patients in FACTT 2006 up to 12 months suggests that management with fluid restriction might be a risk factor for cognitive dysfunction (10). Therefore, an additional study to examine long-term outcomes is also necessary.

Note 1) Short-term mortality was defined as 30-day or 60-day.

Note 2) Out of 28 days, the number of days for which the patient is not dependent on the mechanical ventilator. If the patient dies within 28 days, the number should be zero.

Note 3) Need for renal replacement therapy within 60 days

1. Donnelly SC, MacGregor I, Zamani A, et al. Plasma elastase levels and the development of the adult respiratory distress syndrome. *Am J Respir Crit Care Med* **151**(5): 1428-33, 1995. PMID 7735596
2. Moraes TJ, Chow CW, Downey GP. Proteases and lung injury. *Crit Care Med* **31**(4 Suppl): S189-94, 2003. PMID 12682439
3. Iwata K, Doi A, Ohji G, et al. Effect of neutrophil elastase inhibitor (sivelestat sodium) in the treatment of acute lung injury (ALI) and acute respiratory distress syndrome (ARDS): a systematic review and meta-analysis. *Intern Med* **49**(22): 2423-32, 2010. PMID 21088343
4. Aikawa N, Kawasaki Y. Clinical utility of the neutrophil elastase inhibitor sivelestat for the treatment of acute respiratory distress syndrome. *Ther Clin Risk Manag* **10**: 621-9, 2014. PMID 25120368

CQ11. Study flow diagram

7687 records identified through database searching

Medline（PubMed）	3575
Embase	3813
Cochrane CENTRAL	235
Ichushi	64

Additional record identified through other sources

Hand-search 7

7694 records identified

1193 duplicates

6501 records after duplicates removed

6429 records excluded

Other than English and Japanese	7
Non-RCT	4735
Different patients /interventions /comparisons	1684
Duplicate	3

72 full-text articles assessed for eligibility

66 of full-text articles excluded

Different interventions/ comparisons	55
Non-RCT	8
Duplicate	3

6 included in meta analysis

Short-term (<90d) mortality	6
Severe adverse effects	2
Ventilator-free days (VFD)	3

CQ11
Risk of bias table, Severe adverse effects

Outcome		Severe adverse effects		risk of bias		serious (-1)			
番号	著者名 発表年（Forest plot表示）	risk of bias評価							
		ランダム割付順番の生成 random sequence generation	割り付けの隠蔽化 allocation concealment	ブラインド blinding		不完全なアウトカムデータ incomplete outcome data	選択されたアウトカムの報告 selective outcome reporting	その他のバイアス Other sources of bias	研究内でのバイアスのリスク Risk of bias within a study
				研究参加者と治療提供者 participants and	アウトカム評価者 outcome assessors				
3	Tamakuma 1998	Unclear risk	Unclear risk	High risk	High risk	Unclear risk	Unclear risk	Unclear risk	High risk
4	Zeiher 2004	Low risk	Low risk	Low risk	Low risk	Low risk	Unclear risk	Low risk	Low risk
番号	著者名 発表年（Forest plot表示）	risk of biasコメント							
3	Tamakuma 1998	ランダム化の方法が未記載	割り付け隠蔽化の方法が未記載	盲検化されていない可能性	盲検化されていない可能性	6 /20が脱落	事前に計画されたプロトコールが入手できなかった	情報が不十分	high2項目
4	Zeiher 2004	中央でランダム化	中央で割り付け、薬剤師により外観から薬剤が推定できないようカバーが	研究参加者、治療提供者ともブラインド化されている	アウトカム評価者もブラインド化されている	データの欠損がない	評価するための十分な情報がない	他のバイアスがない（中間解析で試験中断あり）	low6項目、high0項目

CQ11
Risk of bias table VFD

Outcome		VFD		risk of bias		not serious (0)			
番号	著者名 発表年（Forest plot表示）	risk of bias評価							
		ランダム割付順番の生成 random sequence generation	割り付けの隠蔽化 allocation concealment	ブラインド blinding		不完全なアウトカムデータ incomplete outcome data	選択されたアウトカムの報告 selective outcome reporting	その他のバイアス Other sources of bias	研究内でのバイアスのリスク Risk of bias within a study
				研究参加者と治療提供者 participants and	アウトカム評価者 outcome assessors				
3	Tamakuma 1998	Unclear risk	Unclear risk	High risk	High risk	Unclear risk	Unclear risk	Unclear risk	High risk
4	Zeiher 2004	Low risk	Low risk	Low risk	Low risk	Low risk	Unclear risk	Low risk	Low risk
6	Shirai 2006	Low risk	Low risk	High risk	High risk	Low risk	Unclear risk	Low risk	Unclear risk
番号	著者名 発表年（Forest plot表示）	risk of biasコメント							
3	Tamakuma 1998	ランダム化の方法が未記載	割り付け隠蔽化の方法が未記載	盲検化されていない可能性	盲検化されていない可能性	6 /20が脱落	事前に計画されたプロトコールが入手できなかった	情報が不十分	high2項目
4	Zeiher 2004	中央でランダム化	中央で割り付け、薬剤師により外観から薬剤が推定できないようカバーが	研究参加者、治療提供者ともブラインド化されている	アウトカム評価者もブラインド化されている	データの欠損がない	評価するための十分な情報がない	他のバイアスがない（中間解析で試験中断あり）	low6項目、high0項目
6	Shirai 2006	封筒法で行った	封筒法で行った	対照群は非使用群	対照群は非使用群	100%フォローされた	事前に計画されたプロトコールが閲覧できなかった	この研究には他のバイアスはなし	Low 4項目だが、High 2項目あり、Uncelarとした

	Random sequence generation (selection bias)	Allocation concealment (selection bias)	Blinding of participants and personnel (performance bias)	Blinding of outcome assessment (detection bias)	Incomplete outcome data (attrition bias)	Selective reporting (reporting bias)	Other bias
Endo 2006	?	+	−	+	+	?	?
Kadoi 2004	?	+	+	+	+	?	?
Nakayama 2013	+	+	−	−	+	?	?
Shirai 2006	+	+	−	−	+	?	+
Tamakuma 1998	?	?	−	−	?	?	?
Zeiher 2004	+	+	+	+	+	?	+

Short term mortality

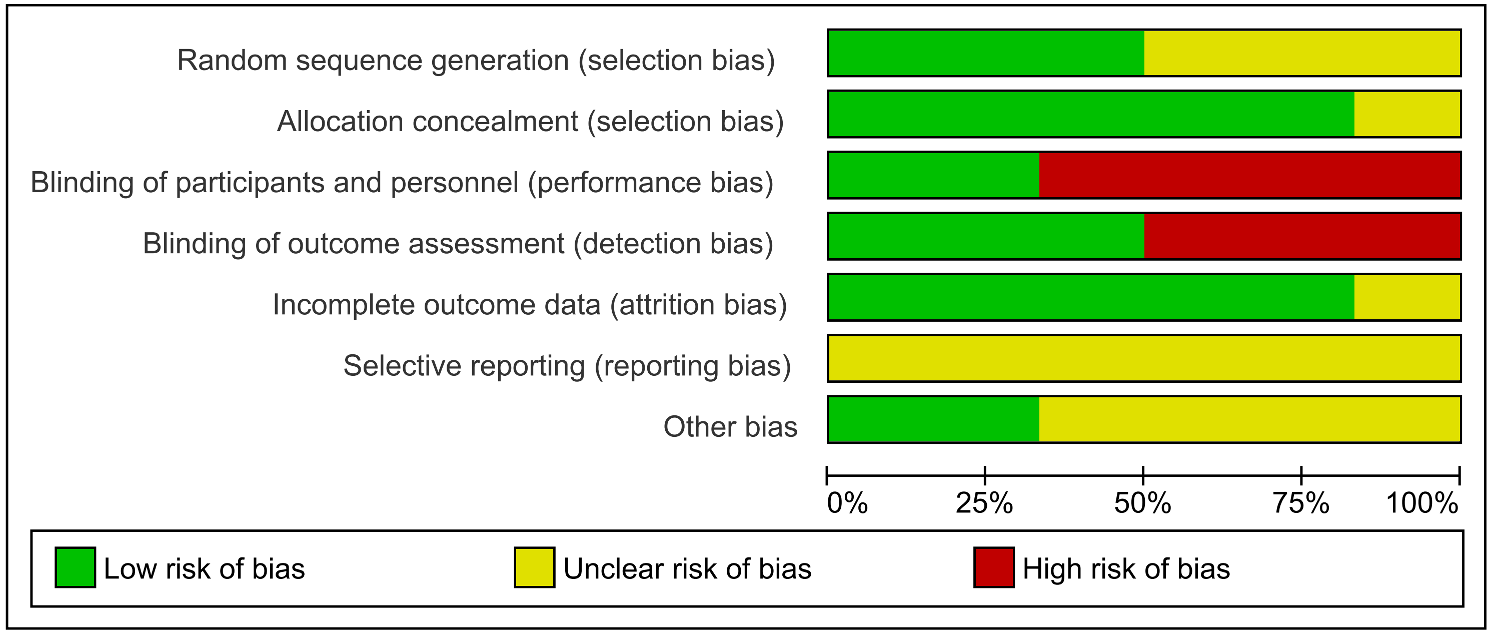

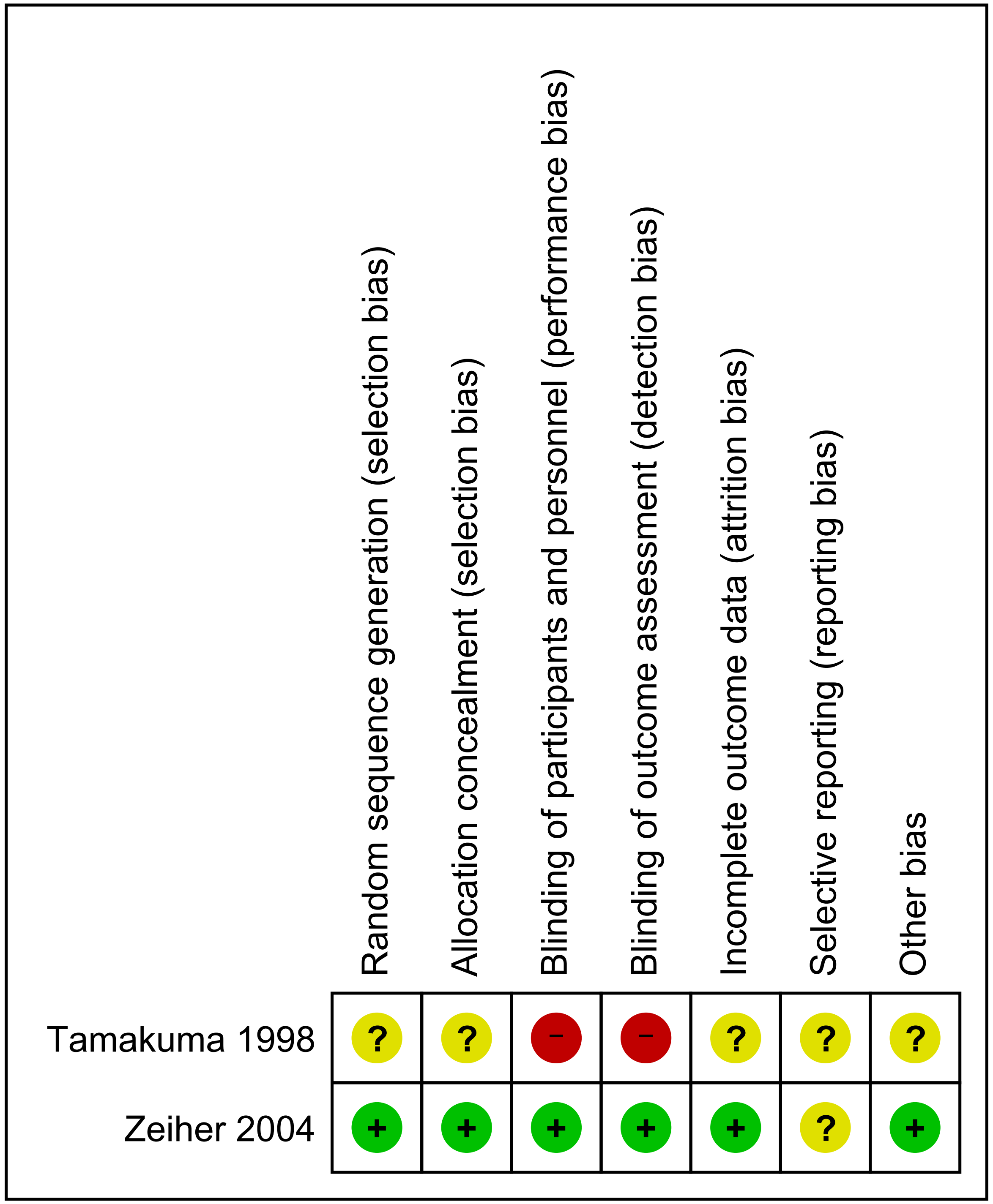

Severe complication

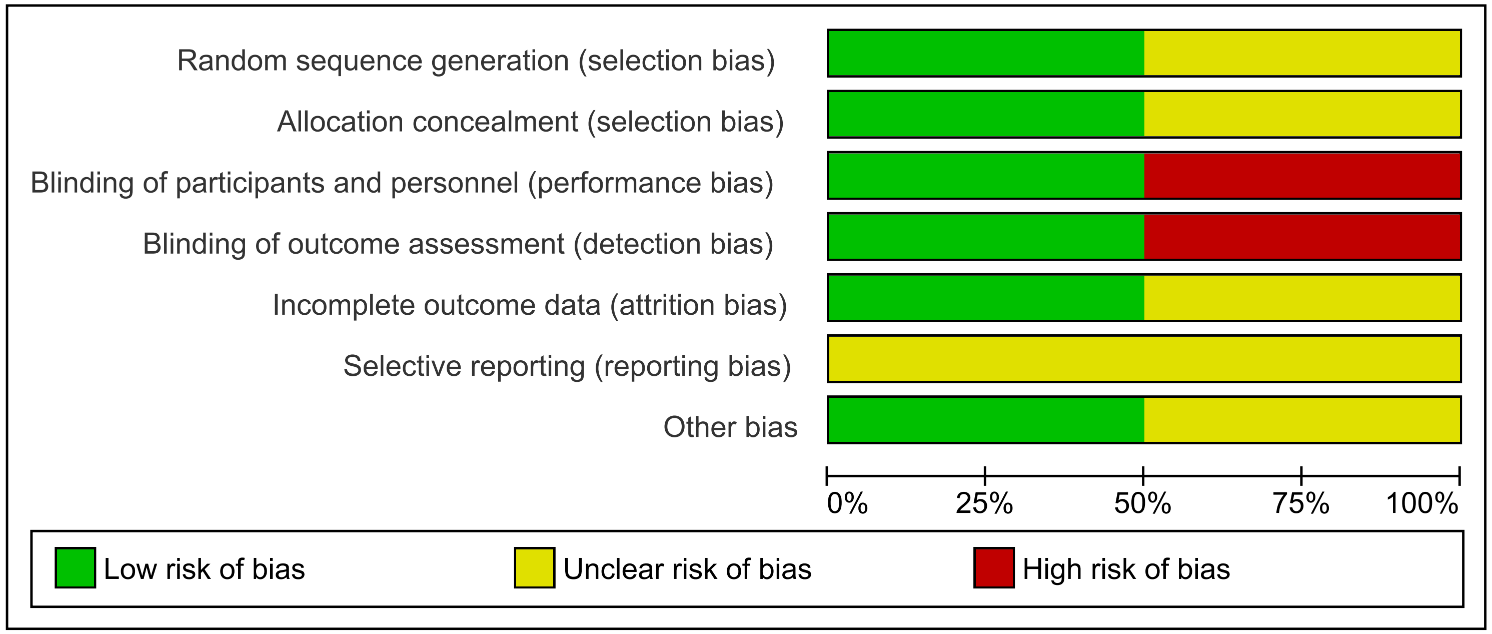

CQ11 Risk of bias summary, Risk of bias graph

VFD

	Random sequence generation (selection bias)	Allocation concealment (selection bias)	Blinding of participants and personnel (performance bias)	Blinding of outcome assessment (detection bias)	Incomplete outcome data (attrition bias)	Selective reporting (reporting bias)	Other bias
Shirai 2006	+	+	−	−	+	?	+
Tamakuma 1998	?	?	−	−	?	?	?
Zeiher 2004	+	+	+	+	+	?	+

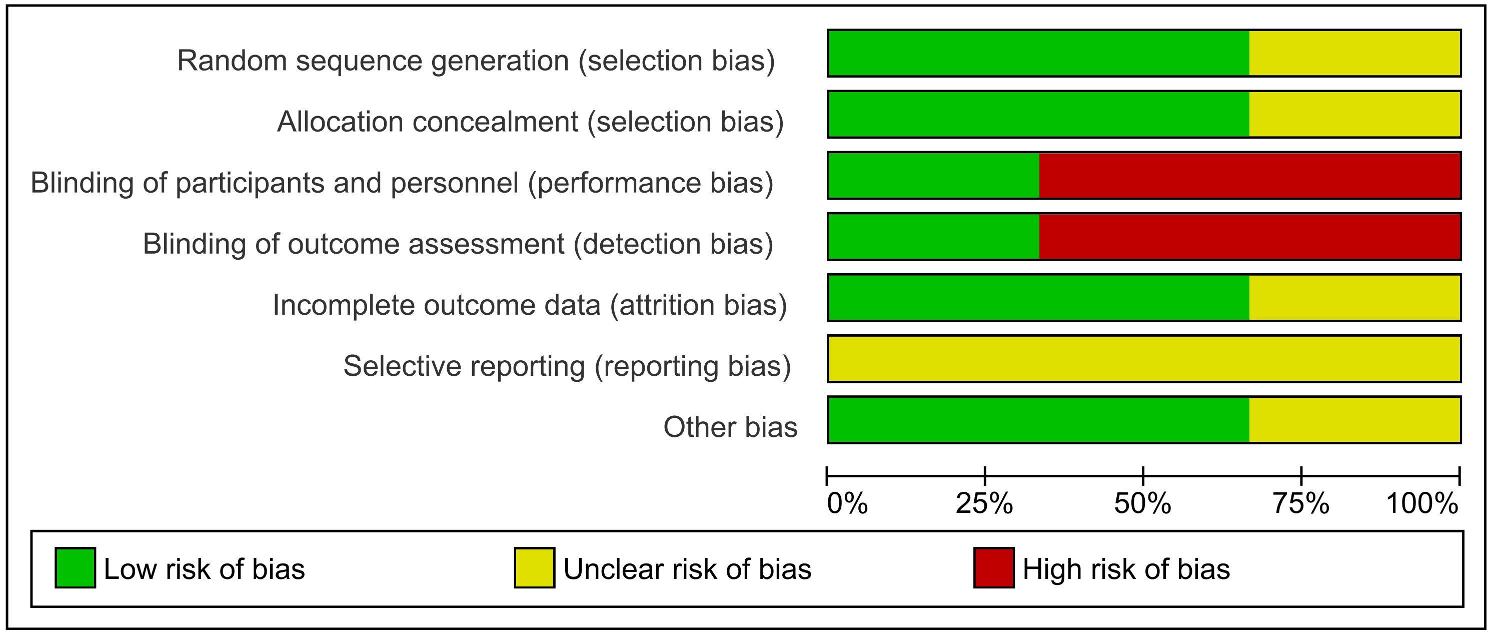

Short term mortality

Study or Subgroup	Sivelestat Events	Sivelestat Total	Control Events	Control Total	Weight	Risk Ratio M-H, Random, 95% CI
Endo 2006	2	13	5	13	5.7%	0.40 [0.09, 1.70]
Kadoi 2004	3	12	3	12	6.1%	1.00 [0.25, 4.00]
Nakayama 2013	2	14	3	8	4.9%	0.38 [0.08, 1.82]
Shirai 2006	0	19	0	16		Not estimable
Tamakuma 1998	25	113	30	108	32.3%	0.80 [0.50, 1.26]
Zeiher 2004	87	241	74	246	51.0%	1.20 [0.93, 1.55]
**Total (95% CI)**		**412**		**403**	**100.0%**	**0.92 [0.64, 1.32]**
Total events	119		115			

Heterogeneity: $Tau^2 = 0.05$; $Chi^2 = 5.79$, df = 4 (P = 0.22); $I^2 = 31\%$
Test for overall effect: Z = 0.43 (P = 0.66)

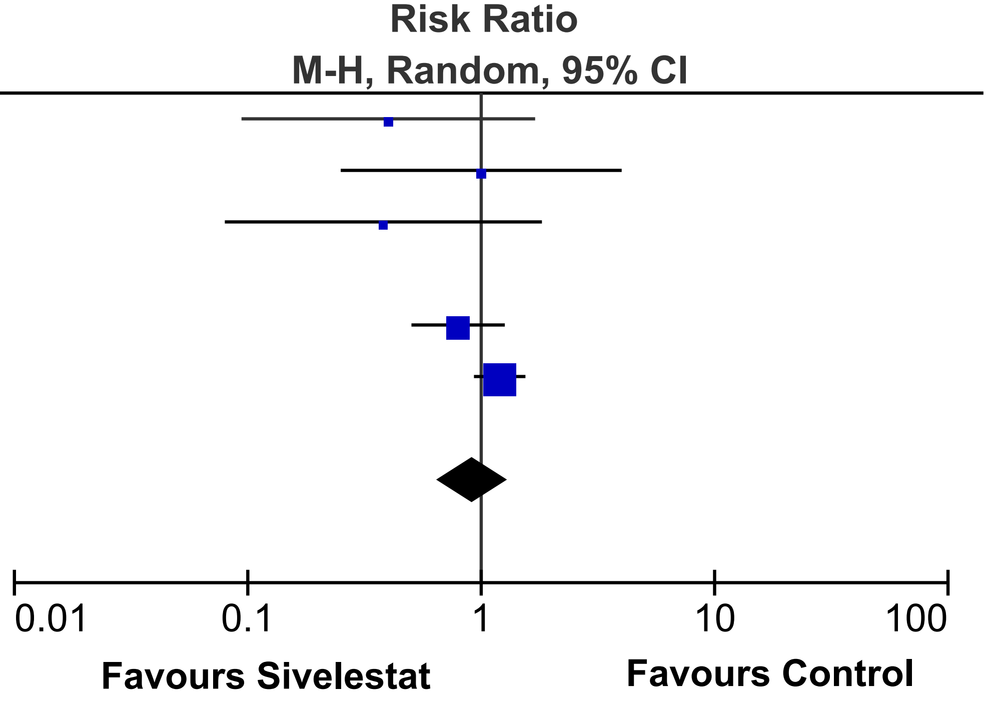

Severe complication

Study or Subgroup	Sivelestat Events	Sivelestat Total	Control Events	Control Total	Weight	Risk Ratio M-H, Random, 95% CI
Tamakuma 1998	21	113	34	109	46.8%	0.60 [0.37, 0.96]
Zeiher 2004	41	241	41	246	53.2%	1.02 [0.69, 1.51]
**Total (95% CI)**		**354**		**355**	**100.0%**	**0.79 [0.47, 1.34]**
Total events	62		75			

Heterogeneity: $Tau^2 = 0.10$; $Chi^2 = 2.91$, df = 1 (P = 0.09); $I^2 = 66\%$
Test for overall effect: Z = 0.86 (P = 0.39)

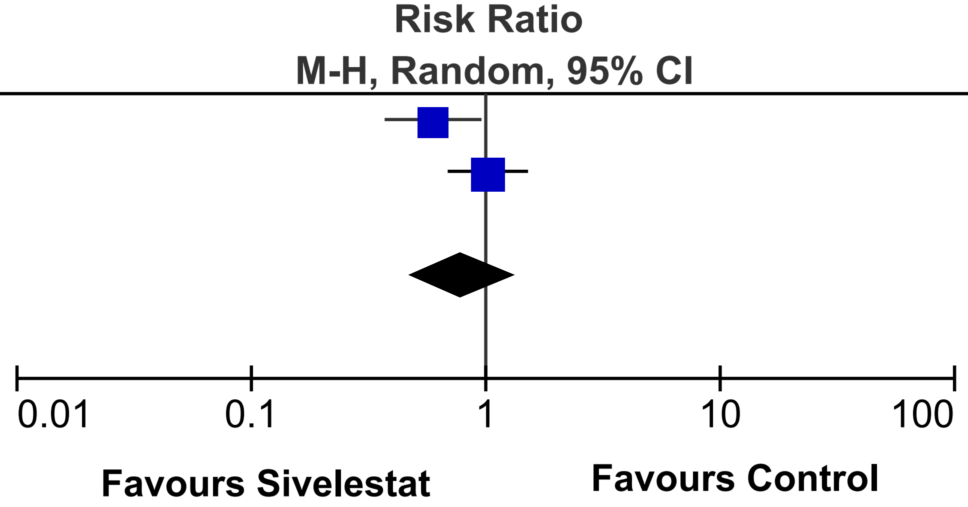

VFD

Study or Subgroup	Sivelestat Mean	Sivelestat SD	Sivelestat Total	Control Mean	Control SD	Control Total	Weight	Mean Difference IV, Random, 95% CI
Shirai 2006	18.1	3.4	19	14.2	4.5	16	31.5%	3.90 [1.22, 6.58]
Tamakuma 1998	12.31	10.57	113	9.96	10.3	108	31.0%	2.35 [-0.40, 5.10]
Zeiher 2004	11.4	10.3	241	11.9	10.1	246	37.6%	-0.50 [-2.31, 1.31]
**Total (95% CI)**			**373**			**370**	**100.0%**	**1.77 [-0.99, 4.52]**

Heterogeneity: $Tau^2 = 4.41$; $Chi^2 = 7.94$, df = 2 (P = 0.02); $I^2 = 75\%$
Test for overall effect: Z = 1.26 (P = 0.21)

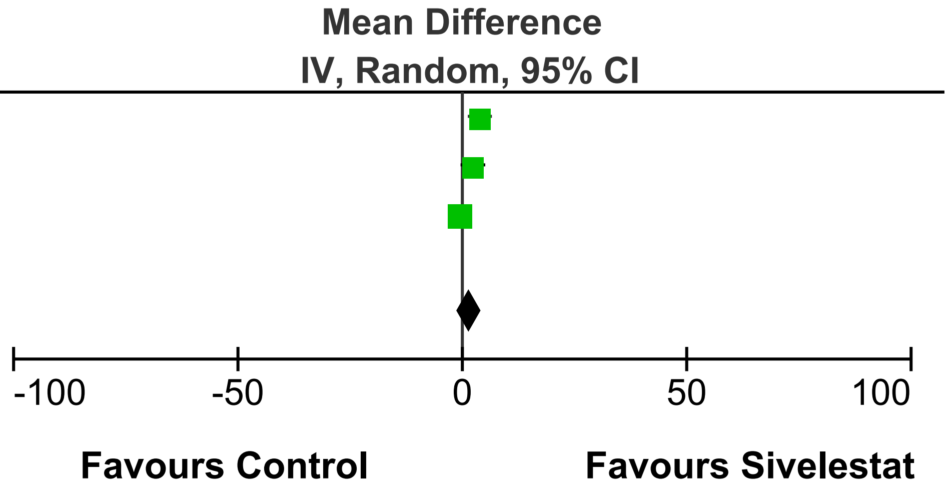

Summary of findings:

Sivelestat compared to placebo for adult ARDS

Patient or population: ARDS
Intervention: Sivelestat
Comparison: placebo

Outcomes	Anticipated absolute effects* (95% CI)		Relative effect (95% CI)	№ of participants (studies)	Quality of the evidence (GRADE)	Comments
	Risk with placebo	Risk with Sivelestat				
Short-term mortality	Study population		RR 0.92 (0.64 to 1.32)	815 (6 RCTs)	⊕◯◯◯ VERY LOW [1 2 3]	
	285 per 1000	**263 per 1000** (183 to 377)				
	Low risk population					
	190 per 1000	**175 per 1000** (122 to 251)				
	High risk population					
	450 per 1000	**414 per 1000** (288 to 594)				
Severe adverse effects	Study population		RR 0.79 (0.47 to 1.34)	709 (2 RCTs)	⊕◯◯◯ VERY LOW [1 2 3]	
	211 per 1000	**167 per 1000** (99 to 283)				
	Low risk population					
	20 per 1000	**16 per 1000** (9 to 27)				
	High risk population					
	240 per 1000	**190 per 1000** (113 to 322)				
VFD	Mean **12.0** days	1.77 days more MD (0.99 fewer to 4.52 more)	-	743 (3 RCTs)	⊕◯◯◯ VERY LOW [1 2 3]	

***The risk in the intervention group** (and its 95% confidence interval) is based on the assumed risk in the comparison group and the **relative effect** of the intervention (and its 95% CI).

CI: Confidence interval; **RR:** Risk ratio; **MD:** Mean difference

GRADE Working Group grades of evidence
High quality: We are very confident that the true effect lies close to that of the estimate of the effect
Moderate quality: We are moderately confident in the effect estimate: The true effect is likely to be close to the estimate of the effect, but there is a possibility that it is substantially different
Low quality: Our confidence in the effect estimate is limited: The true effect may be substantially different from the estimate of the effect
Very low quality: We have very little confidence in the effect estimate: The true effect is likely to be substantially different from the estimate of effect

1. A lot of high risk of bias in the blinding procedure
2. High heterogeneity of I^2 = 31%, 66% and 75%.
3. Wide range of 95%CI due to a limited number of patients

CQ 11

Question: Should neutrophil elastase inhibitors be used in the treatment of adult patients with ARDS?

Quality assessment							№ of patients		Effect		Quality	Importance
**№ of studies**	**Study design**	**Risk of bias**	**Inconsistency**	**Indirectness**	**Imprecision**	**Other considerations**	**Sivelestat for ARDS**	**Placebo**	**Relative (95% CI)**	**Absolute (95% CI)**		
Short-term (<90d) mortality												
6	Randomised trials	Serious[1]	Serious[2]	Not serious	Serious[3]	None	119 per 412 (28.9%)	115 per 403 (28.5%)	**RR 0.92** (0.64 to 1.32)	23 fewer per 1000 (from 91 more to 103 fewer)	⨁⊖⊖⊖ VERY LOW	CRITICAL
								19.0%		15 fewer per 1000 (from 61 more to 68 fewer)		
								45.0%		36 fewer per 1000 (from 144 more to 162 fewer)		
Severe adverse effects												
2	Randomised trials	Serious[1]	Serious[2]	Not serious	Serious[3]	None	62 per 354 (17.5%)	75 per 355 (21.1%)	**RR 0.79** (0.47 to 1.34)	44 fewer per 1000 (from 72 more to 112 fewer)	⨁⊖⊖⊖ VERY LOW	CRITICAL
								2.0%		4 fewer per 1000 (from 7 more to 11 fewer)		
								24.0%		50 fewer per 1000 (from 82 more to 127 fewer)		
VFD												
3	Randomised trials	Serious[1]	Serious[2]	Not serious	Serious[3]	None	373	370	-	MD 1.77 days more (from 0.99 fewer to 4.52 more)	⨁⊖⊖⊖ VERY LOW	CRITICAL

CI: Confidence interval; RR: Risk ratio; MD: Mean difference

1. There is a high risk of bias in the blinding procedure
2. High heterogeneity of I^2 = 31%, 66% and 75%.
3. Wide range of 95%CI due to a limited number of patients

Evidence-to-Decision Table

CQ11：Should neutrophil elastase inhibitors be used in the treatment of adult patients with ARDS?

PATIENTS：ADULT PATIENTS WITH ARDS

INTERVENTION：SIVELESTAT (NEUTROPHIL ELSTASE INHIBITOR)

	CRITERIA	JUDGEMENTS	RESEARCH EVIDENCE	ADDITIONAL CONSIDERATION
PROBLEM	**Is the problem a priority?**	○No<br>○Probably no<br>○Probably yes<br>●Yes<br>------------------<br>○Varies<br>○Don't know	The pathogenesis of acute respiratory distress syndrome (ARDS) is pulmonary edema caused by increased permeability associated with nonspecific alveolar inflammation. Neutrophil elastase is thought to be one of the most important mediators related to the pathogenesis of ARDS (1, 2). A neutrophil elastase inhibitor, available for clinical use in Japan, has been intensively investigated as a treatment option to improve the prognosis of patients with ARDS. Several meta-analyses showed that a neutrophil elastase inhibitor did not improve mortality (3), while other studies suggested its potential benefits (4). This discrepancy indicates the importance of this issue. Since a neutrophil elastase inhibitor is reimbursed by the national health insurance system and is widely administered in Japan, the priority of this issue is high.	
DESIRABLE AND UNDESIRABLE EFFECTS	**What is the overall certainty of the evidence of effects?**	●Very low<br>○Low<br>○Moderate<br>○High<br>------------------<br>○No included studies	(see below)	
	**Is there important uncertainty about or variability in how much people value the main outcomes?**	○Important uncertainty or variability<br>●Possibly important uncertainty or variability<br>○Possibly no important uncertainty or variability<br>○No important uncertainty or variability<br>------------------<br>○No known undesirable outcomes		
	**How substantial are the desirable anticipated effects?**	○Trivial<br>○Small<br>○Moderate<br>○Large<br>------------------<br>●Varies<br>○Don't know		
	**How substantial are the undesirable anticipated effects?**	○Large<br>○Moderate<br>○Small<br>○Trivial<br>------------------<br>●Varies<br>○Don't know		
	**Does the balance between desirable and undesirable effects favor the intervention**	○Favors the comparison<br>○Probably favors the comparison<br>○Does not favor either the intervention or		

The relative importance or values of the main outcomes of interest:

Outcomes	Relative importance	Certainty of the evidence (GRADE)
Mortality (short term)(Note1	CRITICAL	⊕⊖⊖⊖<br>VERY LOW
Significant adverse events	CRITICAL	⊕⊖⊖⊖<br>VERY LOW
VFD(Note2	IMPORTANT	⊕⊖⊖⊖<br>VERY LOW

Summary of findings:

Outcome	Risk with placebo	Risk with intervention	Absolute effect (95% CI)	Relative risk (RR) (95% CI)
Short-term (<90d) mortality(Note1	285 per 1000	263 per 1000 (183 to 377)	23 fewer per 1000 (from 91 more to 103 fewer)	RR 0.92 (0.64 to 1.32)
	190 per 1000	175 per 1000 (122 to 251)	15 fewer per 1000 (from 61 more to 68 fewer)	
	450 per 1000	414 per 1000 (288 to 594)	36 fewer per 1000 (from 144 more to 162 fewer)	
Severe adverse effects	211 per 1000	167 per 1000 (99 to 283)	44 fewer per 1000 (from 72 more to 112 fewer)	RR 0.79 (0.47 to 1.34)
	20 per 1000	16 per 1000 (9 to 27)	4 fewer per 1000 (from 7 more to 11 fewer)	
	240 per 1000	190 per 1000 (113 to 322)	50 fewer per 1000 (from 82 more to 127 fewer)	
VFD(Note2	Average 12.0 days	Average 13.8 days	MD 1.77 more (from 0.99 fewer to 4.52 more)	-

Summary：Neutrophil elastase inhibitor had no effect on short-term (<90d) mortality, the incidence of significant adverse events or the number of VFD. Certainty of the evidence「VERY LOW」

	**or the comparison?**	the comparison<br>○Probably favors the intervention<br>○Favors the intervention<br>------------------<br>●Varies<br>○Don't know		
RESOURCES REQUIRED	**How large are the resource requirements (costs)?**	○Large costs<br>●Moderate costs<br>○Negligible costs and savings<br>○Moderate savings<br>○Large savings<br>------------------<br>○Varies<br>○Don't know	Since neutrophil elastase inhibitors are administered intravenously through peripheral venous catheters, limited equipment is necessary. However, additional costs are required to buy this drug.<br><br>Neutrophil elastase inhibitor 6,216 – 13,551 JPY/day	
	**Does the cost effectiveness of the intervention favor the intervention or the comparison?**	○Favors the comparison<br>●Probably favors the comparison<br>○Does not favor either the intervention or the comparison<br>○Probably favors the intervention<br>○Favors the intervention<br>------------------<br>○Varies<br>○No included studies	The efficacy of this drug has not been confirmed, and additional expenses are necessary to buy this drug.	
EQUITY	**What would be the impact on health equity?**	○Reduced<br>●Probably reduced<br>○Probably no impact<br>○Probably increased<br>○Increased<br>------------------<br>○Varies<br>○Don't know	Since no specialized medical facilities or equipment are necessary, the health equity may be adjusted.	
ACCEPTABILITY	**Is the option acceptable to key stakeholders?**	○No<br>○Probably no<br>○Probably yes<br>○Yes<br>------------------<br>○Varies<br>●Don't know	It is unclear whether it will be accepted by key stakeholders, because this drug is expensive.	
FEASIBILITY	**Is the intervention feasible implement?**	○No<br>○Probably no<br>●Probably yes<br>○Yes<br>------------------<br>○Varies<br>○Don't know	The intervention is feasible to implement, because this drug can be administered intravenously and does not require specialized medical facilities or equipment.	

Recommendation

CQ11：Should neutrophil elastase inhibitors be used in the treatment of adult patients with ARDS?

Balance of consequences	Undesirable consequences clearly outweigh desirable consequences in most settings	Undesirable consequences probably outweigh desirable consequences in most settings	The balance between desirable and undesirable consequences is closely balanced or uncertain	Desirable consequences probably outweigh undesirable consequences in most settings	Desirable consequences clearly outweigh undesirable consequences in most settings
Judgement	○	○	●	○	○

Type of recommendation	Strong recommendation against the intervention	Conditional recommendation against the intervention	Conditional recommendation for either the intervention or the comparison	Conditional recommendation for the intervention
Judgement	○	●	○	○

**Recommendation**	**We do not suggest the use of neutrophil elastase inhibitors in adult patients with ARDS. (GRADE 2D, Strength of recommendation “weak recommendation” / Quality of evidence “very low”)**
**Justification**	**Clinical question**: Should neutrophil elastase inhibitors be used in the treatment of adult patients with ARDS?<br>**Patient or population**：Adult patients with ARDS<br>**Intervention**：Sivelestat (Neutrophil elstase inhibitor)<br>**Comparison**：Placebo<br>**Outcomes:** Mortality (short term) Note 1, Significant adverse events, VFD Note 2<br><br>**Summary of the evidence:** A total of six randomized clinical trials (RCTs, 815 patients) were selected in a systematic review. Meta-analysis demonstrated that neutrophil elastase inhibitors did not improve the short-term (<90 days) mortality (RR 0.92, 95%CI 0.64-1.32), the rate of severe adverse effects (RR 0.79, 95%CI 0.47-1.34) or number of ventilation-free days (VFD) (Mean 1.58 days more, 95%CI 2.72 days fewer to 5.89 days more).<br><br>**Quality of evidence:** Many studies had a high risk of bias in blinding. Moderate to severe inconsistency was observed for (short-term (<90 days) mortality, I^2 = 31%; and severe adverse effects, I^2 = 31%; VFD, I^2 =86%). No indirectness was observed. Since the number of patients was less than optimal for the information size resulting in a large 95%CI, the imprecision of this meta-analysis was high. Publication bias could not be determined because of the small number of reported studies.<br><br>**Judgement of benefit and harm, resources and cost:** Systematic review demonstrated that neither efficacy nor significant adverse effects were found. The benefit was considered to be low compared to the increase in cost.<br><br>**Recommendations:** We do not suggest the use of neutrophil elastase inhibitors in adult patients with ARDS. (GRADE 2D, Strength of recommendation “weak recommendation” / Quality of evidence “very low”)<br><br>**Additional Considerations:** Neutrophil elastase inhibitors are reimbursed by the national health insurance system in Japan to treat patients with ARDS with the proviso that the use of neutrophil elastase inhibitors is not recommended in patients with multiple organ failure (four or more organs), burn injuries, or trauma. A nationwide survey conducted by the Japanese Respiratory Society in 2010 showed that neutrophil elastase inhibitors are widely used in Japan for the treatment of patients with ARDS.
**Subgroup considerations**	None
**Implementation considerations**	Because of a lot of drug incompatibilities, separated infusion lines are often necessary.
**Monitoring and evaluation considerations**	Cardiorespiratory monitoring and blood tests are necessary to identify the onset of adverse effects.
**Research possibilities**	Due to the limited number of high-quality RCTs, large-scale, high-quality clinical trials are necessary to demonstrate the efficacy of neutrophil elastase inhibitors in adult patients with ARDS.

Note 1) Short-term (<90 days) mortality indicates death within 90 days, which was analyzed as the main outcome in each study.
Note 2) Ventilation-free days (VFD) indicates the number of days without a ventilator support during a 28-day period. If patients died within 28 days, VFD was defined as zero.

References

1. Donnelly SC, MacGregor I, Zamani A, et al. Plasma elastase levels and the development of the adult respiratory distress syndrome. *Am J Respir Crit Care Med* **151**(5): 1428-33, 1995. PMID 7735596
2. Moraes TJ, Chow CW, Downey GP. Proteases and lung injury. *Crit Care Med* **31**(4 Suppl): S189-94, 2003. PMID 12682439
3. Iwata K, Doi A, Ohji G, et al. Effect of neutrophil elastase inhibitor (sivelestat sodium) in the treatment of acute lung injury (ALI) and acute respiratory distress syndrome (ARDS): a systematic review and meta-analysis. *Intern Med* **49**(22): 2423-32, 2010. PMID 21088343
4. Aikawa N, Kawasaki Y. Clinical utility of the neutrophil elastase inhibitor sivelestat for the treatment of acute respiratory distress syndrome. *Ther Clin Risk Manag* **10**: 621-9, 2014. PMID 25120368

CQ12. Study flow diagram

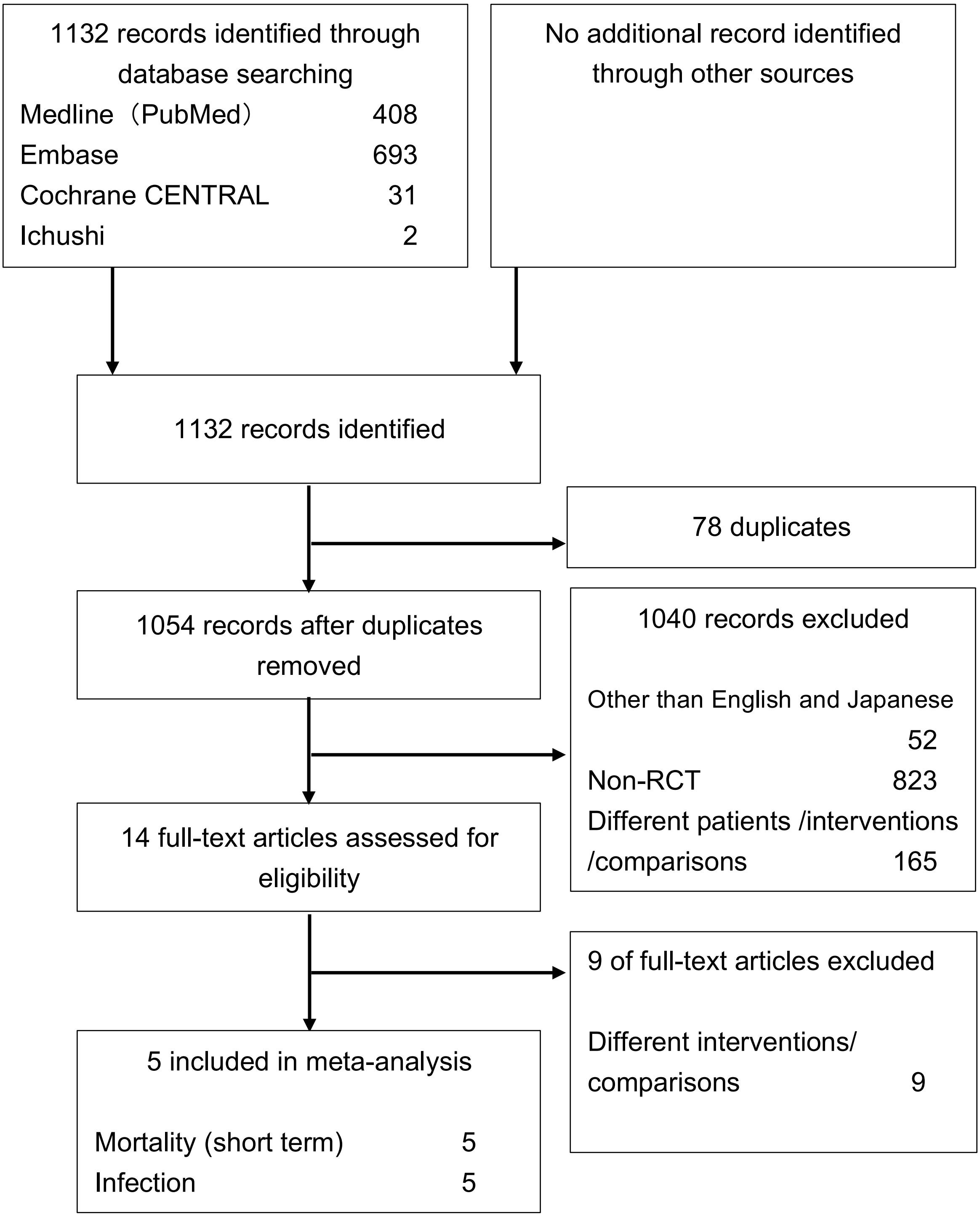

CQ12
Risk of bias table, mortality

Outcome		Short term mortality		risk of bias		not serious (0)			
番号	著者名 発表年（Forest plot表示）	risk of bias評価							
		ランダム割付順番の生成 random sequence generation	割り付けの隠蔽化 allocation concealment	ブラインド blinding		不完全なアウトカムデータ incomplete outcome data	選択されたアウトカムの報告 selective outcome reporting	その他のバイアス Other sources of bias	研究内でのバイアスのリスク Risk of bias within a study
				研究参加者と治療提供者 participants and personnel	アウトカム評価者 outcome assessors				
1	Annane 2006	Unclear risk	Unclear risk	Low risk	Low risk	Low risk	Low risk	High risk	Unclear risk
5	ARDS network 2006	Low risk	Unclear risk	Low risk	Unclear risk	Low risk	Low risk	Unclear risk	Low risk
2	Bernard1987	Low risk	Low risk	Low risk	Low risk	Unclear risk	Unclear risk	Unclear risk	Unclear risk
3	Meduri 1998	Low risk	Low risk	Low risk	Unclear risk	Low risk	Unclear risk	Unclear risk	Unclear risk
4	Meduri 2007	Low risk	Low risk	Low risk	Unclear risk	High risk	Low risk	Unclear risk	Unclear risk
		↑↓「アブストラクトテーブル」からコピペしてください.							
番号	著者名 発表年（Forest plot表示）	risk of biasコメント							
1	Annane 2006	元の論文は、二重盲検化プラセボ対照試験だが、本論文はそれの事後解析である。	元の論文は、二重盲検化プラセボ対照試験だが、本論文はそれの事後解析である。	元の論文は、二重盲検化プラセボ対照試験だが、本論文はそれの事後解析であるがバイアスリスクはない。	元の論文は、二重盲検化プラセボ対照試験だが、本論文はそれの事後解析であるがバイアスリスクはない。	事後解析なので欠損なし。	結果は図表で示されている。	事後解析であること。	ほとんど、Unclear riskのため。
	ARDS network 2006	置換ブロック法を用いて盲検化	記載なし	要約に、「二重盲検を行った」、との記載	記載なし	図により患者数の経過が記載されている。	主要結果から代替結果まで表にまとめられている。	筋弛緩薬の使用	全体として、Low riskが多い。
2	Bernard1987	コンピューターによるランダム化	順番に番号をつけたバイアルに、ステロイドまたはプラセボを入れた。バイアルの中身は盲検化されていた。	研究グループのメンバー、患者本人・家族も盲検化されていた。	研究グループのメンバー、患者本人・家族も盲検化されていた。	ITT（intention-to-treat）解析の記載はないが、実際にはITT解析されたいる。	サンプルサイズの記載がない。<br>途中で試験を中止した疑い。	資金提供した製薬企業と使用薬剤の関連が不明。	ほぼ、Low riskであるため。
3	Meduri 1998	乱数を発生させるジェネレーターを使用。	二重盲検化プラセボ対照試験の記載	二重盲検化プラセボ対照試験の記載	記載なし。	図により患者数の経過が表示されている。	サンプルサイズの記載がない。	連続検定により中断した記載。<br>早期中断の疑い。	クロスオーバー（プラセボ→ステロイド）症例（4例、いずれも死亡）が多い。<br>早期終了している。
4	Meduri 2007	乱数作成装置で作成した乱数表を使用（ネット上の補遺に記載）	封書を利用した盲検化（ネット上の補遺に記載）	「二重盲検化プラセボ対照試験」の記載	明瞭な記載なし<br>（多分、解析時に情報を見ることができている。）	プロトコール違反、脱落者数が母数の10%を超える	表で報告されている。	「結果」が、主要アウトカム（死亡）ではなく、<br>代替のアウトカムである。	ほぼ、Low riskのため。

CQ12

Risk of bias table, the incidence of infection

Outcome		The incidence of infection		risk of bias		not serious (0)			
番号	著者名 発表年（Forest plot表示）	risk of bias評価							
		ランダム割付順番の生成 random sequence generation	割り付けの隠蔽化 allocation concealment	ブラインド blinding		不完全なアウトカムデータ incomplete outcome data	選択されたアウトカムの報告 selective outcome reporting	その他のバイアス Other sources of bias	研究内でのバイアスのリスク Risk of bias within a study
				研究参加者と治療提供者 participants and personnel	アウトカム評価者 outcome assessors				
1	Annane 2006	Unclear risk	Unclear risk	Low risk	Low risk	Low risk	Low risk	High risk	Unclear risk
5	ARDS network 2006	Low risk	Unclear risk	Low risk	Low risk	Low risk	Low risk	Low risk	Unclear risk
2	Bernard1987	Low risk	Low risk	Low risk	Low risk	Unclear risk	Unclear risk	Unclear risk	Unclear risk
3	Meduri 1998	Low risk	Low risk	Low risk	High risk	Low risk	Unclear risk	Unclear risk	Unclear risk
4	Meduri 2007	Low risk	Low risk	Low risk	Unclear risk	High risk	Low risk	Low risk	Unclear risk
番号	著者名 発表年（Forest plot表示）	risk of biasコメント							
1	Annane 2006	元の論文は、二重盲検化プラセボ対照試験だが、本論文はそれの事後解析である。	元の論文は、二重盲検化プラセボ対照試験だが、本論文はそれの事後解析である。	元の論文は、二重盲検化プラセボ対照試験だが、本論文はそれの事後解析であるがバイアスリスクはない。	元の論文は、二重盲検化プラセボ対照試験だが、本論文はそれの事後解析であるがバイアスリスクはない。	事後解析なのでけっそんなし。	結果は図表で示されている。	事後解析であること。	ほとんど、Unclear riskのため。
	ARDS network 2006	置換ブロック法を用いて盲検化	記載なし	要約に、「二重盲検を行った」、との記載	記載なし	図により患者数の経過が記載されている。	主要結果から代替結果まで表にまとめられている。	筋弛緩薬の使用	全体として、Low riskが多い。
2	Bernard1987	コンピューターによるランダム化	順番に番号をつけたバイアルに、ステロイドまたはプラセボを入れた。バイアルの中身は盲検化されていた。	研究グループのメンバー、患者本人・家族も盲検化されていた。	研究グループのメンバー、患者本人・家族も盲検化されていた。	ITT（intention-to-treat）解析の記載はないが、実際にはITT解析されたいる。	サンプルサイズの記載がない。<br>途中で試験を中止した疑い。	資金提供した製薬企業と使用薬剤の関連が不明。	ほぼ、Low riskであるため。
3	Meduri 1998	乱数を発生させるジェネレーターを使用。	二重盲検化プラセボ対照試験の記載	二重盲検化プラセボ対照試験の記載	記載なし。影響する可能性あり。	図により患者数の経過が表示されている。	サンプルサイズの記載がない。	連続検定により中断した記載。<br>早期中断の疑い。	クロスオーバー（プラセボ→ステロイド）症例（4例、いずれも死亡）が多い。<br>早期終了している。
4	Meduri 2007	乱数作成装置で作成した乱数表を使用（ネット上の補遺に記載）	封書を利用した盲検化（ネット上の補遺に記載）	「二重盲検化プラセボ対照試験」の記載	明瞭な記載なし（多分、解析時に情報を見ることができている。）	プロトコール違反、脱落者数が母数の10%を超える	表で報告されている。	表中に報告されている。	ほぼ、Low riskのため。

CQ12
Risk of bias table VFD

Outcome		VFD		risk of bias		not serious (0)			
番号	著者名 発表年<br>(Forest plot表示)	risk of bias評価							
		ランダム割付順番の生成<br>random sequence generation	割り付けの隠蔽化<br>allocation concealment	ブラインド blinding<br>研究参加者と治療提供者<br>participants and personnel	ブラインド blinding<br>アウトカム評価者<br>outcome assessors	不完全なアウトカムデータ<br>incomplete outcome data	選択されたアウトカムの報告<br>selective outcome reporting	その他のバイアス<br>Other sources of bias	研究内でのバイアスのリスク<br>Risk of bias within a study
1	ARDS network 2006	Low risk	Unclear risk	Low risk	Unclear risk	Low risk	Low risk	Unclear risk	Low risk
5	Meduri 1998	Low risk	Low risk	Low risk	Unclear risk	Low risk	Unclear risk	Unclear risk	Unclear risk
2	Meduri 2007	Low risk	Low risk	Low risk	Unclear risk	High risk	Low risk	Low risk	Unclear risk
9									

番号	著者名 発表年<br>(Forest plot表示)	risk of biasコメント							
1	ARDS network 2006	置換ブロック法を用いて盲検化	記載なし	要約に、「二重盲検を行った」、との記載	記載なし	図により患者数の経過が記載されている。	主要結果から代替結果まで表にまとめられている。	筋弛緩薬の使用	全体として、Low riskが多い。
2	Meduri 1998	乱数を発生させるジェネレーターを使用。	二重盲検化プラセボ対照試験の記載	二重盲検化プラセボ対照試験の記載	記載なし。	図により患者数の経過が表示されている。	サンプルサイズの記載がない。	連続検定により中断した記載。<br>早期中断の疑い。	クロスオーバー(プラセボ→ステロイド)症例(4例、いずれも死亡)が多い。<br>早期終了している。
3	Meduri 2007	乱数作成装置で作成した乱数表を使用(ネット上の補遺に記載)	封書を利用した盲検化<br>(ネット上の補遺に記載)	「二重盲検化プラセボ対照試験」の記載	明瞭な記載なし<br>(多分、解析時に情報を見ることができている。)	プロトコール違反、脱落者数が母数の10%を超える	表で報告されている。	報告済み	ほぼ、Low riskのため。

	Random sequence generation (selection bias)	Allocation concealment (selection bias)	Blinding of participants and personnel (performance bias)	Blinding of outcome assessment (detection bias)	Incomplete outcome data (attrition bias)	Selective reporting (reporting bias)	Other bias
Annane 2006	?	?	+	+	+	+	−
ARDS network 2006	+	?	+	+	+	+	+
Bernard 1987	+	+	+	+	?	?	+
Meduri 1998	+	+	+	?	+	?	?
Meduri 2007	+	+	+	?	−	+	+

Short term mortality

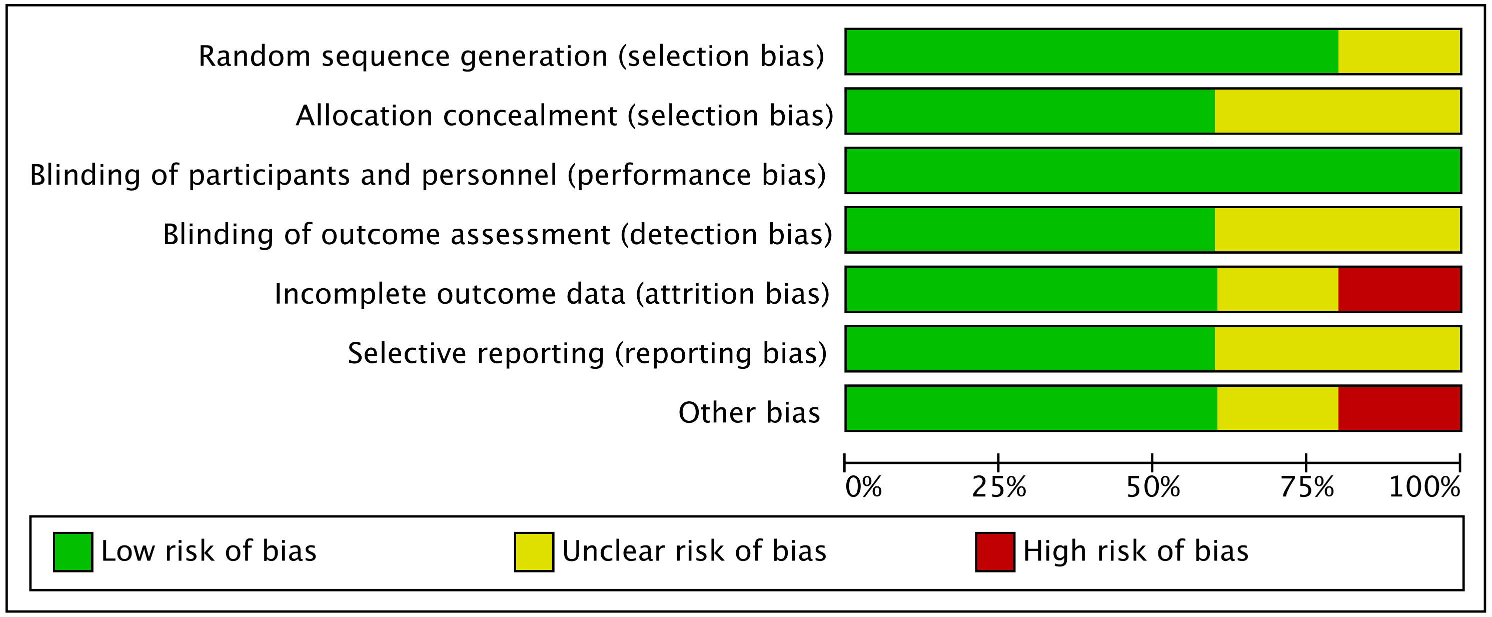

Infection

	Random sequence generation (selection bias)	Allocation concealment (selection bias)	Blinding of participants and personnel (performance bias)	Blinding of outcome assessment (detection bias)	Incomplete outcome data (attrition bias)	Selective reporting (reporting bias)	Other bias
Annane 2006	?	?	+	+	+	+	−
ARDS network 2006	+	?	+	+	+	+	+
Bernard 1987	+	+	+	+	?	?	+
Meduri 1998	+	+	+	−	+	?	?
Meduri 2007	+	+	+	?	−	+	+

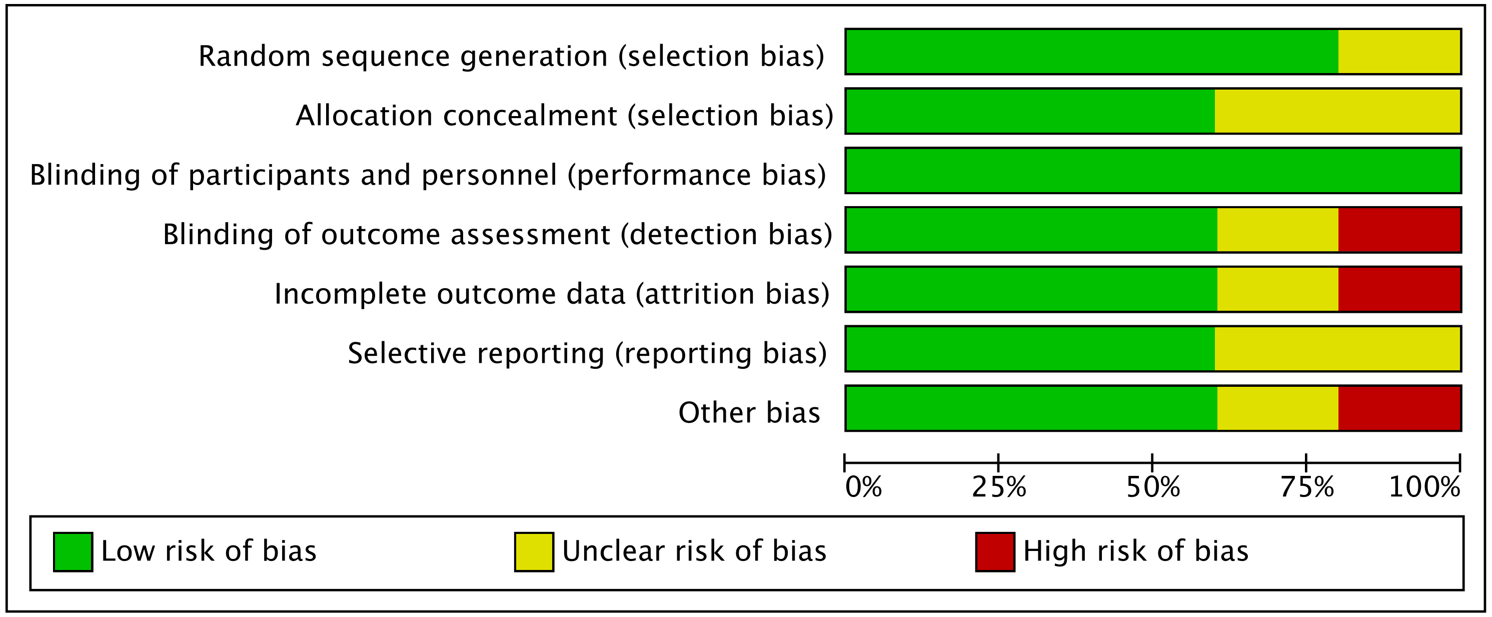

CQ12 Risk of bias summary, Risk of bias graph

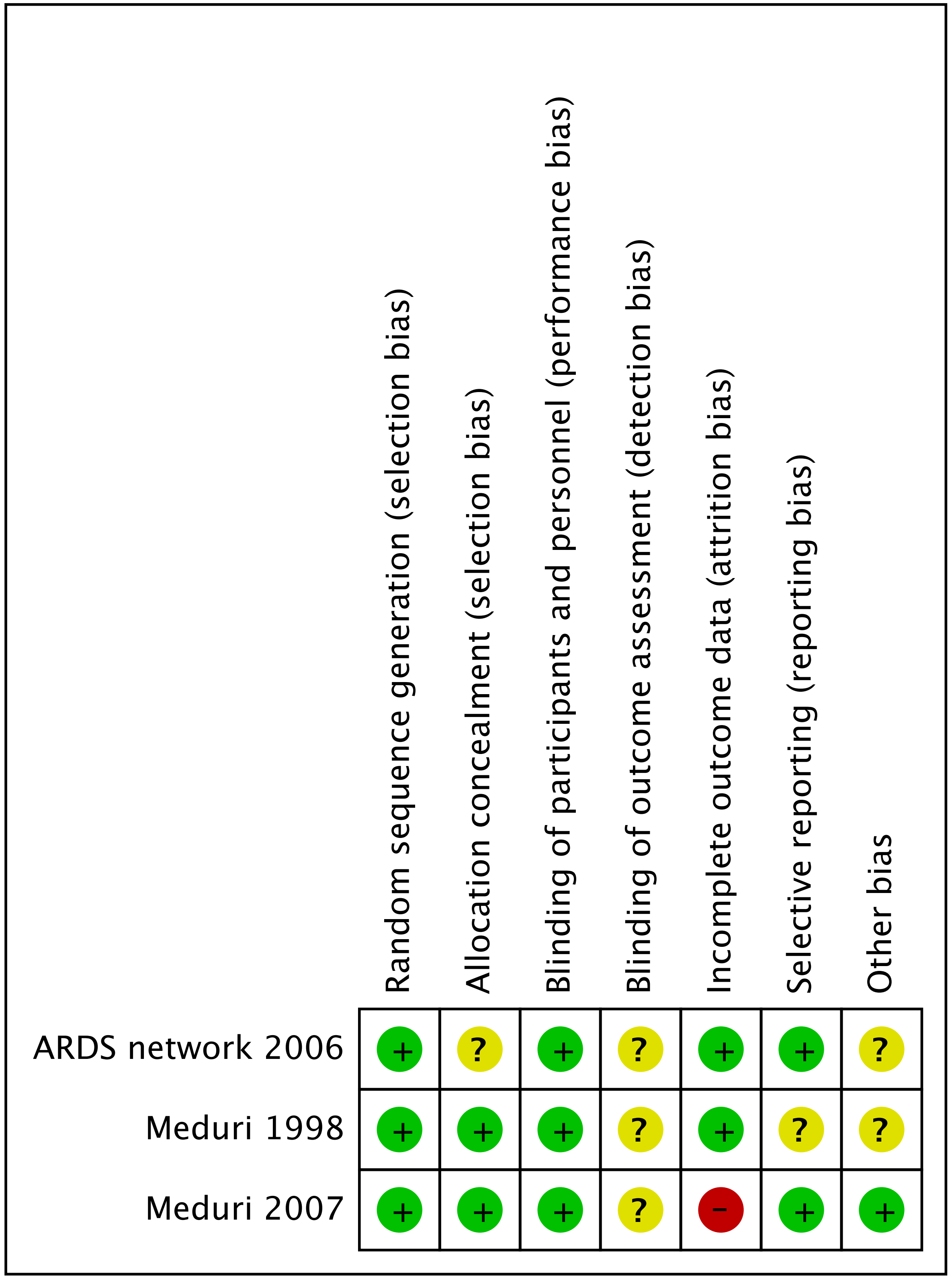

VFD

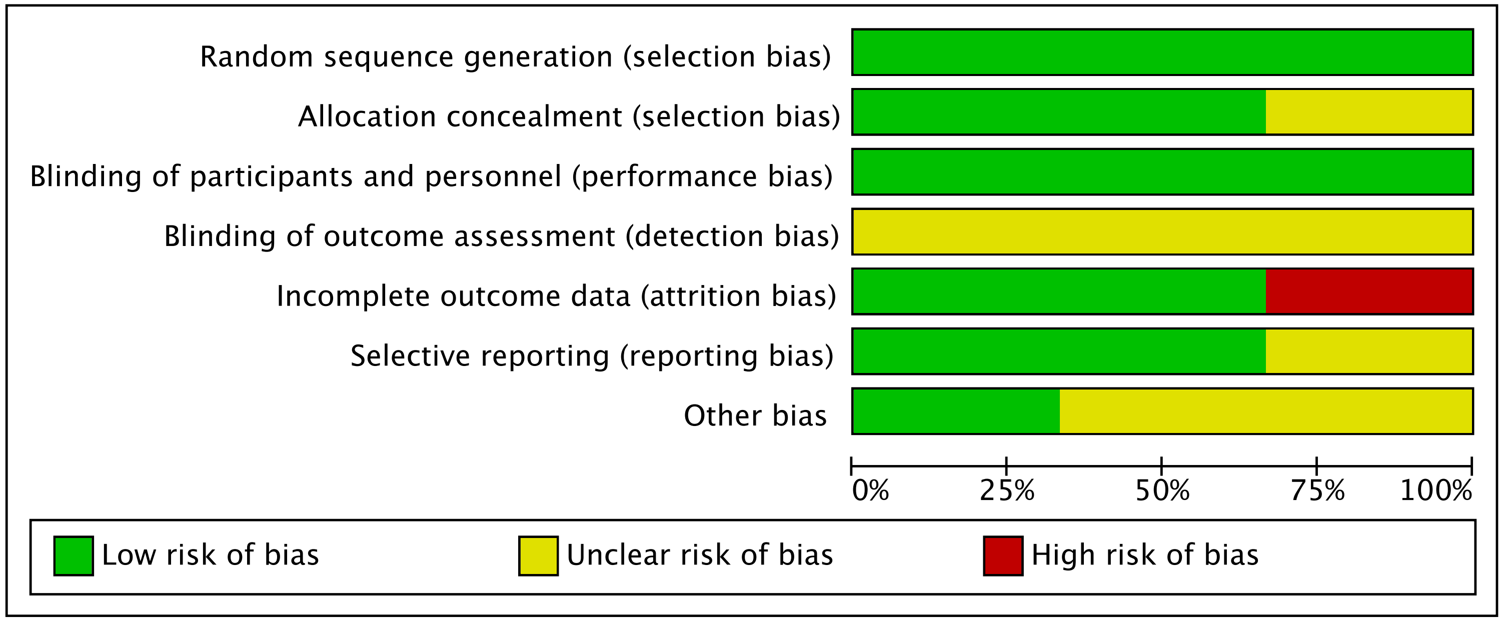

Short term mortality

Study or Subgroup	Steroid Events	Steroid Total	Placebo Events	Placebo Total	Weight	Risk Ratio M-H, Random, 95% CI
Annane 2006	54	85	67	92	37.8%	0.87 [0.71, 1.07]
ARDS network 2006	26	89	26	91	18.6%	1.02 [0.65, 1.62]
Bernard 1987	30	50	31	49	28.3%	0.95 [0.69, 1.29]
Meduri 1998	2	16	5	8	3.0%	0.20 [0.05, 0.81]
Meduri 2007	15	63	12	28	12.4%	0.56 [0.30, 1.03]
**Total (95% CI)**		**303**		**268**	**100.0%**	**0.83 [0.65, 1.07]**
Total events	127		141			

Heterogeneity: $Tau^2 = 0.03$; $Chi^2 = 7.10$, df = 4 (P = 0.13); $I^2 = 44\%$
Test for overall effect: Z = 1.44 (P = 0.15)

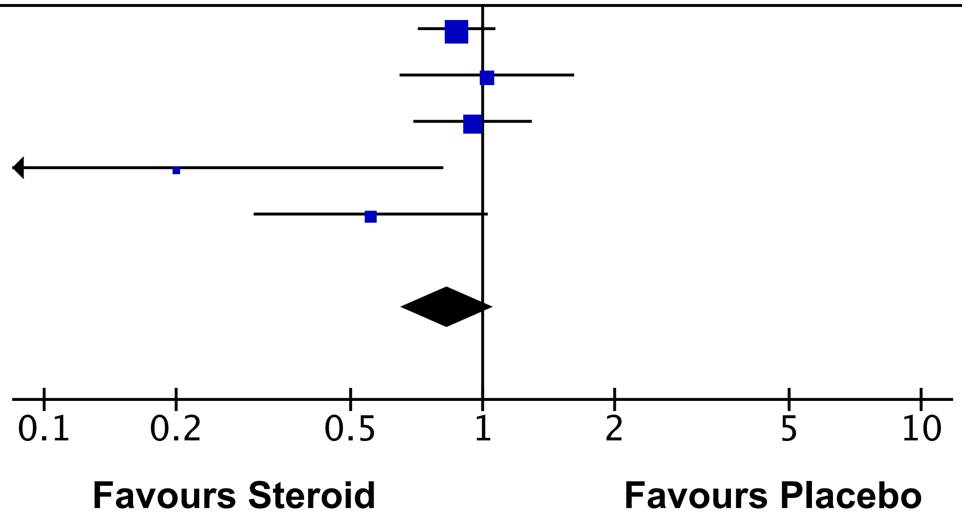

Infection

Study or Subgroup	Steroid Events	Steroid Total	Placebo Events	Placebo Total	Weight	Risk Ratio M-H, Random, 95% CI
Annane 2006	12	85	12	92	10.6%	1.08 [0.51, 2.28]
ARDS network 2006	20	89	30	91	25.0%	0.68 [0.42, 1.11]
Bernard 1987	8	50	5	49	5.4%	1.57 [0.55, 4.46]
Meduri 1998	12	16	6	8	24.5%	1.00 [0.61, 1.63]
Meduri 2007	27	63	17	28	34.5%	0.71 [0.47, 1.07]
**Total (95% CI)**		**303**		**268**	**100.0%**	**0.83 [0.65, 1.06]**
Total events	79		70			

Heterogeneity: $Tau^2 = 0.00$; $Chi^2 = 3.70$, df = 4 (P = 0.45); $I^2 = 0\%$
Test for overall effect: Z = 1.48 (P = 0.14)

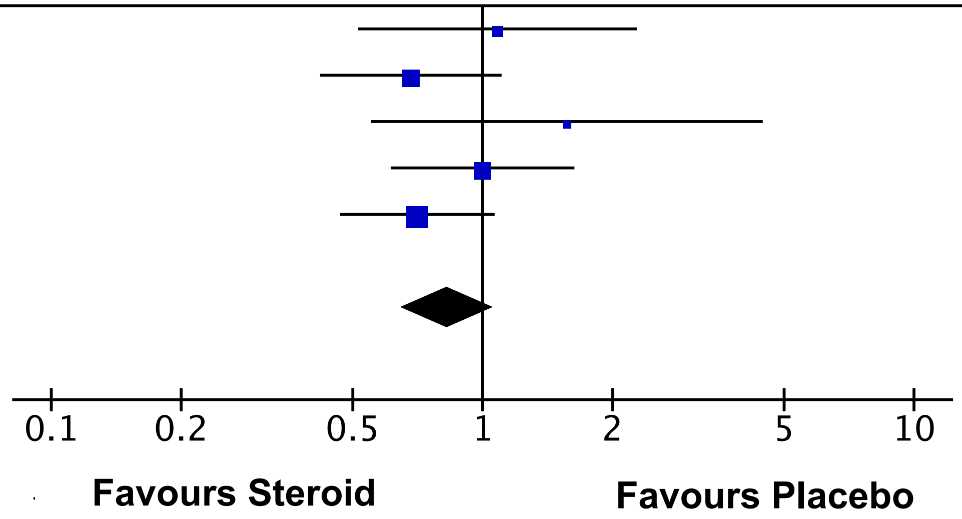

VFD

Study or Subgroup	Steroid Mean	Steroid SD	Steroid Total	Placebo Mean	Placebo SD	Placebo Total	Weight	Mean Difference IV, Random, 95% CI
ARDS network 2006	11.2	9.4	89	6.8	8.5	91	60.6%	4.40 [1.78, 7.02]
Meduri 1998	11	6.8	16	3.5	6.2	8	16.3%	7.50 [2.06, 12.94]
Meduri 2007	16.5	10.1	63	8.7	10.2	28	23.1%	7.80 [3.27, 12.33]
**Total (95% CI)**			**168**			**127**	**100.0%**	**5.69 [3.44, 7.94]**

Heterogeneity: $Tau^2 = 0.40$; $Chi^2 = 2.18$, df = 2 (P = 0.34); $I^2 = 8\%$
Test for overall effect: Z = 4.95 (P < 0.00001)

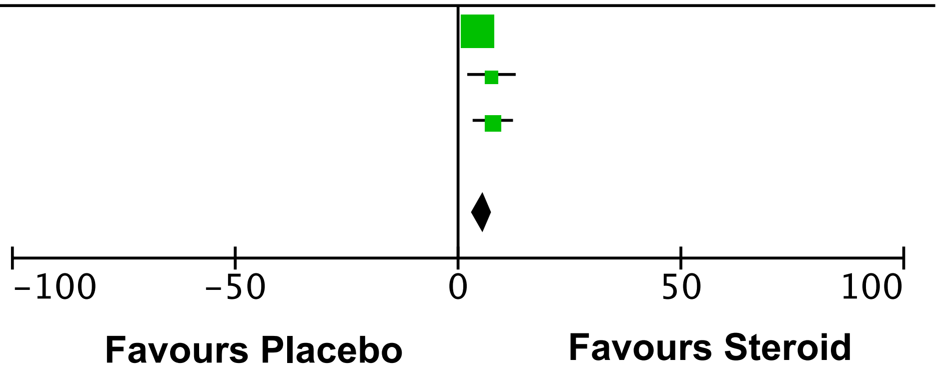

Summary of findings:

ARDS steroids compared to placebo for ARDS patients

Patient or population: ARDS patients
Setting:
Intervention: steroids
Comparison: placebo

Outcomes	Anticipated absolute effects* (95% CI)		Relative effect (95% CI)	№ of participants (studies)	Quality of the evidence (GRADE)	Comments
	Risk with placebo	Risk with ARDS steroids				
Short-term mortality	Study population		**RR 0.83** (0.65 to 1.07)	571 (5 RCTs)	⨁⨁⨁⨁ HIGH	
	526 per 1000	**437 per 1000** (342 to 563)				
	Low					
	238 per 1000	**198 per 1000** (155 to 255)				
	High					
	635 per 1000	**527 per 1000** (413 to 679)				
Infection	Study population		**RR 0.83** (0.65 to 1.06)	571 (5 RCTs)	⨁⨁⨁⨁ HIGH	
	261 per 1000	**217 per 1000** (170 to 277)				
	Low					
	141 per 1000	**117 per 1000** (92 to 149)				
	High					
	426 per 1000	**354 per 1000** (277 to 452)				
VFD	Mean 12.3 days	5.67 days more MD (3.49 more to 7.68 more)	-	295 (3 RCTs)	⨁⨁⨁⨁ HIGH	

***The risk in the intervention group** (and its 95% confidence interval) is based on the assumed risk in the comparison group and the **relative effect** of the intervention (and its 95% CI).

CI: Confidence interval; **RR:** Risk ratio; **MD:** Mean difference

GRADE Working Group grades of evidence
High quality: We are very confident that the true effect lies close to that of the estimate of the effect
Moderate quality: We are moderately confident in the effect estimate: The true effect is likely to be close to the estimate of the effect, but there is a possibility that it is substantially different
Low quality: Our confidence in the effect estimate is limited: The true effect may be substantially different from the estimate of the effect
Very low quality: We have very little confidence in the effect estimate: The true effect is likely to be substantially different from the estimate of effect

CQ12:
Question: Should steroids be used in adult patients with ARDS?

Quality assessment							№ of patients		Effect		Quality	Importance
**№ of studies**	**Study design**	**Risk of bias**	**Inconsistency**	**Indirectness**	**Imprecision**	**Other considerations**	**Steroid**	**Placebo**	**Relative (95% CI)**	**Absolute (95% CI)**		
Mortality (short-term)												
5	Randomized trials	Not serious	Not serious	Not serious	Not serious	None	127/303 (41.9%)	141/268 (52.6%)	**RR 0.83** (0.65 to 1.07)	89 fewer per 1000 (from 37 more to 1844 fewer)	⊕⊕⊕⊕ HIGH	CRITICAL
								23.8%		40 fewer per 1000 (from 17 more to 83 fewer)		
								63.5%		108 fewer per 1000 (from 44 more to 222 fewer)		
Incidence of infection												
5	Randomized trials	Not serious	Not serious	Not serious	Not serious	None	79/303 (26.1%)	70/268 (26.1%)	**RR 0.83** (0.65 to 1.06)	50 fewer per 1000 (from 38 more to 117 fewer)	⊕⊕⊕⊕ HIGH	CRITICAL
								14.1%		27 fewer per 1000 from 20 more to 62 fewer)		
								42.6%		52 fewer per 1000 (from 40 more to 123 fewer)		
VFD												
3	Randomized trials	Not serious	Not serious	Not serious	Not serious	None	127	168	-	MD 5.59 days more (from 3.49 more to 7.68 more)	⊕⊕⊕⊕ HIGH	CRITICAL

CI: Confidence interval, RR: Risk ratio, MD: Mean difference

Evidence-to-Dicision table

CQ12 : Should steroids be used in adult patients with ARDS?

POPULATION : ADULT PATIENTS WITH ARDS

INTERVENTION : ADMINISTRATION OF STEROIDS

	CRITERIA	CRITERIA	CRITERIA	ADDITIONAL CONSIDERATIONS
PROBLEM	**Is the problem a priority?**	◯No<br>◯Probably no<br>◯Probably yes<br>●Yes<br>------------------<br>◯Varies<br>◯Don't know	ARDS is defined as non-cardiogenic pulmonary edema, which may be caused by increased permeability due to epithelial and endothelial cell damage and neutrophil infiltration[1, 2]. Steroids, as anti-inflammatory therapy, may improve the pathologic changes associated with ARDS and a number of studies have assessed the risks and benefits of their use [3]. However, steroid therapy also has the potential to be detrimental to patients, and there is concern regarding an increased risk of infection. Therefore, this issue is a high priority in the management of adult patients with ARDS.	
DESIRABLE AND UNDESIRABLE EFFECTS	**What is the overall certainty of the evidence of effects?**	◯Very low<br>◯Low<br>◯Moderate<br>●High<br>------------------<br>◯No included studies	**The relative importance or values of the main outcomes of interest:** (see table below)	
	**Is there important uncertainty about or variability in how much people value the main outcomes?**	◯Important uncertainty or variability<br>◯Possibly important uncertainty or variability<br>◯Possibly no important uncertainty or variability<br>●No important uncertainty or variability<br>------------------<br>◯No known undesirable outcomes		
	**How substantial are the desirable anticipated effects?**	◯Trivial<br>●Small<br>◯Moderate<br>◯Large<br>------------------<br>◯Varies<br>◯Don't know	**Summary of findings:** (see table below)	
	**How substantial are the undesirable anticipated effects?**	◯Large<br>◯Moderate<br>◯Small<br>●Trivial<br>------------------<br>◯Varies<br>◯Don't know		
	**Does the balance between desirable and undesirable effects favor the intervention or**	◯Favors the comparison<br>◯Probably favors the comparison<br>◯Does not favor either the intervention or the comparison<br>●Probably favors		

The relative importance or values of the main outcomes of interest:

Outcome	Relative importance	Certainty of the evidence (GRADE)
Short term mortality (Note 1)	CRITICAL	⊕⊕⊕⊕<br>HIGH
The incidence of infection	CRITICAL	⊕⊕⊕⊕<br>HIGH
VFD (Note 2)	CRITICAL	⊕⊕⊕⊕<br>HIGH

Summary of findings:

Outcome	Placebo	Steroid	Absolute effect (95% CI)	Relative effect (RR) (95% CI)
Mortality (short-term)	526 / 1000	437 / 1000 (342 to 563)	89 fewer/1000 (from 37 more to 184 fewer)	**RR 0.83** (0.65 to 1.07)
	238 / 1000	198 / 1000 (155 to 255)	40 fewer/1000 (from 17 more to 83 fewer)	
	635 / 1000	527 / 1000 (413 to 679)	108 fewer/1000 (from 44 more to 222 fewer)	
The incidence of infection	261 / 1000	217 / 1000 (170 to 277)	44 fewer/1000 (from 16 more To 91 fewer)	**RR 0.83** (0.65 to 1.06)
	141 / 1000	117 / 1000 (92 to 149)	24 fewer/1000 (from 8 more to 49 fewer)	
	426 / 1000	354 / 1000 (277 to 452)	72 fewer/1000 (from 26 more to 149 fewer)	

<table>
<tr><td></td><td>the comparison?</td><td>the intervention
◯Favors the intervention

◯Varies
◯Don’t know</td><td><table><tr><td>VFD</td><td>Average 6.7 days</td><td>Average 12.3 days</td><td>MD 5.67 more (from 3.49 more to 7.68 more)</td><td>-</td></tr></table>Summary: Steroid administration did not significantly decrease the mortality in comparison with placebo. However, it significantly increased number of ventilator free days (VFD). In addition, steroid therapy did not significantly increase the incidence of infection.
Randomized controlled trials to assess the number of VFD were evaluated the effect of methylprednisolone 1-2mg/kg/day. An RCT conducted by Bernard et al showed that steroid therapy (methylprednisolone 120mg/kg/day) had a trend to increase the incidence of infection (odds ratio=1.57).</td><td></td></tr>
<tr><td rowspan="2">RESOURCES REQUIRED</td><td>How large are the resource requirements (costs)?</td><td>◯Large costs
◯Moderate costs
◯Negligible costs and savings
●Moderate savings
◯Large savings

◯Varies
◯Don’t know</td><td>Steroid therapy is not expensive. If one assumes four weeks of treatment, the cost is expected to be 12,000 -30,000 JPY. Steroids should be available in a majority of hospitals.</td><td></td></tr>
<tr><td>Does the cost-effectiveness of the intervention favor the intervention or the comparison?</td><td>◯Favors the comparison
◯Probably favors the comparison
◯Does not favor either the intervention or the comparison
●Probably favors the intervention
◯Favors the intervention

◯Varies
◯No included studies</td><td>Steroid therapy is not expensive. If one considers the effect on VFD, the cost is less than the benefit.</td><td></td></tr>
<tr><td>EQUITY</td><td>What would be the impact on health equity?</td><td>◯Reduced
◯Probably reduced
◯Probably no impact
◯Probably increased
●Increased

◯Varies
◯Don’t know</td><td>Special medical facilities or equipment are not required for this treatment.</td><td></td></tr>
<tr><td>ACCEPTABILITY</td><td>Is the intervention acceptable to key stakeholders?</td><td>◯No
◯Probably no
●Probably yes
◯Yes

◯Varies
◯Don’t know</td><td></td><td></td></tr>
<tr><td>FEASIBILITY</td><td>Is the intervention feasible implement?</td><td>◯No
◯Probably no
◯Probably yes
●Yes

◯Varies
◯Don’t know</td><td>Special medical facilities or equipment are not required for this procedure..</td><td></td></tr>
</table>

Note 1) short term mortality defined as within 90days and treated as main outcome in each study.
Note 2) VFD means the number of days free from mechanical ventilation in the initial 28 days. If the patient expired within 28 days, VFD was counted as zero.

Recommendation

CQ12 : **Should steroids be used in adult patients with ARDS?**					
**Balance of consequences**	Undesirable consequences *cl*early outweigh desirable consequences in most settings	Undesirable consequences probably outweigh desirable consequences in most settings	The balance between desirable and undesirable consequences is closely balanced or uncertai*n*	Desirable consequences probably outweigh undesirable consequences in most settings	Desirable consequences clearly outweigh undesirable consequences in most settings
Judgement	○	○	○	●	○

**Type of recommendation**	Strong recommendation against the intervention	Conditional recommendation against the intervention	Conditional recommendation for either the intervention or the comparison	Conditional recommendation for the intervention
Judgement	○	○	●	○

Recommendation

We suggest the administration of steroids (equivalent to methylprednisolone 1-2mg/kg/ day) to adult patients with ARDS. (GRADE 2A , strength of recommendation " weak recommendation" / Quality of evidence "high")

Justification

Question: Should steroids be used in adult patients with ARDS?

Patients: Adult patients with ARDS

Interventions: administration of steroids

Comparison: Placebo

Outcomes: Short-term mortality[(Note 1)], the incidence of infection, the number of ventilator free days (VFD)[(Note 2)]

Summary of the evidence: There are a number of randomized controlled trials of steroid therapy in patients with ARDS, including the effect of low to middle dose steroids as such methylprednisolone 1-2mg/kg/day or hydrocortisone 200mg/day, and high doses such as methylprednisolone 120mg/kg/day. Steroid administration did not significantly decrease the mortality compared to placebo. However, it significantly increased the number of ventilator free days (VFD). In addition, steroid therapy did not significantly increase the incidence of infection. All randomized controlled trials to assess the VFD evaluated the effect of methylprednisolone 1-2mg/kg/day. In an RCT conducted by Bernard et al, it was shown that steroid therapy (methylprednisolone 120mg/kg/day) had a trend to increase the incidence of infection (odds ratio=1.57)

Quality of the evidence: We collected RCTs to propose a recommendation on this issue. For each outcome including mortality, the risk of infection and VFD, there was a low risk of bias regarding non-consistency, non-immediateness, and non-precision. There are few studies, and the publication bias is unclear. Therefore, the overall quality of evidence across outcomes is considered to be "high".

Judgement of benefit and harm, resources and cost: Steroid therapy is feasible in almost all facilities in Japan. Unfortunately, it is not evaluated in each study, but it is possible to delay the diagnosis of infections by administering steroids. In addition, a risk of side effects (hyperglycemia, infection, etc.) is associated with this treatment. There is also concern regarding ICU-related muscle weakness due to steroid use. Steroid therapy is feasible in almost all facilities in Japan. There is no significant reduction of mortality with steroid therapy. Steroid therapy did not have a tendency to increase the risk of infection, and is expected to increase the number of VFD. Unfortunately, it is not evaluated in each study, but it is possible to delay the diagnosis of infections by using steroid. In addition, a risk of side effects (hyperglycemia, infection, etc.) is associated with this treatment. There is also concern regarding ICU-related muscle weakness due to steroid use.

In the first panel meeting, both mortality and the incidence of infection were reported. The committee proposed "**We suggest not to use steroids in adult patients with ARDS".** Since not all participants agreed with this recommendation, a vote was conducted; one person supported the suggestion to use steroids, and eleven supported the suggestion not to use steroids. One person who supported not using steroids, had a specific concern regarding a lack of effect on the number of VFD. Therefore, the results regarding VFD as the outcome was reported again. After reporting the additional results about VFD, it was proposed that "**We suggest the use of steroids in adult patients with ARDS".** A second vote was conducted. Ten people supported the suggestion to support using steroids, and two supported the suggestion not to use steroids. The opinions raised included "importance of VFD should not be high as mortality" and "the side effect of steroids which is not evaluate in SR was not negligible."

The authors of RCTs which reported the benefit of steroids in adults with ARDS, reported that the delayed use of steroids, such as 14 days after the onset of ARDS, would be associated with increased mortality, thus, it was suggested to use steroids during the early stage of ARDS[4)].

In a domestic survey conducted by a Japanese respiratory society in 2010, it was reported that many Japanese doctors formerly gave 500-1,000 mg/day of methylprednisolone to patients with ARDS. However, there is no RCT

	which examines the risk or benefit of this therapy, and thus it could not be assessed. Accordingly, future studies are required to assess the impact of the dose or timing of steroid therapy in this cohort.<br><br>Result of two votes by the panel<br>Since there was no unanimous consensus, votes were conducted.

Strength of recommendation	Strong	Weak	Weak	Strong
Recommendation	Recommend to use steroid	Suggest to use steroid	Suggest not to use steroid	Recommend not to use steroid
First vote Without information of VFD	0	1	11	0
First vote With information of VFD	0	10	2	0

In the second vote after reporting information regarding VFD, 10 people (83%) supported the recommendation to "suggest the use of steroids". The panel finally concluded to recommend it. However, the panel also showed concerns such as "importance of VFD should not be high as mortality" and "the side effect of steroid which is not evaluate in SR was not negligible.".

Recommendations; We suggest the administration of steroids (equivalent to methylprednisolone 1-2mg/kg/day) to adult patients with ARDS. (GRADE 2A, strength of recommendation " weak recommendation" / Quality of evidence "high")

Additional considerations: None

**Subgroup considerations**	None
**Implementation considerations**	It is possible to delay the diagnosis of infections by administering steroids. In addition, a risk of side effects (hyperglycemia, infection, etc.) is associated with this treatment. There is also concern regarding ICU-related muscle weakness due to steroid use.
**Monitoring and evaluation considerations**	Standard monitoring is sufficient. Careful evaluation for the development of secondary infections is required.
**Research possibilities**	It is possible that the effects of steroid therapy in adult patients with ARDS may be different according to the timing of initiating therapy, dose, duration of treatment and the manner of tapering the dose. Thus, future RCTs are necessary in consideration of these points as well.

Note 1) short term mortality defined as within 90days and treated as main outcome in each study.
Note 2) VFD means the number of days free from mechanical ventilation in the initial 28 days. If the patient expired within 28 days, VFD was counted as zero.

1. Kollef MH, Schuster DP. The acute respiratory distress syndrome. *The New England journal of medicine* **332**(1): 27-37, 1995. PMID 7646623
2. Force ADT, Ranieri VM, Rubenfeld GD, et al. Acute respiratory distress syndrome: the Berlin Definition. *Jama* **307**(23): 2526-33, 2012. PMID 22797452
3. Thompson BT. Glucocorticoids and acute lung injury. *Critical care medicine* **31**(4 Suppl): S253-7, 2003. PMID 12682449
4. Meduri GU, Marik PE, Chrousos GP, et al. Steroid treatment in ARDS: a critical appraisal of the ARDS network trial and the recent literature. *Intensive care medicine* **34**(1): 61-9, 2008. PMID 18000649

CQ13. Study flow diagram

7687 records identified through database searching

Medline（PubMed）	3575
Embase	3813
Cochrane CENTRAL	235
Ichushi	64

Additional record identified through other sources

Hand-search 7

7694 records identified

1193 duplicates

6501 records after duplicates removed

6429 records excluded

Other than English and Japanese	7
Non-RCT	4735
Different patients /interventions /comparisons	1684
Duplicate	3

72 full-text articles assessed for eligibility

The following full-text articles were excluded with

	total	※1	※2	※3
CQ13-01	65	54	8	3
CQ13-02	71	60	8	3
CQ13-03	70	59	8	3
CQ13-04	70	59	8	3
CQ13-05	65	54	8	3
CQ13-06	69	58	8	3
CQ13-07	62	51	8	3
CQ13-08	70	59	8	3
CQ13-09	68	57	8	3
CQ13-10	71	60	8	3
CQ13-11	71	60	8	3

※1 Different interventions/ comparisons
※2 Non-RCT
※3 Duplicate

The following articles were included in meta analysis

CQ13-01	n=7
CQ13-02	n=1
CQ13-03	n=2
CQ13-04	n=2
CQ13-05	n=7
CQ13-06	n=3
CQ13-07	n=10
CQ13-08	n=2
CQ13-09	n=4
CQ13-10	n=1
CQ13-11	n=1

	Total	Short-term Mortality	Severe adverse effects
CQ13-01	n=7	n=7	n=2
CQ13-02	n=1	n=1	n=1
CQ13-03	n=2	n=2	n=2
CQ13-04	n=2	n=2	n=1
CQ13-05	n=7	n=7	n=4
CQ13-06	n=3	n=2	n=2
CQ13-07	n=10	n=9	n=6
CQ13-08	n=2	n=2	n=2
CQ13-09	n=4	n=4	n=0
CQ13-10	n=1	n=1	n=1
CQ13-11	n=1	n=1	n=0

CQ13-01

Risk of bias table, mortality

Outcome		Short term mortality		risk of bias		serious (−1)			
番号	著者名 発表年 (Forest plot表示)	risk of bias評価							
		ランダム割付順番の生成 random sequence generation	割り付けの隠蔽化 allocation concealment	ブラインド blinding		不完全なアウトカムデータ incomplete outcome data	選択されたアウトカムの報告 selective outcome reporting	その他のバイアス Other sources of bias	研究内でのバイアスのリスク Risk of bias within a study
				研究参加者と治療提供者 participants and personnel	アウトカム評価者 outcome assessors				
1	Dellinger 1998	Unclear risk	Unclear risk	Low risk	Low risk	Low risk	Unclear risk	High risk	Unclear risk
2	Gerlach 2003	Low risk	Low risk	High risk	High risk	Low risk	Unclear risk	Low risk	Unclear risk
3	Mehta 2001	Low risk	Low risk	High risk	High risk	Low risk	Unclear risk	Unclear risk	Unclear risk
4	Michael 1998	Unclear risk	Unclear risk	High risk	Low risk	Unclear risk	Low risk	Low risk	Unclear risk
5	Park 2003	High risk	High risk	High risk	High risk	Low risk	Unclear risk	Low risk	High risk
6	Taylor 2004	Low risk	Low risk	Low risk	Low risk	Low risk	Unclear risk	High risk	Low risk
7	Troncy 1998	Unclear risk	Unclear risk	High risk	High risk	Low risk	Low risk	Unclear risk	Unclear risk

番号	著者名 発表年 (Forest plot表示)	risk of biasコメント							
1	Dellinger 1998	記載なし	記載なし	盲検化を保つために、各施設に非盲検の研究者をおいた	盲検化を保つために、各施設に非盲検の研究者をおいた	約98%フォローされた	事前に計画されたプロトコールが閲覧できなかった	32%で介入を最後まで全うできなかった	LowとUnclearが同数で、Highが1項目あり、Unclearとした
2	Gerlach 2003	外観からわからない抽選	外観からわからない抽選	盲検化されていない	盲検化されていない	100%フォローされた	事前に計画されたプロトコールが閲覧できなかった	研究の中断なし	High 2項目のため、Unclearとした
3	Mehta 2001	computer-generated random number sequenceによってランダム化	computer-generated random number sequenceによって割り付けの隠蔽化	研究参加者、治療提供者ともブラインド化されていない	アウトカム評価者もブラインド化されていない	データの欠損がない	研究プロトコールが利用できない	一部、企業からの資金提供がある	low3項目、high2項目
4	Michael 1998	ランダム化の詳細が未記載	割り付けはブロック法を用いているが、ブラインド化されてないため予測できた可能性がある	研究参加者、治療提供者ともブラインド化されていない	ブラインド化されていないが、影響を受けない	院内死亡のみ記載	研究プロトコールが利用不可能だが、問題ない	他のbiasがない	low3項目、high1項目
5	Park 2003	完全に無作為化にはできなかった	完全に無作為化にはできなかった	対照群は非使用群	対照群は非使用群	100%フォロー	事前に計画されたプロトコールが閲覧できなかった	その他のバイアスは指摘できなかった	Highが4項目ありHighとした
6	Taylor 2004	中央でランダム化	中央割り付け	研究参加者、治療提供者(医師、看護師、呼吸療法士)ともブラインド化されている	アウトカム評価者もブラインド化されている	データの欠損がない	評価するための十分な情報がない	企業からの資金提供がある、protocolが守られていない症例が計31例あった	low5項目、high1項目
7	Troncy 1998	ランダム化の方法が未記載	割り付けの方法が未記載	研究参加者、治療提供者ともブラインド化されていない	アウトカム評価者もブラインド化されていない	データの欠損がない	研究プロトコールが利用不可能だが、本outcomeを含んでいる	評価するための十分な情報がない	low2項目、high2項目

CQ13_01
Risk of bias table, Severe adverse effects

Outcome		Severe adverse effects		risk of bias		not serious (0)			
番号	著者名 発表年（Forest plot表示）	risk of bias評価							
		ランダム割付順番の生成 random sequence generation	割り付けの隠蔽化 allocation concealment	ブラインド blinding		不完全なアウトカムデータ incomplete outcome data	選択されたアウトカムの報告 selective outcome reporting	その他のバイアス Other sources of bias	研究内でのバイアスのリスク Risk of bias within a study
				研究参加者と治療提供者 participants and personnel	アウトカム評価者 outcome assessors				
1	Dellinger 1998	Unclear risk	Unclear risk	Low risk	Low risk	Low risk	Unclear risk	High risk	Unclear risk
6	Taylor 2004	Low risk	Low risk	Low risk	Low risk	Low risk	Unclear risk	High risk	Low risk
番号	著者名 発表年（Forest plot表示）	risk of biasコメント							
1	Dellinger 1998	記載なし	記載なし	盲検化を保つために、各施設に非盲検の研究者をおいた	盲検化を保つために、各施設に非盲検の研究者をおいた	約98%フォローされた	事前に計画されたプロトコールが閲覧できなかった	32%で介入を最後まで全うできなかった	LowとUnclearが同数で、Highが1項目あり、Unclearとした
6	Taylor 2004	中央でランダム化	中央割り付け	研究参加者、治療提供者（医師、看護師、呼吸療法士）ともブラインド化されている	アウトカム評価者もブラインド化されている	データの欠損がない	評価するための十分な情報がない	企業からの資金提供がある、protocolが守られていない症例が計31例あった	low5項目、high1項目

CQ13_02
Risk of bias table, mortality

Outcome		Short term mortality		risk of bias		not serious (0)			
番号	著者名 発表年 (Forest plot表示)	risk of bias評価							
		ランダム割付順番の生成 random sequence generation	割り付けの隠蔽化 allocation concealment	ブラインド blinding		不完全なアウトカムデータ incomplete outcome data	選択されたアウトカムの報告 selective outcome reporting	その他のバイアス Other sources of bias	研究内でのバイアスのリスク Risk of bias within a study
				研究参加者と治療提供者 participants and personnel	アウトカム評価者 outcome assessors				
1	Matthay 2011	Low risk	Low risk	Low risk	Low risk	Low risk	Low risk	Low risk	Low risk
番号	著者名 発表年 (Forest plot表示)	risk of biasコメント							
1	Matthay 2011	中央割り付けで、インターネットによるシステムでランダム化	中央割り付けで、インターネットによるシステムでランダム化	研究参加者、治療提供者ともブラインド化されている	アウトカム評価者もブラインド化されている	データの欠損がない	研究プロトコールが利用でき決められたアウトカムの報告がなされている	他のバイアスがない	low7項目、high0項目

CQ13_02
Risk of bias table, Severe adverse effects

Outcome		Severe adverse effects		risk of bias		not serious (0)			
番号	著者名 発表年（Forest plot表示）	risk of bias評価							
		ランダム割付順番の生成 random sequence generation	割り付けの隠蔽化 allocation concealment	ブラインド blinding		不完全なアウトカムデータ incomplete outcome data	選択されたアウトカムの報告 selective outcome reporting	その他のバイアス Other sources of bias	研究内でのバイアスのリスク Risk of bias within a study
				研究参加者と治療提供者 participants and personnel	アウトカム評価者 outcome assessors				
1	Matthay 2011	Low risk	Low risk	Low risk	Low risk	Low risk	Low risk	Low risk	Low risk
番号	著者名 発表年（Forest plot表示）	risk of biasコメント							
1	Matthay 2011	中央割り付けで、インターネットによるシステムでランダム化	中央割り付けで、インターネットによるシステムでランダム化	研究参加者、治療提供者ともブラインド化されている	アウトカム評価者もブラインド化されている	データの欠損がない	研究プロトコールが利用でき決められたアウトカムの報告がなされている	他のバイアスがない	low7項目、high0項目

CQ13_03
Risk of bias table, mortality

Outcome		Short term mortality		risk of bias		not serious (0)			
番号	著者名 発表年<br>（Forest plot表示）	risk of bias評価							
		ランダム割付順番の生成<br>random sequence generation	割り付けの隠蔽化<br>allocation concealment	ブラインド blinding<br>研究参加者と治療提供者<br>participants and personnel	アウトカム評価者<br>outcome assessors	不完全なアウトカムデータ<br>incomplete outcome data	選択されたアウトカムの報告<br>selective outcome reporting	その他のバイアス<br>Other sources of bias	研究内でのバイアスのリスク<br>Risk of bias within a study
1	Gao 2012	Low risk	Low risk	Low risk	Low risk	Low risk	Low risk	Low risk	Low risk
2	Perkins 2006	Unclear risk	Unclear risk	Low risk	Low risk	Low risk	Unclear risk	Unclear risk	Unclear risk
番号	著者名 発表年<br>（Forest plot表示）	risk of biasコメント							
1	Gao 2012	Computer-generated randomizationを使用し、適切にランダム化された。	Central telephone か web-based randomisation serviceを使用し、適切に隠蔽化された。	割り付けを研究参加者と治療提供者は知らされていなかった。	割り付けを評価者は知らされていなかった。	ほぼ100%フォローされた	100%報告された	他のbiasなし（研究の中断あるも問題なし）	全ての項目がLow risk
2	Perkins 2006	ランダム化の方法の情報が不十分	割り付けの方法が未記載	盲検化されていた。	盲検化されていた。	100%フォローされた。	評価するには不十分な情報	評価するには不十分な情報	Unclearが多い

CQ13_03
Risk of bias table, Severe adverse effects

Outcome		Severe adverse effects		risk of bias		not serious (0)			
番号	著者名 発表年（Forest plot表示）	risk of bias評価							
		ランダム割付順番の生成 random sequence generation	割り付けの隠蔽化 allocation concealment	ブラインド blinding		不完全なアウトカムデータ incomplete outcome data	選択されたアウトカムの報告 selective outcome reporting	その他のバイアス Other sources of bias	研究内でのバイアスのリスク Risk of bias within a study
				研究参加者と治療提供者 participants and personnel	アウトカム評価者 outcome assessors				
1	Gao 2012	Low risk	Low risk	Low risk	Low risk	Low risk	Low risk	Low risk	Low risk
2	Perkins 2006	Unclear risk	Unclear risk	Low risk	Low risk	Low risk	Unclear risk	Unclear risk	Unclear risk
番号	著者名 発表年（Forest plot表示）	risk of biasコメント							
1	Gao 2012	Computer-generated randomizationを使用し、適切にランダム化された。	Central telephone か web-based randomisation serviceを使用し、適切に隠蔽化された。	割り付けを研究参加者と治療提供者は知らされていなかった。	割り付けを評価者は知らされていなかった。	ほぼ100%フォローされた	100%報告された	他のbiasなし（研究の中断あるも問題なし）	全ての項目がLow risk
2	Perkins 2006	ランダム化の方法の情報が不十分	割り付けの方法が未記載	盲検化されていた。	盲検化されていた。	100%フォローされた。	評価するには不十分な情報	評価するには不十分な情報	Unclearが多い

CQ13_04
Risk of bias table, mortality

Outcome		Short term mortality		risk of bias		not serious (0)			
番号	著者名 発表年（Forest plot表示）	risk of bias評価							
		ランダム割付順番の生成 random sequence generation	割り付けの隠蔽化 allocation concealment	ブラインド blinding		不完全なアウトカムデータ incomplete outcome data	選択されたアウトカムの報告 selective outcome reporting	その他のバイアス Other sources of bias	研究内でのバイアスのリスク Risk of bias within a study
				研究参加者と治療提供者 participants and personnel	アウトカム評価者 outcome assessors				
1	Paine 2012	Low risk	Low risk	Low risk	Low risk	Low risk	Low risk	High risk	Low risk
2	Presneill 2002	Unclear risk	Unclear risk	Low risk	Low risk	Low risk	Unclear risk	Unclear risk	Unclear risk
番号	著者名 発表年（Forest plot表示）	risk of biasコメント							
1	Paine 2012	ブロック化無作為で行われた	封筒法で行われた	研究期間中は治療および結果について研究者や臨床家には盲検化された	研究期間中は治療および結果について研究者や臨床家には盲検化された	問題になる脱落なし（2例除外された）	100%報告された	症例の登録が遅く、予定より早期に中止された	Lowがほとんどであり、Lowとした
2	Presneill 2002	ランダム化の方法について記載がなかった	ランダム化の方法について記載がなかった	薬は企業によってあらかじめ準備され、研究者にはデータ収集が終わるまでは明らかにされなかった	薬は企業によってあらかじめ準備され、研究者にはデータ収集が終わるまでは明らかにされなかった	脱落なし	研究プロトコールが閲覧できなかった	介入で高齢であったが、サンプル数が少ない。<br>評価するのに十分な情報がなかった。	Unclearの項目が多かった

CQ13_04
Risk of bias table, Severe adverse effects

Outcome		Severe adverse effects		risk of bias		not serious (0)			
番号	著者名 発表年（Forest plot表示）	risk of bias評価							
		ランダム割付順番の生成 random sequence generation	割り付けの隠蔽化 allocation concealment	ブラインド blinding		不完全なアウトカムデータ incomplete outcome data	選択されたアウトカムの報告 selective outcome reporting	その他のバイアス Other sources of bias	研究内でのバイアスのリスク Risk of bias within a study
				研究参加者と治療提供者 participants and personnel	アウトカム評価者 outcome assessors				
1	Paine 2012	Low risk	Low risk	Low risk	Low risk	Low risk	Low risk	High risk	Low risk
番号	著者名 発表年（Forest plot表示）	risk of biasコメント							
1	Paine 2012	ブロック化無作為で行われた	封筒法で行われた	研究期間中は治療および結果について研究者や臨床家には盲検化された	研究期間中は治療および結果について研究者や臨床家には盲検化された	問題になる脱落なし（2例除外された）	100%報告された	症例の登録が遅く、予定より早期に中止された	Lowがほとんどであり、Lowとした

CQ13_05
Risk of bias Table, mortality

Outcome		Short term mortality		risk of bias		not serious (0)			
番号	著者名 発表年（Forest plot表示）	risk of bias評価							
		ランダム割付順番の生成 random sequence generation	割り付けの隠蔽化 allocation concealment	ブラインド blinding		不完全なアウトカムデータ incomplete outcome data	選択されたアウトカムの報告 selective outcome reporting	その他のバイアス Other sources of bias	研究内でのバイアスのリスク Risk of bias within a study
				研究参加者と治療提供者 participants and personnel	アウトカム評価者 outcome assessors				
1	Abraham 1996	Unclear risk	Unclear risk	Low risk	Low risk	Low risk	Unclear risk	Unclear risk	Unclear risk
2	Abraham 1999	Unclear risk	Unclear risk	Low risk	Low risk	Low risk	Unclear risk	Unclear risk	Unclear risk
3	Bone 1989	Low risk	Low risk	Low risk	Low risk	Low risk	Unclear risk	Unclear risk	Low risk
4	Holcroft 1986	Low risk	Low risk	Low risk	Low risk	Low risk	Unclear risk	Unclear risk	Low risk
5	Rossignon 1990	Unclear risk	Unclear risk	Low risk	Low risk	Low risk	Unclear risk	Low risk	Low risk
6	Slotman 1992	Low risk	Low risk	Low risk	Low risk	Low risk	Low risk	Low risk	Low risk
7	Vincent 2001	Unclear risk	Unclear risk	Low risk	Low risk	Low risk	Unclear risk	Low risk	Low risk

番号	著者名 発表年（Forest plot表示）	risk of biasコメント							
1	Abraham 1996	ランダム化の詳細は未記載	割り付けの詳細は不明だが、薬剤師が薬剤のシリンジおよびチューブを覆い隠蔽した	研究参加者、治療提供者ともブラインド化されている	アウトカム評価者もブラインド化されている	データの欠損がない	評価するための十分な情報がない	評価するための十分な情報がない	low3項目、high0項目
2	Abraham 1999	ランダム化の詳細は未記載	割り付けの詳細は不明だが、薬剤師が薬剤のシリンジおよびチューブを覆い隠蔽した	研究参加者、治療提供者ともブラインド化されている	アウトカム評価者もブラインド化されている	データの欠損がない	評価するための十分な情報がない	評価するための十分な情報がない	low3項目、high0項目
3	Bone 1989	中央割り付けにてランダム化	中央割り付けにてランダム化	研究参加者、治療提供者ともブラインド化されている	アウトカム評価者もブラインド化されている	データの欠損がない	評価するための十分な情報がない	評価するための十分な情報がない	low5項目、high0項目
4	Holcroft 1986	コンピューターによりランダム化	コンピューターにより割り付け	研究参加者、治療提供者ともブラインド化されている	アウトカム評価者もブラインド化されている	データの欠損がない	評価するための十分な情報がない	評価するための十分な情報がない	low4項目、high0項目
5	Rossignon 1990	記載がなく、評価できなかった	記載がなく、評価できなかった	同じ溶媒（エタノール）を用いて、二重盲検で行った	盲検で行われた	100%フォローされている	事前に計画されたプロトコールが閲覧できなかった	事前に計画されたプロトコールが閲覧できなかった	Lowが多く、Lowとした
6	Slotman 1992	適切にランダム化が行われた	研究者たちは割り付けを知らされていなかった。	多施設共同ランダム化プラセボ対照二重盲検試験	多施設共同ランダム化プラセボ対照二重盲検試験	ほぼ100%フォローされた	100%報告された	研究の中断なし	全項目Low risk
7	Vincent 2001	記載がなく、評価できなかった	記載がなく、評価できなかった	二重盲検で行われた	盲検で行われた	死亡以外の脱落は4症例のみ	事前に計画されたプロトコールが閲覧できなかった	予定された中間解析の結果で中止された	Lowが多く、Lowとした

CQ13_05
Risk of bias table, Severe adverse effects

Outcome		Severe adverse effects		risk of bias		not serious (0)			
番号	著者名 発表年（Forest plot表示）	risk of bias評価							
		ランダム割付順番の生成 random sequence generation	割り付けの隠蔽化 allocation concealment	ブラインド blinding		不完全なアウトカムデータ incomplete outcome data	選択されたアウトカムの報告 selective outcome reporting	その他のバイアス Other sources of bias	研究内でのバイアスのリスク Risk of bias within a study
				研究参加者と治療提供者 participants and personnel	アウトカム評価者 outcome assessors				
1	Abraham 1996	Unclear risk	Unclear risk	Low risk	Low risk	Low risk	Unclear risk	Unclear risk	Unclear risk
2	Abraham 1999	Unclear risk	Unclear risk	Low risk	Low risk	Low risk	Unclear risk	Unclear risk	Unclear risk
3	Bone 1989	Low risk	Low risk	Low risk	Low risk	Low risk	Unclear risk	Unclear risk	Low risk
5	Rossignon 1990	Unclear risk	Unclear risk	Low risk	Low risk	Low risk	Unclear risk	Low risk	Low risk
番号	著者名 発表年（Forest plot表示）	risk of biasコメント							
1	Abraham 1996	ランダム化の詳細は未記載	割り付けの詳細は不明だが、薬剤師が薬剤のシリンジおよびチューブを覆い隠蔽した	研究参加者、治療提供者ともブラインド化されている	アウトカム評価者もブラインド化されている	データの欠損がない	評価するための十分な情報がない	評価するための十分な情報がない	low3項目、high0項目
2	Abraham 1999	ランダム化の詳細は未記載	割り付けの詳細は不明だが、薬剤師が薬剤のシリンジおよびチューブを覆い隠蔽した	研究参加者、治療提供者ともブラインド化されている	アウトカム評価者もブラインド化されている	データの欠損がない	評価するための十分な情報がない	評価するための十分な情報がない	low3項目、high0項目
3	Bone 1989	中央割り付けにてランダム化	中央割り付けにてランダム化	研究参加者、治療提供者ともブラインド化されている	アウトカム評価者もブラインド化されている	データの欠損がない	評価するための十分な情報がない	評価するための十分な情報がない	low5項目、high0項目
5	Rossignon 1990	記載がなく、評価できなかった	記載がなく、評価できなかった	同じ溶媒（エタノール）を用いて、二重盲検で行った	盲検で行われた	100%フォローされている	事前に計画されたプロトコールが閲覧できなかった	事前に計画されたプロトコールが閲覧できなかった	Lowが多く、Lowとした

CQ13_06
Risk of bias table, mortality

Outcome		Short term mortality		risk of bias判定		not serious (0)			
番号	著者名 発表年（Forest plot表示）	risk of bias評価							
		ランダム割付順番の生成 random sequence generation	割り付けの隠蔽化 allocation concealment	ブラインド blinding		不完全なアウトカムデータ incomplete outcome data	選択されたアウトカムの報告 selective outcome reporting	その他のバイアス Other sources of bias	研究内でのバイアスのリスク Risk of bias within a study
				研究参加者と治療提供者 participants and personnel	アウトカム評価者 outcome assessors				
2	McAuley 2014	Low risk	Low risk	Low risk	Low risk	Low risk	Low risk	Low risk	Low risk
3	Truwit 2014	Low risk	Unclear risk	Low risk	Low risk	Low risk	Low risk	Low risk	Low risk
番号	著者名 発表年（Forest plot表示）	risk of biasコメント							
2	McAuley 2014	24-hour telephone randomisation serviceを使用し、適切にランダム化された	24-hour telephone randomisation serviceを使用し、適切に隠蔽化された。	割り付けを参加者と治療提供者は知らされていなかった。	割り付けを評価者は知らされていなかった。	ほぼ100%フォローされた	100%報告された	他のbiasなし	全ての項目がLow risk
3	Truwit 2014	Interactive Voice Response System (I.V.R.S.) または web-based システムを使用し行われた	block法を適用	参加者と治療提供者に割り付けは知られていなかった。	評価者に割り付けは知られていなかった。	ほぼ100%フォローされた	100%報告された	他のbiasなし	ほぼ全項目Low risk

CQ13_06
Risk of bias table, Severe adverse effects

Outcome		Severe adverse effects		risk of bias		not serious (0)			
番号	著者名 発表年<br>（Forest plot表示）	risk of bias評価							
		ランダム割付順番の生成<br>random sequence generation	割り付けの隠蔽化<br>allocation concealment	ブラインド<br>blinding<br>研究参加者と治療提供者<br>participants and personnel	アウトカム評価者<br>outcome assessors	不完全なアウトカムデータ<br>incomplete outcome data	選択されたアウトカムの報告<br>selective outcome reporting	その他のバイアス<br>Other sources of bias	研究内でのバイアスのリスク<br>Risk of bias within a study
1	Craig 2011	Low risk	Low risk	Low risk	Low risk	Low risk	Low risk	Low risk	Low risk
2	McAuley 2014	Low risk	Low risk	Low risk	Low risk	Low risk	Low risk	Low risk	Low risk
番号	著者名 発表年<br>（Forest plot表示）	risk of biasコメント							
1	Craig 2011	ブロック無作為化で行った	独立した薬剤師がランダム割付を行い、研究者にブロック数を教えずに無作為化を行った	どちらもカプセル化して、盲検化した	どちらもカプセル化して、盲検化した	100%フォローされた	予定したアウトカムが報告されている	この研究には他のバイアスはない	すべてLowであり、Lowrとした
2	McAuley 2014	24-hour telephone randomisation serviceを使用し、適切にランダム化された	24-hour telephone randomisation serviceを使用し、適切に隠蔽化された。	割り付けを参加者と治療提供者は知らされていなかった。	割り付けを評価者は知らされていなかった。	ほぼ100%フォローされた	100%報告された	他のbiasなし	全ての項目がLow risk

CQ13_07
Risk of bias table, mortality

Outcome		Short term mortality		risk of bias		serious (−1)			
番号	著者名 発表年（Forest plot表示）	risk of bias評価							
		ランダム割付順番の生成 random sequence generation	割り付けの隠蔽化 allocation concealment	ブラインド blinding		不完全なアウトカムデータ incomplete outcome data	選択されたアウトカムの報告 selective outcome reporting	その他のバイアス Other sources of bias	研究内でのバイアスのリスク Risk of bias within a study
				研究参加者と治療提供者 participants and personnel	アウトカム評価者 outcome assessors				
1	Anzueto 1996	Low risk	Low risk	Low risk	Low risk	Low risk	Unclear risk	Low risk	Low risk
2	Gregory 1997	Unclear risk	Unclear risk	High risk	High risk	Low risk	Unclear risk	Low risk	Unclear risk
3	Kesecioglu 2009	Low risk	Low risk	High risk	High risk	Low risk	Unclear risk	Low risk	Low risk
4	Markart 2007	Unclear risk	Unclear risk	High risk	High risk	Low risk	Low risk	Low risk	Unclear risk
5	Spragg 2003	Unclear risk	Unclear risk	High risk	High risk	Low risk	Unclear risk	Unclear risk	High risk
6	Spragg 2004	Unclear risk	Unclear risk	Low risk	Low risk	Low risk	Low risk	Low risk	Low risk
7	Spragg 2011	Low risk	Low risk	Low risk	Low risk	Low risk	Low risk	Low risk	Low risk
9	Weg 1994	Unclear risk	Unclear risk	Low risk	Unclear risk	Low risk	Unclear risk	Unclear risk	Unclear risk
10	Willson 2015	Low risk	Unclear risk	Low risk	Low risk	Low risk	Low risk	High risk	Low risk

番号	著者名 発表年（Forest plot表示）	risk of biasコメント							
1	Anzueto 1996	中央割付でコンピューターによるランダム化を行った	中央割付でコンピューターによるランダム化を行った	不透明な容器にいれ盲検化された	不透明な容器にいれ盲検化された	100%フォローされた	事前に計画されたプロトコールが閲覧できなかった	この研究には他のバイアスはない	Lowが多く、Lowとした
2	Gregory 1997	記載がなく、評価できなかった	記載がなく、評価できなかった	非盲検で行われた	非盲検で行われた	100%フォローされた	事前に計画されたプロトコールが閲覧できなかった	この研究には他のバイアスはない	Unclearが多く、LowとHighがそれぞれ2項目ずつあり、Unclearとした
3	Kesecioglu 2009	電話にて中央割付で行われた	電話にて中央割付で行われた	安全な擬似薬はなく、非盲検で行われた	安全な擬似薬はなく、非盲検で行われた	フォローアップできなかったのは1例のみ	事前に計画されたプロトコールが閲覧できなかった	予定された中間解析で死亡率上昇の傾向があり、中止された	全体的にLowが多く、Lowとした
4	Markart 2007	記載がなく、評価できなかった	記載がなく、評価できなかった	対照群がプラセボではなく、非使用群	対照群がプラセボではなく、非使用群	100%フォローされた	事前に設定したoutcomeが報告されている	この研究には他のバイアスはない	Lowが多いが、UnclearとHighが2項目ずつあり、Unclearとした
5	Spragg 2003	ランダム化の方法は未記載	隠蔽化は未記載	盲検化されていない	盲検化されていない	１００%フォローされた	判断には情報が不十分	判断には情報が不十分	Low riskが1項目のみ
6	Spragg 2004	記載がなく、評価できなかった	記載がなく、評価できなかった	容器や投与するカテーテルも不透明にし盲検化した	容器や投与するカテーテルも不透明にし盲検化した	100%フォローされた	予定されていたアウトカムは報告されていた	この研究には他のバイアスはない	Lowがほとんどであり、Lowとした
7	Spragg 2011	コンピューターが生成した乱数を使用し、適切にランダム化された。	コンピューターが生成した乱数を使用し、適切に隠蔽化された。	割り付けを参加者と治療提供者は知らされていなかった。	割り付けを評価者は知らされていなかった。	ほぼ100%フォローされた	100%報告された	その他のbiasなし	全ての項目がLow risk
9	Weg 1994	ランダム化の方法の情報が不十分	割り付けの方法が未記載	盲検化されていた。	記載なし	100%フォローされた。	評価するには不十分な情報	評価するには不十分な情報	Unclearが多い
10	Willson 2015	乱数表を用いて割り付けられた	allocationはblock法	割り付けを参加者と治療提供者は知らされていなかった。	割り付けを評価者は知らされていなかった。	ほぼ100%フォローされた	100%報告された	研究がスポンサーの意向で中止	全項目ほぼLow risk

CQ13_07
Risk of bias table, Severe adverse effects

Outcome		Severe adverse effects		risk of bias		not serious (0)			
番号	著者名 発表年（Forest plot表示）	risk of bias評価							
		ランダム割付順番の生成 random sequence generation	割り付けの隠蔽化 allocation concealment	ブラインド blinding		不完全なアウトカムデータ incomplete outcome data	選択されたアウトカムの報告 selective outcome reporting	その他のバイアス Other sources of bias	研究内でのバイアスのリスク Risk of bias within a study
				研究参加者と治療提供者 participants and personnel	アウトカム評価者 outcome assessors				
1	Anzueto 1996	Low risk	Low risk	Low risk	Low risk	Low risk	Unclear risk	Low risk	Low risk
2	Gregory 1997	Unclear risk	Unclear risk	High risk	High risk	Low risk	Unclear risk	Low risk	Unclear risk
3	Kesecioglu 2009	Low risk	Low risk	High risk	High risk	Low risk	Unclear risk	Low risk	Low risk
6	Spragg 2004	Unclear risk	Unclear risk	Low risk	Low risk	Low risk	Low risk	Low risk	Low risk
7	Spragg 2011	Low risk	Low risk	Low risk	Low risk	Low risk	Low risk	Low risk	Low risk
10	Willson 2015	Low risk	Unclear risk	Low risk	Low risk	Low risk	Low risk	High risk	Low risk
番号	著者名 発表年（Forest plot表示）	risk of biasコメント							
1	Anzueto 1996	中央割付でコンピューターによるランダム化を行った	中央割付でコンピューターによるランダム化を行った	不透明な容器にいれ盲検化された	不透明な容器にいれ盲検化された	100%フォローされた	事前に計画されたプロトコールが閲覧できなかった	この研究には他のバイアスはない	Lowが多く、Lowとした
2	Gregory 1997	記載がなく、評価できなかった	記載がなく、評価できなかった	非盲検で行われた	非盲検で行われた	100%フォローされた	事前に計画されたプロトコールが閲覧できなかった	この研究には他のバイアスはない	Unclearが多く、LowとHighがそれぞれ2項目ずつあり、Unclearとした
3	Kesecioglu 2009	電話にて中央割付で行われた	電話にて中央割付で行われた	安全な擬似薬はなく、非盲検で行われた	安全な擬似薬はなく、非盲検で行われた	フォローアップできなかったのは1例のみ	事前に計画されたプロトコールが閲覧できなかった	予定された中間解析で死亡率上昇の傾向があり、中止された	全体的にLowが多く、Lowとした
6	Spragg 2004	記載がなく、評価できなかった	記載がなく、評価できなかった	容器や投与するカテーテルも不透明にし盲検化した	容器や投与するカテーテルも不透明にし盲検化した	100%フォローされた	予定されていたアウトカムは報告されていた	この研究には他のバイアスはない	Lowがほとんどであり、Lowとした
7	Spragg 2011	コンピューターが生成した乱数を使用し、適切にランダム化された。	コンピューターが生成した乱数を使用し、適切に隠蔽化された。	割り付けを参加者と治療提供者は知らされていなかった。	割り付けを評価者は知らされていなかった。	ほぼ100%フォローされた	100%報告された	その他のbiasなし	全ての項目がLow risk
10	Willson 2015	乱数表を用いて割り付けられた	allocationはblock法	割り付けを参加者と治療提供者は知らされていなかった。	割り付けを評価者は知らされていなかった。	ほぼ100%フォローされた	100%報告された	研究がスポンサーの意向で中止	全項目ほぼLow risk

CQ13_08
Risk of bias table, mortality

Outcome		Short term mortality		risk of bias		not serious (0)			
番号	著者名 発表年（Forest plot表示）	risk of bias評価							
		ランダム割付順番の生成 random sequence generation	割り付けの隠蔽化 allocation concealment	ブラインド blinding		不完全なアウトカムデータ incomplete outcome data	選択されたアウトカムの報告 selective outcome reporting	その他のバイアス Other sources of bias	研究内でのバイアスのリスク Risk of bias within a study
				研究参加者と治療提供者 participants and personnel	アウトカム評価者 outcome assessors				
1	Cornet 2014	Low risk	High risk	High risk	Low risk	Low risk	Low risk	Low risk	Low risk
2	Liu 2008	Unclear risk	Low risk	Low risk	Low risk	Low risk	Low risk	Low risk	Low risk
番号	著者名 発表年（Forest plot表示）	risk of biasコメント							
1	Cornet 2014	ランダム化はCONSORTガイドラインに準じ、適切にランダム化された。	オープンラベルが適用されている	オープンラベルが適用されている	オープンラベルが評価に影響しにくい	100%フォローされた	100%報告された	研究の中断なし	Low risk 5 High risk 2
2	Liu 2008	ランダム化の方法が未記載	隠蔽された置換ブロック法	ブラインド化の記載あり	ブラインド化の記載あり	データが欠損していない	100%報告されている	研究の中断なし	low5項目

CQ13_08
Risk of bias table, Severe adverse effects

Outcome		Severe adverse effects		risk of bias		serious (−1)			
番号	著者名 発表年（Forest plot表示）	risk of bias評価							
		ランダム割付順番の生成 random sequence generation	割り付けの隠蔽化 allocation concealment	ブラインド blinding		不完全なアウトカムデータ incomplete outcome data	選択されたアウトカムの報告 selective outcome reporting	その他のバイアス Other sources of bias	研究内でのバイアスのリスク Risk of bias within a study
				研究参加者と治療提供者 participants and personnel	アウトカム評価者 outcome assessors				
1	Cornet 2014	Low risk	High risk	High risk	High risk	Low risk	Low risk	Low risk	High risk
2	Liu 2008	Unclear risk	Low risk	Low risk	Low risk	Low risk	Low risk	Low risk	Low risk
番号	著者名 発表年（Forest plot表示）	risk of biasコメント							
1	Cornet 2014	ランダム化はCONSORTガイドラインに準じ、適切にランダム化された。	オープンラベルが適用されている	オープンラベルが適用されている	オープンラベルが評価に影響している可能性がある。	100%フォローされた	100%報告された	研究の中断なし	Low risk 4 High risk 3
2	Liu 2008	ランダム化の方法が未記載	隠蔽された置換ブロック法	ブラインド化の記載あり	ブラインド化の記載あり	データが欠損していない	100%報告されている	研究の中断なし	low5項目

CQ13_09

Risk of bias table, mortality

Outcome		Short term mortality		risk of bias		not serious (0)			
番号	著者名 発表年<br>（Forest plot表示）	risk of bias評価							
		ランダム割付順番の生成<br>random sequence generation	割り付けの隠蔽化<br>allocation concealment	ブラインド blinding<br>研究参加者と治療提供者<br>participants and personnel	ブラインド blinding<br>アウトカム評価者<br>outcome assessors	不完全なアウトカムデータ<br>incomplete outcome data	選択されたアウトカムの報告<br>selective outcome reporting	その他のバイアス<br>Other sources of bias	研究内でのバイアスのリスク<br>Risk of bias within a study
1	Bernard 1997	Low risk	Low risk	Low risk	Low risk	Low risk	Unclear risk	Low risk	Low risk
2	Jepsen 1992	Unclear risk	Unclear risk	Low risk	Low risk	Low risk	Unclear risk	Low risk	Low risk
3	Ortolani 2000	Unclear risk	Unclear risk	High risk	High risk	Low risk	Unclear risk	Unclear risk	Unclear risk
4	Suter 1994	Unclear risk	Unclear risk	Low risk	Low risk	Low risk	Unclear risk	Low risk	Low risk

番号	著者名 発表年<br>（Forest plot表示）	risk of biasコメント							
1	Bernard 1997	コンピューターによるブロック無作為化	コンピューターによるブロック無作為化	同じ量に希釈され、盲検化された	同じ量に希釈され、盲検化された	96%フォローされた	事前に計画されたプロトコールが閲覧できなかった	この研究には他のバイアスがない	Lowが多く、Lowとした
2	Jepsen 1992	記載なし	記載なし	二重盲検で行われた	盲検で行われた	100%フォローされた	事前に計画されたプロトコールが閲覧できなかった	この研究には、他のバイアスがない	Low 4項目>Unclear3項目で、Lowとした
3	Ortolani 2000	ランダム化に関する詳細不明	割り付けに関する詳細不明	研究参加者、治療提供者ともブラインド化されていない	アウトカム評価者ブラインド化されていない	データの欠損がない	評価するための十分な情報がない	評価するための十分な情報がない	low1項目、high2項目
4	Suter 1994	ランダム化に関する詳細不明	割り付けに関する詳細不明	研究参加者、治療提供者ともブラインド化されている	アウトカム評価者もブラインド化されている	データの欠損がない	評価するための十分な情報がない	他のバイアスなし	low4項目、high0項目

CQ13_10
Risk of bias table, mortality

Outcome		Short term mortality		risk of bias		not serious (0)			
番号	著者名 発表年（Forest plot表示）	risk of bias評価							
		ランダム割付順番の生成 random sequence generation	割り付けの隠蔽化 allocation concealment	ブラインド blinding		不完全なアウトカムデータ incomplete outcome data	選択されたアウトカムの報告 selective outcome reporting	その他のバイアス Other sources of bias	研究内でのバイアスのリスク Risk of bias within a study
				研究参加者と治療提供者 participants and personnel	アウトカム評価者 outcome assessors				
1	ARDSnet 2000	Low risk	Low risk	Low risk	Low risk	Unclear risk	Unclear risk	Unclear risk	Low risk
番号	著者名 発表年（Forest plot表示）	risk of biasコメント							
1	ARDSnet 2000	中央割り付けで、コンピュータを用いてランダム化された	中央割り付けで、コンピュータを用いてランダム化された	研究参加者、治療提供者（医師、看護師、呼吸療法士）ともブラインド化されている	アウトカム評価者もブラインド化されている	院内死亡のみ記載（K-M曲線を見ると90日以降死亡率はほとんど変化なし）	評価するための十分な情報がない	有効性を証明できず試験中断	low4項目、high0項目

CQ13_10
Risk of bias table, Severe adverse effects

Outcome		Severe adverse effects		risk of bias		not serious (0)			
番号	著者名 発表年（Forest plot表示）	risk of bias評価							
		ランダム割付順番の生成 random sequence generation	割り付けの隠蔽化 allocation concealment	ブラインド blinding 研究参加者と治療提供者 participants and personnel	ブラインド blinding アウトカム評価者 outcome assessors	不完全なアウトカムデータ incomplete outcome data	選択されたアウトカムの報告 selective outcome reporting	その他のバイアス Other sources of bias	研究内でのバイアスのリスク Risk of bias within a study
1	ARDSnet 2000	Low risk	Low risk	Low risk	Low risk	Low risk	Unclear risk	Unclear risk	Low risk
番号	著者名 発表年（Forest plot表示）	risk of biasコメント							
1	ARDSnet 2000	中央割り付けで、コンピュータを用いてランダム化された	中央割り付けで、コンピュータを用いてランダム化された	研究参加者、治療提供者（医師、看護師、呼吸療法士）ともブラインド化されている	アウトカム評価者もブラインド化されている	データの欠損がない	評価するための十分な情報がない	有効性を証明できず試験中断	low5項目、high0項目

CQ13_11
Risk of bias table, mortality

Outcome		Short term mortality		risk of bias		not serious (0)			
番号	著者名 発表年（Forest plot表示）	risk of bias評価							
		ランダム割付順番の生成 random sequence generation	割り付けの隠蔽化 allocation concealment	ブラインド blinding		不完全なアウトカムデータ incomplete outcome data	選択されたアウトカムの報告 selective outcome reporting	その他のバイアス Other sources of bias	研究内でのバイアスのリスク Risk of bias within a study
				研究参加者と治療提供者 participants and personnel	アウトカム評価者 outcome assessors				
1	Wiedemann 2002	Low risk	Low risk	Low risk	Low risk	Low risk	Unclear risk	Unclear risk	Low risk
番号	著者名 発表年（Forest plot表示）	risk of biasコメント							
1	Wiedemann 2002	コンピュータが生成した割り付けにより行われた	多施設共同ランダム化プラセボ対照二重盲検試験	多施設共同ランダム化プラセボ対照二重盲検試験	多施設共同ランダム化プラセボ対照二重盲検試験	100%報告された	評価には情報が不十分	評価には情報が不十分	Low risk 5 Unclear risk 2

CQ13_01　Risk of bias summary, Risk of bias graph

Short term mortality

	Random sequence generation (selection bias)	Allocation concealment (selection bias)	Blinding of participants and personnel (performance bias)	Blinding of outcome assessment (detection bias)	Incomplete outcome data (attrition bias)	Selective reporting (reporting bias)	Other bias
Dellinger 1998	?	?	+	+	+	?	−
Gerlach 2003	+	+	−	−	+	?	+
Mehta 2001	+	+	−	−	+	?	?
Michael 1998	?	?	−	+	?	+	+
Park 2003	−	−	−	−	+	?	+
Taylor 2004	+	+	+	+	+	?	−
Troncy 1998	?	?	−	−	+	+	?

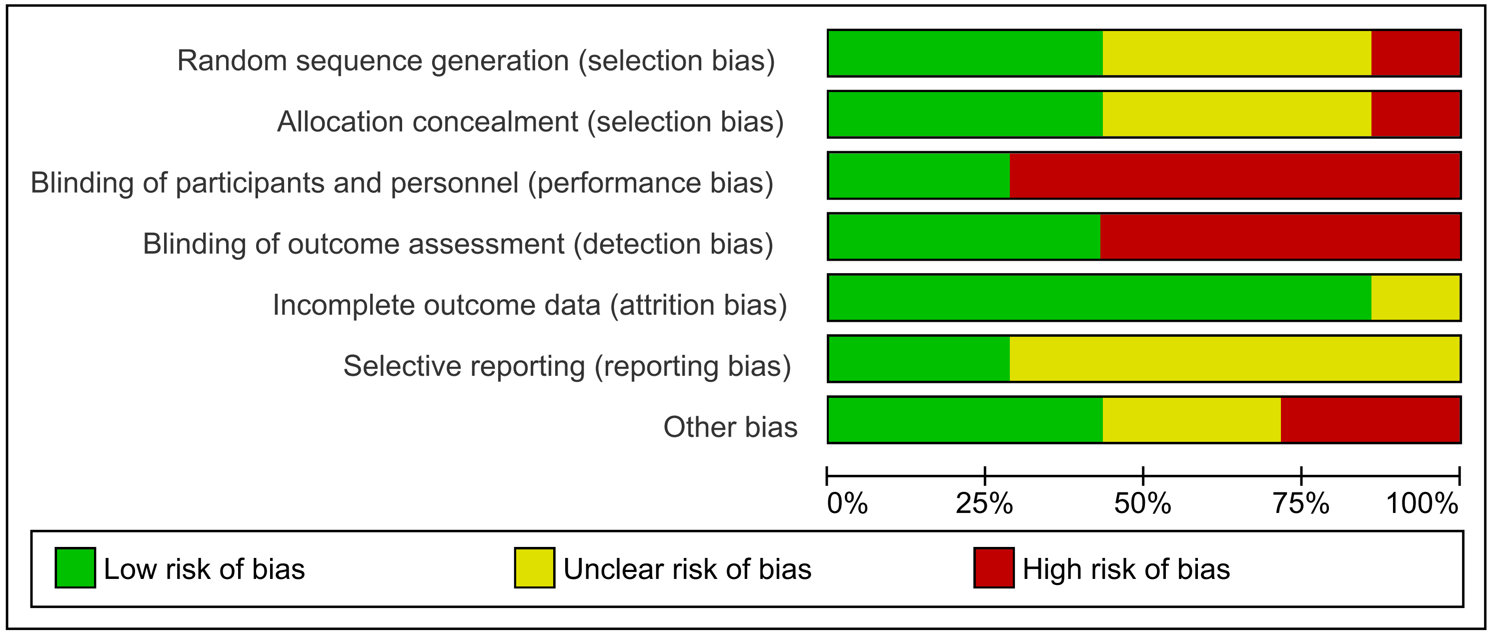

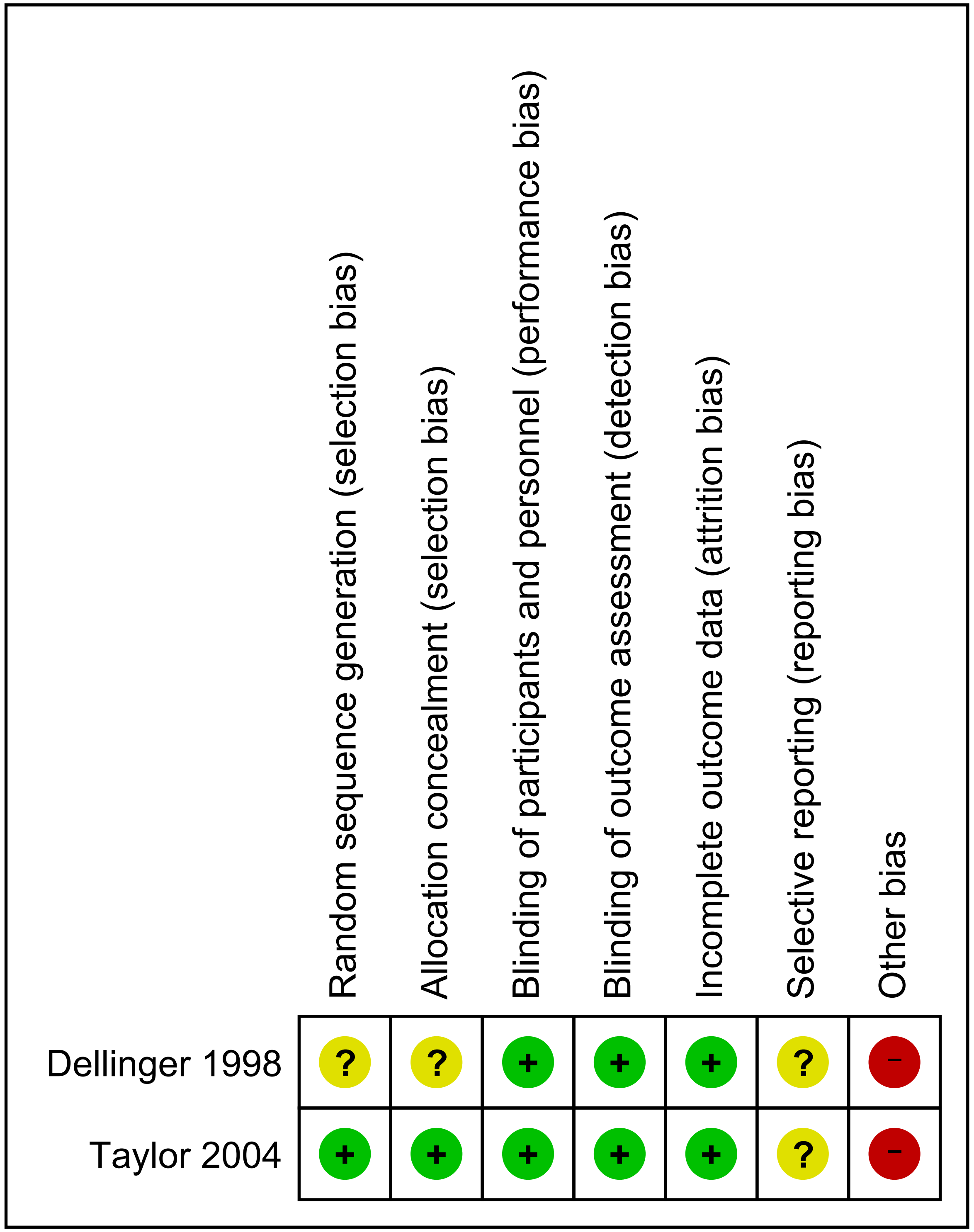

Severe complication

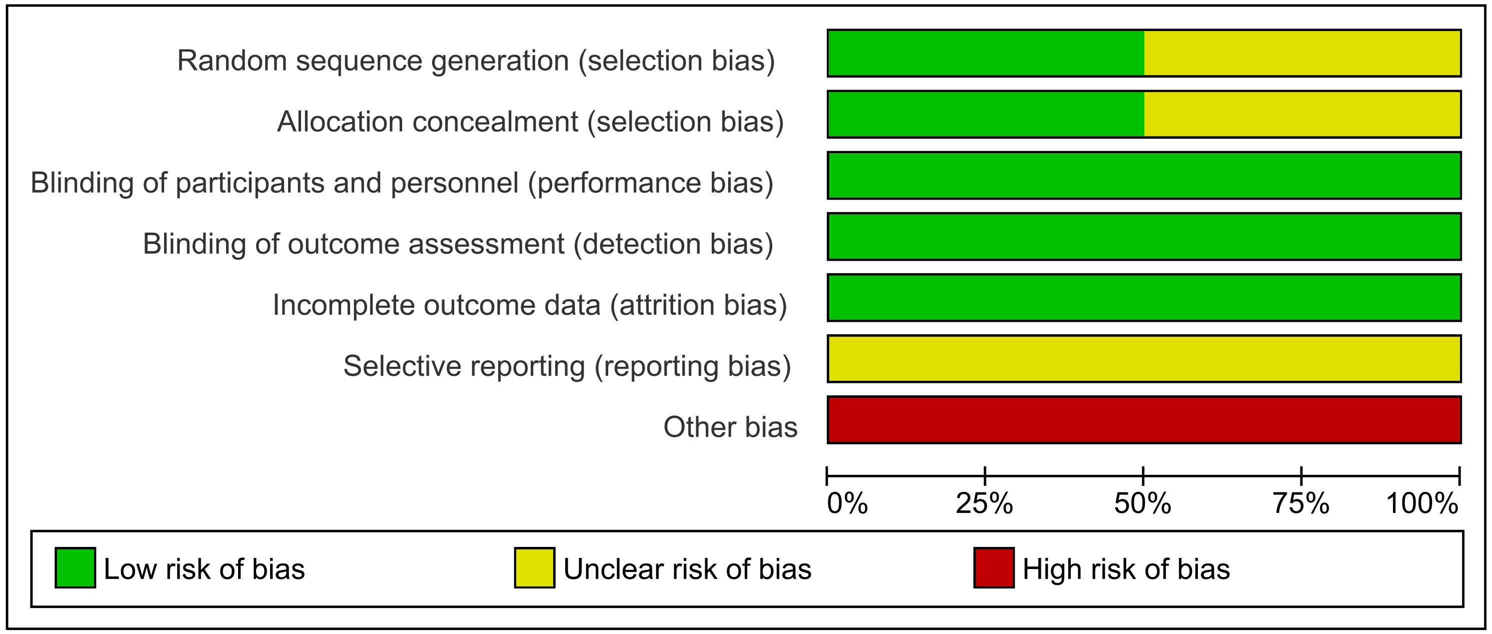

Short term mortality

Study or Subgroup	iNO Events	iNO Total	Placebo Events	Placebo Total	Weight	Risk Ratio M-H, Random, 95% CI
Dellinger 1998	24	120	6	57	9.1%	1.90 [0.82, 4.39]
Gerlach 2003	3	20	4	20	3.4%	0.75 [0.19, 2.93]
Mehta 2001	4	8	3	6	5.7%	1.00 [0.35, 2.88]
Michael 1998	11	18	9	18	18.3%	1.22 [0.68, 2.21]
Park 2003	4	11	2	6	3.4%	1.09 [0.28, 4.32]
Taylor 2004	44	192	39	193	43.8%	1.13 [0.77, 1.66]
Troncy 1998	9	15	8	15	16.2%	1.13 [0.60, 2.11]
**Total (95% CI)**		**384**		**315**	**100.0%**	**1.18 [0.91, 1.52]**
Total events	99		71			

Heterogeneity: Tau² = 0.00; Chi² = 1.87, df = 6 (P = 0.93); I² = 0%

Test for overall effect: Z = 1.26 (P = 0.21)

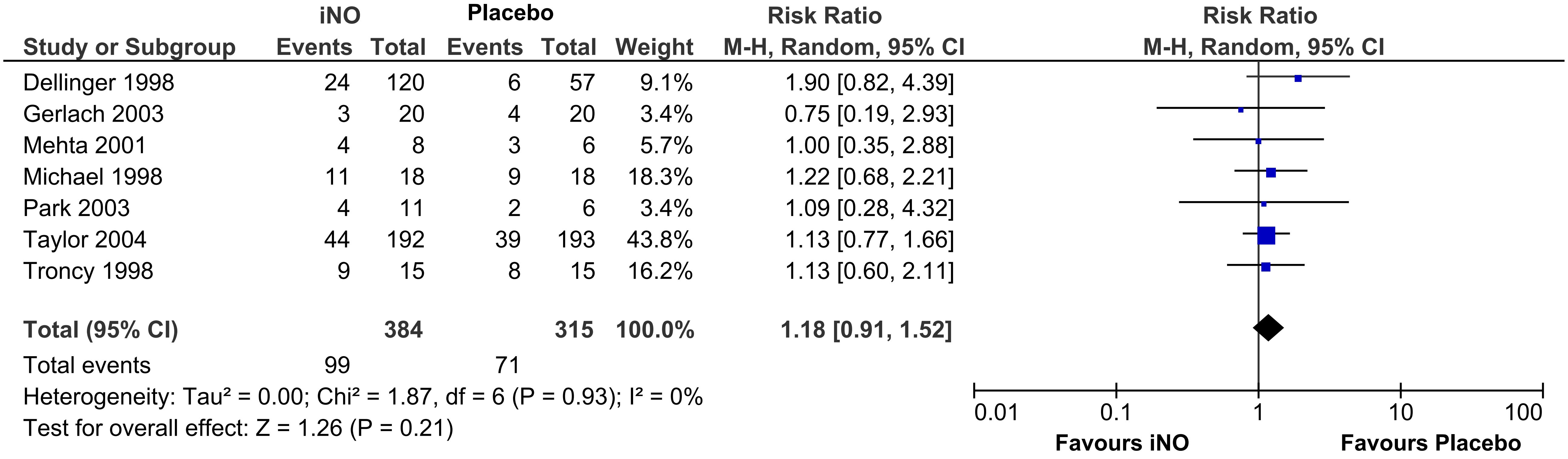

Severe complication

Study or Subgroup	iNO Events	iNO Total	Placebo Events	Placebo Total	Weight	Risk Ratio M-H, Random, 95% CI
Dellinger 1998	7	120	1	57	18.7%	3.33 [0.42, 26.39]
Taylor 2004	10	192	6	193	81.3%	1.68 [0.62, 4.52]
**Total (95% CI)**		**312**		**250**	**100.0%**	**1.90 [0.78, 4.66]**
Total events	17		7			

Heterogeneity: Tau² = 0.00; Chi² = 0.35, df = 1 (P = 0.55); I² = 0%

Test for overall effect: Z = 1.41 (P = 0.16)

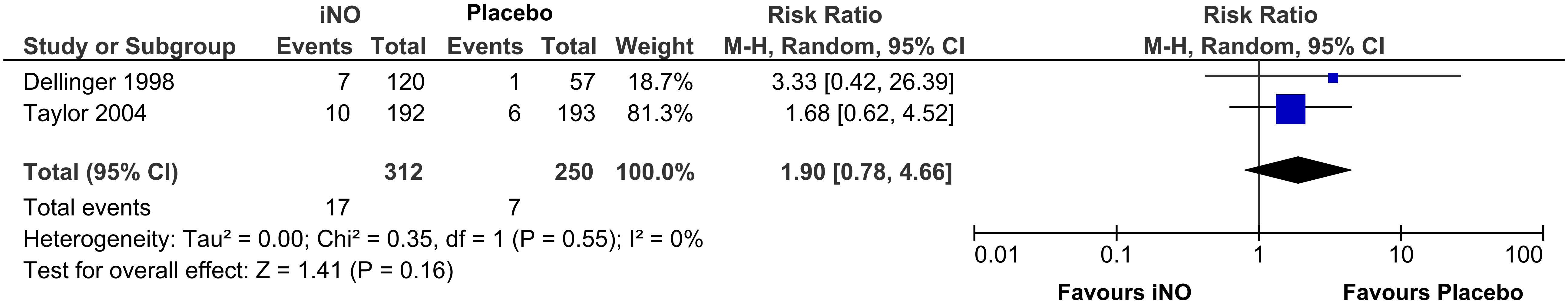

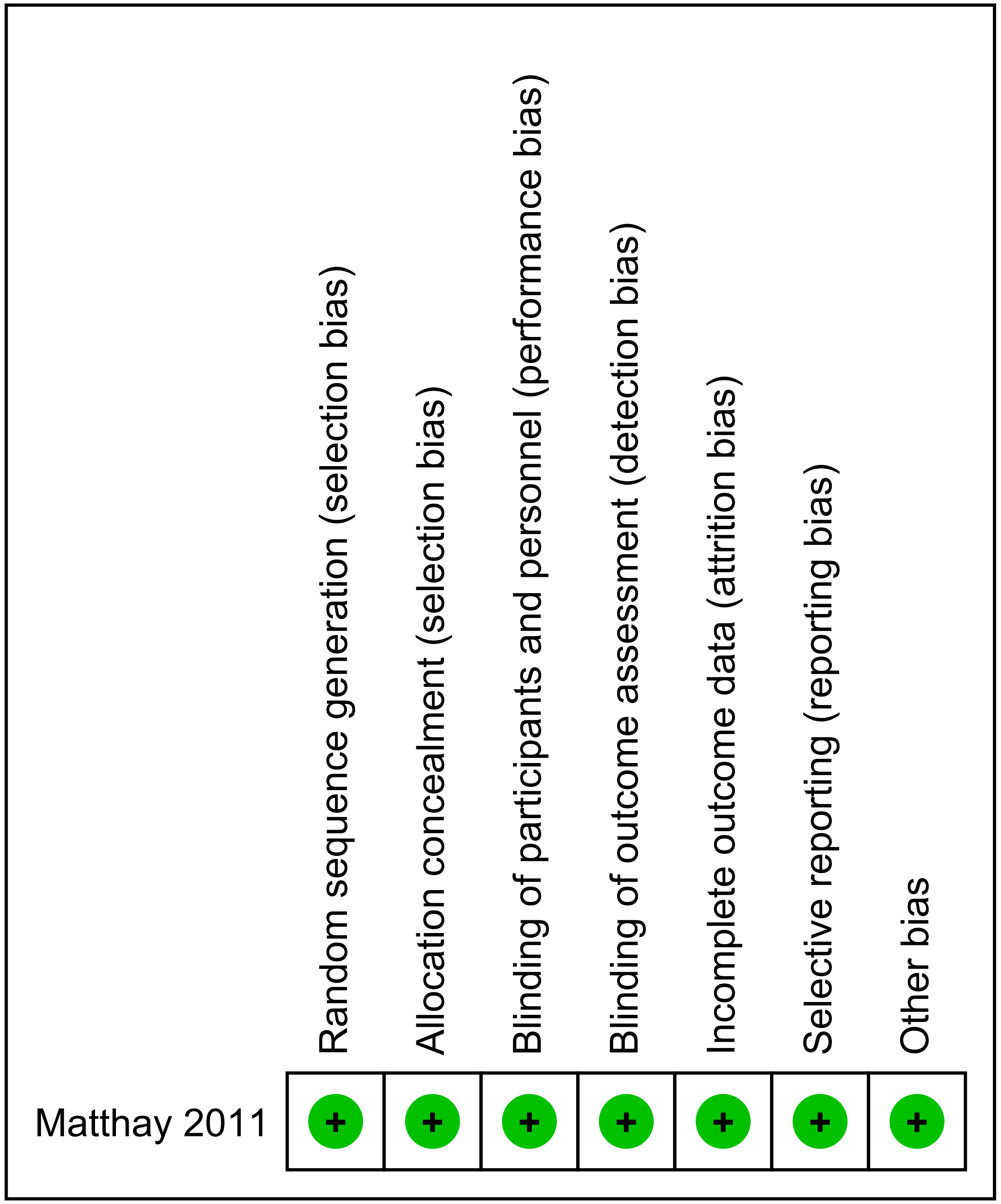

Short term mortality

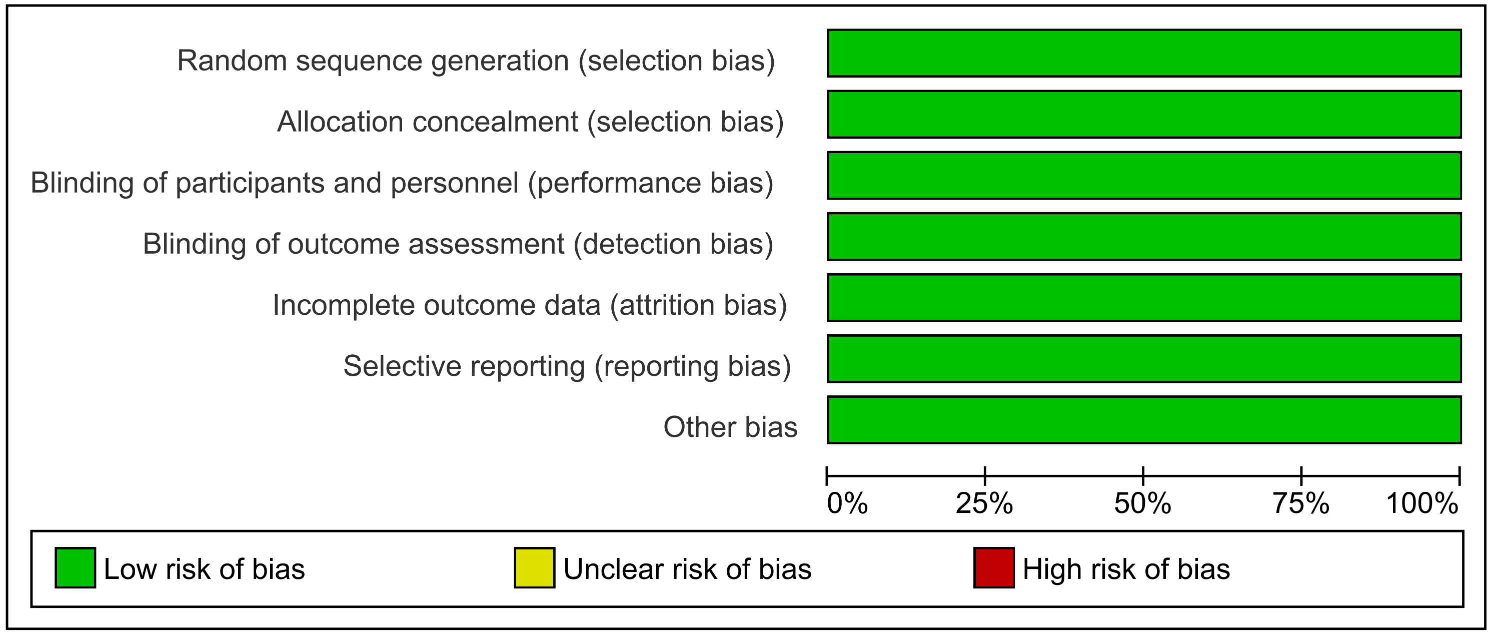

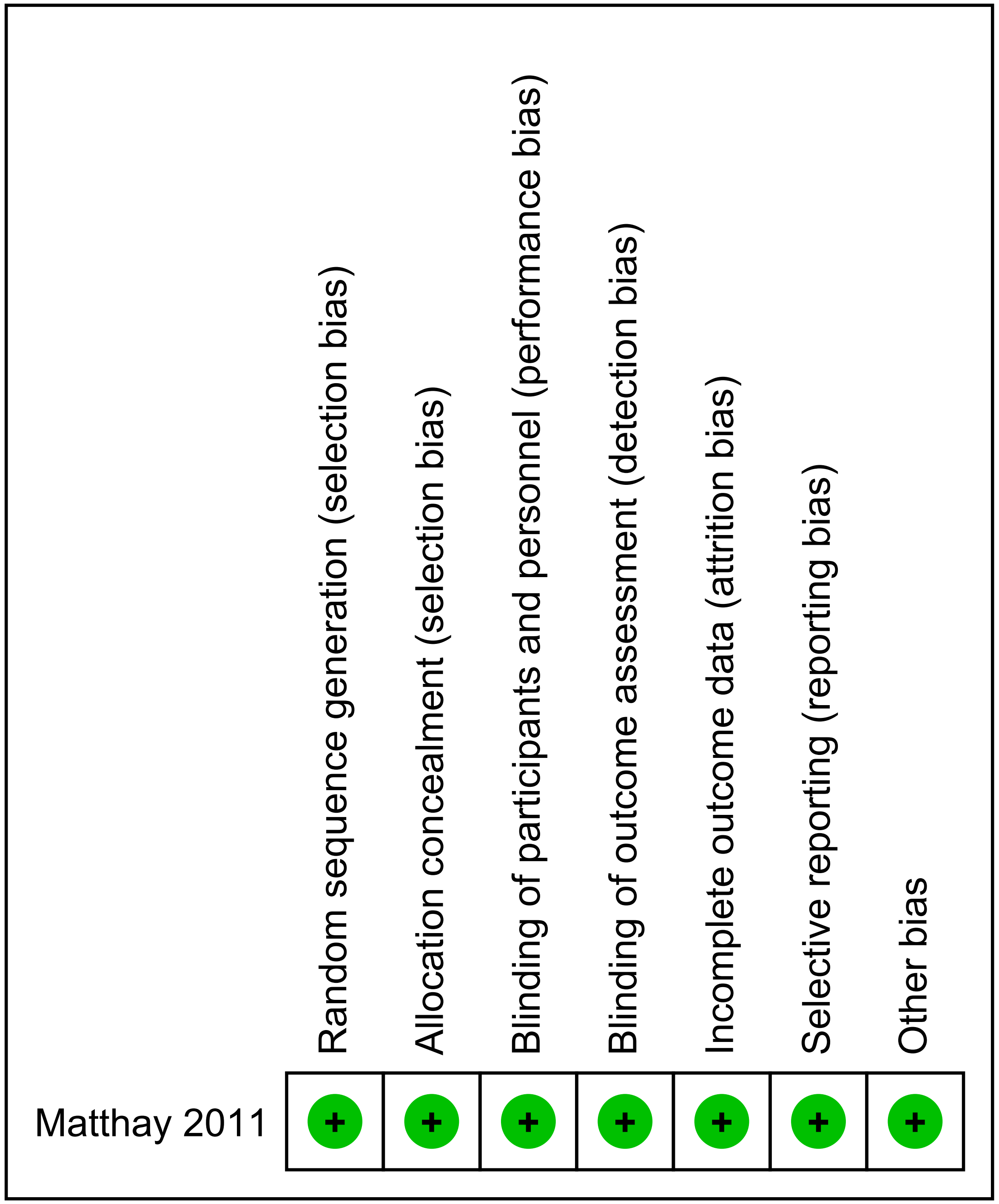

Severe complication

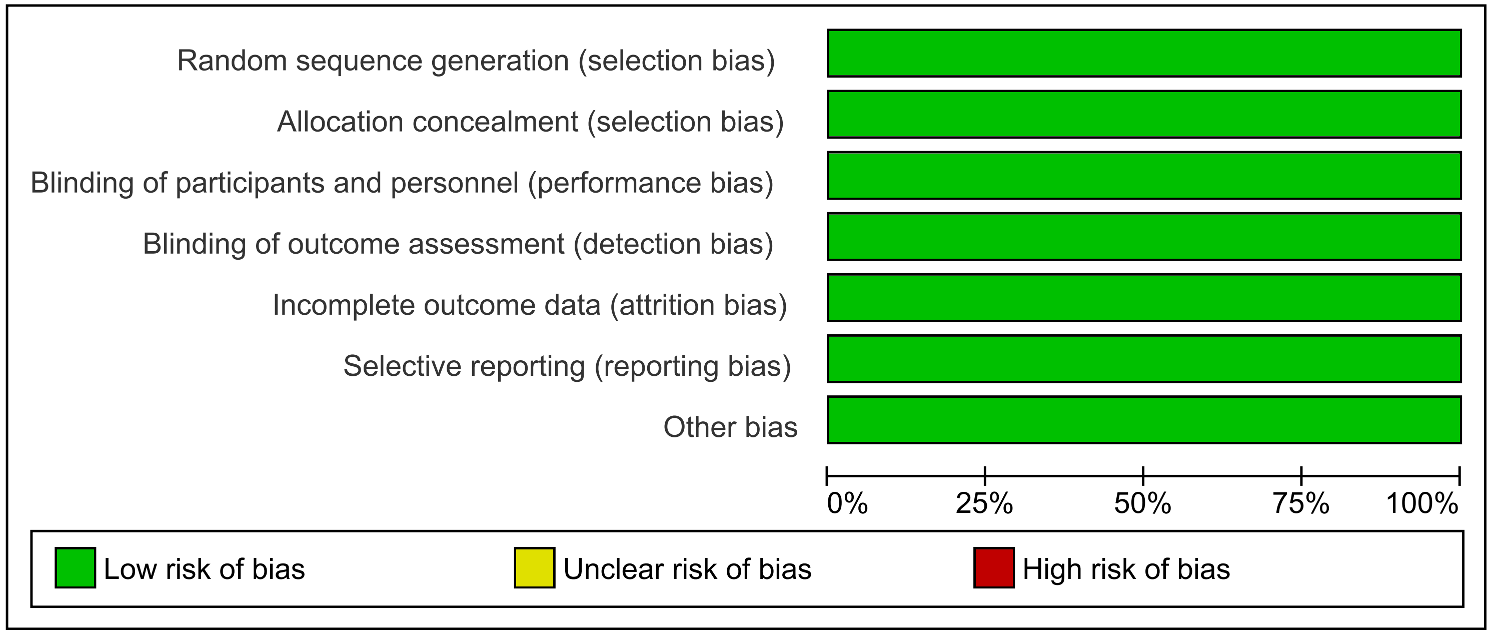

Short term mortality

Study or Subgroup	β2-agonist Events	β2-agonist Total	Placebo Events	Placebo Total	Weight	Risk Ratio M-H, Random, 95% CI
Matthay 2011	37	152	24	130	100.0%	1.32 [0.83, 2.08]
**Total (95% CI)**		**152**		**130**	**100.0%**	**1.32 [0.83, 2.08]**
Total events	37		24			

Heterogeneity: Not applicable
Test for overall effect: Z = 1.19 (P = 0.24)

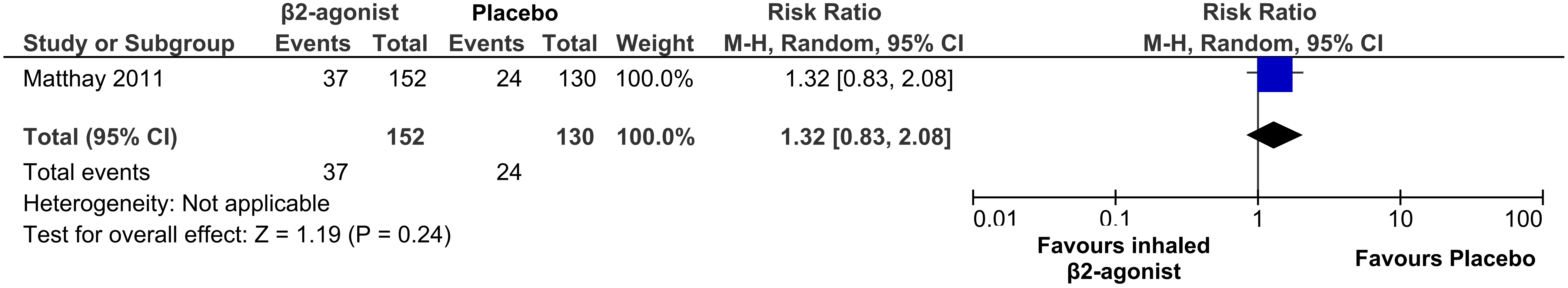

Severe complication

Study or Subgroup	β2-agonist Events	β2-agonist Total	Placebo Events	Placebo Total	Weight	Risk Ratio M-H, Random, 95% CI
Matthay 2011	12	152	4	130	100.0%	2.57 [0.85, 7.76]
**Total (95% CI)**		**152**		**130**	**100.0%**	**2.57 [0.85, 7.76]**
Total events	12		4			

Heterogeneity: Not applicable
Test for overall effect: Z = 1.67 (P = 0.10)

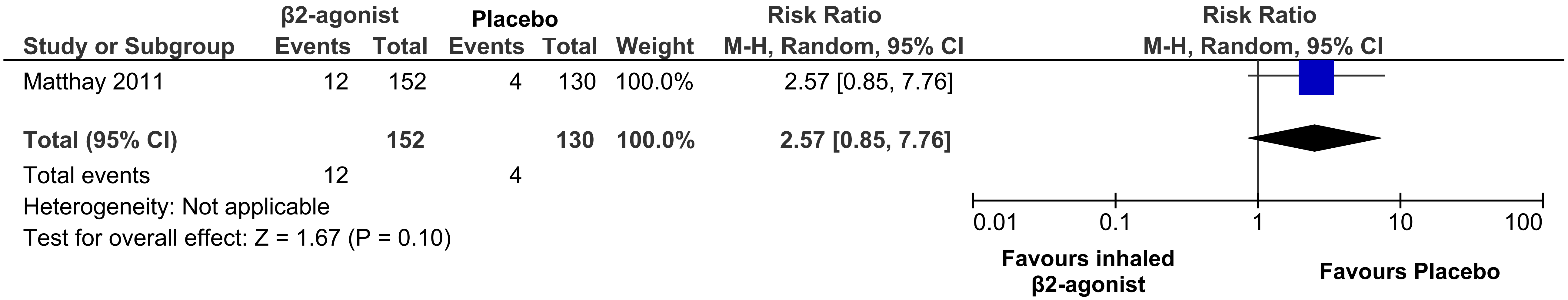

Short term mortality

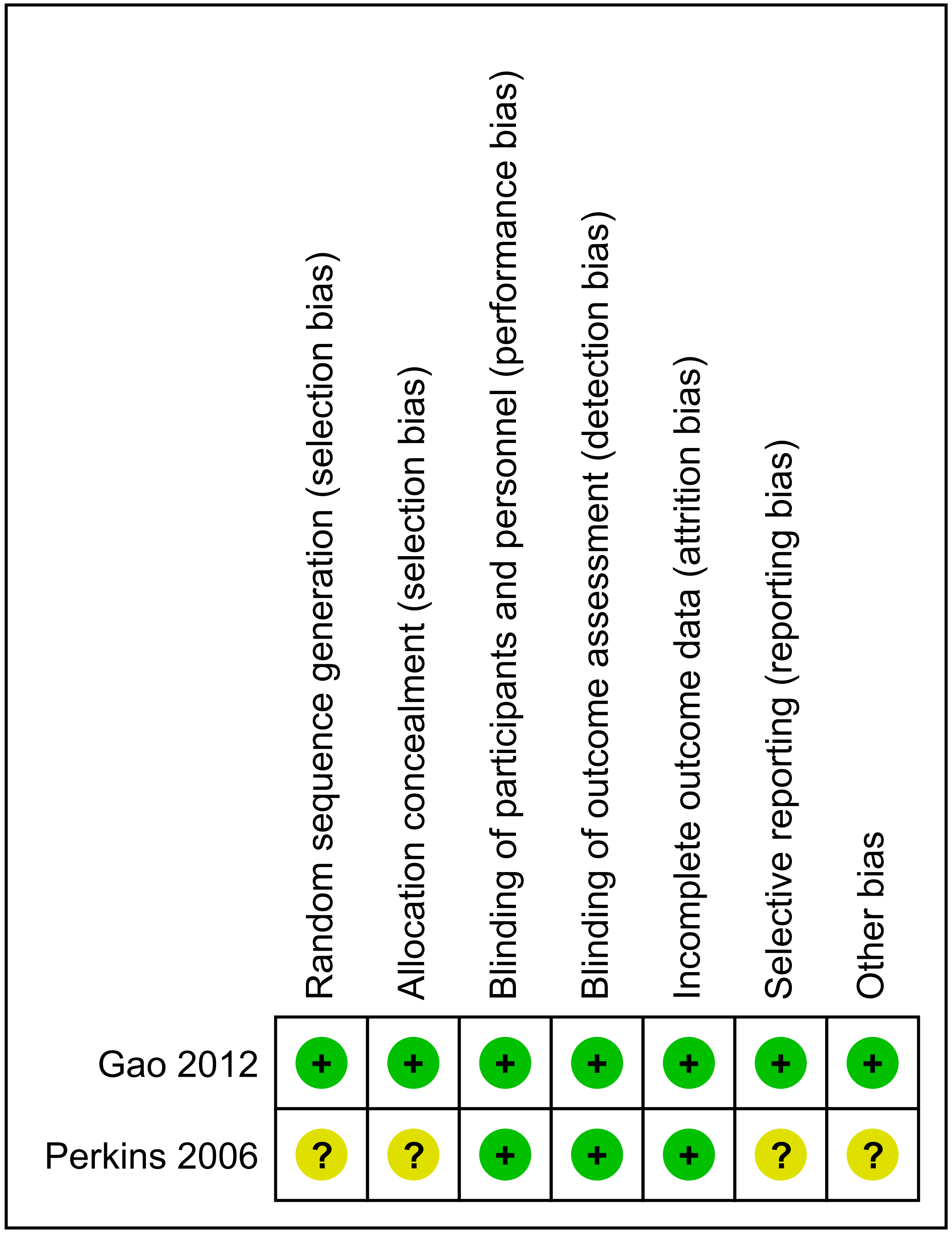

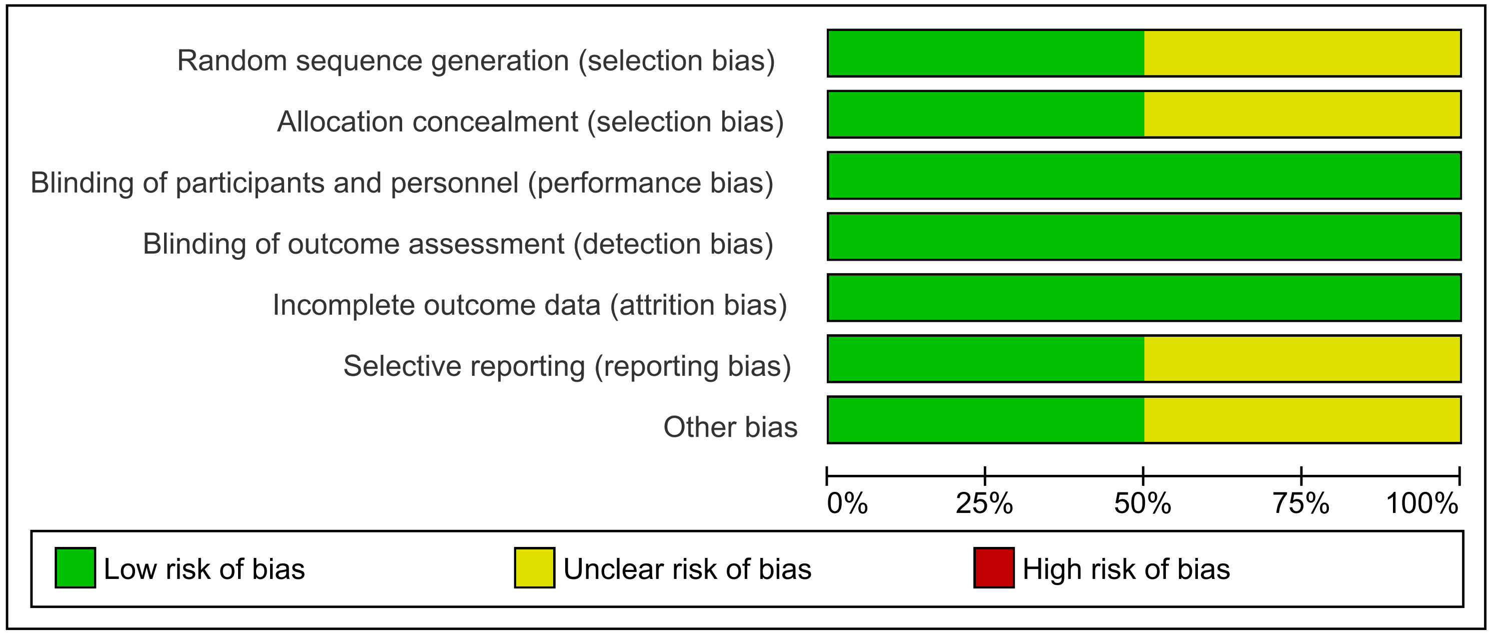

CQ13_03 Risk of bias summary, Risk of bias graph

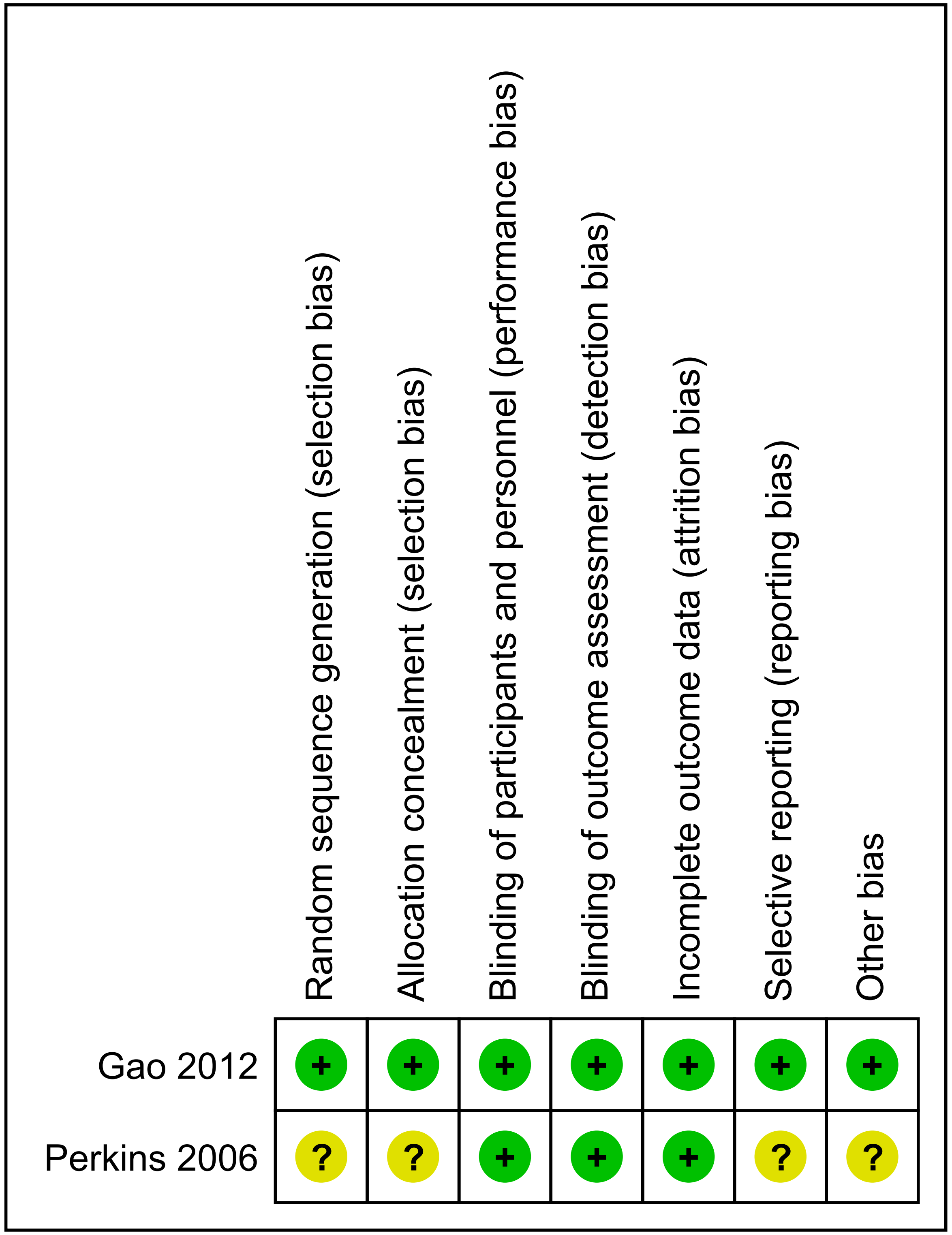

Severe complication

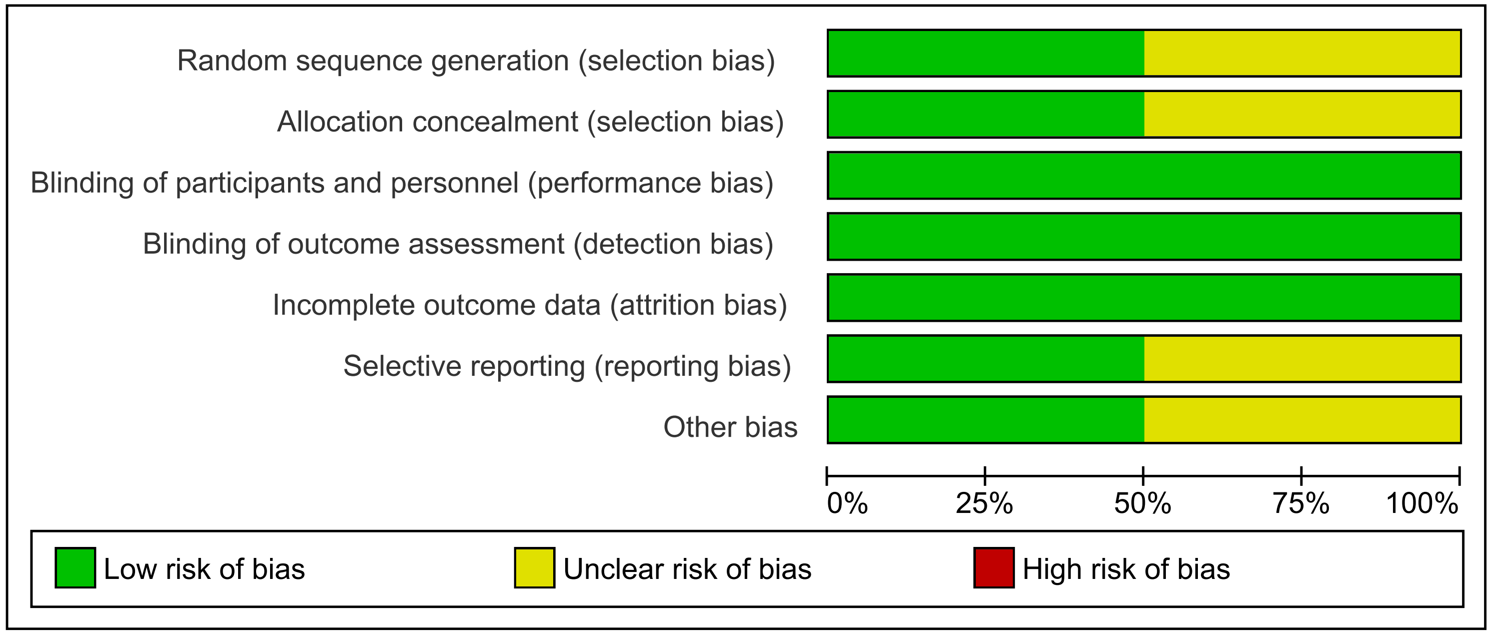

Short term mortality

	β2-agonist		Control			Risk Ratio
Study or Subgroup	Events	Total	Events	Total	Weight	M-H, Random, 95% CI
Gao 2012	55	161	38	163	55.1%	1.47 [1.03, 2.08]
Perkins 2006	11	19	14	21	44.9%	0.87 [0.53, 1.42]
**Total (95% CI)**		**180**		**184**	**100.0%**	**1.16 [0.68, 1.96]**
Total events	66		52			

Heterogeneity: Tau² = 0.10; Chi² = 3.11, df = 1 (P = 0.08); I² = 68%
Test for overall effect: Z = 0.55 (P = 0.58)

Risk Ratio
M-H, Random, 95% CI

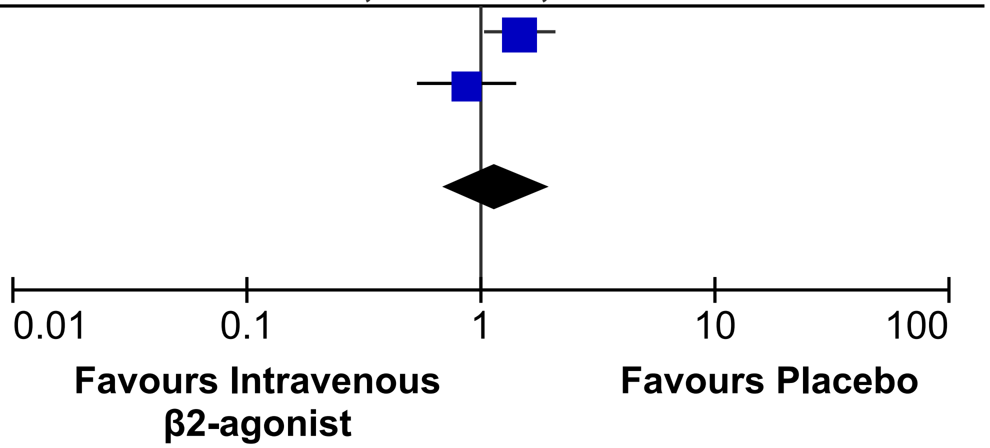

Severe complication

	β2-agonist		Control			Risk Ratio
Study or Subgroup	Events	Total	Events	Total	Weight	M-H, Random, 95% CI
Gao 2012	23	162	2	163	51.5%	11.57 [2.77, 48.28]
Perkins 2006	5	19	2	21	48.5%	2.76 [0.61, 12.61]
**Total (95% CI)**		**181**		**184**	**100.0%**	**5.78 [1.34, 24.92]**
Total events	28		4			

Heterogeneity: Tau² = 0.55; Chi² = 1.97, df = 1 (P = 0.16); I² = 49%
Test for overall effect: Z = 2.35 (P = 0.02)

Risk Ratio
M-H, Random, 95% CI

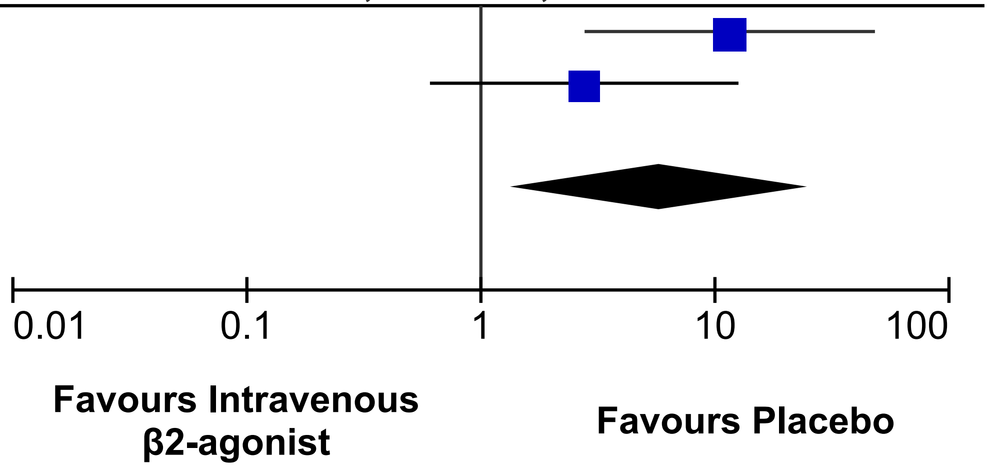

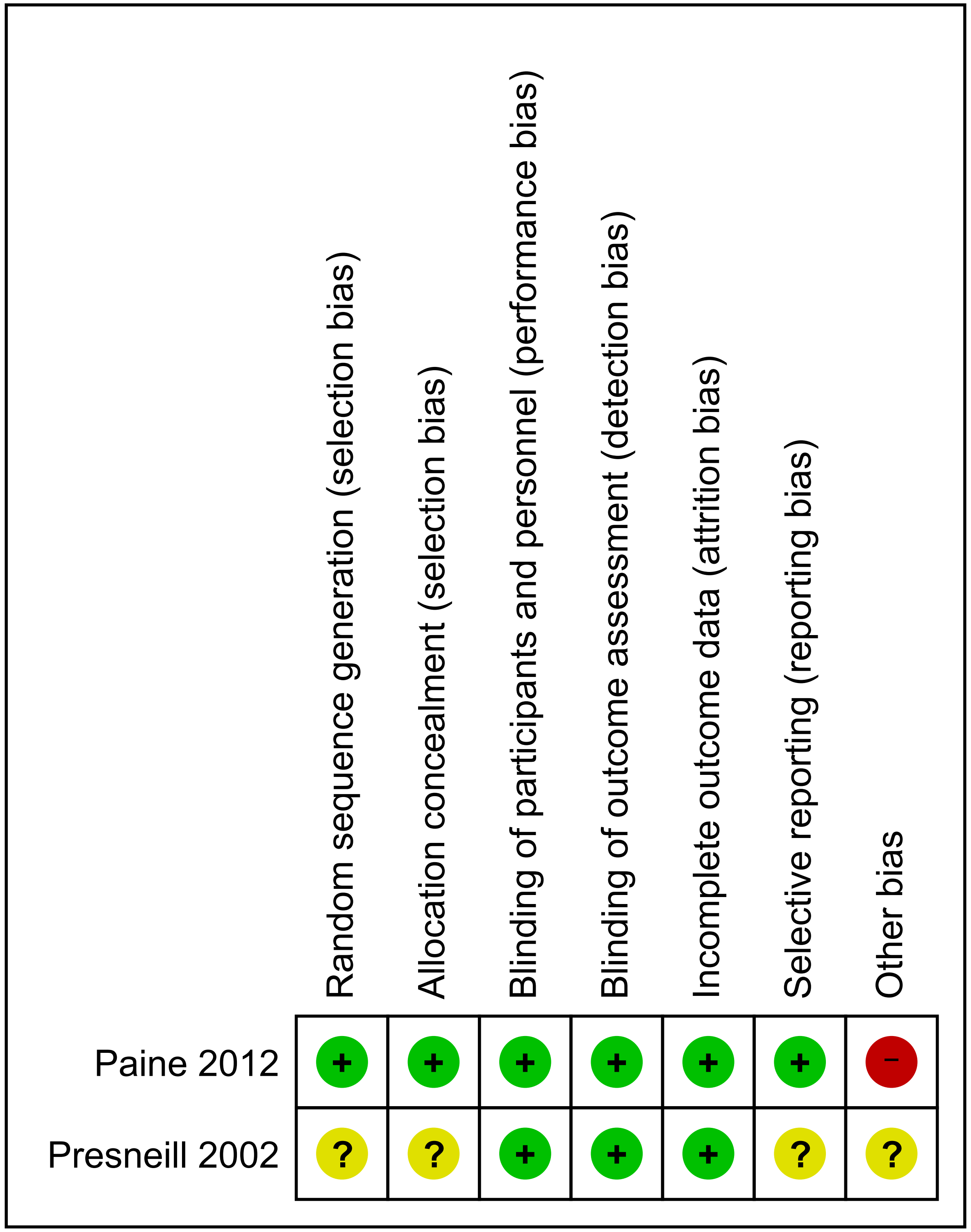

Short term mortality

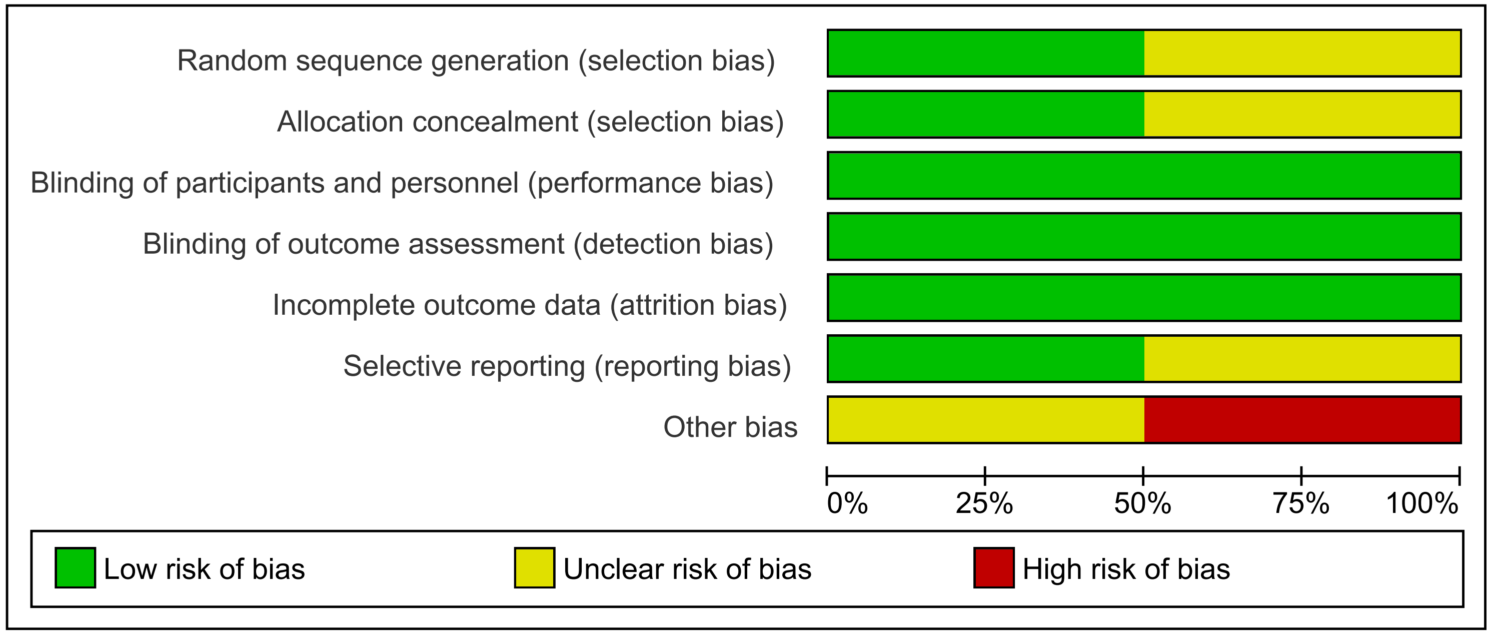

Severe complication

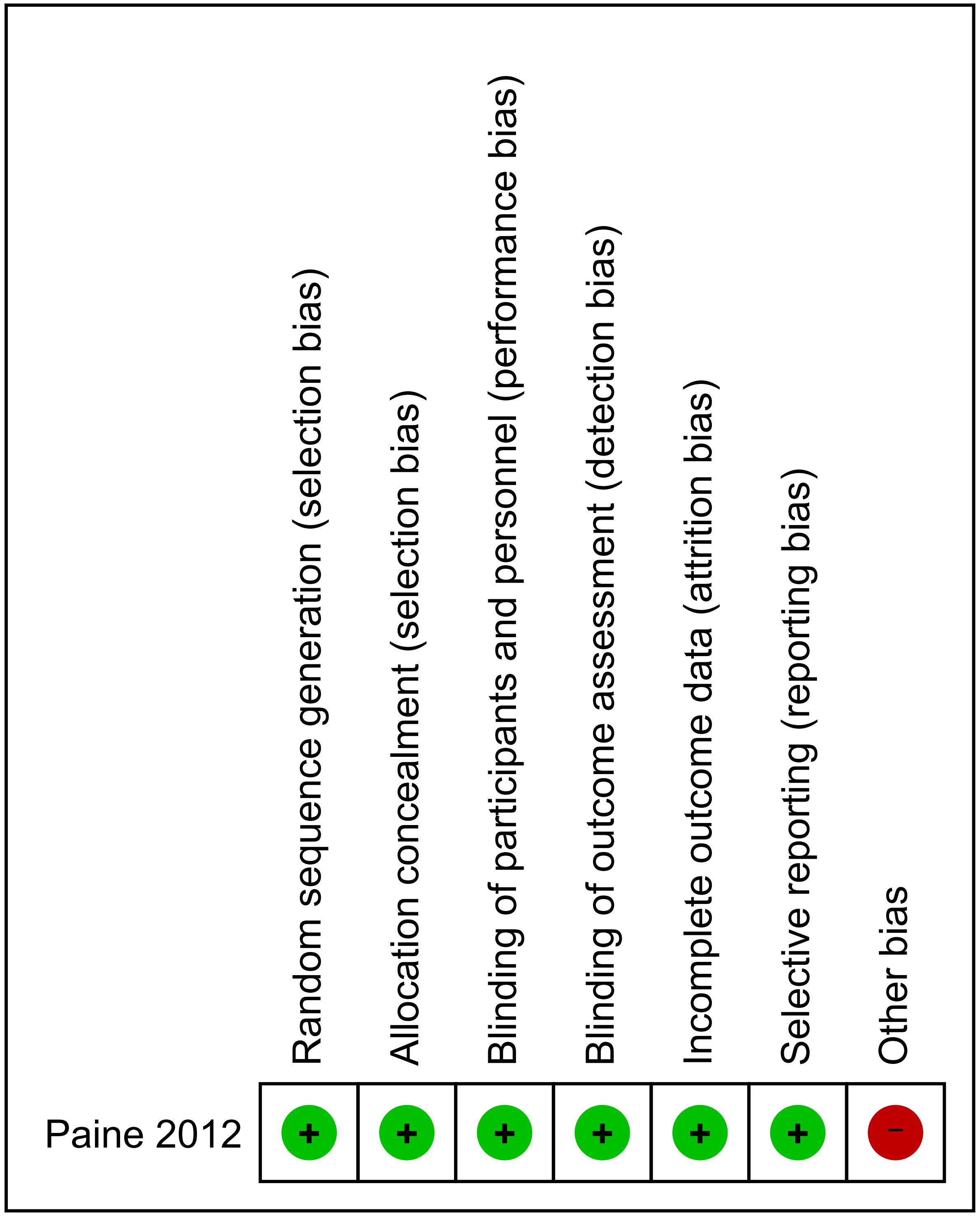

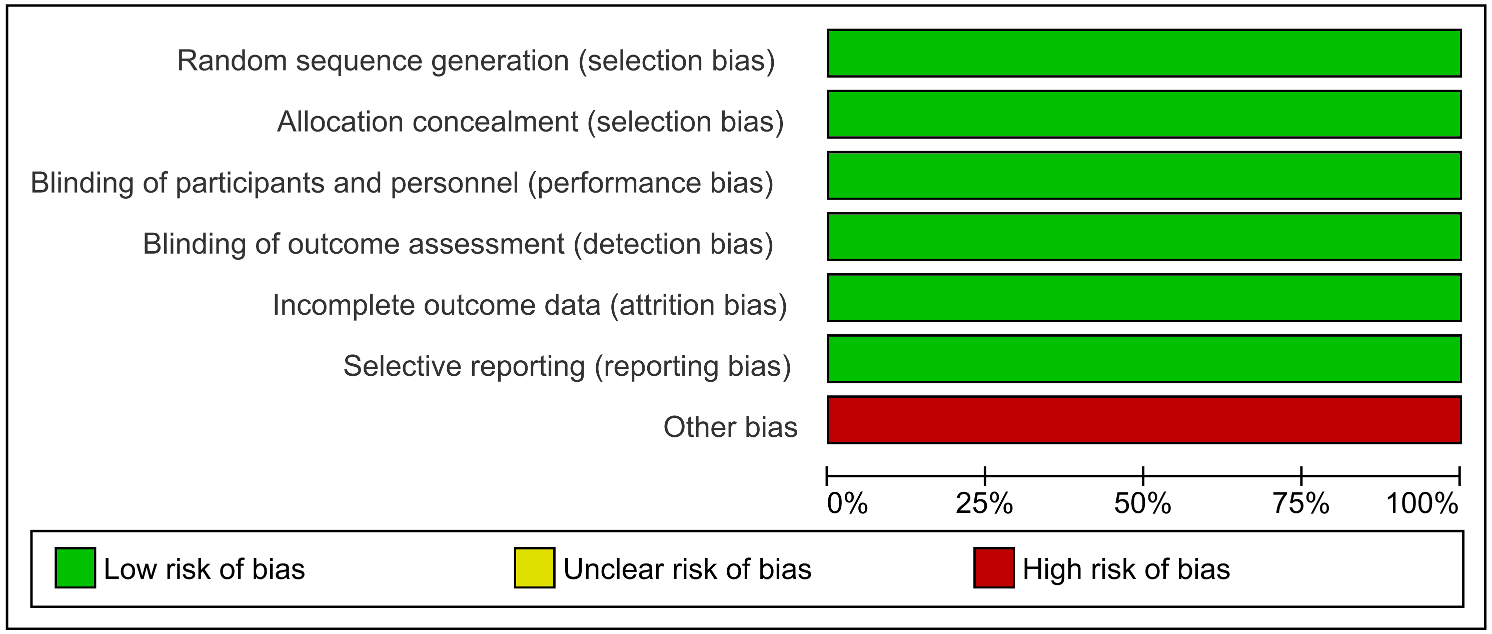

Short term mortality

Study or Subgroup	GM-CSF Events	GM-CSF Total	Placebo Events	Placebo Total	Weight	Risk Ratio M-H, Random, 95% CI
Paine 2012	11	64	15	66	85.9%	0.76 [0.38, 1.52]
Presneill 2002	2	10	2	8	14.1%	0.80 [0.14, 4.49]
**Total (95% CI)**		**74**		**74**	**100.0%**	**0.76 [0.40, 1.46]**
Total events	13		17			

Heterogeneity: Tau² = 0.00; Chi² = 0.00, df = 1 (P = 0.95); I² = 0%
Test for overall effect: Z = 0.82 (P = 0.41)

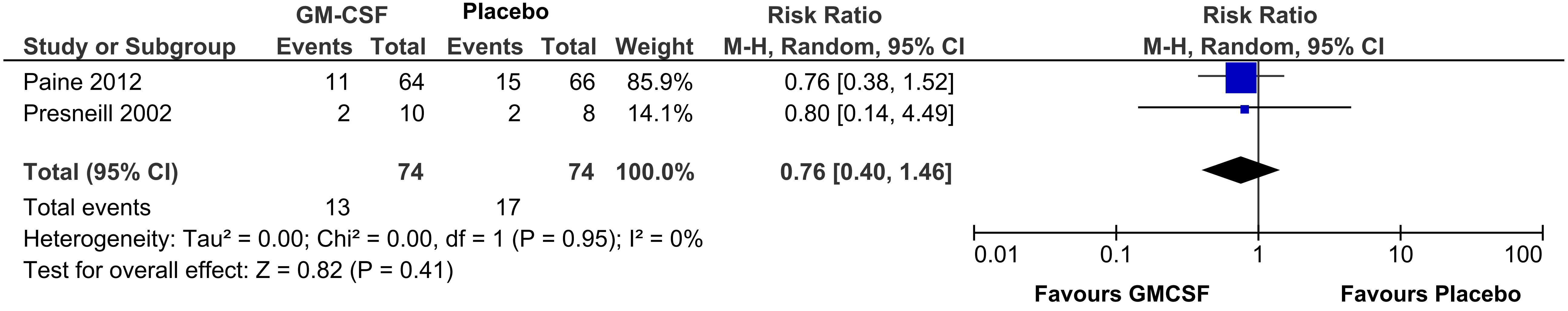

Severe complication

Study or Subgroup	GM-CSF Events	GM-CSF Total	Placebo Events	Placebo Total	Weight	Risk Ratio M-H, Random, 95% CI
Paine 2012	11	64	13	66	100.0%	0.87 [0.42, 1.80]
**Total (95% CI)**		**64**		**66**	**100.0%**	**0.87 [0.42, 1.80]**
Total events	11		13			

Heterogeneity: Not applicable
Test for overall effect: Z = 0.37 (P = 0.71)

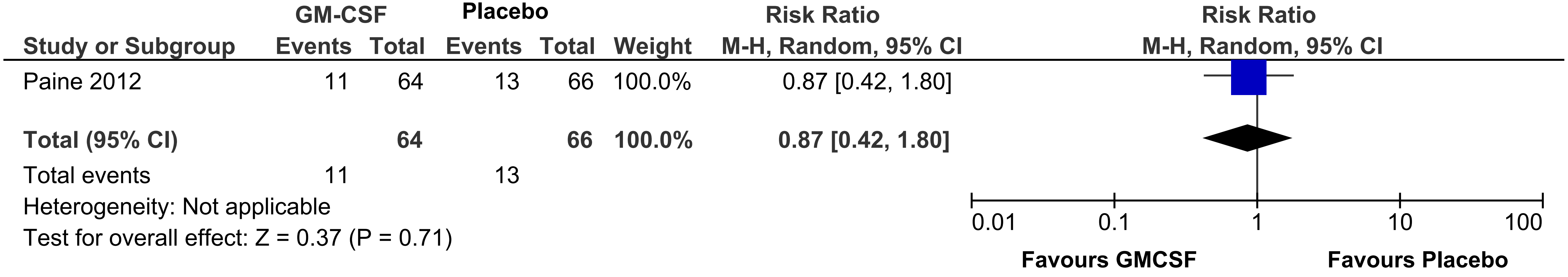

CQ13_05 Risk of bias summary, Risk of bias graph

	Random sequence generation (selection bias)	Allocation concealment (selection bias)	Blinding of participants and personnel (performance bias)	Blinding of outcome assessment (detection bias)	Incomplete outcome data (attrition bias)	Selective reporting (reporting bias)	Other bias
Abraham 1996	?	?	+	+	+	?	?
Abraham 1999	?	?	+	+	+	?	?
Bone 1989	+	+	+	+	+	?	?
Holcroft 1986	+	+	+	+	+	?	?
Rossignon 1990	?	?	+	+	+	?	+
Slotman 1992	+	+	+	+	+	+	+
Vincent 2001	?	?	+	+	+	?	+

Short term mortality

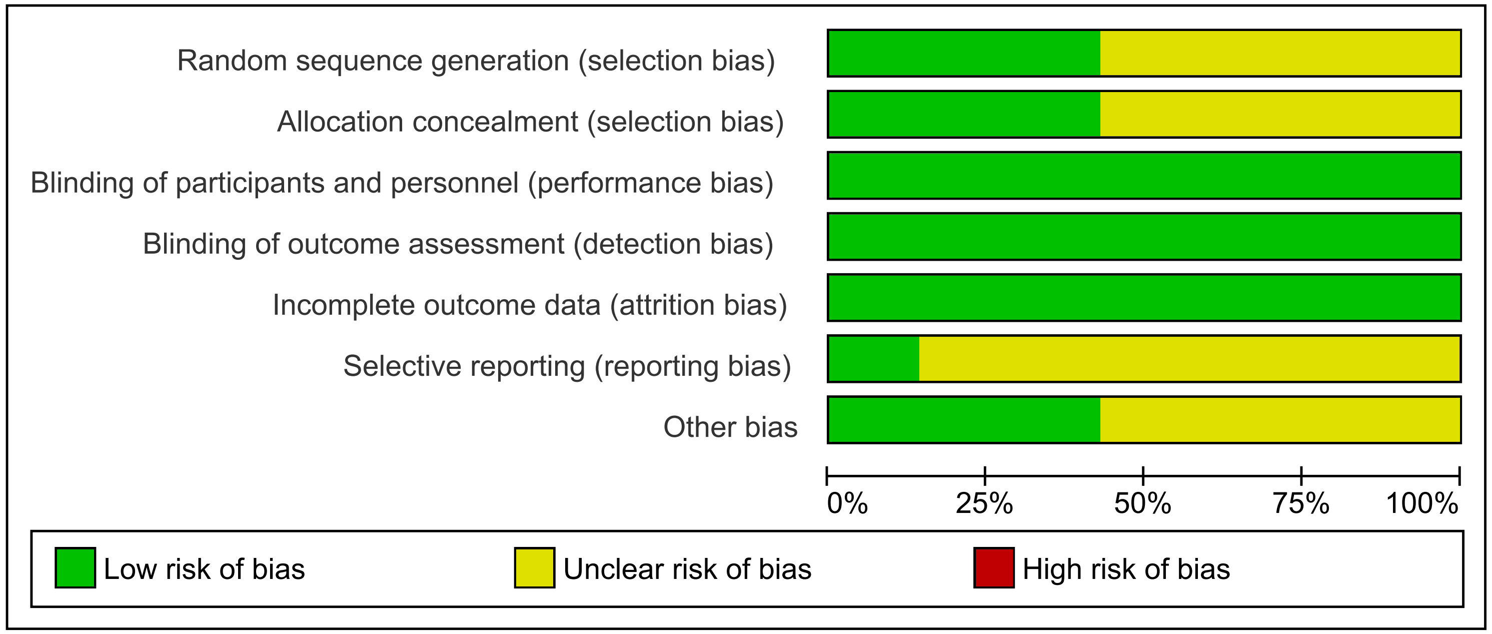

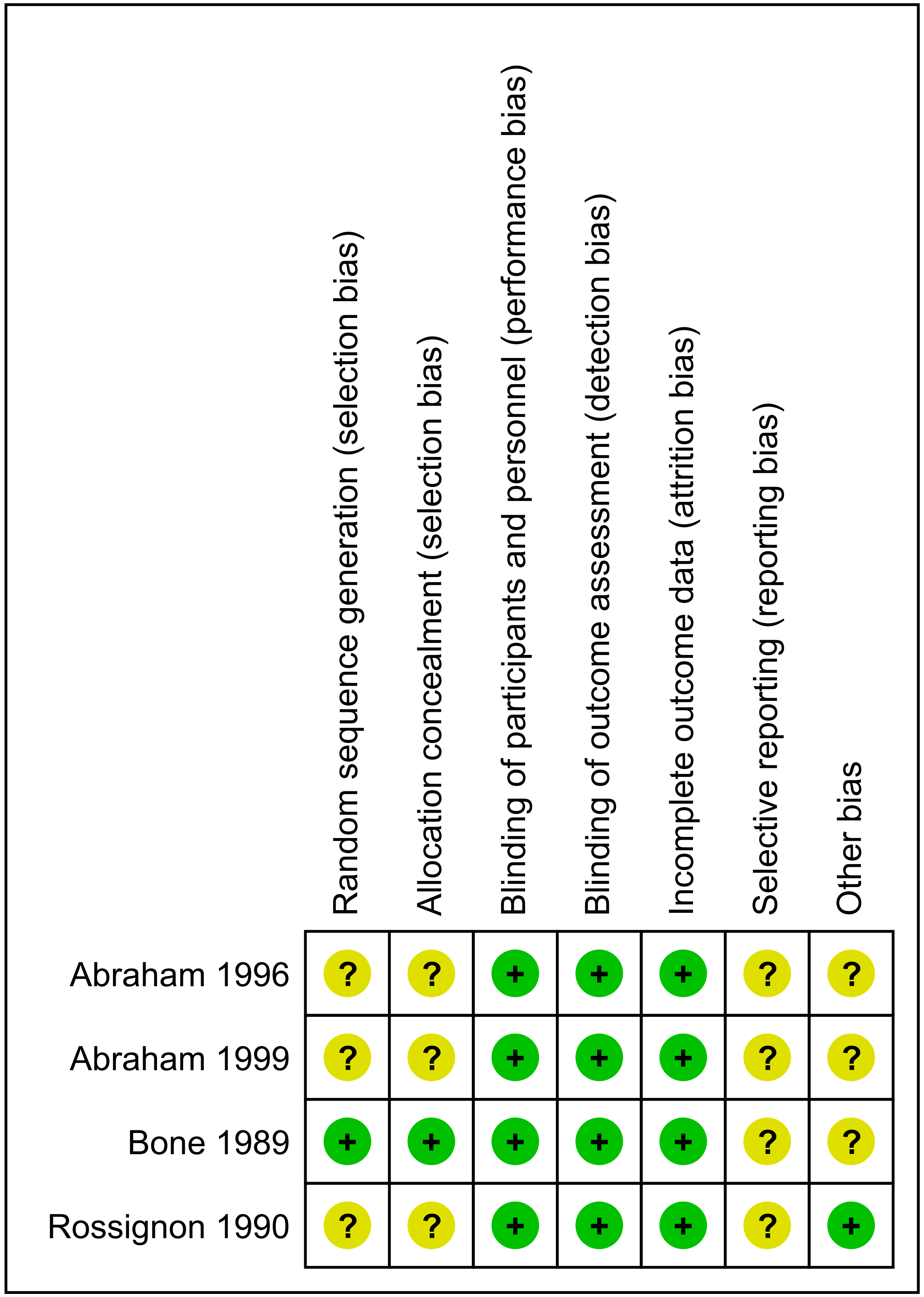

Severe complication

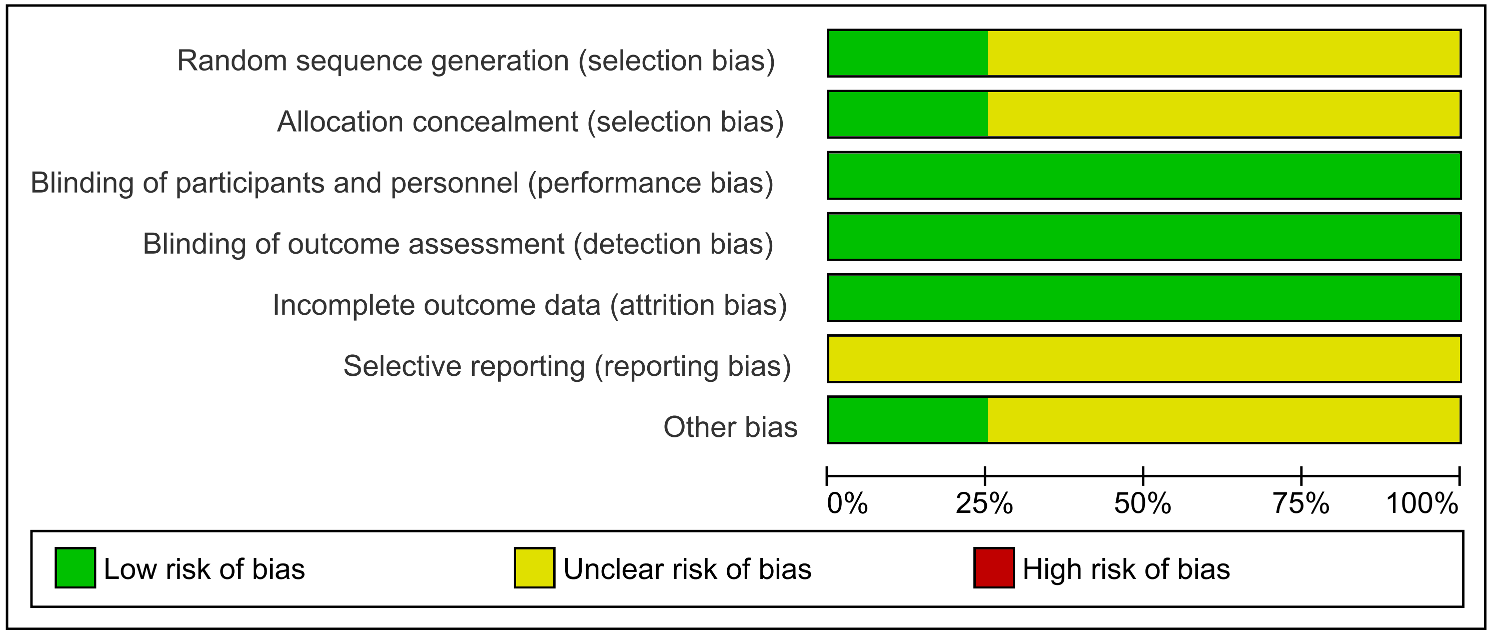

Short term mortality

Study or Subgroup	PGE1 Events	PGE1 Total	Placebo Events	Placebo Total	Weight	Risk Ratio M-H, Random, 95% CI
Abraham 1996	1	17	2	8	0.6%	0.24 [0.02, 2.23]
Abraham 1999	55	178	48	172	26.9%	1.11 [0.80, 1.53]
Bone 1989	30	50	24	50	21.6%	1.25 [0.87, 1.80]
Holcroft 1986	9	20	13	20	9.0%	0.69 [0.39, 1.24]
Rossignon 1990	4	11	7	12	3.7%	0.62 [0.25, 1.56]
Slotman 1992	42	72	37	74	31.1%	1.17 [0.86, 1.57]
Vincent 2001	21	70	9	32	7.1%	1.07 [0.55, 2.06]
**Total (95% CI)**		**418**		**368**	**100.0%**	**1.07 [0.90, 1.28]**
Total events	162		140			

Heterogeneity: Tau² = 0.00; Chi² = 6.29, df = 6 (P = 0.39); I² = 5%
Test for overall effect: Z = 0.76 (P = 0.45)

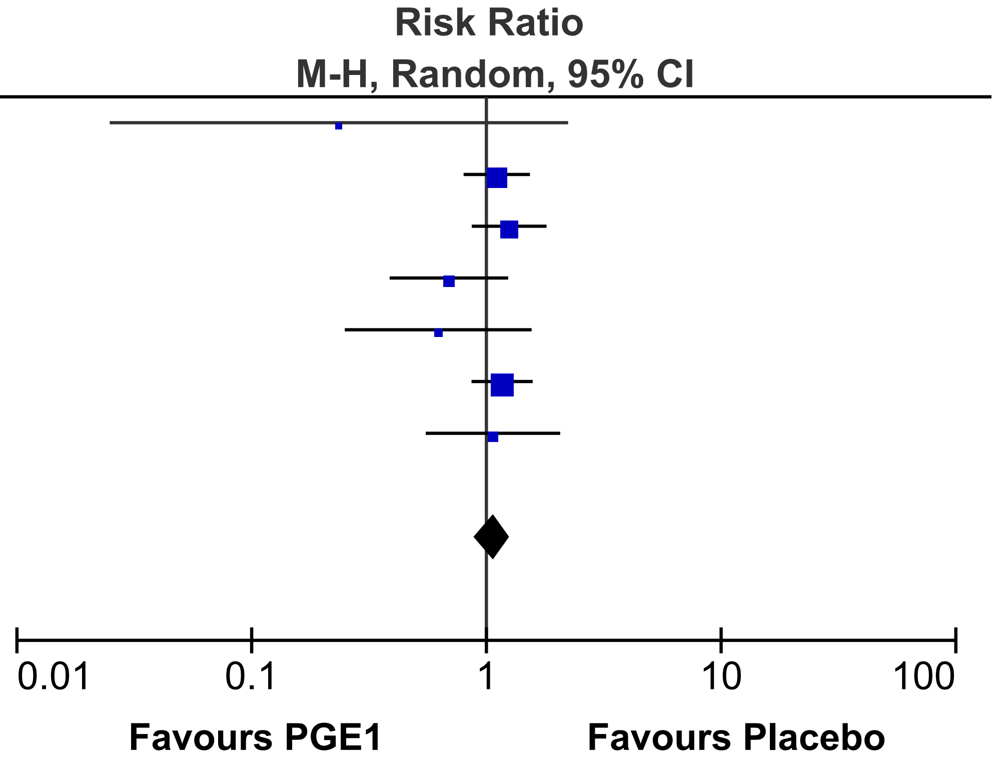

Severe complication

Study or Subgroup	PGE1 Events	PGE1 Total	Placebo Events	Placebo Total	Weight	Risk Ratio M-H, Random, 95% CI
Abraham 1996	7	17	3	8	21.3%	1.10 [0.38, 3.17]
Abraham 1999	93	178	29	172	48.3%	3.10 [2.16, 4.44]
Bone 1989	10	50	7	50	26.4%	1.43 [0.59, 3.45]
Rossignon 1990	2	11	0	12	4.1%	5.42 [0.29, 101.77]
**Total (95% CI)**		**256**		**242**	**100.0%**	**2.07 [1.12, 3.83]**
Total events	112		39			

Heterogeneity: Tau² = 0.17; Chi² = 5.48, df = 3 (P = 0.14); I² = 45%
Test for overall effect: Z = 2.33 (P = 0.02)

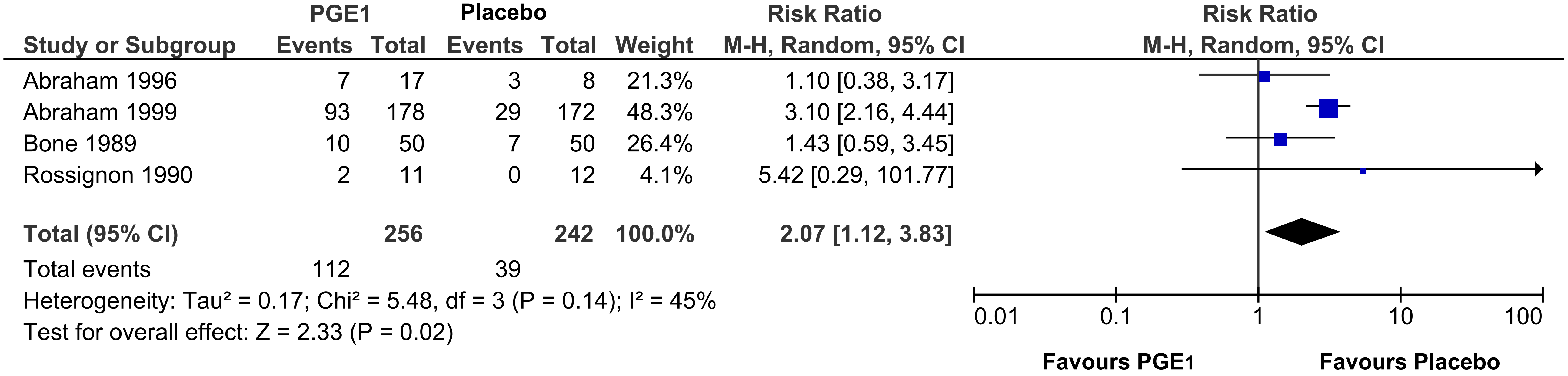

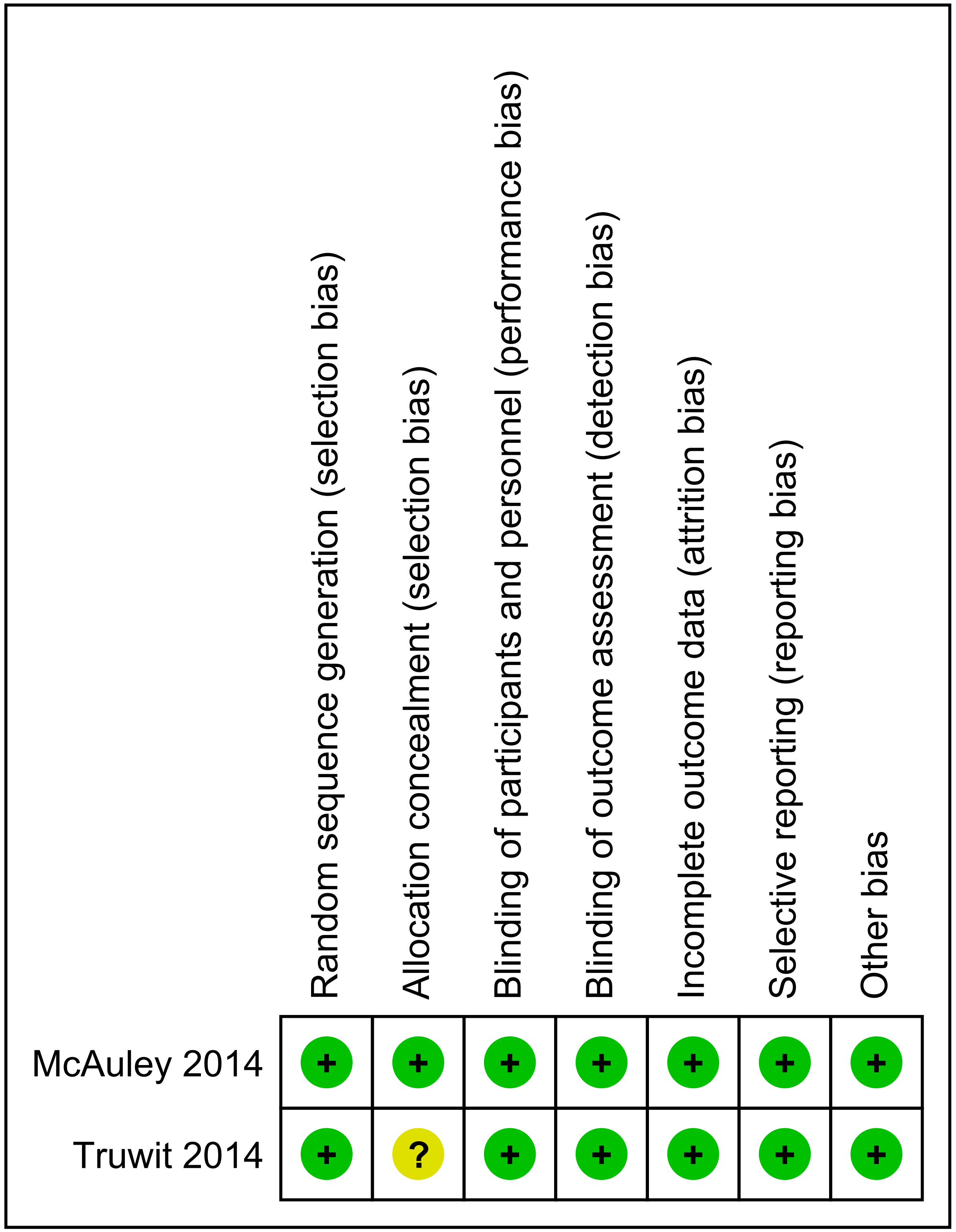

Short term mortality

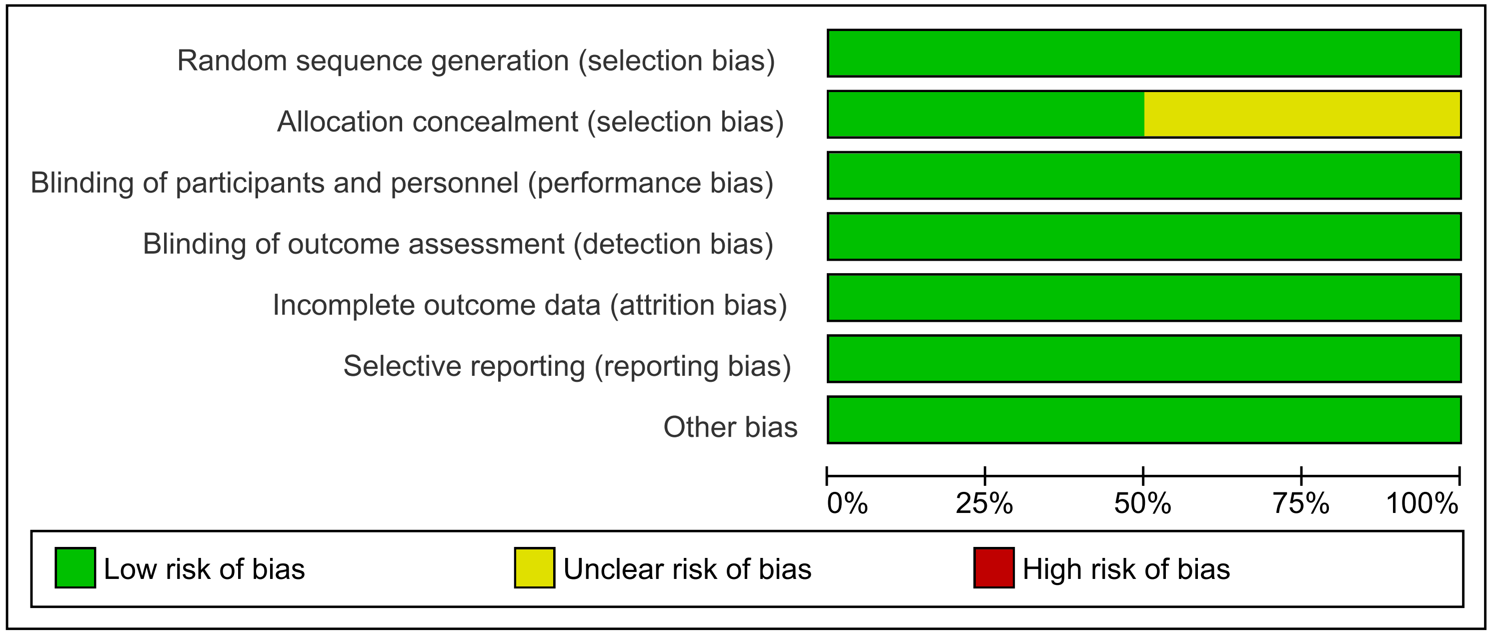

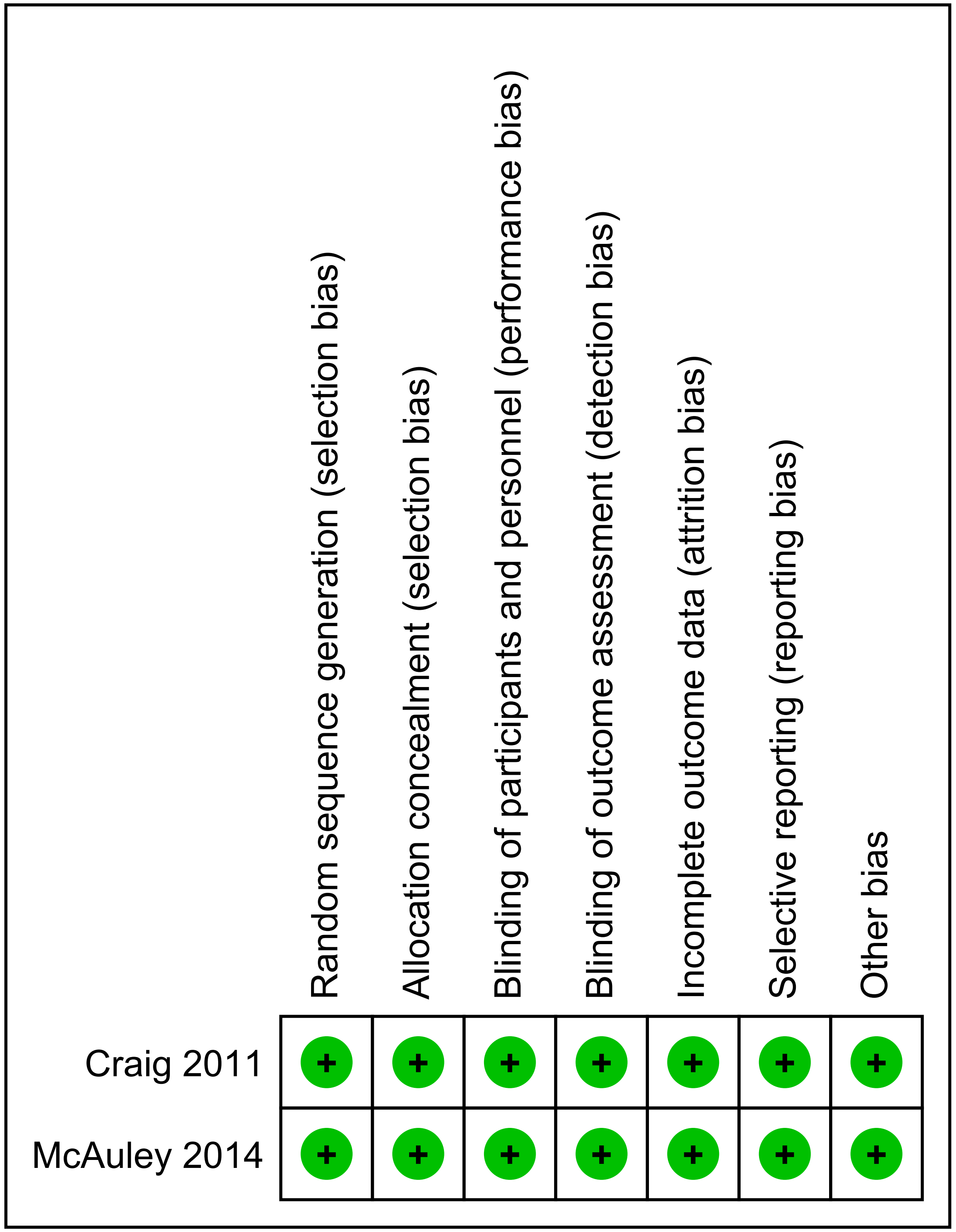

Severe complication

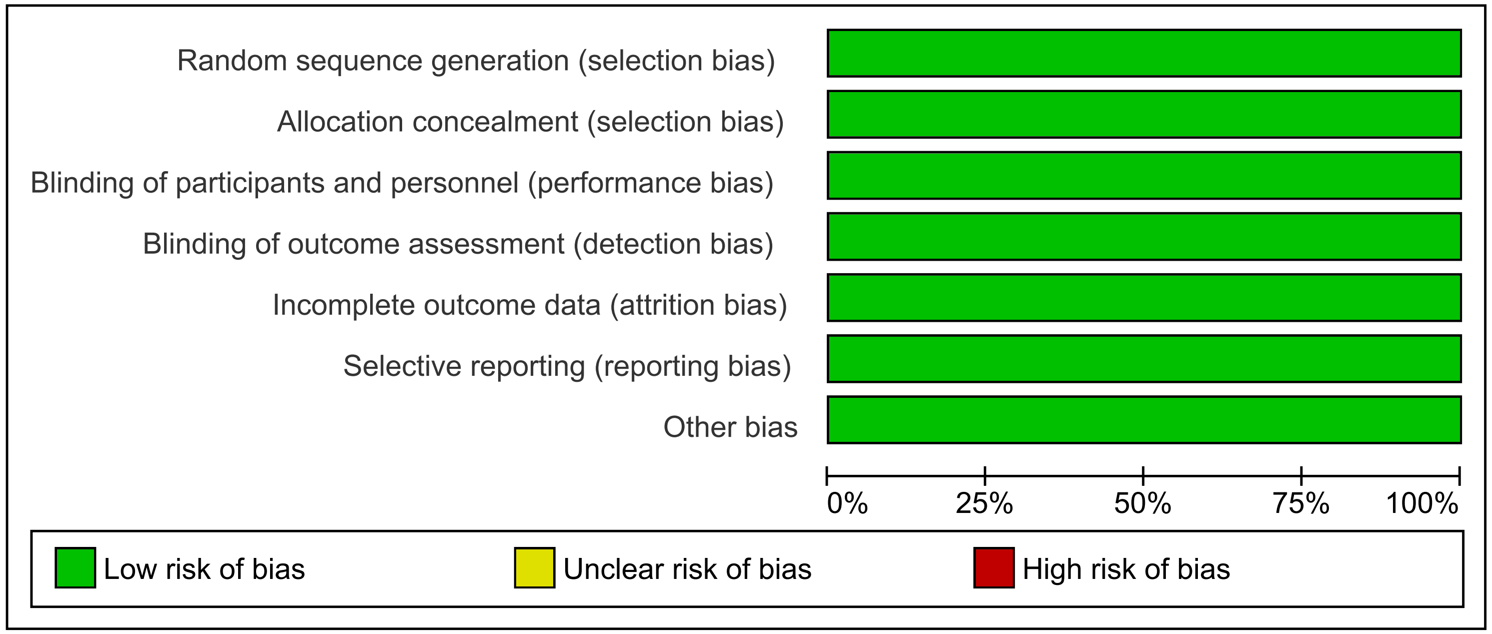

Short term mortality

Study or Subgroup	Statin Events	Statin Total	Placebo Events	Placebo Total	Weight	Risk Ratio M-H, Random, 95% CI
McAuley 2014	57	259	75	280	46.1%	0.82 [0.61, 1.11]
Truwit 2014	108	379	91	366	53.9%	1.15 [0.90, 1.46]
**Total (95% CI)**		**638**		**646**	**100.0%**	**0.98 [0.71, 1.36]**
Total events	165		166			

Heterogeneity: Tau² = 0.04; Chi² = 2.89, df = 1 (P = 0.09); I² = 65%
Test for overall effect: Z = 0.10 (P = 0.92)

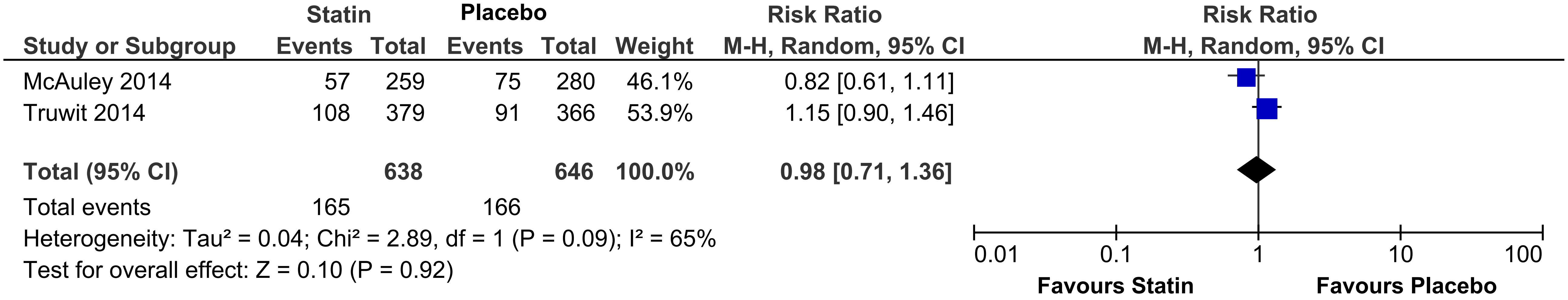

Severe complication

Study or Subgroup	Statin Events	Statin Total	Placebo Events	Placebo Total	Weight	Risk Ratio M-H, Random, 95% CI
Craig 2011	6	30	7	30	35.6%	0.86 [0.33, 2.25]
McAuley 2014	26	259	16	280	64.4%	1.76 [0.96, 3.20]
**Total (95% CI)**		**289**		**310**	**100.0%**	**1.36 [0.69, 2.67]**
Total events	32		23			

Heterogeneity: Tau² = 0.09; Chi² = 1.54, df = 1 (P = 0.21); I² = 35%
Test for overall effect: Z = 0.89 (P = 0.37)

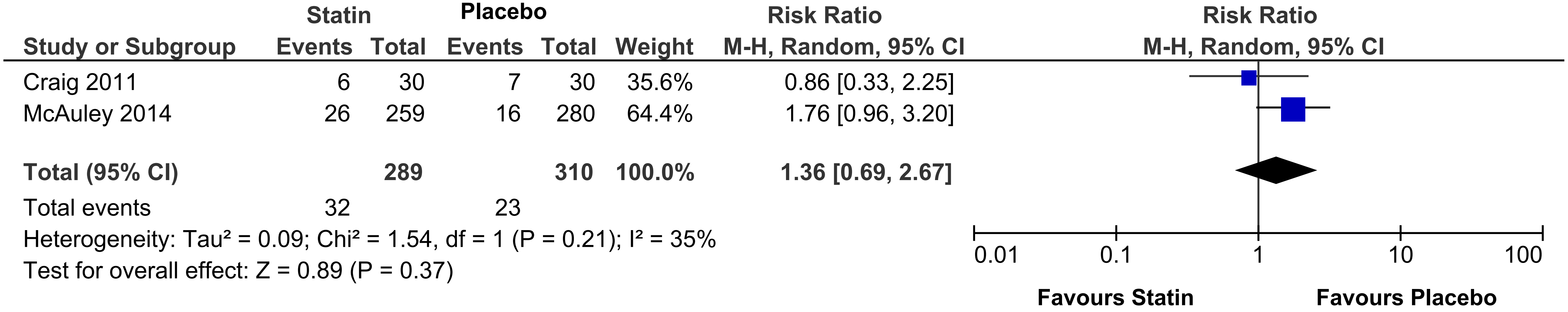

CQ13_07　Risk of bias summary, Risk of bias graph

Short term mortality

	Random sequence generation (selection bias)	Allocation concealment (selection bias)	Blinding of participants and personnel (performance bias)	Blinding of outcome assessment (detection bias)	Incomplete outcome data (attrition bias)	Selective reporting (reporting bias)	Other bias
Anzueto 1996	+	+	+	+	+	?	+
Gregory 1997	?	?	−	−	+	?	+
Kesecioglu 2009	+	+	−	−	+	?	+
Markart 2007	?	?	−	−	+	+	+
Spragg 2003	?	?	−	−	+	?	?
Spragg 2004	?	?	+	+	+	+	+
Spragg 2011	+	+	+	+	+	+	+
Weg 1994	?	?	+	?	+	?	?
Willson 2015	+	?	+	+	+	+	−

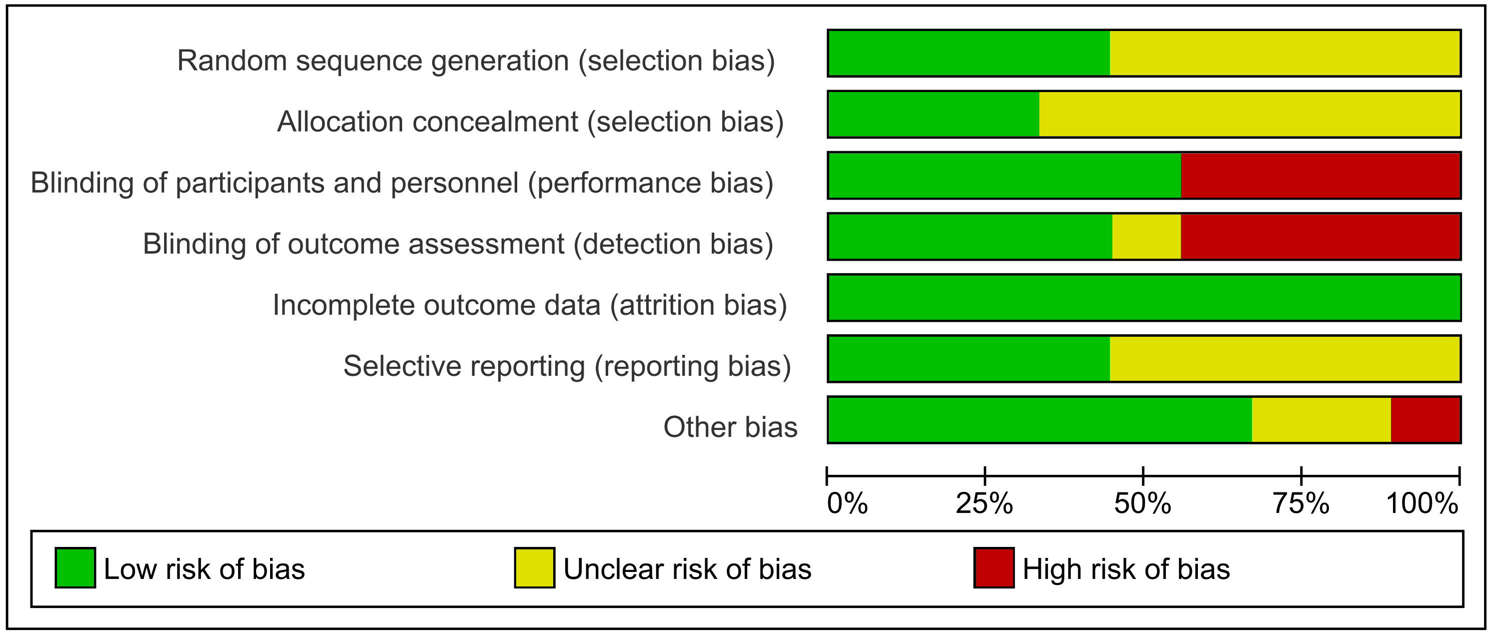

CQ13_07　Risk of bias summary, Risk of bias graph

Severe complication

	Random sequence generation (selection bias)	Allocation concealment (selection bias)	Blinding of participants and personnel (performance bias)	Blinding of outcome assessment (detection bias)	Incomplete outcome data (attrition bias)	Selective reporting (reporting bias)	Other bias
Anzueto 1996	+	+	+	+	+	?	+
Gregory 1997	?	?	−	−	+	?	+
Kesecioglu 2009	+	+	−	−	+	?	+
Spragg 2004	?	?	+	+	+	+	+
Spragg 2011	+	+	+	+	+	+	+
Willson 2015	+	?	+	+	+	+	−

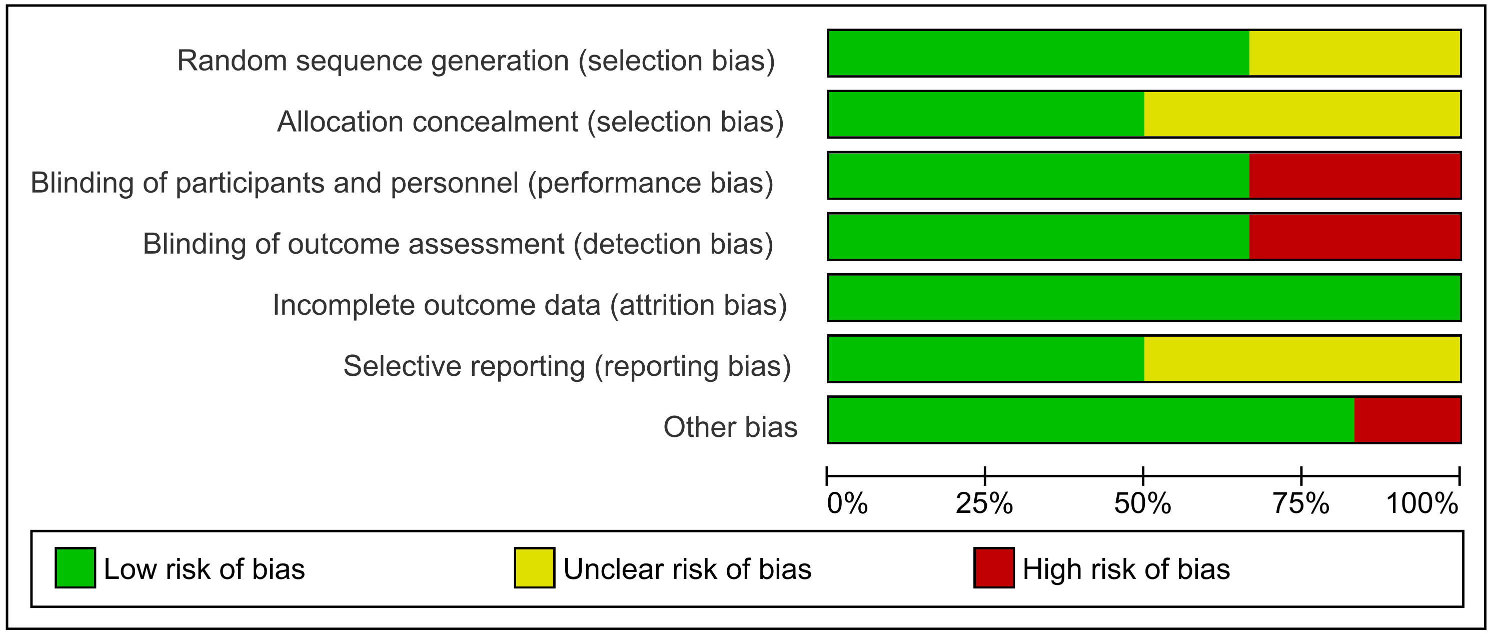

Short term mortality

Study or Subgroup	Surfactant Events	Surfactant Total	Placebo Events	Placebo Total	Weight	Risk Ratio M-H, Random, 95% CI
Anzueto 1996	146	364	144	361	36.3%	1.01 [0.84, 1.20]
Gregory 1997	10	43	7	16	1.9%	0.53 [0.24, 1.16]
Kesecioglu 2009	60	208	51	210	11.2%	1.19 [0.86, 1.64]
Markart 2007	10	14	13	17	6.4%	0.93 [0.61, 1.43]
Spragg 2003	3	15	5	13	0.8%	0.52 [0.15, 1.77]
Spragg 2004	64	224	68	224	14.0%	0.94 [0.71, 1.25]
Spragg 2011	95	419	101	424	19.1%	0.95 [0.74, 1.22]
Weg 1994	6	17	8	17	1.7%	0.75 [0.33, 1.70]
Willson 2015	42	151	41	157	8.5%	1.07 [0.74, 1.54]
**Total (95% CI)**		**1455**		**1439**	**100.0%**	**0.98 [0.88, 1.09]**
Total events	436		438			

Heterogeneity: Tau² = 0.00; Chi² = 5.67, df = 8 (P = 0.68); I² = 0%

Test for overall effect: Z = 0.32 (P = 0.75)

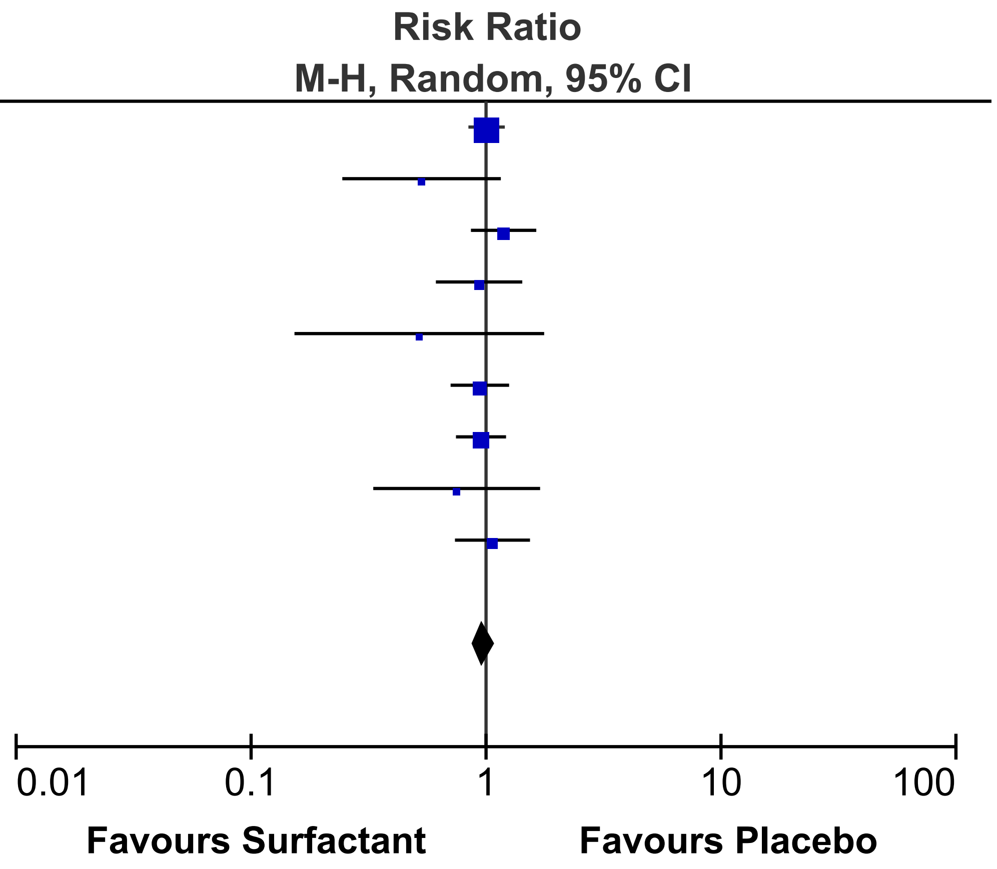

Severe complication

Study or Subgroup	Surfactant Events	Surfactant Total	Placebo Events	Placebo Total	Weight	Risk Ratio M-H, Random, 95% CI
Anzueto 1996	5	364	3	361	5.9%	1.65 [0.40, 6.87]
Gregory 1997	7	43	0	16	1.7%	5.80 [0.35, 96.02]
Kesecioglu 2009	157	208	116	210	40.0%	1.37 [1.18, 1.58]
Spragg 2004	10	224	4	224	8.5%	2.50 [0.80, 7.85]
Spragg 2011	139	419	146	424	38.3%	0.96 [0.80, 1.16]
Willson 2015	15	151	2	157	5.6%	7.80 [1.81, 33.52]
**Total (95% CI)**		**1409**		**1392**	**100.0%**	**1.44 [0.99, 2.09]**
Total events	333		271			

Heterogeneity: Tau² = 0.08; Chi² = 17.43, df = 5 (P = 0.004); I² = 71%

Test for overall effect: Z = 1.91 (P = 0.06)

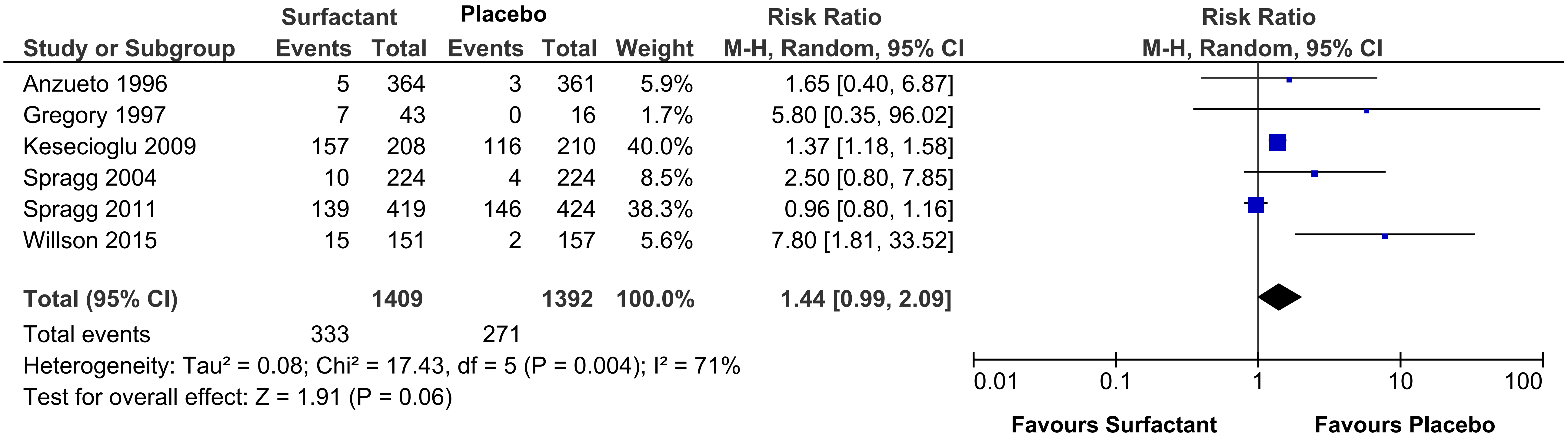

CQ13_08 Risk of bias summary, Risk of bias graph

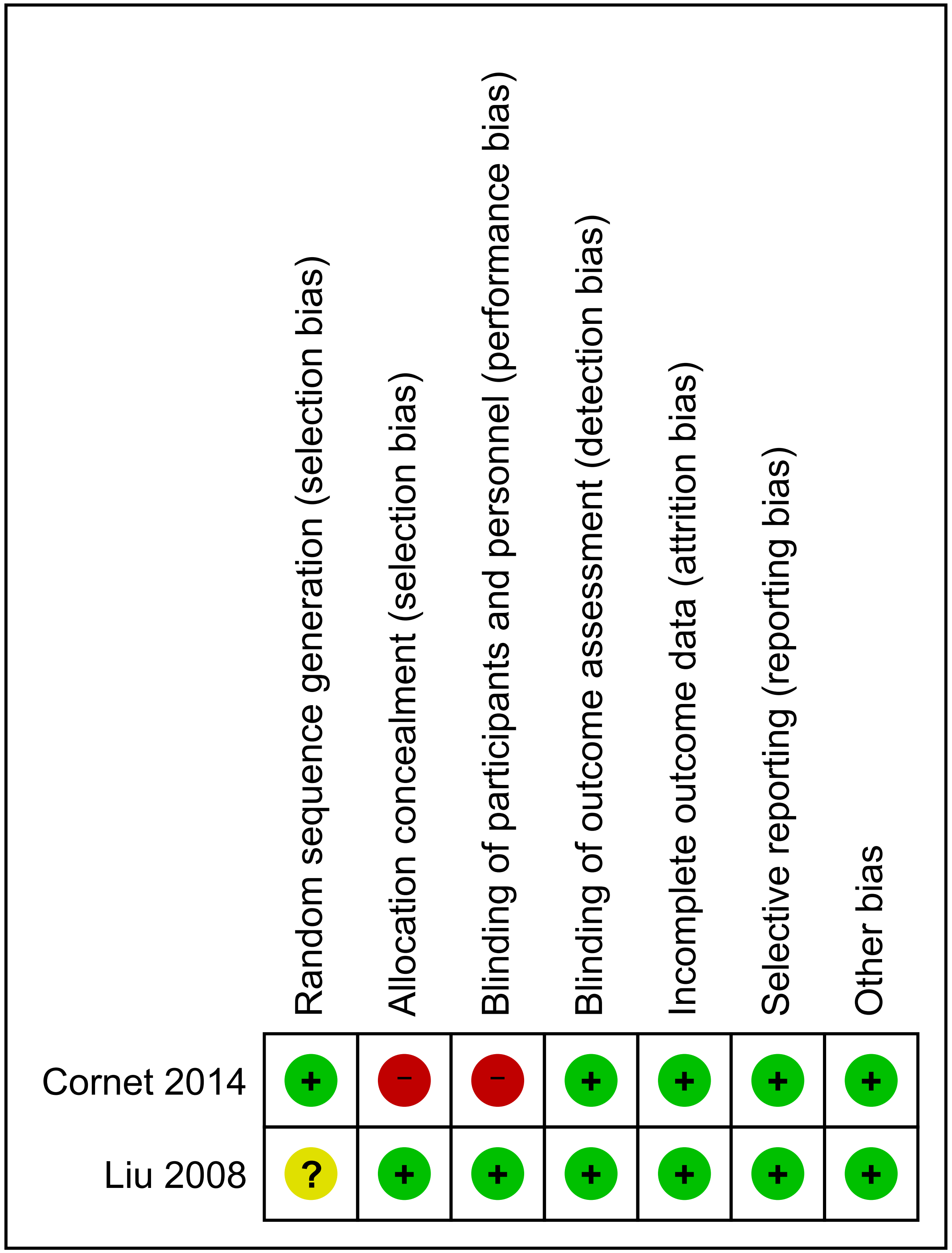

Short term mortality

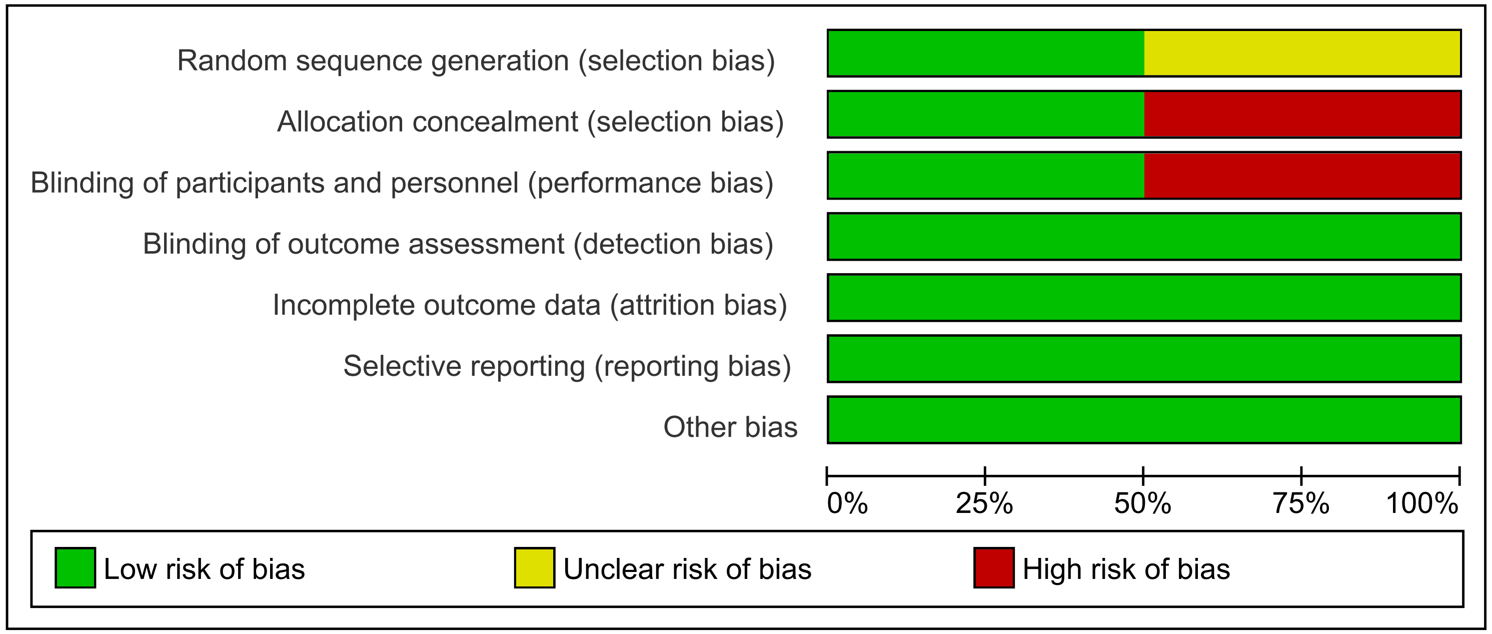

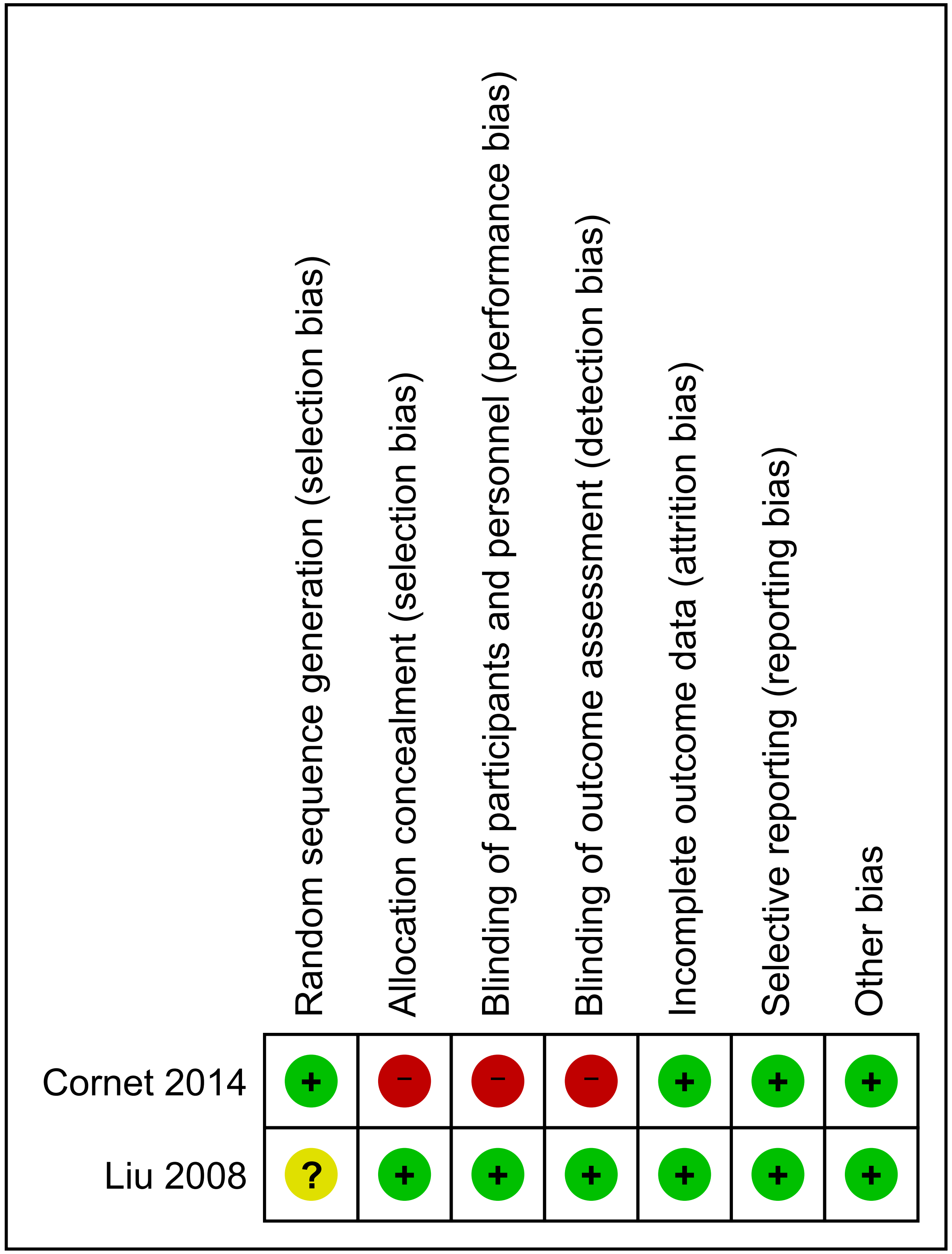

Severe complication

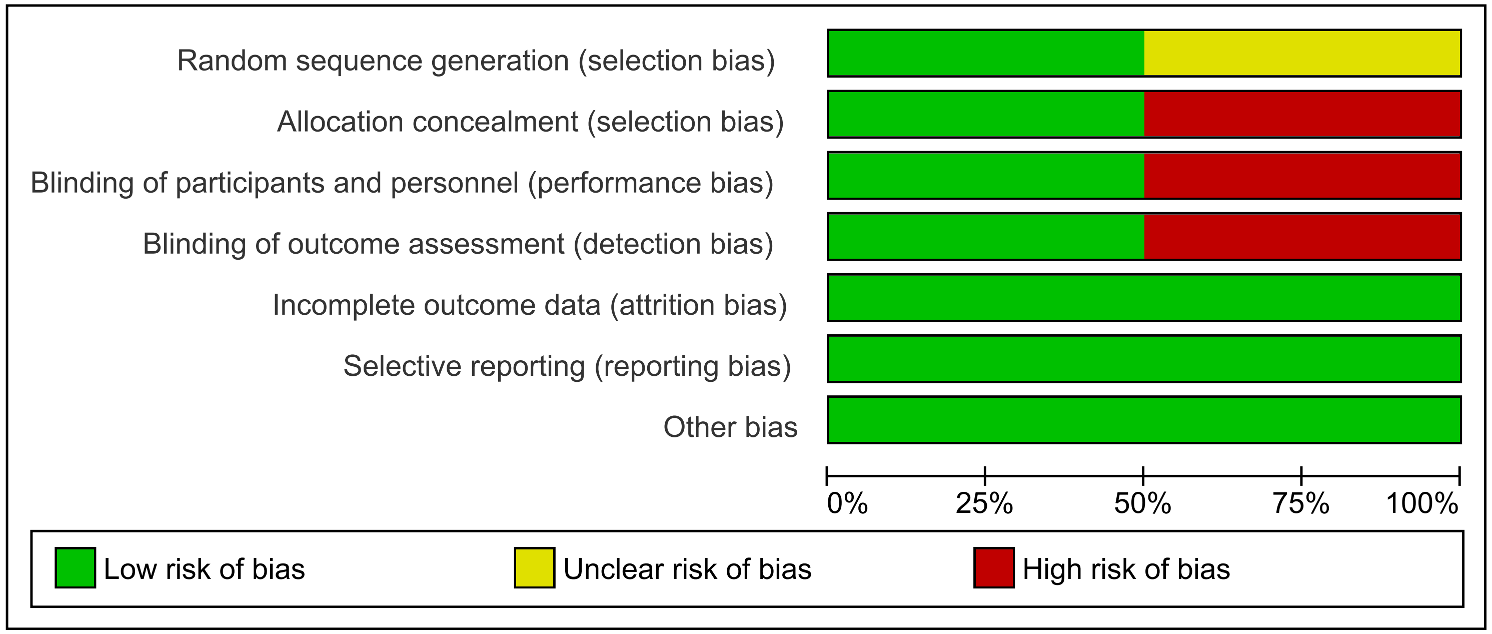

Short term mortality

Study or Subgroup	APC Events	APC Total	Placebo Events	Placebo Total	Weight	Risk Ratio M-H, Random, 95% CI
Cornet 2014	2	33	7	38	40.9%	0.33 [0.07, 1.48]
Liu 2008	5	37	5	38	59.1%	1.03 [0.32, 3.26]
**Total (95% CI)**		**70**		**76**	**100.0%**	**0.64 [0.21, 1.95]**
Total events	7		12			

Heterogeneity: Tau² = 0.19; Chi² = 1.41, df = 1 (P = 0.24); I² = 29%
Test for overall effect: Z = 0.78 (P = 0.44)

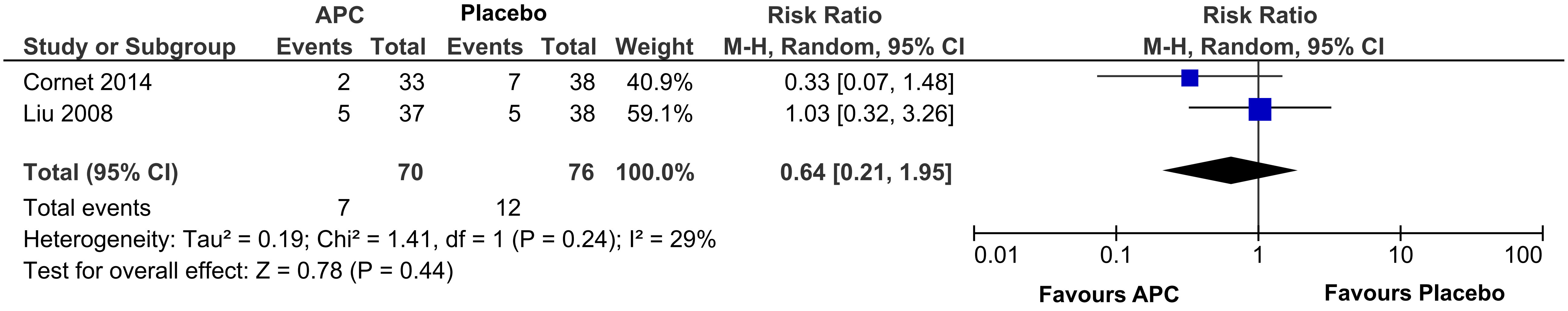

Severe complication

Study or Subgroup	APC Events	APC Total	Placebo Events	Placebo Total	Weight	Risk Ratio M-H, Random, 95% CI
Cornet 2014	2	33	6	38	14.5%	0.38 [0.08, 1.77]
Liu 2008	12	37	14	38	85.5%	0.88 [0.47, 1.64]
**Total (95% CI)**		**70**		**76**	**100.0%**	**0.78 [0.43, 1.40]**
Total events	14		20			

Heterogeneity: Tau² = 0.00; Chi² = 1.01, df = 1 (P = 0.32); I² = 1%
Test for overall effect: Z = 0.83 (P = 0.41)

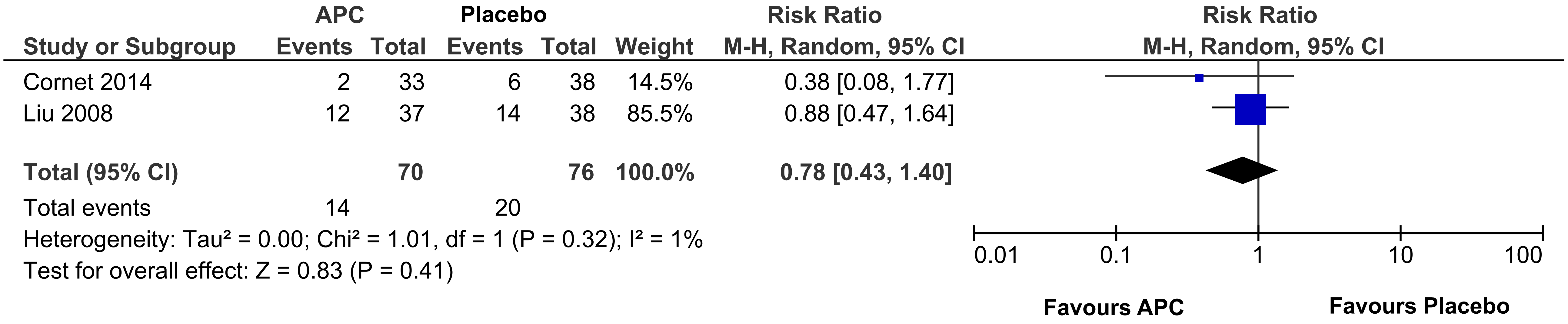

CQ13_09 Risk of bias summary, Risk of bias graph

Short term mortality

	Random sequence generation (selection bias)	Allocation concealment (selection bias)	Blinding of participants and personnel (performance bias)	Blinding of outcome assessment (detection bias)	Incomplete outcome data (attrition bias)	Selective reporting (reporting bias)	Other bias
Bernard 1997	+	+	+	+	+	?	+
Jepsen 1992	?	?	+	+	+	?	+
Ortolani 2000	?	?	−	−	+	?	?
Suter 1994	?	?	+	+	+	?	?

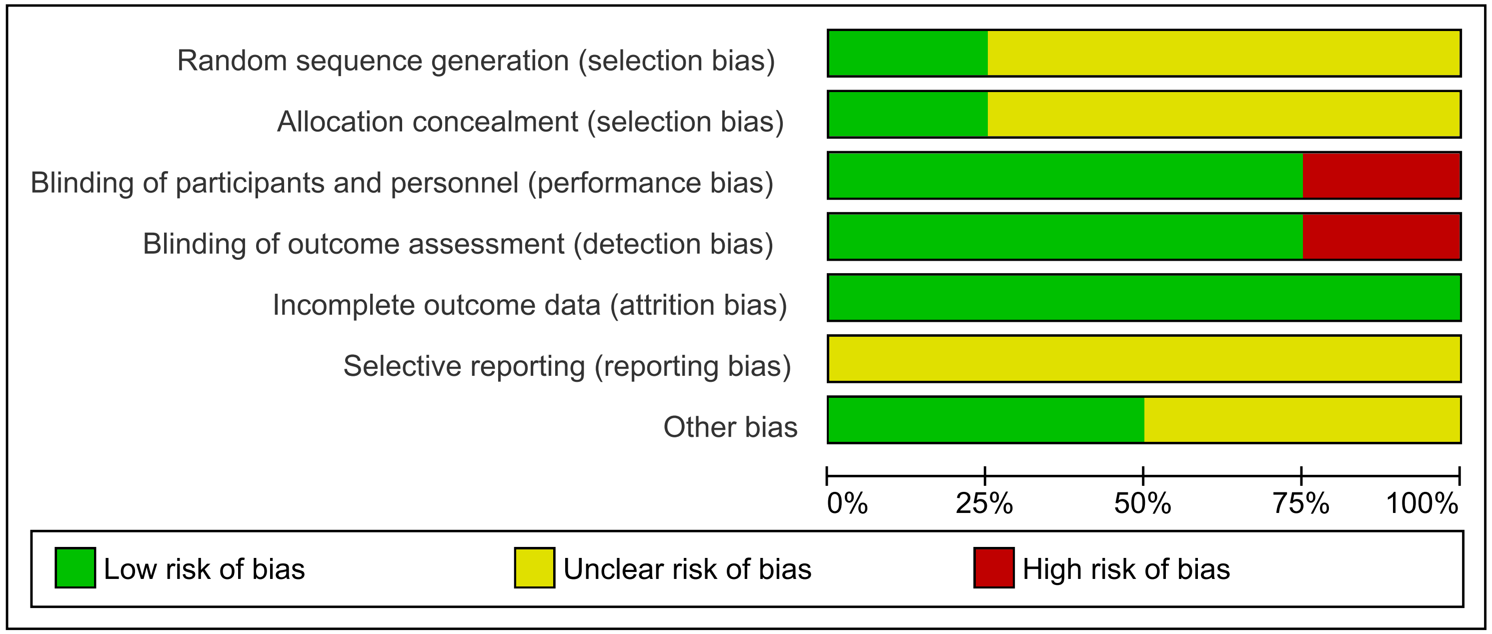

Short term mortality

Study or Subgroup	NAC Events	NAC Total	Placebo Events	Placebo Total	Weight	Risk Ratio M-H, Random, 95% CI
Bernard 1997	5	14	6	15	13.2%	0.89 [0.35, 2.28]
Jepsen 1992	17	32	17	34	52.8%	1.06 [0.67, 1.70]
Ortolani 2000	5	12	7	12	17.1%	0.71 [0.31, 1.63]
Suter 1994	7	32	10	29	17.0%	0.63 [0.28, 1.45]
**Total (95% CI)**		**90**		**90**	**100.0%**	**0.89 [0.63, 1.25]**
Total events	34		40			

Heterogeneity: Tau² = 0.00; Chi² = 1.50, df = 3 (P = 0.68); I² = 0%
Test for overall effect: Z = 0.68 (P = 0.50)

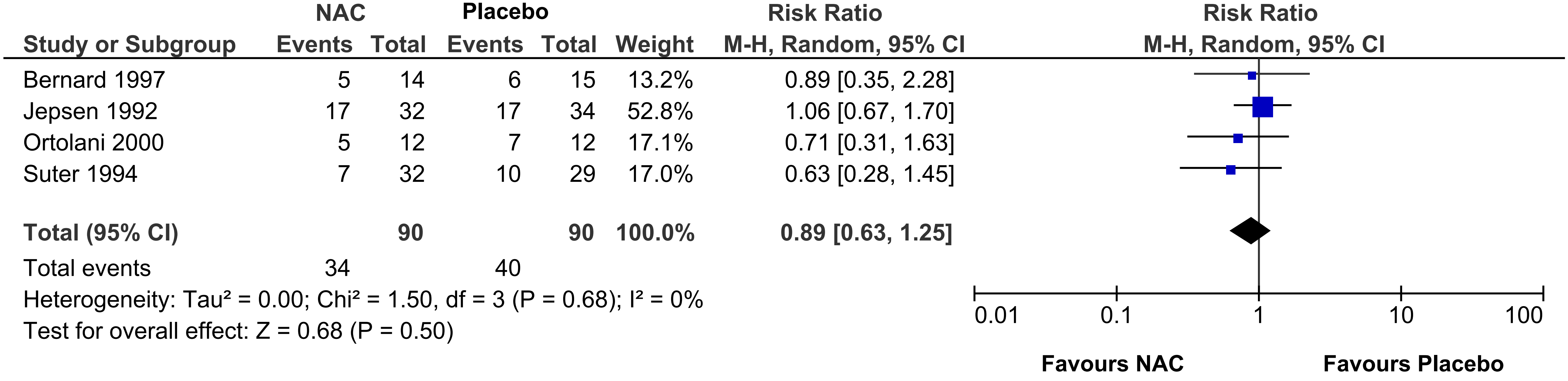

Short term mortality

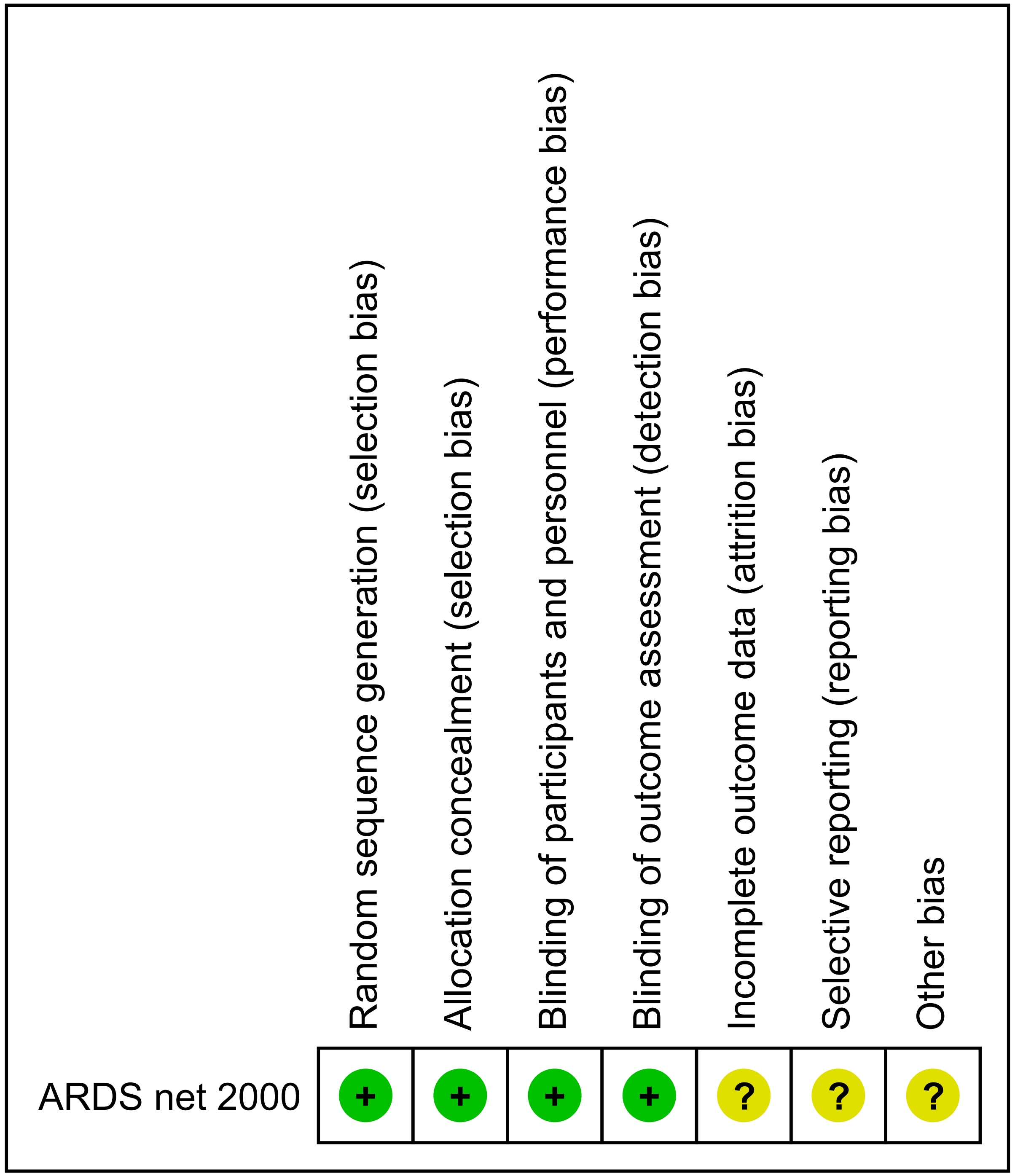

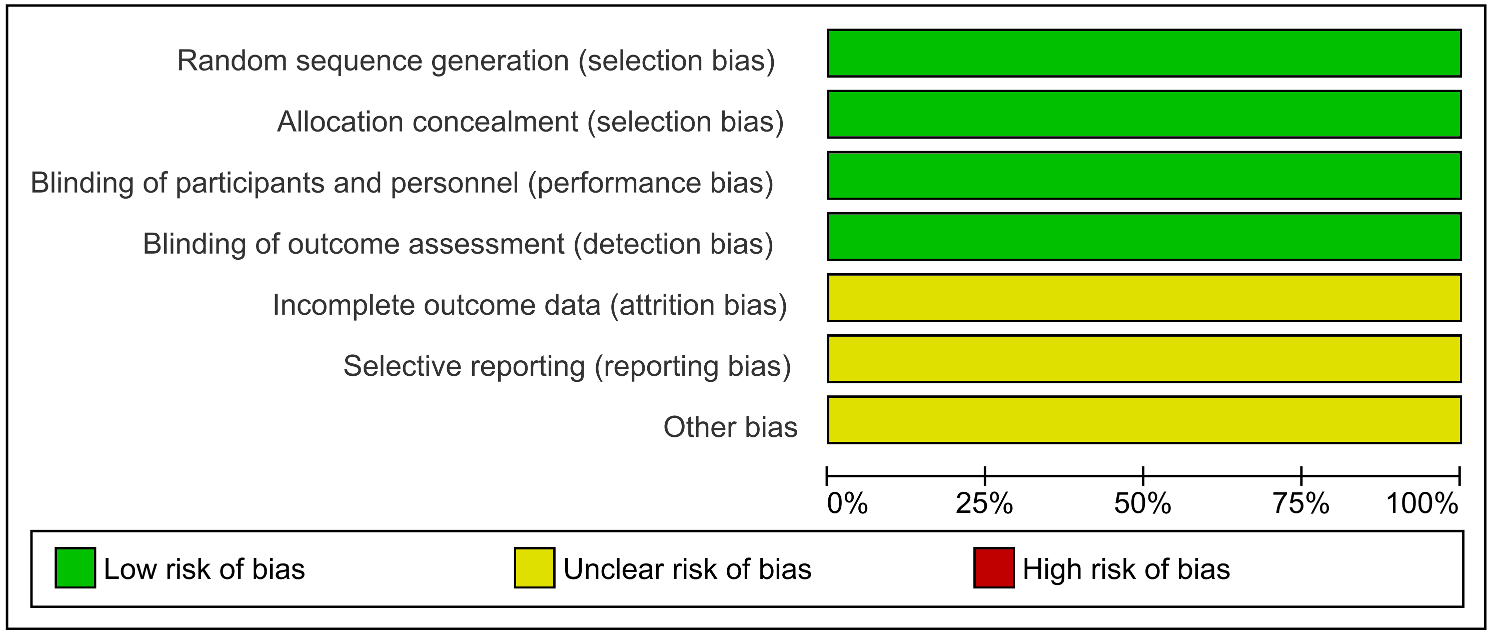

Severe complication

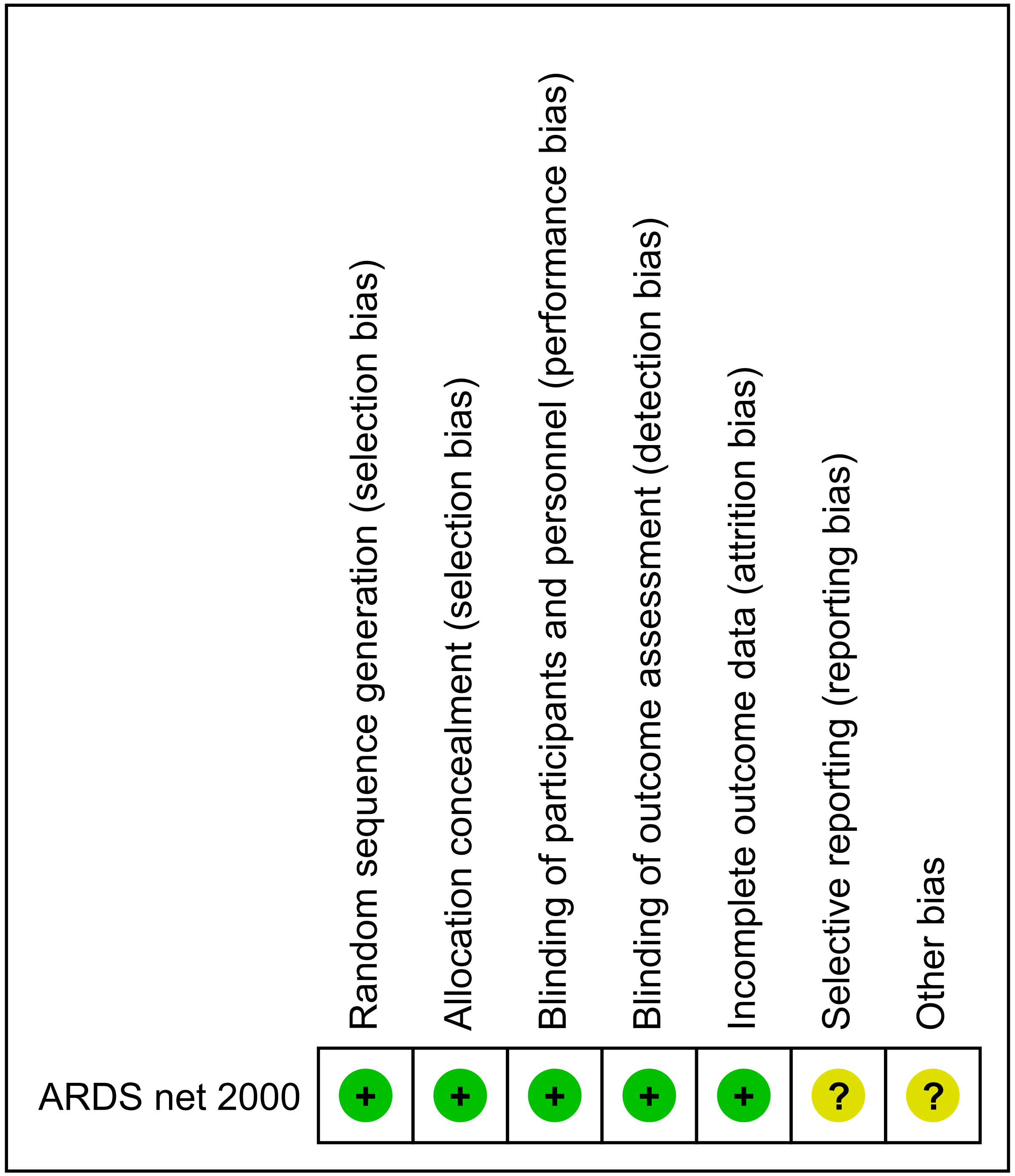

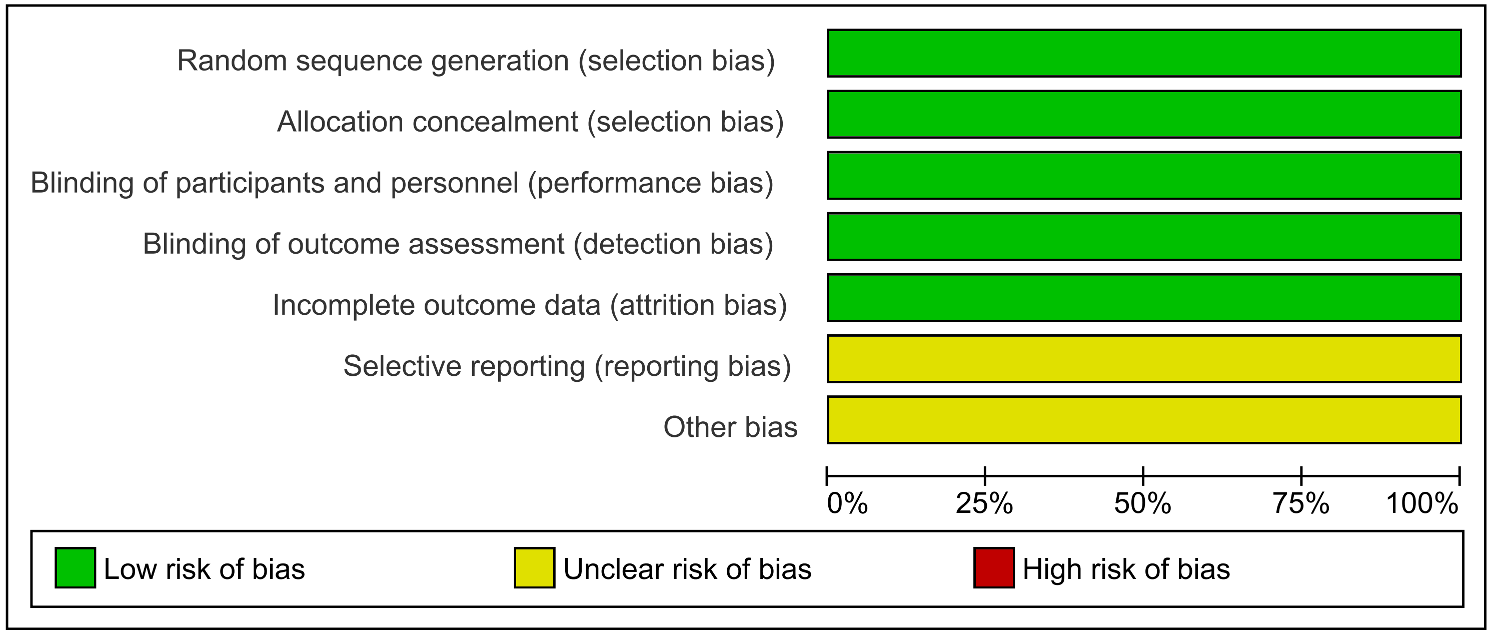

Short term mortality

Study or Subgroup	Ketoconazole Events	Ketoconazole Total	Placebo Events	Placebo Total	Weight	Risk Ratio M-H, Random, 95% CI
ARDS net 2000	41	117	40	117	100.0%	1.02 [0.72, 1.46]
**Total (95% CI)**		**117**		**117**	**100.0%**	**1.02 [0.72, 1.46]**
Total events	41		40			

Heterogeneity: Not applicable
Test for overall effect: Z = 0.14 (P = 0.89)

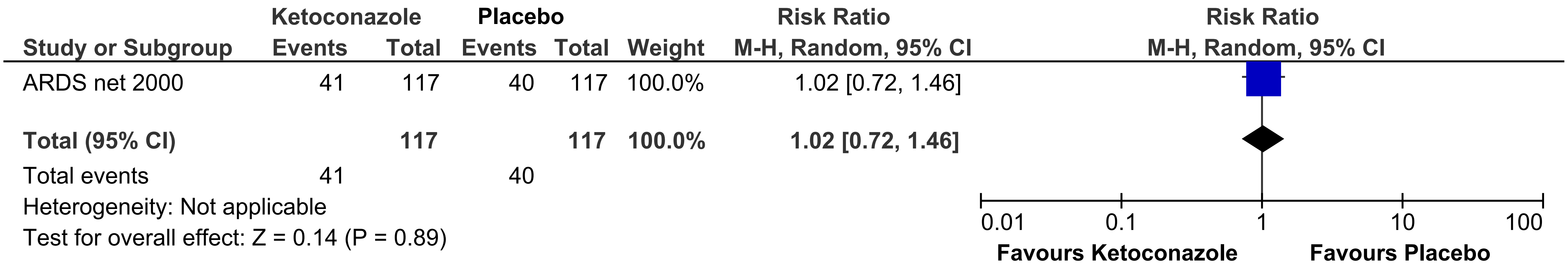

Severe complication

Study or Subgroup	Ketoconazole Events	Ketoconazole Total	Placebo Events	Placebo Total	Weight	Risk Ratio M-H, Random, 95% CI
ARDS net 2000	25	117	20	117	100.0%	1.25 [0.74, 2.12]
**Total (95% CI)**		**117**		**117**	**100.0%**	**1.25 [0.74, 2.12]**
Total events	25		20			

Heterogeneity: Not applicable
Test for overall effect: Z = 0.83 (P = 0.41)

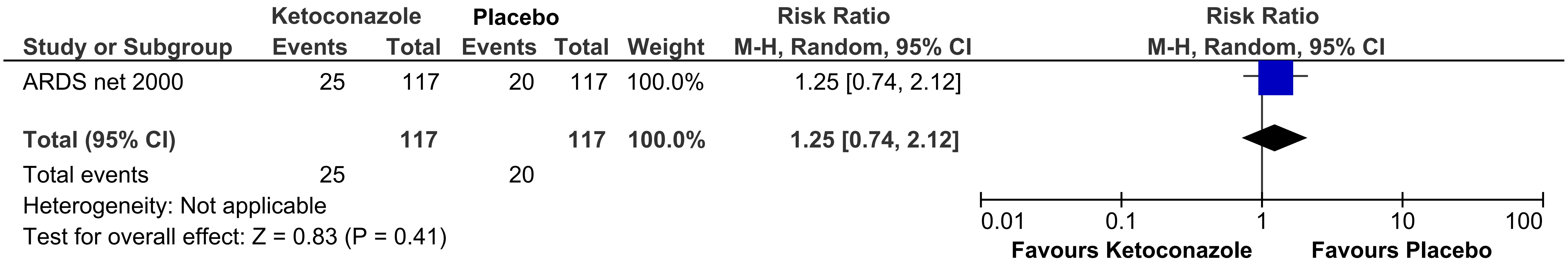

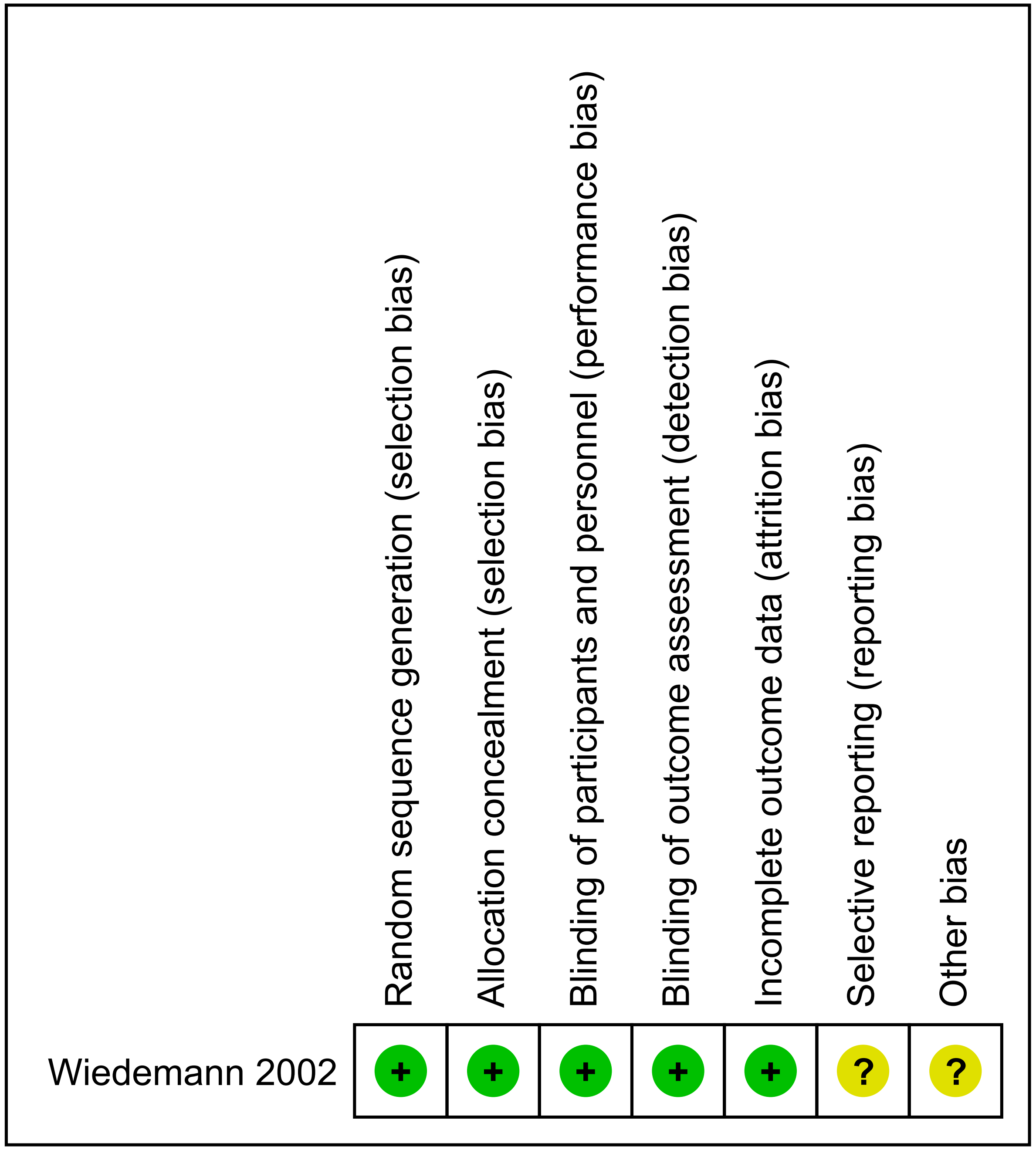

Short term mortality

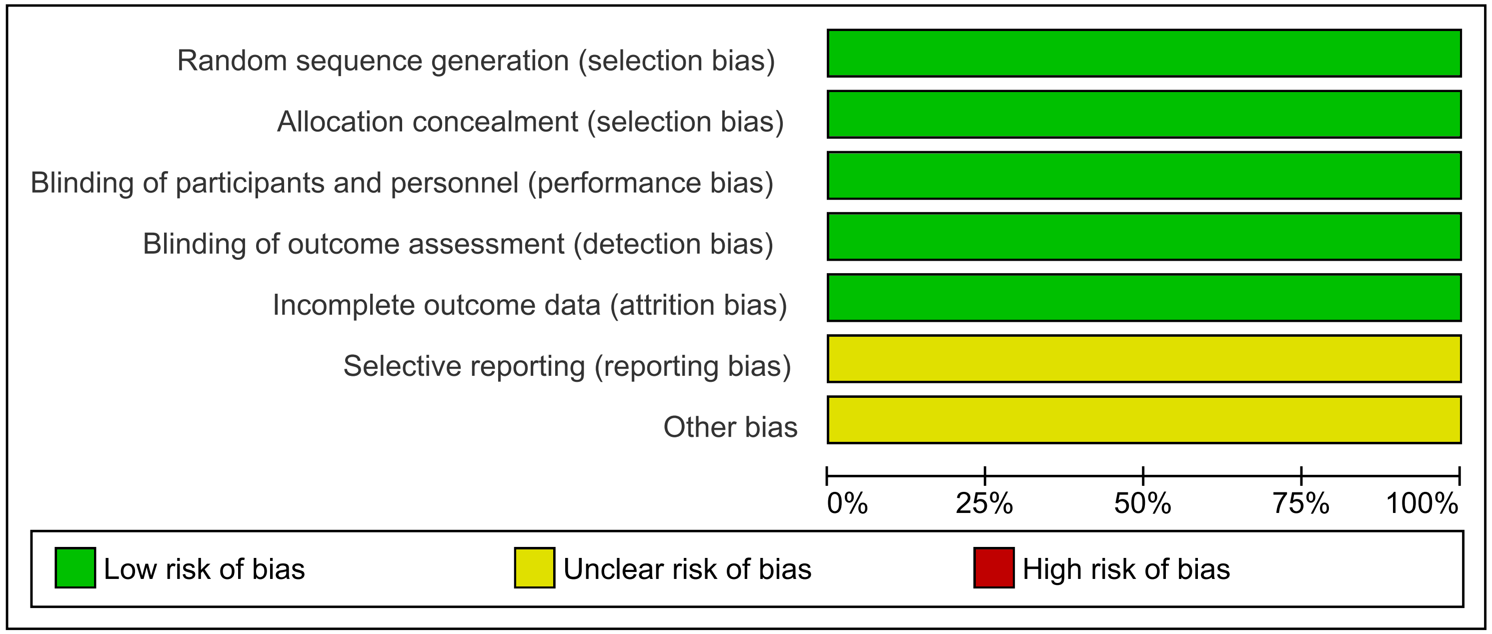

Short term mortality

Study or Subgroup	Lisofylline Events	Lisofylline Total	Placebo Events	Placebo Total	Weight	Risk Ratio M-H, Random, 95% CI
Wiedemann 2002	37	116	29	119	100.0%	1.31 [0.87, 1.98]
**Total (95% CI)**		**116**		**119**	**100.0%**	**1.31 [0.87, 1.98]**
Total events	37		29			

Heterogeneity: Not applicable
Test for overall effect: Z = 1.28 (P = 0.20)

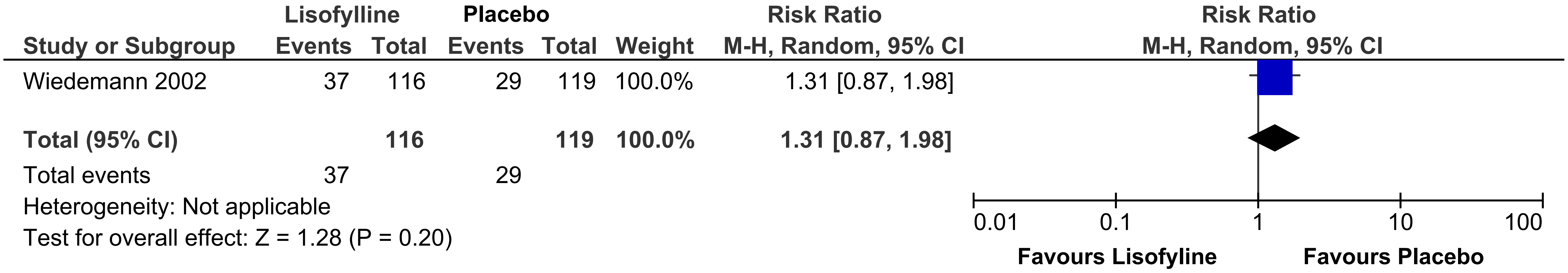

CQ13-01 Summary of findings:

Inhaled nitric oxide compared to placebo for adult ARDS

Patient or population: ARDS
Intervention: Inhaled nitric oxide (NO)
Comparison: placebo

Outcomes	Anticipated absolute effects* (95% CI)		Relative effect (95% CI)	№ of participants (studies)	Quality of the evidence (GRADE)
	Risk with placebo	Risk with Inhaled NO			
Short-term (<90d) mortality	**Study population**		**RR 1.18** (0.91 to 1.52)	699 (7 RCTs)	⊕⊕◯◯ LOW [1,2]
	225 per 1000	**266 per 1000** (205 to 343)			
	**Low**				
	190 per 1000	**224 per 1000** (173 to 289)			
	**High**				
	450 per 1000	**531 per 1000** (410 to 684)			
Severe adverse effects	**Study population**		**RR 1.90** (0.78 to 4.66)	562 (2 RCTs)	⊕⊕⊕◯ MODERATE [2]
	28 per 1000	**53 per 1000** (22 to 130)			
	**Low**				
	20 per 1000	**38 per 1000** (16 to 93)			
	**High**				
	240 per 1000	**456 per 1000** (187 to 1000)			

***The risk in the intervention group** (and its 95% confidence interval) is based on the assumed risk in the comparison group and the **relative effect** of the intervention (and its 95% CI).

CI: Confidence interval; **RR:** Risk ratio

GRADE Working Group grades of evidence
High quality: We are very confident that the true effect lies close to that of the estimate of the effect
Moderate quality: We are moderately confident in the effect estimate: The true effect is likely to be close to the estimate of the effect, but there is a possibility that it is substantially different
Low quality: Our confidence in the effect estimate is limited: The true effect may be substantially different from the estimate of the effect
Very low quality: We have very little confidence in the effect estimate: The true effect is likely to be substantially different from the estimate of effect

1. A lot of high risk of bias in the blinding procedure
2. Wide range of 95%CI due to a limited number of patients

CQ13-02 Summary of findings:

Inhaled beta2 stimulant compared to placebo for adult ARDS

Patient or population: ARDS
Intervention: Inhaled beta2 stimulant
Comparison: placebo

Outcomes	Anticipated absolute effects* (95% CI)		Relative effect (95% CI)	№ of participants (studies)	Quality of the evidence (GRADE)	Comments
	Risk with placebo	Risk with Inhaled beta2 stimulant				
Short-term (<90d) mortality	**Study population**		**RR 1.32** (0.83 to 2.08)	282 (1 RCT)	⨁⨁⨁◯ MODERATE [1]	
	185 per 1000	**244 per 1000** (153 to 384)				
	**Low**					
	190 per 1000	**251 per 1000** (158 to 395)				
	**High**					
	450 per 1000	**594 per 1000** (374 to 936)				
Severe adverse effects	**Study population**		**RR 2.57** (0.85 to 7.76)	282 (1 RCT)	⨁⨁⨁◯ MODERATE [1]	
	31 per 1000	**79 per 1000** (26 to 239)				
	**Low**					
	20 per 1000	**51 per 1000** (17 to 155)				
	**High**					
	240 per 1000	**617 per 1000** (204 to 1000)				

***The risk in the intervention group** (and its 95% confidence interval) is based on the assumed risk in the comparison group and the **relative effect** of the intervention (and its 95% CI).

CI: Confidence interval; **RR:** Risk ratio

GRADE Working Group grades of evidence
High quality: We are very confident that the true effect lies close to that of the estimate of the effect
Moderate quality: We are moderately confident in the effect estimate: The true effect is likely to be close to the estimate of the effect, but there is a possibility that it is substantially different
Low quality: Our confidence in the effect estimate is limited: The true effect may be substantially different from the estimate of the effect
Very low quality: We have very little confidence in the effect estimate: The true effect is likely to be substantially different from the estimate of effect

1. Wide range of 95%CI due to a limited number of patients

CQ13-03 Summary of findings:

Intravenous beta2 stimulant compared to placebo for adult ARDS

Patient or population: ARDS
Intervention: Intravenous beta2 stimulant
Comparison: placebo

Outcomes	Anticipated absolute effects* (95% CI)		Relative effect (95% CI)	№ of participants (studies)	Quality of the evidence (GRADE)	Comments
	Risk with placebo	Risk with Intravenous beta2 stimulant				
Short-term (<90d) mortality	**Study population**		**RR 1.16** (0.68 to 1.96)	364 (2 RCTs)	⊕⊕◯◯ LOW [1,2]	
	283 per 1000	**328 per 1000** (192 to 554)				
	**Low**					
	190 per 1000	**220 per 1000** (129 to 372)				
	**High**					
	450 per 1000	**522 per 1000** (306 to 882)				
Severe adverse effects	**Study population**		**RR 5.78** (1.34 to 24.92)	365 (2 RCTs)	⊕⊕⊕◯ MODERATE [3]	
	22 per 1000	**126 per 1000** (29 to 542)				
	**Low**					
	20 per 1000	**116 per 1000** (27 to 498)				
	**High**					
	240 per 1000	**1000 per 1000** (322 to 1000)				

***The risk in the intervention group** (and its 95% confidence interval) is based on the assumed risk in the comparison group and the **relative effect** of the intervention (and its 95% CI).

CI: Confidence interval; **RR:** Risk ratio

GRADE Working Group grades of evidence
High quality: We are very confident that the true effect lies close to that of the estimate of the effect
Moderate quality: We are moderately confident in the effect estimate: The true effect is likely to be close to the estimate of the effect, but there is a possibility that it is substantially different
Low quality: Our confidence in the effect estimate is limited: The true effect may be substantially different from the estimate of the effect
Very low quality: We have very little confidence in the effect estimate: The true effect is likely to be substantially different from the estimate of effect

1. High value of I^2
2. Wide range of 95%CI due to a limited number of patients
3. The number of patients was less than optimal for the information size.

CQ13-04 Summary of findings:

Granulocyte-macrophage colony-stimulating factor (GM-CSF) compared to placebo for adult ARDS

Patient or population: ARDS
Intervention: Granulocyte-macrophage colony-stimulating factor (GM-CSF)
Comparison: placebo

Outcomes	Anticipated absolute effects* (95% CI)		Relative effect (95% CI)	№ of participants (studies)	Quality of the evidence (GRADE)	Comments
	Risk with placebo	Risk with Granulocyte-macrophage colony-stimulating factor (GM-CSF)				
Short-term (<90d) mortality	Study population		**RR 0.76** (0.40 to 1.46)	148 (2 RCTs)	⊕⊕⊕◯ MODERATE [1]	
	230 per 1000	**175 per 1000** (92 to 335)				
	Low					
	190 per 1000	**144 per 1000** (76 to 277)				
	High					
	450 per 1000	**342 per 1000** (180 to 657)				
Severe adverse effects	Study population		**RR 0.87** (0.42 to 1.80)	130 (1 RCT)	⊕⊕⊕◯ MODERATE [1]	
	197 per 1000	**171 per 1000** (83 to 355)				
	Low					
	20 per 1000	**17 per 1000** (8 to 36)				
	High					
	240 per 1000	**209 per 1000** (101 to 432)				

***The risk in the intervention group** (and its 95% confidence interval) is based on the assumed risk in the comparison group and the **relative effect** of the intervention (and its 95% CI).

CI: Confidence interval; **RR:** Risk ratio

GRADE Working Group grades of evidence
High quality: We are very confident that the true effect lies close to that of the estimate of the effect
Moderate quality: We are moderately confident in the effect estimate: The true effect is likely to be close to the estimate of the effect, but there is a possibility that it is substantially different
Low quality: Our confidence in the effect estimate is limited: The true effect may be substantially different from the estimate of the effect
Very low quality: We have very little confidence in the effect estimate: The true effect is likely to be substantially different from the estimate of effect

1. Wide range of 95%CI due to a limited number of patients

CQ13-05 Summary of findings:

Prostaglandin E_1 compared to placebo for adult ARDS

Patient or population: ARDS
Intervention: Prostaglandin E_1
Comparison: placebo

Outcomes	Anticipated absolute effects* (95% CI)		Relative effect (95% CI)	№ of participants (studies)	Quality of the evidence (GRADE)	Comments
	Risk with placebo	Risk with Prostaglandin E_1				
Short-term (<90d) mortality	Study population		RR 1.07 (0.90 to 1.27)	786 (7 RCTs)	⨁⨁⨁◯ MODERATE [1]	
	380 per 1000	**407 per 1000** (342 to 483)				
	Low					
	190 per 1000	**203 per 1000** (171 to 241)				
	High					
	450 per 1000	**482 per 1000** (405 to 572)				
Severe adverse effects	Study population		RR 2.07 (1.12 to 3.83)	498 (4 RCTs)	⨁⨁⨁◯ MODERATE [1]	
	161 per 1000	**334 per 1000** (180 to 617)				
	Low					
	20 per 1000	**41 per 1000** (22 to 77)				
	High					
	240 per 1000	**497 per 1000** (269 to 919)				

***The risk in the intervention group** (and its 95% confidence interval) is based on the assumed risk in the comparison group and the **relative effect** of the intervention (and its 95% CI).

CI: Confidence interval; **RR:** Risk ratio

GRADE Working Group grades of evidence
High quality: We are very confident that the true effect lies close to that of the estimate of the effect
Moderate quality: We are moderately confident in the effect estimate: The true effect is likely to be close to the estimate of the effect, but there is a possibility that it is substantially different
Low quality: Our confidence in the effect estimate is limited: The true effect may be substantially different from the estimate of the effect
Very low quality: We have very little confidence in the effect estimate: The true effect is likely to be substantially different from the estimate of effect

1. Wide range of 95%CI due to a limited number of patients

CQ13-06 Summary of findings:

Statin compared to placebo for adult ARDS

Patient or population: ARDS
Intervention: Statin
Comparison: placebo

Outcomes	Anticipated absolute effects* (95% CI)		Relative effect (95% CI)	№ of participants (studies)	Quality of the evidence (GRADE)	Comments
	Risk with placebo	Risk with Statin				
Short-term (<90d) mortality	Study population		RR 0.98 (0.71 to 1.36)	1284 (2 RCTs)	⊕⊕◯◯ LOW [1,2]	
	257 per 1000	**252 per 1000** (182 to 349)				
	Low					
	190 per 1000	**186 per 1000** (135 to 258)				
	High					
	450 per 1000	**441 per 1000** (320 to 612)				
Severe adverse effects	Study population		RR 1.36 (0.69 to 2.67)	599 (2 RCTs)	⊕⊕⊕◯ MODERATE [2]	
	74 per 1000	**101 per 1000** (51 to 198)				
	Low					
	20 per 1000	**27 per 1000** (14 to 53)				
	High					
	240 per 1000	**326 per 1000** (166 to 641)				

***The risk in the intervention group** (and its 95% confidence interval) is based on the assumed risk in the comparison group and the **relative effect** of the intervention (and its 95% CI).

CI: Confidence interval; **RR:** Risk ratio

GRADE Working Group grades of evidence
High quality: We are very confident that the true effect lies close to that of the estimate of the effect
Moderate quality: We are moderately confident in the effect estimate: The true effect is likely to be close to the estimate of the effect, but there is a possibility that it is substantially different
Low quality: Our confidence in the effect estimate is limited: The true effect may be substantially different from the estimate of the effect
Very low quality: We have very little confidence in the effect estimate: The true effect is likely to be substantially different from the estimate of effect

1. Increased value of I^2
2. Wide range of 95%CI due to a limited number of patients

CQ13-07 Summary of findings:

Surfactant compared to placebo for adult ARDS

Patient or population: ARDS
Intervention: Surfactant
Comparison: placebo

Outcomes	Anticipated absolute effects* (95% CI)		Relative effect (95% CI)	№ of participants (studies)	Quality of the evidence (GRADE)	Comments
	Risk with placebo	Risk with Surfactant				
Short-term (<90d) mortality	**Study population**		**RR 0.98** (0.88 to 1.09)	2894 (9 RCTs)	⊕⊕◯◯ LOW [1,2]	
	304 per 1000	**298 per 1000** (268 to 332)				
	**Low**					
	190 per 1000	**186 per 1000** (167 to 207)				
	**High**					
	450 per 1000	**441 per 1000** (396 to 491)				
Severe adverse effects	**Study population**		**RR 1.44** (0.99 to 2.09)	2801 (6 RCTs)	⊕◯◯◯ VERY LOW [2,3]	
	195 per 1000	**280 per 1000** (193 to 407)				
	**Low**					
	20 per 1000	**29 per 1000** (20 to 42)				
	**High**					
	240 per 1000	**346 per 1000** (238 to 502)				

***The risk in the intervention group** (and its 95% confidence interval) is based on the assumed risk in the comparison group and the **relative effect** of the intervention (and its 95% CI).

CI: Confidence interval; **RR:** Risk ratio; **MD:** Mean difference

GRADE Working Group grades of evidence
High quality: We are very confident that the true effect lies close to that of the estimate of the effect
Moderate quality: We are moderately confident in the effect estimate: The true effect is likely to be close to the estimate of the effect, but there is a possibility that it is substantially different
Low quality: Our confidence in the effect estimate is limited: The true effect may be substantially different from the estimate of the effect
Very low quality: We have very little confidence in the effect estimate: The true effect is likely to be substantially different from the estimate of effect

1. A lot of high risk of bias in the blinding procedure
2. Wide range of 95%CI due to a limited number of patients
3. Increased value of I^2

CQ13-08 Summary of findings:

Activated protein C compared to placebo for adult ARDS

Patient or population: ARDS
Intervention: Activated protein C
Comparison: placebo

Outcomes	Anticipated absolute effects* (95% CI)		Relative effect (95% CI)	№ of participants (studies)	Quality of the evidence (GRADE)	Comments
	Risk with placebo	Risk with Activated protein C				
Short-term (<90d) mortality	Study population		**RR 0.64** (0.21to1.95)	146 (2 RCTs)	⊕⊕⊕◯ MODERATE [1]	
	158 per 1000	**101 per 1000** (33 to 308)				
	Low					
	190 per 1000	**122 per 1000** (40 to 371)				
	High					
	450 per 1000	**288 per 1000** (95 to 878)				
Severe adverse effects	Study population		**RR 0.78** (0.43to1.40)	146 (2 RCTs)	⊕⊕◯◯ LOW [1,2]	
	263 per 1000	**205 per 1000** (113 to 368)				
	Low					
	20 per 1000	**16 per 1000** (9 to 28)				
	High					
	240 per 1000	**187 per 1000** (103 to 336)				

***The risk in the intervention group** (and its 95% confidence interval) is based on the assumed risk in the comparison group and the **relative effect** of the intervention (and its 95% CI).

CI: Confidence interval; **RR:** Risk ratio

GRADE Working Group grades of evidence
High quality: We are very confident that the true effect lies close to that of the estimate of the effect
Moderate quality: We are moderately confident in the effect estimate: The true effect is likely to be close to the estimate of the effect, but there is a possibility that it is substantially different
Low quality: Our confidence in the effect estimate is limited: The true effect may be substantially different from the estimate of the effect
Very low quality: We have very little confidence in the effect estimate: The true effect is likely to be substantially different from the estimate of effect

1. Wide range of 95%CI due to a limited number of patients
2. A lot of high risk of bias in the selection and blinding procedure

CQ13-09 Summary of findings:

N-acetylcysteine compared to placebo for adult ARDS

Patient or population: ARDS
Intervention: N-acetylcysteine
Comparison: placebo

Outcomes	Anticipated absolute effects* (95% CI)		Relative effect (95% CI)	№ of participants (studies)	Quality of the evidence (GRADE)	Comments
	Risk with placebo	Risk with N-acetylcysteine				
Short-term (<90d) mortality	Study population		**RR 0.89** (0.63 to 1.25)	180 (4 RCTs)	⨁⨁⨁◯ MODERATE [1]	
	444 per 1000	**396 per 1000** (280 to 556)				
	Low					
	190 per 1000	**169 per 1000** (120 to 238)				
	High					
	450 per 1000	**401 per 1000** (284 to 563)				
Severe adverse effects	Study population		---	---	---	
	---	---				

***The risk in the intervention group** (and its 95% confidence interval) is based on the assumed risk in the comparison group and the **relative effect** of the intervention (and its 95% CI).

CI: Confidence interval; **RR:** Risk ratioe

GRADE Working Group grades of evidence
High quality: We are very confident that the true effect lies close to that of the estimate of the effect
Moderate quality: We are moderately confident in the effect estimate: The true effect is likely to be close to the estimate of the effect, but there is a possibility that it is substantially different
Low quality: Our confidence in the effect estimate is limited: The true effect may be substantially different from the estimate of the effect
Very low quality: We have very little confidence in the effect estimate: The true effect is likely to be substantially different from the estimate of effect

1. Wide range of 95%CI due to a limited number of patients

CQ13-10 Summary of findings:

Ketoconazole compared to placebo for adult ARDS

Patient or population: ARDS
Intervention: Ketoconazole
Comparison: placebo

Outcomes	Anticipated absolute effects* (95% CI)		Relative effect (95% CI)	№ of participants (studies)	Quality of the evidence (GRADE)	Comments
	Risk with placebo	Risk with Ketoconazole				
Short-term (<90d) mortality	Study population		**RR 1.02** (0.72 to 1.46)	234 (1 RCT)	⨁⨁⨁◯ MODERATE [1]	
	342 per 1000	**349 per 1000** (246 to 499)				
	Low					
	190 per 1000	**194 per 1000** (137 to 277)				
	High					
	450 per 1000	**459 per 1000** (324 to 657)				
Severe adverse effects	Study population		**RR 1.25** (0.74 to 2.12)	234 (1 RCT)	⨁⨁⨁◯ MODERATE [1]	
	171 per 1000	**214 per 1000** (126 to 362)				
	Low					
	20 per 1000	**25 per 1000** (15 to 42)				
	High					
	240 per 1000	**300 per 1000** (178 to 509)				

***The risk in the intervention group** (and its 95% confidence interval) is based on the assumed risk in the comparison group and the **relative effect** of the intervention (and its 95% CI).

CI: Confidence interval; **RR:** Risk ratio

GRADE Working Group grades of evidence
High quality: We are very confident that the true effect lies close to that of the estimate of the effect
Moderate quality: We are moderately confident in the effect estimate: The true effect is likely to be close to the estimate of the effect, but there is a possibility that it is substantially different
Low quality: Our confidence in the effect estimate is limited: The true effect may be substantially different from the estimate of the effect
Very low quality: We have very little confidence in the effect estimate: The true effect is likely to be substantially different from the estimate of effect

1. Wide range of 95%CI due to a limited number of patients

CQ13-11 Summary of findings:

Lisofylline compared to placebo for adult ARDS

Patient or population: ARDS
Intervention: Lisofylline
Comparison: placebo

Outcomes	Anticipated absolute effects* (95% CI)		Relative effect (95% CI)	№ of participants (studies)	Quality of the evidence (GRADE)	Comments
	Risk with placebo	Risk with Lisofylline				
Short-term (<90d) mortality	**Study population**		RR 1.31 (0.87 to 1.98)	235 (1 RCT)	⊕⊕⊕◯ MODERATE [1]	
	244 per 1000	**319 per 1000** (212 to 483)				
	**Low**					
	190 per 1000	**249 per 1000** (165 to 376)				
	**High**					
	450 per 1000	**590 per 1000** (392 to 891)				
Severe adverse effects	**Study population**		---	---	---	
	---	---				

***The risk in the intervention group** (and its 95% confidence interval) is based on the assumed risk in the comparison group and the **relative effect** of the intervention (and its 95% CI).

CI: Confidence interval; **RR:** Risk ratio

GRADE Working Group grades of evidence
High quality: We are very confident that the true effect lies close to that of the estimate of the effect
Moderate quality: We are moderately confident in the effect estimate: The true effect is likely to be close to the estimate of the effect, but there is a possibility that it is substantially different
Low quality: Our confidence in the effect estimate is limited: The true effect may be substantially different from the estimate of the effect
Very low quality: We have very little confidence in the effect estimate: The true effect is likely to be substantially different from the estimate of effect

1. Wide range of 95%CI due to a limited number of patients

CQ13:

Question: Should the following drugs be used to treat adult patients with ARDS?

(inhaled nitric oxide (NO), inhaled / intravenous β_2 stimulant, granulocyte macrophage colony-stimulating factor (GM-CSF), prostaglandin E_1 (PGE_1), statin, surfactant, activated protein C (APC), N-acetylcysteine (NAC), and ketoconazole or lisofylline)

CQ13-01 Inhaled nitric oxide compared with placebo for adult patients with ARDS

Quality assessment							№ of patients		Effect		Quality	Importance
№ of studies	Study design	Risk of bias	Inconsistency	Indirectness	Imprecision	Other considerations	Inhaled nitric oxide for ARDS	placebo	Relative (95% CI)	Absolute (95% CI)		
Short-term (<90d) mortality												
7	Randomized trials	Serious [1]	Not serious	Not serious	Serious [2]	None	99 per 384 (25.8%)	71 per 315 (22.5%)	RR 1.18 (0.91 to 1.52)	41 more per 1000 (from 20 fewer to 117 more)	⨁⨁⊖⊖ LOW	CRITICAL
								19.0%		34 more per 1000 (from 17 fewer to 99 more)		
								45.0%		81 more per 1000 (from 40 fewer to 234 more)		
Severe adverse events												
2	Randomized trials	Not serious	Not serious	Not serious	Serious [2]	None	17 per 312 (5.4%)	7 per 250 (2.8%)	RR 1.90 (0.78 to 4.66)	25 more per 1000 (from 6 fewer to 102 more)	⨁⨁⨁⊖ MODERATE	CRITICAL
								2.0%		18 more per 1000 (from 4 fewer to 73 more)		
								24.0%		216 more per 1000 (from 53 fewer to 878 more)		

CI: Confidence interval; RR: Risk ratio

1. There is a high risk of bias in the blinding procedure
2. Wide range of 95%CI due to a limited number of patients

CQ13-02 Inhaled $beta_2$ stimulant compared with placebo for adult patients with ARDS

Quality assessment							№ of patients		Effect		Quality	Importance
№ of studies	Study design	Risk of bias	Inconsistency	Indirectness	Imprecision	Other considerations	Inhaled $beta_2$ stimulant for ARDS	placebo	Relative (95% CI)	Absolute (95% CI)		
Short-term (<90d) mortality												
1	Randomized trials	Not serious	Not serious	Not serious	Serious [1]	None	37 per 152 (24.3%)	24 per 130 (18.5%)	RR 1.32 (0.83 to 2.08)	59 more per 1000 (from 31 fewer to 199 more)	⊕⊕⊕⊖ MODERATE [1]	CRITICAL
								19.0%		61 more per 1000 (from 32 fewer to 205 more)		
								45.0%		144 more per 1000 (from 77 fewer to 486 more)		
Severe adverse events												
1	Randomized trials	Not serious	Not serious	Not serious	Serious [1]	None	12 per 152 (7.9%)	4 per 130 (3.1%)	RR 2.57 (0.85 to 7.76)	48 more per 1000 (from 5 fewer to 208 more)	⊕⊕⊕⊖ MODERATE [1]	CRITICAL
								2.0%		31 more per 1000 (from 3 fewer to 135 more)		
								24.0%		377 more per 1000 (from 36 fewer to 1000 more)		

CI: Confidence interval; RR: Risk ratio

1. Wide range of 95%CI due to a limited number of patients

CQ13-03 Intravenous $beta_2$ stimulant compared with placebo for adult patients with ARDS

Quality assessment							№ of patients		Effect		Quality	Importance
№ of studies	Study design	Risk of bias	Inconsistency	Indirectness	Imprecision	Other considerations	Intravenous $beta_2$ stimulant for ARDS	placebo	Relative (95% CI)	Absolute (95% CI)		
Short-term (<90d) mortality												
2	Randomized trials	Not serious	Serious [1]	Not serious	Serious [2]	None	66 per 180 (36.7%)	52 per 184 (28.3%)	RR 1.16 (0.68 to 1.96)	45 more per 1000 (from 90 fewer to 271 more)	⊕⊕⊕⊝ LOW	CRITICAL
								19.0%		30 more per 1000 (from 61 fewer to 182 more)		
								45.0%		72 more per 1000 (from 144 fewer to 432 more)		
Severe adverse events												
2	Randomized trials	Not serious	Not serious	Not serious	Serious [3]	None	28 per 181 (15.5%)	4 per 184 (2.2%)	RR 5.78 (1.34 to 24.92)	104 more per 1000 (from 7 more to 520 more)	⊕⊕⊕⊝ MODERATE	CRITICAL
								2.0%		96 more per 1000 (from 7 more to 478 more)		
								24.0%		1000 more per 1000 (from 82 more to 1000 more)		

CI: Confidence interval; RR: Risk ratio

1. High value of I^2
2. Wide range of 95%CI due to a limited number of patients
3. The number of patients was smaller than optimal for the information size.

CQ13-04 Granulocyte-macrophage colony-stimulating factor (GM-CSF) compared with placebo for adult patients with ARDS

Quality assessment							№ of patients		Effect		Quality	Importance
№ of studies	Study design	Risk of bias	Inconsistency	Indirectness	Imprecision	Other considerations	GM-CSF for ARDS	placebo	Relative (95% CI)	Absolute (95% CI)		
Short-term (<90d) mortality												
2	Randomized trials	Not serious	Not serious	Not serious	Serious [1]	None	13 per 74 (17.6%)	17 per 74 (23.0%)	RR 0.76 (0.40 to 1.46)	55 fewer per 1000 (from 106 more to 138 fewer)	⊕⊕⊕⊖ MODERATE [1]	CRITICAL
								19.0%		46 fewer per 1000 (from 87 more to 114 fewer)		
								45.0%		108 fewer per 1000 (from 207 more to 270 fewer)		
Severe adverse events												
1	Randomized trials	Not serious	Not serious	Not serious	Serious [1]	None	11 per 64 (17.2%)	13 per 66 (19.7%)	RR 0.87 (0.42 to 1.80)	26 fewer per 1000 (from 114 fewer to 158 more)	⊕⊕⊕⊖ MODERATE [1]	CRITICAL
								2.0%		3 fewer per 1000 (from 12 fewer to 16 more)		
								24.0%		31 fewer per 1000 (from 139 fewer to 192 more)		

CI: Confidence interval; RR: Risk ratio

1. Wide range of 95%CI due to a limited number of patients

CQ13-05 Prostaglandin E_1 compared with placebo for adult patients with ARDS

Quality assessment							№ of patients		Effect		Quality	Importance
№ of studies	Study design	Risk of bias	Inconsistency	Indirectness	Imprecision	Other considerations	Prostaglandin E_1 for ARDS	placebo	Relative (95% CI)	Absolute (95% CI)		
Short-term (<90d) mortality												
7	Randomized trials	Not serious	Not serious	Not serious	Serious [1]	None	162 per 418 (38.8%)	140 per 368 (38.0%)	RR 1.07 (0.90 to 1.27)	**27 more per 1000** (from 38 fewer to 103 more)	⊕⊕⊕⊖ MODERATE	CRITICAL
								19.0%		**13 more per 1000** (from 19 fewer to 51 more)		
								45.0%		**32 more per 1000** (from 45 fewer to 122 more)		
Severe adverse events												
4	Randomized trials	Not serious	Not serious	Not serious	Serious [1]	None	112 per 256 (43.8%)	39 per 242 (16.1%)	RR 2.07 (1.12 to 3.83)	**172 more per 1000** (from 19 more to 456 more)	⊕⊕⊕⊖ MODERATE	CRITICAL
								2.0%		**21 more per 1000** (from 2 more to 57 more)		
								24.0%		**257 more per 1000** (from 29 more to 679 more)		

CI: Confidence interval; RR: Risk ratio

1. Wide range of 95%CI due to a limited number of patients

CQ13-06 Statin compared with placebo for adult patients with ARDS

Quality assessment							№ of patients		Effect		Quality	Importance
№ of studies	Study design	Risk of bias	Inconsistency	Indirectness	Imprecision	Other considerations	Statin for ARDS	placebo	Relative (95% CI)	Absolute (95% CI)		
Short-term (<90d) mortality												
2	Randomized trials	Not serious	Serious [1]	Not serious	Serious [2]	None	165 per 638 (25.9%)	166 per 646 (25.7%)	RR 0.98 (0.71 to 1.36)	5 fewer per 1000 (from 75 fewer to 93 more)	⊕⊕⊖⊖ LOW [1,2]	CRITICAL
								19.0%		4 fewer per 1000 (from 55 fewer to 68 more)		
								45.0%		9 fewer per 1000 (from 131 fewer to 162 more)		
Severe adverse events												
2	Randomized trials	Not serious	Not serious	Not serious	Serious [2]	None	32 per 289 (11.1%)	23 per 310 (7.4%)	RR 1.36 (0.69 to 2.67)	27 more per 1000 (from 23 fewer to 124 more)	⊕⊕⊕⊖ MODERATE [2]	CRITICAL
								2.0%		7 more per 1000 (from 6 fewer to 33 more)		
								24.0%		86 more per 1000 (from 74 fewer to 401 more)		

CI: Confidence interval; RR: Risk ratio

1. Large I^2 value
2. Wide range of 95%CI due to a limited number of patients

CQ13-07 Surfactant compared with placebo for adult patients with ARDS

Quality assessment							№ of patients		Effect		Quality	Importance
№ of studies	Study design	Risk of bias	Inconsistency	Indirectness	Imprecision	Other considerations	Surfactant for ARDS	placebo	Relative (95% CI)	Absolute (95% CI)		
Short-term (<90d) mortality												
9	Randomized trials	Serious [1]	Not serious	Not serious	Serious [2]	None	436 per 1455 (30.0%)	438 per 1439 (30.4%)	RR 0.98 (0.88 to 1.09)	6 fewer per 1000 (from 27 more to 37 fewer)	⊕⊕⊖⊖ LOW [1 2]	CRITICAL
								19.0%		4 fewer per 1000 (from 17 more to 23 fewer)		
								45.0%		9 fewer per 1000 (from 41 more to 54 fewer)		
Severe adverse events												
6	Randomized trials	Not serious	Very serious [3]	Not serious	Serious [2]	None	333 per 1409 (23.6%)	271 per 1392 (19.5%)	RR 1.44 (0.99 to 2.09)	86 more per 1000 (from 2 fewer to 212 more)	⊕⊖⊖⊖ VERYLOW [2 3]	CRITICAL
								2.0%		9 more per 1000 (from 0 fewer to 22 more)		
								24.0%		106 more per 1000 (from 2 fewer to 262 more)		

CI: Confidence interval; RR: Risk ratio

1. Significant numbers of high risk of bias in the blinding procedure
2. Wide range of 95%CI due to a limited number of patients
3. Large I^2 value

CQ13-08 Activated protein C compared with placebo for adult patients with ARDS

Quality assessment							№ of patients		Effect		Quality	Importance
№ of studies	Study design	Risk of bias	Inconsistency	Indirectness	Imprecision	Other considerations	Activated protein C for ARDS	placebo	Relative (95% CI)	Absolute (95% CI)		
Short-term (<90d) mortality												
2	Randomized trials	Not serious	Not serious	Not serious	Serious [1]	None	7 per 70 (10.0%)	12 per 76 (15.8%)	RR 0.64 (0.21 to 1.95)	57 fewer per 1000 (from 125 fewer to 150 more)	⊕⊕⊕⊖ MODERATE [1]	CRITICAL
								19.0%		68 fewer per 1000 (from 150 fewer to 181 more)		
								45.0%		162 fewer per 1000 (from 356 fewer to 428 more)		
Severe adverse events												
2	Randomized trials	Serious [2]	Not serious	Not serious	Serious [1]	None	14 per 70 (20.0%)	20 per 76 (26.3%)	RR 0.78 (0.43 to 1.40)	58 fewer per 1000 (from 105 more to 150 fewer)	⊕⊕⊖⊖ LOW [1,2]	CRITICAL
								2.0%		4 fewer per 1000 (from 8 more to 11 fewer)		
								24.0%		53 fewer per 1000 (from 96 more to 137 fewer)		

CI: Confidence interval; RR: Risk ratio

1. Wide range of 95%CI due to a limited number of patients
2. There is a high risk of bias in the selection and blinding procedure

CQ13-09 N-acetylcysteine compared with placebo for adult patients with ARDS

Quality assessment							№ of patients		Effect		Quality	Importance
№ of studies	Study design	Risk of bias	Inconsistency	Indirectness	Imprecision	Other considerations	N-acetylcysteine for ARDS	placebo	Relative (95% CI)	Absolute (95% CI)		
Short-term (<90d) mortality												
4	Randomized trials	Not serious	Not serious	Not serious	Serious [1]	None	34 per 90 (37.8%)	40 per 90 (44.4%)	RR 0.89 (0.63 to 1.25)	49 fewer per 1000 (from 111 more to 164 fewer)	⊕⊕⊕⊝ MODERATE [1]	CRITICAL
								19.0%		21 fewer per 1000 (from 48 more to 70 fewer)		
								45.0%		49 fewer per 1000 (from 113 more to 167 fewer)		

CI: Confidence interval; RR: Risk ratio

1. Wide range of 95%CI due to a limited number of patients

CQ13-10 Ketoconazole compared with placebo for adult patients with ARDS

Quality assessment							№ of patients		Effect		Quality	Importance
№ of studies	Study design	Risk of bias	Inconsistency	Indirectness	Imprecision	Other considerations	Ketoconazole for ARDS	placebo	Relative (95% CI)	Absolute (95% CI)		
Short-term (<90d) mortality												
1	Randomized trials	Not serious	Not serious	Not serious	Serious [1]	None	41 per 117 (35.0%)	40 per 117 (34.2%)	RR 1.02 (0.72 to 1.46)	7 more per 1000 (from 96 fewer to 157 more)	⊕⊕⊕⊖ MODERATE [1]	CRITICAL
								19.0%		4 more per 1000 (from 53 fewer to 87 more)		
								45.0%		9 more per 1000 (126 fewer to 207 more)		
Severe adverse events												
1	Randomized trials	Not serious	Not serious	Not serious	Serious [1]	None	25 per 117 (21.4%)	20 per 117 (17.1%)	RR 1.25 (0.74 to 2.12)	43 more per 1000 (from 44 fewer to 191 more)	⊕⊕⊕⊖ MODERATE [1]	CRITICAL
								2.0%		5 more per 1000 (from 5 fewer to 22 more)		
								24.0%		60 more per 1000 (from 62 fewer to 269 more)		

CI: Confidence interval; RR: Risk ratio

1. Wide range of 95%CI due to a limited number of patients

CQ13-11 Lisofylline compared with placebo for adult patients with ARDS

Quality assessment							№ of patients		Effect		Quality	Importance
№ of studies	Study design	Risk of bias	Inconsistency	Indirectness	Imprecision	Other considerations	Lisofylline for ARDS	placebo	Relative (95% CI)	Absolute (95% CI)		
Short-term (<90d) mortality												
1	Randomized trials	Not serious	Not serious	Not serious	Serious [1]	None	37 per 116 (31.9%)	29 per 119 (24.4%)	RR 1.31 (0.87 to 1.98)	76 more per 1000 (from 32 fewer to 239 more)	⊕⊕⊕⊖ MODERATE [1]	CRITICAL
								19.0%		59 more per 1000 (from 25 fewer to 186 more)		
								45.0%		140 more per 1000 (from 59 fewer to 441 more)		

CI: Confidence interval; RR: Risk ratio

1. Wide range of 95%CI due to a limited number of patients

Evidence-to-Decision Table

CQ13 : SHOULD THE FOLLOWING DRUGS BE USED FOR ADULT PATIENTS WITH ARDS?

(inhaled nitric oxide (NO), inhaled/ intravenous $beta_2$ stimulant, granulocyte macrophage colony-stimulating factor (GM-CSF), prostaglandin E_1 (PGE_1), statin, surfactant, activated protein C (APC), N-acetylcysteine (NAC), ketoconazole, and lisofylline)

PATIENTS: ADULT PATIENTS WITH ARDS

INTERVENTION: DRUG

(inhaled nitric oxide (NO), inhaled/ intravenous $beta_2$ stimulant, granulocyte macrophage colony-stimulating factor (GM-CSF), prostaglandin E_1 (PGE_1), statin, surfactant, activated protein C (APC), N-acetylcysteine (NAC), ketoconazole and lisofylline)

	CRITERIA	JUDGEMENTS	RESEARCH EVIDENCE	ADDITIONAL CONSIDERATION
PROBLEM	**Is the problem a priority?**	○No<br>○Probably no<br>●Probably yes<br>○Yes<br>------------------<br>○Varies<br>○Don't know	The pathogenesis of ARDS is non-cardiogenic pulmonary edema with increased permeability caused by nonspecific inflammation in the pulmonary alveolar space (1). A number of factors including alveolar epithelial injury, increased pulmonary vascular resistance due to hypoxic pulmonary vasoconstriction, ventilation-perfusion mismatch and endogenous surfactant dysfunction are associated with the pathogenesis of ARDS. Therefore, a number of drugs have been investigated to treat ARDS including inhaled nitric oxide (NO) (2) as a pulmonary vasodilator, aerosolized/ intravenous β_2 stimulants (3-6) to resolve pulmonary edema, granulocyte-macrophage colony stimulating factor (GM-CSF) (7, 8) promoting growth of alveolar macrophages and alveolar epithelial cells, prostaglandin E_1 (PGE_1) (9, 10) as an anti-inflammatory agent, 3-hydroxy-3-methylglutaryl (HMG-CoA) reductase inhibitors including statin (11, 12), the antifungal drug ketoconazole (13, 14), lisofylline (15, 16), activated protein C (APC) (17, 18) which has anticoagulant and anti-inflammatory properties, and N-acetylcysteine (NAC) with antioxidant effects and exogenous surfactant supplementation to improve endogenous surfactant dysfunction. These agents have variable domestic availability, cost, and safety. If these agents are clinically indicated for the treatment of patients with ARDS, off-label use is mandatory in Japan. Due to the very limited number of effective agents for the treatment of ARDS, the priority of this problem is high.	
DESIRABLE AND UNDESIRABLE EVENTS	**What is the overall certainty of the evidence of events?**	●Very low<br>○Low<br>○Moderate<br>○High<br>------------------<br>○No included studies	The relative importance or values of the main outcomes of interest: (see tables below)	
	**Is there important uncertainty about or variability in how much people value the main outcomes?**	○Important uncertainty or variability<br>●Possibly important uncertainty or variability<br>○Possibly no important uncertainty or variability<br>○No important uncertainty or variability<br>------------------<br>○No known undesirable outcomes		
	**How substantial are the desirable anticipated events?**	○Trivial<br>○Small<br>○Moderate<br>○Large<br>------------------<br>●Varies<br>○Don't know		
	**How substantial are the undesirable anticipated events?**	○Large<br>○Moderate<br>○Small<br>○Trivial<br>------------------<br>●Varies<br>○Don't know		

The relative importance or values of the main outcomes of interest:

CQ13-01 inhaled NO

Outcome	Relative importance	Certainty of the evidence (GRADE)
Mortality (short term) (NOTE1	CRITICAL	⊕⊕⊖⊖<br>LOW
Significant adverse events	CRITICAL	⊕⊕⊕⊖<br>MODERATE

CQ13-02 inhaled β_2 stimulant

Outcome	Relative importance	Certainty of the evidence (GRADE)
Mortality (short term) (NOTE1	CRITICAL	⊕⊕⊕⊖<br>MODERATE
Severe adverse events	CRITICAL	⊕⊕⊕⊖<br>MODERATE

CQ13-03 intravenous β_2 stimulant

Outcome	Relative importance	Certainty of the evidence (GRADE)
Mortality (short term) (NOTE1	CRITICAL	⊕⊕⊖⊖<br>LOW
Significant adverse events	CRITICAL	⊕⊕⊕⊖<br>MODERATE

Does the balance between desirable and undesirable effects favor the intervention or the comparison?

○Favors the comparison
○Probably favors the comparison
○Does not favor either the intervention or the comparison
○Probably favors the intervention
○Favors the intervention

●Varies
○Don't know

CQ13-04 granulocyte macrophage colony-stimulating factor (GM-CSF)

Outcome	Relative importance	Certainty of the evidence (GRADE)
Mortality (short term) (NOTE1	CRITICAL	⊕⊕⊕⊝ MODERATE
Significant adverse events	CRITICAL	⊕⊕⊕⊝ MODERATE

CQ13-05 prostaglandin E_1

Outcome	Relative importance	Certainty of the evidence (GRADE)
Mortality (short term) (NOTE1	CRITICAL	⊕⊕⊕⊝ MODERATE
Significant adverse events	CRITICAL	⊕⊕⊕⊝ MODERATE

CQ13-06 statin

Outcome	Relative importance	Certainty of the evidence (GRADE)
Mortality (short term) (NOTE1	CRITICAL	⊕⊕⊝⊝ LOW
Significant adverse events	CRITICAL	⊕⊕⊕⊝ MODERATE

CQ13-07 surfactant

Outcome	Relative importance	Certainty of the evidence (GRADE)
Mortality (short term) (NOTE1	CRITICAL	⊕⊕⊝⊝ LOW
Significant adverse events	CRITICAL	⊕⊝⊝⊝ VERY LOW

CQ13-08 activated protein C

Outcome	Relative importance	Certainty of the evidence (GRADE)
Mortality (short term) (NOTE1	CRITICAL	⊕⊕⊕⊝ MODERATE
Significant adverse events	CRITICAL	⊕⊕⊝⊝ LOW

CQ13-09 N-acetylcystein

Outcome	Relative importance	Certainty of the evidence (GRADE)
Mortality (short term) (NOTE1	CRITICAL	⊕⊕⊕⊝ MODERATE
Significant adverse events	CRITICAL	No studies

CQ13-10 ketoconazole

Outcome	Relative importance	Certainty of the evidence (GRADE)
Mortality (short term) (NOTE1	CRITICAL	⊕⊕⊕⊝ MODERATE
Significant adverse events	CRITICAL	⊕⊕⊕⊝ MODERATE

CQ13-11 lisofylline

Outcome	Relative importance	Certainty of the evidence (GRADE)
Mortality (short term) (NOTE1	CRITICAL	⨁⨁⨁⊝ MODERATE
Significant adverse events	CRITICAL	No studies

Summary of findings:
CQ13-01 inhaled nitric oxide

Outcome	Placebo	Intervention	Absolute effect (95% CI)	Relative risk (95% CI)
Mortality (short term) (NOTE1	225 / 1000	266 / 1000 (205 to 343)	41 more per 1000 (from 20 fewer to 117 more)	**RR 1.18** (0.91 to 1.52)
	190 / 1000	224 / 1000 (173 to 289)	34 more per 1000 (from 17 fewer to 99 more)	
	450 / 1000	531 / 1000 (410 to 684)	81 more / 1000 (from 40 fewer to 234 more)	
Significant adverse events	28 / 1000	53 / 1000 (22 to 130)	25 more per 1000 (from 6 fewer to 102 more)	**RR 1.90** (0.78 to 4.66)
	20 / 1000	38 / 1000 (16 to 93)	18 more per 1000 (from 4 fewer to 73 more)	
	240 / 1000	456 / 1000 (187 to 1000)	216 more per 1000 (from 53 fewer to 878 more)	

Summary : inhaled nitric oxide had no effect on mortality (short) or the rate of significant adverse events. Certainty of the evidence「LOW」

CQ13-02 inhaled β_2 agonist

Outcome	Placebo	Intervention	Absolute effect (95% CI)	Relative risk (95% CI)
Mortality (short term) (NOTE1	185 / 1000	244 / 1000 (153 to 384)	59 more per 1000 (from 31 fewer to 199 more)	**RR 1.32** (0.83 to 2.08)
	190 / 1000	251 / 1000 (158 to 395)	61 more per 1000 (from 32 fewer to 205 more)	
	450 / 1000	594 / 1000 (374 to 936)	144 more per 1000 (from 77 fewer to 486 more)	
Significant adverse events	31 / 1000	79 / 1000 (26 to 239)	48 more per 1000 (from 5 fewer to 208 more)	**RR 2.57** (0.85 to 7.76)
	20 / 1000	51 / 1000 (17 to 155)	31 more per 1000 (from 3 fewer to 135 more)	
	240 / 1000	617 / 1000 (204 to 1000)	377 more per 1000 (from 36 fewer to 1000 more)	

Summary : inhaled β_2 agonist had no effect on mortality (short) or the rate of significant adverse events. Certainty of the evidence「MODERATE」

CQ13-03 intravenous β_2 agonist

Outcome	Placebo	Intervention	Absolute effect (95% CI)	Relative risk (95% CI)
Mortality (short term) (NOTE1	283 / 1000	328 / 1000 (192 to 554)	45 more per 1000 (from 90 fewer to 271 more)	**RR 1.16** (0.68 to 1.96)
	190 / 1000	220 / 1000 (129 to 372)	30 more per 1000 (from 61 fewer to 182 more)	
	450 / 1000	522 / 1000 (306 to 882)	72 more per 1000 (from 144 fewer to 432 more)	
Significant adverse events	22 / 1000	126 / 1000 (29 to 542)	104 more per 1000 (from 7 more to 520 more)	**RR 5.78** (1.34 to 24.92)
	20 / 1000	116 / 1000 (27 to 498)	96 more per 1000 (from 7 more to 478 more)	
	240 / 1000	1000 / 1000 (322 to 1000)	1000 more per 1000 (from 82 more to 1000 more)	

Summary : intravenous β_2 agonist had no effect on mortality (short), but significantly increased the rate of significant adverse events. Certainty of the evidence「MODERATE」

CQ13-04 granulocyte macrophage colony-stimulating factor (GM-CSF)

Outcome	Placebo	Intervention	Absolute effect (95% CI)	Relative risk (95% CI)
Mortality (short term) (NOTE1	230 / 1000	175 / 1000 (92 to 335)	55 fewer per 1000 (from 106 more to 138 fewer)	**RR 0.76** (0.40 to 1.46)
	190 / 1000	144 / 1000 (76 to 277)	46 fewer per 1000 (from 87 more to 114 fewer)	
	450 / 1000	342 / 1000 (180 to 657)	108 fewer per 1000 (from 207 more to 270 fewer)	
Severe adverse events	197 / 1000	171 / 1000 (83 to 355)	26 fewer per 1000 (from 114 fewer to 158 more)	**RR 0.87** (0.42 to 1.80)
	20 / 1000	17 / 1000 (8 to 36)	3 fewer per 1000 (from 12 fewer to 16 more)	
	240 / 1000	209 / 1000 (101 to 432)	31 fewer per 1000 (from 139 fewer to 192 more)	

Summary : GM-CSF had no effect on mortality (short) or the rate of significant adverse events. Certainty of the evidence「MODERATE」

CQ13-05 prostaglandin E_1

Outcome	Placebo	Intervention	Absolute effect (95% CI)	Relative risk (95% CI)
Mortality (short term) (NOTE1	380 / 1000	407 / 1000 (342 to 483)	27 more per 1000 (from 38 fewer to 103 more)	**RR 1.07** (0.90 to 1.27)
	190 / 1000	203 / 1000 (171 to 241)	13 more per 1000 (from 19 fewer to 51 more)	
	450 / 1000	482 / 1000 (405 to 572)	32 more per 1000 (from 45 fewer to 122 more)	
Significant adverse events	161 / 1000	334 / 1000 (180 to 617)	172 more per 1000 (from 19 more to 456 more)	**RR 2.07** (1.12 to 3.83)
	20 / 1000	41 / 1000 (22 to 77)	21 more per 1000 (from 2 more to 57 more)	
	240 / 1000	497 / 1000 (269 to 919)	257 more per 1000 (from 29 more to 679 more)	

Summary：prostaglandin E_1 had no effect on mortality (short), but significantly increased the rate of significant adverse events. Certainty of the evidence「MODERATE」

CQ13-06 statin

Outcome	Placebo	Intervention	Absolute effect (95% CI)	Relative risk (95% CI)
Mortality (short term) (NOTE1	257 / 1000	252 / 1000 (182 to 349)	5 fewer per 1000 (from 75 fewer to 93 more)	**RR 0.98** (0.71 to 1.36)
	190 / 1000	186 / 1000 (135 to 258)	4 fewer per 1000 (from 55 fewer to 68 more)	
	450 / 1000	441 / 1000 (320 to 612)	9 fewer per 1000 (from 131 fewer to 162 more)	
Significant adverse events	74 / 1000	101 / 1000 (51 to 198)	27 more per 1000 (from 23 fewer to 124 more)	**RR 1.36** (0.69 to 2.67)
	20 / 1000	27 / 1000 (14 to 53)	7 more per 1000 (from 6 fewer to 33 more)	
	240 / 1000	326 / 1000 (166 to 641)	86 more per 1000 (from 74 fewer to 401 more)	

Summary：statin had no effect on mortality (short) or the rate of significant adverse events. Certainty of the evidence「LOW」

CQ13-07 surfactant

Outcome	Placebo	Intervention	Absolute effect (95% CI)	Relative risk (95% CI)
Mortality (short term) (NOTE1	304 / 1000	298 / 1000 (268 to 332)	6 fewer per 1000 (from 27 more to 37 fewer)	**RR 0.98** (0.88 to 1.09)
	190 / 1000	186 / 1000 (167 to 207)	4 fewer per 1000 (from 17 more to 23 fewer)	
	450 / 1000	441 / 1000 (396 to 491)	9 fewer per 1000 (from 41 more to 54 fewer)	
Significant adverse events	195 / 1000	280 / 1000 (193 to 407)	86 more per 1000 (from 2 fewer to 212 more)	**RR 1.44** (0.99 to 2.09)
	20 / 1000	29 / 1000 (20 to 42)	9 more per 1000 (from 0 fewer to 22 more)	
	240 / 1000	346 / 1000 (238 to 502)	106 more per 1000 (from 2 fewer to 262 more)	

Summary：surfactant had no effect on mortality (short) or the rate of significant adverse events. Certainty of the evidence「VERY LOW」

CQ13-08 activated protein C

Outcome	Placebo	Intervention	Absolute effect (95% CI)	Relative risk (95% CI)
Mortality (short term) （NOTE1	158 / 1000	101 / 1000 (33 to 308)	57 fewer per 1000 (from 125 fewer to 150 more)	**RR 0.64** (0.21 to 1.95)
	190 / 1000	122 / 1000 (40 to 371)	68 fewer per 1000 (from 150 fewer to 181 more)	
	450 / 1000	288 / 1000 (95 to 878)	162 fewer per 1000 (from 356 fewer to 428 more)	
Significant adverse events	263 / 1000	205 / 1000 (113 to 368)	58 fewer per 1000 (from 105 more to 150 fewer)	**RR 0.78** (0.43 to 1.40)
	20 / 1000	16 / 1000 (9 to 28)	4 fewer per 1000 (from 8 more to 11 fewer)	
	240 / 1000	187 / 1000 (103 to 336)	53 more per 1000 (from 96 more to 137 fewer)	

Summary : activated protein C had no effect on mortality (short) or the rate of significant adverse events. Certainty of the evidence「MODERATE」

CQ13-09 N-acetylcystein

Outcome	Placebo	Intervention	Absolute effect (95% CI)	Relative risk (95% CI)
Mortality (short term) （NOTE1	444 / 1000	396 / 1000 (280 to 556)	49 fewer per 1000 (from 111 more to 164 fewer)	**RR 0.89** (0.63 to 1.25)
	190 / 1000	169 / 1000 (120 to 238)	21 fewer per 1000 (from 48 more to 70 fewer)	
	450 / 1000	401 / 1000 (284 to 563)	49 fewer per 1000 (from 113 more to 167 fewer)	
Significant adverse events	No studies			

Summary N-acetylcystein had no effect on mortality (short). Certainty of the evidence「MODERATE」

CQ13-10 ketoconazole

Outcome	Placebo	Intervention	Absolute effect (95% CI)	Relative risk (95% CI)
Mortality (short term) (NOTE1	342 / 1000	349 / 1000 (246 to 499)	7 more per 1000 (from 96 fewer to 157 more)	**RR 1.02** (0.72 to 1.46)
	190 / 1000	194 / 1000 (137 to 277)	4 more per 1000 (from 53 fewer to 87 more)	
	450 / 1000	459 / 1000 (324 to 657)	9 more per 1000 (from 126 fewer to 207 more)	
Significant adverse events	171 / 1000	214 / 1000 (126 to 362)	43 more per 1000 (from 44 fewer to 191 more)	**RR 1.25** (0.74 to 2.12)
	20 / 1000	25 / 1000 (15 to 42)	5 more per 1000 (from 5 fewer to 22 more)	
	240 / 1000	300 / 1000 (178 to 509)	60 more per 1000 (from 62 fewer to 269 more)	

Summary：ketoconazole had no effect on mortality (short) or the rate of significant adverse events. Certainty of the evidence「MODERATE」

CQ13-11 lisofylline

Outcome	Placebo	Intervention	Absolute effect (95% CI)	Relative risk (95% CI)
Mortality (short term) (NOTE1	244 / 1000	319 / 1000 (212 to 483)	76 more per 1000 (from 32 fewer to 239 more)	**RR 1.31** (0.87 to 1.98)
	190 / 1000	249 / 1000 (165 to 376)	59 more per 1000 (from 25 fewer to 186 more)	
	450 / 1000	590 / 1000 (392 to 891)	140 more per 1000 (from 59 fewer to 441 more)	
Significant adverse events	No studies			

Summary lisofylline had no effect on mortality (short). Certainty of the evidence「MODERATE」

RESOURCES REQUIRED

How large are the resource requirements (costs)?

○Large costs
○Moderate costs
○Negligible costs and savings
●Moderate savings
○Large savings

○Varies
○Don't know

Inhaled NO and aerosolized β_2 stimulant require special equipment for administration. Intravenous β_2 stimulant, PGE_1, APC, NAC, and lisofylline require a minimal amount of special equipment for intravenous administration. GM-CSF requires a minimal amount of special equipment for intravenous and subcutaneous administration. Statin requires no special equipment for oral administration. Intra-tracheal administration of surfactant requires no special equipment. Ketoconazole requires special equipment for enteral administration through the stomach, duodenum or jejunum. These drugs incur an additional cost for purchase.

inhaled NO	(not covered by the national health insurance system in Japan)
inhaled β_2 stimulant	30 JPY/day (not covered by the national health insurance system in Japan)
intravenous β_2 stimulant	160 yen/day (not covered by the national health insurance system in Japan)
GM-CSF	(not sold in Japan)
PGE_1	~ 10,000 JPY/day (not covered by national health insurance in Japan)
Statin	110 JPY/day (not covered by the national health insurance system in Japan)
Surfactant	92,000 JPY/day (not covered by the national health insurance system in Japan)

<table>
<tr><td></td><td></td><td></td><td>APC 1,280,000 JPY/day (not covered by the national health insurance system in Japan)
NAC (not sold for intravenous administration in Japan)
Ketoconazole (not sold in Japan)
Lisofylline (not sold in Japan)</td><td></td></tr>
<tr><td></td><td>Does the cost effectiveness of the intervention favor the intervention or the comparison?</td><td>○Favors the comparison
●Probably favors the comparison
○Does not favor either the intervention or the comparison
○Probably favors the intervention
○Favors the intervention

○Varies
○No included studies</td><td>Since the efficacy of these drugs has not been proven, overall costs will increase by the amount of the drug.</td><td></td></tr>
<tr><td>EQUITY</td><td>What would be the impact on health equity?</td><td>○Reduced
○Probably reduced
○Probably no impact
●Probably increased
○Increased

○Varies
○Don't know</td><td>· Inhaled NO requires special medical facilities and equipment. Aerosolized β_2 stimulant requires special equipment for administration during mechanical ventilation. GM-CSF, intravenous NAC, ketoconazole, and lisofylline are not available in Japan. Surfactant is generally not used for the treatment of adult patients. APC has not been widely used. Therefore, these eight drugs are likely to have a significant impact on health inequity.
· Intravenous β_2 stimulant, PGE1, and statins are predicted to have a small effect on health inequity, since no special medical facilities or equipment are required.</td><td></td></tr>
<tr><td>ACCEPTABILITY</td><td>Is the option acceptable to key stakeholders?</td><td>○No
○Probably no
○Probably yes
○Yes

●Varies
○Don't know</td><td>· Inhaled NO has not been covered by the national health insurance system in Japan and requires special equipment. GM-CSF, intravenous NAC, ketoconazole, and lisofylline are not available in Japan. Surfactant and APC are very expensive and not widely used. Therefore, these seven drugs are not likely to be easily accepted by key stakeholders.
· Although aerosolized β_2 stimulant is commonly used in intensive care, it may or may not be acceptable to key stakeholders, since special medical equipment is required during mechanical ventilation.
· Although Intravenous β_2 stimulant is commonly used in intensive care, the use of intravenous β_2 stimulant is unlikely to be accepted in general hospitals based on the need for strict circulatory system monitoring. It may or may not be accepted by key stakeholders.
· PGE1 and statin are likely to be accepted by key stakeholders since they are widely available in Japan.</td><td></td></tr>
<tr><td>FEASIBILITY</td><td>Is the intervention feasible implement?</td><td>○No
○Probably no
○Probably yes
○Yes

●Varies
○Don't know</td><td>· PGE_1 and APC can be administered intravenously, while statin is administered orally. The use of these drugs is feasible, since no special medical facilities or equipment are required.
· Intravenous β_2 stimulant can be administered intravenously, but may require special equipment including intravenous infusion pumps. Therefore, it is unclear whether they will be accepted in all general hospitals.
· Inhaled NO is inhaled with a special device. Aerosolized β_2 stimulant requires a special medical device, particularly during mechanical ventilation. Therefore, these two drugs cannot be used in all medical facilities.
· Because surfactant can be administered intra-tracheally, the use of surfactant is limited to special facilities where sufficient respiratory monitoring and management can be provided. It is not practical to use surfactant in all medical facilities.
· GM-CSF, NAC, ketoconazole, and lisofylline are not available in Japan. The use of these drugs is not feasible.</td><td></td></tr>
</table>

Evidence-to-Decision Table

CQ13 : SHOULD THE FOLLOWING DRUGS BE USED FOR ADULT PATIENTS WITH ARDS?
(inhaled nitric oxide (NO), inhaled/ intravenous beta$_2$ stimulant, granulocyte macrophage colony-stimulating factor (GM-CSF), prostaglandin E_1 (PGE_1), statin, surfactant, activated protein C (APC), N-acetylcysteine (NAC), ketoconazole, and lisofylline)

**Balance of consequences**	Undesirable consequences clearly outweigh desirable consequences in most settings	Undesirable consequences probably outweigh desirable consequences in most settings	The balance between desirable and undesirable consequences is closely balanced or uncertain	Desirable consequences probably outweigh undesirable consequences in most settings	Desirable consequences clearly outweigh undesirable consequences in most settings
Judgement	○	○	●	○	○

**Type of recommendation**	Strong recommendation against the intervention	Conditional recommendation against the intervention	Conditional recommendation for either the intervention or the comparison	Conditional recommendation for the intervention
Judgement	○	●	○	○

Recommendation

Recommendation:
We do not recommend using the following drugs to treat adult patients with ARDS (strength of recommendation "strong recommendation").
GRADE 1B Inhaled/ intravenous β_2 stimulant, prostaglandin E_1 (PGE_1), activated protein C (APC), ketoconazole, and lisofylline (Quality of evidence "moderate")
GRADE 1C Inhaled nitric oxide (NO) (Quality of evidence "low")
GRADE 1D Surfactant (Quality of evidence "very low")

We do not suggest using the following drugs to treat adult patients with ARDS (strength of recommendation "weak recommendation").
GRADE 2B granulocyte macrophage colony-stimulating factor (GM-CSF), N-acetylcysteine (NAC) (Quality of evidence "moderate")
GRADE 2C Statin (Quality of evidence "low")

●Supplementary conditions: These drugs are not approved for clinical use by the Japanese national health insurance system.

Justification

Clinical question: Should the following drugs be used to treat adult patients with ARDS? (ref. Intervention)
Patient or population : Adult patients with ARDS
Intervention : inhaled nitric oxide (NO), inhaled/ intravenous beta$_2$ stimulant, granulocyte macrophage colony-stimulating factor (GM-CSF), prostaglandin E_1 (PGE_1), statin, surfactant, activated protein C (APC), N-acetylcysteine (NAC), ketoconazole, and lisofylline
Comparison : placebo
Outcomes: Mortality (short term) [Note 1], Significant adverse events

Summary of the evidence:

1. A total of 7 RCTs (699 patients) evaluating the efficacy of inhaled NO were selected in a systematic review. Meta-analysis demonstrated that inhaled NO is not associated with improvement in short-term (<90 days) mortality (RR 1.18, 95%CI 0.91-1.52) or the rate of significant adverse events (RR 1.90, 95%CI 0.78-4.66).
2. A total of 1 RCT (282 patients) evaluating the efficacy of inhaled β_2 stimulant was selected in a systematic review. Meta-analysis demonstrated that inhaled β_2 stimulant is not associated with an improvement in short-term (<90 days) mortality (RR 1.32, 95%CI 0.83-2.08), or the rate of significant adverse events (RR 2.57, 95%CI 0.85-7.76).
3. A total of 2 RCTs (365 patients) evaluating the efficacy of intravenous β_2 stimulant were included in a systematic review. Meta-analysis showed that intravenous β_2 stimulant is not associated with improvement in short-term (<90 days) mortality (RR 1.16, 95%CI 0.68-1.96). The rate of significant adverse events with administration of intravenous β_2 stimulant was significantly increased (RR 5.78, 95%CI 1.34-24.92).
4. A total of 2 RCTs (148 patients) evaluating the efficacy of GM-CSF were selected for analysis in a systematic review. The meta-analysis showed that GM-CSF is not associated with an improvement in short-term (<90 days) mortality (RR 0.76, 95%CI 0.40-1.46), or rate of significant adverse events (RR 0.87, 95%CI 0.42-1.80).
5. A total of 8 RCTs (786 patients) evaluating the efficacy of PGE_1 administration were included in a systematic review. The meta-analysis demonstrated that PGE_1 is not associated with an

improvement in short-term (<90 days) mortality (RR 1.07, 95%CI 0.90-1.27). However, PGE_1 is significantly associated with an increase in the rate of significant adverse events (RR 2.07, 95%CI 1.12-3.83).

6. A total of 2 RCTs (1,284 patients) evaluating the efficacy of statin were selected for analysis in a systematic review. The meta-analysis demonstrated that statin did not have beneficial effects in terms of short-term (<90 days) mortality (RR 0.98, 95%CI 0.71-1.36) or the rate of significant adverse events (RR 1.36, 95%CI 0.69-2.67).
7. A total of 10 RCTs (2,894 patients) evaluating the efficacy of surfactant were included in a systematic review. The meta-analysis demonstrated that surfactant is not associated with an improvement in short-term (<90 days) mortality (RR 0.98, 95%CI 0.88-1.09), or rate of significant adverse events (RR 1.44, 95%CI 0.99-2.09).
8. A total of 2 RCTs (146 patients) evaluating the efficacy of APC were selected for analysis in a systematic review. The meta-analysis demonstrated that APC had no beneficial effects on short-term (<90 days) mortality (RR 0.64, 95%CI 0.21-1.95), or the rate of significant adverse events (RR 0.78, 95%CI 0.43-1.40).
9. A total of 4 RCTs (180 patients) evaluating the efficacy of NAC were included in a systematic review. The meta-analysis demonstrated that NAC is not associated with improvement in short-term (<90 days) mortality (RR 0.89, 95%CI 0.63-1.25). No RCT evaluated the rate of significant adverse events with the use of NAC.
10. A total of 1 RCT (234 patients) evaluating the efficacy of ketoconazole was selected for analysis in a systematic review. The meta-analysis demonstrated that ketoconazole did not improve the short-term (<90 days) mortality (RR 1.02, 95%CI 0.72-1.46), or the rate of significant adverse events (RR 1.25, 95%CI 0.74-2.12).
11. A total of 1 RCTs (235 patients) evaluating the efficacy of lisofylline was selected in a systematic review. The meta-analysis demonstrated that lisofylline had beneficial effects in terms of short-term (<90 days) mortality (RR 1.31, 95%CI 0.87-1.98). No RCT evaluated the rate of significant adverse events associated with the use of lisofylline.

Quality of evidence:

1. Regarding inhaled nitric oxide, a majority of studies had a high risk for bias in blinding with regard to short-term (<90 days) mortality. The risk of bias for the occurrence of significant adverse events was not high. No inconsistency was observed in analysis of short-term (<90 days) mortality (I^2 = 0%) or significant adverse effects (I^2 = 0%,). No indirectness was observed. Since the number of patients was smaller than the optimal information size and therefore the 95%CI was large, the imprecision of this meta-analysis was high. Publication bias could not be determined because of the small number of reported studies.
2. Regarding inhaled β_2 stimulant, no risk of bias was observed. Inconsistency could not be evaluated because of the small number of reported studies. No indirectness was observed. Since the number of patients was smaller than the optimal information size and therefore 95%CI was large, the imprecision of this meta-analysis was high. Publication bias could not be determined because of the small number of reported studies.
3. Regarding intravenous β_2 stimulant, no risk of bias was observed. Inconsistency ranged from moderate to severe (short-term (<90 days) mortality, I^2 = 68%; severe adverse events, I^2 = 49%, respectively). No indirectness was observed. Since the number of patients was smaller than the optimal information size and therefore 95%CI was large, the imprecision of this meta-analysis was high. Publication bias could not be determined because of the small number of reported studies.
4. Regarding GM-CSF, no risk of bias was observed. No inconsistency was observed in short-term (<90 days) mortality (I^2 = 0%). Inconsistency in significant adverse events could not be determined because of the small number of reported studies (only one RCT included). No indirectness was observed. Since the number of patients was smaller than the optimal information size and therefore 95%CI was large, the imprecision of this meta-analysis was high. Publication bias could not be determined because of the small number of reported studies.
5. Regarding PGE_1, no risk of bias was observed. No inconsistency was observed in short-term (<90 days) mortality (I^2 = 0%), while moderate inconsistency was observed in significant adverse events (I^2 = 45%). No indirectness was observed. Since the number of patients was smaller than the optimal information size and therefore 95%CI was large, the imprecision of this meta-analysis was high. Publication bias could not be determined because of the small number of reported studies.
6. Regarding statin, no risk of bias was observed. Moderate to severe inconsistency was observed (short-term (<90 days) mortality, I^2 = 65%; significant adverse events, I^2 = 35%, respectively). No indirectness was observed. Since the number of patients was smaller than the optimal information size and therefore 95%CI was large, the imprecision of this meta-analysis was high. Publication bias could not be determined because of the small number of reported studies.
7. Regarding surfactant, high risk of bias was observed in blinding of short-term (<90 days) mortality. No risk of bias was observed in the rate of significant adverse events. No inconsistency was observed in short-term (<90 days) mortality (I^2 = 0%), while severe inconsistency was observed in the rate of significant adverse events (I^2 = 71%). No indirectness was observed. Since the number of patients was smaller than the optimal information size and therefore 95%CI was large, the imprecision of this meta-analysis was high. Publication bias could not be determined because of the small number of reported

	studies.<br>8. Regarding APC, no risk of bias was observed in short-term (<90 days) mortality, while high risk of bias was observed in blinding and concealment of significant adverse events. Mild inconsistency was observed (short-term [<90 days] mortality, I^2 = 29%; severe adverse events, I^2 = 1%, respectively). No indirectness was observed. Since the number of patients smaller than the optimal information size and therefore 95%CI was large, the imprecision of this meta-analysis was high. Publication bias could not be determined because of the small number of reported studies.<br>9. Regarding NAC, no risk of bias was observed. No inconsistency was observed (short-term (<90 days) mortality, I^2 = 0%). No indirectness was observed. Since the number of patients was smaller than the optimal information size and therefore 95%CI was large, the imprecision of this meta-analysis was high. Publication bias could not be determined because of the small number of reported studies.<br>10. Regarding ketoconazole, no serious risk of bias was observed. Inconsistency could not be determined because of the small number of reported studies (only one RCT included). No indirectness was observed. Since the number of patients was smaller than the optimal information size and therefore 95%CI was large, the imprecision of this meta-analysis was high. Publication bias could not be determined because of the small number of reported studies.<br>11. Regarding lisofylline, no serious risk of bias was observed. Inconsistency could not be determined because of the small number of reported studies (only one RCT included). No indirectness was observed. Since the number of patients was smaller than the optimal information size and therefore 95%CI was large, the imprecision of this meta-analysis was high. Publication bias could not be determined because of the small number of reported studies.<br><br>**Judgement of benefit and harm, resources and cost:** Systematic review demonstrated that neither efficacy nor the rate of significant adverse effects was found for any drug except intravenous β_2 stimulant and PGE_1. The benefit was considered to be small compared to the increase in cost. However, intravenous β_2 stimulant and PGE_1 are associated with an increase in the rate of significant adverse events. With these medications, the benefit was considered to be small compared to the increase in cost.<br><br>**Recommendations:**<br>We do not recommend using the following drugs to treat adult patients with ARDS (strength of recommendation “strong recommendation”).<br>GRADE 1B Inhaled/ intravenous β_2 stimulant, prostaglandin E_1 (PGE_1), activated protein C (APC), ketoconazole, and lisofylline (Quality of evidence ”moderate”)<br>GRADE 1C Inhaled nitric oxide (NO) (Quality of evidence ”low”)<br>GRADE 1D Surfactant (Quality of evidence ”very low”)<br><br>We do not suggest using the following drugs to treat adult patients with ARDS (strength of recommendation “weak recommendation”).<br>GRADE 2B granulocyte macrophage colony-stimulating factor (GM-CSF), N-acetylcysteine (NAC) (Quality of evidence ”moderate”)<br>GRADE 2C Statin (Quality of evidence ”low”)<br><br>**Additional Considerations:** These drugs are not approved for clinical use by the Japanese national health insurance system.
**Subgroup considerations**	None
**Implementation considerations**	• Inhaled NO requires special facility and equipment.<br>• Inhaled β_2 stimulant requires uncommon equipment during mechanical ventilation.<br>• GM-CSF, NAC, ketoconazole and lisofylline are uncommon drugs that are not available in Japan.<br>• Surfactant is an uncommon drug in the treatment of adult patients.<br>• APC is an uncommon drug.<br>• Intravenous β_2 stimulant, PGE_1 and statin do not require special facility or equipment.
**Monitoring and evaluation considerations**	Cardiorespiratory monitoring and blood testing are necessary to detect adverse events.
**Research possibilities**	Because of a limited number of high-quality RCTs, large-scale, high-quality clinical trials are necessary to evaluate the efficacy of these medications.

Note 1: Short-term (<90 days) mortality indicates death within 90 days, which was analyzed as a main outcome in each study.

References

1. Matthay MA, Ware LB, Zimmerman GA. The acute respiratory distress syndrome. *J Clin Invest* **122**(8): 2731-40, 2012. PMID 22850883
2. Adhikari NK, Burns KE, Friedrich JO, et al. Effect of nitric oxide on oxygenation and mortality in acute lung injury: systematic review and meta-analysis. *BMJ* **334**(7597): 779, 2007. PMID 17383982
3. Sakuma T, Okaniwa G, Nakada T, et al. Alveolar fluid clearance in the resected human lung. *Am J Respir Crit Care Med* **150**(2): 305-10,

1994. PMID 8049807
4. Basran GS, Hardy JG, Woo SP, et al. Beta-2-adrenoceptor agonists as inhibitors of lung vascular permeability to radiolabelled transferrin in the adult respiratory distress syndrome in man. *Eur J Nucl Med* **12**(8): 381-4, 1986. PMID 2878809
5. Sartori C, Allemann Y, Duplain H, et al. Salmeterol for the prevention of high-altitude pulmonary edema. *N Engl J Med* **346**(21): 1631-6, 2002. PMID 12023995
6. Atabai K, Ware LB, Snider ME, et al. Aerosolized beta(2)-adrenergic agonists achieve therapeutic levels in the pulmonary edema fluid of ventilated patients with acute respiratory failure. *Intensive Care Med* **28**(6): 705-11, 2002. PMID 12107675
7. Trapnell BC, Whitsett JA. Gm-CSF regulates pulmonary surfactant homeostasis and alveolar macrophage-mediated innate host defense. *Annu Rev Physiol* **64**: 775-802, 2002. PMID 11826288
8. Huffman Reed JA, Rice WR, Zsengeller ZK, et al. GM-CSF enhances lung growth and causes alveolar type II epithelial cell hyperplasia in transgenic mice. *Am J Physiol* **273**(4 Pt 1): L715-25, 1997. PMID 9357845
9. Eierman DF, Yagami M, Erme SM, et al. Endogenously opsonized particles divert prostanoid action from lethal to protective in models of experimental endotoxemia. *Proc Natl Acad Sci U S A* **92**(7): 2815-9, 1995. PMID 7708730
10. Rossetti RG, Brathwaite K, Zurier RB. Suppression of acute inflammation with liposome associated prostaglandin E1. *Prostaglandins* **48**(3): 187-95, 1994. PMID 7809384
11. Terblanche M, Almog Y, Rosenson RS, et al. Statins: panacea for sepsis? *Lancet Infect Dis* **6**(4): 242-8, 2006. PMID 16554249
12. Jacobson JR, Barnard JW, Grigoryev DN, et al. Simvastatin attenuates vascular leak and inflammation in murine inflammatory lung injury. *Am J Physiol Lung Cell Mol Physiol* **288**(6): L1026-32, 2005. PMID 15665042
13. Lelcuk S, Huval WV, Valeri CR, et al. Inhibition of ischemia-induced thromboxane synthesis in man. *J Trauma* **24**(5): 393-6, 1984. PMID 6325720
14. Williams JG, Maier RV. Ketoconazole inhibits alveolar macrophage production of inflammatory mediators involved in acute lung injury (adult respiratory distress syndrome). *Surgery* **112**(2): 270-7, 1992. PMID 1322565
15. Bursten SL, Federighi D, Wald J, et al. Lisofylline causes rapid and prolonged suppression of serum levels of free fatty acids. *J Pharmacol Exp Ther* **284**(1): 337-45, 1998. PMID 9435196
16. Rice GC, Rosen J, Weeks R, et al. CT-1501R selectively inhibits induced inflammatory monokines in human whole blood ex vivo. *Shock* **1**(4): 254-66, 1994. PMID 7735959
17. Cornet AD, van Nieuw Amerongen GP, Beishuizen A, et al. Activated protein C in the treatment of acute lung injury and acute respiratory distress syndrome. *Expert Opin Drug Discov* **4**(3): 219-27, 2009. PMID 23489122
18. Bernard GR, Vincent JL, Laterre PF, et al. Efficacy and safety of recombinant human activated protein C for severe sepsis. *N Engl J Med* **344**(10): 699-709, 2001. PMID 11236773
