# Supplementary material for: The clinical practice guideline for the management of ARDS in Japan
Source: J Intensive Care. 2017 Jul 25;5:50. doi: 10.1186/s40560-017-0222-3 (PMC5526253; doi:10.1186/s40560-017-0222-3)
Supplement: Supplementary file 2 — contains tables disclosing intellectual and financial conflicts of interest for each person who participated in the creation of this guideline. (PDF 76 kb) [file 40560_2017_222_MOESM2_ESM.pdf]

Role of each member, and financial and academic COI

	Systematic review	Panel member	Financial COI	Academic COI
● Core members				
Satoru Hashimoto	-	Chair person	None	Senior Editor, Journal of Intensive Care<br>JRS *1, ALI/ARDS clinical practice guideline member
Masamitsu Sanui	-	CQ1-CQ13	None	None
Moritoki Egi	CQ1*, CQ12*	-	None	JSICM*2, member for clinical practice guideline for sepsis<br>JSICM*2, member for clinical practice guideline for nutrition management
Sinichiro Ohshimo	CQ9*, CQ11*, CQ13*	CQ1-CQ8, CQ10, CQ12	None	None
Junji Shiotsuka	CQ3		None	None
Ryutaro Seo	CQ2*, CQ4	CQ1, CQ3, CQ5-CQ13	None	None
Ryoma Tanaka	CQ8	-	None	None
Yu Tanaka	CQ4, CQ10	-	None	None
Yasuhiro Norisue	CQ5*, CQ6*	-	None	None
Yoshiro Hayashi	CQ7	-	None	None
Eishu Nango		CQ1-CQ13	None	Support member for developing clinical practice guideline, MIND*3 guideline center, JCQHC*4<br>Member of GRADE working group
● Systematic review committee members				
Yoshitaka Aoki	CQ1, CQ10*	-	None	None
Kohkichi Andoh	CQ8	-	None	None
Yusuke Iizuka	CQ3*, CQ9	-	None	None
Hitoshi Imaizumi	CQ7	-	None	JSICM*2, member for clinical practice guideline for sepsis
Satoshi Okamori	CQ5	-	None	None
Motoshi Kainuma	CQ1	-	None	None
Jun Kataoka	CQ6	-	None	None
Tetsuro Kamo	CQ4	-	None	None
Atsushi Kawaguchi	CQ2, CQ3, CQ5, CQ8	-	None	None
Junji Kumasawa	CQ2	-	None	None
Kiyoyasu Kurahashi	CQ5, CQ7	-	None	None
Kunihiko Kooguchi	CQ3	-	None	None
Yutaka Kondo	CQ2	-	None	None
Masaaki Sakuraya	CQ10*, CQ11, CQ13	CQ4, CQ9	None	None
Akira Shimoyama	CQ12	-	None	None
So Suzuki	CQ12	-	None	None
Hiroyuki Suzuki	CQ6	-	None	None
Motohiro Sekino	CQ6, CQ11, CQ13	-	None	None
Mikio Nakajima	CQ9	-	None	None
Tetsuro Nishimura	CQ4	-	None	None
Tatsuma Fukuda	CQ1, CQ7*	-	None	None
Jun Makino	CQ3, CQ9	-	None	None
Ryoichi Miyashita	CQ11, CQ13	-	None	None
Ryutaro Moriwaki	CQ12	-	None	None
Hideto Yasuda	CQ4*, CQ8*	CQ2, CQ11	None	None

Shigenori Yoshitake	CQ5	-	None	None
Yumi Yamashita	CQ1-CQ13	-	None	None
Yoshiko Nakagawa	CQ1-CQ13	-	None	None
Takaaki Suzuki	CQ1-CQ13	-	None	None
Toshiyuki Aokage	-	-	None	None
Kimitaka Tajimi	-	-	None	JSRCM *5 ARDS clinical practice guideline member
Hidemichi Yuasa	-	-	None	None
● Panelists				
Kazuya Ichikado	-	CQ1-CQ13	None	JRS *1ALI/ARDS clinical practice guideline member
Hideaki Imanaka	-	CQ1-CQ13	None	JRS *1ALI/ARDS clinical practice guideline member<br>JRS *1 NPPV clinical practice guideline member
Ayumu Nozaki	-	CQ1-CQ13	None	None
Ryo Kozu	-	CQ1-CQ13	None	JSICM J-PAD *6 clinical practice guideline member
Takeshi Unoki	-	CQ1-CQ13	None	None
Yoshinori Takahashi	-	CQ1-CQ13	None	None
Akimichi Serita	-	CQ1-CQ13	None	None
Eriko Takezawa	-	CQ1-CQ13	None	None
● External evaluation committee members				
Morio Aihara	-	-	None	Member of GRADE working group
Taku Yabuki	-	-	None	None
Takeo Nakayama	-	-	None	None
Toshio Fukuoka	-	-	None	None

Eight panelists were recruited from public announcement. Four core members and two systematic review members also participated in panel conference as a panelist. If they participated in certain systematic review, he/she was replace by other members as show in the table.

*: Leader of each CQ's systematic review
*1: JRS, Japanese Respiratory Society
*2: JSICM, Japanese Society of Intensive Care Medicine
*3: MIND, Medical Information Network Distribution Service
*4: JCQHC, Japan Council for Quality Health Care
*5: JSRCM, J Japanese Society of Respiratory Care Medicine
*6: J-PAD, Japanese Pain, Agitation, and Delirium
